# Supplementary material for: Nickel-catalysed migratory hydroalkynylation and enantioselective hydroalkynylation of olefins with bromoalkynes
Source: Nat Commun. 2021 Jun 18;12:3792. doi: 10.1038/s41467-021-24094-9 (PMC8213830; doi:10.1038/s41467-021-24094-9)
Supplement: Supplementary file 1 — Supplementary Information [file 41467_2021_24094_MOESM1_ESM.pdf]

Supplementary Information

Nickel-catalysed migratory hydroalkynylation and enantioselective hydroalkynylation of olefins with bromoalkynes

Jiang et al.

Supplementary Methods

General reagent information. Solvents were either purified and dried by passage through alumina and Q5 reactant-packed columns on a solvent purification system or bought from the commercial sources and transferred to the glovebox without exposure to air. Other commercial reagents were purchased from Sigma-Aldrich, Acros, Alfa Aesar, TCI, Aladdin, J&K, Energy Chemical, Bide Pharmatech Ltd. and were used as received. Flash chromatography was performed using glass columns with silica gel (*SiliaFlash*® P60, particle size 40-63 µm, Silicycle).

$NiBr_2$·diglyme (CAS 312696-09-6) was purchased from Sigma-Aldrich;
$NiI_2 \cdot xH_2O$ (CAS 7790-34-3) was purchased from Strem Chemical;
Na_2CO_3 (CAS 497-19-8) was purchased from Alfa Aesar;
$K_3PO_4 \cdot H_2O$ (CAS 27176-10-9) was purchased from Sigma-Aldrich;
NaI (CAS 7681-82-5) was purchased from Alfa Aesar (ACS, 99.0% min.);
DME (CAS 110-71-4, 99.9%, extra dry, with molecular sieves, water≤30 ppm (by K.F.), EnergySeal) was purchased from Energy Chemical;
Benzotrifluoride ($PhCF_3$, CAS 98-08-8, 99%, SuperDry, water≤10 ppm, J&K Seal) was purchased from J&K;
Diglyme (CAS 111-96-6, 99.5%, SuperDry, J&K Seal) was purchased from J&K;
Bathocuproine (CAS 4733-39-5) was purchased from Energy Chemical;
4-Phenyl-1-butene (CAS 768-56-9) was purchased from TCI;
PMHS (CAS 63148-57-2, poly(methylhydrosiloxane)) was purchased from Sigma-Aldrich and stored under nitrogen at –20 °C in glove box;
$(MeO)_3SiH$ (CAS 2487-90-3) was purchased from Energy Chemical and stored under nitrogen at –20 °C in glove box;

Safety note: MSDS indicates that $(MeO)_3SiH$ is a corrosive and flammable liquid. According to the literatures[1,2], it may form pyrophoric gas (possibly SiH_4) during the storage or reaction. Although during our reaction, we used $(MeO)_3SiH$ without incident and SiH_4 was not observed, we urge the users of these procedures to be alert to the possibility of SiH_4 formation and possible exotherms and to take suitable precautions (suitable eye protection is also required).

General analytical information. All compounds (starting materials and products) were characterized by ^1H NMR, ^{13}C NMR, IR spectroscopy and high-resolution mass spectrometry. ^1H NMR spectra were recorded on Bruker 500 MHz spectrometer and are referenced relative to residual $CDCl_3$ proton signals at δ 7.26 ppm. ^{19}F NMR spectra were recorded on a Bruker 500 MHz spectrometer and are referenced to $CFCl_3$ (δ 0.0

ppm). Data for ^{1}H and ^{19}F NMR are reported as follows: chemical shift (δ ppm), multiplicity (s = singlet, d = doublet, t = triplet, q = quartet, m = multiplet, br = broad), integration, and coupling constant (Hz). ^{13}C NMR spectra were recorded on a Bruker 500 MHz spectrometer and are referenced to $CDCl_3$ at δ 77.16 ppm. The ^{13}C NMR spectra were obtained with ^{1}H decoupling. Data for ^{13}C NMR are reported in terms of chemical shift and multiplicity where appropriate. IR spectra were obtained on a Bruker Alpha and was reported in terms of frequency of absorption (cm^{-1}). GC analyses were performed on Agilent 7890 or 8890 gas chromatograph with an FID detector using a J&W DB-1 column (10 m, 0.1 mm I.D.). Low Resolution Mass spectra were obtained from on an Agilent 5977A GC-MS. r.r. refers to regioisomeric ratio, representing the ratio of the terminal coupling product to the sum of all other isomers as determined by GC and GC-MS analysis. High Resolution Mass spectra were obtained from on an Agilent 6540 Q-TOF mass spectrometer, operating electrospray ionization (ESI) mode. High pressure liquid chromatography (HPLC) was performed on Agilent 1260 Series chromatographs using Daicel Chiralcel columns (250 mm). Optical rotations were measured on a Rudolph Research Analytical Autopol VI automatic polarimeter using a 50 mm pathlength cell at 589 nm with $[\alpha]_D$ values reported in degrees; concentration (c) is in g/100 mL.

Medium-sized screw-cap test tubes (8 mL) were used for all 0.20 mmol scale reactions:

a Fisher 13×100 mm tube (Cat. No. 14-959-35C)

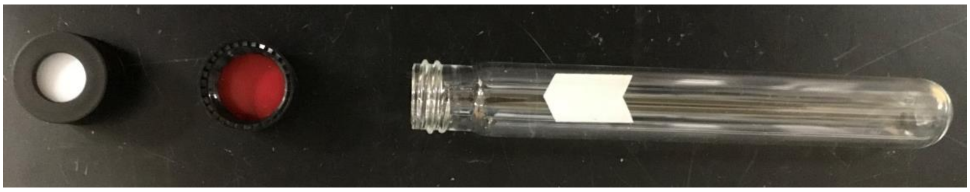

b Cap with Septa: Thermo Scientific ASM PHN CAP w/PTFE/SIL (Cat. No. 03378316)

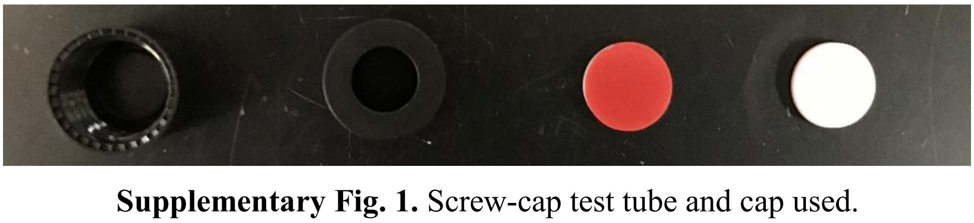

Supplementary Fig. 1. Screw-cap test tube and cap used.

NiH-Catalyzed Reductive Migratory Hydroalkynylation

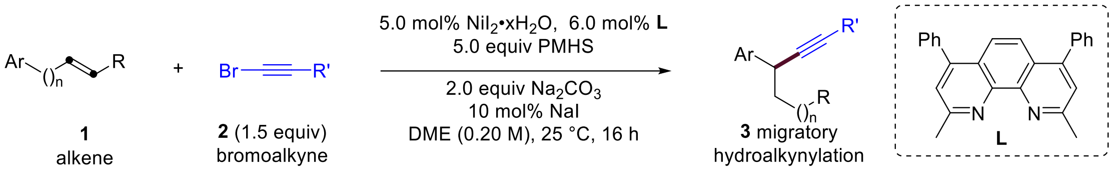

<u>General procedure (A)</u> for NiH-catalyzed reductive migratory hydroalkynylation. In a nitrogen-filled glove box, to an oven-dried 8 mL screw-cap vial equipped with a magnetic stir bar were added $NiI_2 \cdot xH_2O$ (3.8 mg, 5.0 mol%), **L** (4.3 mg, 6.0 mol%), Na_2CO_3 (42.4 mg, 2.0 equiv), NaI (3.0 mg, 10.0 mol%) and anhydrous DME (1.0 mL). The mixture was stirred for 20 min at room temperature (stirred at 800 rpm) before the addition of PMHS (60 μL, 1.0 mmol, 5.0 equiv). Stirring was continued for an additional 5 min at room temperature before the addition of olefin **1** (0.20 mmol, 1.0 equiv) and bromoalkyne **2** (0.30 mmol, 1.5 equiv). The tube was sealed with a teflon-lined screw cap, removed from the glove box and the reaction was stirred at 25 °C for up to 16 h (the mixture was stirred at 1000 rpm). After the reaction was complete, the reaction was quenched upon the addition of H_2O, and the mixture was extracted with EtOAc. The organic layer was concentrated to give the crude product. Dodecane (20 μL) was added as an internal standard for GC analysis. The product was purified by flash column chromatography (petroleum ether/EtOAc) for each substrate. The yields reported are the average of at least two experiments, unless otherwise indicated.

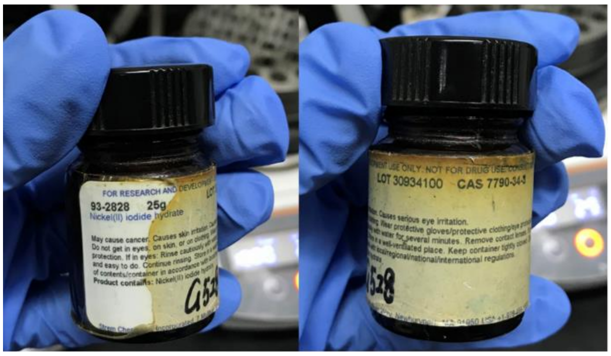

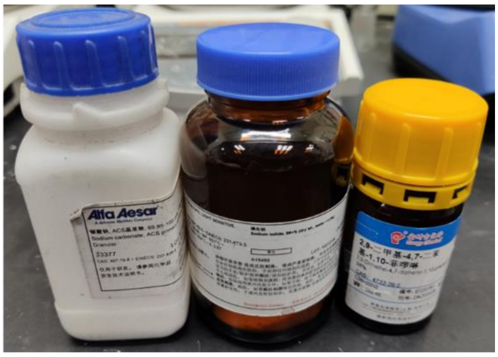

$NiI_2•xH_2O$

Na_2CO_3, NaI, **L**

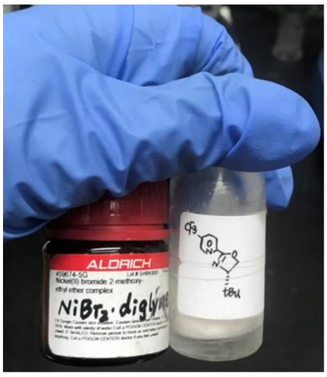

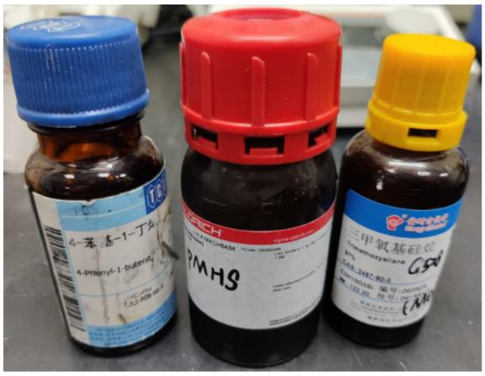

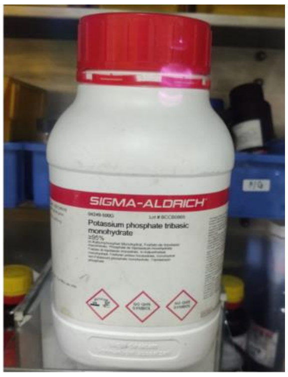

L*, $NiBr_2$•diglyme

PMHS, $(MeO)_3SiH$, 4-Phenyl-1-butene

$K_3PO_4•H_2O$

Supplementary Fig. 2. Catalyst and reagents to be used.

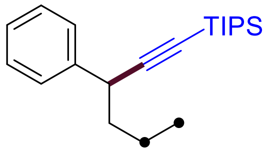

Triisopropyl(3-phenylhex-1-yn-1-yl)silane (Figure 3, **3a**). From **4-phenyl-1-butene** (**1a)** (26.4 mg, 0.20 mmol) and **(bromoethynyl)triisopropylsilane** (**2a**) (78.4 mg, 0.30 mmol), the title compound was prepared following the general procedure **A** using $NiI_2 \cdot xH_2O$ (3.8 mg, 5.0 mol%), **L** (4.3 mg, 6.0 mol%), Na_2CO_3 (42.4 mg, 2.0 equiv), NaI (3.0 mg, 10 mol%), PMHS (60.0 µL, 5.0 equiv) and anhydrous DME (1.0 mL). The reaction mixture was stirred for 16 h at 25 °C. The crude material was purified by flash column chromatography (petroleum ether) to provide the title compound as a colorless liquid in 82% yield (51.6 mg), and >99:1 rr was detected by GC.

^{1}H NMR (500 MHz, $CDCl_3$) δ 7.44 – 7.39 (m, 2H), 7.36 – 7.30 (m, 2H), 7.27 – 7.22 (m, 1H), 3.74 (dd, *J* = 7.9, 6.4 Hz, 1H), 1.78 – 1.69 (m, 2H), 1.60 – 1.46 (m, 2H), 1.20 – 1.06 (m, 21H), 0.96 (t, *J* = 7.4 Hz, 3H);

^{13}C NMR (126 MHz, $CDCl_3$) δ 142.4, 128.4, 127.6, 126.5, 110.2, 83.1, 41.4, 38.8, 20.6, 18.8, 13.9, 11.4;

HRMS (ESI) calcd. for $C_{21}H_{34}SiNa$ $[M+Na]^+$ *m/z* 337.2322, found 337.2320;

IR (neat, cm^{-1}) 2941, 2864, 2164, 1462, 1097, 663.

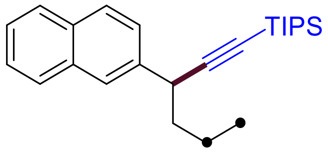

Triisopropyl(3-(naphthalen-2-yl)hex-1-yn-1-yl)silane (Figure 3, **3b**). From **2-(but-3-en-1-yl)naphthalene** (**1b**) (36.4 mg, 0.20 mmol), and **(bromoethynyl)triisopropylsilane** (**2a**) (78.4 mg, 0.30 mmol), the title compound was prepared following the general procedure **A** using $NiI_2 \cdot xH_2O$ (3.8 mg, 5.0 mol%), **L** (4.3 mg, 6.0 mol%), Na_2CO_3 (42.4 mg, 2.0 equiv), NaI (3.0 mg, 10 mol%), PMHS (60.0 µL, 5.0 equiv) and anhydrous DME (1.0 mL). The reaction mixture was stirred for 16 h at 25 °C. The crude material was purified by flash column chromatography (petroleum ether) to provide the title compound as a colorless liquid in 54% yield (39.6 mg),

and >99:1 rr was detected by GC.

^{1}H NMR (500 MHz, $CDCl_3$) δ 7.90 – 7.86 (m, 1H), 7.84 – 7.78 (m, 3H), 7.54 – 7.42 (m, 3H), 3.88 (t, *J* = 7.1 Hz, 1H), 1.88 – 1.75 (m, 2H), 1.64 – 1.39 (m, 2H), 1.19 – 1.07 (m, 21H), 0.95 (t, *J* = 7.4 Hz, 3H);

^{13}C NMR (126 MHz, $CDCl_3$) δ 139.8, 133.5, 132.4, 128.0, 127.8, 127.7, 126.2, 126.1 (2C), 125.5, 110.2, 83.5, 41.0, 38.9, 20.6, 18.8, 13.9, 11.5;

HRMS (ESI) calcd. for $C_{25}H_{36}SiNa$ $[M+Na]^+$ *m/z* 387.2478, found 387.2480;

IR (neat, cm^{-1}) 2956, 2891, 2167, 1462, 854, 744.

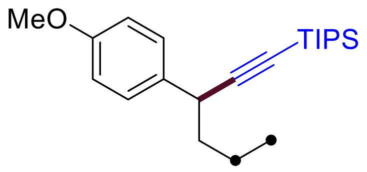

Triisopropyl(3-(4-methoxyphenyl)hex-1-yn-1-yl)silane (Figure 3, **3c**). From **1-(but-3-en-1-yl)-4-methoxybenzene** (**1c**) (32.4 mg, 0.20 mmol), and **(bromoethynyl)triisopropylsilane** (**2a**) (78.4 mg, 0.30 mmol), the title compound was prepared following the general procedure **A** using $NiI_2{\cdot}xH_2O$ (3.8 mg, 5.0 mol%), **L** (4.3 mg, 6.0 mol%), Na_2CO_3 (42.4 mg, 2.0 equiv), NaI (3.0 mg, 10 mol%), PMHS (60.0 μL, 5.0 equiv) and anhydrous diglyme (1.0 mL). The reaction mixture was stirred for 16 h at 25 °C. The crude material was purified by flash column chromatography (petroleum ether) to provide the title compound as a colorless liquid in 75% yield (51.8 mg), and >99:1 rr was detected by GC.

^{1}H NMR (500 MHz, $CDCl_3$) δ 7.30 (d, *J* = 8.6 Hz, 2H), 6.87 (d, *J* = 8.6 Hz, 2H), 3.81 (s, 3H), 3.67 (dd, *J* = 8.0, 6.3 Hz, 1H), 1.75 – 1.64 (m, 2H), 1.59 – 1.35 (m, 2H), 1.12 – 0.97 (m, 21H), 0.93 (t, *J* = 7.4 Hz, 3H);

^{13}C NMR (126 MHz, $CDCl_3$) δ 158.3, 134.6, 128.5, 113.8, 110.6, 82.8, 55.3, 41.4, 37.9, 20.5, 18.8, 13.9, 11.4;

HRMS (ESI) calcd. for $C_{22}H_{36}OSiNa$ $[M+Na]^+$ m/z 367.2428, found 367.2426;

IR (neat, cm^{-1}) 2955, 2863, 2167, 1610, 1462, 882.

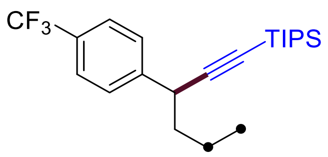

Triisopropyl(3-(4-(trifluoromethyl)phenyl)hex-1-yn-1-yl)silane (Figure 3, **3d**). From **1-(but-3-en-1-yl)-4-(trifluoromethyl)benzene** (**1d**) (40.0 mg, 0.20 mmol), and **(bromoethynyl)triisopropylsilane** (**2a**) (78.4 mg, 0.30 mmol), the title compound was prepared following the general procedure **A** using $NiI_2 \cdot xH_2O$ (3.8 mg, 5.0 mol%), **L** (4.3 mg, 6.0 mol%), Na_2CO_3 (42.4 mg, 2.0 equiv), NaI (3.0 mg, 10 mol%), PMHS (60.0 μL, 5.0 equiv) and anhydrous diglyme (1.0 mL). The reaction mixture was stirred for 16 h at 25 ºC. The crude material was purified by flash column chromatography (petroleum ether) to provide the title compound as a colorless liquid in 92% yield (70.0 mg), and >99:1 rr was detected by GC.

^{1}H NMR (500 MHz, $CDCl_3$) δ 7.58 (d, *J* = 8.1 Hz, 2H), 7.50 (d, *J* = 8.1 Hz, 2H), 3.77 (dd, *J* = 7.9, 6.5 Hz, 1H), 1.76 – 1.67 (m, 2H), 1.58 – 1.46 (m, 2H), 1.16 – 1.04 (m, 21H), 0.94 (t, *J* = 7.3 Hz, 3H);

^{13}C NMR (126 MHz, $CDCl_3$) δ 146.5, 128.9 (q, *J* = 32.4 Hz), 127.9, 125.4 (q, *J* = 3.9 Hz), 124.2 (q, *J* = 272.4 Hz), 109.0, 84.1, 41.1, 38.7, 20.5, 18.8, 13.8, 11.4;

^{19}F NMR (471 MHz, $CDCl_3$) δ –62.3;

HRMS (ESI) calcd. for $C_{22}H_{33}F_3SiNa$ $[M+Na]^+$ *m/z* 405.2196, found 405.2198;

IR (neat, cm^{-1}) 2958, 2865, 2169, 1463, 882, 675.

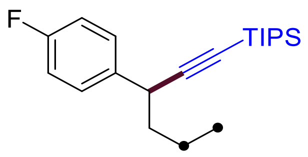

(3-(4-Fluorophenyl)hex-1-yn-1-yl)triisopropylsilane (Figure 3, **3e**). From **1-(but-3-en-1-yl)-4-fluorobenzene** (**1e**) (30.0 mg, 0.20 mmol), and **(bromoethynyl)triisopropylsilane** (**2a**) (78.4 mg, 0.30 mmol), the title compound was prepared following the general procedure **A** using $NiI_2 \cdot xH_2O$ (3.8 mg, 5.0 mol%), **L** (4.3 mg, 6.0 mol%), Na_2CO_3 (42.4 mg, 2.0 equiv), NaI (3.0 mg, 10 mol%), PMHS (60.0 μL, 5.0 equiv) and anhydrous DME (1.0 mL). The reaction mixture was stirred for 16

h at 25 °C. The crude material was purified by flash column chromatography (petroleum ether) to provide the title compound as a colorless liquid in 65% yield (43.2 mg), and >99:1 rr was detected by GC.

^{1}H NMR (500 MHz, $CDCl_3$) δ 7.37 – 7.30 (m, 2H), 7.04 – 6.96 (m, 2H), 3.69 (dd, *J* = 8.1, 6.2 Hz, 1H), 1.74 – 1.65 (m, 2H), 1.55 – 1.38 (m, 2H), 1.12 – 1.04 (m, 21H), 0.93 (t, *J* = 7.4 Hz, 3H);

^{13}C NMR (126 MHz, $CDCl_3$) δ 161.7 (d, *J* = 244.6 Hz), 138.1 (d, *J* = 3.3 Hz), 129.0 (d, *J* = 8.8 Hz), 115.1 (d, *J* = 21.3 Hz), 110.0, 83.4, 41.3, 38.0, 20.5, 18.8, 13.8, 11.4;

^{19}F NMR (471 MHz, $CDCl_3$) δ –116.8;

HRMS (ESI) calcd. for $C_{21}H_{33}FSiNa$ $[M+Na]^+$ *m/z* 355.2228, found 355.2225;

IR (neat, cm^{-1}) 2957, 2864, 2168, 1604, 1462, 665.

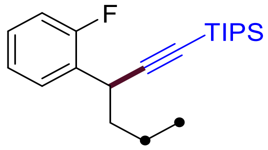

(3-(2-Fluorophenyl)hex-1-yn-1-yl)triisopropylsilane (Figure 3, **3f**). From **1-(but-3-en-1-yl)-2-fluorobenzene** (**1f**) (30.0 mg, 0.20 mmol), and **(bromoethynyl)triisopropylsilane** (**2a**) (78.4 mg, 0.30 mmol), the title compound was prepared following the general procedure **A** using $NiI_2 \cdot xH_2O$ (3.8 mg, 5.0 mol%), **L** (4.3 mg, 6.0 mol%), Na_2CO_3 (42.4 mg, 2.0 equiv), NaI (3.0 mg, 10 mol%), PMHS (60.0 μL, 5.0 equiv) and anhydrous DME (1.0 mL). The reaction mixture was stirred for 16 h at 25 °C. The crude material was purified by flash column chromatography (petroleum ether) to provide the title compound as a colorless liquid in 75% yield (49.8 mg), and >99:1 rr was detected by GC.

^{1}H NMR (500 MHz, $CDCl_3$) δ 7.64 – 7.56 (m, 1H), 7.24 – 7.17 (m, 1H), 7.15 – 7.08 (m, 1H), 7.05 – 6.93 (m, 1H), 4.07 (dd, *J* = 8.2, 5.9 Hz, 1H), 1.76 – 1.64 (m, 2H), 1.58 – 1.44 (m, 2H), 1.13 – 1.07 (m, 21H), 0.94 (t, *J* = 7.3 Hz, 3H);

^{13}C NMR (126 MHz, $CDCl_3$) δ 160.0 (d, *J* = 246.1 Hz), 129.4 (d, *J* = 4.1 Hz), 129.3, 128.2 (d, *J* = 8.1 Hz), 124.1 (d, *J* = 3.5 Hz), 115.2 (d, *J* = 22.3 Hz), 109.1, 83.1, 39.7,

31.9 (d, J = 3.3 Hz), 20.5, 18.8, 13.8, 11.4;

^{19}F NMR (471 MHz, $CDCl_3$) δ –119.8;

HRMS (ESI) calcd. for $C_{21}H_{33}FSiNa$ $[M+Na]^+$ *m/z* 355.2228, found 355.2224;

IR (neat, cm^{-1}) 2957, 2864, 2169, 1228, 882, 675.

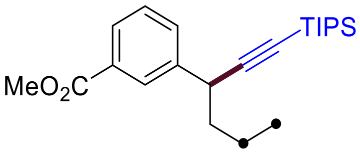

Methyl 3-(1-(triisopropylsilyl)hex-1-yn-3-yl)benzoate (Figure 3, **3g**). From **methyl 3-(but-3-en-1-yl)benzoate** (**1g**) (38.0 mg, 0.20 mmol), and **(bromoethynyl)triisopropylsilane** (**2a**) (78.4 mg, 0.30 mmol), the title compound was prepared following the general procedure **A** using $NiI_2·xH_2O$ (3.8 mg, 5.0 mol%), **L** (4.3 mg, 6.0 mol%), Na_2CO_3 (42.4 mg, 2.0 equiv), NaI (3.0 mg, 10 mol%), PMHS (60.0 μL, 5.0 equiv) and anhydrous diglyme (1.0 mL). The reaction mixture was stirred for 16 h at 25 °C. The crude material was purified by flash column chromatography (petroleum ether/EtOAc = 50:1) to provide the title compound as a colorless liquid in 82% yield (61.0 mg), and >99:1 rr was detected by GC.

^{1}H NMR (500 MHz, $CDCl_3$) δ 8.11 – 8.06 (m, 1H), 7.94 – 7.87 (m, 1H), 7.61 – 7.54 (m, 1H), 7.39 (t, J = 7.7 Hz, 1H), 3.91 (s, 3H), 3.76 (t, J = 7.2 Hz, 1H), 1.80 – 1.67 (m, 2H), 1.55 – 1.36 (m, 2H), 1.13 – 1.05 (m, 21H), 0.93 (t, J = 7.4 Hz, 3H);

^{13}C NMR (126 MHz, $CDCl_3$) δ 167.2, 142.8, 132.1, 130.3, 128.8, 128.5, 127.9, 109.4, 83.9, 52.1, 41.1, 38.5, 20.5, 18.7, 13.8, 11.4;

HRMS (ESI) calcd. for $C_{23}H_{36}O_2SiNa$ $[M+Na]^+$ *m/z* 395.2377, found 395.2374;

IR (neat, cm^{-1}) 2941, 2864, 2168, 1462, 855, 675.

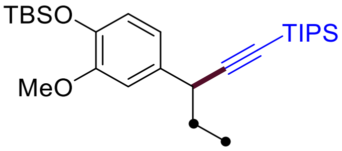

***tert*-Butyl(2-methoxy-4-(1-(triisopropylsilyl)pent-1-yn-3-yl)phenoxy)dimethylsilane** (Figure 3, **3h**). From **(4-allyl-2-methoxyphenoxy)(tert-**

butyl)dimethylsilane (**1h**) (55.7 mg, 0.20 mmol), and **(bromoethynyl)triisopropylsilane** (**2a**) (78.4 mg, 0.30 mmol), the title compound was prepared following the general procedure **A** using $NiI_2·xH_2O$ (3.8 mg, 5.0 mol%), **L** (4.3 mg, 6.0 mol%), Na_2CO_3 (42.4 mg, 2.0 equiv), NaI (3.0 mg, 10 mol%), PMHS (60.0 μL, 5.0 equiv) and anhydrous DME (1.0 mL). The reaction mixture was stirred for 16 h at 25 °C. The crude material was purified by flash column chromatography (petroleum ether/EtOAc = 100:1) to provide the title compound as a colorless liquid in 57% yield (52.4 mg), and >99:1 rr was detected by GC.

^{1}H NMR (500 MHz, $CDCl_3$) δ 6.98 (d, *J* = 1.8 Hz, 1H), 6.81 – 6.73 (m, 2H), 3.80 (s, 3H), 3.61 (dd, *J* = 8.2, 5.4 Hz, 1H), 1.86 – 1.65 (m, 2H), 1.13 – 1.07 (m, 21H), 1.03 – 0.96 (m, 12H), 0.16 (s, 3H), 0.16 (s, 3H);

^{13}C NMR (126 MHz, $CDCl_3$) δ 150.7, 143.5, 135.5, 120.5, 119.7, 111.5, 110.5, 83.2, 55.4, 40.1, 32.2, 25.8, 18.8, 18.5, 11.7, 11.4, –4.4, –4.5;

HRMS (ESI) calcd. for $C_{27}H_{48}O_2Si_2Na$ $[M+Na]^+$ *m/z* 483.3085, found 483.3082;

IR (neat, cm^{-1}) 2938, 2863, 2166, 1463, 1282, 728.

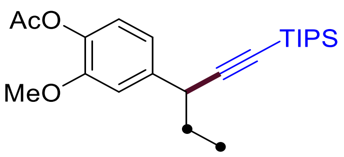

2-Methoxy-4-(1-(triisopropylsilyl)pent-1-yn-3-yl)phenyl acetate (Figure 3, **3i**). From **4-allyl-2-methoxyphenyl acetate** (**1i**) (41.2 mg, 0.20 mmol), and **(bromoethynyl)triisopropylsilane** (**2a**) (78.4 mg, 0.30 mmol), the title compound was prepared following the general procedure **A** using $NiI_2·xH_2O$ (3.8 mg, 5.0 mol%), **L** (4.3 mg, 6.0 mol%), Na_2CO_3 (42.4 mg, 2.0 equiv), NaI (3.0 mg, 10 mol%), PMHS (60.0 μL, 5.0 equiv) and anhydrous diglyme (1.0 mL). The reaction mixture was stirred for 16 h at 25 °C. The crude material was purified by flash column chromatography (petroleum ether/EtOAc = 100:1) to provide the title compound as a colorless liquid in 78% yield (60.8 mg), and >99:1 rr was detected by GC.

^{1}H NMR (500 MHz, $CDCl_3$) δ 7.11 (d, *J* = 2.0 Hz, 1H), 6.97 (d, *J* = 8.1 Hz, 1H), 6.90 (dd, *J* = 8.1, 2.0 Hz, 1H), 3.83 (s, 3H), 3.66 (dd, *J* = 8.5, 5.3 Hz, 1H), 2.31 (s, 3H), 1.88

– 1.78 (m, 1H), 1.77 – 1.68 (m, 1H), 1.14 – 1.07 (m, 21H), 1.05 (t, J = 7.3 Hz, 3H);

^{13}C NMR (126 MHz, $CDCl_3$) δ 169.2, 150.8, 140.9, 138.2, 122.4, 119.7, 111.8, 109.5, 83.8, 55.8, 40.4, 32.2, 20.8, 18.8, 11.8, 11.4;

HRMS (ESI) calcd. for $C_{23}H_{36}O_3SiNa$ $[M+Na]^+$ m/z 411.2326, found 411.2322;

IR (neat, cm^{-1}) 2958, 2864, 2167, 1462, 881, 673.

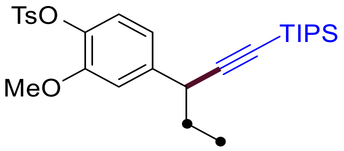

2-Methoxy-4-(1-(triisopropylsilyl)pent-1-yn-3-yl)phenyl 4-methylbenzenesulfonate (Figure 3, **3j**). From **4-allyl-2-methoxyphenyl 4-methylbenzenesulfonate** (**1j**) (63.7 mg, 0.20 mmol), and **(bromoethynyl)triisopropylsilane** (**2a**) (78.4 mg, 0.30 mmol), the title compound was prepared following the general procedure **A** using $NiI_2 \cdot xH_2O$ (3.8 mg, 5.0 mol%), **L** (4.3 mg, 6.0 mol%), Na_2CO_3 (42.4 mg, 2.0 equiv), NaI (3.0 mg, 10 mol%), PMHS (60.0 μL, 5.0 equiv) and anhydrous diglyme (1.0 mL). The reaction mixture was stirred for 16 h at 25 °C. The crude material was purified by flash column chromatography (petroleum ether/EtOAc = 100:1) to provide the title compound as a colorless liquid in 99% yield (98.7 mg), and >99:1 rr was detected by GC.

^{1}H NMR (500 MHz, $CDCl_3$) δ 7.77 – 7.70 (m, 2H), 7.28 (d, J = 8.1 Hz, 2H), 7.07 (d, J = 8.3 Hz, 1H), 6.96 (d, J = 2.0 Hz, 1H), 6.83 (dd, J = 8.3, 2.0 Hz, 1H), 3.61 (dd, J = 8.3, 5.3 Hz, 1H), 3.52 (s, 3H), 2.43 (s, 3H), 1.83 – 1.73 (m, 1H), 1.73 – 1.63 (m, 1H), 1.08 (t, J = 2.2 Hz, 21H), 1.00 (t, J = 7.3 Hz, 3H);

^{13}C NMR (126 MHz, $CDCl_3$) δ 151.5, 144.9, 142.3, 136.9, 133.3, 129.3, 128.7, 123.7, 119.6, 111.9, 109.3, 84.0, 55.4, 40.3, 32.0, 21.7, 18.7, 11.6, 11.3;

HRMS (ESI) calcd. for $C_{28}H_{40}O_4SSiNa$ $[M+Na]^+$ m/z 523.2309, found 523.2304;

IR (neat, cm^{-1}) 2942, 2865, 2168, 1373, 1092, 649.

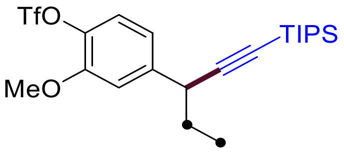

2-Methoxy-4-(1-(triisopropylsilyl)pent-1-yn-3-yl)phenyl trifluoromethanesulfonate (Figure 3, **3k**). From **4-allyl-2-methoxyphenyl trifluoromethanesulfonate** (**1k**) (59.2 mg, 0.20 mmol), and **(bromoethynyl)triisopropylsilane** (**2a**) (78.4 mg, 0.30 mmol), the title compound was prepared following the general procedure **A** using $NiI_2\cdot xH_2O$ (3.8 mg, 5.0 mol%), **L** (4.3 mg, 6.0 mol%), Na_2CO_3 (42.4 mg, 2.0 equiv), NaI (3.0 mg, 10 mol%), PMHS (60.0 μL, 5.0 equiv) and anhydrous diglyme (1.0 mL). The reaction mixture was stirred for 16 h at 25 °C. The crude material was purified by flash column chromatography (petroleum ether/EtOAc = 100:1) to provide the title compound as a colorless liquid in 96% yield (91.4 mg), and >99:1 rr was detected by GC.

^{1}H NMR (500 MHz, $CDCl_3$) δ 7.18 (d, *J* = 2.0 Hz, 1H), 7.15 (d, *J* = 8.2 Hz, 1H), 6.93 (dd, *J* = 8.4, 2.0 Hz, 1H), 3.91 (s, 3H), 3.69 (dd, *J* = 8.4, 5.3 Hz, 1H), 1.88 – 1.78 (m, 1H), 1.76 – 1.67 (m, 1H), 1.12 – 1.10 (m, 21H), 1.05 (t, *J* = 7.3 Hz, 3H);

^{13}C NMR (126 MHz, $CDCl_3$) δ 151.2, 143.8, 137.3, 122.1, 119.9, 118.9 (q, *J* = 321.3 Hz), 112.5, 108.8, 84.6, 56.1, 40.4, 32.1, 18.8, 11.7, 11.4;

^{19}F NMR (471 MHz, $CDCl_3$) δ –73.9;

HRMS (ESI) calcd. for $C_{22}H_{33}F_3O_4SSiNa$ $[M+Na]^+$ *m/z* 501.1713, found 501.1709;

IR (neat, cm^{-1}) 2941, 2865, 2169, 1503, 1205, 881.

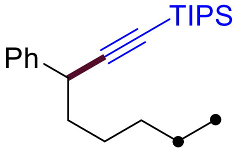

Triisopropyl(3-phenyloct-1-yn-1-yl)silane (Figure 3, **3l**). From **hex-5-en-1-ylbenzene (1l)** (32.1 mg, 0.20 mmol) and **(bromoethynyl)triisopropylsilane (2a)** (78.4 mg, 0.30 mmol), the title compound was prepared following the general procedure **A** using $NiI_2\cdot xH_2O$ (7.6 mg, 10.0 mol%), **L** (8.6 mg, 12.0 mol%), Na_2CO_3 (42.4 mg, 2.0 equiv), NaI (6.0 mg, 20 mol%), PMHS (60.0 μL, 5.0 equiv) and

anhydrous DME (2.0 mL). The reaction mixture was stirred for 16 h at 25 °C. The crude material was purified by flash column chromatography (petroleum ether) to provide the title compound as a colorless liquid in 44% yield (30.3 mg), and >99:1 rr was detected by GC.

^{1}H NMR (400 MHz, $CDCl_3$) δ 7.42 – 7.35 (m, 2H), 7.36 – 7.27 (m, 2H), 7.27 – 7.17 (m, 1H), 3.69 (dd, J = 7.8, 6.4 Hz, 1H), 1.78 – 1.64 (m, 2H), 1.53 – 1.42 (m, 2H), 1.33 – 1.26 (m, 4H), 1.13 – 1.00 (m, 21H), 0.91 – 0.85 (m, 3H);

^{13}C NMR (101 MHz, $CDCl_3$) δ 142.5, 128.4, 127.5, 126.5, 110.2, 83.2, 39.2, 39.0, 31.6, 27.0, 22.6, 18.8, 14.1, 11.4;

HRMS (ESI) calcd. for $C_{23}H_{38}SiNa$ $[M+Na]^+$ *m/z* 365.2635, found 365.2636;

IR (neat, cm^{-1}) 2939, 2863, 2167, 1462, 882, 663.

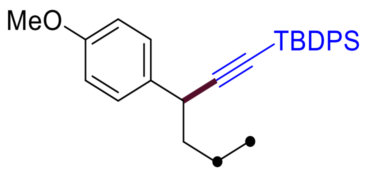

***tert*-Butyldiphenyl(3-phenylhex-1-yn-1-yl)silane** (Figure 3, **3m**). From **1-(but-3-en-1-yl)-4-methoxybenzene** (**1m**) (32.4 mg, 0.20 mmol), and **(bromoethynyl)(*tert*-butyl)diphenylsilane** (**2b**) (103.0 mg, 0.30 mmol), the title compound was prepared following the general procedure **A** using $NiI_2{\cdot}xH_2O$ (3.8 mg, 5.0 mol%), **L** (4.3 mg, 6.0 mol%), Na_2CO_3 (42.4 mg, 2.0 equiv), NaI (3.0 mg, 10 mol%), PMHS (60.0 µL, 5.0 equiv) and anhydrous diglyme (1.0 mL). The reaction mixture was stirred for 16 h at 25 °C. The crude material was purified by flash column chromatography (petroleum ether) to provide the title compound as a colorless liquid in 95% yield (80.9 mg), and >99:1 rr was detected by GC.

^{1}H NMR (500 MHz, $CDCl_3$) 8.14 – 7.62 (m, 4H), 7.43 – 7.32 (m, 8H), 6.94 – 6.85 (m, 2H), 3.87 – 3.70 (m, 4H), 1.90 – 1.71 (m, 2H), 1.63 – 1.46 (m, 2H), 1.11 (s, 9H), 0.97 (t, J = 7.4 Hz, 3H);

^{13}C NMR (126 MHz, $CDCl_3$) δ 158.4, 135.7, 134.1, 134.0, 133.9, 129.4, 128.6, 127.7, 113.9, 113.3, 82.0, 55.4, 41.2, 38.1, 27.2, 20.7, 18.7, 13.9;

HRMS (ESI) calcd. for $C_{29}H_{35}OSi$ $[M+H]^+$ *m/z* 427.2452, found 427.2446;

IR (neat, cm^{-1}) 2956, 2856, 2168, 1509, 1248, 697.

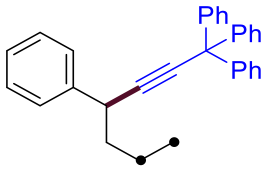

Hept-2-yne-1,1,1,4-tetrayltetrabenzene (Figure 3, **3n**). From **4-phenyl-1- butene** (**1n**) (26.4 mg, 0.20 mmol) and **(3-bromoprop-2-yne-1,1,1-triyl)tribenzene** (**2c**) (104.2 mg, 0.30 mmol), the title compound was prepared following the general procedure **A** using $NiI_2·xH_2O$ (3.8 mg, 5.0 mol%), **L** (4.3 mg, 6.0 mol%), Na_2CO_3 (42.4 mg, 2.0 equiv), NaI (3.0 mg, 10.0 mol%), PMHS (60 μL, 1.0 mmol, 5.0 equiv) and anhydrous diglyme (1.0 mL). The reaction mixture was stirred for 16 h at 25 °C. The crude material was purified by flash column chromatography (petroleum ether) to provide the title compound as a colorless liquid in 40% yield (31.8 mg), and >99:1 rr was detected by GC.

^{1}H NMR (500 MHz, $CDCl_3$) δ 7.40 – 7.37 (m, 2H), 7.33 – 7.20 (m, 18H), 3.83 (dd, *J* = 8.4, 6.0 Hz, 1H), 1.90 – 1.74 (m, 2H), 1.54 – 1.43 (m, 2H), 0.93 (t, *J* = 7.4 Hz, 3H);

^{13}C NMR (126 MHz, $CDCl_3$) δ 145.9, 142.8, 129.3, 128.4, 127.9, 127.6, 126.7, 126.5, 89.0, 87.0, 55.8, 41.2, 38.0, 20.7, 13.9;

HRMS (ESI) calcd. for $C_{31}H_{28}Na$ $[M+Na]^+$ m/z 423.2083, found 423.2090;

IR (neat, cm^{-1}) 2957, 2929, 1490, 1446, 756, 696;

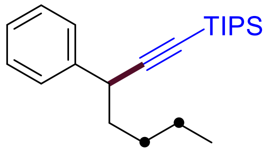

Triisopropyl(3-phenylhept-1-yn-1-yl)silane (Figure 3, **3o**). From **pent-3-en-1-ylbenzene** (**1o**) (29.2 mg, 0.20 mmol) and **(bromoethynyl)triisopropylsilane (2a)** (78.4 mg, 0.30 mmol), the title compound was prepared following the general procedure **A** using $NiI_2·xH_2O$ (3.8 mg, 5.0 mol%), **L** (4.3 mg, 6.0 mol%), Na_2CO_3 (42.4

mg, 2.0 equiv), NaI (3.0 mg, 10.0 mol%), PMHS (60 μL, 1.0 mmol, 5.0 equiv) and anhydrous diglyme (1.0 mL). The reaction mixture was stirred for 16 h at 25 °C. The crude material was purified by flash column chromatography (petroleum ether) to provide the title compound as a colorless liquid in 71% yield (46.6 mg), and >99:1 rr was detected by GC.

^{1}H NMR (500 MHz, $CDCl_3$) δ 7.41 – 7.37 (m, 2H), 7.34 – 7.29 (m, 2H), 7.25 – 7.19 (m, 1H), 3.70 (dd, *J* = 8.1, 6.2 Hz, 1H), 1.82 – 1.69 (m, 2H), 1.52 – 1.42 (m, 2H), 1.40 – 1.25 (m, 2H), 1.20 – 1.05 (m, 21 H), 0.89 (t, *J* = 7.3 Hz, 3H);

^{13}C NMR (126 MHz, $CDCl_3$) δ 142.5, 128.4, 127.5, 126.5, 110.2, 83.2, 39.0, 38.9, 29.6, 22.4, 18.8, 14.1, 11.4;

HRMS (ESI) calcd. for $C_{22}H_{36}SiNa$ $[M+Na]^+$ m/z 351.2478, found 351.2477;

IR (neat, cm^{-1}) 2940, 2864, 2168, 1462, 738, 664.

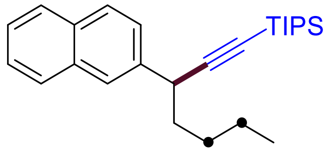

Triisopropyl(3-(naphthalen-2-yl)hept-1-yn-1-yl)silane (Figure 3, **3p**). From **2-(pent-3-en-1-yl)naphthalene** (**1p**) (39.3 mg, 0.20 mmol) and **(bromoethynyl)triisopropylsilane (2a)** (78.4 mg, 0.30 mmol), the title compound was prepared following the general procedure **A** using $NiI_2·xH_2O$ (3.8 mg, 5.0 mol%), **L** (4.3 mg, 6.0 mol%), Na_2CO_3 (42.4 mg, 2.0 equiv), NaI (3.0 mg, 10.0 mol%), PMHS (60 μL, 1.0 mmol, 5.0 equiv) and anhydrous DME (1.0 mL). The reaction mixture was stirred for 16 h at 25 °C. The crude material was purified by flash column chromatography (petroleum ether) to provide the title compound as a colorless liquid in 84% yield (63.8 mg), and >99:1 rr was detected by GC.

^{1}H NMR (500 MHz, $CDCl_3$) δ 7.89 – 7.85 (m, 1H), 7.84 – 7.77 (m, 3H), 7.53 – 7.38 (m, 3H), 3.86 (dd, *J* = 7.9, 6.2 Hz, 1H), 1.88 – 1.77 (m, 2H), 1.54 – 1.46 (m, 2H), 1.42 – 1.29 (m, 2H), 1.17 – 1.08 (m, 21H), 0.90 (t, *J* = 7.3 Hz, 3H);

^{13}C NMR (126 MHz, $CDCl_3$) δ 139.8, 133.5, 132.4, 128.0, 127.8, 127.7, 126.1, 126.0,

125.5, 110.2, 83.5, 39.1, 38.6, 29.6, 22.5, 18.8, 14.1, 11.5;

HRMS (ESI) calcd. for $C_{26}H_{38}SiNa$ $[M+Na]^+$ m/z 401.2635, found 401.2637;

IR (neat, cm^{-1}) 2939, 2863, 2167, 1462, 882, 674.

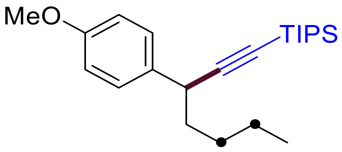

Triisopropyl(3-(4-methoxyphenyl)hept-1-yn-1-yl)silane (Figure 3, **3q**). From **1-methoxy-4-(pent-3-en-1-yl)benzene** (**1q**) (35.3 mg, 0.20 mmol) and **(bromoethynyl)triisopropylsilane (2a)** (78.4 mg, 0.30 mmol), the title compound was prepared following the general procedure **A** using $NiI_2{\cdot}xH_2O$ (3.8 mg, 5.0 mol%), **L** (4.3 mg, 6.0 mol%), Na_2CO_3 (42.4 mg, 2.0 equiv), NaI (3.0 mg, 10.0 mol%), PMHS (60 μL, 1.0 mmol, 5.0 equiv) and anhydrous DME (2.0 mL). The reaction mixture was stirred for 16 h at 25 °C. The crude material was purified by flash column chromatography (petroleum ether) to provide the title compound as a colorless liquid in 70% yield (50.0 mg), and >99:1 rr was detected by GC.

^{1}H NMR (500 MHz, $CDCl_3$) δ 7.31 – 7.27 (m, 2H), 6.88 – 6.79 (m, 2H), 3.80 (s, 3H), 3.64 (dd, J = 7.8, 6.5 Hz, 1H), 1.75 – 1.65 (m, 2H), 1.48 – 1.40 (m, 2H), 1.36 – 1.27 (m, 2H), 1.12 – 1.01 (m, 21H), 0.88 (t, J = 7.3 Hz, 3H);

^{13}C NMR (126 MHz, $CDCl_3$) δ 158.2, 134.6, 128.5, 113.7, 110.6, 82.9, 55.4, 39.0, 38.1, 29.5, 22.5, 18.9, 18.8, 14.1, 11.4;

HRMS (ESI) calcd. for $C_{23}H_{38}OSiNa$ $[M+Na]^+$ m/z 381.2584, found 381.2581;

IR (neat, cm^{-1}) 2940, 2864, 2167, 1510, 1247, 677.

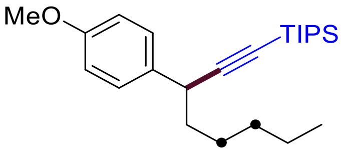

Triisopropyl(3-(4-methoxyphenyl)oct-1-yn-1-yl)silane (Figure 3, **3r**). From **1-(hex-3-en-1-yl)-4-methoxybenzene** (**1r**) (38.1 mg, 0.20 mmol) and **(bromoethynyl)triisopropylsilane (2a)** (78.4 mg, 0.30 mmol), the title compound was

prepared following the general procedure **A** using $NiI_2 \cdot xH_2O$ (3.8 mg, 5.0 mol%), **L** (4.3 mg, 6.0 mol%), Na_2CO_3 (42.4 mg, 2.0 equiv), NaI (3.0 mg, 10.0 mol%), PMHS (60 μL, 1.0 mmol, 5.0 equiv) and anhydrous DME (2.0 mL). The reaction mixture was stirred for 16 h at 25 °C. The crude material was purified by flash column chromatography (petroleum ether/EtOAc = 100:1) to provide the title compound as a colorless liquid in 77% yield (57.6 mg), and >99:1 rr was detected by GC.

^{1}H NMR (500 MHz, $CDCl_3$) δ 7.32 – 7.27 (m, 2H), 6.90 – 6.82 (m, 2H), 3.80 (s, 3H), 3.70 – 3.55 (m, 1H), 1.73 – 1.65 (m, 2H), 1.52 – 1.41 (m, 2H), 1.34 – 1.24 (m, 4H), 1.14 – 1.02 (m, 21H), 0.92 – 0.83 (m, 3H);

^{13}C NMR (126 MHz, $CDCl_3$) δ 158.2, 134.6, 128.5, 113.7, 110.6, 82.9, 55.4, 39.3, 38.1, 31.6, 27.0, 22.7, 18.9, 18.8, 14.1, 11.4;

HRMS (ESI) calcd. for $C_{24}H_{40}OSiNa$ $[M+Na]^+$ m/z 395.2741, found 395.2743;

IR (neat, cm^{-1}) 2937, 2863, 2167, 1510, 1245, 667.

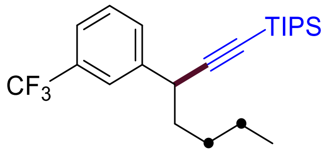

Triisopropyl(3-(3-(trifluoromethyl)phenyl)hept-1-yn-1-yl)silane (Figure 3, **3s**). From **1-(pent-3-en-1-yl)-3-(trifluoromethyl)benzene** (**1s**) (42.8 mg, 0.20 mmol) and **(bromoethynyl)triisopropylsilane (2a)** (78.4 mg, 0.30 mmol), the title compound was prepared following the general procedure **A** using $NiI_2 \cdot xH_2O$ (3.8 mg, 5.0 mol%), **L** (4.3 mg, 6.0 mol%), Na_2CO_3 (42.4 mg, 2.0 equiv), NaI (3.0 mg, 10.0 mol%), PMHS (60 μL, 1.0 mmol, 5.0 equiv) and anhydrous diglyme (1.0 mL). The reaction mixture was stirred for 16 h at 25 °C. The crude material was purified by flash column chromatography (petroleum ether) to provide the title compound as a colorless liquid in 69% yield (54.9 mg), and >99:1 rr was detected by GC.

^{1}H NMR (500 MHz, $CDCl_3$) δ 7.73 – 7.70 (m, 1H), 7.56 – 7.51 (m, 1H), 7.50 – 7.47 (m, 1H), 7.45 – 7.40 (m, 1H), 3.75 (dd, *J* = 8.3, 6.1 Hz, 1H), 1.80 – 1.65 (m, 2H), 1.53 – 1.43 (m, 2H), 1.40 – 1.28 (m, 2H), 1.12 – 1.07 (m, 21H), 0.90 (t, *J* = 7.3 Hz, 3H);

^{13}C NMR (126 MHz, $CDCl_3$) δ 143.5, 130.9, 130.8 (q, J = 32.2 Hz), 128.8, 124.4 (q, J = 3.9 Hz), 124.3 (q, J = 272.7 Hz), 123.5 (q, J = 3.9 Hz), 109.0, 84.4, 38.8, 38.7, 29.5, 22.4, 18.7, 14.1, 11.4;

^{19}F NMR (471 MHz, $CDCl_3$) δ –62.6;

HRMS (ESI) calcd. for $C_{23}H_{35}F_3SiNa$ $[M+Na]^+$ m/z 419.2352, found 419.2351;

IR (neat, cm^{-1}) 2941, 2865, 2170, 1325, 1126, 663.

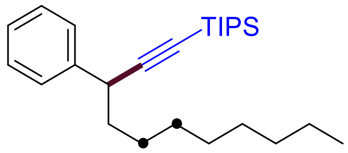

Triisopropyl(3-phenylundec-1-yn-1-yl)silane (Figure 3, **3t**). From **non-3-en-1-ylbenzene** (**1t**) (40.5 mg, 0.20 mmol) and **(bromoethynyl)triisopropylsilane (2a)** (78.4 mg, 0.30 mmol), the title compound was prepared following the general procedure **A** using $NiI_2·xH_2O$ (3.8 mg, 5.0 mol%), **L** (4.3 mg, 6.0 mol%), Na_2CO_3 (42.4 mg, 2.0 equiv), NaI (3.0 mg, 10.0 mol%), PMHS (60 μL, 1.0 mmol, 5.0 equiv) and anhydrous DME (2.0 mL). The reaction mixture was stirred for 16 h at 25 °C. The crude material was purified by flash column chromatography (petroleum ether) to provide the title compound as a colorless liquid in 85% yield (65.5 mg), and >99:1 rr was detected by GC.

^{1}H NMR (500 MHz, $CDCl_3$) δ 7.40 – 7.36 (m, 2H), 7.34 – 7.29 (m, 2H), 7.24 – 7.19 (m, 1H), 3.69 (dd, J = 8.2, 6.2 Hz, 1H), 1.78 – 1.65 (m, 2H), 1.52 – 1.39 (m, 2H), 1.35 – 1.16 (m, 10H), 1.13 – 1.05 (m, 21H), 0.87 (t, J = 6.9 Hz, 3H);

^{13}C NMR (126 MHz, $CDCl_3$) δ 142.5, 128.4, 127.5, 126.5, 110.2, 83.2, 39.2, 39.0, 32.0, 29.6, 29.3, 27.4, 22.8, 18.8, 14.2, 11.4;

HRMS (ESI) calcd. for $C_{26}H_{44}SiNa$ $[M+Na]^+$ m/z 407.3104, found 407.3105;

IR (neat, cm^{-1}) 2924, 2856, 2168, 1454, 966, 696.

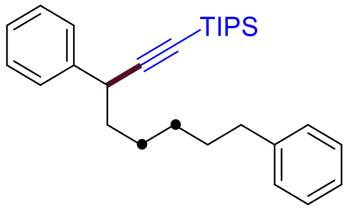

(3,8-Diphenyloct-1-yn-1-yl)triisopropylsilane (Figure 3, **3u**). From **1,6-diphenylhex-3-ene** (**1u**) (47.3 mg, 0.20 mmol) and **(bromoethynyl)triisopropylsilane (2a)** (78.4 mg, 0.30 mmol), the title compound was prepared following the general procedure **A** using $NiI_2 \cdot xH_2O$ (3.8 mg, 5.0 mol%), **L** (4.3 mg, 6.0 mol%), Na_2CO_3 (42.4 mg, 2.0 equiv), NaI (3.0 mg, 10.0 mol%), PMHS (60 μL, 1.0 mmol, 5.0 equiv) and anhydrous DME (1.0 mL). The reaction mixture was stirred for 16 h at 25 °C. The crude material was purified by flash column chromatography (petroleum ether) to provide the title compound as a colorless liquid in 89% yield (74.7 mg), and >99:1 rr was detected by GC.

^{1}H NMR (500 MHz, $CDCl_3$) δ 7.40 – 7.37 (m, 2H), 7.34 – 7.30 (m, 2H), 7.30 – 7.26 (m, 2H), 7.25 – 7.21 (m, 1H), 7.20 – 7.15 (m, 3H), 3.70 (dd, *J* = 8.0, 6.2 Hz, 1H), 2.63 – 2.57 (m, 2H), 1.83 – 1.70 (m, 2H), 1.66 – 1.58 (m, 2H), 1.57 – 1.50 (m, 2H), 1.43 – 1.31 (m, 2H), 1.13 – 1.07 (m, 21H);

^{13}C NMR (126 MHz, $CDCl_3$) δ 142.9, 142.3, 128.5, 128.4, 128.3, 127.5, 126.5, 125.7, 110.1, 83.3, 39.1, 38.9, 35.9, 31.5, 29.0, 27.2, 18.8, 11.4;

HRMS (ESI) calcd. for $C_{29}H_{42}SiNa$ $[M+Na]^+$ m/z 441.2948, found 441.2953;

IR (neat, cm^{-1}) 2937, 2862, 2167, 1453, 882, 697.

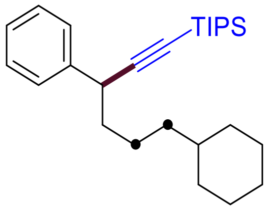

(6-Cyclohexyl-3-phenylhex-1-yn-1-yl)triisopropylsilane (Figure 3, **3v**). From **(4-cyclohexylbut-3-en-1-yl)benzene** (**1v**) (42.9 mg, 0.20 mmol) and **(bromoethynyl)triisopropylsilane (2a)** (78.4 mg, 0.30 mmol), the title compound was

prepared following the general procedure **A** using $NiI_2·xH_2O$ (3.8 mg, 5.0 mol%), **L** (4.3 mg, 6.0 mol%), Na_2CO_3 (42.4 mg, 2.0 equiv), NaI (3.0 mg, 10.0 mol%), PMHS (60 μL, 1.0 mmol, 5.0 equiv) and anhydrous diglyme (1.0 mL). The reaction mixture was stirred for 16 h at 25 °C. The crude material was purified by flash column chromatography (petroleum ether) to provide the title compound as a colorless liquid in 83% yield (65.8 mg), and >99:1 rr was detected by GC.

^{1}H NMR (500 MHz, $CDCl_3$) δ 7.40 – 7.36 (m, 2H), 7.33 – 7.28 (m, 2H), 7.24 – 7.19 (m, 1H), 3.77 – 3.63 (m, 1H), 1.73 – 1.60 (m, 7H), 1.54 – 1.40 (m, 2H), 1.24 – 1.13 (m, 6H), 1.12 – 1.04 (m, 21H), 0.91 – 0.78 (m, 2H);

^{13}C NMR (126 MHz, $CDCl_3$) δ 142.5, 128.4, 127.5, 126.5, 110.2, 83.2, 39.5, 39.0, 37.6, 37.1, 33.5, 33.4, 26.8, 26.6, 26.5, 24.7, 18.9, 18.8, 11.4;

HRMS (ESI) calcd. for $C_{27}H_{44}SiNa$ $[M+Na]^+$ m/z 419.3104, found 419.3101;

IR (neat, cm^{-1}) 2921, 2862, 2168, 1450, 882, 664.

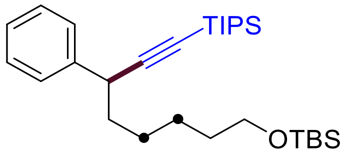

***tert*-Butyldimethyl((6-phenyl-8-(triisopropylsilyl)oct-7-yn-1-yl)oxy)silane** (Figure 3, **3w**). From ***tert*-butyldimethyl((6-phenylhex-3-en-1-yl)oxy)silane** (**1w**) (58.1 mg, 0.20 mmol) and **(bromoethynyl)triisopropylsilane (2a)** (78.4 mg, 0.30 mmol), the title compound was prepared following the general procedure **A** using $NiI_2·xH_2O$ (3.8 mg, 5.0 mol%), **L** (4.3 mg, 6.0 mol%), Na_2CO_3 (42.4 mg, 2.0 equiv), NaI (3.0 mg, 10.0 mol%), PMHS (60 μL, 1.0 mmol, 5.0 equiv) and anhydrous diglyme (1.0 mL). The reaction mixture was stirred for 16 h at 25 °C. The crude material was purified by flash column chromatography (petroleum ether/EtOAc = 100:1) to provide the title compound as a colorless liquid in 45% yield (42.5 mg), and >99:1 rr was detected by GC.

^{1}H NMR (500 MHz, $CDCl_3$) δ 7.41 – 7.36 (m, 2H), 7.34 – 7.29 (m, 2H), 7.24 – 7.20 (m, 1H), 3.69 (dd, *J* = 8.0, 6.3 Hz, 1H), 3.58 (t, *J* = 6.6 Hz, 2H), 1.78 – 1.67 (m,2H),

1.54 – 1.42 (m, 4H), 1.38 – 1.28 (m, 2H), 1.12 – 0.98 (m, 21H), 0.89 (s, 9H), 0.04 (s, 6H);

^{13}C NMR (126 MHz, $CDCl_3$) δ 142.4, 128.4, 127.5, 126.5, 110.1, 83.3, 63.3, 39.2, 38.9, 32.9, 27.2, 26.1, 25.6, 18.8, 18.5, 11.4, –5.1;

HRMS (ESI) calcd. for $C_{29}H_{53}OSi_2$ $[M+H]^+$ m/z 473.3629, found 473.3633;

IR (neat, cm^{-1}) 2937, 2862, 2168, 1098, 833, 663.

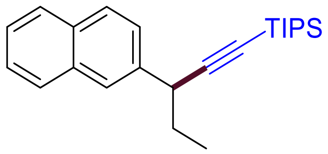

Triisopropyl(3-(naphthalen-2-yl)pent-1-yn-1-yl)silane (Figure 3, **3x**). From **2-(prop-1-en-1-yl)naphthalene** (**1x**) (33.6 mg, 0.20 mmol) and **(bromoethynyl)triisopropylsilane** (**2a**) (78.4 mg, 0.30 mmol), the title compound was prepared following the general procedure **A** using $NiI_2 \cdot xH_2O$ (3.8 mg, 5.0 mol%), **L** (4.3 mg, 6.0 mol%), Na_2CO_3 (42.4 mg, 2.0 equiv), NaI (3.0 mg, 10.0 mol%), PMHS (60 μL, 1.0 mmol, 5.0 equiv) and anhydrous DME (2.0 mL). The reaction mixture was stirred for 16 h at 25 °C. The crude material was purified by flash column chromatography (petroleum ether) to provide the title compound as a colorless liquid in 95% yield (66.5 mg), and >99:1 rr was detected by GC.

^{1}H NMR (400 MHz, $CDCl_3$) δ 7.92 – 7.87 (m, 1H), 7.85 – 7.78 (m, 3H), 7.52 – 7.41 (m, 3H), 3.83 (dd, J = 8.1, 5.5 Hz, 1H), 1.98 – 1.80 (m, 2H), 1.16 – 1.11 (m, 21H), 1.06 (t, J = 7.3 Hz, 3H);

^{13}C NMR (101 MHz, $CDCl_3$) δ 139.5, 133.5, 132.5, 128.0, 127.8, 127.7, 126.3, 126.2, 126.1, 125.6, 109.9, 83.7, 40.6, 31.9, 18.8, 11.8, 11.5;

HRMS (ESI) calcd. for $C_{24}H_{34}SiNa$ $[M+Na]^+$ m/z 373.2322, found 373.2318;

IR (neat, cm^{-1}) 2940, 2863, 2167, 882, 676.

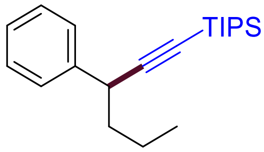

Triisopropyl(3-phenylhex-1-yn-1-yl)silane (Figure 3, **3y**). From **but-1-en-1-ylbenzene** (**1y**) (26.4 mg, 0.20 mmol) and **(bromoethynyl)triisopropylsilane** (**2a**) (78.4 mg, 0.30 mmol), the title compound was prepared following the general procedure **A** using $NiI_2 \cdot xH_2O$ (3.8 mg, 5.0 mol%), **L** (4.3 mg, 6.0 mol%), Na_2CO_3 (42.4 mg, 2.0 equiv), NaI (3.0 mg, 10.0 mol%) and PMHS (60 μL, 1.0 mmol, 5.0 equiv), anhydrous DME (2.0 mL). The reaction mixture was stirred for 16 h at 25 °C. The crude material was purified by flash column chromatography (petroleum ether) to provide the title compound as a colorless liquid in 93% yield (58.7 mg), and >99:1 rr was detected by GC.

^{1}H NMR (400 MHz, $CDCl_3$) δ 7.44 – 7.36 (m, 2H), 7.37 – 7.27 (m, 2H), 7.28 – 7.18 (m, 1H), 3.72 (t, *J* = 7.2 Hz, 1H), 1.79 – 1.68 (m, 2H), 1.58 – 1.45 (m, 2H), 1.14 – 1.06 (m, 21H), 0.94 (t, *J* = 7.3 Hz, 3H);

^{13}C NMR (101 MHz, $CDCl_3$) δ 142.4, 128.4, 127.6, 126.5, 110.2, 83.1, 41.3, 38.8, 20.6, 18.8, 13.9, 11.5;

HRMS (ESI) calcd. for $C_{21}H_{34}SiNa$ $[M+Na]^+$ m/z 337.2322, found 337.2320;

IR (neat, cm^{-1}) 2940, 2864, 2167, 1462, 882, 675.

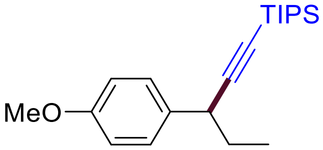

Triisopropyl(3-(4-methoxyphenyl)pent-1-yn-1-yl)silane (Figure 3, **3z**). From **(*E*)-1-methoxy-4-(prop-1-en-1-yl)benzene** (**1z**) (29.6 mg, 0.20 mmol) and **(bromoethynyl)triisopropylsilane** (**2a**) (78.4 mg, 0.30 mmol), the title compound was prepared following the general procedure **A** using $NiI_2 \cdot xH_2O$ (3.8 mg, 5.0 mol%), **L** (4.3 mg, 6.0 mol%), Na_2CO_3 (42.4 mg, 2.0 equiv), NaI (3.0 mg, 10.0 mol%), PMHS (60 μL, 1.0 mmol, 5.0 equiv) and anhydrous DME (1.0 mL). The reaction mixture was stirred for 16 h at 25 °C. The crude material was purified by flash column

chromatography (petroleum ether) to provide the title compound as a colorless liquid in 86% yield (56.8 mg), and >99:1 rr was detected by GC.

^{1}H NMR (400 MHz, $CDCl_3$) δ 7.31 – 7.27 (m, 2H), 6.88 – 6.83 (m, 2H), 3.80 (s, 3H), 3.61 (dd, *J* = 8.1, 5.6 Hz, 1H), 1.82 – 1.66 (m, 2H), 1.14 – 1.05 (m, 21H), 1.01 (t, *J* = 7.3 Hz, 3H);

^{13}C NMR (101 MHz, $CDCl_3$) δ 158.3, 134.2, 128.6, 113.7, 110.3, 83.1, 55.4, 39.7, 32.2, 18.8, 11.7, 11.5;

HRMS (ESI) calcd. for $C_{21}H_{34}OSiNa$ $[M+Na]^+$ m/z 353.2271, found 353.2268;

IR (neat, cm^{-1}) 2940, 2864, 2166, 1510, 1245, 666.

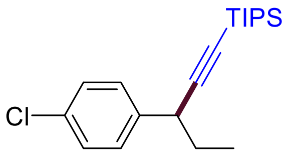

(3-(4-Chlorophenyl)pent-1-yn-1-yl)triisopropylsilane (Figure 3, **3a'**). From **1-chloro-4-(prop-1-en-1-yl)benzene** (**1a'**) (30.5 mg, 0.20 mmol) and **(bromoethynyl)triisopropylsilane** (**2a**) (78.4 mg, 0.30 mmol), the title compound was prepared following the general procedure **A** using $NiI_2·xH_2O$ (3.8 mg, 5.0 mol%), **L** (4.3 mg, 6.0 mol%), Na_2CO_3 (42.4 mg, 2.0 equiv), NaI (3.0 mg, 10.0 mol%), PMHS (60 µL, 1.0 mmol, 5.0 equiv) and anhydrous DME (2.0 mL). The reaction mixture was stirred for 16 h at 25 °C. The crude material was purified by flash column chromatography (petroleum ether) to provide the title compound as a colorless liquid in 94% yield (62.8 mg), and >99:1 rr was detected by GC.

^{1}H NMR (500 MHz, $CDCl_3$) δ 7.37 – 7.27 (m, 4H), 3.63 (dd, *J* = 8.2, 5.6 Hz, 1H), 1.83 – 1.75 (m, 1H), 1.75 – 1.67 (m, 1H), 1.13 – 1.05 (m, 21H), 1.01 (t, *J* = 7.3 Hz, 3H);

^{13}C NMR (126 MHz, $CDCl_3$) δ 140.6, 132.3, 129.0, 128.5, 109.3, 83.8, 39.9, 32.0, 18.8, 11.6, 11.4;

HRMS (ESI) calcd. for $C_{20}H_{31}ClSiNa$ $[M+Na]^+$ m/z 357.1776, found 357.1779;

IR (neat, cm^{-1}) 2941, 2864, 2169, 1407, 881, 661.

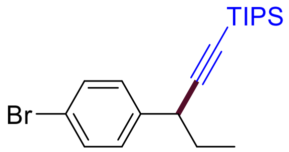

(3-(4-Bromophenyl)pent-1-yn-1-yl)triisopropylsilane (Figure 3, **3b'**). From **1-bromo-4-(prop-1-en-1-yl)benzene** (**1b'**) (39.4 mg, 0.20 mmol) and **(bromoethynyl)triisopropylsilane** (**2a**) (78.4 mg, 0.30 mmol), the title compound was prepared following the general procedure **A** using $NiI_2·xH_2O$ (3.8 mg, 5.0 mol%), **L** (4.3 mg, 6.0 mol%), Na_2CO_3 (42.4 mg, 2.0 equiv), NaI (3.0 mg, 10.0 mol%), PMHS (60 μL, 1.0 mmol, 5.0 equiv) and anhydrous DME (2.0 mL). The reaction mixture was stirred for 16 h at 25 °C. The crude material was purified by flash column chromatography (petroleum ether) to provide the title compound as a colorless liquid in 96% yield (72.7 mg), and >99:1 rr was detected by GC.

^{1}H NMR (500 MHz, $CDCl_3$) δ 7.47 – 7.41 (m, 2H), 7.28 – 7.23 (m, 2H), 3.62 (dd, *J* = 8.1, 5.5 Hz, 1H), 1.83 – 1.75 (m, 1H), 1.75 – 1.65 (m, 1H), 1.10 – 1.08 (m, 21H), 1.01 (t, *J* = 7.4 Hz, 3H);

^{13}C NMR (126 MHz, $CDCl_3$) δ 141.1, 131.4, 129.4, 120.4, 109.2, 83.9, 40.0, 32.0, 18.8, 11.6, 11.4;

HRMS (ESI) calcd. for $C_{20}H_{31}BrSiNa$ $[M+Na]^+$ m/z 401.1271, found 401.1270;

IR (neat, cm^{-1}) 2940, 2864, 2169, 1461, 1101, 881.

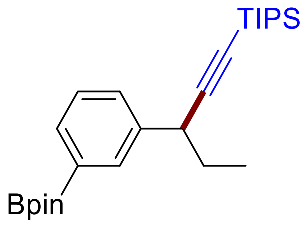

Triisopropyl(3-(3-(4,4,5,5-tetramethyl-1,3,2-dioxaborolan-2-yl)phenyl)pent-1-yn-1-yl)silane (Figure 3, **3c'**). From **4,4,5,5-tetramethyl-2-(3-(prop-1-en-1-yl)phenyl)-1,3,2-dioxaborolane** (**1c'**) (48.8 mg, 0.20 mmol) and **(bromoethynyl)triisopropylsilane** (**2a**) (78.4 mg, 0.30 mmol), the title compound was prepared following the general procedure **A** using $NiI_2·xH_2O$ (3.8 mg, 5.0 mol%), **L**

(4.3 mg, 6.0 mol%), Na_2CO_3 (42.4 mg, 2.0 equiv), NaI (3.0 mg, 10.0 mol%), PMHS (60 μL, 1.0 mmol, 5.0 equiv) and anhydrous DME (1.0 mL). The reaction mixture was stirred for 16 h at 25 °C. The crude material was purified by flash column chromatography (petroleum ether/EtOAc = 50:1) to provide the title compound as a colorless liquid in 69% yield (59.1 mg), and >99:1 rr was detected by GC.

^{1}H NMR (500 MHz, $CDCl_3$) δ 7.85 – 7.81 (m, 1H), 7.66 (d, *J* = 7.3 Hz, 1H), 7.53 – 7.47 (m, 1H), 7.32 (t, *J* = 7.5 Hz, 1H), 3.66 (dd, *J* = 8.5, 5.3 Hz, 1H), 1.87 – 1.77 (m, 1H), 1.76 – 1.68 (m, 1H), 1.34 (s, 12H), 1.13 – 1.07 (m, 21H), 1.02 (t, *J* = 7.3 Hz, 3H);

^{13}C NMR (126 MHz, $CDCl_3$) δ 141.4, 134.1, 133.0, 130.5, 127.8, 110.0, 83.8, 83.4, 40.5, 32.1, 25.0, 24.9, 18.8, 11.9, 11.5;

HRMS (ESI) calcd. for $C_{26}H_{43}BO_2SiNa$ $[M+Na]^+$ m/z 449.3018, found 449.3019;

IR (neat, cm^{-1}) 2940, 2864, 2168, 1357, 1143, 673.

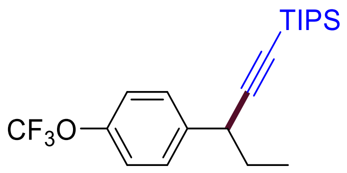

Triisopropyl(3-(4-(trifluoromethoxy)phenyl)pent-1-yn-1-yl)silane (Figure 3, **3d'**). From **1-(prop-1-en-1-yl)-4-(trifluoromethoxy)benzene** (**1d'**) (40.4 mg, 0.20 mmol) and **(bromoethynyl)triisopropylsilane** (**2a**) (78.4 mg, 0.30 mmol), the title compound was prepared following the general procedure **A** using $NiI_2 \cdot xH_2O$ (3.8 mg, 5.0 mol%), **L** (4.3 mg, 6.0 mol%), Na_2CO_3 (42.4 mg, 2.0 equiv), NaI (3.0 mg, 10.0 mol%), PMHS (60 μL, 1.0 mmol, 5.0 equiv) and anhydrous DME (2.0 mL). The reaction mixture was stirred for 16 h at 25 °C. The crude material was purified by flash column chromatography (petroleum ether) to provide the title compound as a colorless liquid in 93% yield (71.8 mg), and >99:1 rr was detected by GC.

^{1}H NMR (500 MHz, $CDCl_3$) δ 7.43 – 7.38 (m, 2H), 7.16 (d, *J* = 8.2 Hz, 2H), 3.68 (dd, *J* = 8.3, 5.4 Hz, 1H), 1.86 – 1.66 (m, 2H), 1.12 – 1.07 (m, 21H), 1.02 (t, *J* = 7.3 Hz, 3H);

^{13}C NMR (126 MHz, $CDCl_3$) δ 147.9, 140.8, 128.9, 120.9, 120.7 (q, *J* = 257.2 Hz),

109.2, 84.1, 39.9, 32.1, 18.8, 11.7, 11.4;

^{19}F NMR (471 MHz, $CDCl_3$) δ –57.9;

HRMS (ESI) calcd. for $C_{21}H_{31}F_3OSiNa$ $[M+Na]^+$ m/z 407.1988, found 407.1987;

IR (neat, cm^{-1}) 2942, 2865, 2170, 1256, 1162, 673.

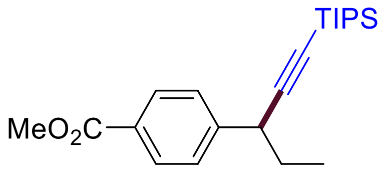

Methyl 4-(1-(triisopropylsilyl)pent-1-yn-3-yl)benzoate (Figure 3, **3e'**). From **methyl 4-(prop-1-en-1-yl)benzoate** (**1e'**) (35.2 mg, 0.20 mmol) and **(bromoethynyl)triisopropylsilane** (**2a**) (78.4 mg, 0.30 mmol), the title compound was prepared following the general procedure **A** using $NiI_2 \cdot xH_2O$ (3.8 mg, 5.0 mol%), **L** (4.3 mg, 6.0 mol%), Na_2CO_3 (42.4 mg, 2.0 equiv), NaI (3.0 mg, 10.0 mol%), PMHS (60 μL, 1.0 mmol, 5.0 equiv) and anhydrous DME (1.0 mL). The reaction mixture was stirred for 16 h at 25 °C. The crude material was purified by flash column chromatography (petroleum ether/EtOAc = 50:1) to provide the title compound as a colorless liquid in 76% yield (54.4 mg), and >99:1 rr was detected by GC.

^{1}H NMR (400 MHz, $CDCl_3$) δ 8.02 – 7.95 (m, 2H), 7.48 – 7.41 (m, 2H), 3.91 (s, 3H), 3.70 (dd, *J* = 8.1, 5.5 Hz, 1H), 1.86 – 1.69 (m, 2H), 1.15 – 1.04 (m, 21H), 1.01 (t, *J* = 7.4 Hz, 3H);

^{13}C NMR (101 MHz, $CDCl_3$) δ 167.1, 147.4, 129.8, 128.6, 127.7, 109.0, 84.2, 52.1, 40.5, 31.9, 18.8, 11.7, 11.4;

HRMS (ESI) calcd. for $C_{22}H_{34}O_2SiNa$ $[M+Na]^+$ m/z 381.2220, found 381.2221;

IR (neat, cm^{-1}) 2941, 2864, 2169, 1725, 1276, 676.

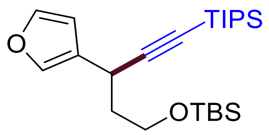

***tert*-Butyl((3-(furan-3-yl)-5-(triisopropylsilyl)pent-4-yn-1-yl)oxy)dimethylsilane** (Figure 3, **3f'**). From ***tert*-butyl((3-(furan-3-yl)allyl)oxy)dimethylsilane** (**1f'**) (47.7 mg, 0.20 mmol) and **(bromoethynyl)triisopropylsilane** (**2a**) (78.4 mg, 0.30 mmol), the title compound was prepared following the general procedure **A** using $NiI_2\cdot xH_2O$ (3.8 mg, 5.0 mol%), **L** (4.3 mg, 6.0 mol%), Na_2CO_3 (42.4 mg, 2.0 equiv), NaI (3.0 mg, 10.0 mol%), PMHS (60 µL, 1.0 mmol, 5.0 equiv) and anhydrous DME (1.0 mL). The reaction mixture was stirred for 16 h at 25 ^{o}C. The crude material was purified by flash column chromatography (petroleum ether/EtOAc = 200:1) to provide the title compound as a colorless liquid in 85% yield (71.9 mg), and >99:1 rr was detected by GC.

^{1}H NMR (500 MHz, $CDCl_3$) δ 7.39 – 7.37 (m, 1H), 7.37 – 7.35 (m, 1H), 6.40 – 6.34 (m, 1H), 3.90 – 3.83 (m, 1H), 3.81 – 3.71 (m, 2H), 2.01 – 1.91 (m, 1H), 1.89 – 1.80 (m, 1H), 1.11 – 1.02 (m, 21H), 0.91 (s, 9H), 0.07 (s, 3H), 0.07 (s, 3H);

^{13}C NMR (126 MHz, $CDCl_3$) δ 143.1, 139.5, 126.2, 110.0, 109.1, 82.0, 60.6, 39.9, 26.1, 25.9, 18.8, 18.4, 11.4, –5.1, –5.2;

HRMS (ESI) calcd. for $C_{24}H_{44}O_2Si_2Na$ $[M+Na]^+$ m/z 443.2772, found 443.2767;

IR (neat, cm^{-1}) 2942, 2864, 2168, 1103, 833, 775.

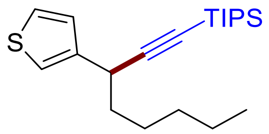

Triisopropyl(3-(thiophen-3-yl)oct-1-yn-1-yl)silane (Figure 3, **3g'**). From **3-(hex-1-en-1-yl)thiophene** (**1g'**) (33.3 mg, 0.20 mmol) and **(bromoethynyl)triisopropylsilane** (**2a**) (78.4 mg, 0.30 mmol), the title compound was prepared following the general procedure **A** using $NiI_2\cdot xH_2O$ (3.8 mg, 5.0 mol%), **L** (4.3 mg, 6.0 mol%), Na_2CO_3 (42.4 mg, 2.0 equiv), NaI (3.0 mg, 10.0 mol%), PMHS (60 µL, 1.0 mmol, 5.0 equiv) and anhydrous DME (1.0 mL). The reaction mixture was stirred for 16 h at 25 ^{o}C. The crude

material was purified by flash column chromatography (petroleum ether) to provide the title compound as a colorless liquid in 91% yield (63.2 mg).

^{1}H NMR (500 MHz, $CDCl_3$) δ 7.29 – 7.23 (m, 1H), 7.21 – 7.16 (m, 1H), 7.05 (dd, *J* = 4.9, 1.3 Hz, 1H), 3.79 (dd, *J* = 8.6, 5.3 Hz, 1H), 1.85 – 1.66 (m, 2H), 1.57 – 1.45 (m, 2H), 1.36 – 1.25 (m, 4H), 1.14 – 1.05 (m, 21H), 0.89 (t, *J* = 7.0 Hz, 3H);

^{13}C NMR (126 MHz, $CDCl_3$) δ 142.9, 127.1, 125.5, 120.7, 110.0, 82.6, 37.7, 34.3, 31.5, 26.8, 22.7, 18.8, 14.1, 11.4;

HRMS (ESI) calcd. for $C_{21}H_{36}SSiNa$ $[M+Na]^+$ m/z 371.2199, found 371.2200;

IR (neat, cm^{-1}) 2936, 2863, 2168, 1461, 882, 664;

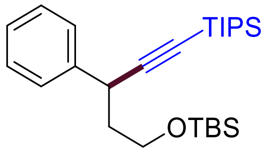

***tert*-Butyldimethyl((3-phenyl-5-(triisopropylsilyl)pent-4-yn-1-yl)oxy)silane** (Figure 3, **3h'**). From ***tert*-butyl(cinnamyloxy)dimethylsilane** (**1h'**) (49.7 mg, 0.20 mmol) and **(bromoethynyl)triisopropylsilane** (**2a**) (78.4 mg, 0.30 mmol), the title compound was prepared following the general procedure **A** using $NiI_2·xH_2O$ (3.8 mg, 5.0 mol%), **L** (4.3 mg, 6.0 mol%), Na_2CO_3 (42.4 mg, 2.0 equiv), NaI (3.0 mg, 10.0 mol%), PMHS (60 μL, 1.0 mmol, 5.0 equiv) and anhydrous DME (1.0 mL). The reaction mixture was stirred for 16 h at 25 °C. The crude material was purified by flash column chromatography (petroleum ether) to provide the title compound as a colorless liquid in 65% yield (55.8 mg), and >99:1 rr was detected by GC.

^{1}H NMR (400 MHz, $CDCl_3$) δ 7.44 – 7.37 (m, 2H), 7.31 (t, *J* = 7.6 Hz, 2H), 7.27 – 7.17 (m, 1H), 3.90 (dd, *J* = 9.4, 5.7 Hz, 1H), 3.87 – 3.81 (m, 1H), 3.74 – 3.67 (m, 1H), 2.04 – 1.83 (m, 2H), 1.17 – 1.05 (m, 21H), 0.91 (s, 9H), 0.07 (s, 3H), 0.06 (s, 3H);

^{13}C NMR (101 MHz, $CDCl_3$) δ 142.1, 128.4, 127.6, 126.6, 109.7, 83.4, 60.8, 42.2, 35.2, 26.1, 18.8, 18.4, 11.4, –5.1, –5.1;

HRMS (ESI) calcd. for $C_{26}H_{46}OSi_2Na$ $[M+Na]^+$ m/z 453.2979, found 453.2977;

IR (neat, cm^{-1}) 2941, 2863, 2167, 1104, 832, 698.

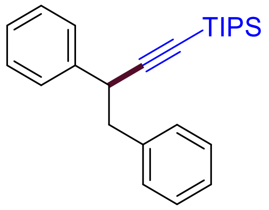

(3,4-Biphenylbut-1-yn-1-yl)triisopropylsilane (Figure 3, **3i'**). From **(*E*)-1,2-diphenylethene** (**1i'**) (36.1 mg, 0.20 mmol) and **(bromoethynyl)triisopropylsilane** (**2a**) (78.4 mg, 0.30 mmol), the title compound was prepared following the general procedure **A** using $NiI_2 \cdot xH_2O$ (3.8 mg, 5.0 mol%), **L** (4.3 mg, 6.0 mol%), Na_2CO_3 (42.4 mg, 2.0 equiv), NaI (3.0 mg, 10.0 mol%), PMHS (60 μL, 1.0 mmol, 5.0 equiv) and anhydrous DME (2.0 mL). The reaction mixture was stirred for 16 h at 25 °C. The crude material was purified by flash column chromatography (petroleum ether/EtOAc = 100:1) to provide the title compound as a colorless liquid in 91% yield (65.9 mg), and >99:1 rr was detected by GC.

^{1}H NMR (400 MHz, $CDCl_3$) δ 7.45 – 7.34 (m, 4H), 7.33 – 7.26 (m, 4H), 7.24 – 7.20 (m, 2H), 4.04 (dd, *J* = 8.3, 6.2 Hz,1H), 3.18 – 3.00 (m, 2H), 1.20 – 0.76 (m, 21H);

^{13}C NMR (101 MHz, $CDCl_3$) δ 141.5, 138.9, 129.6, 128.4, 128.1, 127.8, 126.8, 126.4, 109.2, 84.5, 45.5, 41.3, 18.7, 11.4;

HRMS (ESI) calcd. for $C_{25}H_{34}SiNa$ $[M+Na]^+$ m/z 385.2322, found 385.2321;

IR (neat, cm^{-1}) 2941, 2864, 2169, 1453, 882, 697.

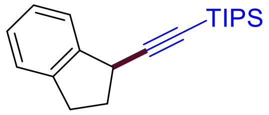

((2,3-Dihydro-1*H*-inden-1-yl)ethynyl)triisopropylsilane (Figure 3, **3j'**). From **1*H*-indene** (**1j'**) (23.2 mg, 0.20 mmol) and **(bromoethynyl)triisopropylsilane** (**2a**) (78.4 mg, 0.30 mmol), the title compound was prepared following the general procedure **A** using $NiI_2 \cdot xH_2O$ (3.8 mg, 5.0 mol%), **L** (4.3 mg, 6.0 mol%), Na_2CO_3 (42.4 mg, 2.0 equiv), NaI (3.0 mg, 10.0 mol%), PMHS (60 μL, 1.0 mmol, 5.0 equiv) and anhydrous DME (1.0 mL). The reaction mixture was stirred for 16 h at 25 °C. The crude material was purified by flash column chromatography (petroleum ether) to provide the title

compound as a colorless liquid in 94% yield (56.3 mg), and >99:1 rr was detected by GC.

^{1}H NMR (500 MHz, $CDCl_3$) δ 7.45 – 7.39 (m, 1H), 7.26 – 7.16 (m, 3H), 4.08 – 3.99 (m, 1H), 3.03 – 2.93 (m, 1H), 2.92 – 2.81 (m, 1H), 2.65 – 2.44 (m, 1H), 2.20 – 2.03 (m, 1H), 1.15 – 1.03 (m, 21H);

^{13}C NMR (126 MHz, $CDCl_3$) δ 143.9, 143.0, 127.0, 126.6, 124.4, 124.3, 110.3, 81.1, 37.4, 34.8, 31.6, 18.8, 11.4;

HRMS (ESI) calcd. for $C_{20}H_{30}SiNa$ [M+Na]$^+$ m/z 321.2009, found 321.2009;

IR (neat, cm^{-1}) 2941, 2864, 2172, 1460, 881, 671.

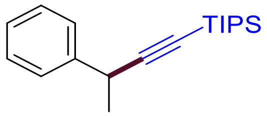

Triisopropyl(3-phenylbut-1-yn-1-yl)silane (Figure 3, **3k'**). From **styrene** (**1k'**) (20.8 mg, 0.20 mmol) and **(bromoethynyl)triisopropylsilane** (**2a**) (78.4 mg, 0.30 mmol), the title compound was prepared following the general procedure **A** using $NiI_2·xH_2O$ (3.8 mg, 5.0 mol%), **L** (4.3 mg, 6.0 mol%), Na_2CO_3 (42.4 mg, 2.0 equiv), NaI (3.0 mg, 10.0 mol%), PMHS (60 μL, 1.0 mmol, 5.0 equiv) and anhydrous DME (1.0 mL). The reaction mixture was stirred for 16 h at 25 °C. The crude material was purified by flash column chromatography (petroleum ether) to provide the title compound as a colorless liquid in 57% yield (32.4 mg), and >99:1 rr was detected by GC.

^{1}H NMR (500 MHz, $CDCl_3$) δ 7.47 (d, *J* = 7.9 Hz, 2H), 7.37 (t, *J* = 7.7 Hz, 2H), 7.30 – 7.25 (m, 1H), 3.87 (q, *J* = 7.1 Hz, 1H), 1.56 (d, *J* = 7.2 Hz, 3H), 1.21 – 1.04 (m, 21H).

^{13}C NMR (126 MHz, $CDCl_3$) δ 143.4, 128.5, 127.0, 126.6, 111.3, 82.4, 33.1, 25.2, 18.8, 11.4.

HRMS (ESI) calcd. for $C_{19}H_{30}SiNa$ [M+Na]$^+$ *m/z* 309.2009, found 309.2011;

IR (neat, cm^{-1}) 2941, 2864, 2164, 1462, 1097, 663.

Enantioselective NiH-Catalyzed Reductive Hydroalkynylation.

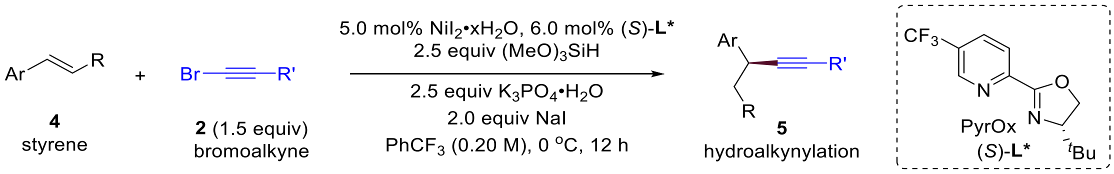

<u>General procedure (B)</u> for enantioselective NiH-catalyzed reductive hydroalkynylation. In a nitrogen-filled glove box, to an oven-dried 8 mL screw-cap vial equipped with a magnetic stir bar were added $NiI_2{\cdot}xH_2O$ (3.8 mg, 5.0 mol%), (*S*)-**L*** (3.3 mg, 6.0 mol%), $K_3PO_4{\cdot}H_2O$ (115.1 mg, 2.5 equiv), NaI (60.0 mg, 2.0 equiv) and anhydrous $PhCF_3$ (1.0 mL). The mixture was stirred for 20 min at room temperature (stirred at 800 rpm) before the addition of $(MeO)_3SiH$ (64 μL, 0.50 mmol, 2.5 equiv). Stirring was continued for an additional 5 min at room temperature before the addition of olefin **4** (0.20 mmol, 1.0 equiv) and bromoalkyne **2** (0.30 mmol, 1.5 equiv). The tube was sealed with a teflon-lined screw cap, removed from the glove box and the reaction was stirred at 0 °C for up to 12 h (the mixture was stirred at 800 rpm). After the reaction was complete, the reaction was quenched upon the addition of H_2O, and the mixture was extracted with EtOAc. The organic layer was concentrated to give the crude product. Dodecane (20 μL) was added as an internal standard for GC analysis. The product was purified by flash column chromatography (petroleum ether/EtOAc) for each substrate. The yields reported are the average of at least two experiments, unless otherwise indicated. The enantiomeric excesses (% ee) of the corresponding products were determined by HPLC analysis using chiral stationary phases.

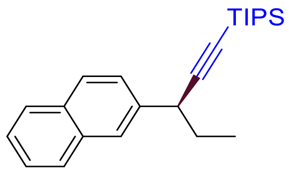

(*S*)-Triisopropyl(3-(naphthalen-2-yl)pent-1-yn-1-yl)silane (Figure 4, **5a**). From **2-(prop-1-en-1-yl)naphthalene** (**4a**) (33.6 mg, 0.20 mmol) and **(bromoethynyl)triisopropylsilane** (**2a**) (78.4 mg, 0.30 mmol), the title compound was prepared following the general procedure **B** using $NiI_2 \cdot xH_2O$ (3.8 mg, 5.0 mol%), (*S*)-**L*** (3.3 mg, 6.0 mol%), $K_3PO_4 \cdot H_2O$ (115.1 mg, 2.5 equiv), NaI (60.0 mg, 2.0 equiv), $(MeO)_3SiH$ (64 μL, 0.5 mmol, 2.5 equiv) and anhydrous $PhCF_3$ (1.0 mL). The reaction mixture was stirred for 12 h at 0 ^{o}C. The crude material was purified by flash column chromatography (petroleum ether) to provide the title compound as a colorless liquid in 71% yield (49.7 mg).

^{1}H NMR (500 MHz, $CDCl_3$) δ 7.94 (s, 1H), 7.89 – 7.80 (m, 3H), 7.61 – 7.44 (m, 3H), 3.88 (dd, *J* = 8.0, 5.6 Hz, 1H), 2.01 – 1.93 (m, 1H), 1.92 – 1.85 (m, 1H), 1.23 – 1.16 (m, 21H), 1.11 (t, *J* = 7.3 Hz, 3H);

^{13}C NMR (126 MHz, $CDCl_3$) δ 139.5, 133.5, 132.5, 128.0, 127.8, 127.7, 126.2, 126.1, 126.0, 125.6, 109.9, 83.7, 40.6, 31.9, 18.8, 11.8, 11.5;

HRMS (ESI) calcd. for $C_{24}H_{35}Si$ $[M+H]^+$ m/z 351.2503, found 351.2504;

IR (neat, cm^{-1}) 2940, 2863, 2167, 1461, 882, 669;

$[\alpha]_D^{18}$ = –23.9 (c = 1.76, $CHCl_3$); 94% *ee*;

HPLC analysis CHIRALCEL OD-H column, *n*-hexane/*iso*-propanol = 100/0, flow rate 0.8 mL/min, 220 nm UV detector, t_R (minor) = 7.5 min, t_R (major) = 8.6 min.

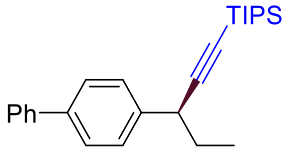

(*S*)-(3-([1,1'-Biphenyl]-4-yl)pent-1-yn-1-yl)triisopropylsilane (Figure 4, **5b**). From **4-(prop-1-en-1-yl)-1,1'-biphenyl** (**4b**) (38.9 mg, 0.20 mmol) and **(bromoethynyl)triisopropylsilane** (**2a**) (78.4 mg, 0.30 mmol), the title compound was

prepared following the general procedure **B** using $NiI_2 \cdot xH_2O$ (3.8 mg, 5.0 mol%), (*S*)-**L*** (3.3 mg, 6.0 mol%), $K_3PO_4 \cdot H_2O$ (115.1 mg, 2.5 equiv), NaI (60.0 mg, 2.0 equiv), $(MeO)_3SiH$ (64 µL, 0.5 mmol, 2.5 equiv) and anhydrous $PhCF_3$ (1.0 mL). The reaction mixture was stirred for 12 h at 0 °C. The crude material was purified by flash column chromatography (petroleum ether) to provide the title compound as a colorless liquid in 66% yield (49.5 mg).

^{1}H NMR (500 MHz, $CDCl_3$) δ 7.67 – 7.62 (m, 2H), 7.62 – 7.58 (m, 2H), 7.53 – 7.44 (m, 4H), 7.41 – 7.30 (m, 1H), 3.76 (dd, *J* = 8.3, 5.4 Hz, 1H), 1.97 – 1.75 (m, 2H), 1.20 – 1.14 (m, 21H), 1.11 (t, *J* = 7.4 Hz, 3H);

^{13}C NMR (126 MHz, $CDCl_3$) δ 141.2, 141.0, 139.5, 128.8, 128.0, 127.3, 127.2, 127.1, 109.8, 83.5, 40.2, 32.2, 18.8, 11.8, 11.5;

HRMS (APCI) calcd. for $C_{26}H_{36}SiNa$ $[M+Na]^+$ m/z 399.2478, found 399.2474;

IR (neat, cm^{-1}) 2958, 2863, 2167, 1486, 761, 695;

$[\alpha]_D^{18}$ = –11.9 (c = 1.46, $CHCl_3$); 94% *ee*;

HPLC analysis CHIRALCEL OD-H column, *n*-hexane/*iso*-propanol = 100/0, flow rate 0.8 mL/min, 220 nm UV detector, t_R (minor) = 10.4 min, t_R (major) = 12.4 min.

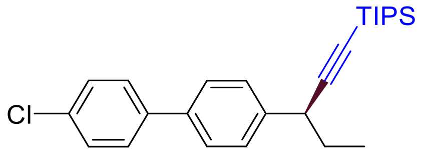

(*S*)-(3-([1,1'-Biphenyl]-4-yl)pent-1-yn-1-yl)triisopropylsilane (Figure 4, **5c**). From **4-chloro-4'-(prop-1-en-1-yl)-1,1'-biphenyl** (**4c**) (45.7 mg, 0.20 mmol) and **(bromoethynyl)triisopropylsilane** (**2a**) (78.4 mg, 0.30 mmol), the title compound was prepared following the general procedure **B** using $NiI_2 \cdot xH_2O$ (3.8 mg, 5.0 mol%), (*S*)-**L*** (3.3 mg, 6.0 mol%), $K_3PO_4 \cdot H_2O$ (115.1 mg, 2.5 equiv), NaI (60.0 mg, 2.0 equiv), $(MeO)_3SiH$ (64 µL, 0.5 mmol, 2.5 equiv) and anhydrous $PhCF_3$ (1.0 mL). The reaction mixture was stirred for 12 h at 0 °C. The crude material was purified by flash column chromatography (petroleum ether) to provide the title compound as a colorless liquid in 51% yield (42.1 mg).

^{1}**H NMR** (500 MHz, $CDCl_3$) δ 7.56 – 7.52 (m, 4H), 7.51 – 7.46 (m, 2H), 7.44 – 7.39 (m, 2H), 3.74 (dd, *J* = 8.3, 5.4 Hz, 1H), 1.92 – 1.76 (m, 2H), 1.19 – 1.12 (m, 21H), 1.09 (t, *J* = 7.3 Hz, 3H);

^{13}**C NMR** (126 MHz, $CDCl_3$) δ 141.6, 139.5, 138.2, 133.3, 128.9, 128.3, 128.2, 126.9, 109.7, 83.6, 40.2, 32.1, 18.8, 11.8, 11.5;

HRMS (ESI) calcd. for $C_{26}H_{35}ClSiNa$ $[M+Na]^+$ m/z 433.2089, found 433.2092

IR (neat, cm^{-1}) 2940, 2864, 2167, 1484, 814, 671;

$[\alpha]_D^{18}$ = –3.3 (c = 1.38, $CHCl_3$); 96% *ee*;

HPLC analysis CHIRALCEL two connected OD-H columns, *n*-hexane/*iso*-propanol = 100/0, flow rate 0.8 mL/min, 220 nm UV detector, t_R (minor) = 13.5 min, t_R (major) = 14.2 min.

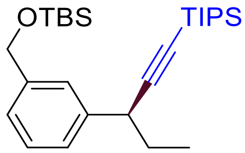

(*S*)-*tert*-Butyldimethyl((3-(1-(triisopropylsilyl)pent-1-yn-3-yl)benzyl)oxy)silane (Figure 4, **5d**). From ***tert*-butyldimethyl((3-(prop-1-en-1-yl)benzyl)oxy)silane** (**4d**) (52.5mg, 0.20 mmol) and **(bromoethynyl)triisopropylsilane** (**2a**) (78.4 mg, 0.30 mmol), the title compound was prepared following the general procedure **B** using $NiI_2{\cdot}xH_2O$ (3.8 mg, 5.0 mol%), (*S*)-**L*** (3.3 mg, 6.0 mol%), $K_3PO_4{\cdot}H_2O$ (115.1 mg, 2.5 equiv), NaI (60.0 mg, 2.0 equiv), $(MeO)_3SiH$ (64 μL, 0.5 mmol, 2.5 equiv) and anhydrous $PhCF_3$ (1.0 mL). The reaction mixture was stirred for 12 h at 0 °C. The crude material was purified by flash column chromatography (petroleum ether/EtOAc = 100:1) to provide the title compound as a colorless liquid in 91% yield (81.1 mg).

^{1}**H NMR** (500 MHz, $CDCl_3$) δ 7.38 – 7.33 (m, 1H), 7.33 – 7.28 (m, 2H), 7.26 – 7.22 (m, 1H), 4.78 – 4.72 (m, 2H), 3.68 (dd, *J* = 8.2, 5.5 Hz, 1H), 1.89 – 1.72 (m, 2H), 1.17 – 1.09 (m, 21H), 1.05 (t, *J* = 7.3 Hz, 3H), 0.98 (s, 9H), 0.13 (s, 3H), 0.13 (s, 3H);

^{13}**C NMR** (126 MHz, $CDCl_3$) δ 142.0, 141.5, 128.3, 126.3, 125.5, 124.5, 110.1, 83.2, 65.2, 40.5, 32.1, 26.1, 18.8, 18.5, 11.8, 11.5, –5.0;

HRMS (ESI) calcd. for $C_{27}H_{48}OSi_2Na$ $[M+Na]^+$ m/z 467.3136, found 467.3135;

IR (neat, cm^{-1}) 2929, 2863, 2169, 1077, 835, 670;

$[\alpha]_D^{18}$ = –15.2 (c = 1.33, $CHCl_3$); 90% *ee*;

HPLC analysis CHIRALCEL two connected OD-H columns, *n*-hexane/*iso*-propanol = 100/0, flow rate 0.5 mL/min, 220 nm UV detector, t_R (major) = 14.3 min, t_R (minor) = 14.8 min.

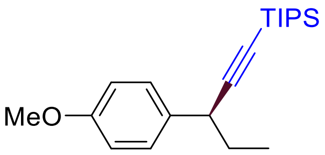

(*S*)-Triisopropyl(3-(4-methoxyphenyl)pent-1-yn-1-yl)silane (Figure 4, **5e**). From **(*E*)-1-methoxy-4-(prop-1-en-1-yl)benzene** (**4e**) (29.6 mg, 0.20 mmol) and **(bromoethynyl)triisopropylsilane** (**2a**) (78.4 mg, 0.30 mmol), the title compound was prepared following the general procedure **B** using $NiI_2\cdot xH_2O$ (3.8 mg, 5.0 mol%), (*S*)-**L*** (3.3 mg, 6.0 mol%), $K_3PO_4\cdot H_2O$ (115.1 mg, 2.5 equiv), NaI (60.0 mg, 2.0 equiv), $(MeO)_3SiH$ (64 μL, 0.5 mmol, 2.5 equiv) and anhydrous $PhCF_3$ (1.0 mL). The reaction mixture was stirred for 12 h at 0 °C. The crude material was purified by flash column chromatography (petroleum ether) to provide the title compound as a colorless liquid in 82% yield (54.1 mg).

^{1}H NMR (500 MHz, $CDCl_3$) δ 7.34 – 7.29 (m, 2H), 6.91 – 6.80 (m, 2H), 3.81 (s, 3H), 3.63 (dd, *J* = 8.2, 5.5 Hz, 1H), 1.84 – 1.75 (m, 1H), 1.78 – 1.67 (m, 1H), 1.15 – 1.07 (m, 21H), 1.03 (t, *J* = 7.3 Hz, 3H);

^{13}C NMR (126 MHz, $CDCl_3$) δ 158.3, 134.2, 128.5, 113.7, 110.3, 83.1, 55.3, 39.7, 32.2, 18.8, 11.7, 11.4;

HRMS (ESI) calcd. for $C_{21}H_{35}OSi$ $[M+H]^+$ m/z 331.2452, found 331.2458;

IR (neat, cm^{-1}) 2940, 2864, 2167, 1510, 1245, 670;

$[\alpha]_D^{18}$ = –16.6 (c = 1.05, $CHCl_3$); 94% *ee*;

HPLC analysis CHIRALCEL two connected OD-H columns, *n*-hexane/*iso*-propanol = 100/0, flow rate 0.5 mL/min, 220 nm UV detector, t_R (major) = 22.7min, t_R (minor)

= 26.5 min.

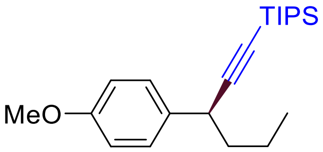

(*S*)-Triisopropyl(3-(4-methoxyphenyl)hex-1-yn-1-yl)silane (Figure 4, **5f**). From **1-(but-1-en-1-yl)-4-methoxybenzene** (**4f**) (32.4 mg, 0.20 mmol) and **(bromoethynyl)triisopropylsilane** (**2a**) (78.4 mg, 0.30 mmol), the title compound was prepared following the general procedure **B** using $NiI_2 \cdot xH_2O$ (3.8 mg, 5.0 mol%), (*S*)-**L*** (3.3 mg, 6.0 mol%), $K_3PO_4 \cdot H_2O$ (115.1 mg, 2.5 equiv), NaI (60.0 mg, 2.0 equiv), $(MeO)_3SiH$ (64 μL, 0.5 mmol, 2.5 equiv) and anhydrous $PhCF_3$ (1.0 mL). The reaction mixture was stirred for 12 h at 0 °C. The crude material was purified by flash column chromatography (petroleum ether) to provide the title compound as a colorless liquid in 82% yield (56.8 mg).

^{1}H NMR (500 MHz, $CDCl_3$) δ 7.35 – 7.30 (m, 2H), 6.90 – 6.86 (m, 2H), 3.82 (s, 3H), 3.69 (dd, *J* = 8.0, 6.3 Hz, 1H), 1.77 – 1.68 (m, 2H), 1.58 – 1.44 (m, 2H), 1.20 – 1.06 (m, 21H), 0.96 (t, *J* = 7.4 Hz, 3H);

^{13}C NMR (126 MHz, $CDCl_3$) δ 158.3, 134.5, 128.5, 113.7, 110.6, 82.8, 55.3, 41.4, 37.9, 20.5, 18.8, 13.9, 11.4;

HRMS (ESI) calcd. for $C_{22}H_{37}OSi$ $[M+H]^+$ m/z 345.2608, found 345.2609;

IR (neat, cm^{-1}) 2941, 2863, 2170, 1453, 696, 674;

$[\alpha]_D^{18}$ = –24.4 (c = 1.22, $CHCl_3$); 94% *ee*;

HPLC analysis CHIRALCEL two connected OD-H columns, *n*-hexane/*iso*-propanol = 100/0, flow rate 0.8 mL/min, 220 nm UV detector, t_R (major) = 13.4 min, t_R (minor) = 15.6 min.

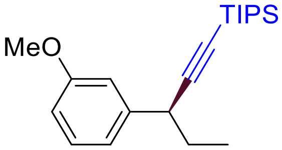

(*S*)-Triisopropyl(3-(3-methoxyphenyl)pent-1-yn-1-yl)silane (Figure 4, **5g**). From **1-**

methoxy-3-(prop-1-en-1-yl)benzene (**4g**) (29.6 mg, 0.20 mmol) and **(bromoethynyl)triisopropylsilane** (**2a**) (78.4 mg, 0.30 mmol), the title compound was prepared following the general procedure **B** using $NiI_2 \cdot xH_2O$ (3.8 mg, 5.0 mol%), (*S*)-**L*** (3.3 mg, 6.0 mol%), $K_3PO_4 \cdot H_2O$ (115.1 mg, 2.5 equiv), NaI (60.0 mg, 2.0 equiv), $(MeO)_3SiH$ (64 µL, 0.5 mmol, 2.5 equiv) and anhydrous $PhCF_3$ (1.0 mL). The reaction mixture was stirred for 12 h at 0 °C. The crude material was purified by flash column chromatography (petroleum ether) to provide the title compound as a colorless liquid in 83% yield (54.6 mg).

^{1}H NMR (500 MHz, $CDCl_3$) δ 7.27 – 7.23 (m, 1H), 7.05 – 7.02 (m, 1H), 6.99 – 6.94 (m, 1H), 6.80 (dd, *J* = 8.2, 2.5 Hz, 1H), 3.83 (s, 3H), 3.68 (dd, *J* = 8.3, 5.4 Hz, 1H), 1.88 – 1.71 (m, 2H), 1.17 – 1.06 (m, 21H), 1.06 (t, *J* = 7.4 Hz, 3H);

^{13}C NMR (126 MHz, $CDCl_3$) δ 159.7, 143.7, 129.3, 120.0, 112.9, 112.4, 109.8, 83.4, 55.2, 40.5, 32.1, 18.8, 11.8, 11.4;

HRMS (ESI) calcd. for $C_{21}H_{34}OSiNa$ $[M+Na]^+$ m/z 353.2271, found 353.2272;

IR (neat, cm^{-1}) 2940, 2864, 2168, 1487, 881, 665;

$[\alpha]_D^{20}$ = –26.5 (c = 1.59, $CHCl_3$); 92% *ee*;

HPLC analysis CHIRALCEL two connected OD-H columns, *n*-hexane/*iso*-propanol = 100/0, flow rate 0.8 mL/min, 220 nm UV detector, t_R (major) = 14.5 min, t_R (minor) = 16.0 min.

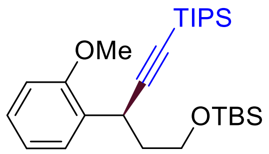

(*S*)-*tert*-Butyl((3-(2-methoxyphenyl)-5-(triisopropylsilyl)pent-4-yn-1-yl)oxy)dimethylsilane (Figure 4, **5h**). From ***tert*-butyl((3-(2-methoxyphenyl)allyl)oxy)dimethylsilane** (**4h**) (55.7 mg, 0.20 mmol) and **(bromoethynyl)triisopropylsilane** (**2a**) (78.4 mg, 0.30 mmol), the title compound was prepared following the general procedure **B** using $NiI_2 \cdot xH_2O$ (3.8 mg, 5.0 mol%), (*S*)-**L*** (3.3 mg, 6.0 mol%), $K_3PO_4 \cdot H_2O$ (115.1 mg, 2.5 equiv), NaI (60.0 mg, 2.0 equiv),

$(MeO)_3SiH$ (64 μL, 0.5 mmol, 2.5 equiv) and anhydrous $PhCF_3$ (1.0 mL). The reaction mixture was stirred for 12 h at 0 °C. The crude material was purified by flash column chromatography (petroleum ether/EtOAc = 100:1) to provide the title compound as a colorless liquid in 88% yield (81.3 mg).

^{1}H NMR (500 MHz, $CDCl_3$) δ 7.61 (dd, *J* = 7.5, 1.8 Hz, 1H), 7.23 – 7.18 (m, 1H), 7.00 – 6.92 (m, 1H), 6.84 (dd, *J* = 8.2, 1.1 Hz, 1H), 4.30 (dd, *J* = 10.0, 4.2 Hz, 1H), 3.93 – 3.86 (m, 1H), 3.84 – 3.78 (m, 4H), 2.06 – 1.94 (m, 1H), 1.80 – 1.67 (m, 1H), 1.15 – 1.05 (m, 21H), 0.92 (s, 9H), 0.08 (s, 3H), 0.07 (s, 3H);

^{13}C NMR (126 MHz, $CDCl_3$) δ 156.1, 130.4, 128.6, 127.7, 120.6, 110.2, 110.0, 82.7, 61.5, 55.3, 40.0, 29.1, 26.0, 18.9, 18.8, 18.4, 11.4, -5.0, -5.1;

HRMS (ESI) calcd. for $C_{27}H_{48}O_2Si_2Na$ $[M+Na]^+$ m/z 483.3085, found 483.3087;

IR (neat, cm^{-1}) 2940, 2863, 2166, 1243, 831, 665;

$[\alpha]_D^{18}$ = –30.6 (c = 1.15, $CHCl_3$); 90% *ee*;

HPLC analysis CHIRALCEL two connected OD-H columns, *n*-hexane/*iso*-propanol = 100/0, flow rate 0.5 mL/min, 220 nm UV detector, t_R (minor) = 15.0 min, t_R (major) = 19.1 min.

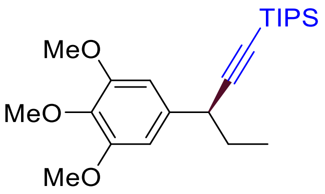

(*S*)-Triisopropyl(3-(3,4,5-trimethoxyphenyl)pent-1-yn-1-yl)silane (Figure 4, **5i**). From **1,2,3-trimethoxy-5-(prop-1-en-1-yl)benzene** (**4i**) (41.7 mg, 0.20 mmol) and **(bromoethynyl)triisopropylsilane** (**2a**) (78.4 mg, 0.30 mmol), the title compound was prepared following the general procedure **B** using $NiI_2·xH_2O$ (3.8 mg, 5.0 mol%), (*S*)-**L*** (3.3 mg, 6.0 mol%), $K_3PO_4·H_2O$ (115.1 mg, 2.5 equiv), NaI (60.0 mg, 2.0 equiv), $(MeO)_3SiH$ (64 μL, 0.5 mmol, 2.5 equiv) and anhydrous $PhCF_3$ (1.0 mL). The reaction mixture was stirred for 12 h at 0 °C. The crude material was purified by flash column chromatography (petroleum ether/EtOAc = 20:1) to provide the title compound as a colorless liquid in 65% yield (51.0 mg).

^{1}H NMR (500 MHz, $CDCl_3$) δ 6.63 (s, 2H), 3.85 (s, 6H), 3.83 (s, 3H), 3.61 (dd, *J* = 8.4, 5.2 Hz, 1H), 1.87 – 1.76 (m, 1H), 1.76 – 1.63 (m, 1H), 1.14 – 1.02 (m, 21H), 1.03 (t, *J* = 7.3 Hz, 3H);

^{13}C NMR (126 MHz, $CDCl_3$) δ 153.0, 137.8, 136.4, 109.7, 104.4, 83.7, 60.9, 56.0, 40.7, 32.3, 18.9, 18.8, 11.8, 11.4;

HRMS (ESI) calcd. for $C_{23}H_{38}O_3SiNa$ $[M+Na]^+$ m/z 413.2482, found 413.2481;

IR (neat, cm^{-1}) 2940, 2864, 2167, 1460, 1127, 674;

$[\alpha]_D^{18}$ = –19.5 (c = 1.51, $CHCl_3$); 88% *ee*;

HPLC analysis CHIRALCEL OD-H column, *n*-hexane/*iso*-propanol = 99/1, flow rate 0.5 mL/min, 220 nm UV detector, t_R (minor) = 10.5 min, t_R (major) = 11.1 min.

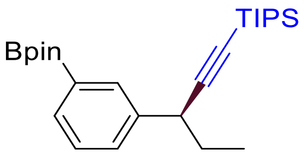

(*S*)-Triisopropyl(3-(3-(4,4,5,5-tetramethyl-1,3,2-dioxaborolan-2-yl)phenyl)pent-1-yn-1-yl)silane (Figure 4, **5j**). From **4,4,5,5-tetramethyl-2-(3-(prop-1-en-1-yl)phenyl)-1,3,2-dioxaborolane** (**4j**) (48.8 mg, 0.20 mmol) and **(bromoethynyl)triisopropylsilane** (**2a**) (78.4 mg, 0.30 mmol), the title compound was prepared following the general procedure **B** using $NiI_2 \cdot xH_2O$ (3.8 mg, 5.0 mol%), (*S*)-**L*** (3.3 mg, 6.0 mol%), $K_3PO_4 \cdot H_2O$ (115.1 mg, 2.5 equiv), NaI (60.0 mg, 2.0 equiv), $(MeO)_3SiH$ (64 μL, 0.5 mmol, 2.5 equiv) and anhydrous $PhCF_3$ (1.0 mL). The reaction mixture was stirred for 12 h at 0 °C. The crude material was purified by flash column chromatography (petroleum ether/EtOAc = 50:1) to provide the title compound as a colorless liquid in 82% yield (69.8 mg).

^{1}H NMR (500 MHz, $CDCl_3$) δ 7.90 – 7.86 (m, 1H), 7.72 – 7.67 (m, 1H), 7.57 – 7.48 (m, 1H), 7.39 – 7.32 (m, 1H), 3.69 (dd, *J* = 8.5, 5.4 Hz, 1H), 1.90 – 1.81 (m, 1H), 1.81 – 1.71 (m, 1H), 1.37 (s, 12H), 1.20 – 1.09 (m, 21H), 1.06 (t, *J* = 7.3 Hz, 3H);

^{13}C NMR (126 MHz, $CDCl_3$) δ 141.3, 134.1, 133.0, 130.5, 127.8, 110.0, 83.8, 83.4, 40.5, 32.1, 25.0, 24.9, 18.8, 11.9, 11.4;

HRMS (ESI) calcd. for $C_{26}H_{43}BO_2SiNa$ $[M+Na]^+$ m/z 449.3018, found 449.3017;

IR (neat, cm^{-1}) 2940, 2864, 2168, 1357, 882, 674;

$[\alpha]_D^{18}$ = –15.7 (c = 1.59, $CHCl_3$); 90% *ee*;

HPLC analysis CHIRALCEL two connected OD-H columns, *n*-hexane/*iso*-propanol = 100/0, flow rate 0.3 mL/min, 220 nm UV detector, t_R (major) = 28.2 min, t_R (minor) = 29.4 min.

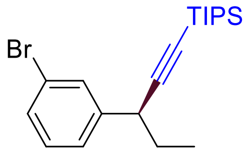

(*S*)-(3-(3-Bromophenyl)pent-1-yn-1-yl)triisopropylsilane (Figure 4, **5k**). From **1-bromo-3-(prop-1-en-1-yl)benzene** (**4k**) (39.4 mg, 0.20 mmol) and **(bromoethynyl)triisopropylsilane** (**2a**) (78.4 mg, 0.30 mmol), the title compound was prepared following the general procedure **B** using $NiBr_2$·diglyme (3.5 mg, 5.0 mol%), (*S*)-**L*** (3.3 mg, 6.0 mol%), $K_3PO_4 \cdot H_2O$ (115.1 mg, 2.5 equiv), NaI (90.0 mg, 3.0 equiv), $(MeO)_3SiH$ (64 μL, 0.5 mmol, 2.5 equiv) and anhydrous DCE (1.0 mL). The reaction mixture was stirred for 12 h at 0 °C. The crude material was purified by flash column chromatography (petroleum ether) to provide the title compound as a colorless liquid in 66% yield (49.6 mg).

^{1}H NMR (500 MHz, $CDCl_3$) δ 7.58 – 7.55 (m, 1H), 7.38 – 7.34 (m, 1H), 7.32 – 7.28 (m, 1H), 7.18 (t, *J* = 7.8 Hz, 1H), 3.63 (dd, *J* = 8.2, 5.4 Hz, 1H), 1.85 – 1.77 (m, 1H), 1.77 – 1.65 (m, 1H), 1.13 – 1.03 (m, 21H), 1.02 (t, *J* = 7.3 Hz, 3H);

^{13}C NMR (126 MHz, $CDCl_3$) δ 144.4, 130.9, 129.9, 129.7, 126.3, 122.5, 108.9, 84.3, 40.1, 31.9, 18.8, 11.7, 11.4;

HRMS (ESI) calcd. for $C_{20}H_{31}BrSiNa$ $[M+Na]^+$ m/z 401.1271, found 401.1272;

IR (neat, cm^{-1}) 2940, 2864, 2169, 1462, 881, 675;

$[\alpha]_D^{18}$ = –22.9 (c = 1.26, $CHCl_3$); 94% *ee*;

HPLC analysis CHIRALPAK IE-3 column, *n*-hexane/*iso*-propanol = 100/0, flow rate 0.3 mL/min, 220 nm UV detector, t_R (minor) = 13.3 min, t_R (major) = 13.8 min.

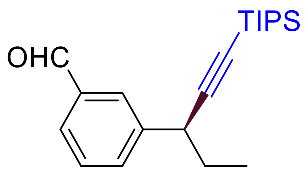

(*S*)-3-(1-(Triisopropylsilyl)pent-1-yn-3-yl)benzaldehyde (Figure 4, **5l**). From **3-(prop-1-en-1-yl)benzaldehyde** (**4l**) (29.2 mg, 0.20 mmol) and **(bromoethynyl)triisopropylsilane** (**2a**) (78.4 mg, 0.30 mmol), the title compound was prepared following the general procedure **B** using $NiI_2·xH_2O$ (3.8 mg, 5.0 mol%), (*S*)-**L*** (3.3 mg, 6.0 mol%), $K_3PO_4·H_2O$ (115.1 mg, 2.5 equiv), NaI (60.0 mg, 2.0 equiv), $(MeO)_3SiH$ (64 µL, 0.5 mmol, 2.5 equiv) and anhydrous $PhCF_3$ (1.0 mL). The reaction mixture was stirred for 12 h at 0 ^{o}C. The crude material was purified by flash column chromatography (petroleum ether/EtOAc = 50:1) to provide the title compound as a colorless liquid in 52% yield (34.4 mg).

^{1}H NMR (500 MHz, $CDCl_3$) δ 10.01 (s, 1H), 7.95 – 7.89 (m, 1H), 7.77 – 7.73 (m, 1H), 7.69 – 7.59 (m, 1H), 7.49 (t, *J* = 7.6 Hz, 1H), 3.75 (dd, *J* = 8.2, 5.5 Hz, 1H), 1.89 – 1.81 (m, 1H), 1.81 – 1.71 (m, 1H), 1.11 – 1.08 (m, 21H), 1.03 (t, *J* = 7.4 Hz, 3H);

^{13}C NMR (126 MHz, $CDCl_3$) δ 192.4, 143.3, 136.6, 133.8, 129.2, 129.1, 128.0, 108.9, 84.4, 40.2, 31.9, 18.8, 11.6, 11.4;

HRMS (ESI) calcd. for $C_{21}H_{32}OSiNa$ $[M+Na]^+$ m/z 351.2115, found 351.2113;

IR (neat, cm^{-1}) 2940, 2864, 2169, 1696, 738, 675;

$[\alpha]_D^{18}$ = –24.0 (c = 0.95, $CHCl_3$); 92% *ee*;

HPLC analysis CHIRALCEL two connected OD-H columns, *n*-hexane/*iso*-propanol = 100/0, flow rate 0.5 mL/min, 220 nm UV detector, t_R (major) = 38.3 min, t_R (minor) = 42.7 min.

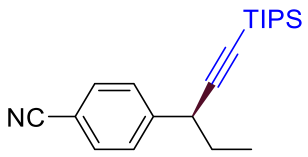

(*S*)-4-(1-(Triisopropylsilyl)pent-1-yn-3-yl)benzonitrile (Figure 4, **5m**). From **4-(prop-1-en-1-yl)benzonitrile** (**4m**) (28.6 mg, 0.20 mmol) and

(bromoethynyl)triisopropylsilane (**2a**) (78.4 mg, 0.30 mmol), the title compound was prepared following the general procedure **B** using $NiBr_2$·diglyme (3.5 mg, 5.0 mol%), (*S*)-**L*** (3.3 mg, 6.0 mol%), $K_3PO_4·H_2O$ (115.1 mg, 2.5 equiv), NaI (90.0 mg, 3.0 equiv), $(MeO)_3SiH$ (64 μL, 0.5 mmol, 2.5 equiv and anhydrous DCE (1.0 mL). The reaction mixture was stirred for 12 h at 0 °C. The crude material was purified by flash column chromatography (petroleum ether/EtOAc = 50:1) to provide the title compound as a colorless liquid in 62% yield (40.1 mg).

^{1}H NMR (500 MHz, $CDCl_3$) δ 7.64 – 7.58 (m, 2H), 7.51 – 7.44 (m, 2H), 3.71 (dd, *J* = 8.3, 5.5 Hz, 1H), 1.85 – 1.77 (m, 1H), 1.77 – 1.67 (m, 1H), 1.12 – 1.02 (m, 21H), 1.01 (t, *J* = 7.4 Hz, 3H);

^{13}C NMR (126 MHz, $CDCl_3$) δ 147.6, 132.3, 128.4, 119.0, 110.6, 108.1, 84.9, 40.6, 31.8, 18.7, 11.6, 11.3;

HRMS (ESI) calcd. for $C_{21}H_{31}NSiNa$ $[M+Na]^+$ m/z 348.2118 found 348.2121;

IR (neat, cm^{-1}) 2940, 2864, 2229, 2169, 882, 665;

$[\alpha]_D^{18}$ = –14.7 (c = 0.38, $CHCl_3$); 94% *ee*;

HPLC analysis CHIRALCEL two connected OD-H columns, *n*-hexane/*iso*-propanol = 100/0, flow rate 0.5 mL/min, 220 nm UV detector, t_R (minor) = 39.4 min, t_R (major) = 41.3 min.

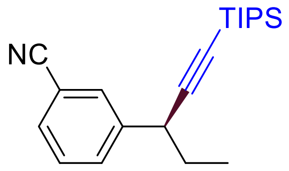

(*S*)-3-(1-(Triisopropylsilyl)pent-1-yn-3-yl)benzonitrile (Figure 4, **5n**). From **4-(prop-1-en-1-yl)benzonitrile** (**4n**) (28.6 mg, 0.20 mmol) and **(bromoethynyl)triisopropylsilane** (**2a**) (78.4 mg, 0.30 mmol), the title compound was prepared following the general procedure **B** using $NiI_2·xH_2O$ (3.8 mg, 5.0 mol%), (*S*)-**L*** (3.3 mg, 6.0 mol%), $K_3PO_4·H_2O$ (115.1 mg, 2.5 equiv), NaI (60.0 mg, 2.0 equiv), $(MeO)_3SiH$ (64 μL, 0.5 mmol, 2.5 equiv) and anhydrous DCE (1.0 mL). The reaction mixture was stirred for 12 h at 0 °C. The crude material was purified by flash column

chromatography (petroleum ether/EtOAc = 100:1) to provide the title compound as a colorless liquid in 65% yield (42.4 mg).

^{1}H NMR (500 MHz, $CDCl_3$) δ 7.70 – 7.67 (m, 1H), 7.63 – 7.59 (m, 1H), 7.55 – 7.51 (m, 1H), 7.42 (t, *J* = 7.7 Hz, 1H), 3.69 (dd, *J* = 8.2, 5.5 Hz, 1H), 1.86 – 1.67 (m, 2H), 1.12 – 1.06 (m, 21H), 1.02 (t, *J* = 7.3 Hz, 3H);

^{13}C NMR (126 MHz, $CDCl_3$) δ 143.6, 132.2, 131.4, 130.5, 129.2, 119.0, 112.5, 108.1, 85.0, 40.1, 31.8, 18.7, 11.6, 11.4;

HRMS (ESI) calcd. for $C_{21}H_{32}NSi$ $[M+H]^+$ m/z 326.2299 found 326.2296;

IR (neat, cm^{-1}) 2964, 2864, 2231, 2169, 882, 664;

$[\alpha]_D^{18}$ = –21.4 (c = 0.29, $CHCl_3$); 94% *ee*;

HPLC analysis CHIRALPAK AD-H column, *n*-hexane/*iso*-propanol = 100/0, flow rate 0.8 mL/min, 220 nm UV detector, t_R (minor) = 13.5 min, t_R (major) = 19.3 min.

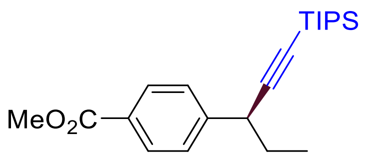

Methyl (*S*)-4-(1-(triisopropylsilyl)pent-1-yn-3-yl)benzoate (Figure 4, **5o**). From **methyl 4-(prop-1-en-1-yl)benzoate** (**4o**) (35.2 mg, 0.20 mmol) and **(bromoethynyl)triisopropylsilane** (**2a**) (78.4 mg, 0.30 mmol), the title compound was prepared following the general procedure **B** using $NiBr_2$·diglyme (3.5 mg, 5.0 mol%), (*S*)-**L*** (3.3 mg, 6.0 mol%), $K_3PO_4 \cdot H_2O$ (115.1 mg, 2.5 equiv), NaI (90.0 mg, 3.0 equiv), $(MeO)_3SiH$ (64 μL, 0.5 mmol, 2.5 equiv) and anhydrous DCE (1.0 mL). The reaction mixture was stirred for 12 h at 0 °C. The crude material was purified by flash column chromatography (petroleum ether/EtOAc = 50:1) to provide the title compound as a colorless liquid in 65% yield (46.5 mg).

^{1}H NMR (500 MHz, $CDCl_3$) δ 8.09 – 7.92 (m, 2H), 7.48 – 7.41 (m, 2H), 3.90 (s, 3H), 3.70 (dd, *J* = 8.3, 5.5 Hz, 1H), 1.86 – 1.64 (m, 2H), 1.17 – 0.98 (m, 21H), 1.02 (t, *J* = 7.3 Hz, 3H);

^{13}C NMR (126 MHz, $CDCl_3$) δ 167.1, 147.4, 129.8, 128.6, 127.7, 108.9, 84.1, 52.1,

40.5, 31.9, 18.7, 11.6, 11.4;

HRMS (ESI) calcd. for $C_{22}H_{35}O_2Si$ $[M+H]^+$ m/z 359.2401, found 359.2405;

IR (neat, cm^{-1}) 2941, 2864, 2168, 1715, 1276, 663;

$[\alpha]_D^{18}$ = –15.6 (c = 1.45, $CHCl_3$); 96% *ee*;

HPLC analysis CHIRALCEL two connected OD-H columns, *n*-hexane/*iso*-propanol = 100/0, flow rate 0.8 mL/min, 220 nm UV detector, t_R (major) = 27.8 min, t_R (minor) = 30.7 min.

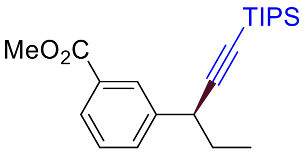

Methyl (*S*)-3-(1-(triisopropylsilyl)pent-1-yn-3-yl)benzoate (Figure 4, **5p**). From **methyl 3-(prop-1-en-1-yl)benzoate** (**4p**) (35.2 mg, 0.20 mmol) and **(bromoethynyl)triisopropylsilane** (**2a**) (78.4 mg, 0.30 mmol), the title compound was prepared following the general procedure **B** using $NiI_2{\cdot}xH_2O$ (3.8 mg, 5.0 mol%), (*S*)-**L*** (3.3 mg, 6.0 mol%), $K_3PO_4{\cdot}H_2O$ (115.1 mg, 2.5 equiv), NaI (60.0 mg, 2.0 equiv), $(MeO)_3SiH$ (64 μL, 0.5 mmol, 2.5 equiv) and anhydrous $PhCF_3$ (1.0 mL). The reaction mixture was stirred for 12 h at 0 °C. The crude material was purified by flash column chromatography (petroleum ether/EtOAc = 50:1) to provide the title compound as a colorless liquid in 83% yield (59.6mg).

^{1}H NMR (500 MHz, $CDCl_3$) δ 8.13 – 8.06 (m, 1H), 7.94 – 7.89 (m, 1H), 7.62 – 7.53 (m, 1H), 7.39 (t, *J* = 7.7 Hz, 1H), 3.91 (s, 3H), 3.72 (dd, *J* = 8.2, 5.4 Hz, 1H), 1.88 – 1.70 (m, 2H), 1.12 – 1.08 (m, 21H), 1.02 (t, *J* = 7.3 Hz, 3H);

^{13}C NMR (126 MHz, $CDCl_3$) δ 167.2, 142.4, 132.2, 130.3, 128.9, 128.4, 128.0, 109.2, 84.2, 52.1, 40.3, 32.0, 18.8, 11.7, 11.4;

HRMS (ESI) calcd. for $C_{22}H_{35}O_2Si$ $[M+H]^+$ m/z 359.2401, found 359.2405;

IR (neat, cm^{-1}) 2941, 2864, 2169, 1726, 1281, 665;

$[\alpha]_D^{18}$ = –31.5 (c = 0.59, $CHCl_3$); 94% *ee*;

HPLC analysis CHIRALCEL OD-H column, *n*-hexane/*iso*-propanol = 100/0, flow

rate 0.5 mL/min, 220 nm UV detector, t_R (major) = 20.5 min, t_R (minor) = 22.2 min.

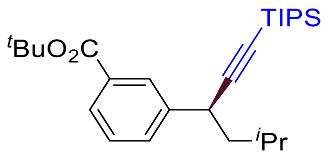

tert*-Butyl (*S*)-3-(5-methyl-1-(triisopropylsilyl)hex-1-yn-3-yl)benzoate** (Figure 4, **5q**). From ***tert*-butyl 3-(3-methylbut-1-en-1-yl)benzoate** (**4q**) (49.3 mg, 0.20 mmol) and **(bromoethynyl)triisopropylsilane** (**2a**) (78.4 mg, 0.30 mmol), the title compound was prepared following the general procedure **B** using $NiI_2·xH_2O$ (3.8 mg, 5.0 mol%), (*S*)-**L (3.3 mg, 6.0 mol%), $K_3PO_4·H_2O$ (115.1 mg, 2.5 equiv), NaI (60.0 mg, 2.0 equiv), $(MeO)_3SiH$ (64 μL, 0.5 mmol, 2.5 equiv) and anhydrous $PhCF_3$ (1.0 mL). The reaction mixture was stirred for 12 h at 0 °C. The crude material was purified by flash column chromatography (petroleum ether/EtOAc = 100:1) to provide the title compound as a colorless liquid in 71% yield (60.5 mg).

^{1}H NMR (500 MHz, $CDCl_3$) δ 7.97 – 7.93 (m, 1H), 7.87 – 7.82 (m, 1H), 7.57 – 7.53 (m, 1H), 7.36 (t, *J* = 7.7 Hz, 1H), 3.76 (dd, *J* = 10.1, 5.7 Hz, 1H), 1.95 – 1.84 (m, 1H), 1.74 – 1.67 (m, 1H), 1.59 (s, 9H), 1.51 – 1.40 (m, 1H), 1.12 – 0.98 (m, 21H), 0.97 (d, *J* = 6.6 Hz, 3H), 0.94 (d, *J* = 6.7 Hz, 3H);

^{13}C NMR (126 MHz, $CDCl_3$) δ 165.9, 143.1, 132.3, 131.6, 128.5, 128.4, 127.8, 109.7, 83.4, 81.1, 48.5, 37.1, 28.3, 26.3, 23.2, 21.7, 18.9, 18.8, 11.4;

HRMS (ESI) calcd. for $C_{27}H_{44}O_2SiNa$ $[M+Na]^+$ m/z 451.3003, found 451.3004;

IR (neat, cm^{-1}) 2941, 2865, 2168, 1715, 1291, 1159;

$[\alpha]_D^{18}$ = –26.2 (c = 1.27, $CHCl_3$); 94% *ee*;

HPLC analysis CHIRALCEL two connected OD-H columns, *n*-hexane/*iso*-propanol = 100/0, flow rate 0.5 mL/min, 220 nm UV detector, t_R (minor) = 19.2 min, t_R (major) = 19.9 min.

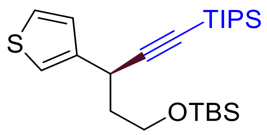

(*R*)-*tert*-Butyldimethyl((3-(thiophen-3-yl)-5-(triisopropylsilyl)pent-4-yn-1-yl)oxy)silane (Figure 4, **5r**). From ***tert*-butyldimethyl((3-(thiophen-3-yl)allyl)oxy)silane** (**4r**) (50.9 mg, 0.20 mmol) and **(bromoethynyl)triisopropylsilane** (**2a**) (78.4 mg, 0.30 mmol), the title compound was prepared following the general procedure **B** using $NiI_2{\cdot}xH_2O$ (3.8 mg, 5.0 mol%), (*S*)-**L*** (3.3 mg, 6.0 mol%), $K_3PO_4{\cdot}H_2O$ (115.1 mg, 2.5 equiv), NaI (60.0 mg, 2.0 equiv), $(MeO)_3SiH$ (64 μL, 0.5 mmol, 2.5 equiv) and anhydrous $PhCF_3$ (1.0 mL). The reaction mixture was stirred for 12 h at 0 °C. The crude material was purified by flash column chromatography (petroleum ether) to provide the title compound as a colorless liquid in 88% yield (76.8 mg).

^{1}H NMR (500 MHz, $CDCl_3$) δ 7.29 – 7.25 (m, 1H), 7.21 – 7.17 (m, 1H), 7.07 (dd, *J* = 5.0, 1.3 Hz, 1H), 3.98 (dd, *J* = 9.6, 5.1 Hz, 1H), 3.90 – 3.83 (m, 1H), 3.77 – 3.69 (m, 1H), 2.07 – 1.97 (m, 1H), 1.96 – 1.84 (m, 1H), 1.15 – 1.03 (m, 21H), 0.91 (s, 9H), 0.08 (s, 3H), 0.07 (s, 3H);

^{13}C NMR (126 MHz, $CDCl_3$) δ 142.5, 127.2, 125.7, 120.9, 109.5, 82.8, 60.7, 40.8, 30.6, 26.1, 18.8, 18.4, 11.4, –5.1, –5.2;

HRMS (ESI) calcd. for $C_{24}H_{44}OSSi_2Na$ $[M+Na]^+$ m/z 459.2544, found 459.2549;

IR (neat, cm^{-1}) 2941, 2863, 2168, 1103, 831, 775;

$[\alpha]_D^{18}$ = –22.0 (c = 1.10, $CHCl_3$); 82% *ee*;

HPLC analysis CHIRALCEL two connected OD-H columns, *n*-hexane/*iso*-propanol = 100/0, flow rate 0.3 mL/min, 220 nm UV detector, t_R (major) = 24.5 min, t_R (minor) = 25.4 min.

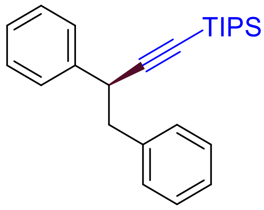

(*S*)-(3,4-Diphenylbut-1-yn-1-yl)triisopropylsilane (Figure 4, **5s**). From **(*E*)-1,2-diphenylethene** (**4s**) (36.1 mg, 0.20 mmol) and **(bromoethynyl)triisopropylsilane** (**2a**) (78.4 mg, 0.30 mmol), the title compound was prepared following the general procedure **B** using $NiI_2 \cdot xH_2O$ (3.8 mg, 5.0 mol%), (*S*)-**L*** (3.3 mg, 6.0 mol%), $K_3PO_4 \cdot H_2O$ (115.1 mg, 2.5 equiv), NaI (60.0 mg, 2.0 equiv), $(MeO)_3SiH$ (64 μL, 0.5 mmol, 2.5 equiv) and anhydrous $PhCF_3$ (1.0 mL). The reaction mixture was stirred for 12 h at 0 °C. The crude material was purified by flash column chromatography (petroleum ether/EtOAc = 100:1) to provide the title compound as a colorless liquid in 73% yield (52.9 mg).

^{1}H NMR (500 MHz, $CDCl_3$) δ 7.39 – 7.31 (m, 4H), 7.29 – 7.21 (m, 4H), 7.20 – 7.15 (m, 2H), 4.00 (dd, *J* = 8.5, 6.1 Hz, 1H), 3.16 – 2.93 (m, 2H), 1.13 – 0.85 (m, 21H);

^{13}C NMR (126 MHz, $CDCl_3$) δ 141.5, 138.9, 129.6, 128.4, 128.0, 127.8, 126.8, 126.4, 109.2, 84.5, 45.5, 41.3, 18.7, 11.4;

HRMS (ESI) calcd. for $C_{25}H_{34}SiNa$ $[M+Na]^+$ m/z 385.2322, found 385.2325;

IR (neat, cm^{-1}) 2941, 2863, 2170, 1453, 882, 696;

$[\alpha]_D^{18}$ = –20.7 (c = 1.16, $CHCl_3$); 94% *ee*;

HPLC analysis CHIRALCEL two connected OD-H columns, *n*-hexane/*iso*-propanol = 100/0, flow rate 0.8 mL/min, 220 nm UV detector, t_R (minor) = 16.7 min, t_R (major) = 19.6 min.

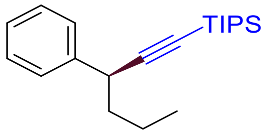

(*S*)-Triisopropyl(3-phenylhex-1-yn-1-yl)silane (Figure 4, **5t**). From **but-1-en-1-ylbenzene** (**4t**) (26.4 mg, 0.20 mmol) and **(bromoethynyl)triisopropylsilane** (**2a**)

(78.4 mg, 0.30 mmol), the title compound was prepared following the general procedure **B** using $NiI_2 \cdot xH_2O$ (3.8 mg, 5.0 mol%), (*S*)-**L*** (3.3 mg, 6.0 mol%), $K_3PO_4 \cdot H_2O$ (115.1 mg, 2.5 equiv), NaI (60.0 mg, 2.0 equiv), $(MeO)_3SiH$ (64 μL, 0.5 mmol, 2.5 equiv) and anhydrous $PhCF_3$ (1.0 mL). The reaction mixture was stirred for 12 h at 0 °C. The crude material was purified by flash column chromatography (petroleum ether) to provide the title compound as a colorless liquid in 81% yield (50.9 mg).

^{1}H NMR (500 MHz, $CDCl_3$) δ 7.42 – 7.37 (m, 2H), 7.35 – 7.29 (m, 2H), 7.25 – 7.18 (m, 1H), 3.72 (dd, *J* = 7.9, 6.5 Hz, 1H), 1.78 – 1.65 (m, 2H), 1.58 – 1.41 (m, 2H), 1.10 (t, *J* = 2.8 Hz, 21H), 0.94 (t, *J* = 7.4 Hz, 3H);

^{13}C NMR (126 MHz, $CDCl_3$) δ 142.4, 128.4, 127.6, 126.5, 110.2, 83.1, 41.3, 38.8, 20.6, 18.8, 13.9, 11.4;

HRMS (ESI) calcd. for $C_{21}H_{34}SiNa$ $[M+Na]^+$ m/z 337.2322, found 337.2321;

IR (neat, cm^{-1}) 2940, 2864, 2167, 1462, 882, 670;

$[\alpha]_D^{18}$ = –18.2 (c = 1.44, $CHCl_3$); 92% *ee*;

HPLC analysis CHIRALCEL two connected OD-H columns, *n*-hexane/*iso*-propanol = 100/0, flow rate 0.3 mL/min, 220 nm UV detector, t_R (major) = 24.5 min, t_R (minor) = 25.2 min.

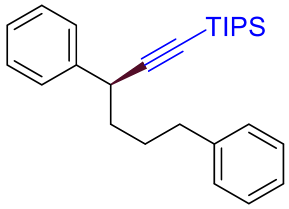

(*S*)-(3,6-Diphenylhex-1-yn-1-yl)triisopropylsilane (Figure 4, **5u**). From **but-1-ene-1,4-diyldibenzene** (**4u**) (41.7 mg, 0.20 mmol) and **(bromoethynyl)triisopropylsilane** (**2a**) (78.4 mg, 0.30 mmol), the title compound was prepared following the general procedure **B** using $NiI_2 \cdot xH_2O$ (3.8 mg, 5.0 mol%), (*S*)-**L*** (3.3 mg, 6.0 mol%), $K_3PO_4 \cdot H_2O$ (115.1 mg, 2.5 equiv), NaI (60.0 mg, 2.0 equiv), $(MeO)_3SiH$ (64 μL, 0.5 mmol, 2.5 equiv) and anhydrous $PhCF_3$ (1.0 mL). The reaction mixture was stirred for

12 h at 0 °C. The crude material was purified by flash column chromatography (petroleum ether) to provide the title compound as a colorless liquid in 90% yield (70.2 mg).

^{1}H NMR (500 MHz, $CDCl_3$) δ 7.43 – 7.38 (m, 2H), 7.36 – 7.27 (m, 4H), 7.27 – 7.16 (m, 4H), 3.77 (dd, *J* = 7.8, 5.7 Hz, 1H), 2.73 – 2.56 (m, 2H), 1.96 – 1.72 (m, 4H), 1.15 – 1.10 (m, 21H);

^{13}C NMR (126 MHz, $CDCl_3$) δ 142.3, 142.1, 128.6, 128.5, 128.4, 127.5, 126.6, 125.8, 109.9, 83.5, 38.8, 38.6, 35.6, 29.1, 18.9, 18.8, 11.4;

HRMS (ESI) calcd. for $C_{27}H_{38}SiNa$ $[M+Na]^+$ m/z 413.2635, found 413.2637;

IR (neat, cm^{-1}) 2940, 2863, 2168, 1453, 882, 696;

$[\alpha]_D^{18}$ = –25.3 (c = 1.37, $CHCl_3$); 92% *ee*;

HPLC analysis CHIRALCEL two connected OD-H columns, *n*-hexane/*iso*-propanol = 100/0, flow rate 0.8 mL/min, 220 nm UV detector, t_R (minor) = 16.4 min, t_R (major) = 18.0 min.

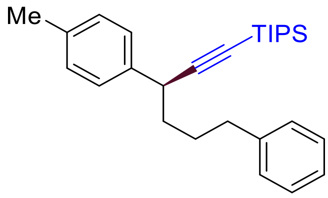

(*S*)-Triisopropyl(6-phenyl-3-(*p*-tolyl)hex-1-yn-1-yl)silane (Figure 4, **5v**). From **1-methyl-4-(4-phenylbut-1-en-1-yl)benzene** (**4v**) (44.5 mg, 0.20 mmol) and **(bromoethynyl)triisopropylsilane** (**2a**) (78.4 mg, 0.30 mmol), the title compound was prepared following the general procedure **B** using $NiI_2 \cdot xH_2O$ (3.8 mg, 5.0 mol%), (*S*)-**L*** (3.3 mg, 6.0 mol%), $K_3PO_4 \cdot H_2O$ (115.1 mg, 2.5 equiv), NaI (60.0 mg, 2.0 equiv), $(MeO)_3SiH$ (64 μL, 0.5 mmol, 2.5 equiv) and anhydrous $PhCF_3$ (1.0 mL). The reaction mixture was stirred for 12 h at 0 °C. The crude material was purified by flash column chromatography (petroleum ether) to provide the title compound as a colorless liquid in 84% yield (67.7 mg).

^{1}H NMR (500 MHz, $CDCl_3$) δ 7.33 – 7.28 (m, 4H), 7.23 – 7.18 (m, 3H), 7.16 (d, *J* = 7.8 Hz, 2H), 3.74 (dd, *J* = 7.8, 5.9 Hz, 1H), 2.75 – 2.61 (m, 2H), 2.37 (s, 3H), 1.95 – 1.84 (m, 2H), 1.83 – 1.76 (m, 2H), 1.16 – 1.10 (m, 21H);

^{13}C NMR (126 MHz, $CDCl_3$) δ 142.4, 139.1, 136.1, 129.1, 128.5, 128.3, 127.4, 125.7, 110.1, 83.2, 38.7, 38.4, 35.6, 29.1, 21.1, 18.8, 11.4;

HRMS (ESI) calcd. for $C_{28}H_{40}SiNa$ $[M+Na]^+$ m/z 427.2791, found 427.2791;

IR (neat, cm^{-1}) 2940, 2862, 2168, 1454, 882, 675;

$[\alpha]_D^{18}$ = –21.3 (c = 1.26, $CHCl_3$); 94% *ee*;

HPLC analysis CHIRALCEL two connected OD-H columns, *n*-hexane/*iso*-propanol = 100/0, flow rate 0.8 mL/min, 220 nm UV detector, t_R (minor) = 15.6 min, t_R (major) = 18.9 min.

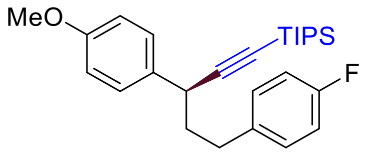

(*S*)-(5-(4-Fluorophenyl)-3-(4-methoxyphenyl)pent-1-yn-1-yl)triisopropylsilane (Figure 4, **5w**). From **1-fluoro-4-(3-(4-methoxyphenyl)allyl)benzene** (**4w**) (48.5 mg, 0.20 mmol) and **(bromoethynyl)triisopropylsilane** (**2a**) (78.4 mg, 0.30 mmol), the title compound was prepared following the general procedure **B** using $NiI_2 \cdot xH_2O$ (3.8 mg, 5.0 mol%), (*S*)-**L*** (3.3 mg, 6.0 mol%), $K_3PO_4 \cdot H_2O$ (115.1 mg, 2.5 equiv), NaI (60.0 mg, 2.0 equiv), $(MeO)_3SiH$ (64 μL, 0.5 mmol, 2.5 equiv) and anhydrous $PhCF_3$ (1.0 mL). The reaction mixture was stirred for 12 h at 0 °C. The crude material was purified by flash column chromatography (petroleum ether/EtOAc = 100:1) to provide the title compound as a colorless liquid in 89% yield (75.7 mg).

^{1}H NMR (500 MHz, $CDCl_3$) δ 7.36 – 7.29 (m, 2H), 7.19 – 7.13 (m, 2H), 7.02 – 6.95 (m, 2H), 6.93 – 6.85 (m, 2H), 3.82 (s, 3H), 3.70 – 3.64 (m, 1H), 2.87 – 2.76 (m, 2H), 2.05 – 1.96 (m, 2H), 1.21 – 1.09 (m, 21H);

^{13}C NMR (126 MHz, $CDCl_3$) δ 161.4 (d, *J* = 243.8 Hz), 158.4, 137.5 (d, *J* = 3.1 Hz), 133.9, 129.9 (d, *J* = 7.8 Hz), 128.4, 115.2 (d, *J* = 20.9 Hz), 113.9, 109.9, 83.8, 55.3, 41.1, 37.5, 32.8, 18.8, 11.4;

^{19}F NMR (471 MHz, $CDCl_3$) δ –117.6;

HRMS (ESI) calcd. for $C_{27}H_{37}FOSiNa$ $[M+Na]^+$ m/z 447.2490, found 447.2489;

IR (neat, cm^{-1}) 2941, 2863, 2168, 1508, 1246, 825;

$[\alpha]_D^{18}$ = –29.5 (c = 1.35, $CHCl_3$); 94% *ee*;

HPLC analysis CHIRALCEL two connected OD-H columns, *n*-hexane/*iso*-propanol = 100/0, flow rate 0.5 mL/min, 220 nm UV detector, t_R (major) = 64.1 min, t_R (minor) = 81.1 min.

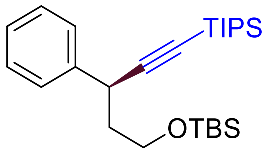

(*S*)-*tert*-Butyldimethyl((3-phenyl-5-(triisopropylsilyl)pent-4-yn-1-yl)oxy)silane (Figure 4, **5x**). From ***tert*-butyl(cinnamyloxy)dimethylsilane** (**4x**) (49.7 mg, 0.20 mmol) and **(bromoethynyl)triisopropylsilane** (**2a**) (78.4 mg, 0.30 mmol), the title compound was prepared following the general procedure **B** using $NiI_2 \cdot xH_2O$ (3.8 mg, 5.0 mol%), (*S*)-**L*** (3.3 mg, 6.0 mol%), $K_3PO_4 \cdot H_2O$ (115.1 mg, 2.5 equiv), NaI (60.0 mg, 2.0 equiv), $(MeO)_3SiH$ (64 μL, 0.5 mmol, 2.5 equiv) and anhydrous $PhCF_3$ (1.0 mL). The reaction mixture was stirred for 12 h at 0 °C. The crude material was purified by flash column chromatography (petroleum ether) to provide the title compound as a colorless liquid in 70% yield (60.3 mg).

^{1}H NMR (500 MHz, $CDCl_3$) δ 7.44 – 7.39 (m, 2H), 7.35 – 7.30 (m, 2H), 7.25 – 7.21 (m, 1H), 3.92 (dd, *J* = 9.4, 5.4 Hz, 1H), 3.90 – 3.84 (m, 1H), 3.76 – 3.70 (m, 1H), 2.04 – 1.88 (m, 2H), 1.19 – 1.06 (m, 21H), 0.93 (s, 9H), 0.09 (s, 3H), 0.08 (s, 3H);

^{13}C NMR (126 MHz, $CDCl_3$) δ 142.1, 128.5, 127.6, 126.6, 109.7, 83.4, 60.8, 42.2, 35.2, 26.1, 18.9, 18.8, 18.4, 11.4, –5.1, –5.2;

HRMS (ESI) calcd. for $C_{26}H_{47}OSi_2$ $[M+H]^+$ m/z 431.3160, found 431.3154;

IR (neat, cm^{-1}) 2942, 2864, 2172, 1741, 1247, 665;

$[\alpha]_D^{18}$ = –24.4 (c = 1.24, $CHCl_3$); 90% *ee*;

HPLC analysis CHIRALCEL two connected OD-H columns, *n*-hexane/*iso*-propanol = 100/0, flow rate 0.5 mL/min, 220 nm UV detector, t_R (minor) = 14.3 min, t_R (major) = 15.0 min.

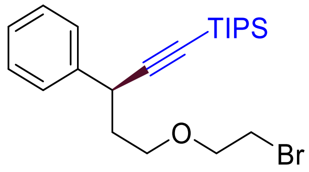

(*S*)-(5-(2-Bromoethoxy)-3-phenylpent-1-yn-1-yl)triisopropylsilane (Figure 4, **5y**). From **(*E*)-(3-(2-bromoethoxy)prop-1-en-1-yl)benzene** (**4y**) (48.2 mg, 0.20 mmol) and **(bromoethynyl)triisopropylsilane** (**2a**) (78.4 mg, 0.30 mmol), the title compound was prepared following the general procedure **B** using $NiI_2 \cdot xH_2O$ (3.8 mg, 5.0 mol%), (*S*)-**L*** (3.3 mg, 6.0 mol%), $K_3PO_4 \cdot H_2O$ (115.1 mg, 2.5 equiv), NaI (60.0 mg, 2.0 equiv), $(MeO)_3SiH$ (64 μL, 0.5 mmol, 2.5 equiv) and anhydrous $PhCF_3$ (1.0 mL). The reaction mixture was stirred for 12 h at 0 °C. The crude material was purified by flash column chromatography (petroleum ether/EtOAc = 100:1) to provide the title compound as a colorless liquid in 81% yield (68.5 mg).

^{1}H NMR (500 MHz, $CDCl_3$) δ 7.44 – 7.40 (m, 2H), 7.36 – 7.30 (m, 2H), 7.26 – 7.22 (m, 1H), 3.95 (dd, *J* = 9.1, 6.0 Hz, 1H), 3.76 (t, *J* = 6.2 Hz, 2H), 3.74 – 3.69 (m, 1H), 3.60 – 3.53 (m, 1H), 3.48 (t, *J* = 6.2 Hz, 2H), 2.09 – 1.90 (m, 2H), 1.22 – 0.89 (m, 21H);

^{13}C NMR (126 MHz, $CDCl_3$) δ 141.6, 128.5, 127.6, 126.8, 109.4, 83.6, 70.9, 68.6, 38.9, 35.4, 30.5, 18.8, 11.4;

HRMS (ESI) calcd. for $C_{22}H_{36}BrOSi$ $[M+H]^+$ m/z 423.1713, found 423.1712;

IR (neat, cm^{-1}) 2941, 2863, 2167, 1113, 882, 665;

$[\alpha]_D^{18}$ = –20.7 (c = 1.47, $CHCl_3$); 88% *ee*;

HPLC analysis CHIRALCEL OD-H column, *n*-hexane/*iso*-propanol = 99/1, flow rate 0.5 mL/min, 220 nm UV detector, t_R (minor) = 7.1min, t_R (major) = 11.6 min.

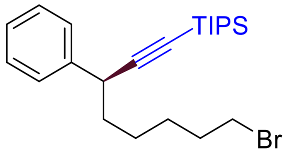

(*S*)-(8-Bromo-3-phenyloct-1-yn-1-yl)triisopropylsilane (Figure 4, **5z**). From **(6-bromohex-1-en-1-yl)benzene** (**4z**) (47.8 mg, 0.20 mmol) and **(bromoethynyl)triisopropylsilane** (**2a**) (78.4 mg, 0.30 mmol), the title compound was prepared following the general procedure **B** using NiI_2·xH_2O (3.8 mg, 5.0 mol%), (*S*)-**L*** (3.3 mg, 6.0 mol%), K_3PO_4·H_2O (115.1 mg, 2.5 equiv), NaI (60.0 mg, 2.0 equiv), $(MeO)_3SiH$ (64 µL, 0.5 mmol, 2.5 equiv) and anhydrous $PhCF_3$ (1.0 mL). The reaction mixture was stirred for 12 h at 0 °C. The crude material was purified by flash column chromatography (petroleum ether) to provide the title compound as a colorless liquid in 62% yield (52.0 mg).

^{1}H NMR (500 MHz, $CDCl_3$) δ 7.40 – 7.36 (m, 2H), 7.34 – 7.29 (m, 2H), 7.25 – 7.20 (m, 1H), 3.71 (dd, *J* = 8.2, 6.1 Hz, 1H), 3.39 (t, *J* = 6.8 Hz, 2H), 1.89 – 1.80 (m, 2H), 1.77 – 1.70 (m, 2H), 1.54 – 1.40 (m, 4H), 1.16 – 1.04 (m, 21H);

^{13}C NMR (126 MHz, $CDCl_3$) δ 142.1, 128.4, 127.5, 126.6, 109.8, 83.5, 38.9, 38.8, 33.9, 32.8, 27.9, 26.5, 18.8, 11.4;

HRMS (ESI) calcd. for $C_{23}H_{38}BrSi$ $[M+H]^+$ m/z 421.1921, found 421.1922;

IR (neat, cm^{-1}) 2941, 2864, 2169, 1735, 1152, 674;

$[\alpha]_D^{18}$ = –16.8 (c = 0.57, $CHCl_3$); 94% *ee*;

HPLC analysis CHIRALCEL two connected OD-H columns, *n*-hexane/*iso*-propanol = 100/0, flow rate 0.5 mL/min, 220 nm UV detector, t_R (major) = 28.3 min, t_R (minor) = 36.2 min.

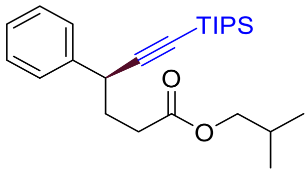

Isobutyl (*S*)-4-phenyl-6-(triisopropylsilyl)hex-5-ynoate (Figure 4, **5a'**). From **isobutyl (*E*)-4-phenylbut-3-enoate** (**4a'**) (43.7 mg, 0.20 mmol) and **(bromoethynyl)triisopropylsilane** (**2a**) (78.4 mg, 0.30 mmol), the title compound was prepared following the general procedure **B** using $NiI_2{\cdot}xH_2O$ (3.8 mg, 5.0 mol%), (*S*)-**L*** (3.3 mg, 6.0 mol%), $K_3PO_4{\cdot}H_2O$ (115.1 mg, 2.5 equiv), NaI (60.0 mg, 2.0 equiv), $(MeO)_3SiH$ (64 μL, 0.5 mmol, 2.5 equiv) and anhydrous $PhCF_3$ (1.0 mL). The reaction mixture was stirred for 12 h at 0 °C. The crude material was purified by flash column chromatography (petroleum ether/EtOAc = 100:1) to provide the title compound as a colorless liquid in 67% yield (53.9 mg).

^{1}H NMR (500 MHz, $CDCl_3$) δ 7.42 – 7.37 (m, 2H), 7.35 – 7.30 (m, 2H), 7.26 – 7.20 (m, 1H), 3.90 – 3.75 (m, 3H), 2.61 – 2.53 (m, 1H), 2.52 – 2.41 (m, 1H), 2.17 – 2.08 (m, 1H), 2.04 – 1.95 (m, 1H), 1.95 – 1.85 (m, 1H), 1.13 – 0.96 (m, 21H), 0.93 (s, 3H), 0.92 (s, 3H);

^{13}C NMR (126 MHz, $CDCl_3$) δ 173.4, 141.2, 128.6, 127.6, 126.9, 108.8, 84.4, 70.6, 38.1, 33.9, 31.9, 27.8, 19.2, 18.8, 11.4;

HRMS (ESI) calcd. for $C_{25}H_{41}O_2Si$ $[M+H]^+$ m/z 401.2870, found 401.2871;

IR (neat, cm^{-1}) 2942, 2864, 2173, 1742, 1151, 664;

$[\alpha]_D^{18}$ = –12.8 (c = 0.69, $CHCl_3$); 92% *ee*;

HPLC analysis CHIRALPAK two connected AD-H columns, *n*-hexane/*iso*-propanol = 100/0, flow rate 0.5 mL/min, 220 nm UV detector, t_R (major) = 33.2 min, t_R (minor) = 38.8 min.

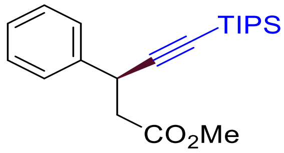

Methyl (*S*)-3-phenyl-5-(triisopropylsilyl)pent-4-ynoate (Figure 4, **5b'**). From **methyl (*E*)-4-phenylbut-3-enoate** (**4b'**) (32.4 mg, 0.20 mmol) and **(bromoethynyl)triisopropylsilane** (**2a**) (78.4 mg, 0.30 mmol), the title compound was prepared following the general procedure **B** using $NiI_2 \cdot xH_2O$ (3.8 mg, 5.0 mol%), (*S*)-**L*** (3.3 mg, 6.0 mol%), $K_3PO_4 \cdot H_2O$ (115.1 mg, 2.5 equiv), NaI (60.0 mg, 2.0 equiv), $(MeO)_3SiH$ (64 µL, 0.5 mmol, 2.5 equiv) and anhydrous $PhCF_3$ (1.0 mL). The reaction mixture was stirred for 12 h at 0 °C. The crude material was purified by flash column chromatography (petroleum ether/EtOAc = 50:1) to provide the title compound as a colorless liquid in 66% yield (45.6 mg).

^{1}H NMR (500 MHz, $CDCl_3$) δ 7.45 – 7.37 (m, 2H), 7.36 – 7.28 (m, 2H), 7.27 – 7.22 (m, 1H), 4.24 (dd, *J* = 8.9, 6.5 Hz, 1H), 3.67 (s, 3H), 2.80 (dd, *J* = 15.1, 8.9 Hz, 1H), 2.73 (dd, *J* = 15.1, 6.5 Hz, 1H), 1.26 – 0.64 (m, 21H);

^{13}C NMR (126 MHz, $CDCl_3$) δ 171.4, 140.4, 128.7, 127.5, 127.2, 108.1, 84.1, 51.9, 44.0, 35.4, 18.7, 11.3;

HRMS (ESI) calcd. for $C_{21}H_{33}O_2Si$ $[M+H]^+$ m/z 345.2244, found 345.2247;

IR (neat, cm^{-1}) 2942, 2864, 2174, 1742, 882, 664;

$[\alpha]_D^{18}$ = –12.0 (c = 0.93, $CHCl_3$); 84% *ee*;

HPLC analysis CHIRALCEL OD-H column, *n*-hexane/*iso*-propanol = 99/1, flow rate 0.5 mL/min, 220 nm UV detector, t_R (minor) = 8.1 min, t_R (major) = 20.9 min.

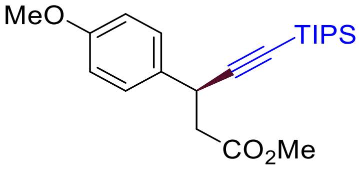

Methyl (*S*)-3-(4-methoxyphenyl)-5-(triisopropylsilyl)pent-4-ynoate (Figure 4, **5c'**). From **methyl (*E*)-4-(4-methoxyphenyl)but-3-enoate** (**4c'**) (38.4 mg, 0.20 mmol) and **(bromoethynyl)triisopropylsilane** (**2a**) (78.4 mg, 0.30 mmol), the title compound was

prepared following the general procedure **B** using $NiI_2\cdot xH_2O$ (3.8 mg, 5.0 mol%), (*S*)-**L*** (3.3 mg, 6.0 mol%), $K_3PO_4\cdot H_2O$ (115.1 mg, 2.5 equiv), NaI (60.0 mg, 2.0 equiv), $(MeO)_3SiH$ (64 μL, 0.5 mmol, 2.5 equiv) and anhydrous $PhCF_3$ (1.0 mL). The reaction mixture was stirred for 12 h at 0 °C. The crude material was purified by flash column chromatography (petroleum ether/EtOAc = 20:1) to provide the title compound as a colorless liquid in 66% yield (49.1 mg).

^{1}H NMR (500 MHz, $CDCl_3$) δ 7.35 – 7.29 (m, 2H), 6.91 – 6.81 (m, 2H), 4.18 (dd, *J* = 8.7, 6.7 Hz, 1H), 3.80 (s, 3H), 3.66 (s, 3H), 2.77 (dd, *J* = 15.1, 8.7 Hz, 1H), 2.69 (dd, *J* = 15.1, 6.7 Hz, 1H), 1.12 – 0.90 (m, 21H);

^{13}C NMR (126 MHz, $CDCl_3$) δ 171.4, 158.7, 132.5, 128.54, 114.0, 108.5, 83.8, 55.4, 51.8, 44.1, 34.6, 18.7, 11.3;

HRMS (ESI) calcd. for $C_{22}H_{34}O_3SiNa$ $[M+Na]^+$ m/z 397.2169, found 397.2169;

IR (neat, cm^{-1}) 2942, 2864, 2172, 1741, 1247, 665;

$[\alpha]_D^{18}$ = –5.3 (c = 0.30, $CHCl_3$); 86% *ee*;

HPLC analysis CHIRALCEL OD-H column, *n*-hexane/*iso*-propanol = 99/1, flow rate 0.5 mL/min, 220 nm UV detector, t_R (minor) = 9.5 min, t_R (major) = 14.2 min.

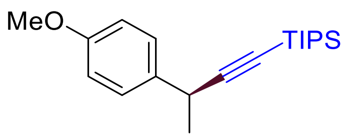

(*S*)-Triisopropyl(3-(4-methoxyphenyl)but-1-yn-1-yl)silane (Figure 4, **5d'**). From **1-methoxy-4-vinylbenzene** (**4d'**) (26.8 mg, 0.20 mmol) and **(bromoethynyl)triisopropylsilane** (**2a**) (78.4 mg, 0.30 mmol), the title compound was prepared following the general procedure **B** using $NiI_2\cdot xH_2O$ (3.8 mg, 5.0 mol%), (*S*)-**L*** (3.3 mg, 6.0 mol%), $K_3PO_4\cdot H_2O$ (115.1 mg, 2.5 equiv), NaI (60.0 mg, 2.0 equiv), $(MeO)_3SiH$ (64 μL, 0.5 mmol, 2.5 equiv) and anhydrous $PhCF_3$ (1.0 mL). The reaction mixture was stirred for 12 h at 0 °C. The crude material was purified by flash column chromatography (petroleum ether) to provide the title compound as a colorless liquid in 87% yield (55.0 mg).

^{1}H NMR (500 MHz, $CDCl_3$) δ 7.38 – 7.31 (m, 2H), 6.89 – 6.83 (m, 2H), 3.84 – 3.74 (m, 4H), 1.49 (d, *J* = 7.1 Hz, 3H), 1.13 – 1.05 (m, 21H);

^{13}C NMR (126 MHz, $CDCl_3$) δ 158.3, 135.6, 127.9, 113.8, 111.7, 82.1, 55.3, 32.2, 25.3, 18.8, 11.4;

HRMS (ESI) calcd. for $C_{20}H_{33}OSi$ $[M+H]^+$ m/z 317.2295, found 317.2294;

IR (neat, cm^{-1}) 2940, 2864, 2164, 1510, 1243, 665;

$[\alpha]_D^{18}$ = –4.0 (c = 1.15 $CHCl_3$); 92% *ee*;

HPLC analysis CHIRALCEL two connected OD-H columns, *n*-hexane/*iso*-propanol = 100/0, flow rate 0.5 mL/min, 220 nm UV detector, t_R (major) = 24.9 min, t_R (minor) = 26.5 min.

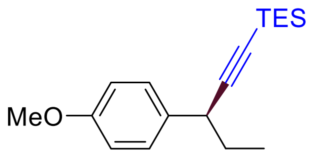

(*S*)-Triethyl(3-(4-methoxyphenyl)pent-1-yn-1-yl)silane (Figure 4, **5e'**). From **(*E*)-1-methoxy-4-(prop-1-en-1-yl)benzene** (**4e**) (29.6 mg, 0.20 mmol) and **(bromoethynyl)triethylsilane** (**2e**) (65.8 mg, 0.30 mmol), the title compound was prepared following the general procedure **B** using $NiI_2 \cdot xH_2O$ (3.8 mg, 5.0 mol%), (*S*)-**L*** (3.3 mg, 6.0 mol%), $K_3PO_4 \cdot H_2O$ (115.1 mg, 2.5 equiv), NaI (60.0 mg, 2.0 equiv), $(MeO)_3SiH$ (64 μL, 0.5 mmol, 2.5 equiv) and anhydrous $PhCF_3$ (1.0 mL). The reaction mixture was stirred for 12 h at 0 °C. The crude material was purified by flash column chromatography (petroleum ether) to provide the title compound as a colorless liquid in 40 % yield (23.1 mg).

^{1}H NMR (500 MHz, $CDCl_3$) δ 7.31 – 7.27 (m, 2H), 6.88 – 6.83 (m, 2H), 3.80 (s, 3H), 3.59 (dd, *J* = 8.1, 5.7 Hz, 1H), 1.83 – 1.65 (m, 2H), 1.08 – 0.94 (m, 12H), 0.67 – 0.56 (m, 6H);

^{13}C NMR (126 MHz, $CDCl_3$) δ 158.3, 134.1, 128.5, 113.8, 109.8, 84.3, 55.3, 39.6, 32.1, 11.7, 7.6, 4.7;

HRMS (ESI) calcd. for $C_{18}H_{28}OSi$ $[M+H]^+$ m/z 289.1982, found 289.1976;

IR (neat, cm^{-1}) 2954, 2873, 2167, 1510, 1245, 722;

$[\alpha]_D^{20}$ = –13.6 (c = 0.88 $CHCl_3$); 92% *ee*;

HPLC analysis CHIRALCEL two connected OD-H columns, *n*-hexane/*iso*-propanol = 100/0, flow rate 0.5 mL/min, 220 nm UV detector, t_R (major) = 23.6 min, t_R (minor) = 26.8 min.

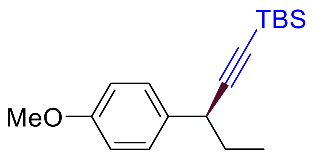

(*S*)-*tert*-Butyl(3-(4-methoxyphenyl)pent-1-yn-1-yl)dimethylsilane (Figure 4, **5f'**). From **(*E*)-1-methoxy-4-(prop-1-en-1-yl)benzene** (**4e**) (29.6 mg, 0.20 mmol) and **(bromoethynyl)(*tert*-butyl)dimethylsilane** (**2f**) (65.8 mg, 0.30 mmol), the title compound was prepared following the general procedure **B** using $NiI_2{\cdot}xH_2O$ (3.8 mg, 5.0 mol%), (*S*)-**L*** (3.3 mg, 6.0 mol%), $K_3PO_4{\cdot}H_2O$ (115.1 mg, 2.5 equiv), NaI (60.0 mg, 2.0 equiv), $(MeO)_3SiH$ (64 μL, 0.5 mmol, 2.5 equiv) and anhydrous $PhCF_3$ (1.0 mL). The reaction mixture was stirred for 12 h at 0 °C. The crude material was purified by flash column chromatography (petroleum ether) to provide the title compound as a colorless liquid in 50 % yield (28.8 mg).

^{1}H NMR (500 MHz, $CDCl_3$) δ 7.30 – 7.25 (m, 2H), 6.91 – 6.82 (m, 2H), 3.80 (s, 3H), 3.58 (dd, *J* = 8.0, 5.8 Hz, 1H), 1.82 – 1.62 (m, 2H), 1.01 – 0.95 (m, 12H), 0.13 (s, 6H);

^{13}C NMR (126 MHz, $CDCl_3$) δ 158.3, 134.0, 128.5, 113.8, 109.2, 85.3, 55.3, 39.6, 32.0, 26.2, 16.7, 11.7, –4.2;

HRMS (ESI) calcd. for $C_{18}H_{28}OSi$ $[M+H]^+$ m/z 289.1982, found 289.1975;

IR (neat, cm^{-1}) 2928, 2856, 2168, 1510, 1245, 824;

$[\alpha]_D^{20}$ = –10.7 (c = 1.12 $CHCl_3$); 94% *ee*;

HPLC analysis CHIRALCEL two connected OD-H columns, *n*-hexane/*iso*-propanol = 100/0, flow rate 0.5 mL/min, 220 nm UV detector, t_R (major) = 23.1 min, t_R (minor) = 26.8 min.

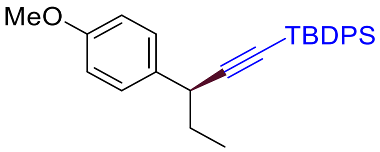

(*S*)-*tert*-Butyl(3-(4-methoxyphenyl)pent-1-yn-1-yl)diphenylsilane (Table 3, **5g'**). From **(*E*)-1-methoxy-4-(prop-1-en-1-yl)benzene** (**4e**) (29.6 mg, 0.20 mmol) and **(bromoethynyl)(*tert*-butyl)diphenylsilane (2b)** (103.0 mg, 0.30 mmol), the title compound was prepared following the general procedure **B** using $NiI_2·xH_2O$ (3.8 mg, 5.0 mol%), (*S*)-**L*** (3.3 mg, 6.0 mol%), $K_3PO_4·H_2O$ (115.1 mg, 2.5 equiv), NaI (60.0 mg, 2.0 equiv), $(MeO)_3SiH$ (64 μL, 0.5 mmol, 2.5 equiv) and anhydrous $PhCF_3$ (1.0 mL). The reaction mixture was stirred for 12 h at 0 °C. The crude material was purified by flash column chromatography (petroleum ether/EtOAc = 100:1) to provide the title compound as a colorless liquid in 42% yield (34.6 mg).

^{1}H NMR (500 MHz, $CDCl_3$) δ 7.85 – 7.80 (m, 4H), 7.42 – 7.34 (m, 8H), 6.93 – 6.82 (m, 2H), 3.82 (s, 3H), 3.76 (dd, *J* = 7.7, 6.1 Hz, 1H), 1.92 – 1.78 (m, 2H), 1.12 – 0.99 (m, 12H);

^{13}C NMR (126 MHz, $CDCl_3$) δ 158.4, 135.7, 134.0, 133.9, 133.7, 129.4, 128.6, 127.7, 113.9, 113.1, 82.3, 55.4, 39.9, 32.1, 27.2, 18.7, 11.9;

HRMS (ESI) calcd. for $C_{28}H_{32}OSiNa$ $[M+Na]^+$ m/z 435.2115, found 435.2115;

IR (neat, cm^{-1}) 2929, 2856, 2169, 1510, 1246, 698;

$[\alpha]_D^{18}$ = –12.9 (c = 0.65 $CHCl_3$); 96% *ee*;

HPLC analysis CHIRALCEL OD-H column, *n*-hexane/*iso*-propanol = 100/0, flow rate 0.8 mL/min, 220 nm UV detector, t_R (minor) = 26.9 min, t_R (major) = 28.6 min.

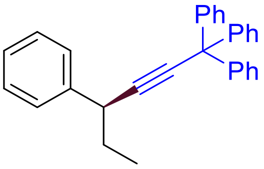

(*S*)-Hex-2-yne-1,1,1,4-tetrayltetrabenzene (Table 3, **5h'**). From **(*E*)-prop-1-en-1-ylbenzene** (**4h'**) (23.6 mg, 0.20 mmol) and **(3-bromoprop-2-yne-1,1,1-triyl)tribenzene (2c)** (104.2 mg, 0.30 mmol), the title compound was prepared

following the general procedure **B** using $NiI_2 \cdot xH_2O$ (3.8 mg, 5.0 mol%), (*S*)-**L*** (3.3 mg, 6.0 mol%), $K_3PO_4 \cdot H_2O$ (115.1 mg, 2.5 equiv), NaI (60.0 mg, 2.0 equiv), $(MeO)_3SiH$ (64 μL, 0.5 mmol, 2.5 equiv) and anhydrous $PhCF_3$ (1.0 mL). The reaction mixture was stirred for 12 h at 0 °C. The crude material was purified by flash column chromatography (petroleum ether/EtOAc = 100:1) to provide the title compound as a colorless liquid in 50% yield (38.5 mg).

^{1}H NMR (500 MHz, $CDCl_3$) δ 7.41 – 7.37 (m, 2H), 7.34 – 7.20 (m, 18H), 3.78 (dd, *J* = 7.9, 6.0 Hz, 1H), 1.98 – 1.71 (m, 2H), 1.03 (t, *J* = 7.4 Hz, 3H);

^{13}C NMR (126 MHz, $CDCl_3$) δ 145.9, 142.4, 129.3, 128.4, 128.0, 127.7, 126.7, 126.6, 89.3, 86.8, 55.8, 39.8, 32.1, 12.0;

HRMS (ESI) calcd. for $C_{30}H_{27}$ $[M+H]^+$ m/z 387.2107, found 387.2102;

IR (neat, cm^{-1}) 3285, 2921, 1489, 1446, 753, 697;

$[\alpha]_D^{18}$ = –7.5 (c = 1.02 $CHCl_3$); 92% *ee*;

HPLC analysis CHIRALCEL two connected OD-H columns, *n*-hexane/*iso*-propanol = 100/0, flow rate 0.5 mL/min, 220 nm UV detector, t_R (minor) = 45.5 min, t_R (major) = 48.2 min.

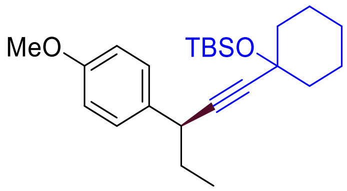

(*S*)-*tert*-Butyl((1-(3-(4-methoxyphenyl)pent-1-yn-1-yl)cyclohexyl)oxy)dimethylsilane (Table 3, **5i'**). From **(*E*)-1-methoxy-4-(prop-1-en-1-yl)benzene** (**4e**) (29.6 mg, 0.20 mmol) and **((1-(bromoethynyl)cyclohexyl)oxy)(*tert*-butyl)dimethylsilane** (**2d**) (95.2 mg, 0.30 mmol), the title compound was prepared following the general procedure **B** using $NiI_2 \cdot xH_2O$ (3.8 mg, 5.0 mol%), (*S*)-**L*** (3.3 mg, 6.0 mol%), $K_3PO_4 \cdot H_2O$ (115.1 mg, 2.5 equiv), NaI (60.0 mg, 2.0 equiv), $(MeO)_3SiH$ (64 μL, 0.5 mmol, 2.5 equiv) and anhydrous $PhCF_3$ (1.0 mL). The reaction mixture was stirred for 12 h at 0 °C. The crude material was purified by flash column chromatography (petroleum ether/EtOAc =

100:1) to provide the title compound as a colorless liquid in 66 % yield (50.9 mg).

^{1}H NMR (500 MHz, $CDCl_3$) δ 7.28 – 7.22 (m, 2H), 6.88 – 6.79 (m, 2H), 3.80 (s, 3H), 3.55 (t, *J* = 6.9 Hz, 1H), 1.85 – 1.69 (m, 4H), 1.67 – 1.47 (m, 6H), 1.47 – 1.39 (m, 1H), 1.33 – 1.27 (m, 1H), 0.97 (t, *J* = 7.4 Hz, 3H), 0.87 (s, 9H), 0.13 (s, 3H), 0.12 (s, 3H);

^{13}C NMR (126 MHz, $CDCl_3$) δ 158.3, 134.3, 128.6, 113.7, 87.4, 86.5, 69.5, 55.4, 41.7, 41.6, 38.6, 31.6, 25.9, 25.5, 23.2, 23.1, 18.2, 11.9, –2.6, –2.7;

HRMS (ESI) calcd. for $C_{24}H_{39}O_2Si$ $[M+H]^+$ m/z 387.2714, found 387.2716;

IR (neat, cm^{-1}) 2928, 2854, 1510, 1249, 836, 776;

$[\alpha]_D^{18}$ = –12.8 (c = 0.72 $CHCl_3$); 86% *ee*;

HPLC analysis CHIRALCEL two connected OD-H columns, *n*-hexane/*iso*-propanol = 100/0, flow rate 0.5 mL/min, 220 nm UV detector, t_R (major) = 21.9 min, t_R (minor) = 24.6 min.

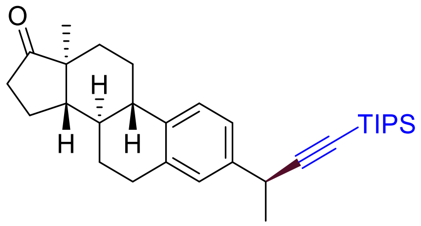

(8*R*,9*S*,13*S*,14*S*)-13-Methyl-3-((*S*)-4-(triisopropylsilyl)but-3-yn-2-yl)-6,7,8,9,11,12,13,14,15,16-decahydro-17*H*-cyclopenta[a]phenanthren-17-one (Table 3, **5j'**). From **(8*R*,9*S*,13*S*,14*S*)-13-methyl-3-vinyl-6,7,8,9,11,12,13,14,15,16-decahydro-17*H*-cyclopenta[*a*]phenanthren-17-one** (**4j'**) (56.1 mg, 0.20 mmol) and **(bromoethynyl)triisopropylsilane** (**2a**) (78.4 mg, 0.30 mmol), the title compound was prepared following the general procedure **B** using $NiI_2·xH_2O$ (3.8 mg, 5.0 mol%), (*S*)-**L*** (3.3 mg, 6.0 mol%), $K_3PO_4·H_2O$ (115.1 mg, 2.5 equiv), NaI (60.0 mg, 2.0 equiv), $(MeO)_3SiH$ (64 μL, 0.5 mmol, 2.5 equiv) and anhydrous $PhCF_3$ (1.0 mL). The reaction mixture was stirred for 12 h at 0 °C. The crude material was purified by flash column chromatography (petroleum ether/EtOAc = 20:1) to provide the title compound as a colorless liquid in 74% yield (68.1 mg).

^{1}H NMR (500 MHz, $CDCl_3$) δ 7.28 – 7.24 (m, 1H), 7.22 – 7.18 (m, 2H), 3.77 (q, *J* = 7.1 Hz, 1H), 2.98 – 2.86 (m, 2H), 2.51 (dd, *J* = 19.0, 8.7 Hz, 1H), 2.47 – 2.39 (m, 1H), 2.34 – 2.26 (m, 1H), 2.20 – 2.11 (m, 1H), 2.10 – 2.00 (m, 2H), 2.00 – 1.93 (m, 1H), 1.67 – 1.58 (m, 2H), 1.58 – 1.42 (m, 7H), 1.18 – 1.06 (m, 21H), 0.92 (s, 3H);

^{13}C NMR (126 MHz, $CDCl_3$) δ 221.0, 140.8, 138.0, 136.5, 127.7, 125.5, 124.4, 111.4, 82.2, 50.6, 48.1, 44.4, 38.3, 35.9, 32.5, 31.7, 29.5, 26.6, 25.8, 25.2, 21.7, 18.8, 13.9, 11.4;

HRMS (ESI) calcd. for $C_{31}H_{46}OSiNa$ $[M+Na]^+$ m/z 485.3210, found 485.3207;

IR (neat, cm^{-1}) 2928, 2863, 2165, 1738, 735, 675;

$[\alpha]_D^{18}$ = +93.4 (c = 0.91 $CHCl_3$); 97:3 *dr*;

HPLC analysis CHIRALCEL two connected OD-H columns, *n*-hexane/*iso*-propanol = 99.5/0.5, flow rate 0.5 mL/min, 220 nm UV detector, t_R (minor) = 27.6 min, t_R (major) = 29.1 min.

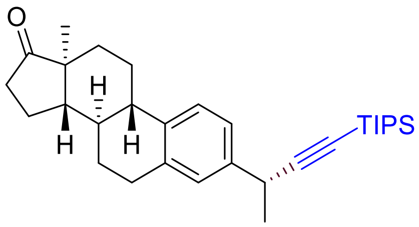

(8*R*,9*S*,13*S*,14*S*)-13-Methyl-3-((*R*)-4-(triisopropylsilyl)but-3-yn-2-yl)-6,7,8,9,11,12,13,14,15,16-decahydro-17*H*-cyclopenta[a]phenanthren-17-one (Table 3, **5k'**). From **(8*R*,9*S*,13*S*,14*S*)-13-methyl-3-vinyl-6,7,8,9,11,12,13,14,15,16-decahydro-17*H*-cyclopenta[*a*]phenanthren-17-one** (**4j'**) (56.1 mg, 0.20 mmol) and **(bromoethynyl)triisopropylsilane** (**2a**) (78.4 mg, 0.30 mmol), the title compound was prepared following the general procedure **B** using $NiI_2 \cdot xH_2O$ (3.8 mg, 5.0 mol%), (*R*)-**L*** (3.3 mg, 6.0 mol%), $K_3PO_4 \cdot H_2O$ (115.1 mg, 2.5 equiv), NaI (60.0 mg, 2.0 equiv), $(MeO)_3SiH$ (64 µL, 0.5 mmol, 2.5 equiv) and anhydrous $PhCF_3$ (1.0 mL). The reaction mixture was stirred for 12 h at 0 °C. The crude material was purified by flash column chromatography (petroleum ether/EtOAc = 20:1) to provide the title compound as a colorless liquid in 81% yield (74.8 mg).

^{1}H NMR (500 MHz, $CDCl_3$) δ 7.28 – 7.25 (m, 1H), 7.22 – 7.18 (m, 2H), 3.77 (q, *J* = 7.1 Hz, 1H), 2.92 (dd, *J* = 9.1, 4.2 Hz, 2H), 2.51 (dd, *J* = 19.0, 8.7 Hz, 1H), 2.47 – 2.40 (m, 1H), 2.34 – 2.26 (m, 1H), 2.20 – 2.11 (m, 1H), 2.11 – 2.00 (m, 2H), 2.00 – 1.94 (m, 1H), 1.67 – 1.58 (m, 2H), 1.58 – 1.41 (m, 7H), 1.15 – 0.99 (m, 21H), 0.92 (s, 3H);

^{13}C NMR (126 MHz, $CDCl_3$) δ 221.0, 140.8, 137.9, 136.5, 127.7, 125.5, 124.4, 111.5, 82.2, 50.6, 48.1, 44.4, 38.2, 35.9, 32.5, 31.7, 29.5, 26.6, 25.8, 25.2, 21.7, 18.8, 13.9, 11.4;

HRMS (ESI) calcd. for $C_{31}H_{46}OSiNa$ $[M+Na]^+$ m/z 485.3210, found 485.3205;

IR (neat, cm^{-1}) 2926, 2863, 2165, 1740, 1009, 670;

$[\alpha]_D^{18}$ = +89.5 (c = 1.20 $CHCl_3$); 5:95 *dr*;

HPLC analysis CHIRALCEL two connected OD-H columns, *n*-hexane/*iso*-propanol = 99.5/0.5, flow rate 0.5 mL/min, 220 nm UV detector, t_R (major) = 27.6 min, t_R (minor) = 29.2 min.

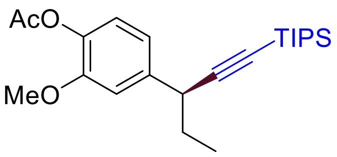

(*S*)-2-Methoxy-4-(1-(triisopropylsilyl)pent-1-yn-3-yl)phenyl acetate (Figure 5a, (*S*)-**3i**). From **4-allyl-2-methoxyphenyl acetate** (**1i**) (41.2 mg, 0.20 mmol) and **(bromoethynyl)triisopropylsilane (2a)** (78.4 mg, 0.30 mmol), the title compound was prepared following the general procedure **B** using $NiI_2 \cdot xH_2O$ (3.8 mg, 5.0 mol%), (*S*)-**L*** (3.3 mg, 6.0 mol%), $K_3PO_4 \cdot H_2O$ (115.1 mg, 2.5 equiv), NaI (60.0 mg, 2.0 equiv), $(MeO)_3SiH$ (64 μL, 0.5 mmol, 2.5 equiv) and anhydrous $PhCF_3$ (1.0 mL). The reaction mixture was stirred for 12 h at 0 °C. The crude material was purified by flash column chromatography (petroleum ether) to provide the title compound as a colorless liquid in 78 % yield (60.5 mg).

^{1}H NMR (500 MHz, $CDCl_3$) δ 7.10 (d, *J* = 1.9 Hz, 1H), 6.96 (d, *J* = 8.1 Hz, 1H), 6.89 (dd, *J* = 8.2, 1.9 Hz, 1H), 3.82 (s, 3H), 3.65 (dd, *J* = 8.5, 5.3 Hz, 1H), 2.31 (s, 3H), 1.87 – 1.77 (m, 1H), 1.76 – 1.66 (m, 1H), 1.11 – 1.06 (m, 21H), 1.04 (t, *J* = 7.4 Hz, 3H);

^{13}**C NMR** (126 MHz, $CDCl_3$) δ 169.3, 150.8, 141.0, 138.2, 122.4, 119.8, 111.8, 109.6, 83.8, 55.9, 40.4, 32.2, 20.8, 18.8, 11.8, 11.4;

HRMS (ESI) calcd. for $C_{23}H_{36}O_3Si$ $[M+Na]^+$ m/z 411.2326, found 411.2319;

IR (neat, cm^{-1}) 2940, 2864, 2167, 1766, 1508, 1195;

$[\alpha]_D^{20}$ = –20.5 (c = 0.85 $CHCl_3$); 90% *ee*;

HPLC analysis CHIRALCEL two connected OD-H columns, *n*-hexane/*iso*-propanol = 99/1, flow rate 0.5 mL/min, 220 nm UV detector, t_R (minor) = 17.7 min, t_R (major) = 20.6 min.

Gram-Scale Experiment

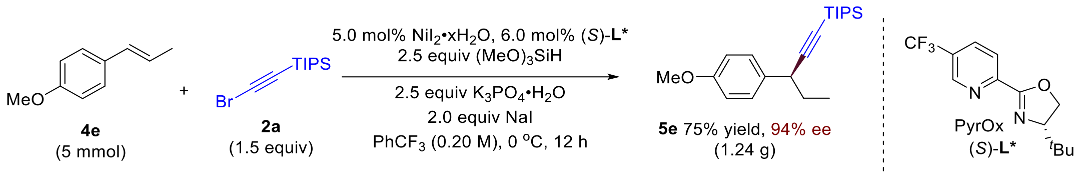

In a nitrogen-filled glove box, to an oven-dried 50 mL round bottom flask equipped with a magnetic stir bar were added $NiI_2 \cdot xH_2O$ (95.0 mg, 5.0 mol%), (*S*)-**L*** (82.5 mg, 6.0 mol%), $K_3PO_4 \cdot H_2O$ (2.88 g, 2.5 equiv), NaI (1.50 g, 2.0 equiv) and anhydrous $PhCF_3$ (25.0 mL, 0.20M). The mixture was stirred for 20 min at room temperature before the addition of $(MeO)_3SiH$ (1.60 mL, 12.5 mmol, 2.5 equiv). Stirring was continued for an additional 5 min at room temperature before (*E*)-1-methoxy-4-(prop-1-en-1-yl)benzene **4e** (5.0 mmol, 1.0 equiv) and (bromoethynyl)triisopropylsilane **2a** (7.5 mmol, 1.5 equiv) were added to the resulting mixture in this order. The flask was sealed with a rubber stopper, removed from the glove box and equipped with a N_2 balloon, and stirred at 0 °C for up to 12 h (the mixture was stirred at 800 rpm). After the reaction was complete, the reaction was quenched upon the addition of H_2O, and the mixture was extracted with EtOAc. The organic layer was concentrated. The crude material was purified by flash column chromatography (petroleum ether) to provide **5e** as a colorless liquid in 75% yield (1.24 g). The ee (94%) was determined via HPLC analysis (General procedure **B**).

Synthetic Transformations

Removing the TIPS Group in 5e

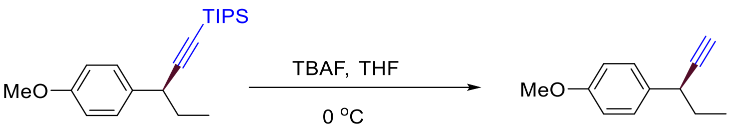

5e, 94% ee **6** 78%, 94% ee

A 10-mL Schlenk tube was charged with the product **5e** (66.0 mg, 0.2 mmol) and THF (2 mL), and filled with nitrogen. To the solution cooled to 0 °C was added a solution of tetrabutylammonium fluoride (1.0 mol/L solution in THF; 2.0 mL, 2.0 mmol) dropwise via syringe over 2 min. After 4 h, the reaction was quenched with water (2 mL). The layers were separated, and the aqueous layer was extracted with diethyl ether (3 x 20 mL). The combined organic layers were washed with brine (50 mL), dried over Na_2SO_4, filtered, and concentrated. Then the mixture was purified by silica gel column chromatography (eluted with petroleum ether) to afford the desilylation product **6** as colorless oil (27.2 mg, 78% yield, 94% ee).

^{1}H NMR (500 MHz, $CDCl_3$) δ 7.36 – 7.09 (m, 2H), 7.00 – 6.71 (m, 2H), 3.80 (s, 3H), 3.59 – 3.39 (m, 1H), 2.27 (d, J = 2.5 Hz, 1H), 1.83 – 1.72 (m, 2H), 0.99 (t, J = 7.4 Hz, 3H);

^{13}C NMR (126 MHz, $CDCl_3$) δ 158.5, 133.6, 128.5, 113.9, 86.3, 70.8, 55.4, 38.4, 31.5, 11.7;

HRMS (ESI) calcd. for $C_{12}H_{14}ONa$ $[M+Na]^+$ m/z 197.0937, found 197.0934;

IR (neat, cm^{-1}) 3292, 2940, 1509, 1243, 1034, 632;

$[\alpha]_D^{18}$ = –21.0 (c = 0.59 $CHCl_3$); 94% *ee*;

HPLC analysis CHIRALCEL OD-H column, *n*-hexane/*iso*-propanol = 100/0, flow rate 0.5 mL/min, 220 nm UV detector, t_R (major) = 44.2 min, t_R (minor) = 51.9 min.

Click Reaction

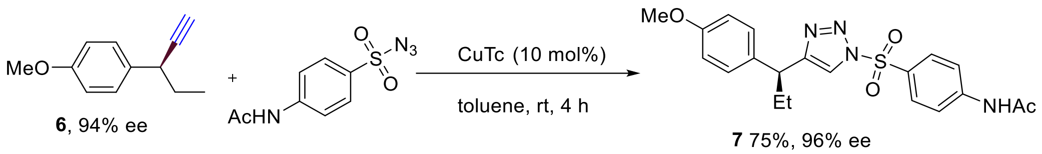

To a solution of CuTc (3.8 mg, 0.02 mmol) and **6** (34.8 mg, 0.2 mmol) in toluene (2 mL), 4-acetamidobenzenesulfonyl azide (48.0 mg, 0.2 mmol) was added, and the mixture was stirred at room temperature for 4 h. After the reaction was completed, the organic solvent was removed under vacuum, and the residue was purified by column chromatography (DCM/EtOAc = 5:1) to give the product **7** (62.1 mg, 75% yield, 96% ee) as a white solid.

^{1}H NMR (500 MHz, DMSO-d_6) δ 10.57 (s, 1H), 8.65 (s, 1H), 8.05 (d, J = 9.0 Hz, 2H), 7.86 (d, J = 9.0 Hz, 2H), 7.17 (d, J = 8.8 Hz, 2H), 6.84 (d, J = 8.7 Hz, 2H), 3.92 (t, J = 7.8 Hz, 1H), 3.70 (s, 3H), 2.09 (s, 3H), 2.07 – 1.99 (m, 1H), 1.93 – 1.83 (m, 1H), 0.74 (t, J = 7.3 Hz, 3H);

^{13}C NMR (126 MHz, DMSO-d_6) δ 169.5, 157.8, 151.5, 146.0, 134.5, 129.9, 128.6, 127.8, 121.5, 119.1, 113.8, 54.9, 42.9, 28.2, 24.2, 12.1;

HRMS (ESI) calcd. for $C_{20}H_{22}N_4O_4SNa$ [M+Na]$^+$ m/z 437.1254, found 437.1256;

IR (neat, cm^{-1}) 3330, 3107, 2961, 1394, 1176, 577;

m.p. 135.6 – 136.7 ^{o}C;

$[\alpha]_D^{18}$ = +21.8 (c = 0.57 Acetone); 96% *ee*;

HPLC analysis CHIRALPAK IC column, *n*-hexane/*iso*-propanol = 80/20, flow rate 0.8 mL/min, 254 nm UV detector, t_R (major) = 24.7 min, t_R (minor) = 30.3 min.

Conversion of the Terminal Alkyne into Aldehyde

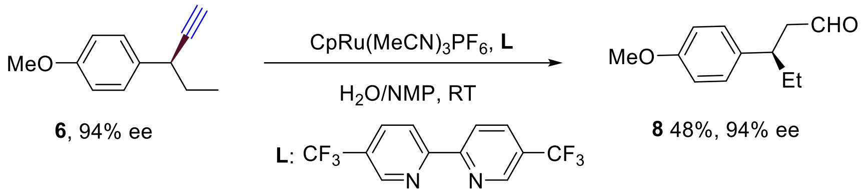

To a Schlenk tube containing [$CpRu(MeCN)_3$]PF_6 (1.7 mg, 0.0040 mmol, 2.0 mol%) and 5,5'- bis(trifluoromethyl)-2,2'-bipyridine **L** (1.2 mg, 0.0040 mmol, 2.0 mol%) was added **6** (0.20 mmol) in a mixture of NMP (0.8 mL) and water (0.2 mL) under nitrogen atmosphere and the reaction mixture was stirred at 25 °C overnight. After completion of reaction (monitored by TLC), the reaction mixture was diluted by EtOAc, washed by brine, dried over Na_2SO_4, filtered, and concentrated under reduced pressure. The residue was purified by column chromatography on silica gel (petroleum ether/EtOAc = 20/1) to afford **8** as a colorless oil (18.4 mg, 48%, 94% ee).

^{1}H NMR (500 MHz, $CDCl_3$) δ 9.65 (t, J = 2.2 Hz, 1H), 7.12 – 7.07 (m, 2H), 6.87 – 6.81 (m, 2H), 3.79 (s, 3H), 3.08 – 2.99 (m, 1H), 2.72 – 2.63 (m, 2H), 1.73 – 1.64 (m, 1H), 1.64 – 1.52 (m, 1H), 0.80 (t, J = 7.4 Hz, 3H);

^{13}C NMR (126 MHz, $CDCl_3$) δ 202.4, 158.3, 135.7, 128.5, 114.0, 55.3, 50.5, 41.1, 29.8, 12.0;

HRMS (ESI) calcd. for $C_{12}H_{17}O_2$ $[M+H]^+$ m/z 193.1223, found 193.1224;

IR (neat, cm^{-1}) 2960, 2927, 1708, 1512, 1249, 829;

$[\alpha]_D^{18}$ = –14.8 (c = 0.31 $CHCl_3$); 94% *ee*;

HPLC analysis CHIRALPAK IC column, *n*-hexane/*iso*-propanol = 99/1, flow rate 0.5 mL/min, 220 nm UV detector, t_R (minor) = 26.1 min, t_R (major) = 27.6 min.

Semi-Reduction of the Triple Bond by DIBAL-H

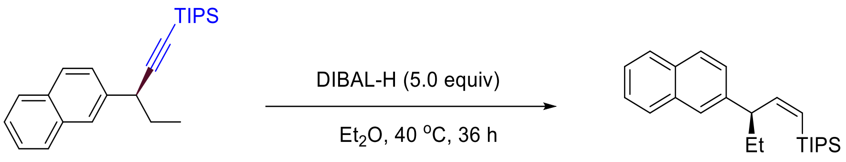

5a, 94% ee　　　　**9**, 98%, 94% ee

To a solution of **5a** (70.0 mg, 0.2 mmol) in Et_2O (2 mL), DIBAL-H (1.0 mL, 1.0 M solution in hexanes) was added, then the mixture was stirred at 40 °C for 36 h. After the reaction was completed, aqueous solution of NaOH was added to quench extra DIABL-H, and the solution was extracted by Et_2O. The combined organic layer was dried over $MgSO_4$, and filtered. After the removal of the solvent, the residue was purified by column chromatography (petroleum ether) to give product **9** (69.0 mg, 98% yield, 94% ee) as colorless oil.

^{1}H NMR (500 MHz, $CDCl_3$) δ 7.85 – 7.78 (m, 3H), 7.67 – 7.63 (m, 1H), 7.52 – 7.38 (m, 3H), 6.84 (dd, *J* = 14.4, 10.7 Hz, 1H), 5.50 (d, *J* = 14.4 Hz, 1H), 3.60 – 3.32 (m, 1H), 1.91 – 1.76 (m, 2H), 1.28 – 1.17 (m, 3H), 1.12 (d, *J* = 7.4 Hz, 9H), 1.04 (d, *J* = 7.4 Hz, 9H), 0.86 (t, *J* = 7.4 Hz, 3H);

^{13}C NMR (126 MHz, $CDCl_3$) δ 152.9, 142.2, 133.7, 132.2, 128.0, 127.8, 127.7, 126.2, 126.1, 125.9, 125.2, 123.2, 51.8, 31.1, 19.1, 19.0, 12.5, 11.9;

HRMS (ESI) calcd. for $C_{24}H_{36}SiNa$ $[M+Na]^+$ m/z 375.2478, found 375.2480;

IR (neat, cm^{-1}) 2939, 2863, 1460, 743, 664, 474;

$[\alpha]_D^{18}$ = –147.1 (c = 1.07 $CHCl_3$); 94% *ee*;

HPLC analysis CHIRALCEL two connected OD-H columns, *n*-hexane/*iso*-propanol = 100/0, flow rate 0.5 mL/min, 220 nm UV detector, t_R (major) = 23.5 min, t_R (minor) = 26.8 min.

Oxidative Cleavage of the Triple Bond to Carboxylic Acid

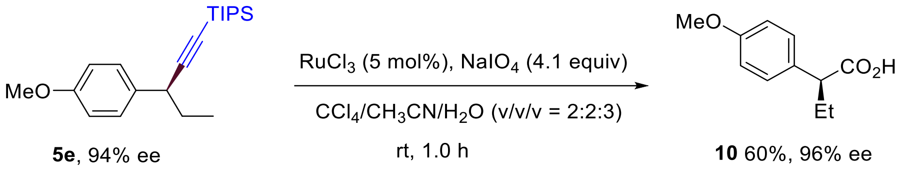

To a solution of **5e** (66.0 mg, 0.2 mmol) in a mixed solvent of CCl_4, CH_3CN and H_2O (1.7 mL, v/v/v 2:2:3), $RuCl_3$ (2.0 mg, 5 mol%) and sodium periodate (175.4 mg, 4.1 equiv) were added, then the mixture was stirred vigorously at room temperature for 1 h. After reaction completion, the mixture was extracted with dichloromethane (3 x 5 mL). The combined organic phase was dried over Na_2SO_4. The organic solvent was removed under vacuum, and the residue was purified via a preparative plate (petroleum ether /EtOAc) to give acid **10** (23.2 mg, 60%, 96% ee) as a colorless oil.

^{1}H NMR (500 MHz, $CDCl_3$) δ 7.25 – 7.21 (m, 2H), 6.87 – 6.84 (m, 2H), 3.79 (s, 3H), 3.41 (t, *J* = 7.7 Hz, 1H), 2.17 – 2.00 (m, 1H), 1.85 – 1.69 (m, 1H), 0.90 (t, *J* = 7.4 Hz, 3H);

^{13}C NMR (126 MHz, $CDCl_3$) δ 179.7, 159.0, 130.6, 129.2, 114.1, 55.4, 52.4, 26.4, 12.2;

HRMS (ESI) calcd. for $C_{11}H_{14}O_3Na$ $[M+Na]^+$ m/z 217.0835, found 217.0834;

IR (neat, cm^{-1}) 2964, 2930, 1703, 1511, 1250, 1178;

$[\alpha]_D^{18}$ = +50.0 (c = 0.14 $CHCl_3$); 96% *ee*;

HPLC analysis CHIRALPAK AD-H column, *n*-hexane/*iso*-propanol/TFA = 90/10/0.1, flow rate 0.5 mL/min, 220 nm UV detector, t_R (minor) = 17.5 min, t_R (major) = 20.3 min.

Conditions Optimization

Supplementary Table 1. The conditions optimization for enantioselective NiH-catalyzed reductive hydroalkynylation.

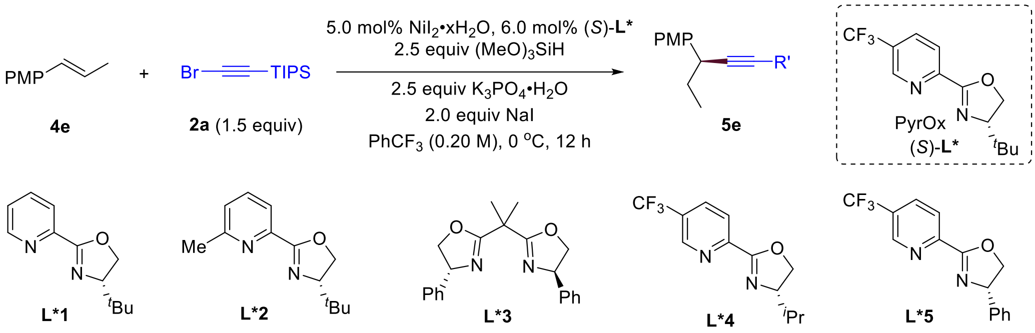

Entry	Variation from the standard conditions	Yield (%)[a]	ee (%)[b]
1	None	86	94
2	w/o $NiI_2 \cdot xH_2O$	NR	
3	w/o $K_3PO_4 \cdot H_2O$	NR	
4	w/o NaI	29	94
5	**L*1** instead of **L***	44	82
6	**L*2** instead of **L***	73	10
7	**L*3** instead of **L***	40	62
8	**L*4** instead of **L***	12	88
9	**L*5** instead of **L***	16	86
10	PMHS instead of $(MeO)_3SiH$	<5	nd
11	DMMS instead of $(MeO)_3SiH$	42	94
12	DME instead of $PhCF_3$	13	84
13	DCE instead of $PhCF_3$	82	92
14	$Ni(NO_3)_2 \cdot 6H_2O$ instead of $NiI_2 \cdot xH_2O$	79	94
15	NiI_2 instead of $NiI_2 \cdot xH_2O$	<5	nd
16	Na_2CO_3 instead of $K_3PO_4 \cdot H_2O$	6	92
17	MeOLi instead of $K_3PO_4 \cdot H_2O$	<5	nd
18	KI instead of NaI	70	94
19	TBAI instead of NaI	41	94
20	25 °C	57	90

[a]Yield were determined by GC using dodecane as internal standard; [b]The enantiomeric excesses (ee) were determined by chiral HPLC analysis.

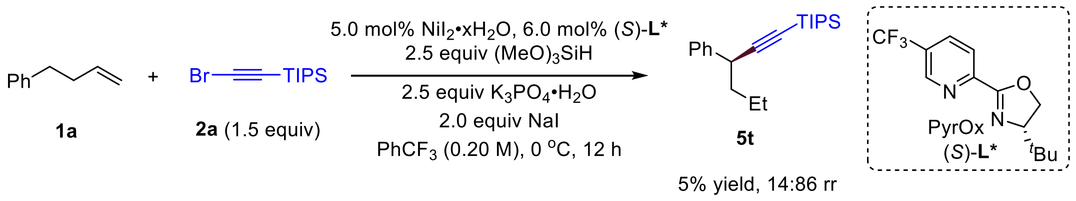

Unsuccessful Substrates

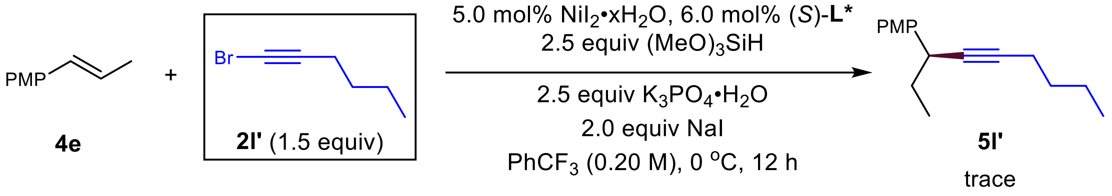

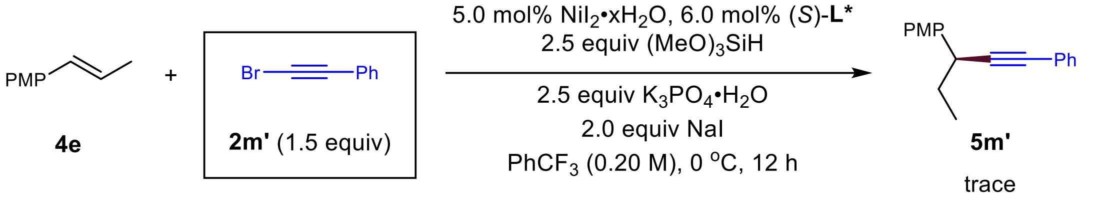

Preparation of Substrates

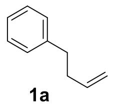

1a

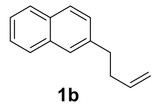

1b

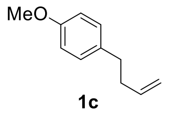

1c

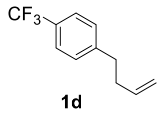

1d

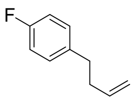

1e

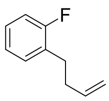

1f

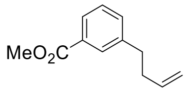

1g

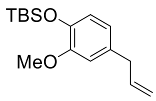

1h

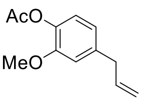

1i

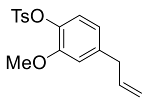

1j

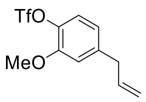

1k

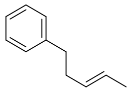

1o

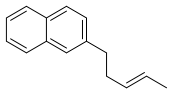

1p

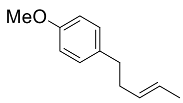

1q

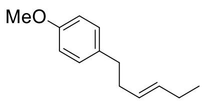

1r

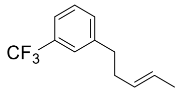

1s

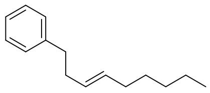

1t

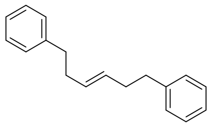

1u

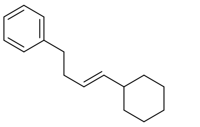

1v

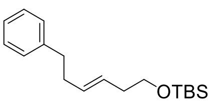

1w

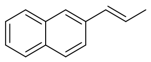

1x

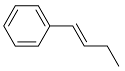

1y

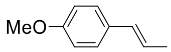

1z

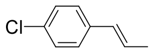

1a'

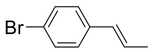

1b'

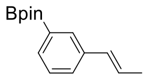

1c'

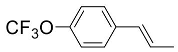

1d'

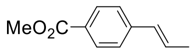

1e'

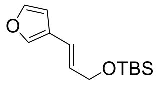

1f'

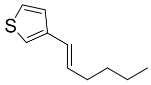

1g'

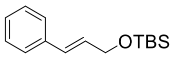

1h'

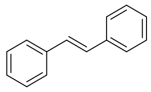

1i'

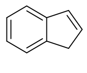

1j'

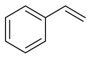

1k'

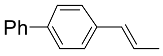

4b

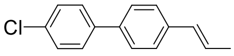

4c

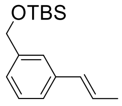

4d

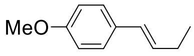

4f

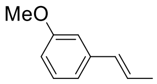

4g

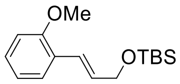

4h

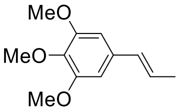

4i

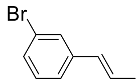

4k

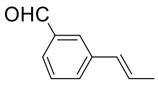

4l

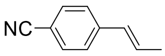

4m

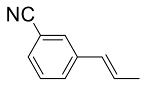

4n

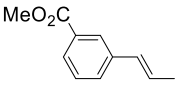

4p

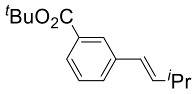

4q

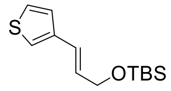

4r

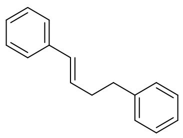

4u

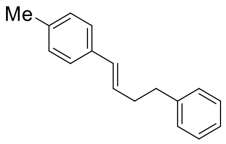

4v

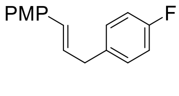

4w

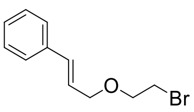

4y

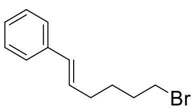

4z

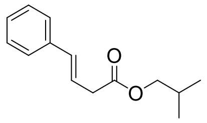

4a'

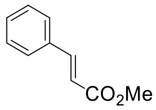

4b'

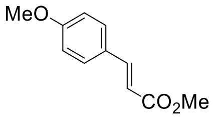

4c'

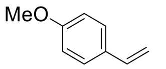

4d'

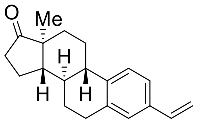

4j'

Compounds **1a**, **1z**, **1i'**, **1j'**, **1k'**, **4b'**, and **4d'** are commercially available. Compounds **1b**[7], **1c**[8], **1d**[8], **1e**[8], **1f**[9], **1n**[9], **1g**[10], **1j**[10], **1u**[10], **4u**[10], **4a'**[10], **1h**[11], **1i**[12], **1k**[13], **1p**[14], **1q**[14], **1r**[15], **1s**[16], **4j'**[16], **1t**[17], **1v**[17], **1w**[18], **1x**[19], **1a'**[19], **1b'**[19], **4b**[19], **4f**[19], **4g**[19], **4i**[19], **1y**[20], **1c'**[20], **1e'**[20], **1g'**[20], **4d**[20], **4m**[20], **1h'**[21], **4k**[22], **4l**[23], **4p**[24], **4w**[25], **4y**[26], **4z**[27], **4v**[28], **4c'**[29], and **4n**[30] were prepared according to the previously reported procedures.

General procedure (C) for the synthesis of olefins 1d', 4c, and 4q

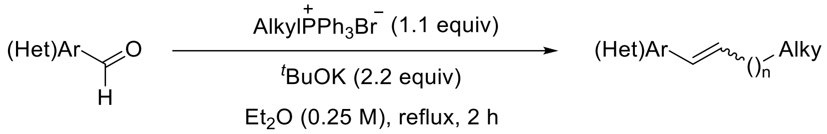

A flame dried 250 round bottom flask was charged with alkyl triphenyl phosphonium bromide (1.1 equiv), KO^{t}Bu (2.2 equiv, ***Note: hydroscopic, stored under nitrogen. Exposure to air should be less than 5 minutes***), a stir bar, and anhydrous diethyl ether (0.25 M). A reflux condense was attached and the reaction mixture was heated to reflux for over 1 h before adding a solution of aldehyde (1.0 equiv) in anhydrous diethyl ether. The reaction was monitored by TLC and upon completion of the reaction (~ 2 h), the solvent was removed in vacuo. The resulting compound was diluted with hexanes, filtered over a thick plug of silica gel and concentrated again. The reaction was purified via column chromatography to get the desired compound.

Characterization of Substrates:

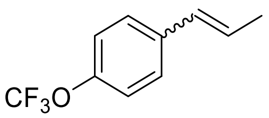

1-(Prop-1-en-1-yl)-4-(trifluoromethoxy)benzene (1d'):

Yield 78% (1.57 g, ~ 1:1.8 *E/Z*), colorless liquid;

^{1}H NMR (500 MHz, $CDCl_3$) δ 7.35 – 7.28 (m, 2H), 7.21 – 7.12 (m, 2H), 6.45 – 6.35 (m, 1H), [6.28 – 6.17 (m, 0.35H) & 5.88 – 5.79 (m, 0.65H), *due to Z/E*], 1.94 – 1.80 (m, 3H);

^{13}C NMR (126 MHz, $CDCl_3$) δ (147.9 & 147.5, *due to Z/E*), (136.7, 136.3 & *due to Z/E*), (130.0 & 129.6, *due to Z/E*), (128.5 & 127.6, *due to Z/E*), (126.9 & 126.8, *due to Z/E*), (121.0 & 120.6, *due to Z/E*), 120.5 (q, J = 257.4), (18.4 & 14.4, *due to Z/E*);

^{19}F NMR (471 MHz, CDCl3) δ –57.8;

HRMS (ESI) calcd. for $C_{10}H_{10}F_3O$ [M+H]$^+$ m/z 203.0678, found 203.0676;

IR (neat, cm^{-1}) 2921, 1507, 1253, 1155, 814, 526.

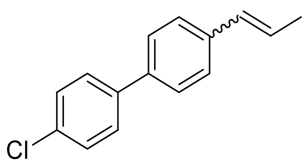

4-Chloro-4'-(prop-1-en-1-yl)-1,1'-biphenyl (4c):

Yield 65% (~ 1:3 *E/Z*), white solid;

^{1}H NMR (500 MHz, $CDCl_3$) δ 7.58 – 7.48 (m, 4H), 7.44 – 7.36 (m, 4H), 6.52 – 6.41 (m, 1H), [6.36 – 6.26 (m, 0.24H) & 5.92 – 5.80 (m, 0.76H), *due to Z/E*], [1.97 (dd, *J* = 7.2, 1.8 Hz, 2.32H) & 1.93 (dd, *J* = 6.6, 1.7 Hz, 0.75H), *due to Z/E*];

^{13}C NMR (126 MHz, $CDCl_3$) δ 139.41, (138.2 & 137.9, *due to Z/E*), (137.4 & 137.1, *due to Z/E*), (133.4 & 133.3, *due to Z/E*), 130.58, (129.5 & 129.42, *due to Z/E*), (129.1 & 129.0, *due to Z/E*), (128.3 & 128.2, *due to Z/E*), (127.4 & 127.1, *due to Z/E*), (126.7 & 126.4, *due to Z/E*), (18.72 & 14.92, *due to Z/E*);

HRMS (ESI) calcd. for $C_{15}H_{13}ClNa$ $[M+Na]^+$ m/z 251.0598, found 251.0598;

IR (neat, cm^{-1}) 2913, 1473, 1093, 821, 787, 504;

m.p. 93.3 – 94.4 °C.

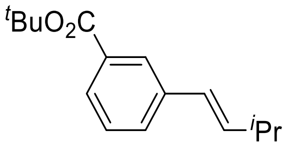

***tert*-Butyl (*E*)-3-(3-methylbut-1-en-1-yl)benzoate (4q)**:

Yield 60% (> 95:5 *Z/E*), colorless liquid;

^{1}H NMR (500 MHz, $CHCl_3$) δ 7.94 – 7.89 (m, 1H), 7.88 – 7.82 (m, 1H), 7.44 – 7.33 (m, 2H), 6.32 (d, *J* = 11.6 Hz, 1H), 5.53 (dd, *J* = 11.6, 10.2 Hz, 1H), 2.94 – 2.80 (m, 1H), 1.60 (s, 9H), 1.07 (s, 3H), 1.06 (s, 3H);

^{13}C NMR (126 MHz, $CDCl_3$) δ 165.8, 141.3, 137.9, 132.6, 132.0, 129.7, 128.1, 127.5, 125.7, 81.0, 28.3, 27.3, 23.2;

HRMS (ESI) calcd. for $C_{16}H_{22}O_2Na$ $[M+Na]^+$ m/z 269.1512, found 269.1513;

IR (neat, cm^{-1}) 2963, 1713, 1367, 1293, 1156, 773.

General procedure (D) for the preparation of olefins 1f', 4h and **4r**[3,4]

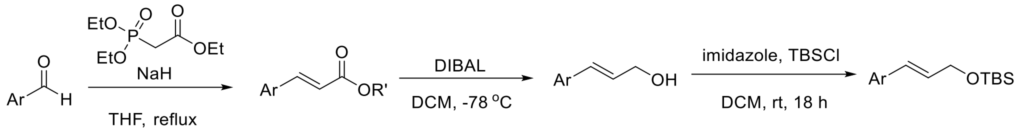

Step 1:

Phosphonoacetate (1.2 equiv) was added dropwise under argon over a period of 5 minutes to a stirred suspension of sodium hydride (55% dispersion in oil, 1.2 equiv) in dry THF (1.3 mL per 1.2 mmol of sodium hydride), and the resulting mixture was stirred at 0 °C for additional 15 min. A solution of aldehyde (1.0 equiv) in dry THF (1.0 M) was slowly added to the resulting mixture, and it was stirred at 0 °C for additional 0.5 h. Subsequently, the reaction mixture was refluxed overnight and then cooled to ambient temperature, and the reaction was quenched with saturated aqueous NH_4Cl. The aqueous layer was extracted with EtOAc (30 mL × 3). The combined organic layers were washed with brine, dried over Na_2SO_4, and concentrated in vacuo. The crude product was used without further purification.

Step 2:

To a stirred solution of ethyl acrylates (1.0 equiv) in anhydrous CH_2Cl_2 (0.71 M) was added dropwise diisobutylaluminum hydride (1.0 M in toluene, 2.2 equiv) under argon at −78 °C. The mixture was stirred at −78 °C for 1.5 h, and the reaction was quenched with 10% aqueous NaOH (1.0 mL per 1.0 mL of diisobutylaluminum hydride solution). The resulting mixture was allowed to warm to ambient temperature and stirred for additional 1 h. The aqueous layer was extracted with CH_2Cl_2 (30 mL × 3), and the combined organic layers were washed with brine, dried over Na_2SO_4, and concentrated in vacuo. Purification by flash silica gel column chromatography using petroleum ether/EtOAc (v/v = 5:1–3:1) as an eluent gave the corresponding allylic alcohols.

Step 3:

In a flame-dried 25 mL round bottom flask was added allylic alcohols (1 equiv) in dry CH_2Cl_2 (0.3 M). Then imidazole (1.5 equiv) and trialkylsilyl chloride (1.3 equiv) were added. The reaction was stirred at room temperature for 18 h. The reaction was

quenched with water. The reaction was extracted with ether (2 x 15 mL). Organic layers were combined, washed with brine (20 mL), dried over magnesium sulfate, filtered and concentrated in vacuum to provide a crude oil which was purified by flash chromatography to provide the desired product.

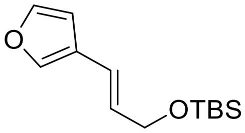

***tert*-Butyl((3-(furan-3-yl)allyl)oxy)dimethylsilane (1f')**:

Yield 90% (> 95:5 *E/Z*), colorless liquid;

^{1}H NMR (500 MHz, $CDCl_3$) δ 7.45 – 7.33 (m, 2H), 6.54 – 6.51 (m, 1H), 6.48 – 6.42 (m, 1H), 6.08 – 5.95 (m, 1H), 4.30 (dd, *J* = 5.2, 1.8 Hz, 2H), 0.95 (s, 9H), 0.11 (s, 6H);

^{13}C NMR (126 MHz, $CDCl_3$) δ 143.5, 140.3, 128.8, 124.0, 119.4, 107.7, 63.8, 26.1, 18.5, -5.0;

HRMS (ESI) calcd. for $C_{13}H_{22}O_2SiNa$ $[M+Na]^+$ m/z 261.1281, found 261.1282;

IR (neat, cm^{-1}) 2956, 2856, 1254, 1071, 836, 776.

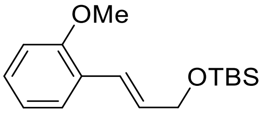

***tert*-Butyl((3-(2-methoxyphenyl)allyl)oxy)dimethylsilane** (**4h**):

Yield 80% (~ 5:1 *E/Z*), colorless liquid;

^{1}H NMR (500 MHz, $CDCl_3$) δ [7.45 (dd, *J* = 7.6, 1.7 Hz, 0.83H) & 7.14 (dd, *J* = 7.5, 1.7 Hz, 0.16H), *due to Z/E*], [7.29 – 7.26 (m, 0.13H) & 7.25 – 7.19 (m, 0.86H), *due to Z/E*], 6.96 – 6.89 (m, 2H), [6.87 (d, *J* = 8.2 Hz, 1H) & 6.66 (d, *J* = 11.7 Hz, 0.16H), *due to Z/E*], [6.36 – 6.25 (m, 0.82H) & 5.92 – 5.84 (m, 0.16H), *due to Z/E*], [4.39 – 4.37 (m, 1.81H) & 4.37 – 4.36 (m, 0.18H), *due to Z/E*], [3.85 (s, 2.44H) & 3.84 (s, 0.55H), *due to Z/E*], [0.97 (s, 7.40H) & 0.91 (s, 1.60H), *due to Z/E*], [0.13 (s, 4.96H) & 0.06 (s, 1.04H), *due to Z/E*];

^{13}C NMR (126 MHz, $CDCl_3$) δ (157.0 & 156.8, *due to Z/E*), 132.0, (130.3 & 129.9, *due to Z/E*), (128.7 & 128.4, *due to Z/E*), (127.0 & 126.3, *due to Z/E*), (125.4 & 124.7, *due to Z/E*), (120.7 & 120.1, *due to Z/E*), (110.9 &110.4, *due to Z/E*), (64.5 & 60.5, *due to Z/E*), (55.5 & 55.5, *due to Z/E*), (26.1 & 26.0, *due to Z/E*), (18.5 & 18.4, *due to Z/E*), (–4.9 & –5.0, *due to Z/E*);

HRMS (ESI) calcd. for $C_{16}H_{26}O_2SiNa$ $[M+Na]^+$ m/z 301.1594, found 301.1592;

IR (neat, cm^{-1}) 2929, 2855, 1437, 1241, 830, 747.

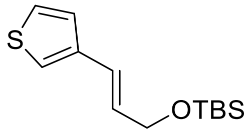

***tert*-Butyldimethyl((3-(thiophen-3-yl)allyl)oxy)silane** (**4r**):

Yield 85% (> 95:5 *E/Z*), colorless liquid;

^{1}H NMR (500 MHz, $CDCl_3$) δ 7.28 – 7.25 (m, 1H), 7.23 – 7.20 (m, 1H), 7.15 – 7.13 (m, 1H), 6.64 – 6.57 (m, 1H), 6.19 – 6.12 (m, 1H), 4.33 (dd, *J* = 5.1, 1.8 Hz, 2H), 0.96 (s, 9H), 0.13 (s, 6H);

^{13}C NMR (126 MHz, $CDCl_3$) δ 139.8, 129.1, 125.9, 125.2, 123.9, 121.7, 63.8, 26.1, 18.6, –5.0;

HRMS (ESI) calcd. for $C_{13}H_{22}OSSiNa$ $[M+Na]^+$ m/z 277.1053, found 277.1050;

IR (neat, cm^{-1}) 2953, 2855, 1252, 1061, 831, 771.

General procedure (E) for the preparation of olefin (*E*)-4h-D[4,5,6]

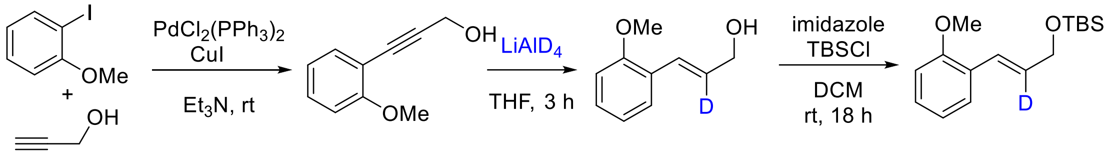

Step 1:

To a stirred solution of substituted iodobenzene (10 mmol, 1.0 equiv) in triethylamine (50 mL) under nitrogen were sequentially added $Pd(PPh_3)_2Cl_2$ (2 mol %) and CuI (4 mol %) at room temperature. The mixture was allowed to stir for 10 min. Then propargyl alcohol (11 mmol, 1.1 equiv) was added. The mixture was allowed to stir overnight. After the reaction was finished, water was added and the solution was extracted with ethyl acetate; The combined extract was dried with anhydrous $MgSO_4$. Solvent was removed, and the residue was separated by column chromatography to give the aryl substituted propargyl alcohol.

Step 2:

To a flame dried round-bottomed flask charged with $LiAlD_4$ (210 mg, 5 mmol, 100 mol%) under nitrogen was added THF (15 mL, 0.33 M with respect to propargylic alcohol). The reaction vessel was placed in an ice batch. After 5 minutes a solution of aryl substituted propargyl alcohol (5 mmol, 100 mol%) in dry THF (5 mL, 1.0 M with respect to propargylic alcohol) was added slowly and the mixture was stirred at room temperature for 3 hours. After the reaction vessel was placed in an ice batch, water (1 mL), NaOH (1 mL, 10% aqueous solution) and water (3 mL) were added to the reaction mixture. After 10 minutes, $MgSO_4$ was added and the reaction mixture was filtered (celite) with the aid of CH_2Cl_2 (10 mL) and the filtrate was concentrated under reduced pressure. The resulting oily residue was subjected to the next step without further purification.

Step 3:

To a flame-dried 25 mL round bottom flask was added allylic alcohol (1 equiv) in dry DCM (0.3 M). Then imidazole (1.5 equiv) and trialkylsilyl chloride (1.3 equiv) were added. The reaction was stirred at room temperature for 18 h. The reaction was quenched with water and was extracted with ether (2 x 15 mL). Organic layers were

combined, washed with brine (20 mL), dried over magnesium sulfate, filtered and concentrated in vacuum to provide a crude oil which was purified by flash chromatography to provide the desired product.

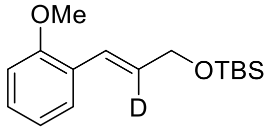

(*E*)-*tert*-Butyl((3-(2-methoxyphenyl)allyl-2-*d*)oxy)dimethylsilane (4h-D):

Yield 65%, colorless liquid;

^{1}H NMR (500 MHz, $CDCl_3$) δ 7.43 (dd, *J* = 7.6, 1.7 Hz, 1H), 7.24 – 7.18 (m, 1H), 6.94 – 6.83 (m, 3H), 4.36 (d, *J* = 1.8 Hz, 2H), 3.84 (s, 3H), 0.95 (s, 9H), 0.12 (s, 6H);

^{13}C NMR (126 MHz, $CDCl_3$) δ 156.8, 129.6 (t, *J* = 23.6 Hz), 128.4, 127.1, 126.3, 124.6, 120.7, 110.9, 64.5, 55.6, 26.1, 18.6, –4.9;

HRMS (ESI) calcd. for $C_{16}H_{25}DO_2SiNa$ $[M+Na]^+$ m/z 302.1657, found 302.1658;

IR (neat, cm^{-1}) 2928, 2855, 1462, 1244, 833, 747.

Isotopic Labelling Experiments

a) NiD experiment: NiD *syn*-hydrometallation is not the enantio-determining step

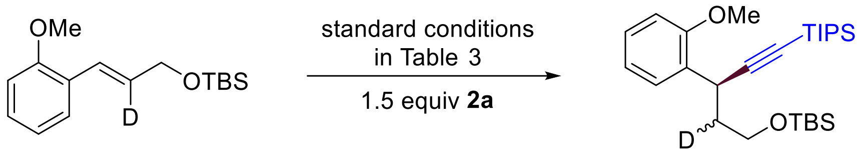

(*E*)-**4h-D** **5h-D** 81% yield, 0.55:0.45 dr, 92% ee

tert*-Butyl(((2*S*,3*S*)-3-(2-methoxyphenyl)-5-(triisopropylsilyl)pent-4-yn-1-yl-2-*d*)oxy)dimethylsilane** (Figure 5, **5h-D**). From **(*E*)-*tert*-butyl((3-(2-methoxyphenyl)allyl-2-*d*)oxy)dimethylsilane** (**4h-D**) (55.9 mg, 0.20 mmol) and **(bromoethynyl)triisopropylsilane** (**2a**) (78.4 mg, 0.30 mmol), the title compound was prepared following the general procedure **B** using $NiI_2 \cdot xH_2O$ (3.8 mg, 5.0 mol%), (*S*)-**L (3.3 mg, 6.0 mol%), $K_3PO_4 \cdot H_2O$ (115.1 mg, 2.5 equiv), NaI (60.0 mg, 2.0 equiv), $(MeO)_3SiH$ (64 μL, 0.5 mmol, 2.5 equiv), anhydrous $PhCF_3$ (1.0 mL). The reaction mixture was stirred for 12 h at 0 °C. The crude material was purified by flash column chromatography (petroleum ether/EtOAc = 100:1) to provide the title compound as a colorless liquid in 81% yield (74.4 mg).

^{1}H NMR (500 MHz, $CDCl_3$) δ 7.64 – 7.58 (m, 1H), 7.25 – 7.17 (m, 1H), 6.99 – 6.91 (m, 1H), 6.84 (dd, *J* = 8.2, 1.1 Hz, 1H), 4.32 – 4.26 (m, 1H), 3.92 – 3.86 (m, 1H), 3.83 – 3.78 (m, 4H), [2.03 – 1.95 (m, 0.45H) & 1.77 – 1.68 (m, 0.55H), *due to dr*], 1.15 – 1.05 (m, 21H), 0.92 (s, 9H), 0.08 (s, 3H), 0.07 (s, 3H);

^{13}C NMR (126 MHz, $CDCl_3$) δ 156.1, 130.4, 128.6, 127.7, 120.6, 110.3, 110.1, 82.7, 61.5, 55.3, 39.8 – 39.4 (m, 1C), 29.1, 26.0, 18.9, 18.8, 18.4, 11.4, –5.0, –5.1;

^{2}H NMR (92 MHz, $CDCl_3$) δ 2.00 (0.55D), 1.74 (0.45D);

HRMS (ESI) calcd. for $C_{27}H_{47}DO_2Si_2Na$ $[M+Na]^+$ m/z 484.3148, found 484.3147;

IR (neat, cm^{-1}) 2943, 2864, 2164, 1244, 1090, 835;

$[\alpha]_D^{18}$ = –31.2 (c = 1.24, $CHCl_3$); 92% *ee*;

HPLC analysis CHIRALCEL two connected OD-H columns, *n*-hexane/*iso*-propanol = 100/0, flow rate 0.5 mL/min, 220 nm UV detector, t_R (minor) = 14.5 min, t_R (major) = 18.0 min.

b) Crossover experiment: no intermolecular H/D scrambled crossover products

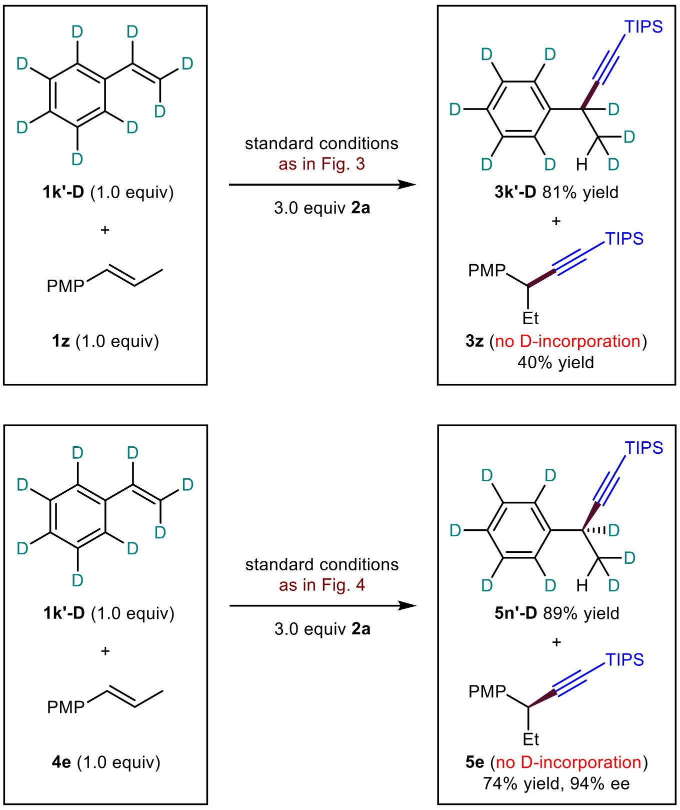

From **styrene-d_8 (1k'-D)** (11.2 mg, 0.1 mmol) and **(*E*)-1-methoxy-4-(prop-1-en-1-yl)benzene** (**1z**) (14.8 mg, 0.1 mmol). the title compound was prepared following the general procedure **A** using $NiI_2 \cdot xH_2O$ (3.8 mg, 10 mol%), **L** (3.3 mg, 12 mol%), Na_2CO_3 (42.4 mg, 4.0 equiv), NaI (3.0 mg, 0.2 equiv), PMHS (60 μL, 1.0 mmol, 10.0 equiv), anhydrous DME (1.0 mL). The reaction mixture was stirred for 16 h at 25 °C. The crude material was purified by flash column chromatography (petroleum ether) to provide the title compound.

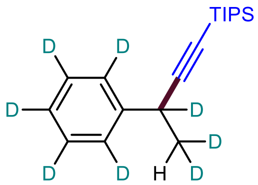

Triisopropyl(3-(phenyl-d_5)but-1-yn-1-yl-4,4-d_3)silane (Figure 5, **3k'-D**):

Yield 81%, colorless liquid;

^{1}H NMR (400 MHz, $CDCl_3$) δ 1.49 (s, 1H), 1.15 – 1.06 (m, 21H);

^{13}**C NMR** (101 MHz, $CDCl_3$) δ 143.2, [128.2 & 128.0 & 127.7] (1C), [126.8 & 126.6 & 126.3] (1C), [126.0 & 125.8] (1C), 111.4, 82.3, 24.9 – 24.1, 18.8, 11.4;

^{2}**H NMR** (92 MHz, $CDCl_3$) [7.44 & 7.35, (5D)], 3.78 (1D), 1.47 (2D);

HRMS (ESI) calcd. for $C_{19}H_{22}D_8SiNa$ $[M+H]^+$ m/z 295.2692, found 295.2686;

IR (neat, cm^{-1}) 2942, 2864, 2167, 1462, 996, 882;

From **styrene-*d*$_8$ (1k'-D)** (11.2 mg, 0.1 mmol) and **(*E*)-1-methoxy-4-(prop-1-en-1-yl)benzene** (**4e**) (14.8 mg, 0.1 mmol). the title compound was prepared following the general procedure **B** using $NiI_2 \cdot xH_2O$ (3.8 mg, 10 mol%), (*S*)-**L*** (3.3 mg, 12 mol%), $K_3PO_4 \cdot H_2O$ (115.1 mg, 5.0 equiv), NaI (60.0 mg, 4.0 equiv), $(MeO)_3SiH$ (64 μL, 0.5 mmol, 5.0 equiv), anhydrous $PhCF_3$ (1.0 mL). The reaction mixture was stirred for 12 h at 0 °C. The crude material was purified by flash column chromatography (petroleum ether) to provide the title compound.

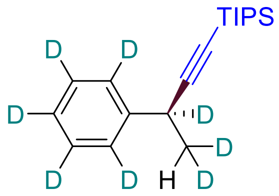

(*S*)-Triisopropyl(3-(phenyl-*d*$_5$)but-1-yn-1-yl-3,4,4-*d*$_3$)silane (Figure 5, **5n'-D**):

Yield 89%, colorless liquid;

^{1}**H NMR** (500 MHz, $CDCl_3$) δ 1.47 (s, 1H), 1.15 – 0.77 (m, 21H);

^{13}**C NMR** (126 MHz, $CDCl_3$) δ 143.2, [128.2 & 128.0 & 127.8] (1C), [126.8 & 126.6 & 126.4] (1C), [126.2 & 126.0 & 125.9] (1C), 111.4, 82.3, 24.9 – 24.2 (m, 1C), 18.8, 11.4;

^{2}**H NMR** (92 MHz, $CDCl_3$) δ [7.46 & 7.36, (5D)], 3.79 (1D), 1.48 (2D);

HRMS (ESI) calcd. for $C_{19}H_{22}D_8SiNa$ $[M+Na]^+$ m/z 317.2511, found 317.2507;

IR (neat, cm^{-1}) 2942, 2864, 2168, 1462, 882, 659;

Supplementary Note 1. Spectroscopic Data (GC Traces)

1) *n*-Dodecane (t_R = 2.0 min) was used as internal standard for GC yield.

2) GC analysis was performed on Agilent 7890B gas chromatograph with an FID detector using a J & W DB-1 column (10 m, 0.1 mm I.D.). GC-MS analysis was performed on an Agilent 7890B gas chromatograph with 5977A MSD mass spectrum using an HP-5 MS column (30 m, 0.25 mm I.D.).

3) GC method: 100 method starts at 100 °C holds the oven at this temperature for 1 minute, then ramp of 50 °C/min till 250 °C and hold the oven at this temperature for 3 minutes (or 5 minutes for 100B method, or 16 minutes for 100C method).

4) rr refers to regioisomeric ratio, represents the ratio of major product to the sum of all other isomers as determined by GC, all isomers' peaks were confirmed by GC-MS analysis.

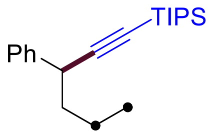

3a

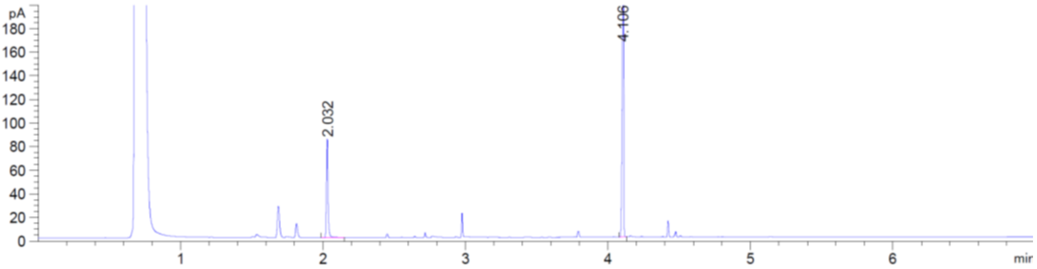

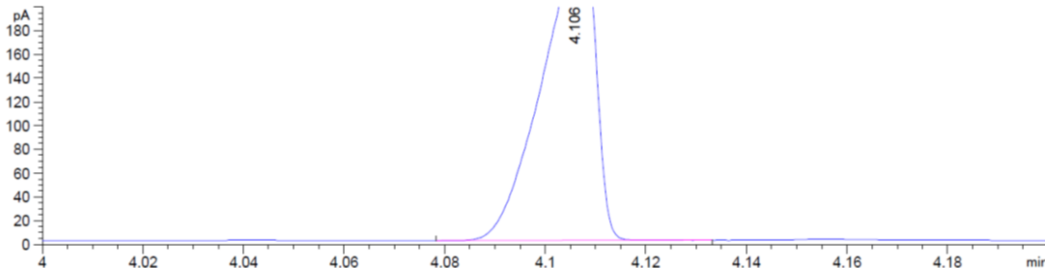

Peak #	Time [min]	Type	Width [min]	Area [pA*s]	Height [pA]	Area %
1	4.106	BB	0.0113	186.16582	244.35548	1.000e2

Supplementary Fig. 3. GC spectra of compound **3a**, Related to Figure 3.

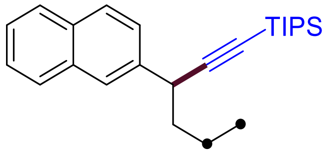

3b

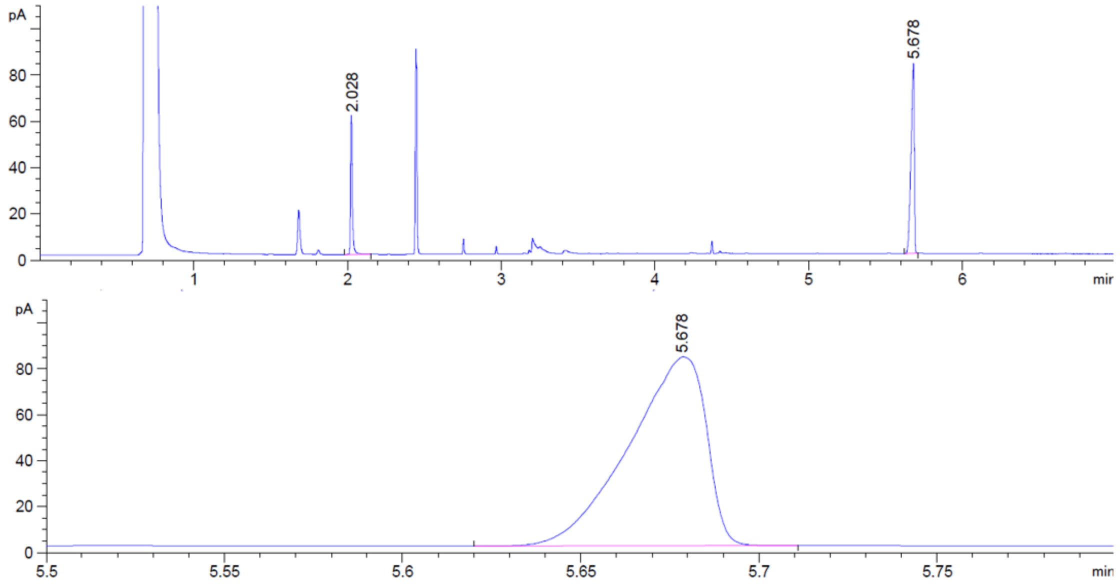

Peak #	Time [min]	Type	Width [min]	Area [pA*s]	Height [pA]	Area %
1	5.678	BB	0.0232	123.87083	82.12784	1.000e2

Supplementary Fig. 4. GC spectra of compound **3b**, Related to Figure 3.

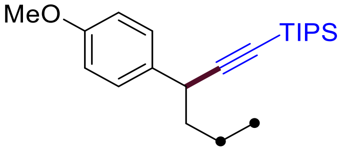

3c

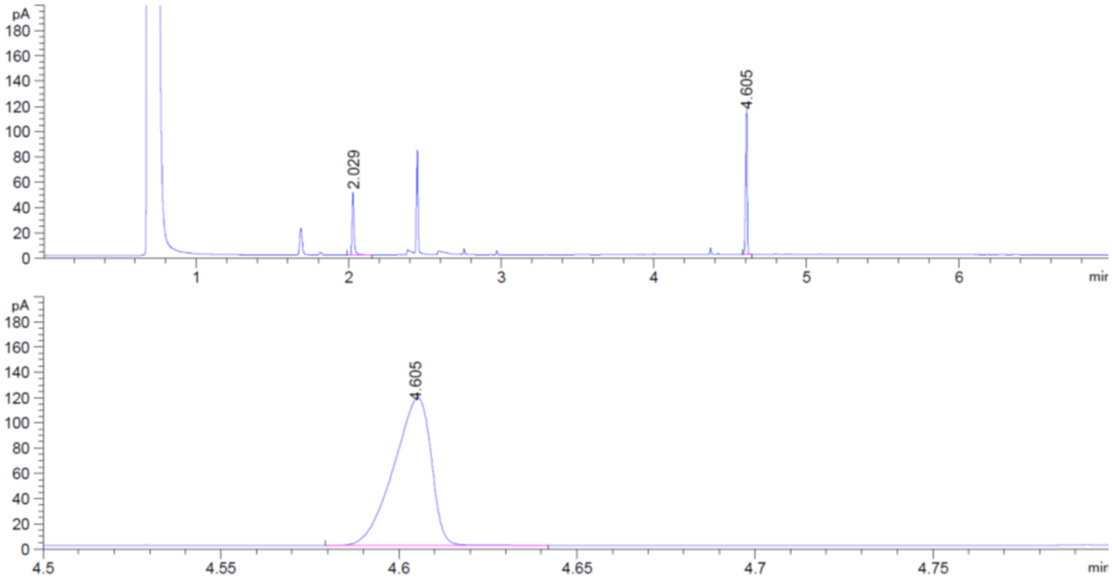

Peak #	Time [min]	Type	Width [min]	Area [pA*s]	Height [pA]	Area %
1	4.605	BB	0.0121	85.66869	111.75015	1.000e2

Supplementary Fig. 5. GC spectra of compound **3c**, Related to Figure 3.

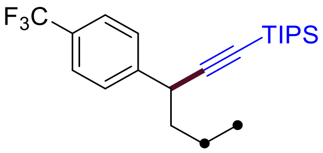

3d

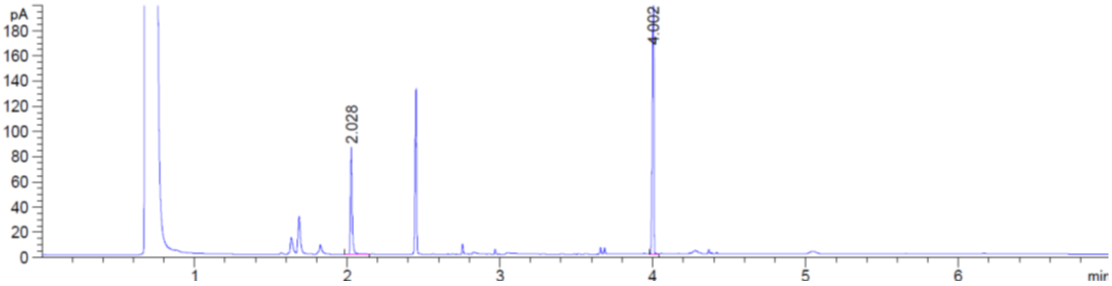

Peak #	Time [min]	Type	Width [min]	Area [pA*s]	Height [pA]	Area %
1	4.002	BB	0.0114	154.49745	219.06358	1.000e2

Supplementary Fig. 6. GC spectra of compound **3d**, Related to Figure 3.

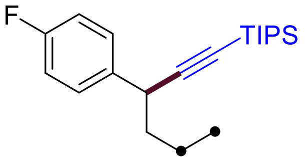

3e

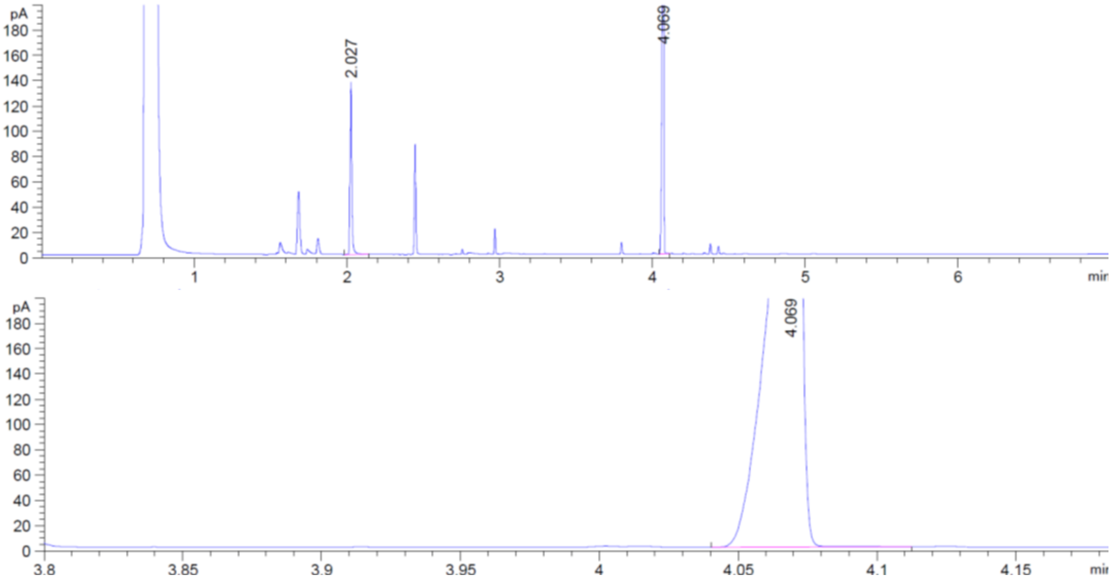

Peak #	Time [min]	Type	Width [min]	Area [pA*s]	Height [pA]	Area %
1	4.069	BB	0.0132	306.20294	355.76334	1.000e2

Supplementary Fig. 7. GC spectra of compound **3e**, Related to Figure 3.

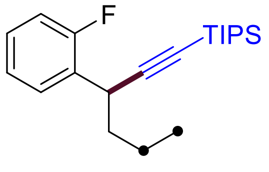

3f

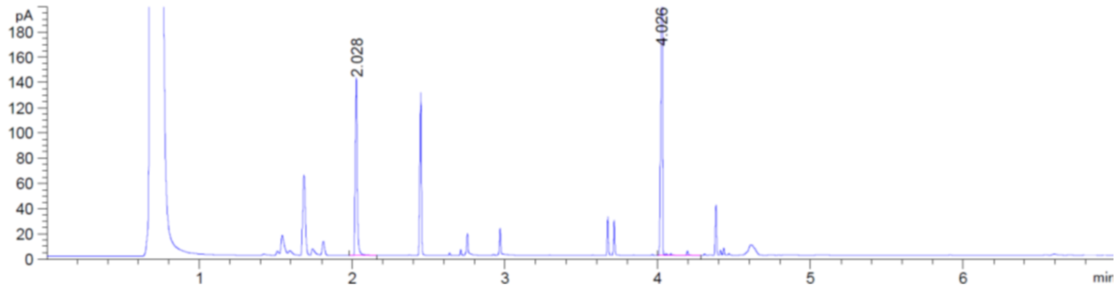

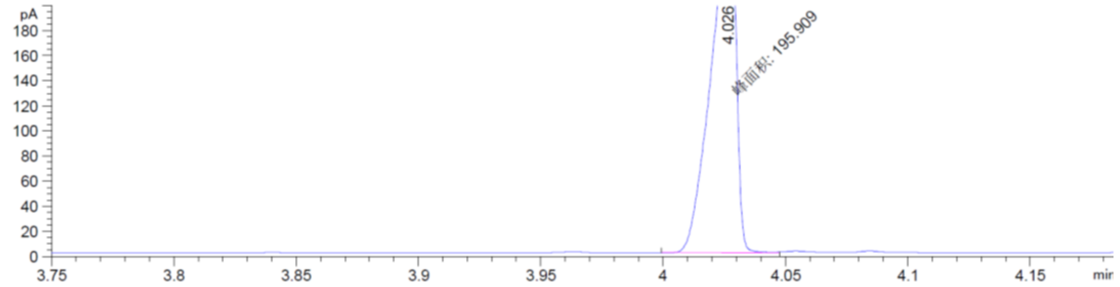

Peak #	Time [min]	Type	Width [min]	Area [pA*s]	Height [pA]	Area %
1	4.026	MM	0.0113	195.90855	290.07196	1.000e2

Supplementary Fig. 8. GC spectra of compound **3f**, Related to Figure 3.

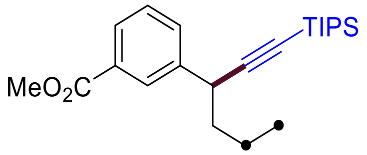

3g

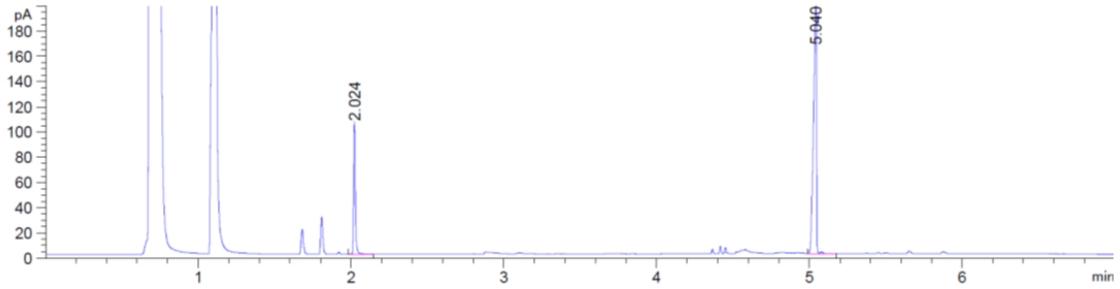

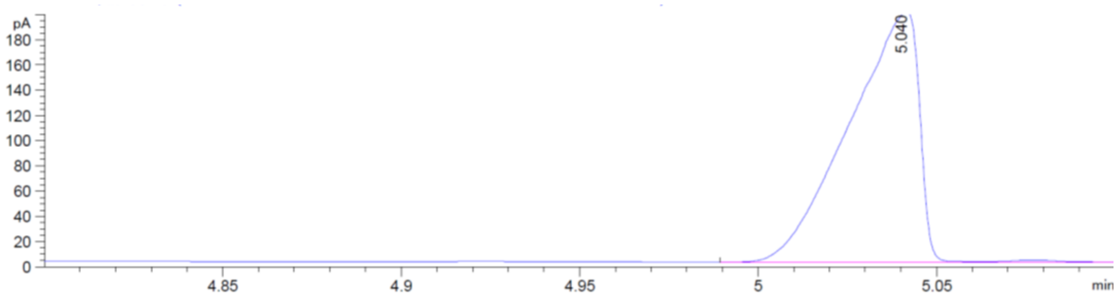

Peak #	Time [min]	Type	Width [min]	Area [pA*s]	Height [pA]	Area %
1	5.040	BV R	0.0188	271.26648	201.11446	1.000e2

Supplementary Fig. 9. GC spectra of compound **3g**, Related to Figure 3.

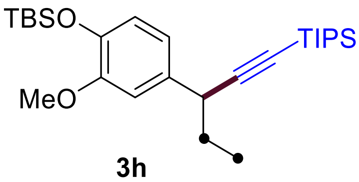

3h

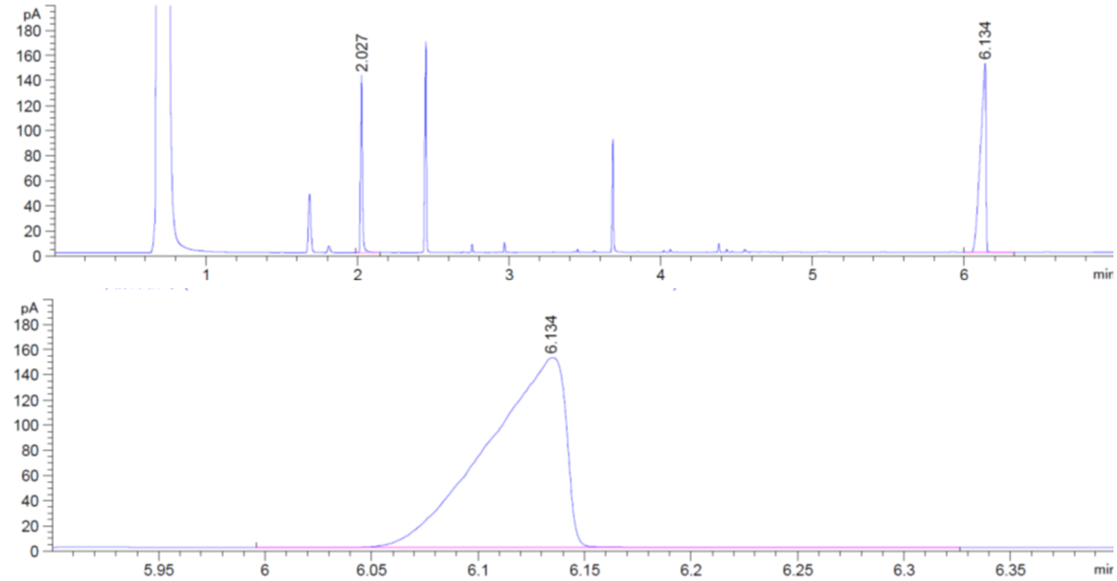

Peak #	Time [min]	Type	Width [min]	Area [pA*s]	Height [pA]	Area %
1	6.134	BB	0.0337	383.35068	150.40926	1.000e2

Supplementary Fig. 10. GC spectra of compound **3h**, Related to Figure 3.

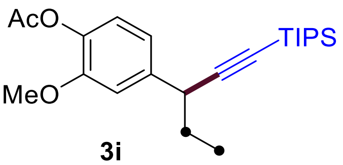

3i

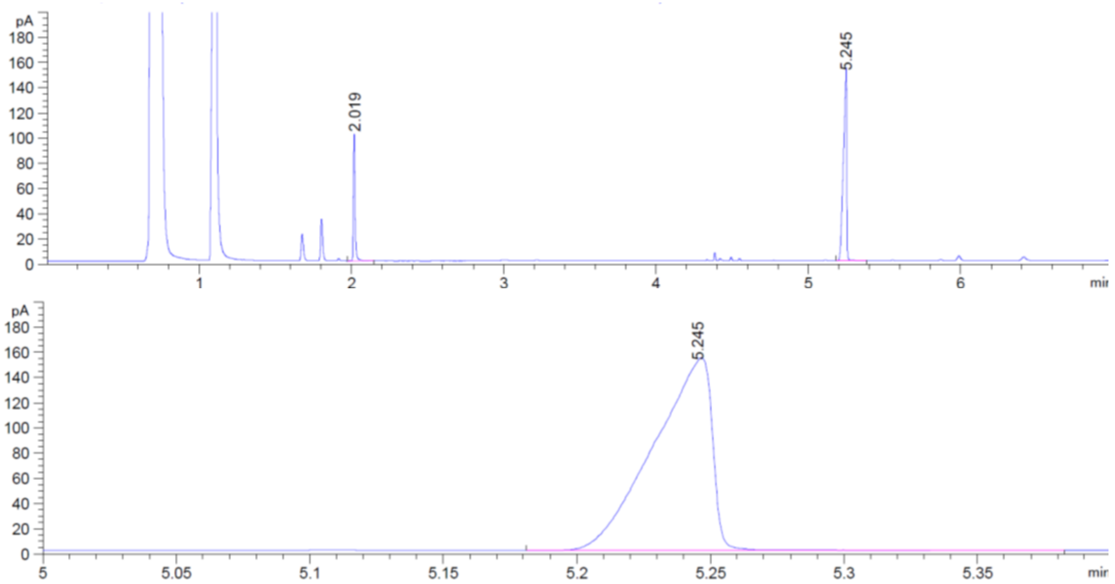

Peak #	Time [min]	Type	Width [min]	Area [pA*s]	Height [pA]	Area %
1	5.245	BB	0.0224	232.19344	147.24724	1.000e2

Supplementary Fig. 11. GC spectra of compound **3i**, Related to Figure 3.

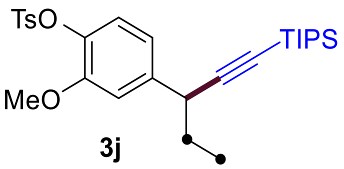

3j

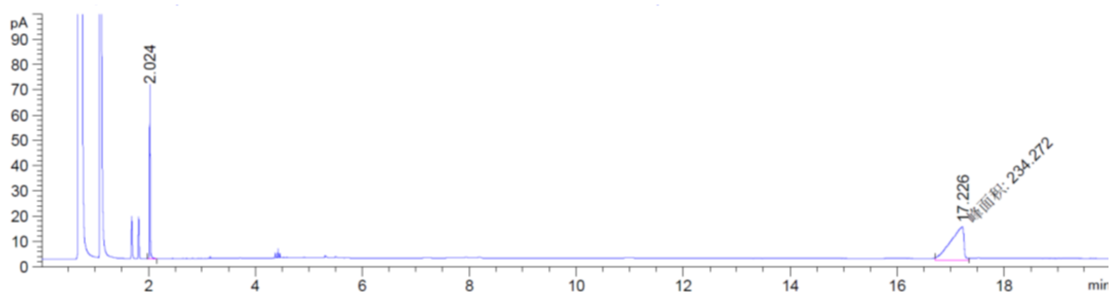

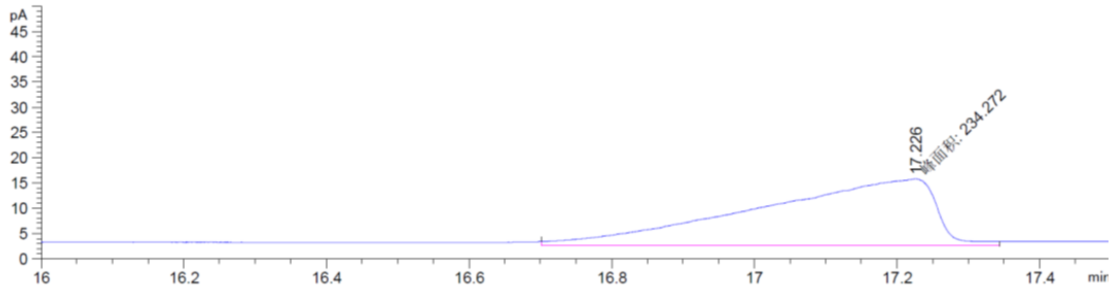

Peak #	Time [min]	Type	Width [min]	Area [pA*s]	Height [pA]	Area %
1	17.226	MM	0.2940	234.27243	13.28021	1.000e2

Supplementary Fig. 12. GC spectra of compound **3j**, Related to Figure 3.

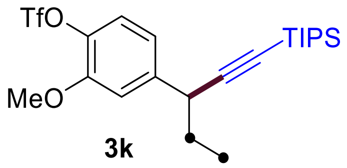

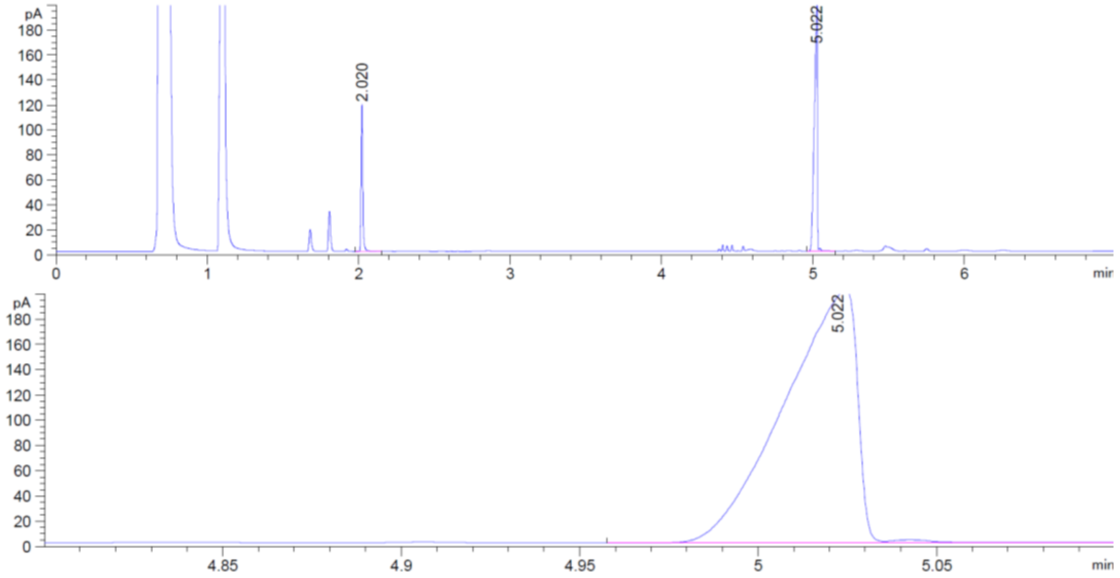

Peak #	Time [min]	Type	Width [min]	Area [pA*s]	Height [pA]	Area %
1	5.022	BB	0.0218	285.20779	195.70508	1.000e2

Supplementary Fig. 13. GC spectra of compound **3k**, Related to Figure 3.

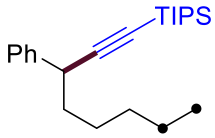

3l

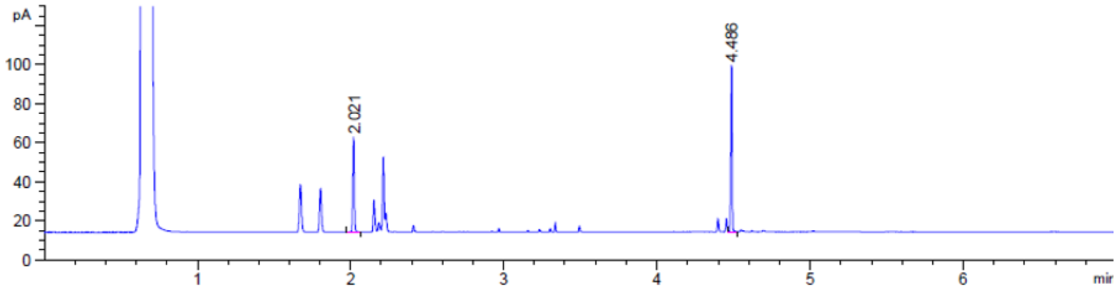

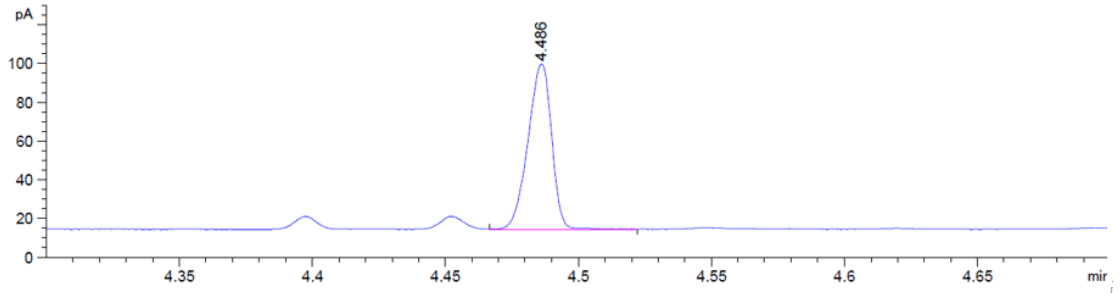

Peak #	Time [min]	Type	Width [min]	Area [pA*s]	Height [pA]	Area %
1	4.486	VB	0.0105	52.76726	84.63114	1.000e2

Supplementary Fig. 14. GC spectra of compound **3l**, Related to Figure 3.

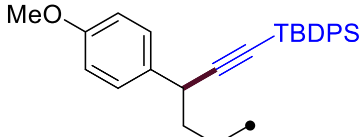

3m

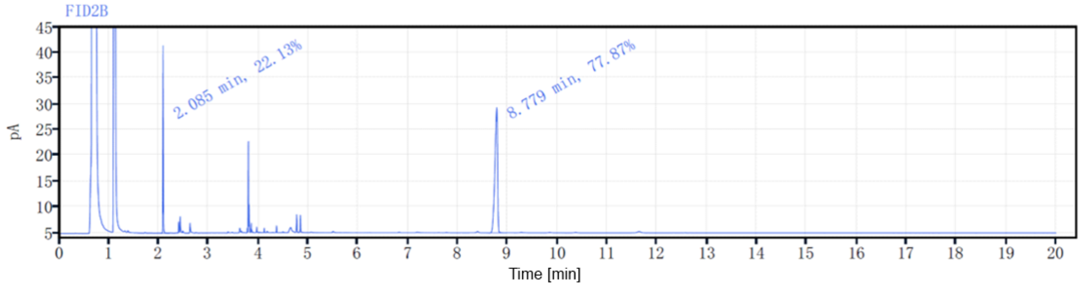

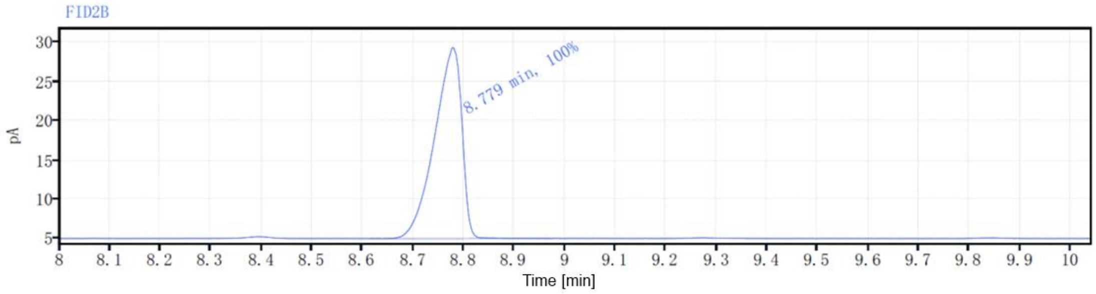

RetTime (min)	Signal	Width (time)	Area	Height	Area%
8.779	FID2B	0.359	91.9	24.4	100.00

Supplementary Fig. 15. GC spectra of compound **3m**, Related to Figure 3.

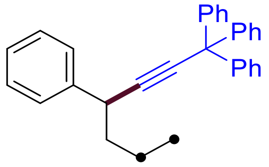

3n

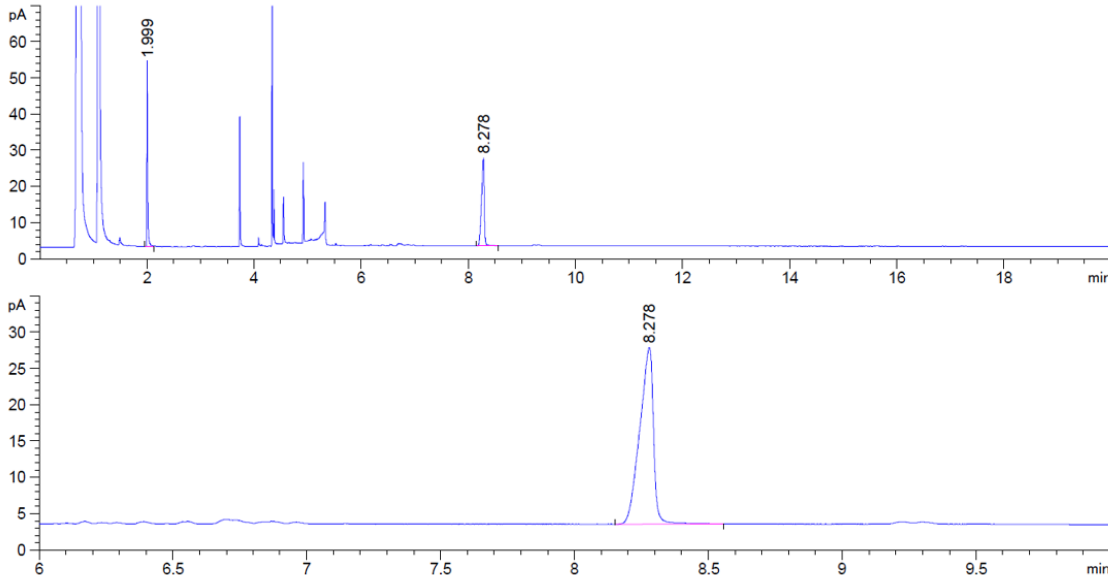

Peak #	Time [min]	Type	Width [min]	Area [pA*s]	Height [pA]	Area %
1	8.278	BB	0.0515	87.30582	24.33629	1.000e2

Supplementary Fig. 16. GC spectra of compound **3n**, Related to Figure 3.

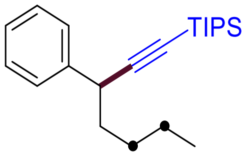

3o

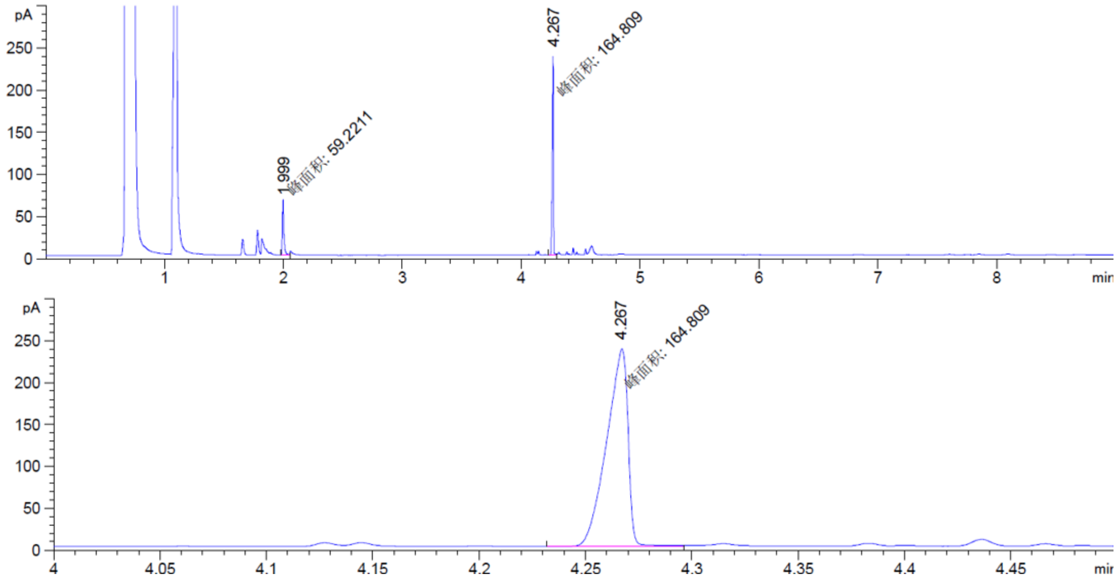

Peak #	Time [min]	Type	Width [min]	Area [pA*s]	Height [pA]	Area %
1	4.267	MF	0.0114	164.80936	241.84032	1.000e2

Supplementary Fig. 17. GC spectra of compound **3o**, Related to Figure 3.

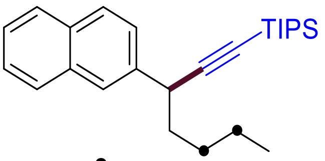

3p

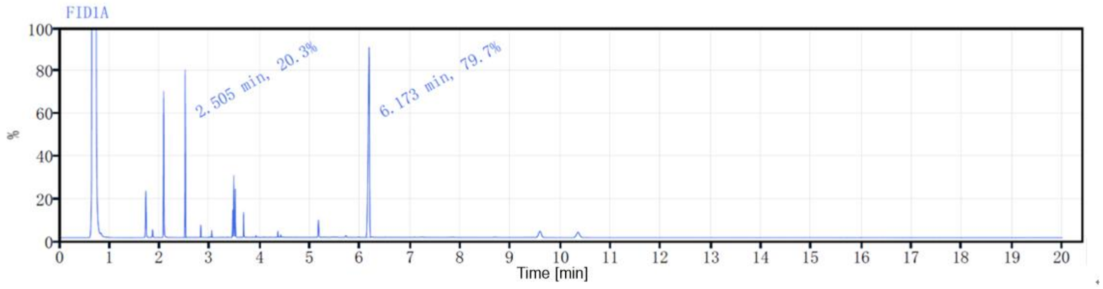

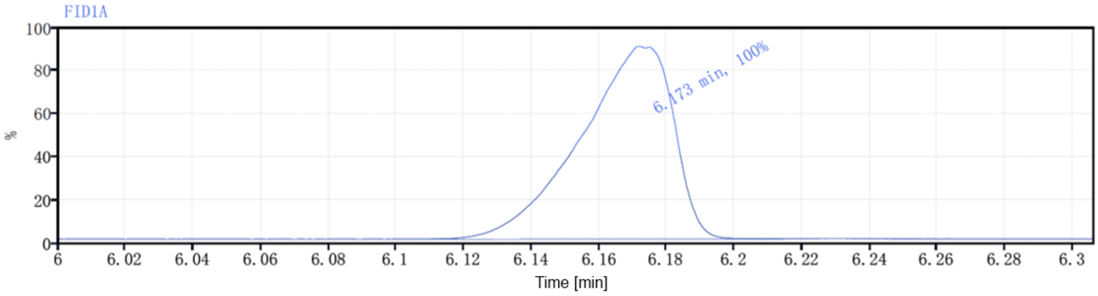

RetTime (min)	Signal	Width (min)	Area	Height	Area%
6. 173	FID1A	0. 118	116. 5	60. 7	100. 00

Supplementary Fig. 18. GC spectra of compound **3p**, Related to Figure 3.

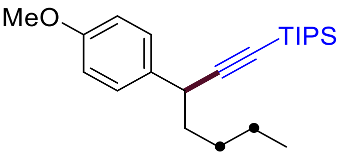

3q

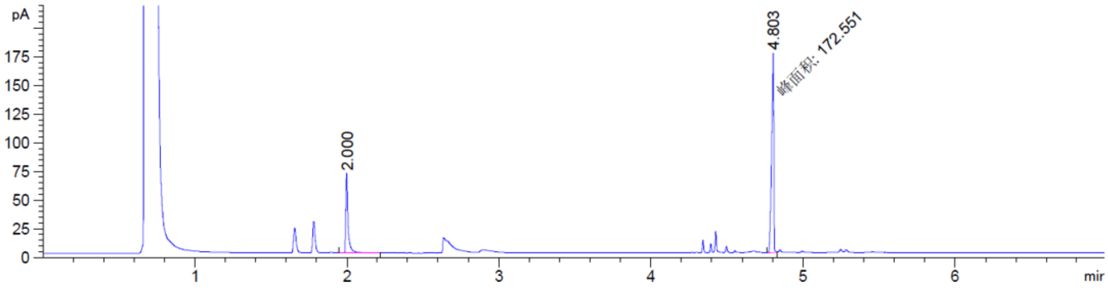

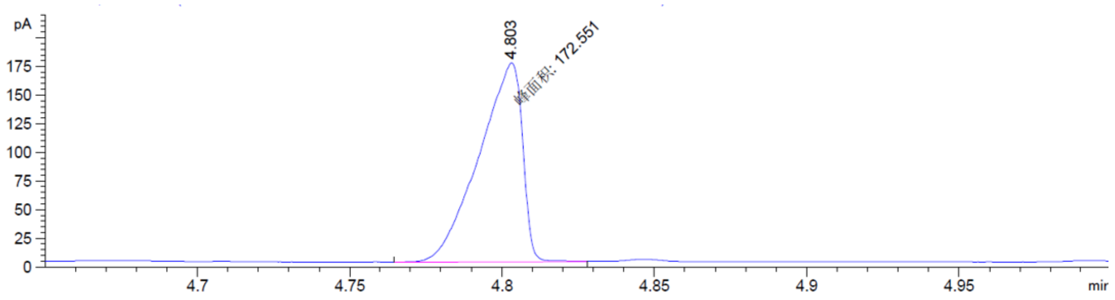

Peak #	Time [min]	Type	Width [min]	Area [pA*s]	Height [pA]	Area %
1	4.803	MF	0.0166	172.55072	173.33826	1.000e2

Supplementary Fig. 19. GC spectra of compound **3q**, Related to Figure 3.

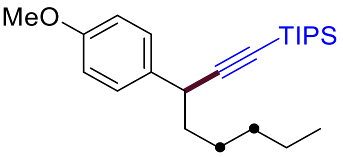

3r

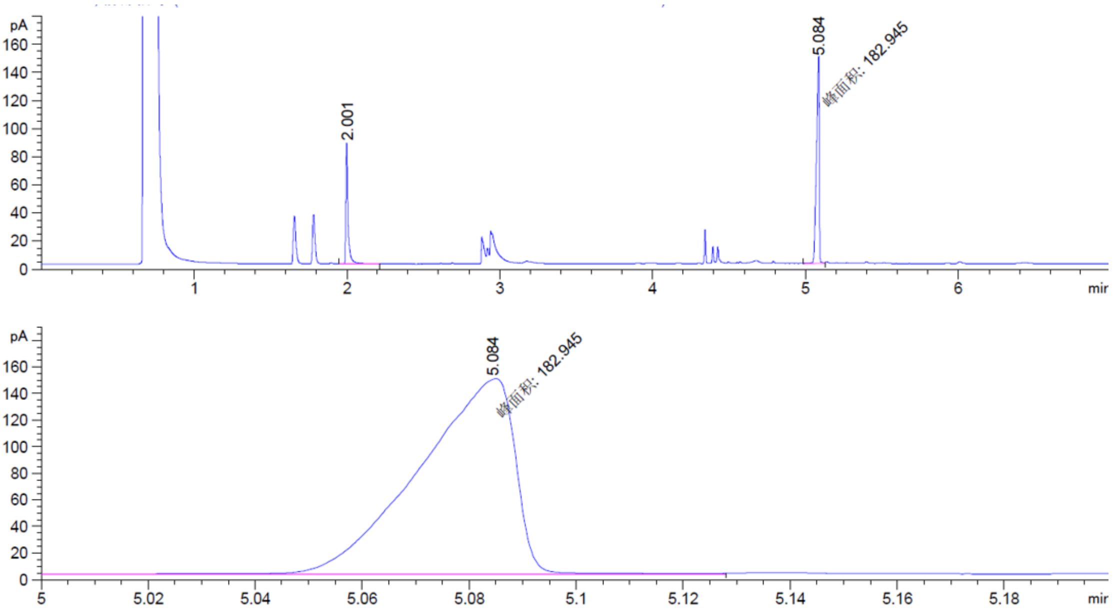

Peak #	Time [min]	Type	Width [min]	Area [pA*s]	Height [pA]	Area %
1	5.084	MF	0.0209	182.94456	146.10146	1.000e2

Supplementary Fig. 20. GC spectra of compound **3r**, Related to Figure 3.

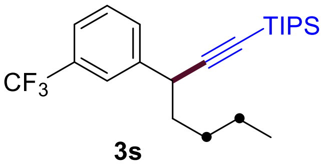

3s

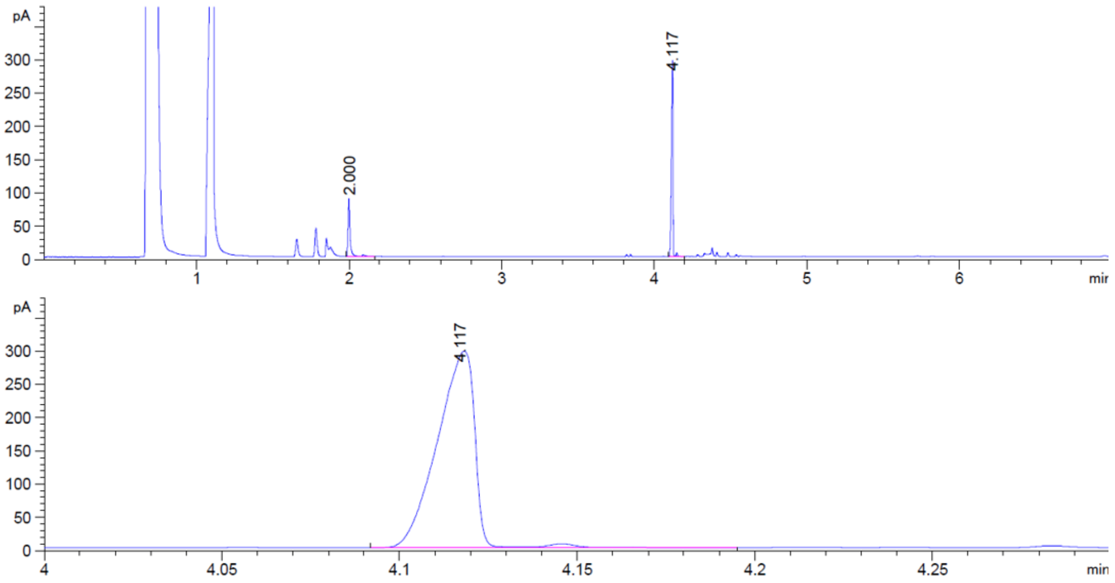

Peak #	Time [min]	Type	Width [min]	Area [pA*s]	Height [pA]	Area %
1	4.117	BV R	0.0123	218.62238	273.70059	1.000e2

Supplementary Fig. 21. GC spectra of compound **3s**, Related to Figure 3.

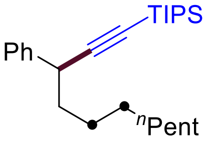

3t

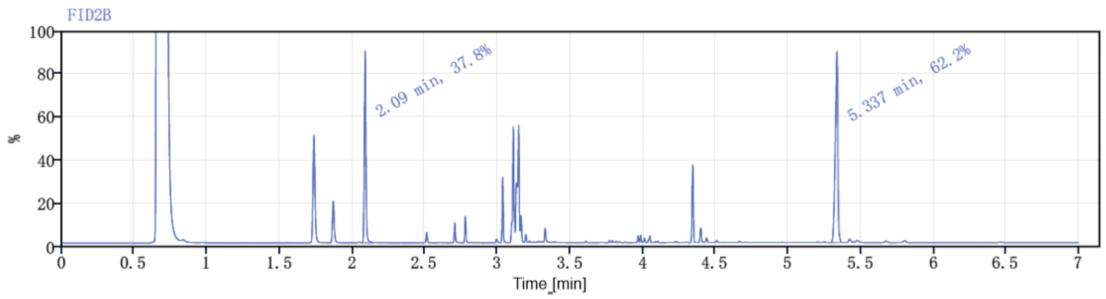

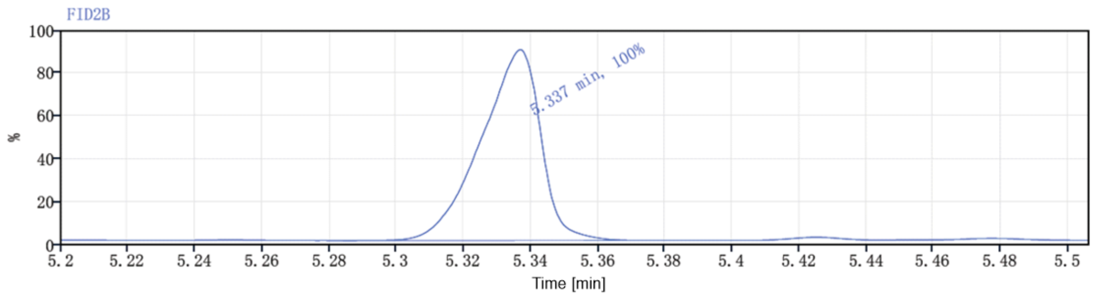

RetTime (min)	Signal	Width (min)	Area	Height	Area%
5.337	FID2B	0.114	118.5	95.6	100.00

Supplementary Fig. 22. GC spectra of compound **3t**, Related to Figure 3.

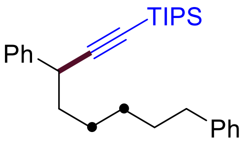

3u

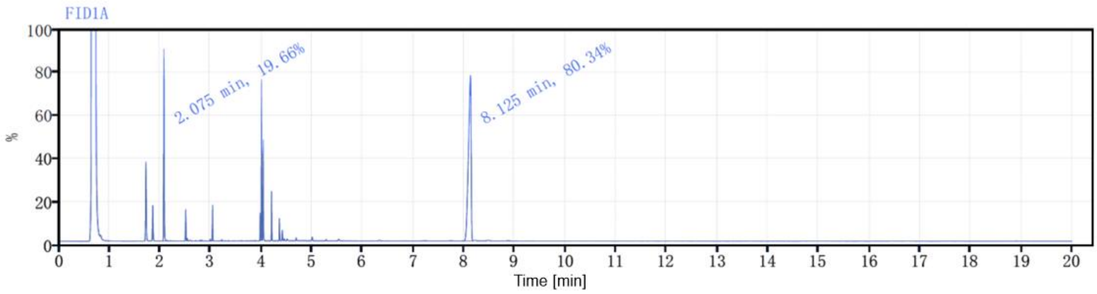

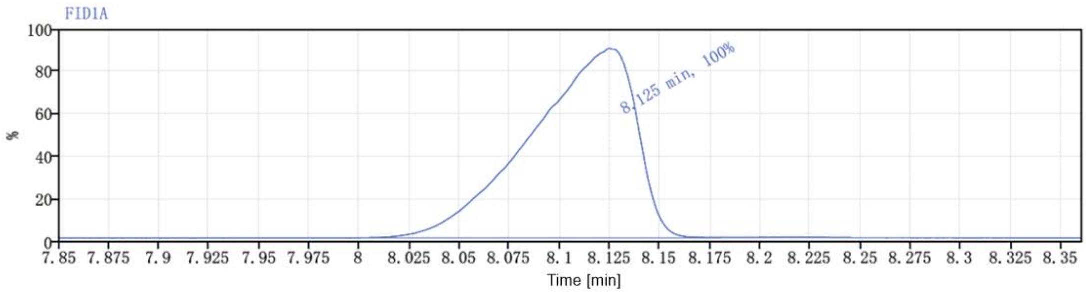

RetTime (min)	Signal	Width (min)	Area	Height	Area%
8.125	FID1A	0.188	147.4	40.2	100.00

Supplementary Fig. 23. GC spectra of compound **3u**, Related to Figure 3.

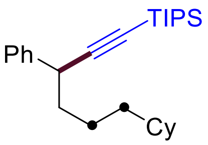

3v

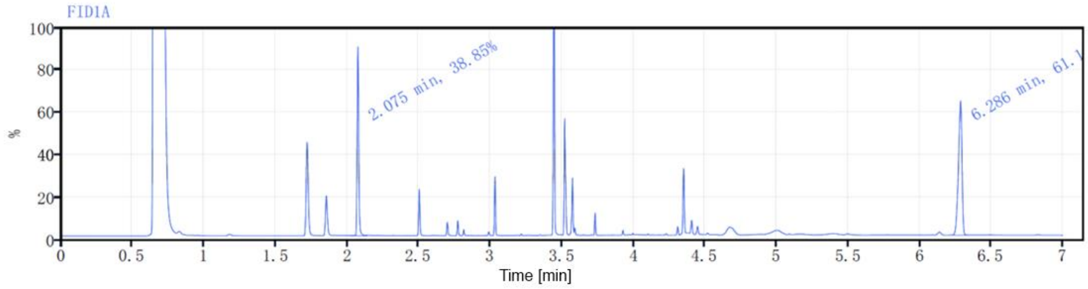

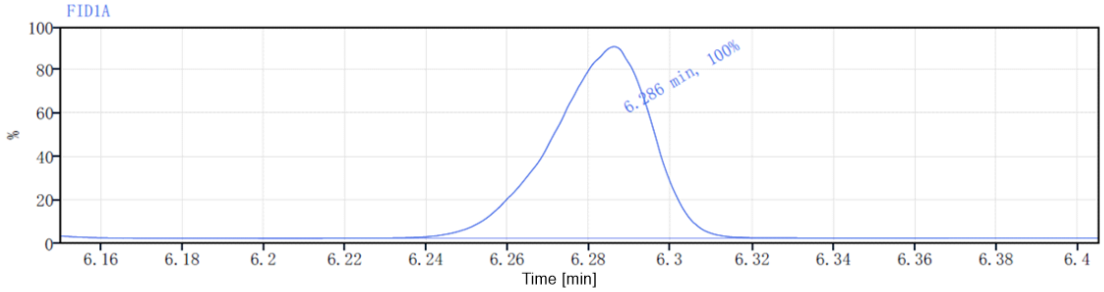

RetTime (min)	Signal	Width (min)	Area	Height	Area%
6.286	FID1A	0.170	62.0	35.9	100.00

Supplementary Fig. 24. GC spectra of compound **3v**, Related to Figure 3.

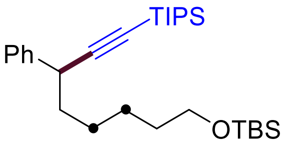

3w

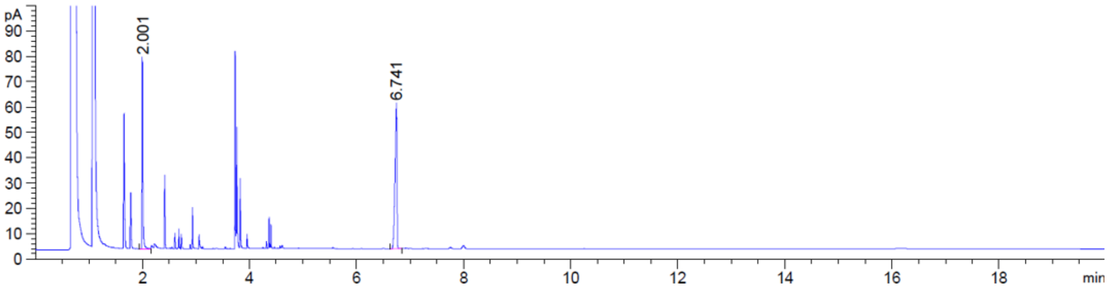

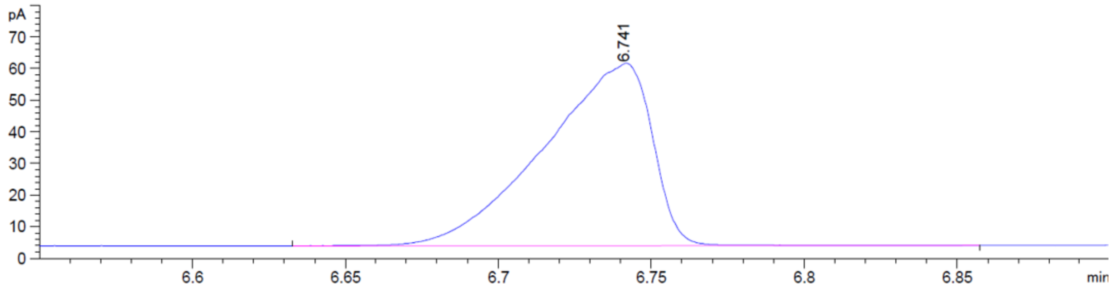

Peak #	Time [min]	Type	Width [min]	Area [pA*s]	Height [pA]	Area %
1	6.741	BB	0.0356	142.18549	56.85785	1.000e2

Supplementary Fig. 25. GC spectra of compound **3w**, Related to Figure 3.

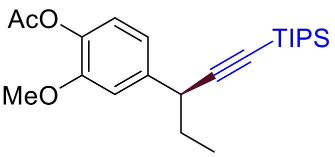

(*S*)-**3i**

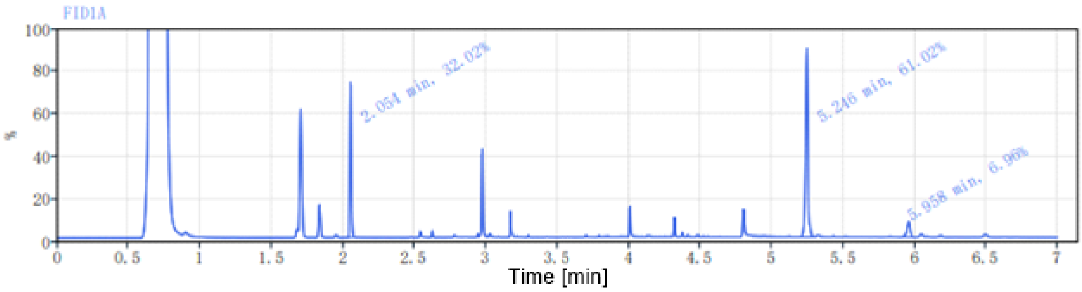

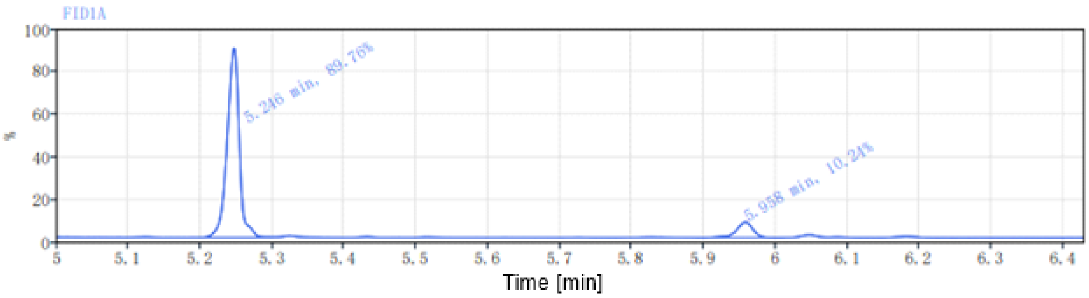

RetTime	Signal	Width (min)	Area	Height	Area%
5.246	FID1A	0.116	37.6	31.7	89.76
5.958	FID1A	0.120	4.3	2.6	10.24

Supplementary Fig. 26. GC spectra of compound (*S*)-**3i**, Related to Figure 5a.

Spectroscopic Data (NMR Spectrum)

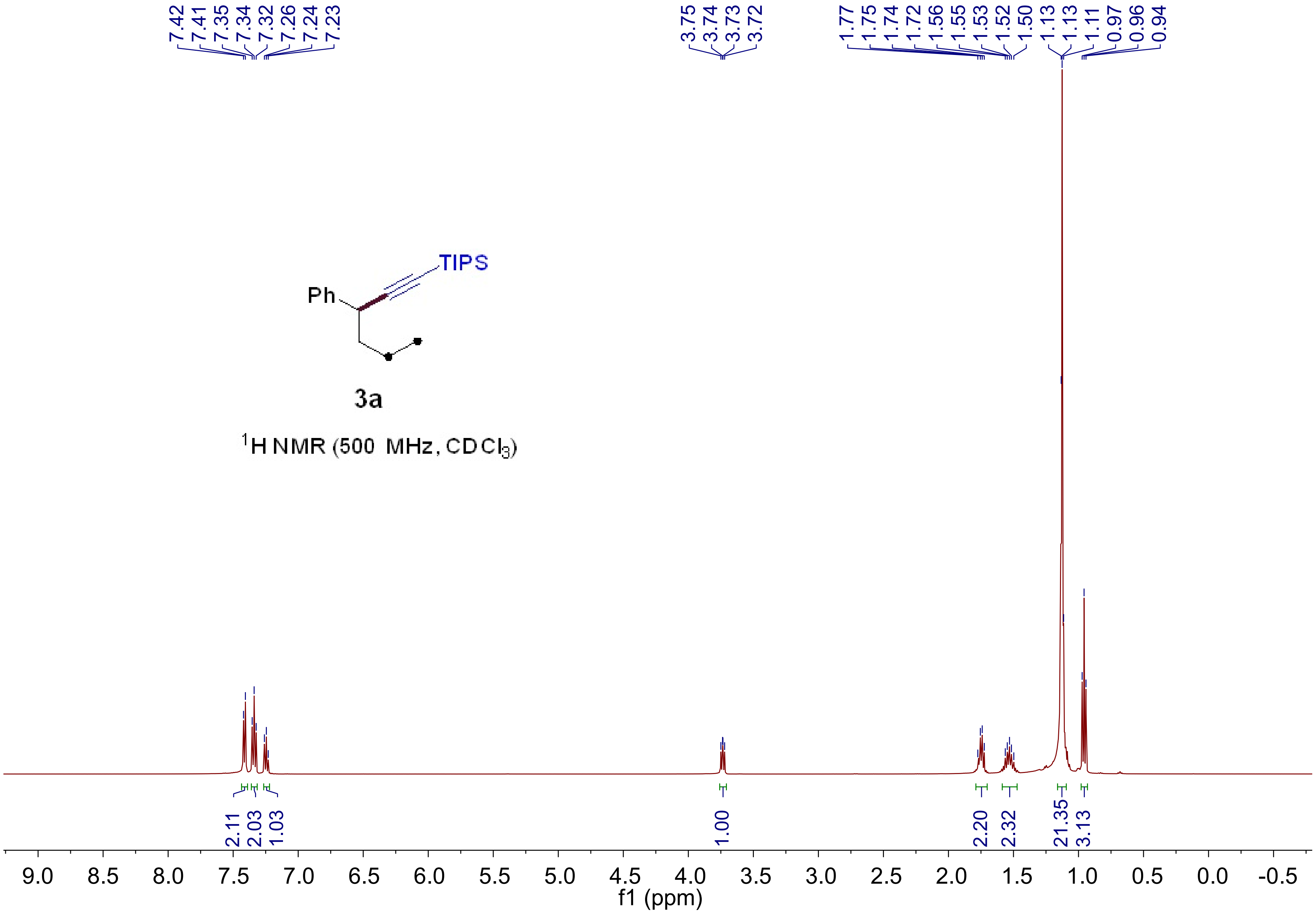

Supplementary Fig. 27. ^{1}H NMR (500 MHz, $CDCl_3$) spectra for compound **3a**

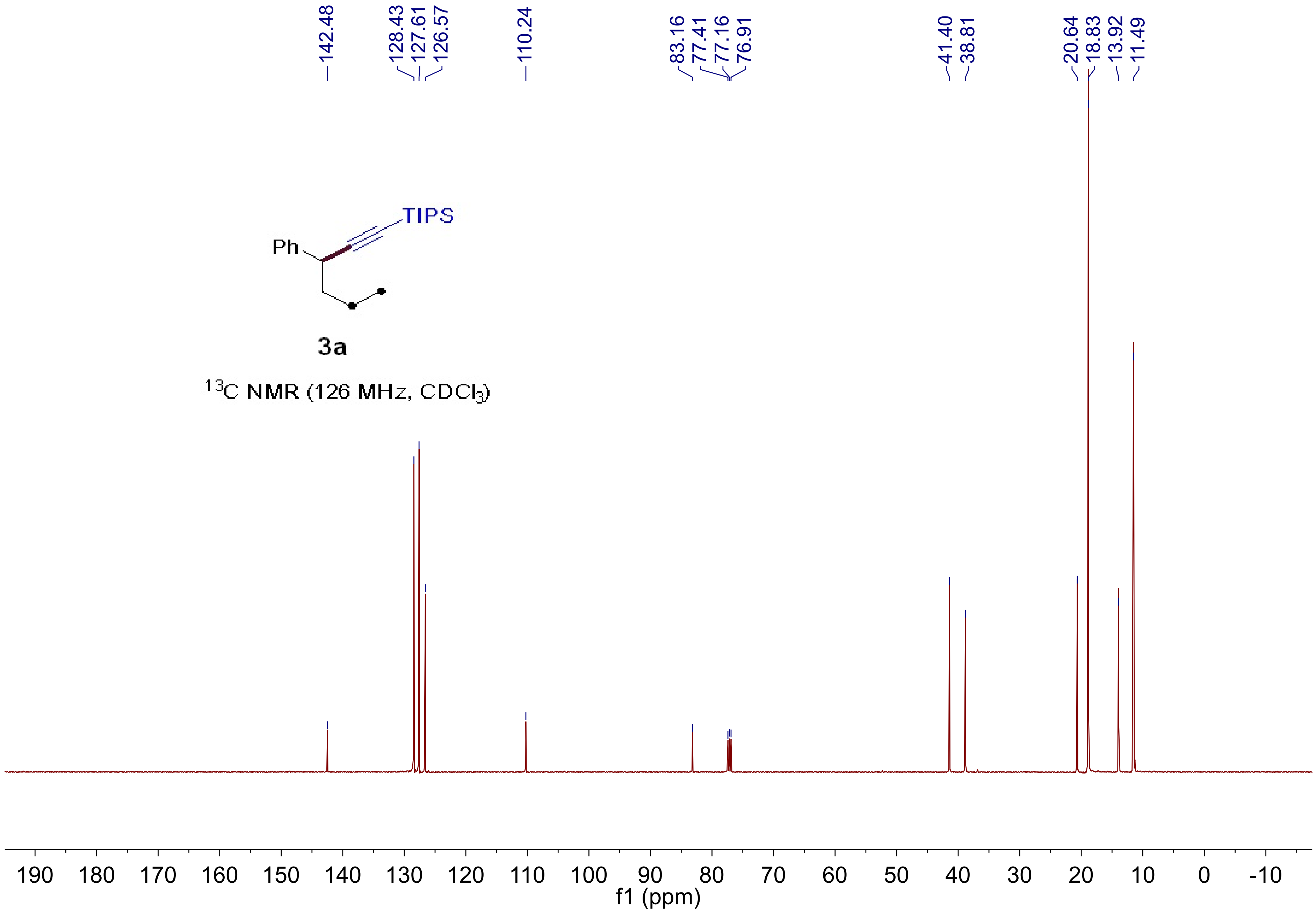

Supplementary Fig. 28. ^{13}C NMR (126 MHz, $CDCl_3$) spectra for compound **3a**

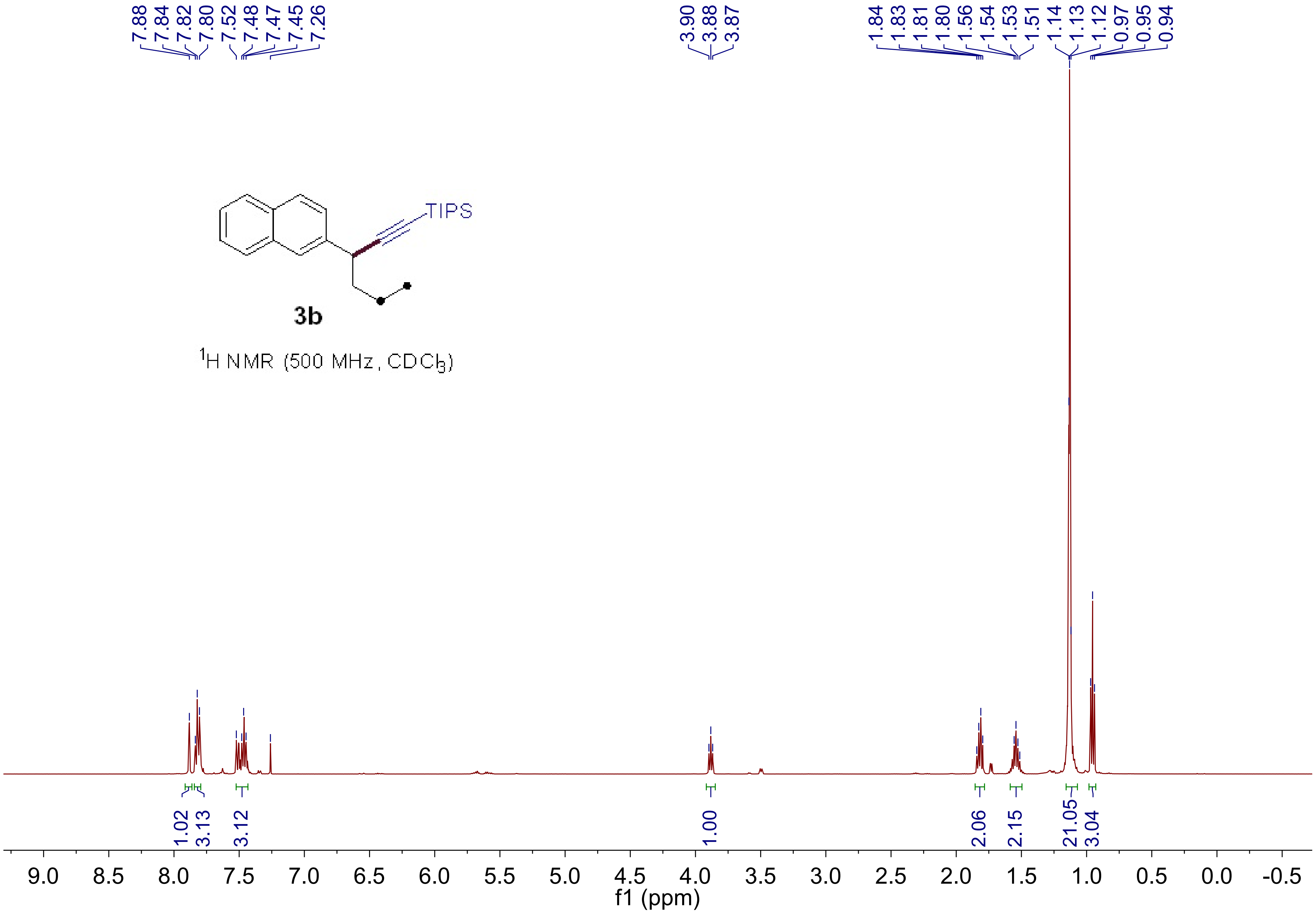

Supplementary Fig. 29. ^{1}H NMR (500 MHz, $CDCl_3$) spectra for compound **3b**

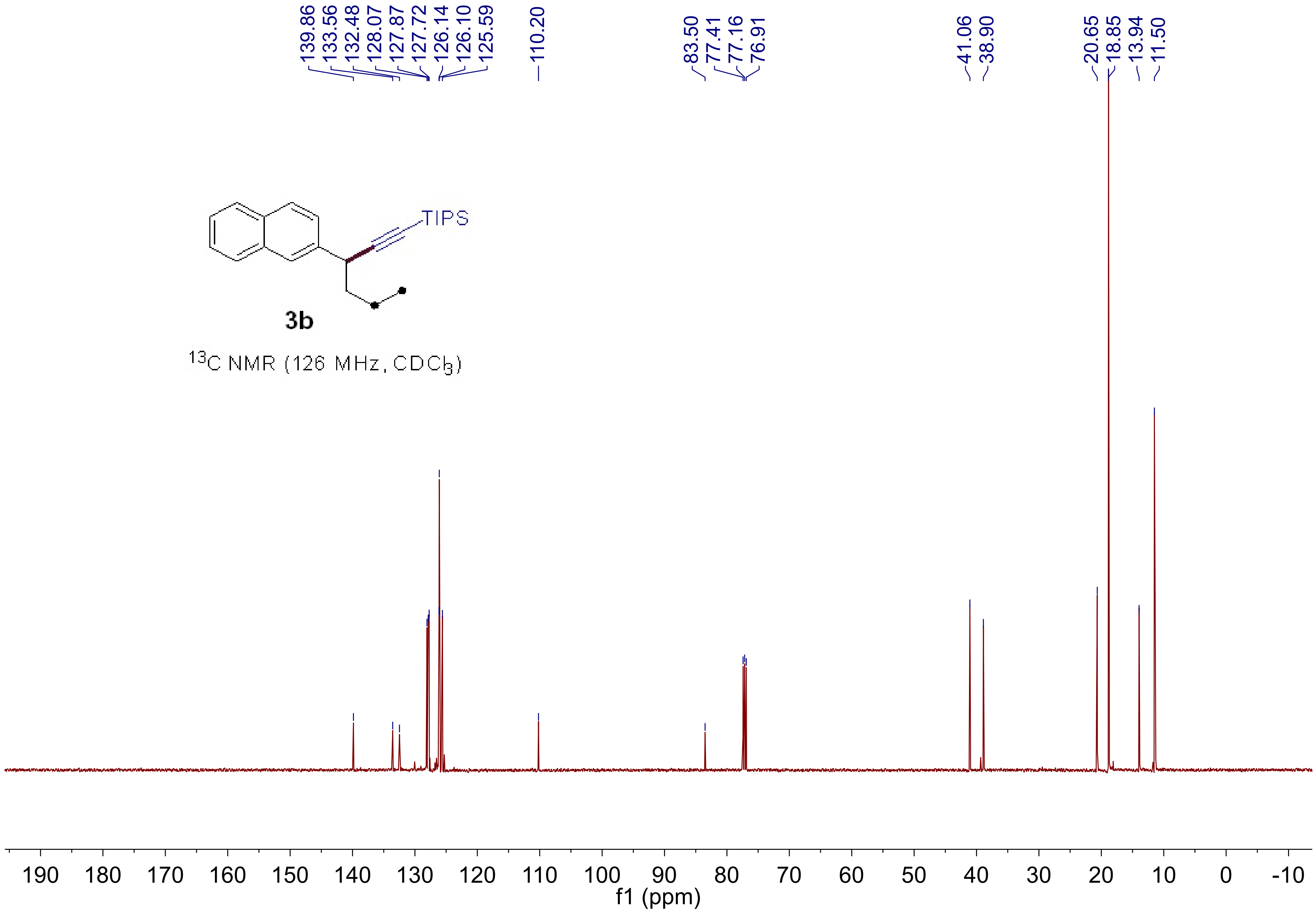

Supplementary Fig. 30. ^{13}C NMR (126 MHz, $CDCl_3$) spectra for compound **3b**

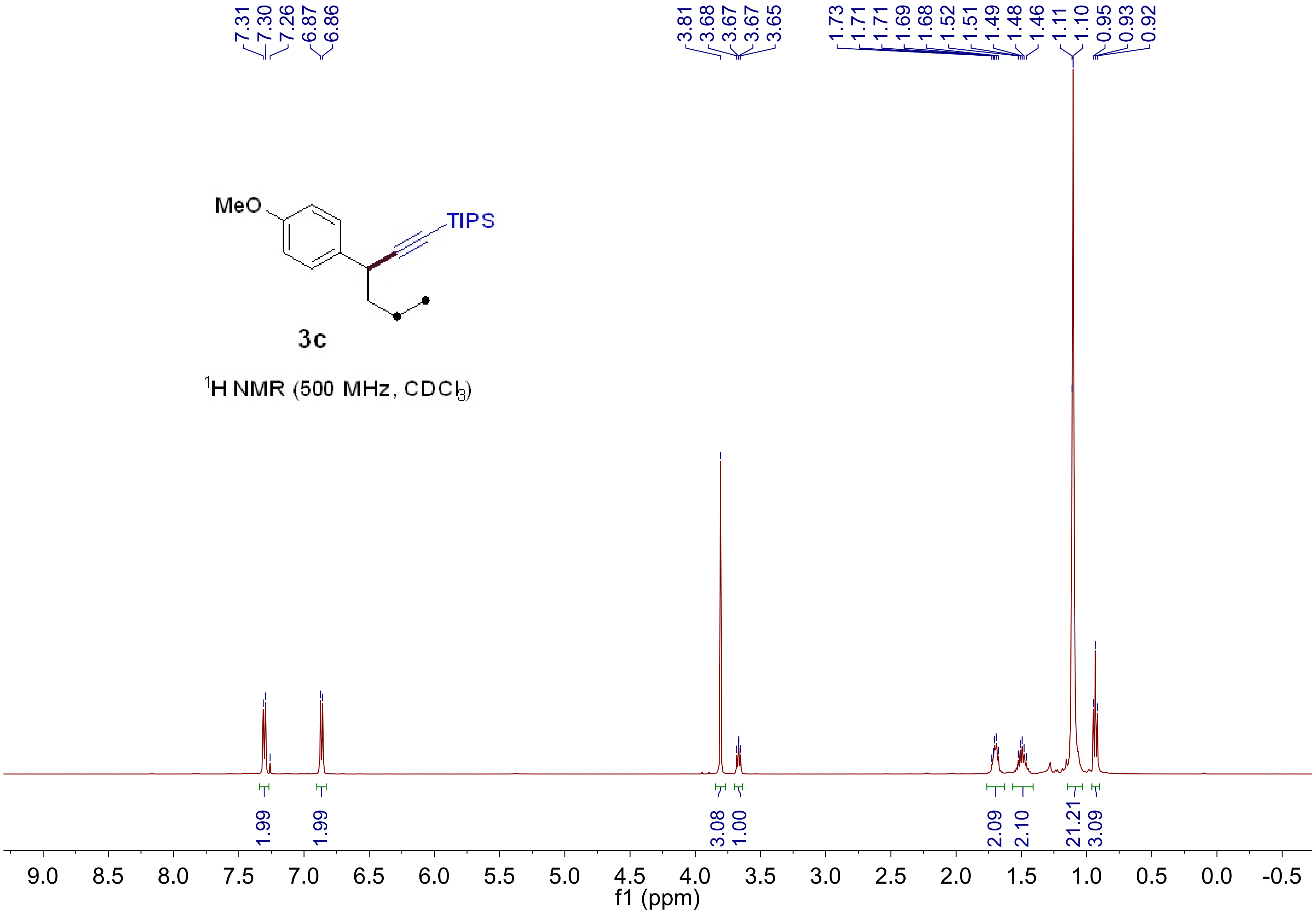

Supplementary Fig. 31. ^{1}H NMR (500 MHz, $CDCl_3$) spectra for compound **3c**

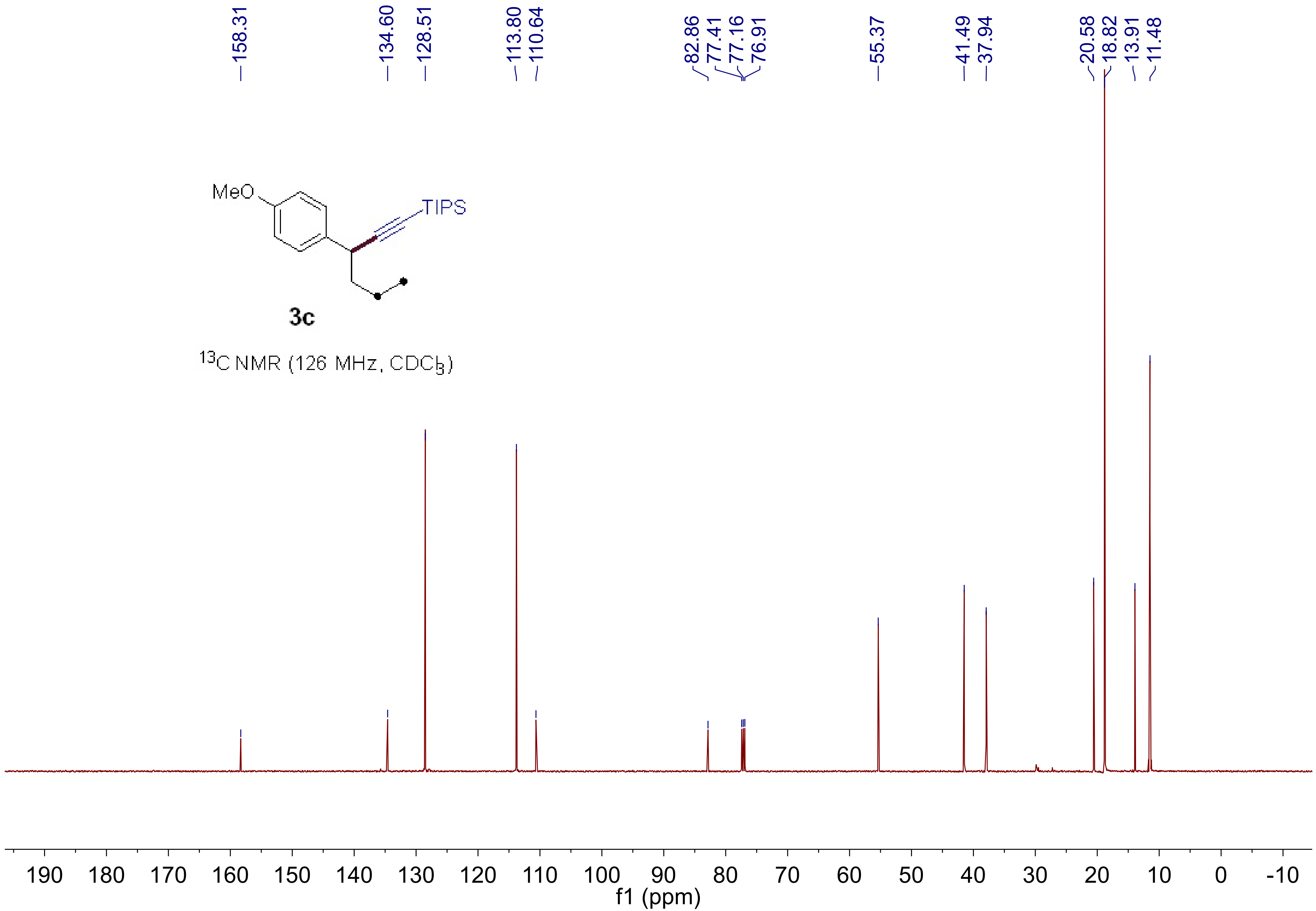

Supplementary Fig. 32. ^{13}C NMR (126 MHz, $CDCl_3$) spectra for compound **3c**

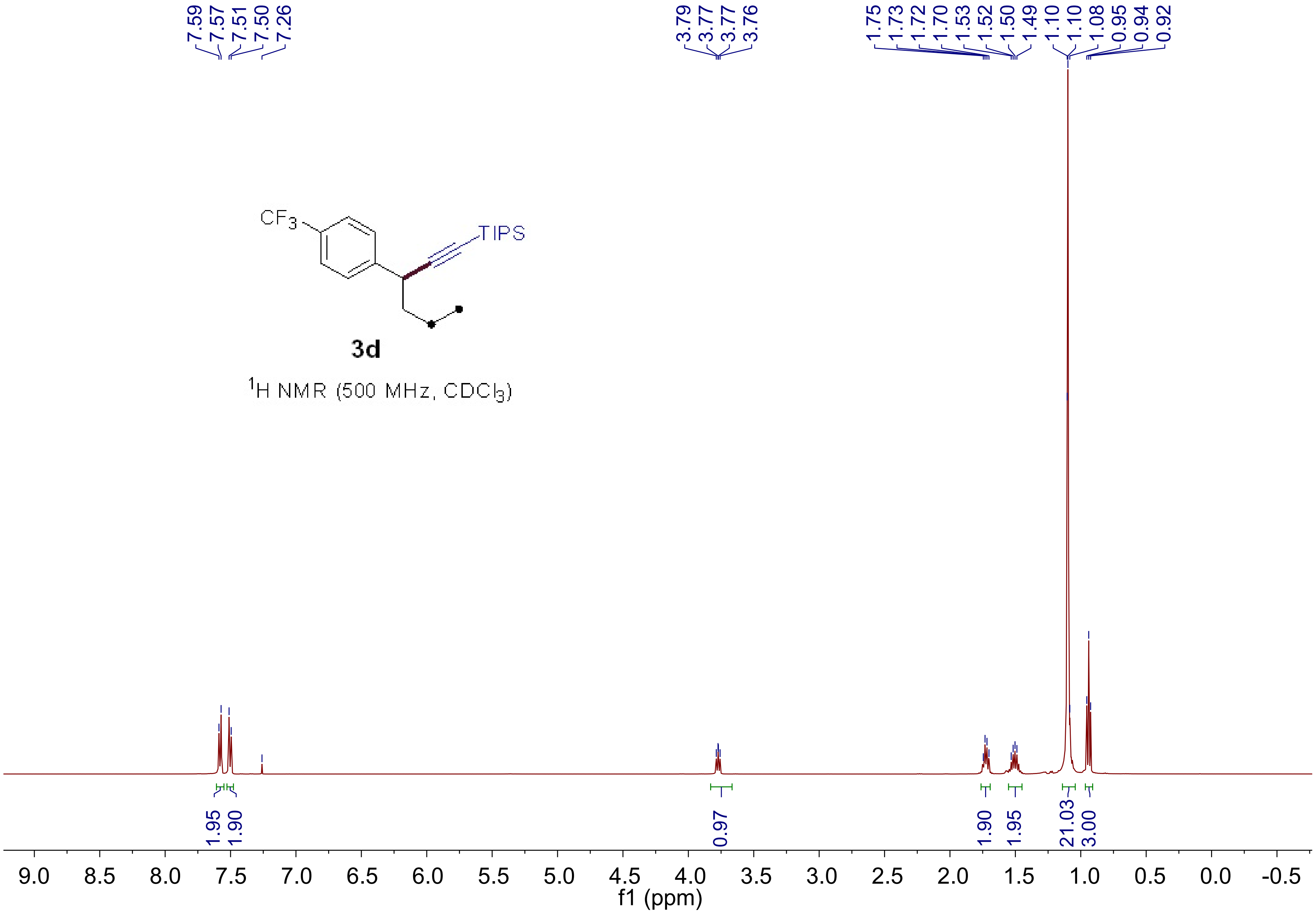

Supplementary Fig. 33. ^{1}H NMR (500 MHz, $CDCl_3$) spectra for compound **3d**

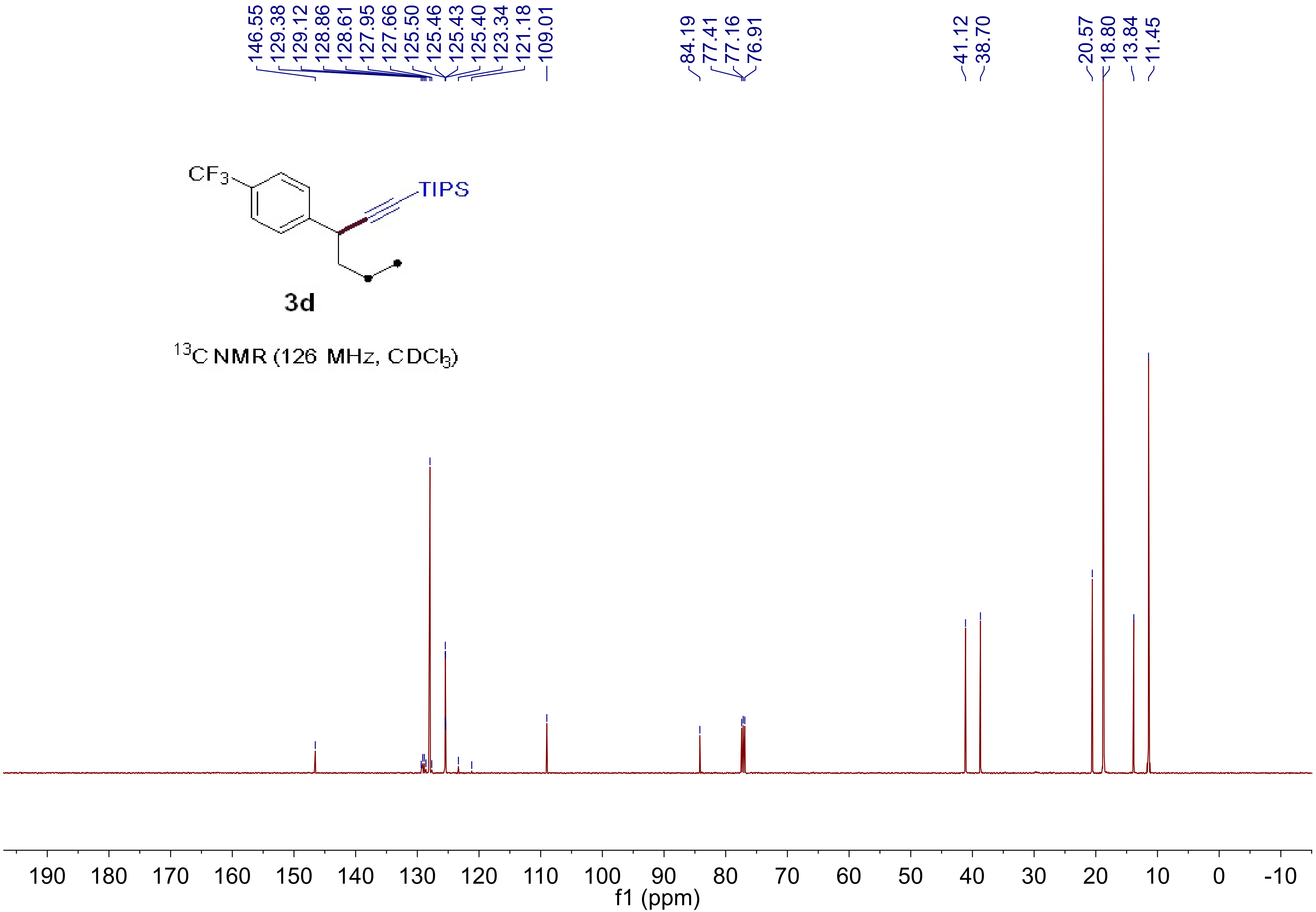

Supplementary Fig. 34. ^{13}C NMR (126 MHz, $CDCl_3$) spectra for compound **3d**

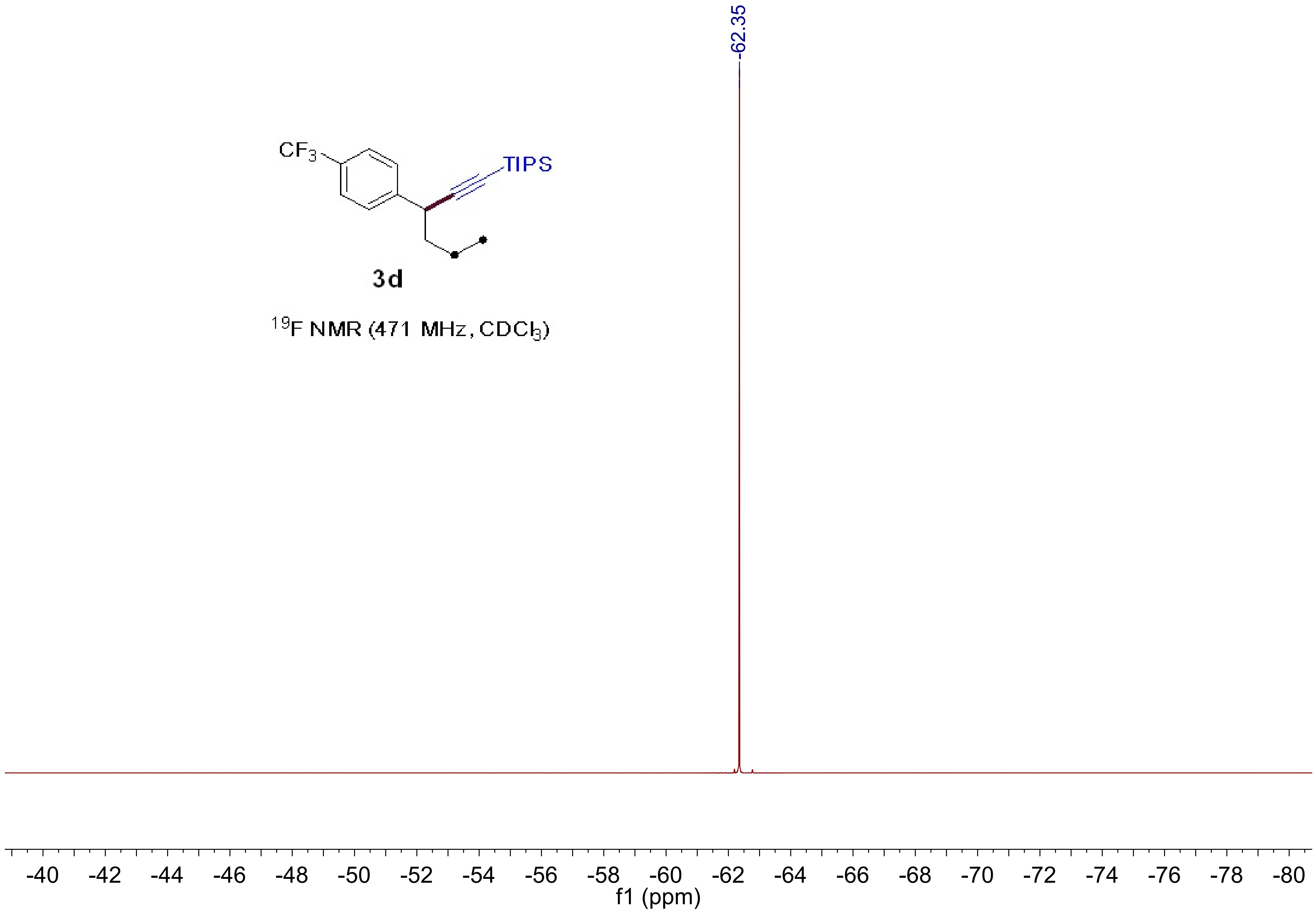

Supplementary Fig. 35. ^{19}F NMR (471 MHz, $CDCl_3$) spectra for compound **3d**

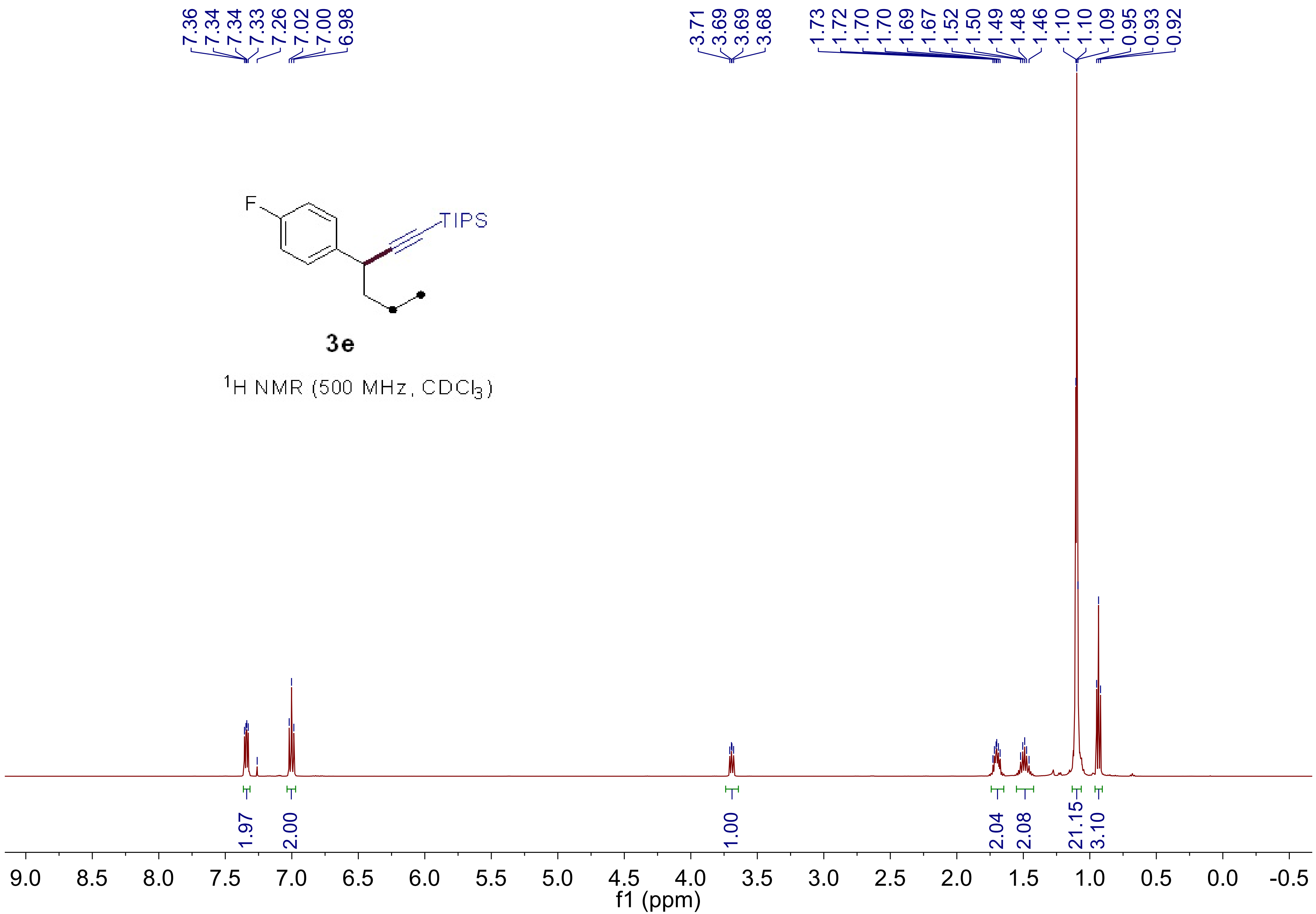

Supplementary Fig. 36. ^{1}H NMR (500 MHz, $CDCl_3$) spectra for compound **3e**

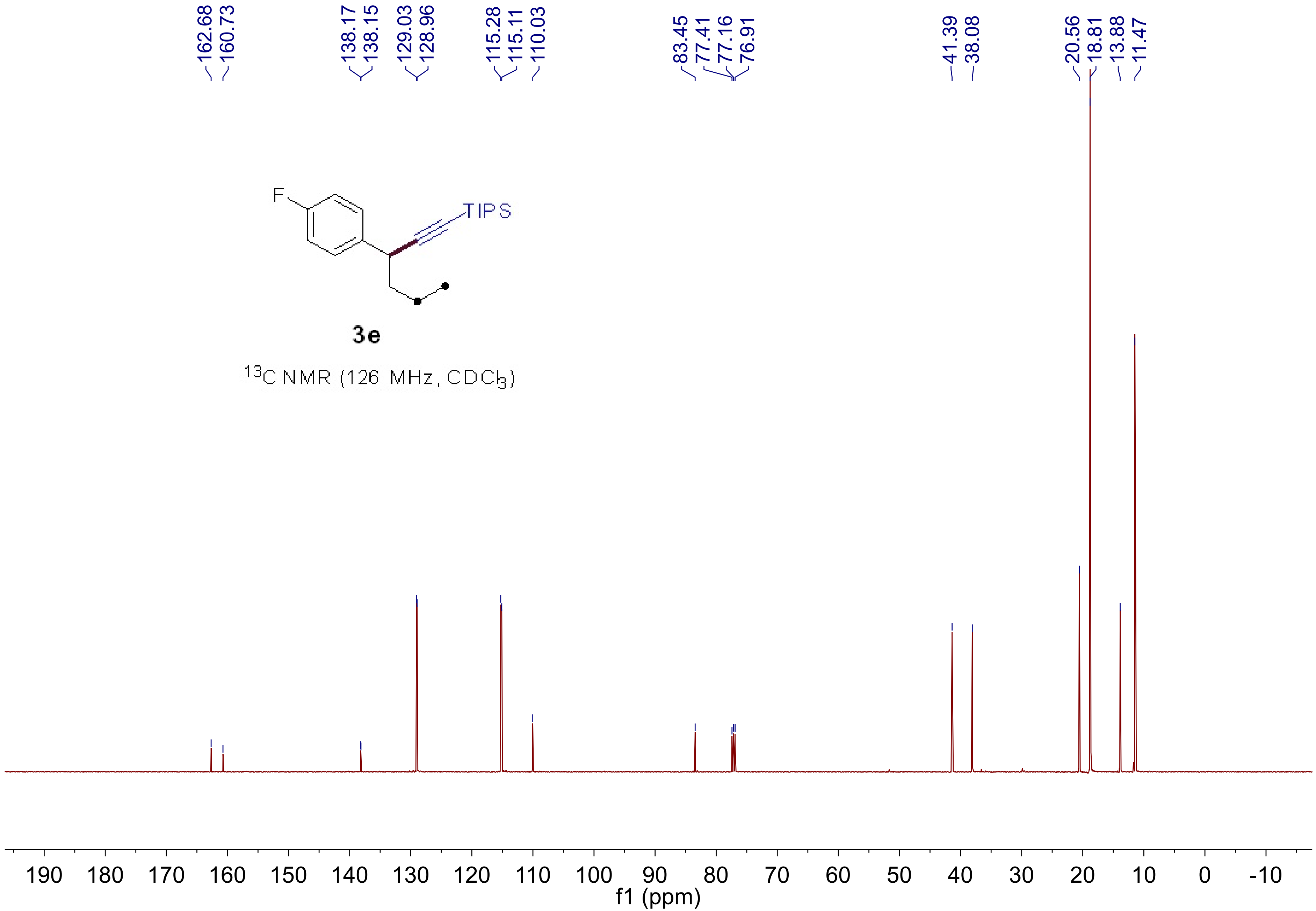

Supplementary Fig. 37. ^{13}C NMR (126 MHz, $CDCl_3$) spectra for compound **3e**

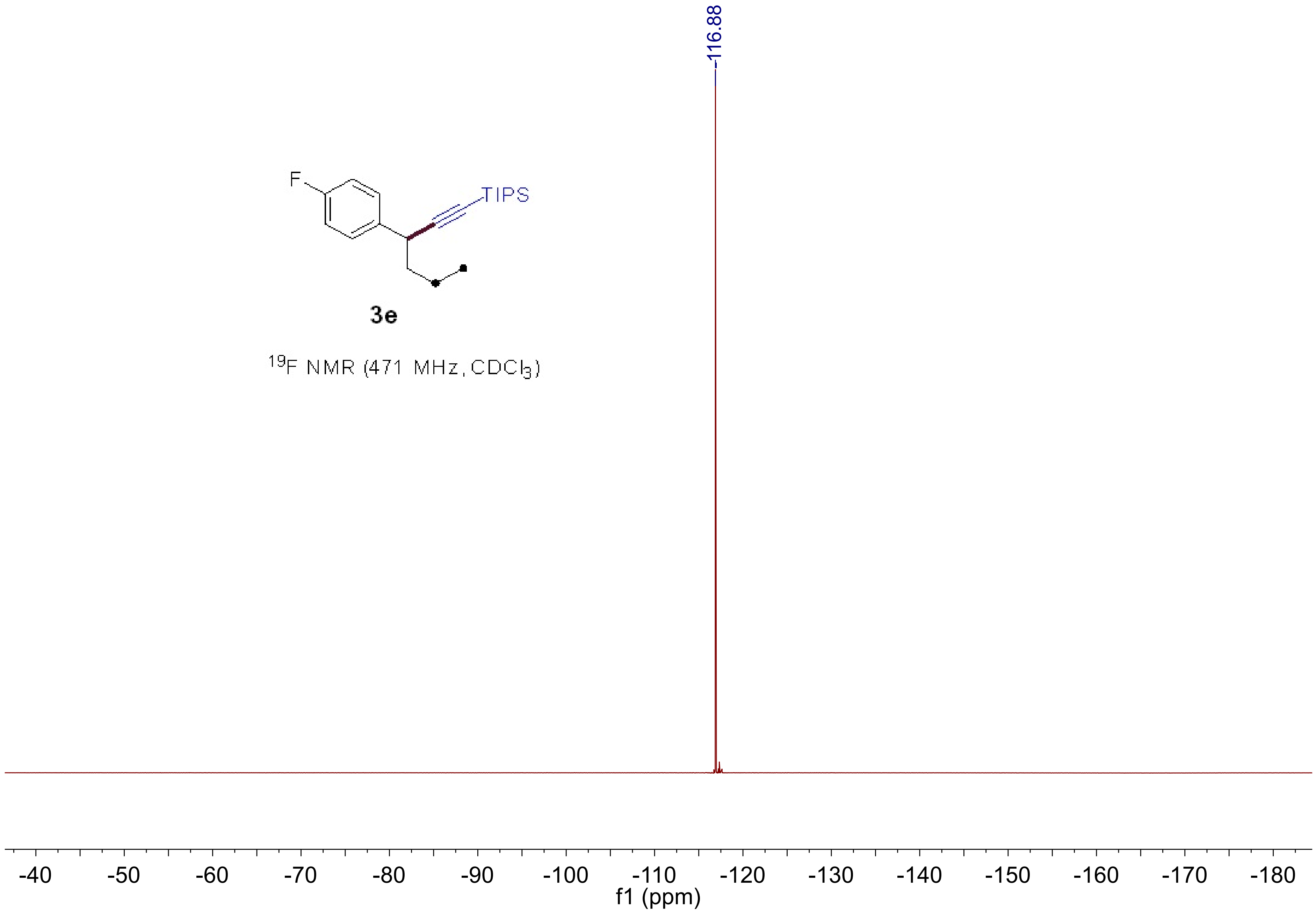

Supplementary Fig. 38. ^{19}F NMR (471 MHz, $CDCl_3$) spectra for compound **3e**

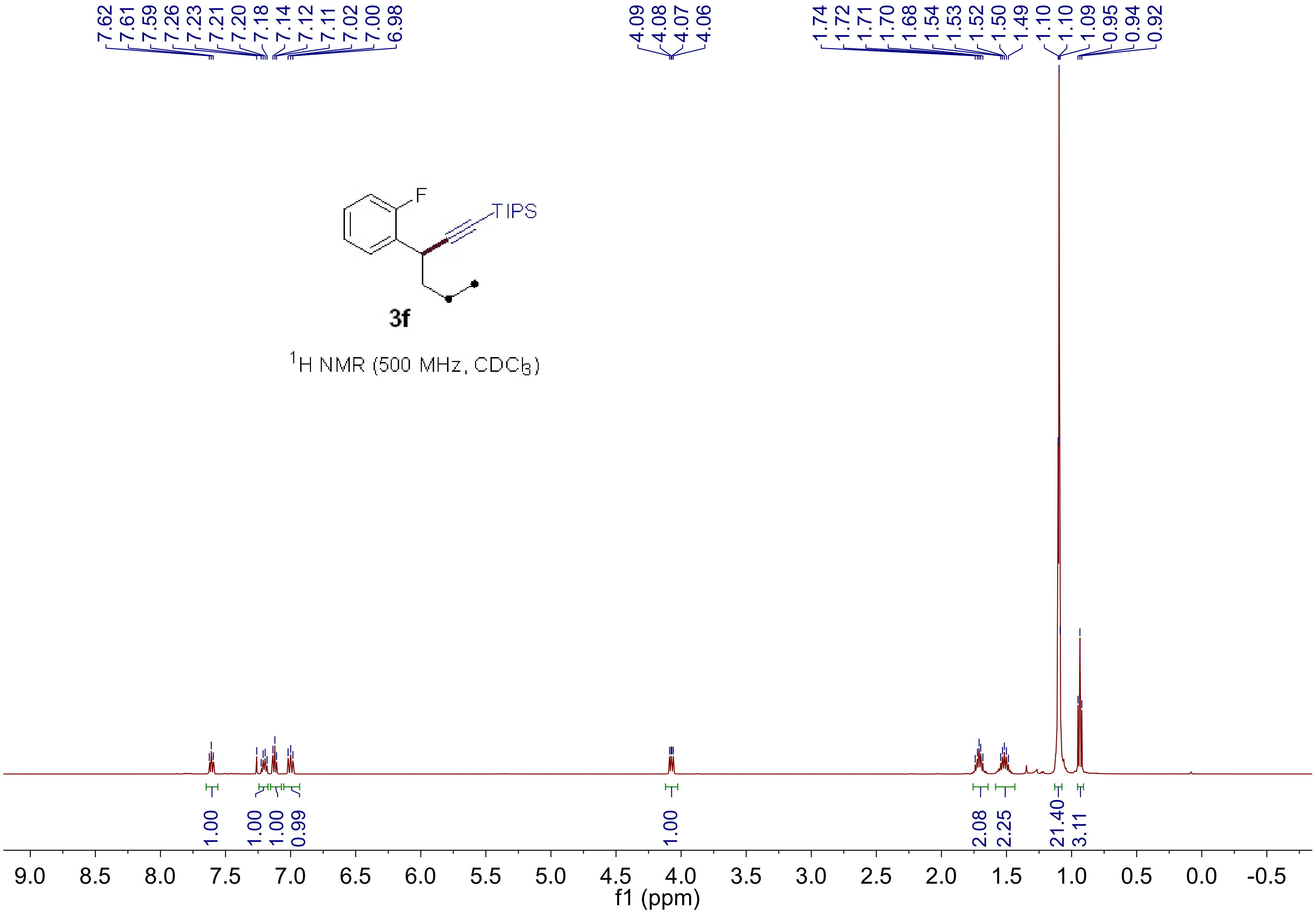

Supplementary Fig. 39. ^{1}H NMR (500 MHz, $CDCl_3$) spectra for compound **3f**

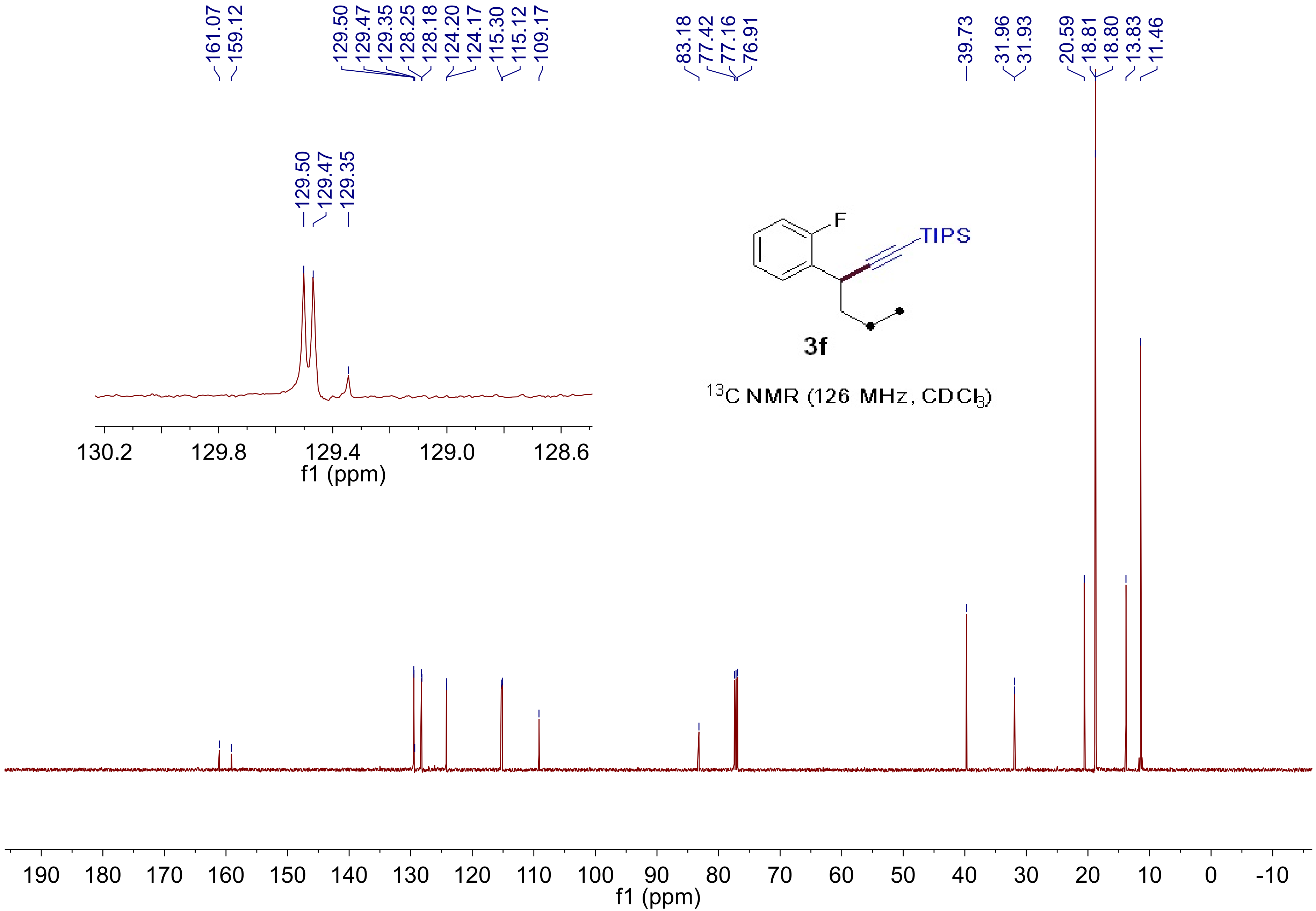

Supplementary Fig. 40. ^{13}C NMR (126 MHz, $CDCl_3$) spectra for compound **3f**

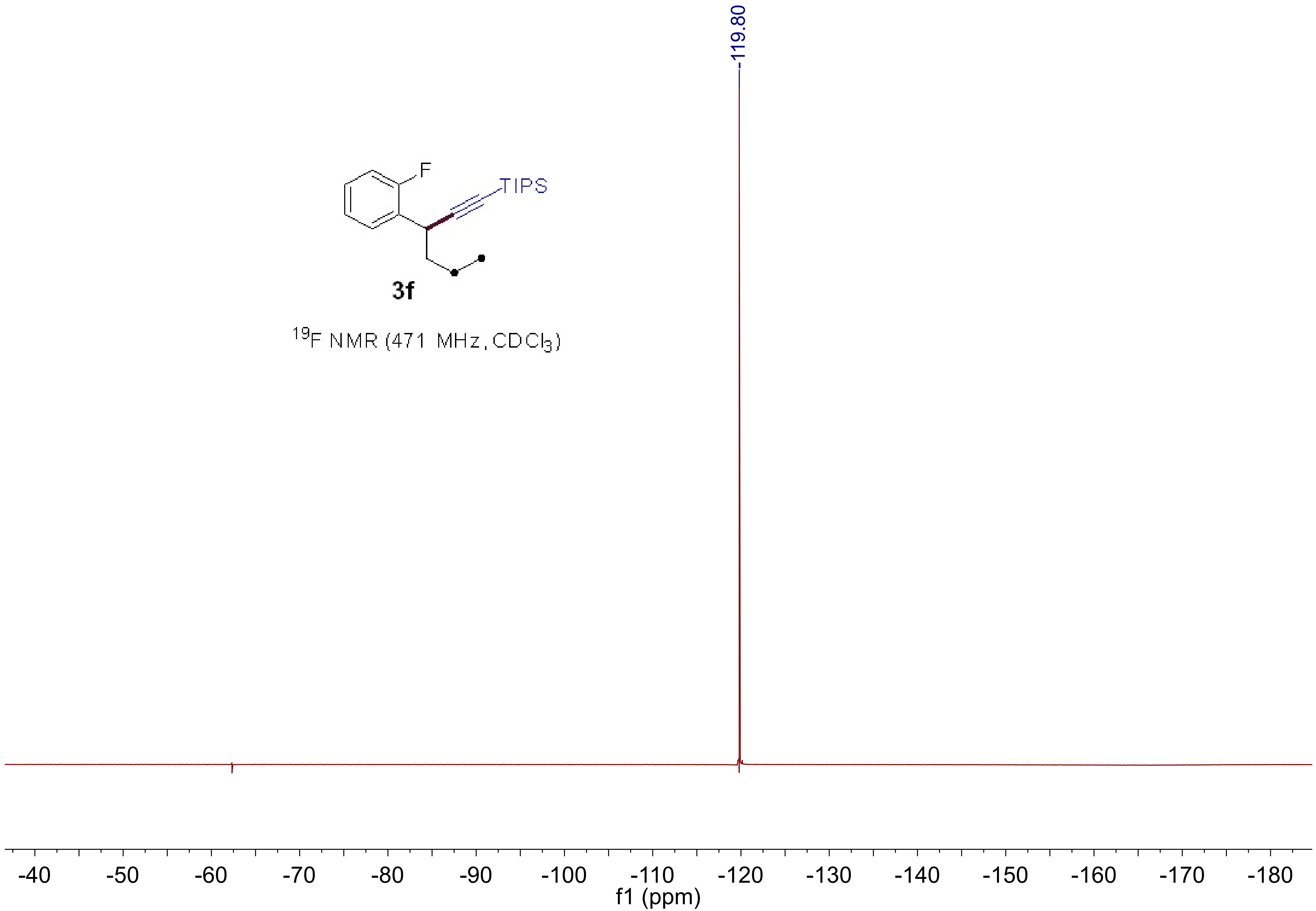

Supplementary Fig. 41. ^{19}F NMR (471 MHz, $CDCl_3$) spectra for compound **3f**

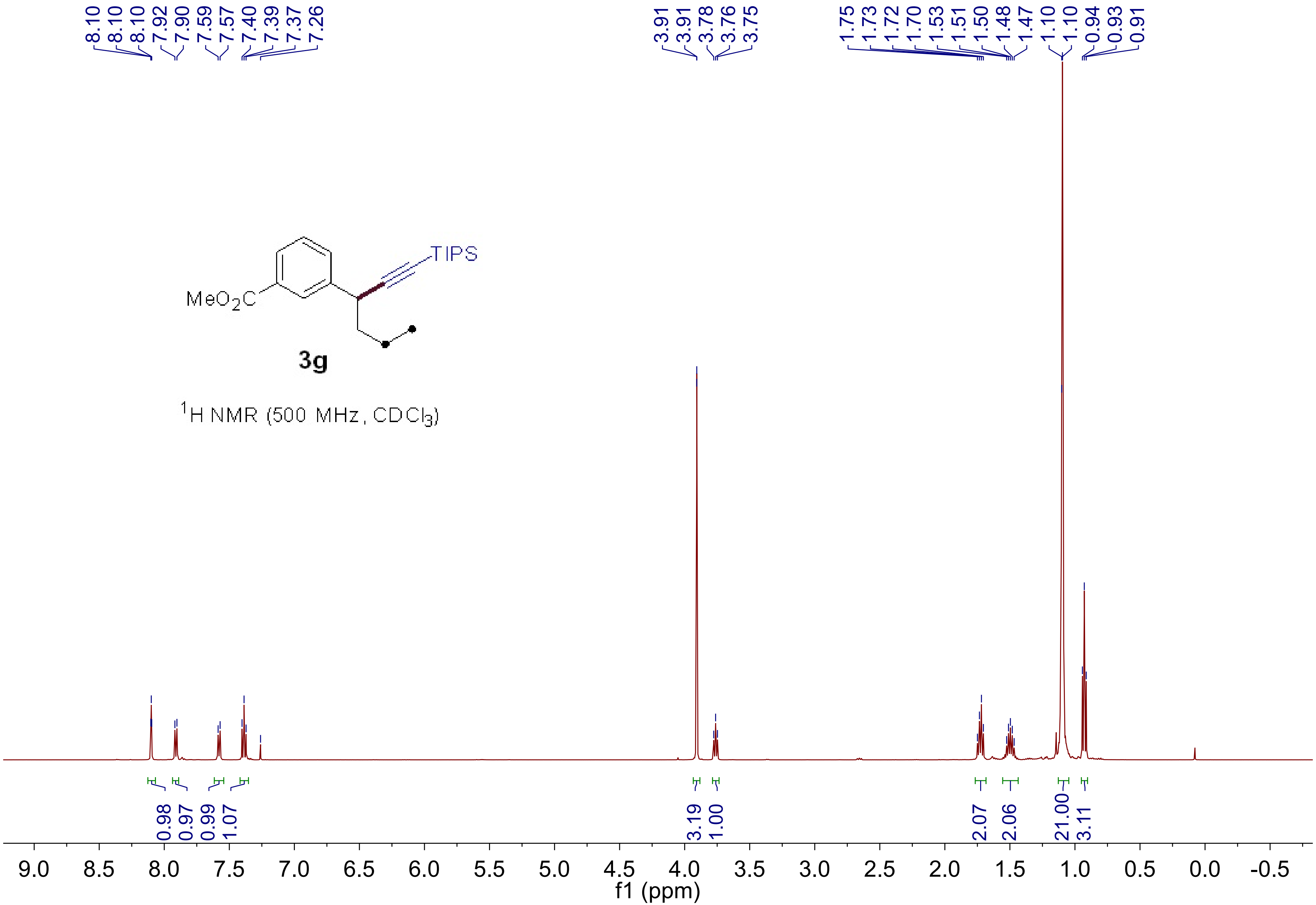

Supplementary Fig. 42. ^{1}H NMR (500 MHz, $CDCl_3$) spectra for compound **3g**

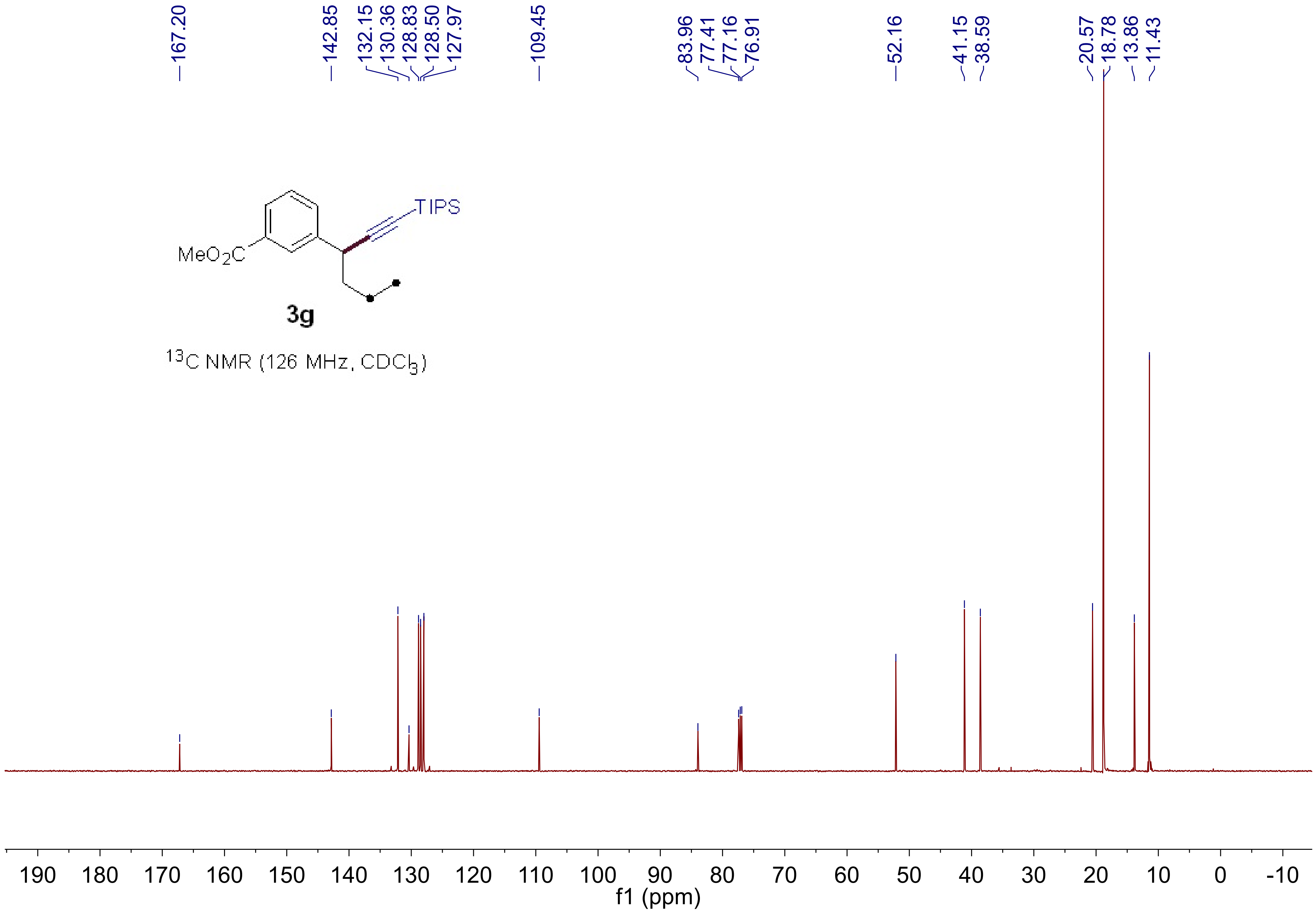

Supplementary Fig. 43. ^{13}C NMR (126 MHz, $CDCl_3$) spectra for compound **3g**

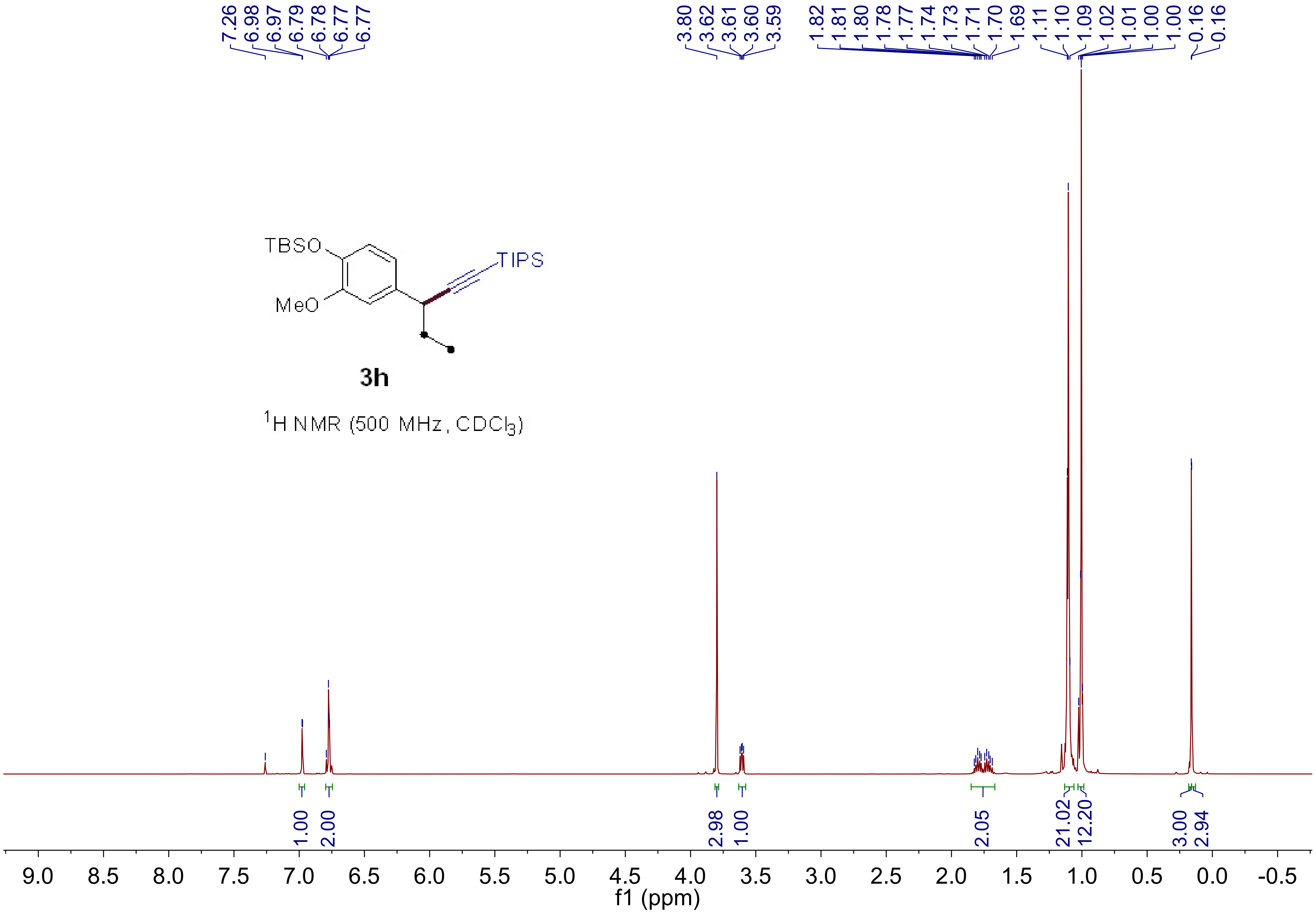

Supplementary Fig. 44. ^{1}H NMR (500 MHz, $CDCl_3$) spectra for compound **3h**

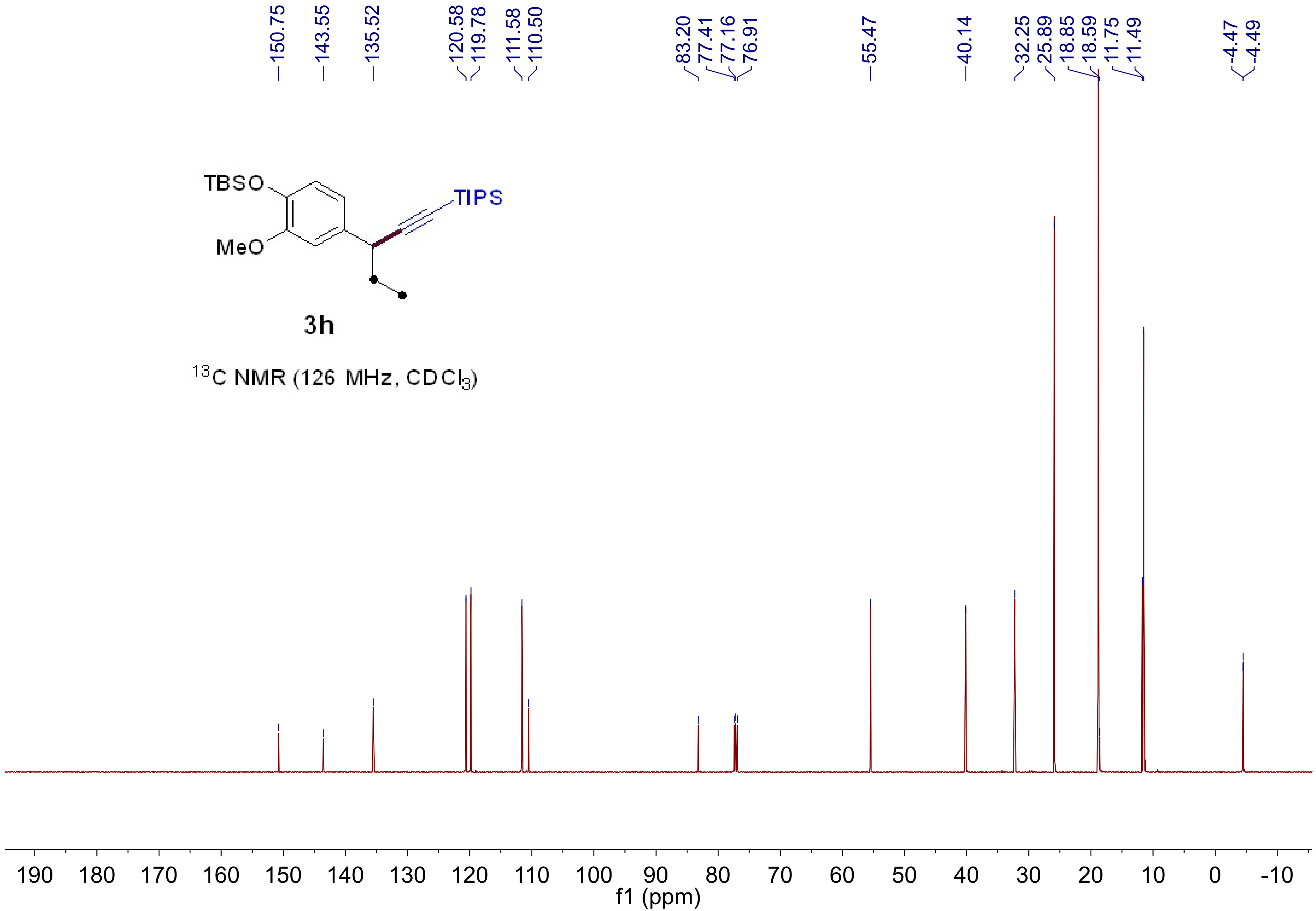

Supplementary Fig. 45. ^{13}C NMR (126 MHz, $CDCl_3$) spectra for compound **3h**

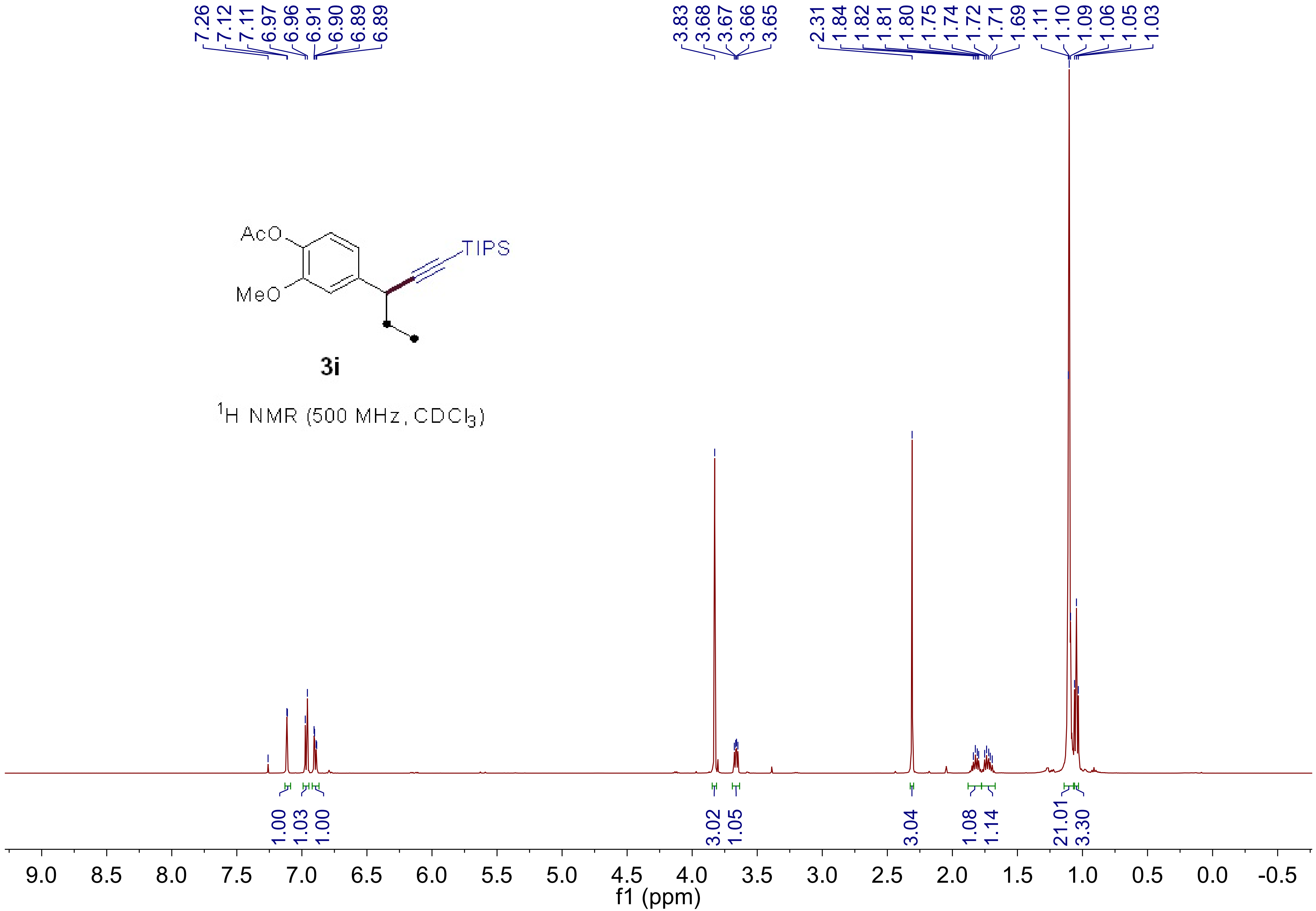

Supplementary Fig. 46. ^{1}H NMR (500 MHz, $CDCl_3$) spectra for compound **3i**

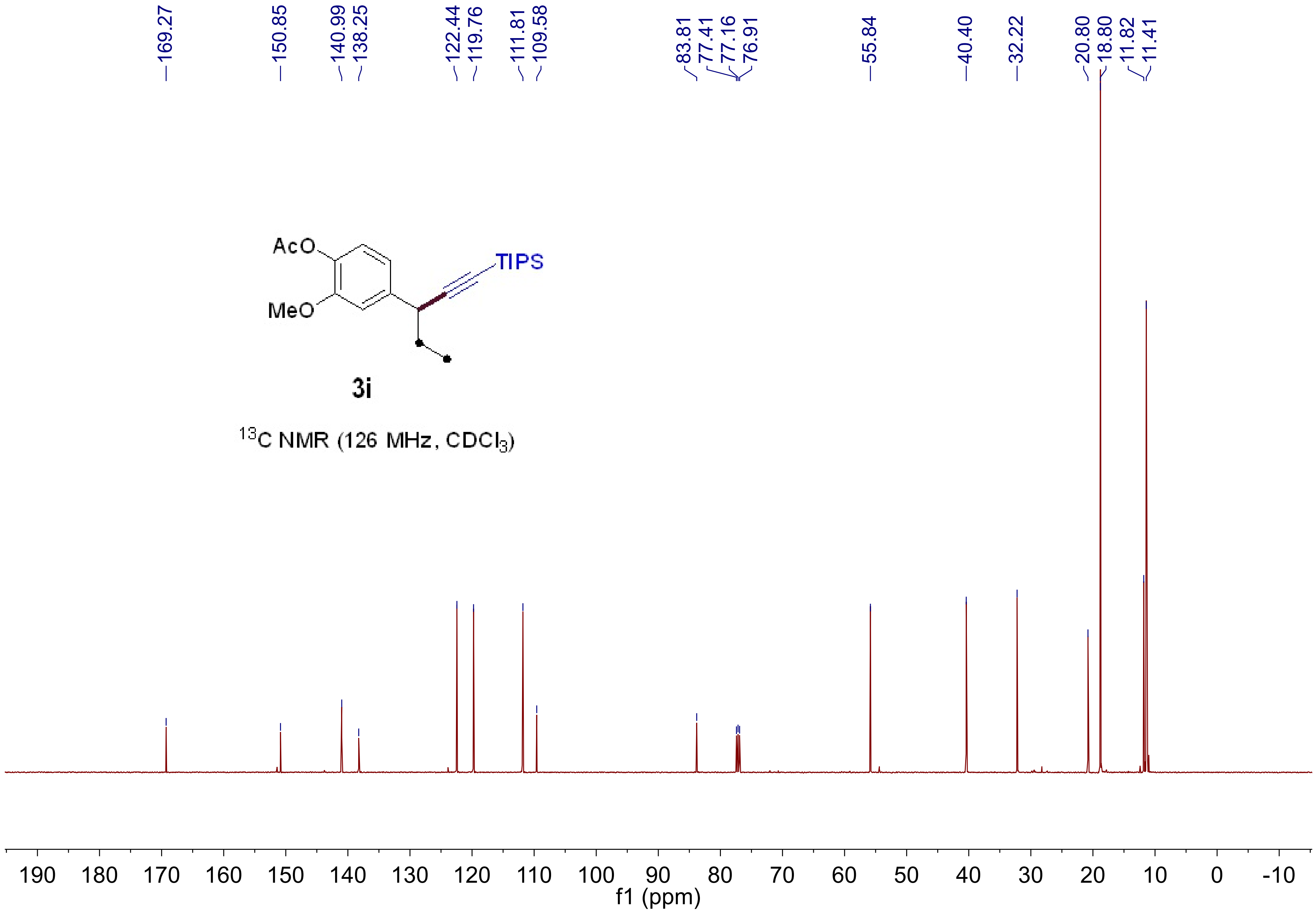

Supplementary Fig. 47. ^{13}C NMR (126 MHz, $CDCl_3$) spectra for compound **3i**

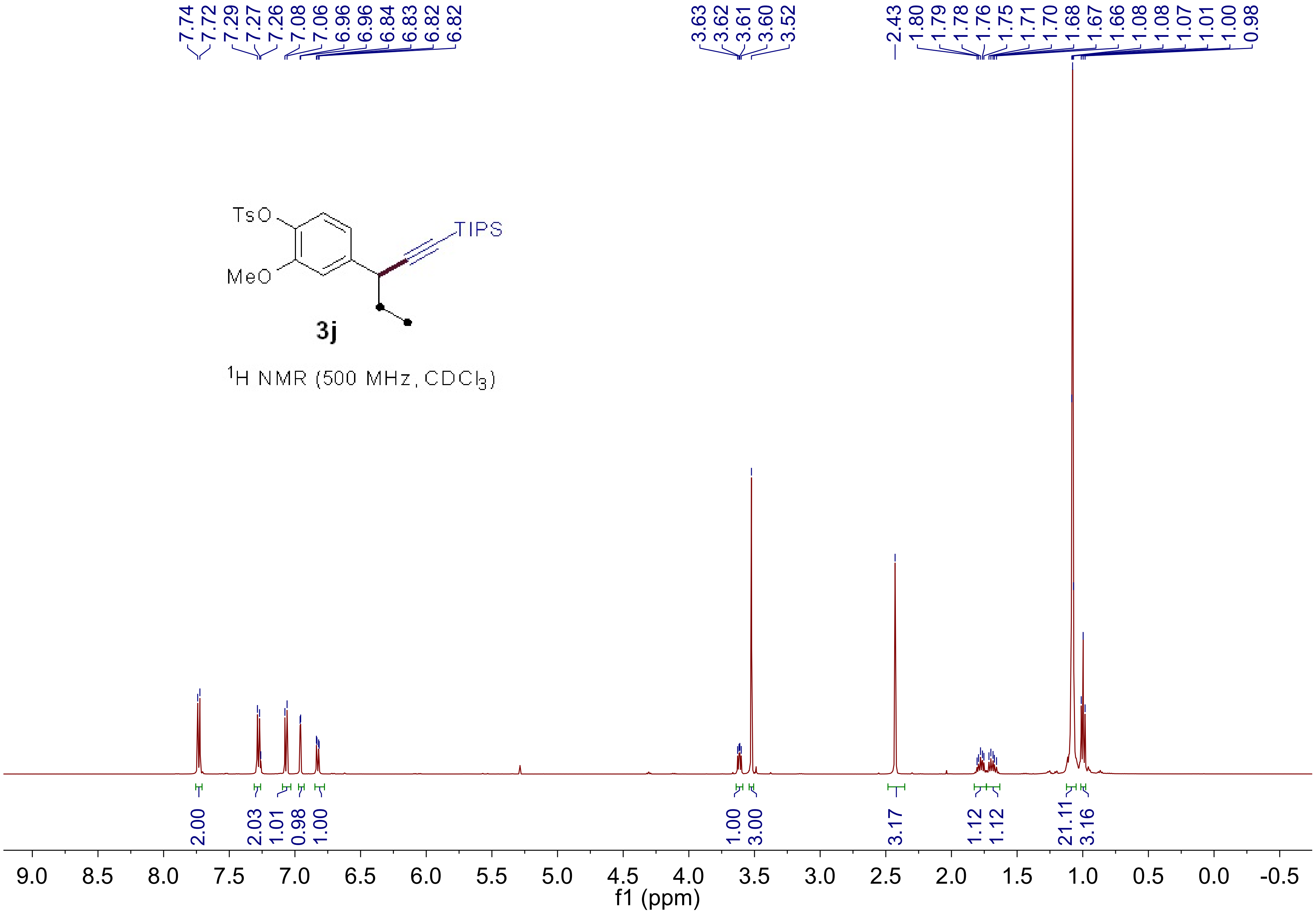

Supplementary Fig. 48. ^{1}H NMR (500 MHz, $CDCl_3$) spectra for compound **3j**

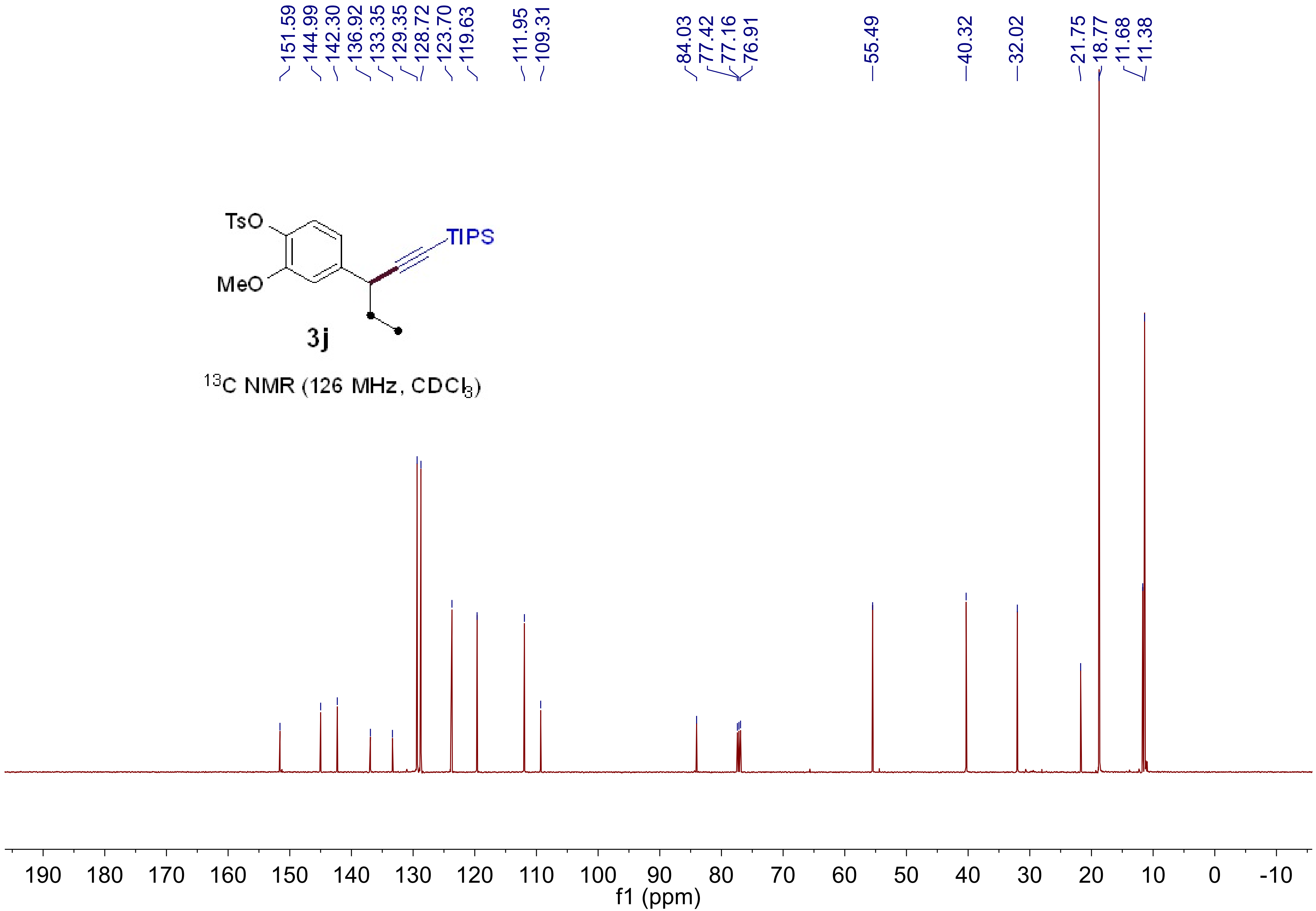

Supplementary Fig. 49. ^{13}C NMR (126 MHz, $CDCl_3$) spectra for compound 3j

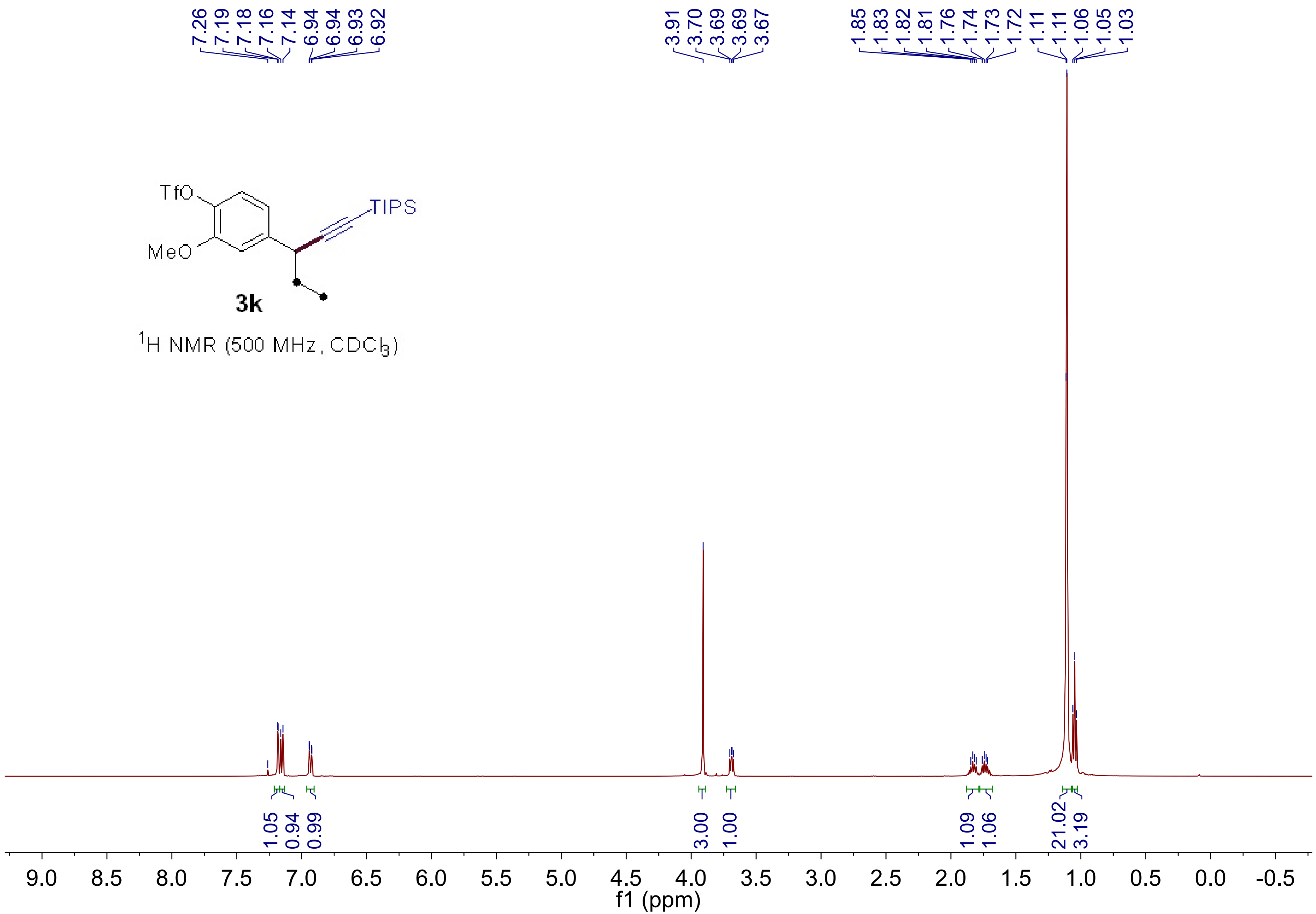

Supplementary Fig. 50. ^{1}H NMR (500 MHz, $CDCl_3$) spectra for compound **3k**

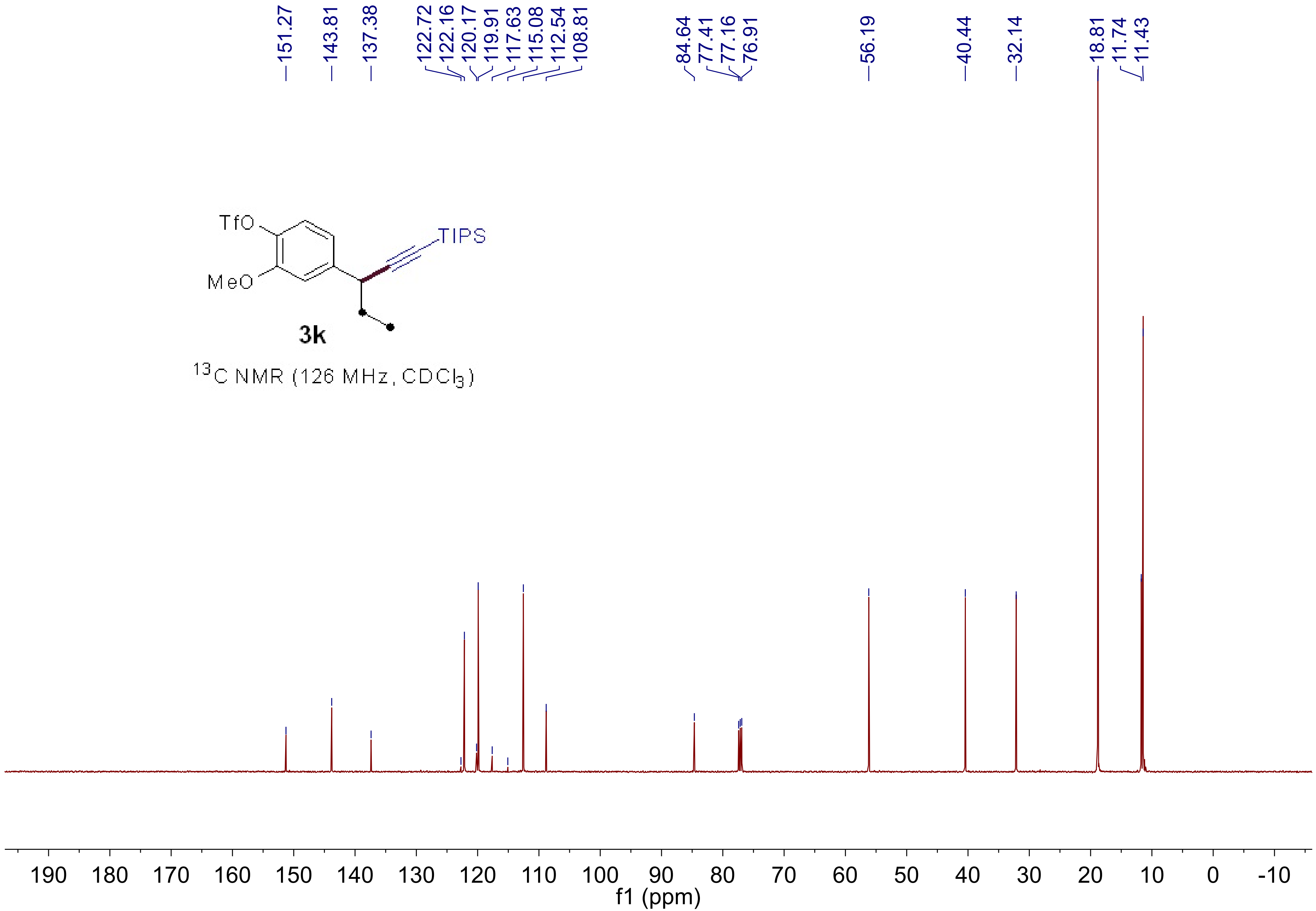

Supplementary Fig. 51. ^{13}C NMR (126 MHz, $CDCl_3$) spectra for compound **3k**

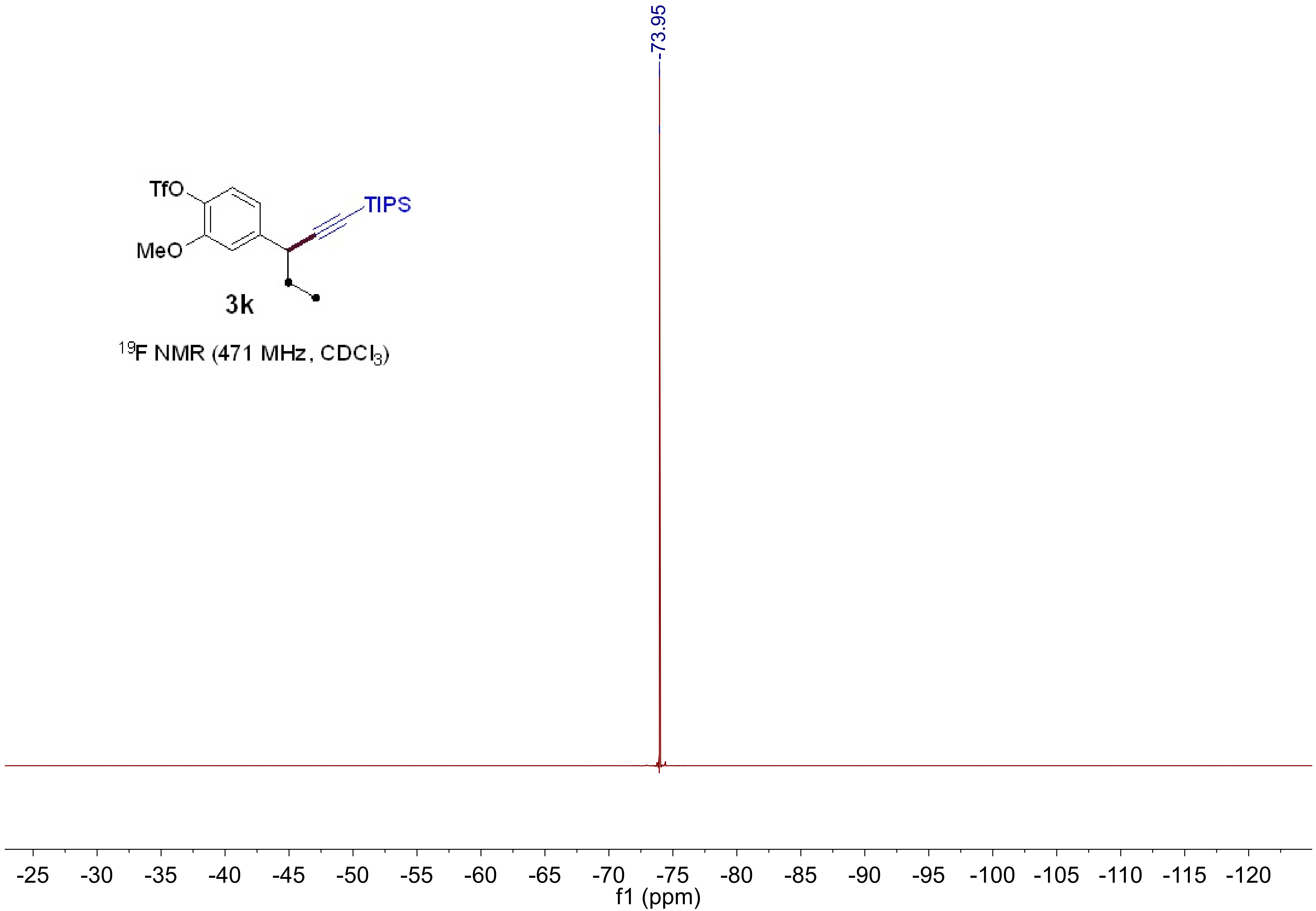

Supplementary Fig. 52. ^{19}F NMR (471 MHz, $CDCl_3$) spectra for compound **3k**

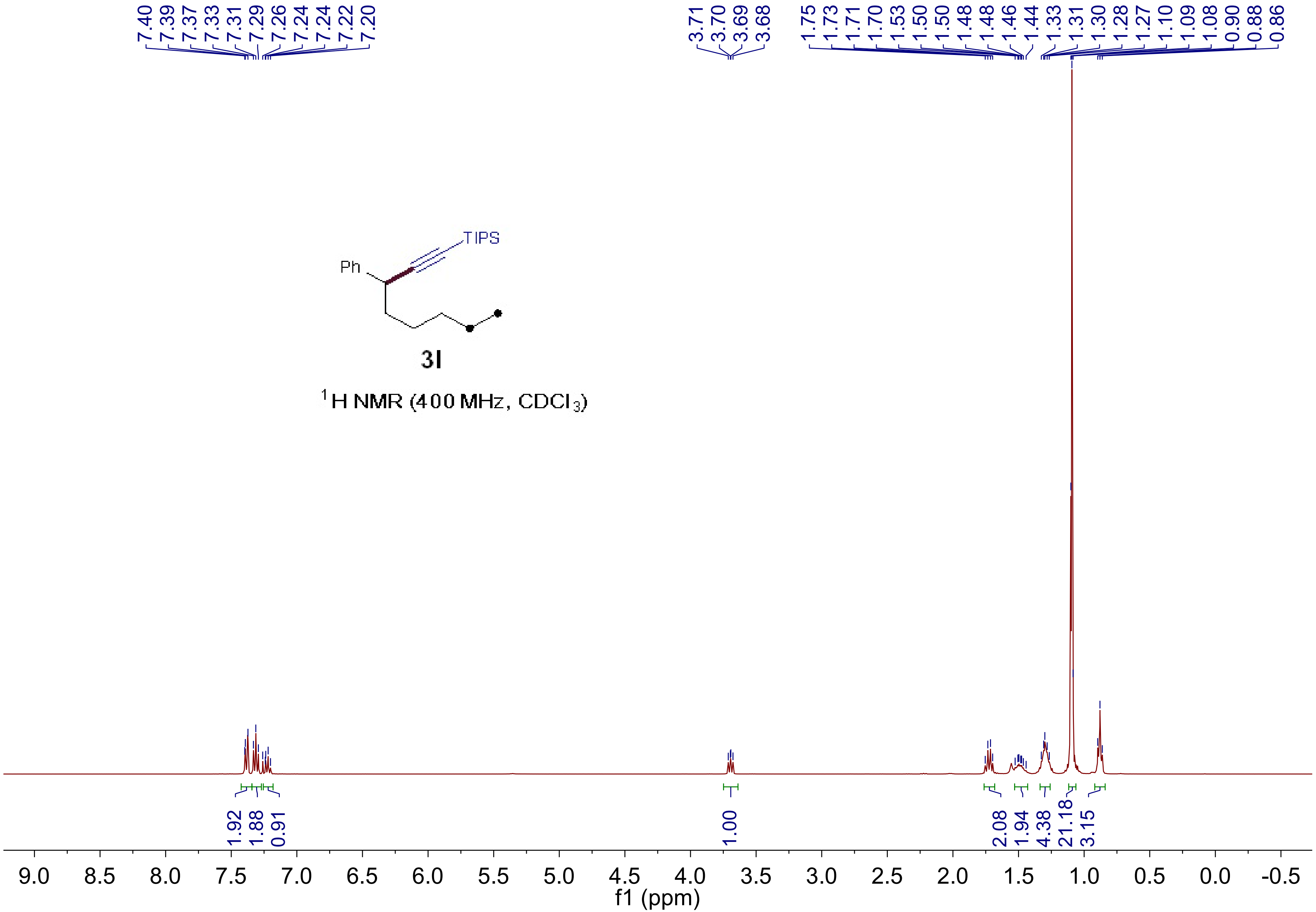

Supplementary Fig. 53. ^{1}H NMR (400 MHz, $CDCl_3$) spectra for compound **3l**

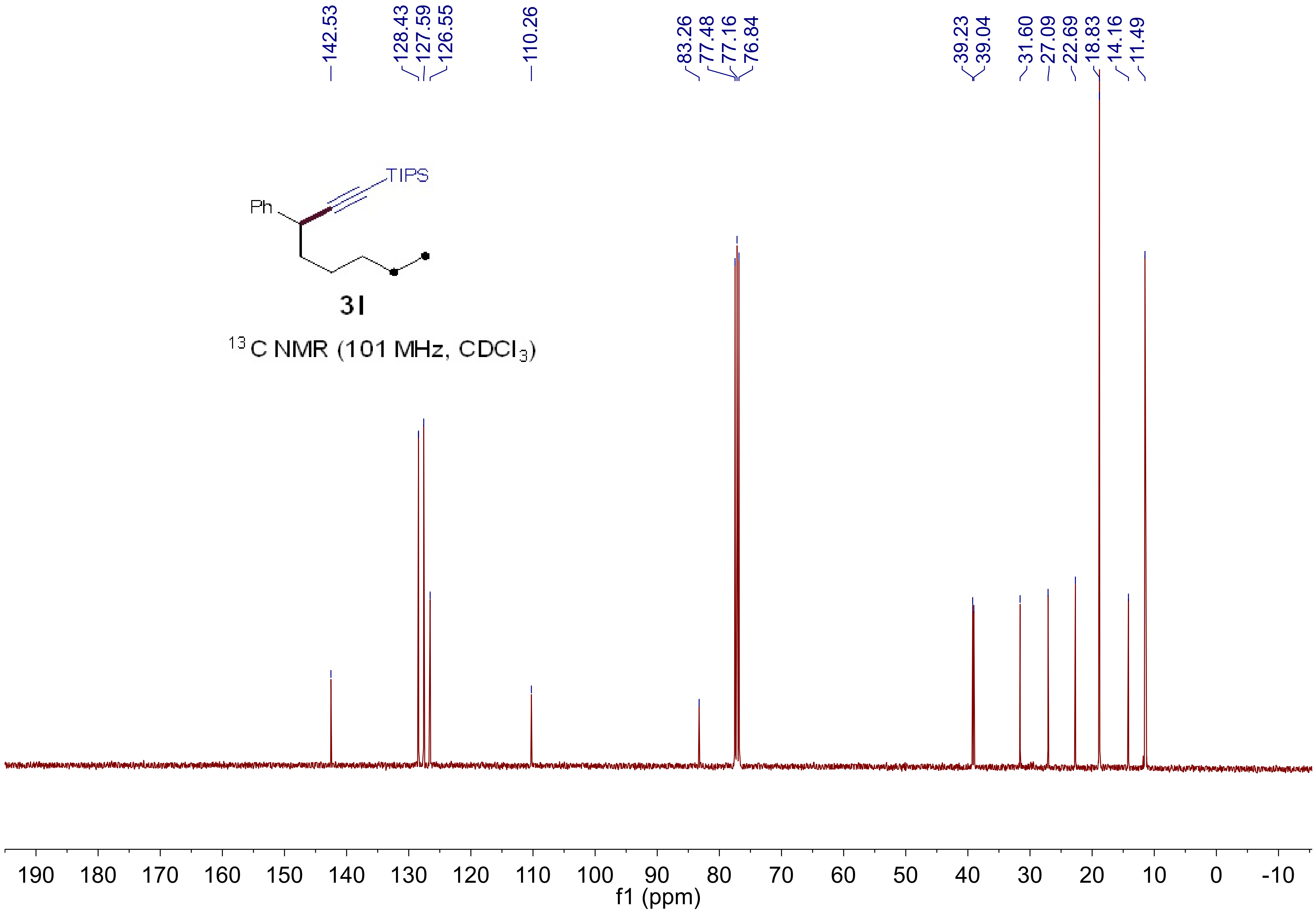

Supplementary Fig. 54. ^{13}C NMR (101 MHz, $CDCl_3$) spectra for compound **3l**

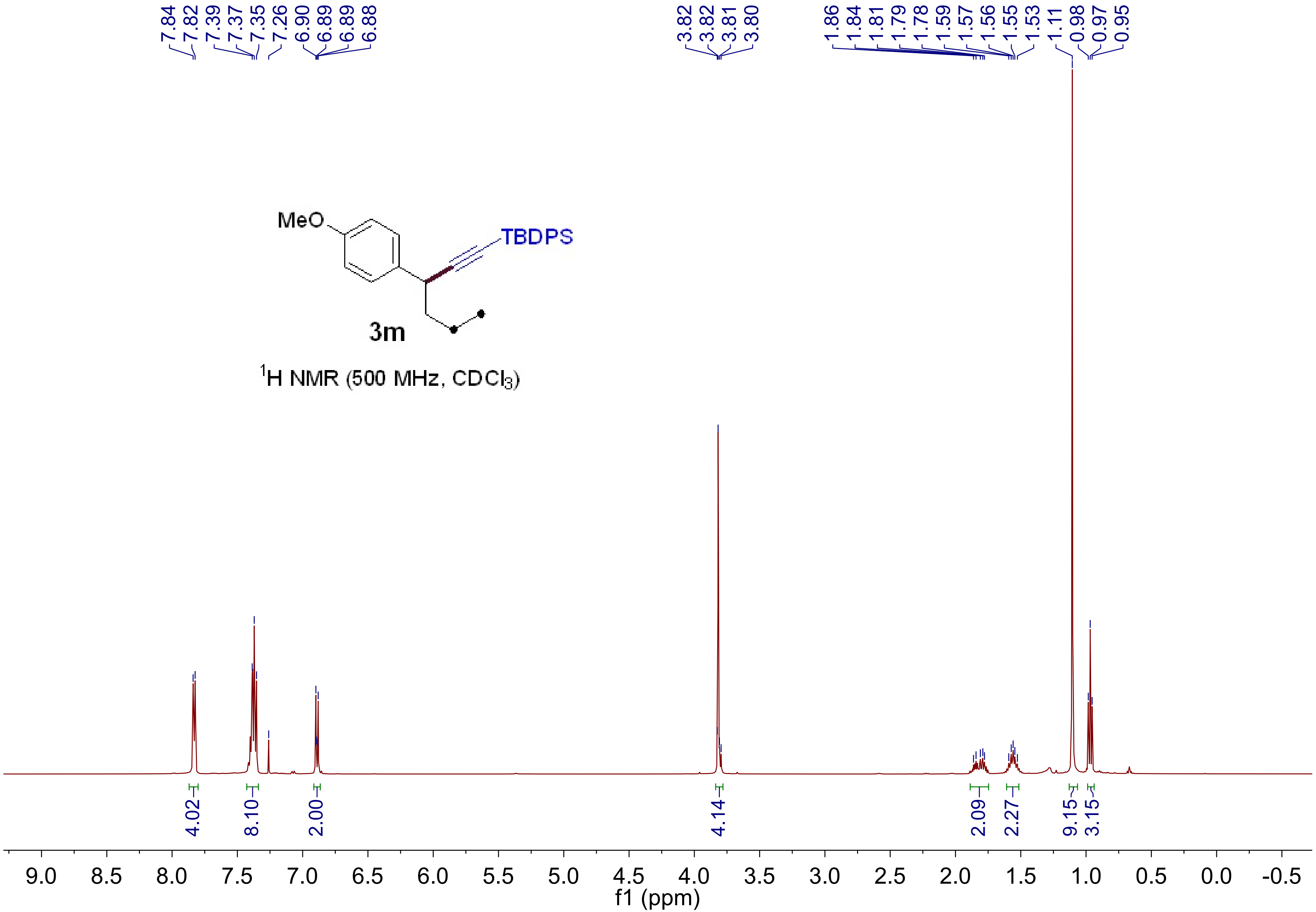

Supplementary Fig. 55. ^{1}H NMR (500 MHz, $CDCl_3$) spectra for compound **3m**

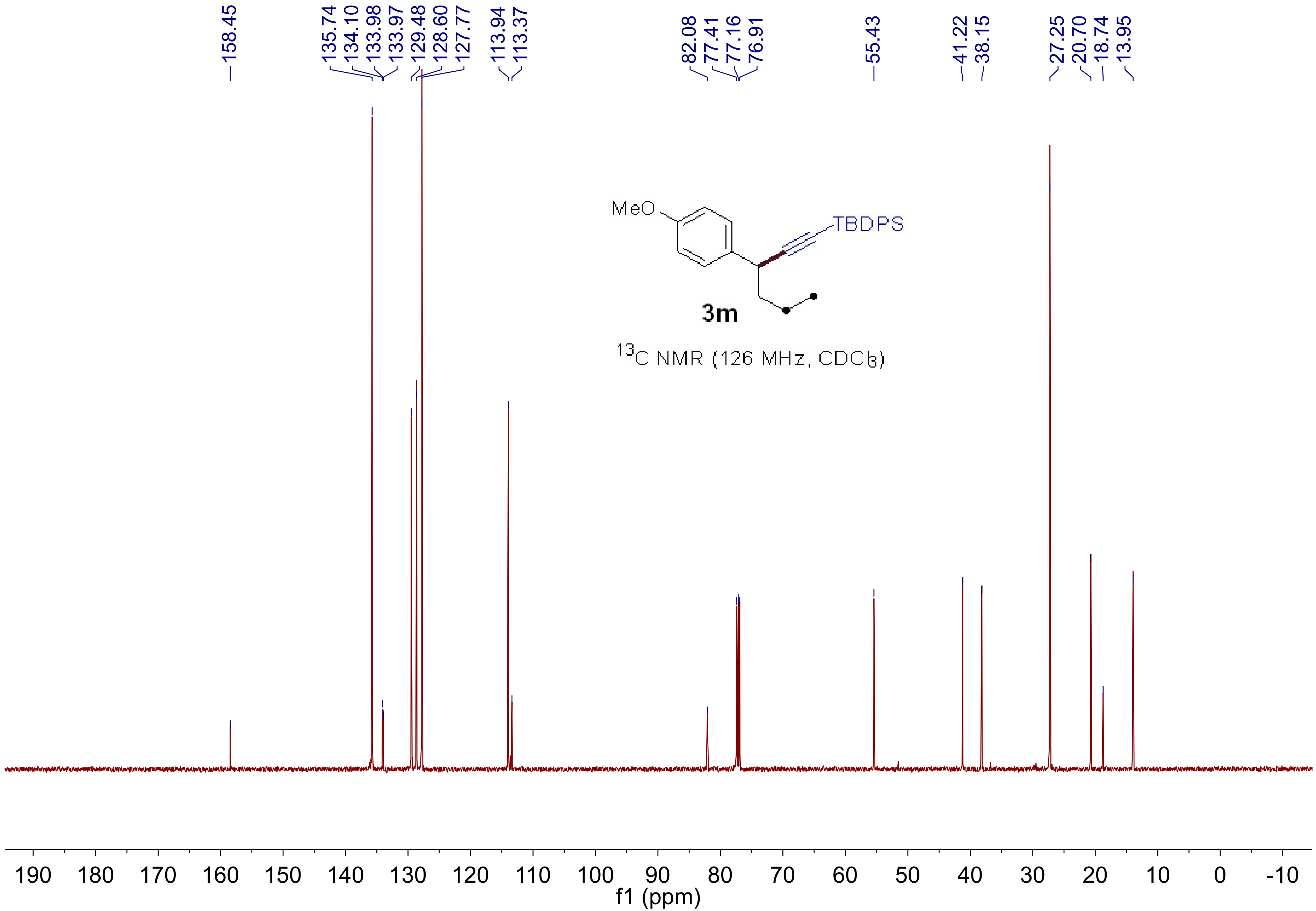

Supplementary Fig. 56. ^{13}C NMR (126 MHz, $CDCl_3$) spectra for compound **3m**

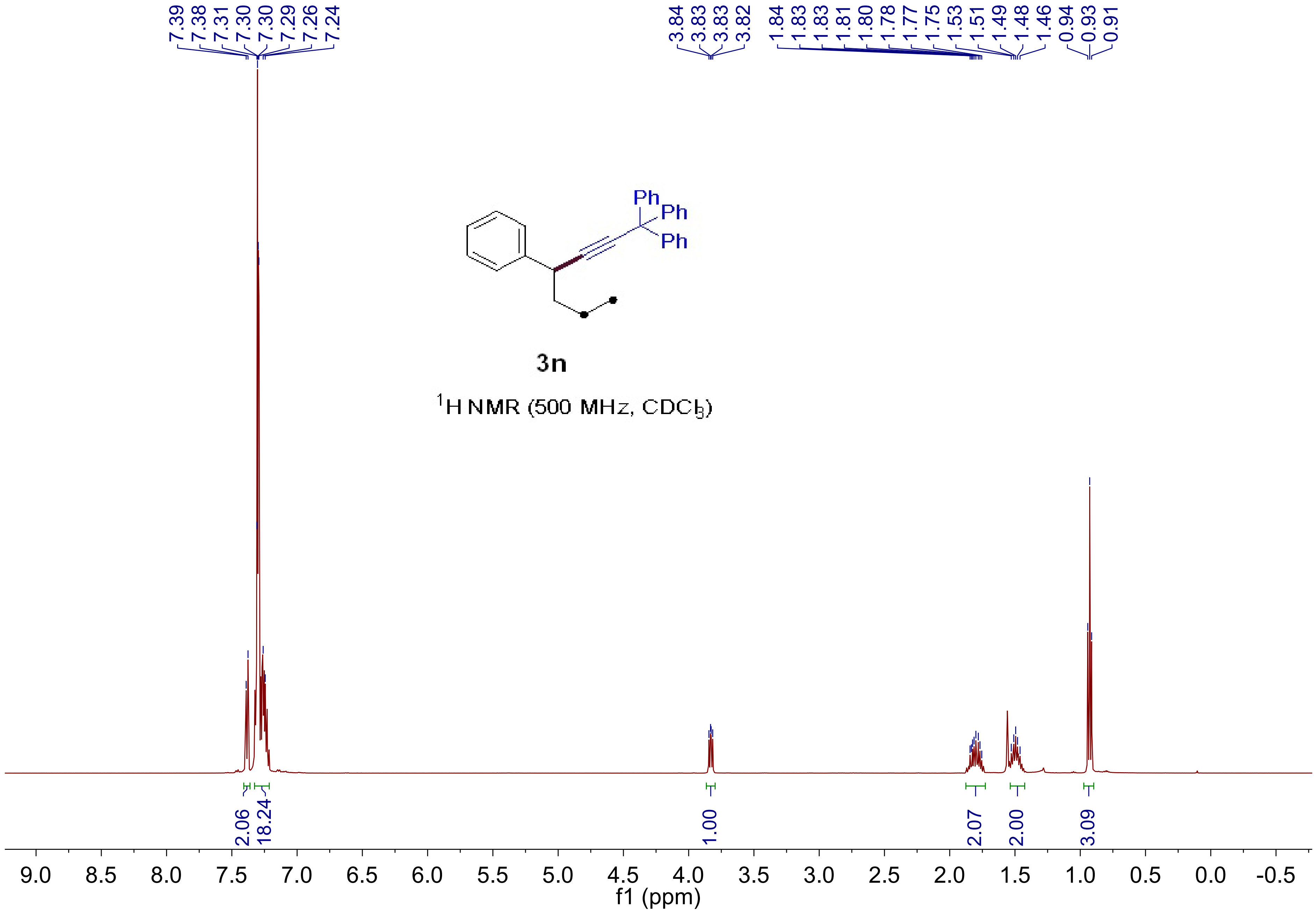

Supplementary Fig. 57. ^{1}H NMR (500 MHz, $CDCl_3$) spectra for compound **3n**

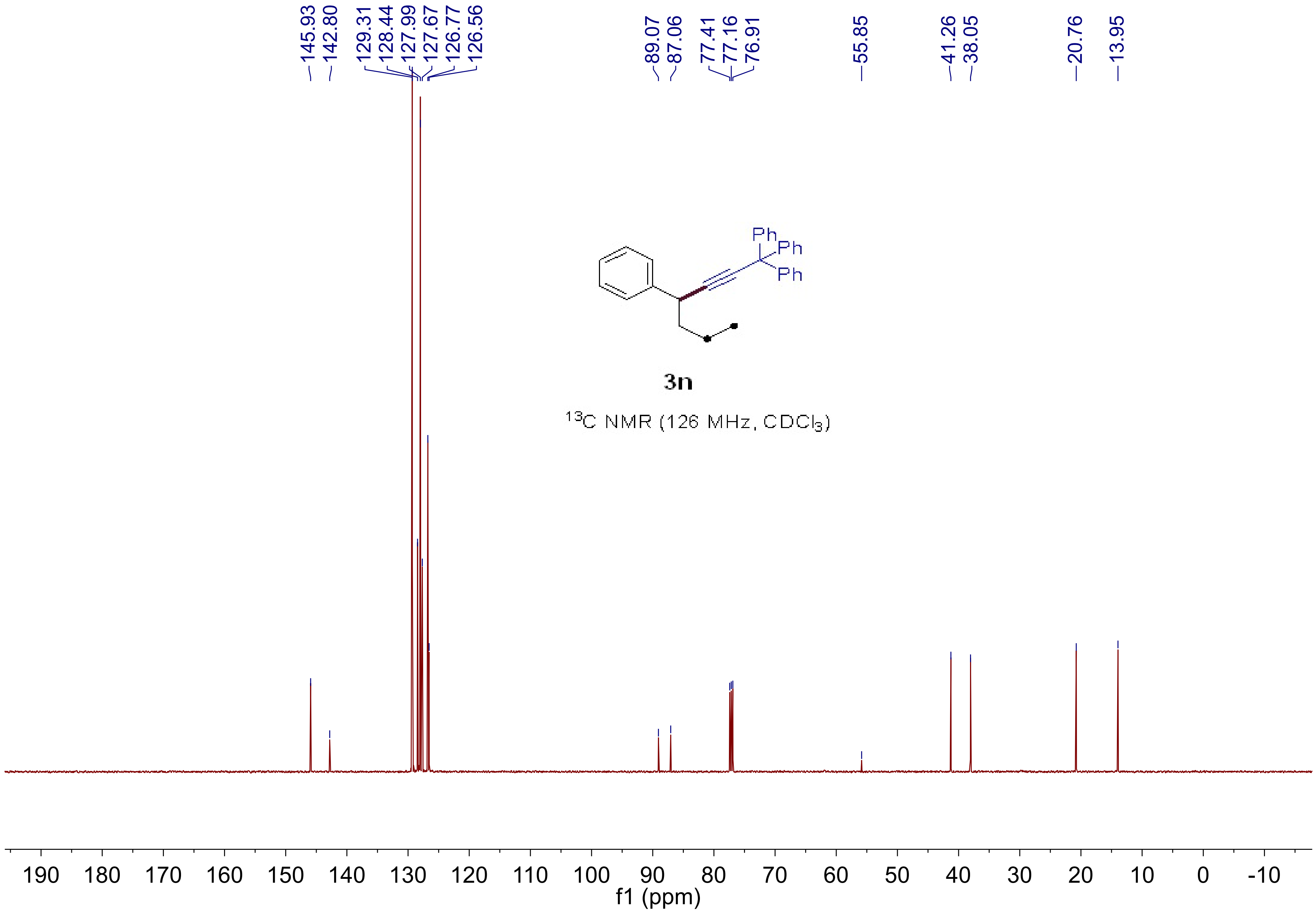

Supplementary Fig. 58. ^{13}C NMR (126 MHz, $CDCl_3$) spectra for compound **3n**

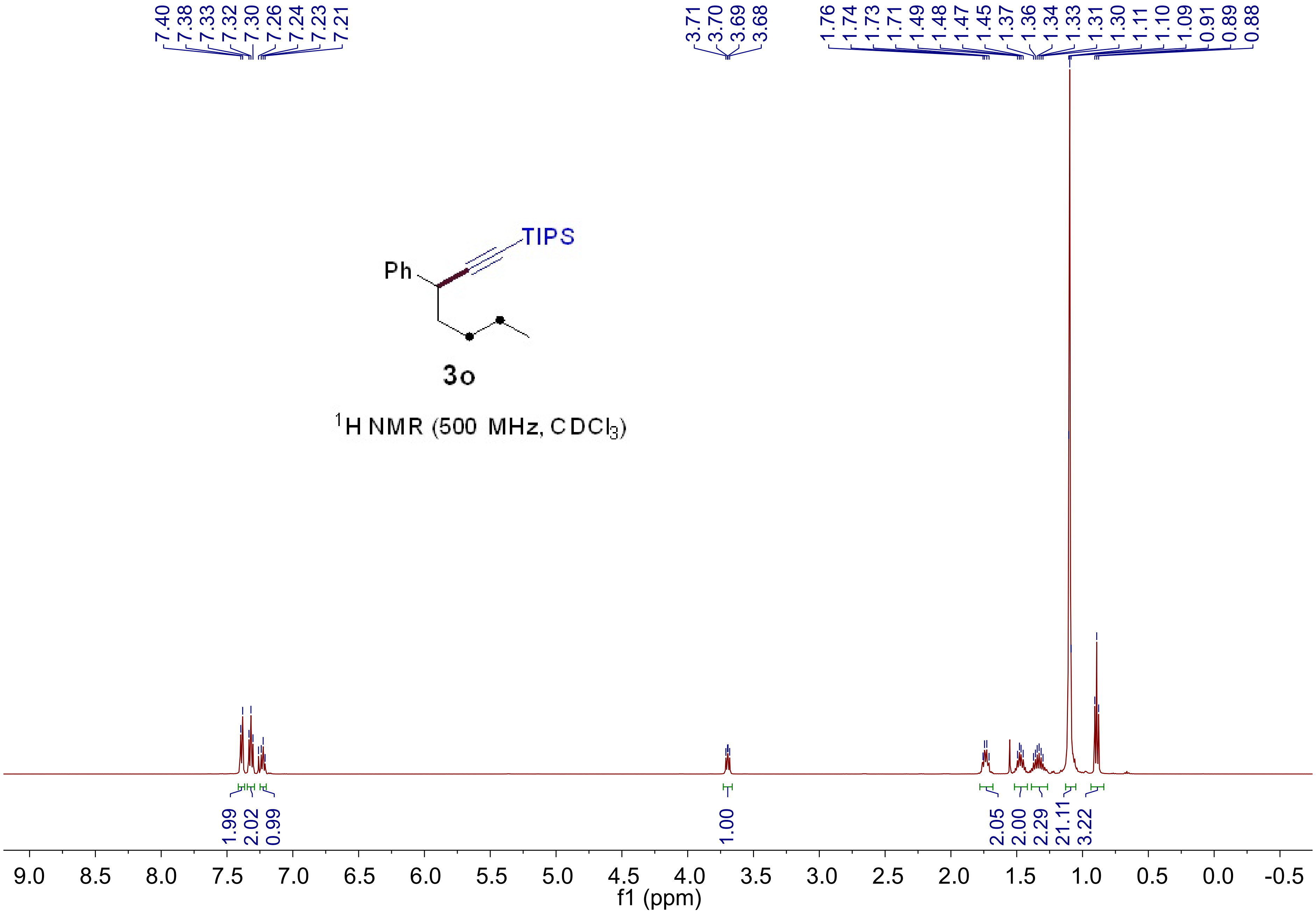

Supplementary Fig. 59. ^{1}H NMR (500 MHz, $CDCl_3$) spectra for compound **3o**

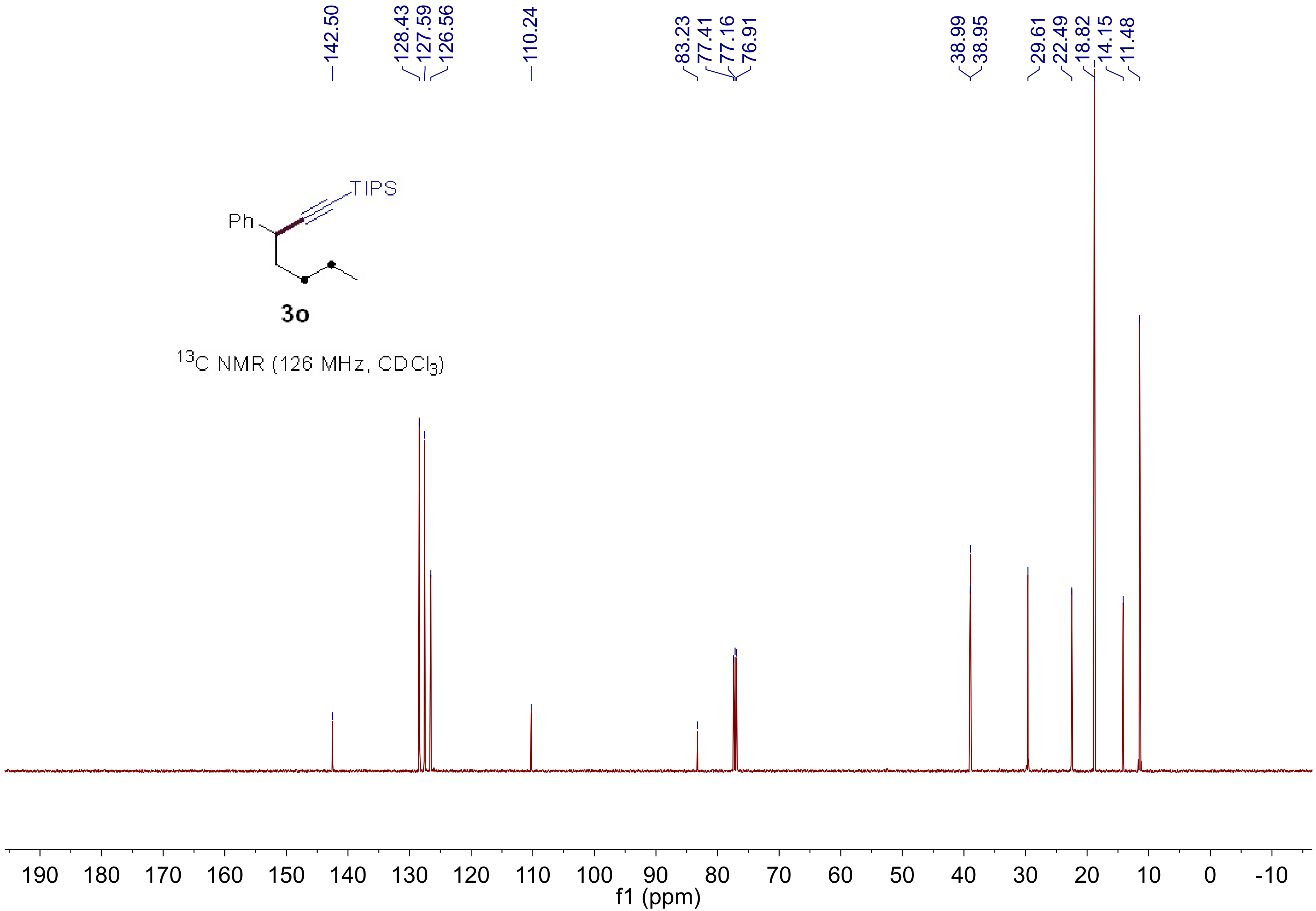

Supplementary Fig. 60. ^{13}C NMR (126 MHz, $CDCl_3$) spectra for compound **3o**

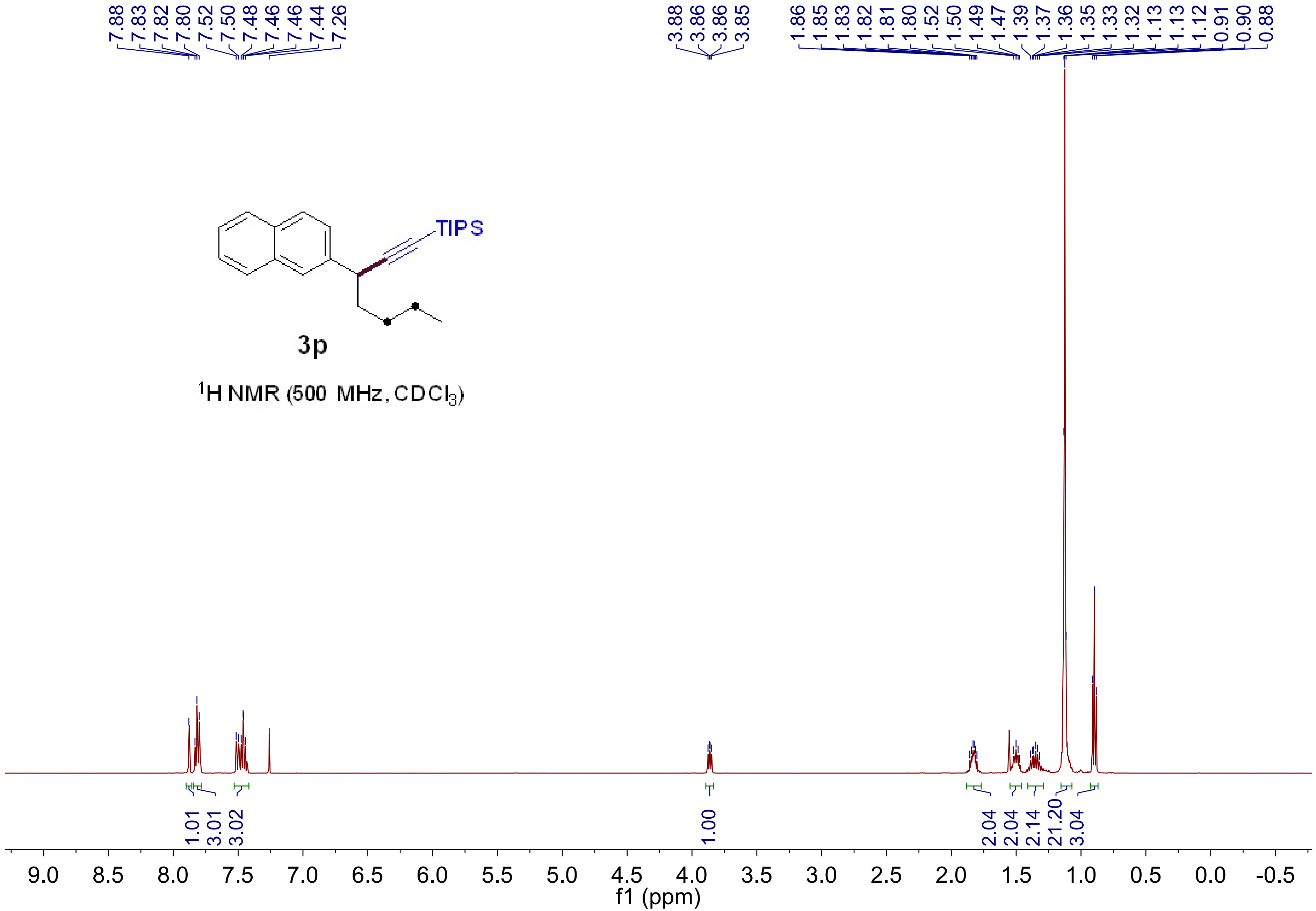

Supplementary Fig. 61. ^{1}H NMR (500 MHz, $CDCl_3$) spectra for compound **3p**

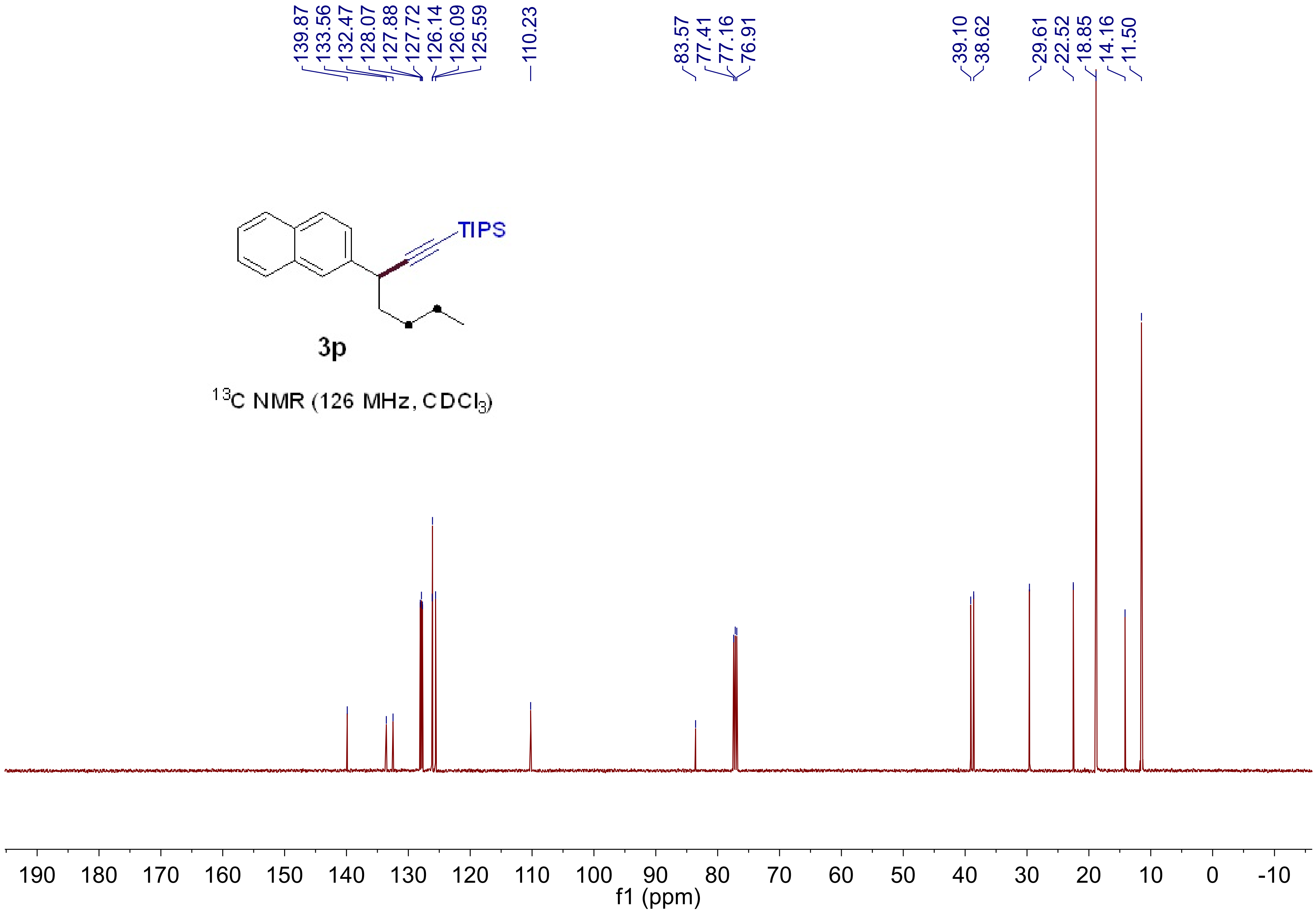

Supplementary Fig. 62. ^{13}C NMR (126 MHz, $CDCl_3$) spectra for compound **3p**

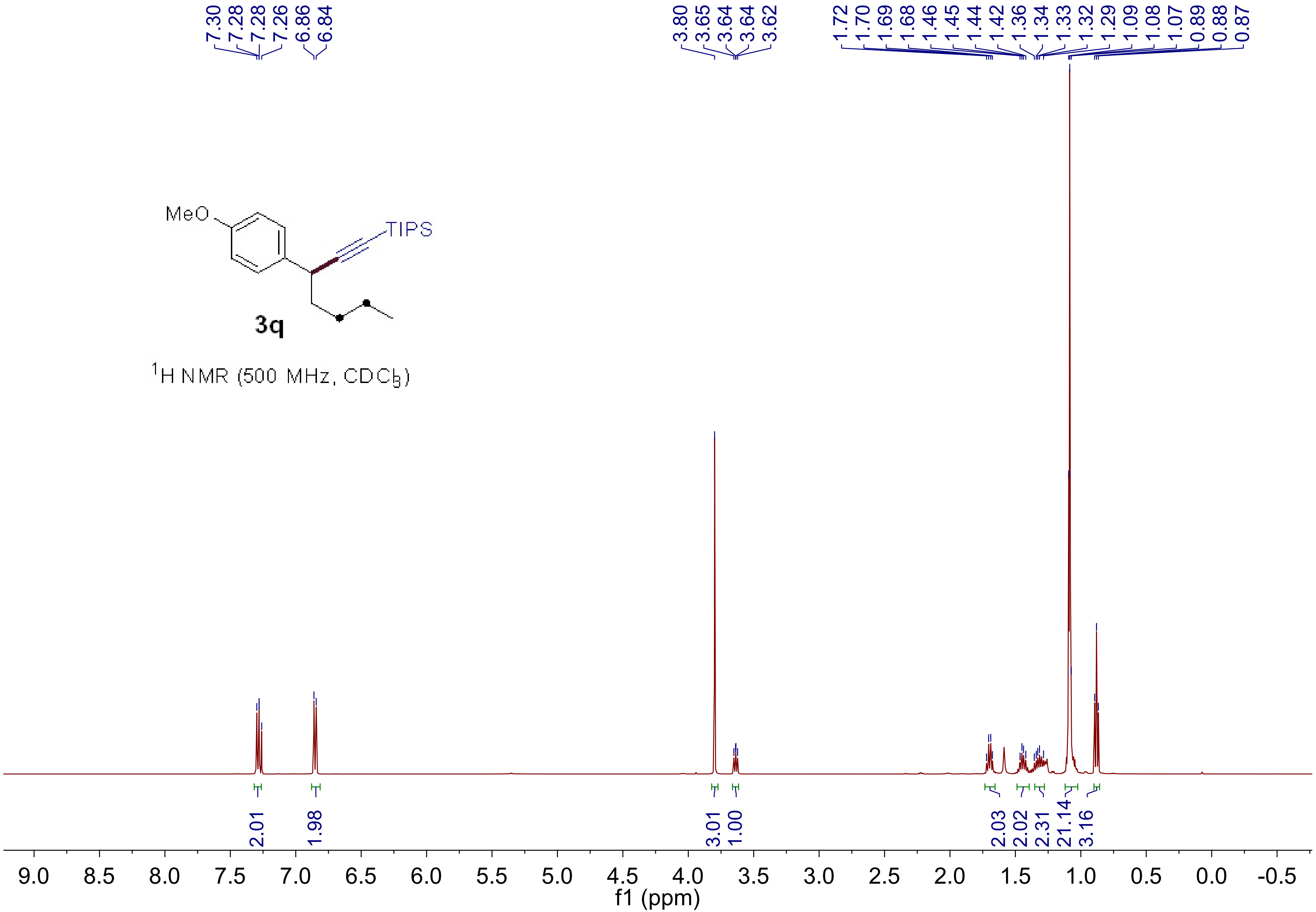

Supplementary Fig. 63. ^{1}H NMR (500 MHz, $CDCl_3$) spectra for compound **3q**

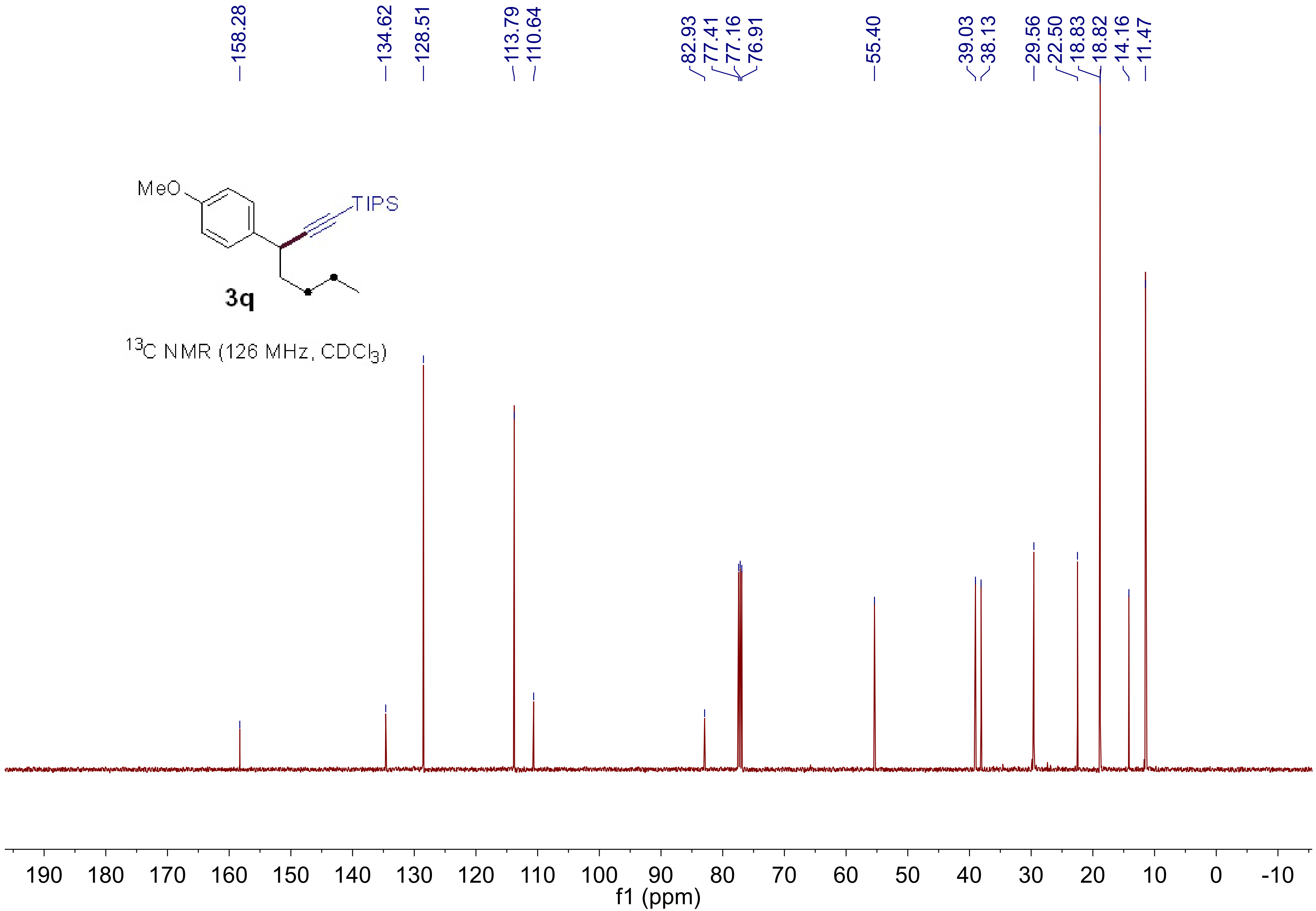

Supplementary Fig. 64. ^{13}C NMR (126 MHz, $CDCl_3$) spectra for compound **3q**

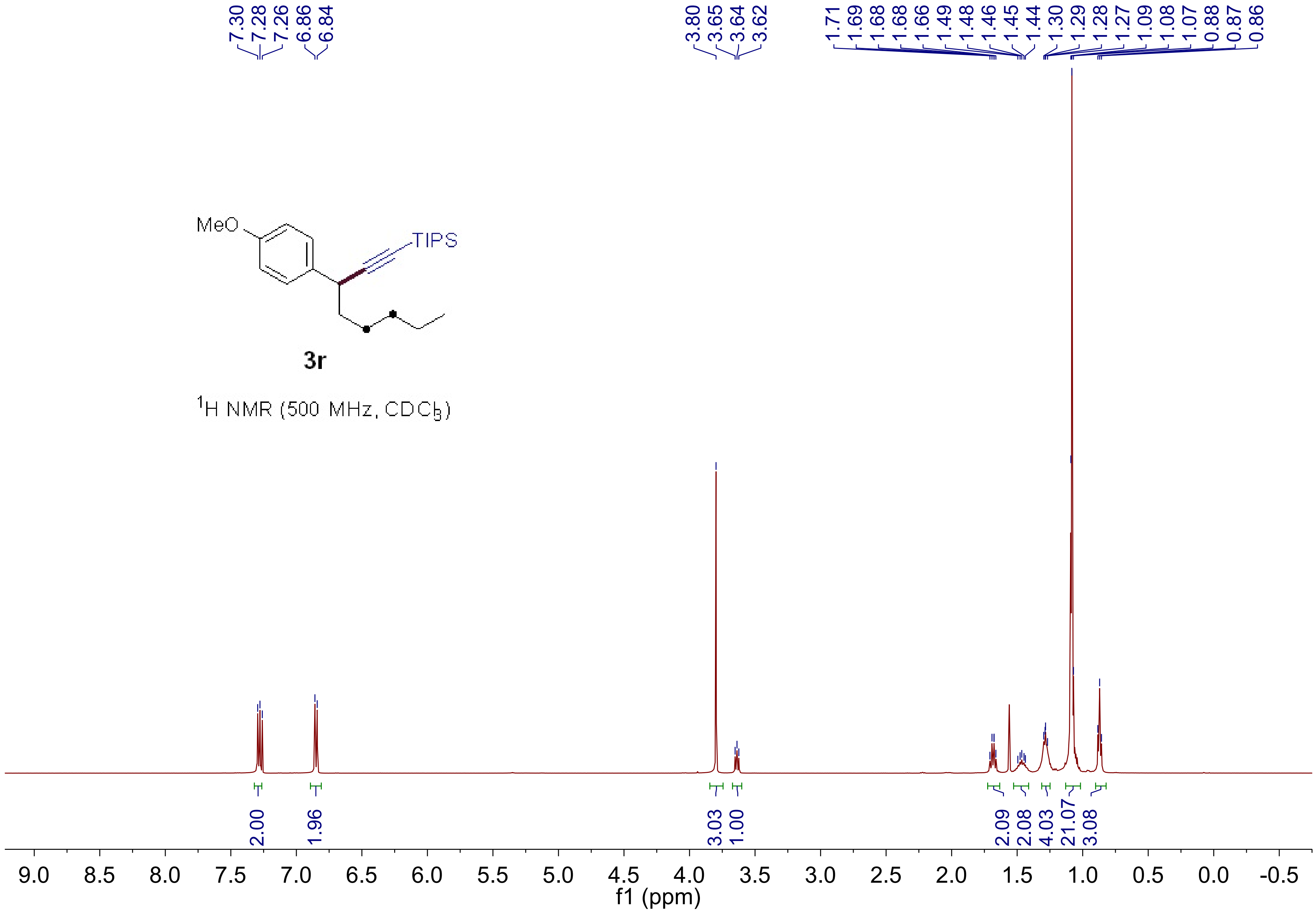

Supplementary Fig. 65. ^{1}H NMR (500 MHz, $CDCl_3$) spectra for compound **3r**

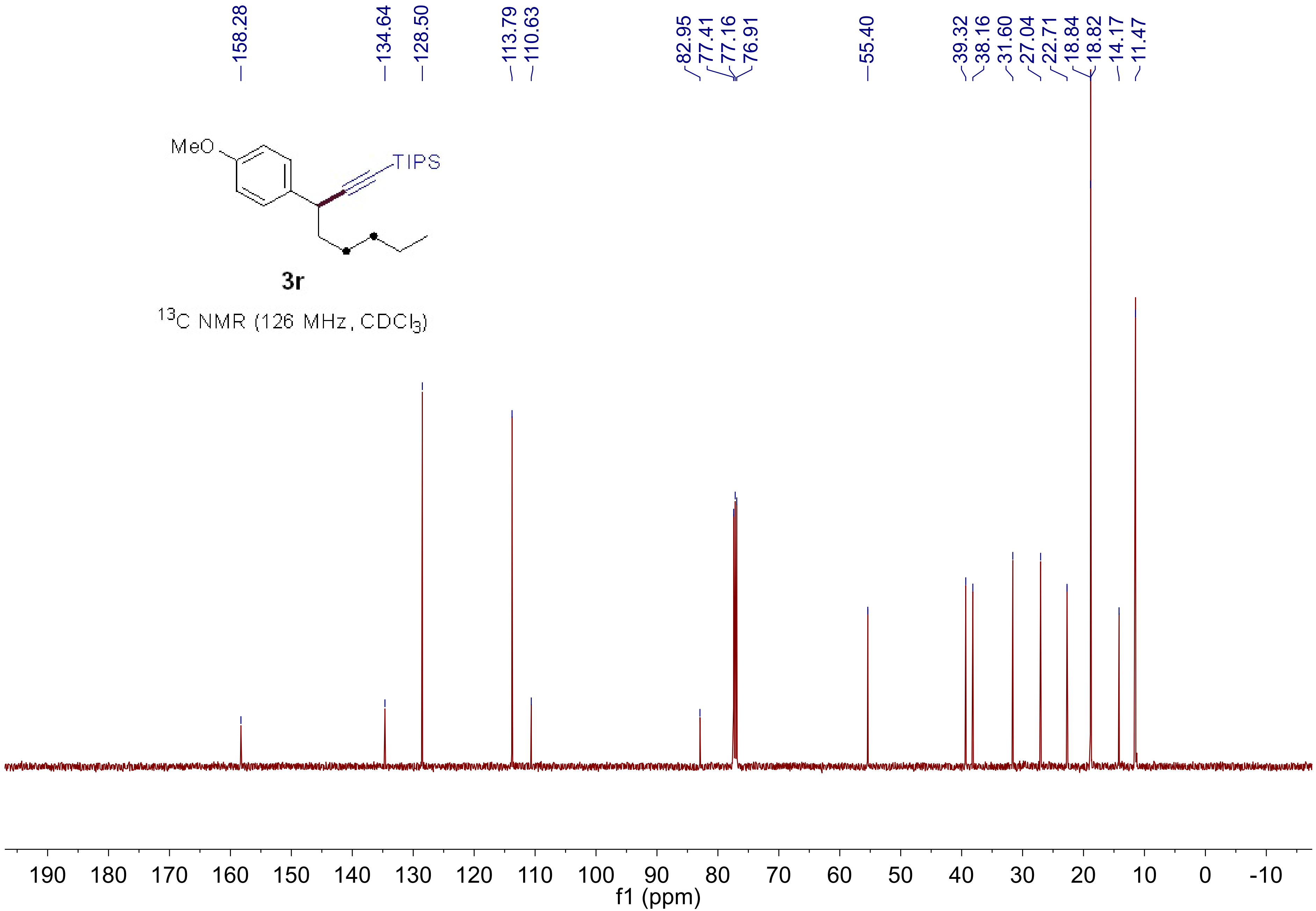

Supplementary Fig. 66. ^{13}C NMR (126 MHz, $CDCl_3$) spectra for compound **3r**

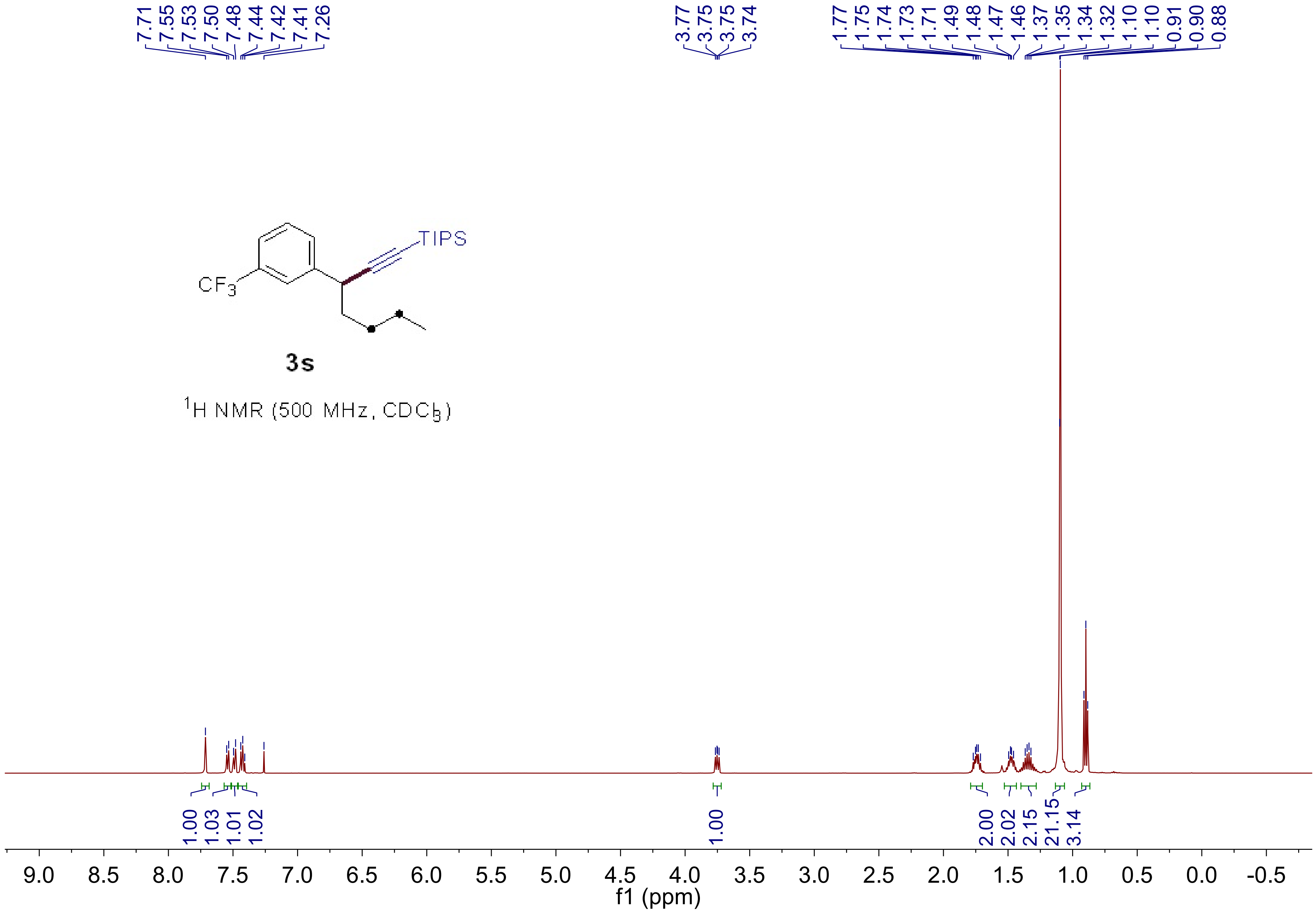

Supplementary Fig. 67. ^{1}H NMR (500 MHz, $CDCl_3$) spectra for compound **3s**

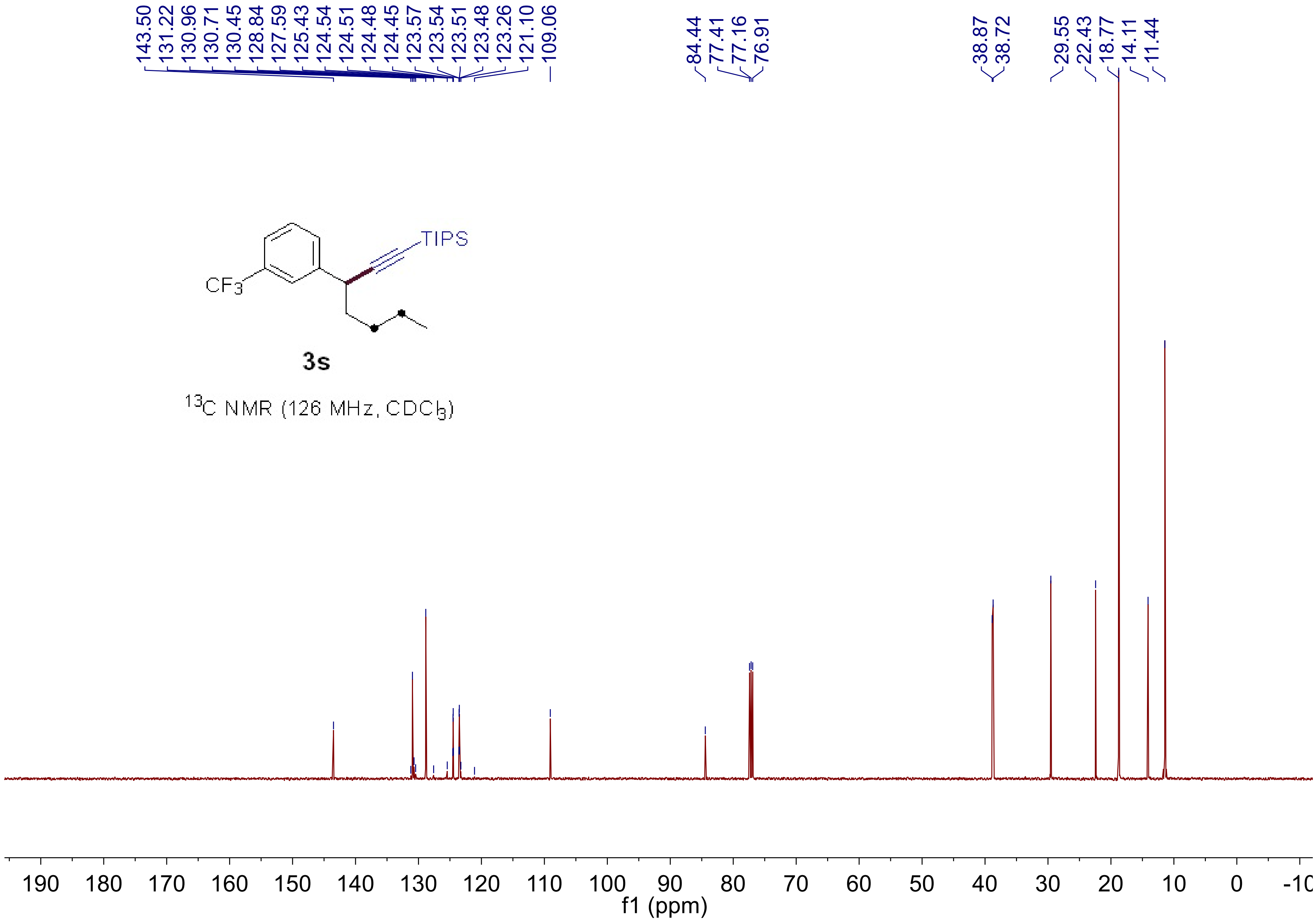

Supplementary Fig. 68. ^{13}C NMR (126 MHz, $CDCl_3$) spectra for compound **3s**

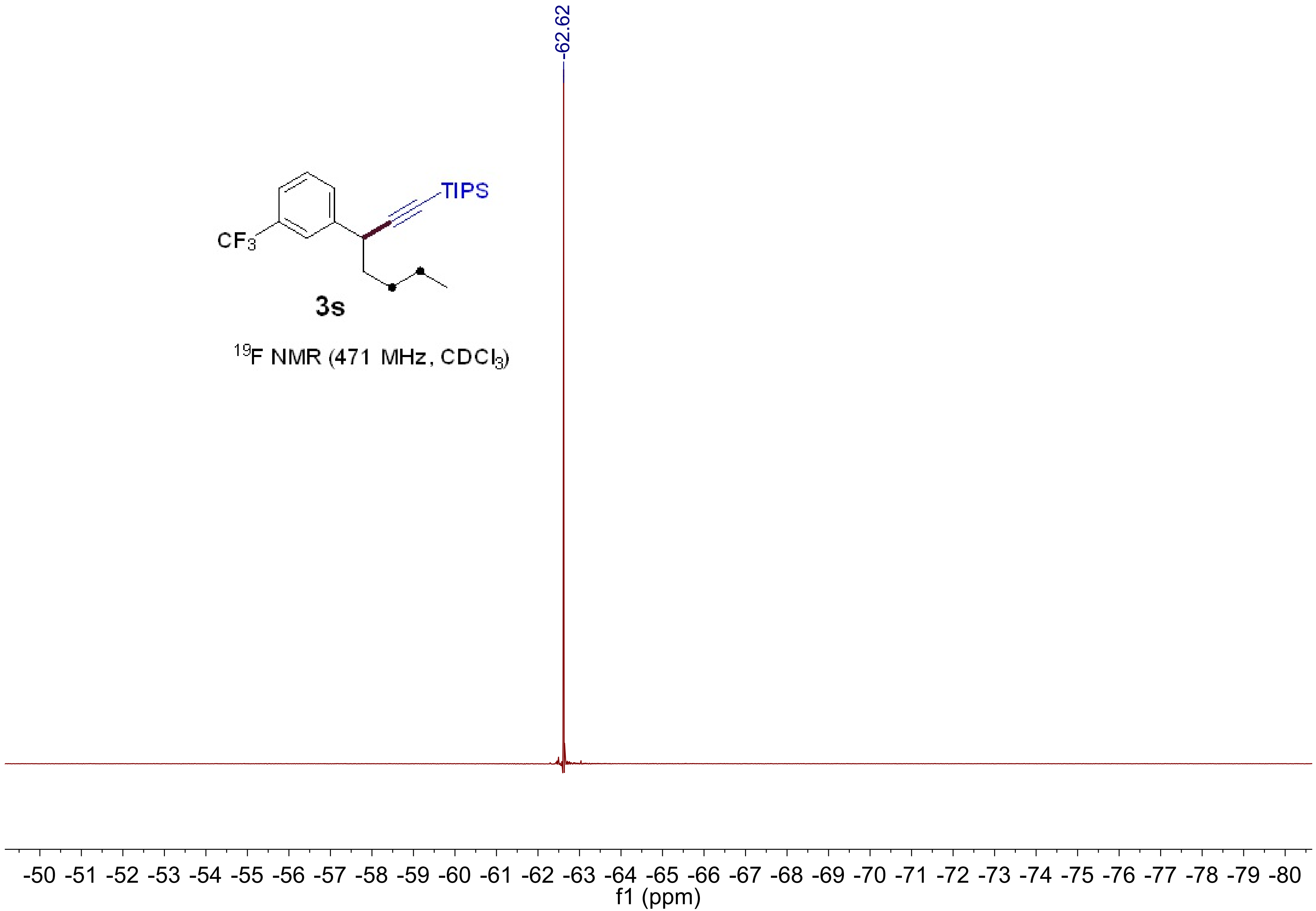

Supplementary Fig. 69. ^{19}F NMR (471 MHz, $CDCl_3$) spectra for compound **3s**

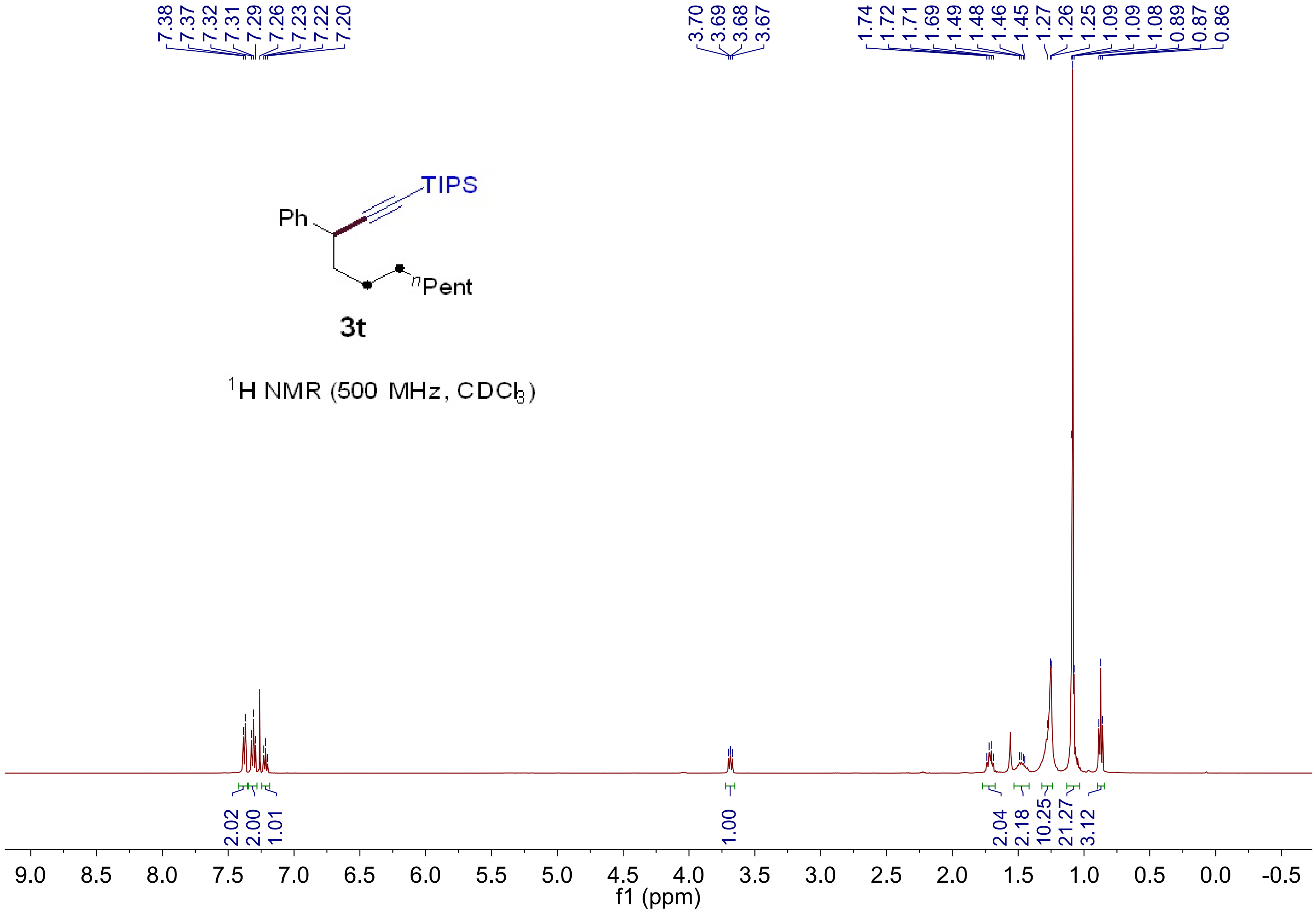

Supplementary Fig. 70. ^{1}H NMR (500 MHz, $CDCl_3$) spectra for compound **3t**

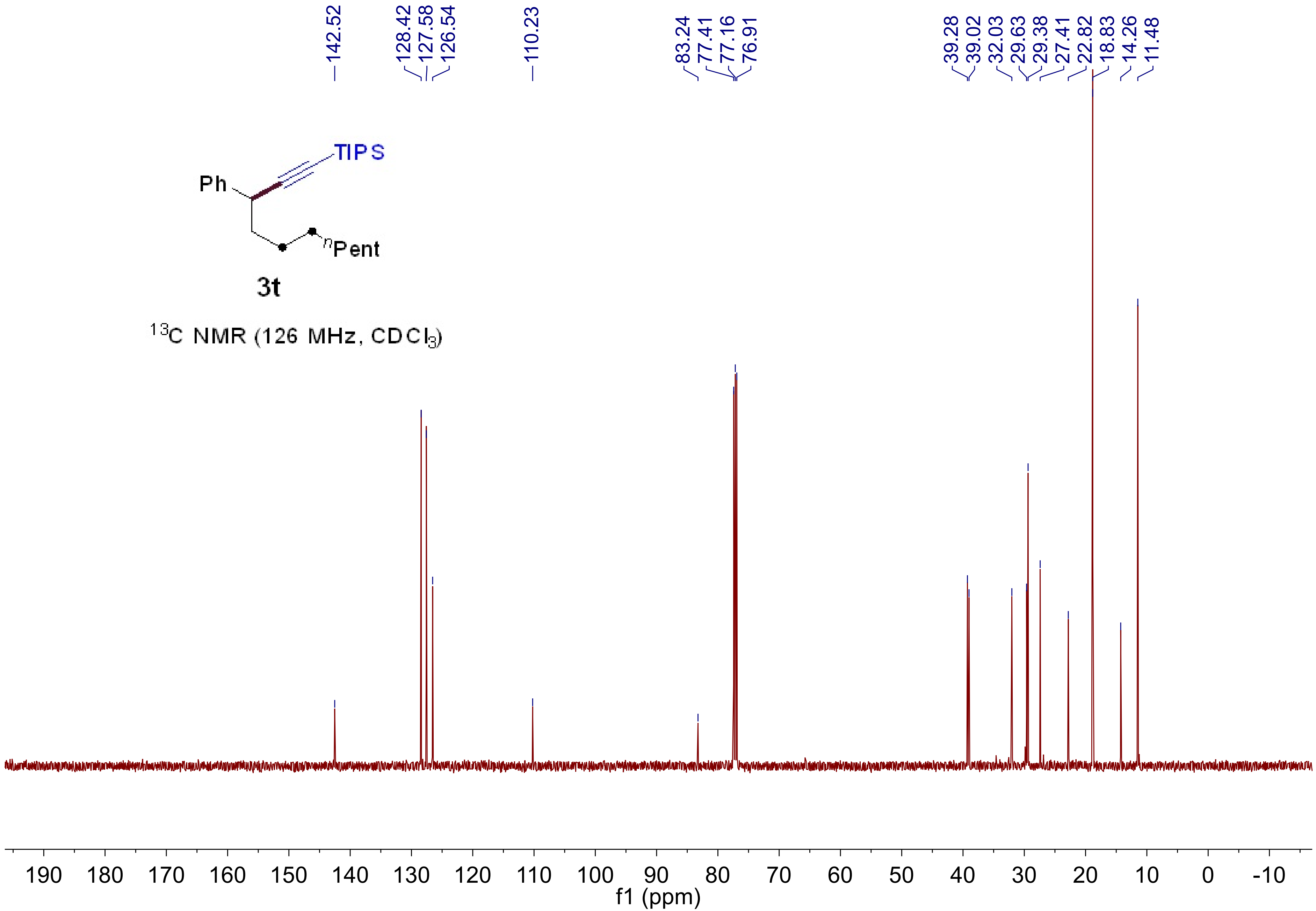

Supplementary Fig. 71. ^{13}C NMR (126 MHz, $CDCl_3$) spectra for compound **3t**

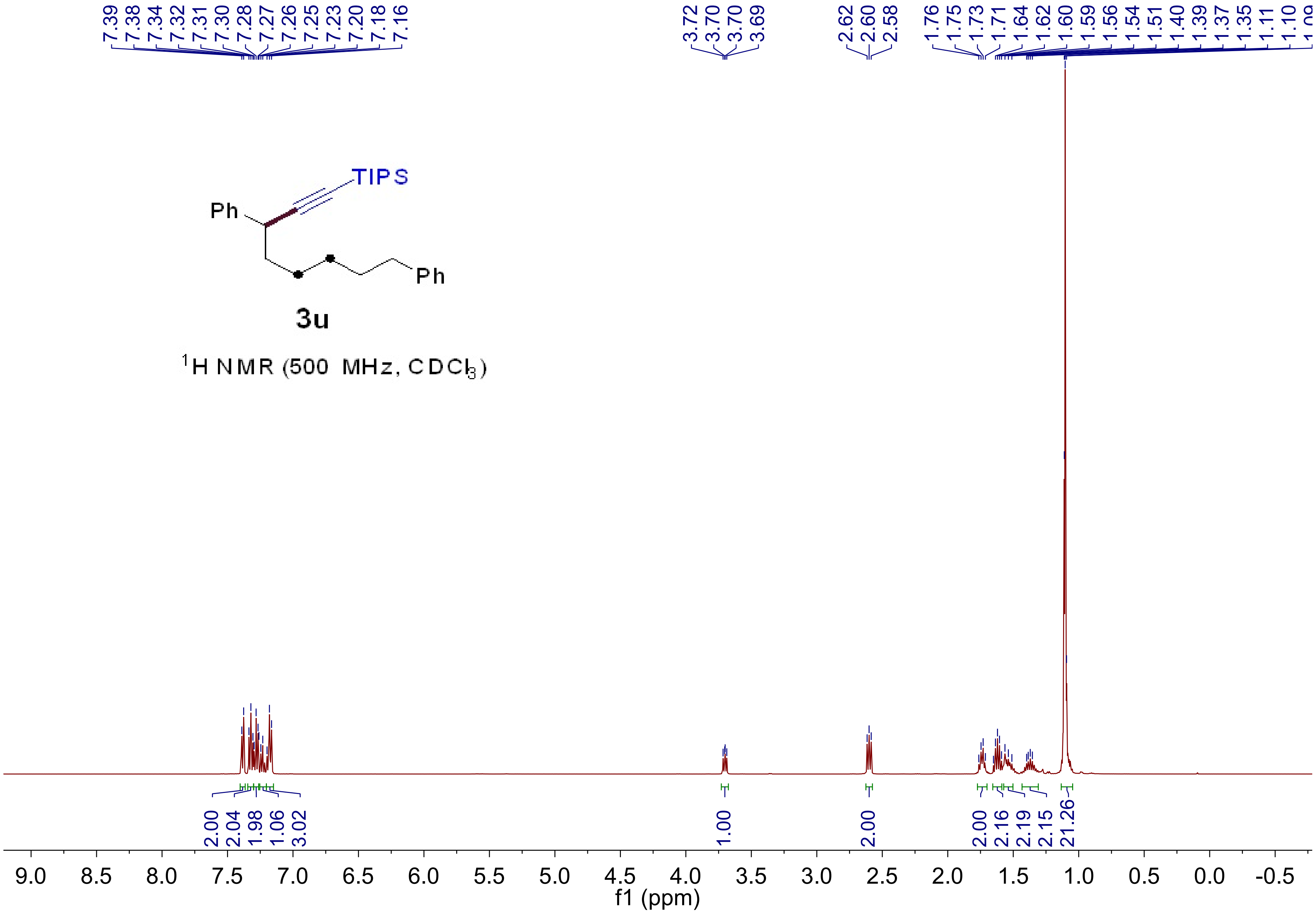

Supplementary Fig. 72. ^{1}H NMR (500 MHz, $CDCl_3$) spectra for compound **3u**

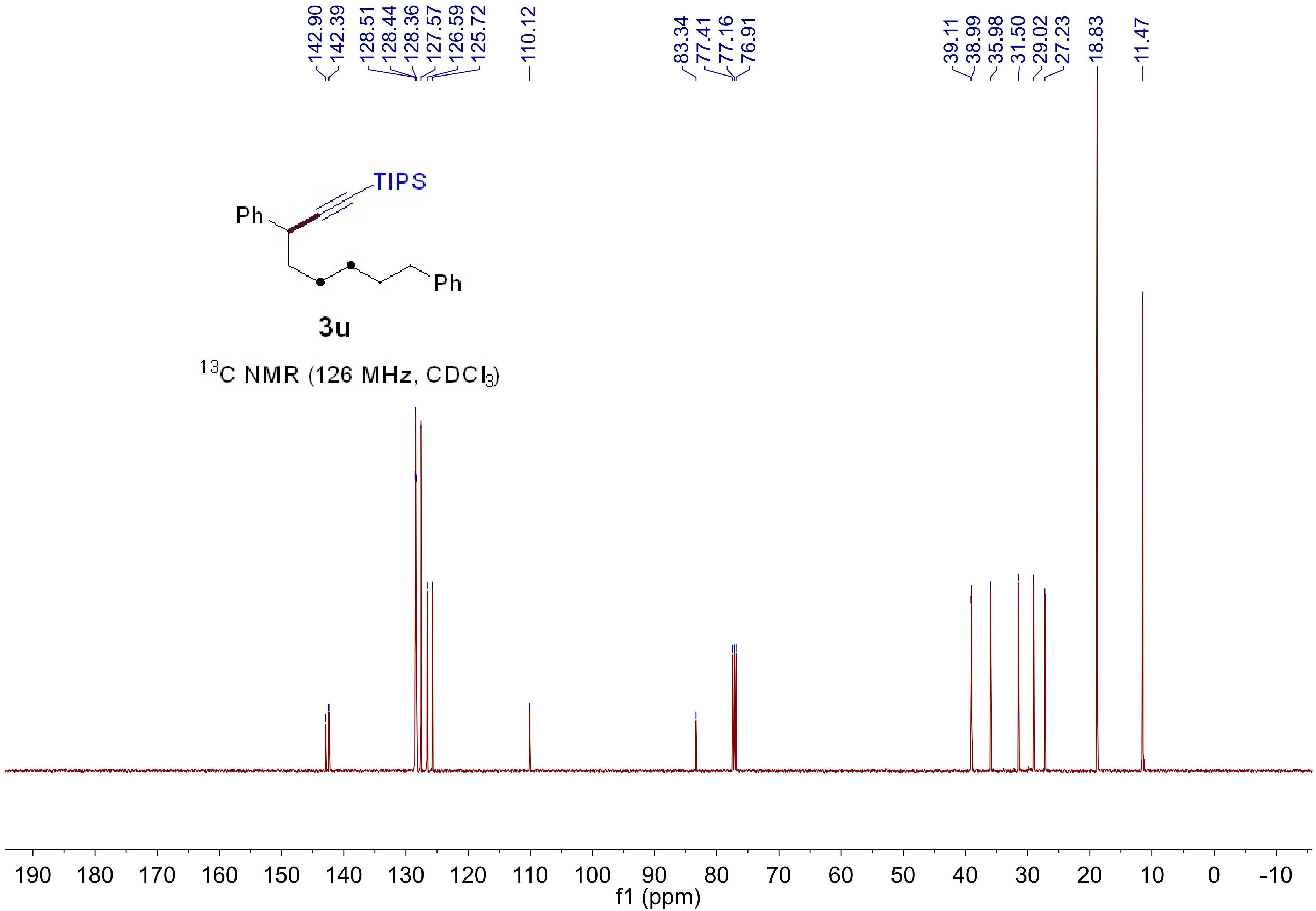

Supplementary Fig. 73. ^{13}C NMR (126 MHz, CDCl$_3$) spectra for compound **3u**

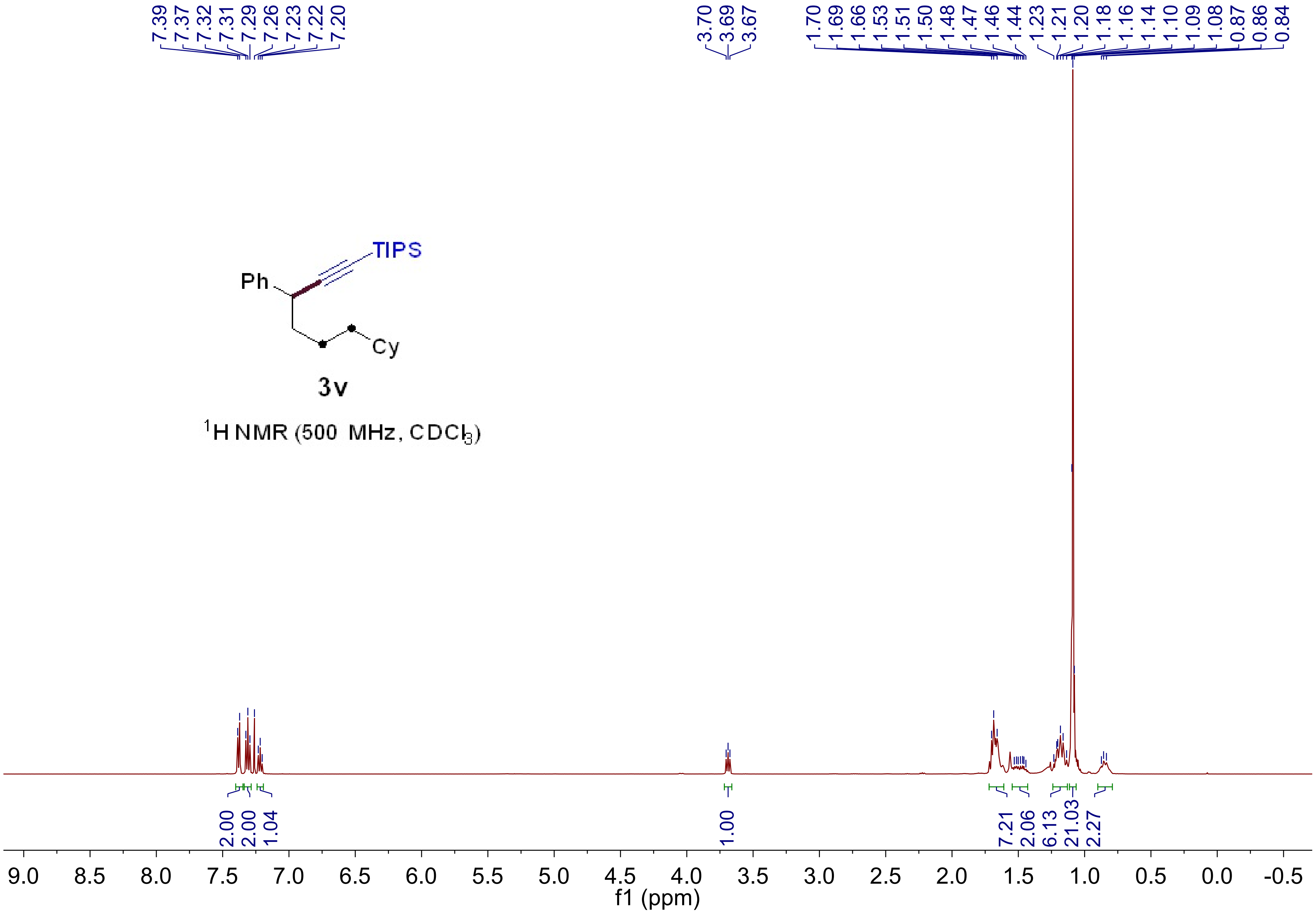

Supplementary Fig. 74. ^{1}H NMR (500 MHz, $CDCl_3$) spectra for compound **3v**

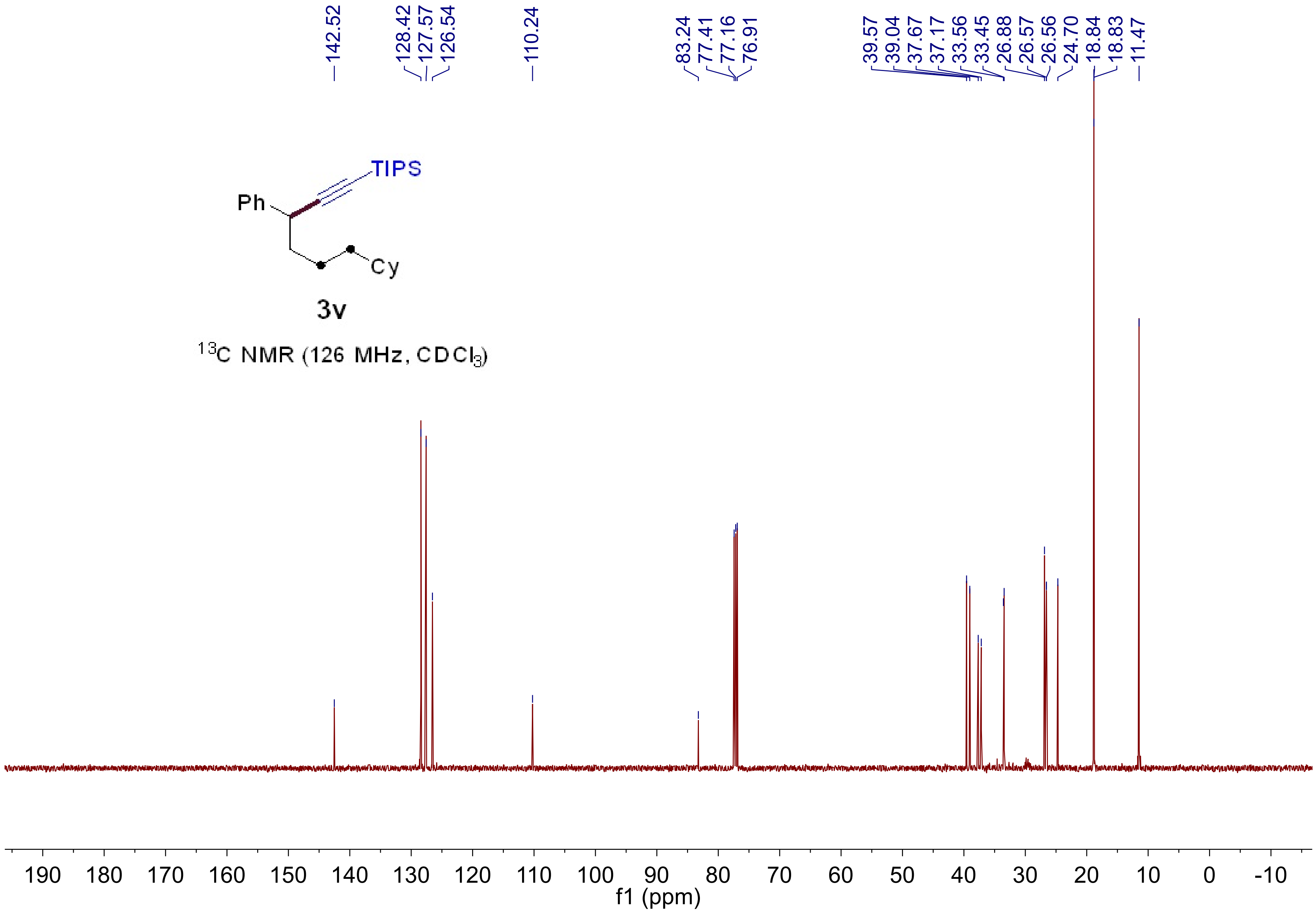

Supplementary Fig. 75. ^{13}C NMR (126 MHz, $CDCl_3$) spectra for compound **3v**

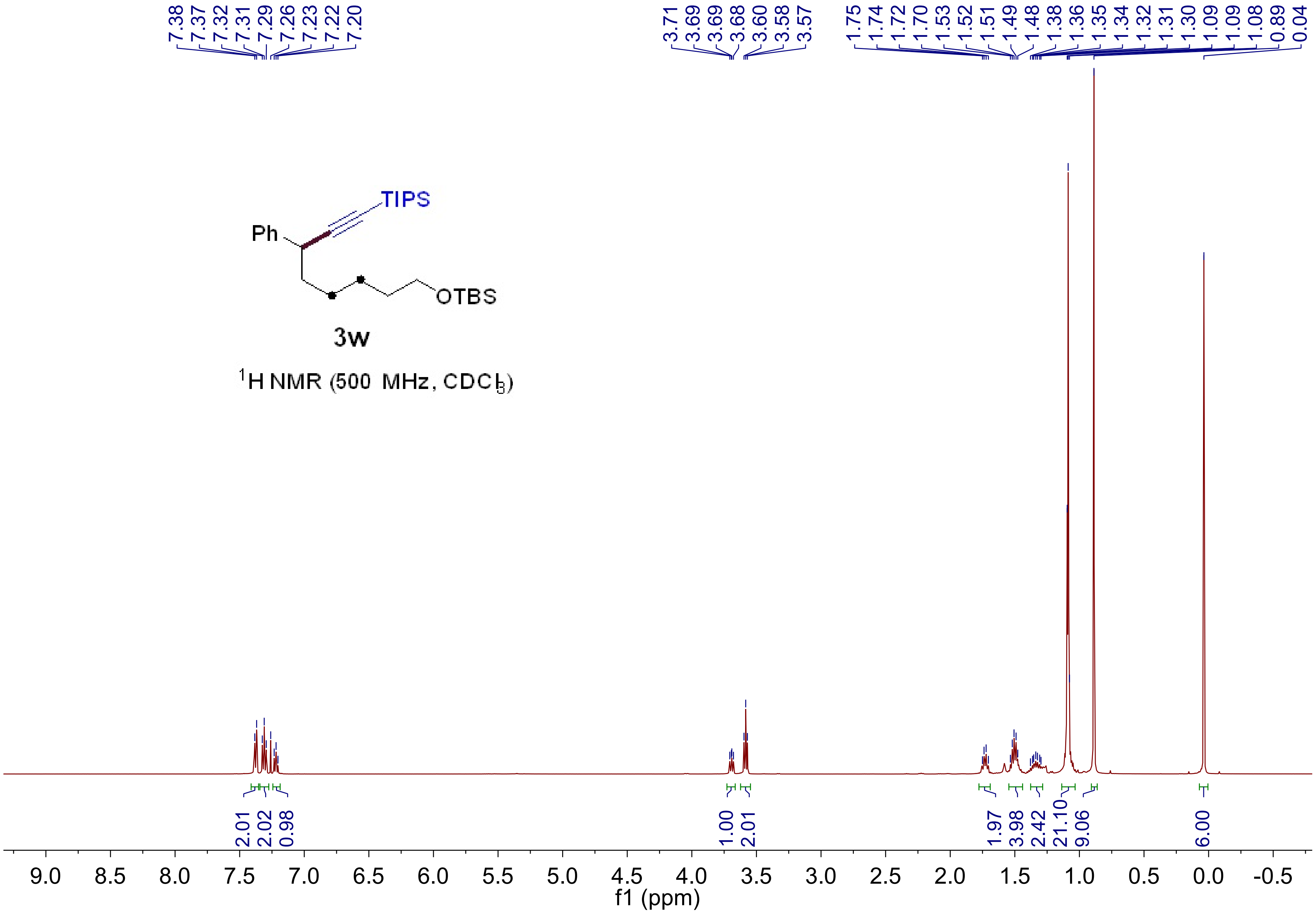

Supplementary Fig. 76. ^{1}H NMR (500 MHz, $CDCl_3$) spectra for compound **3w**

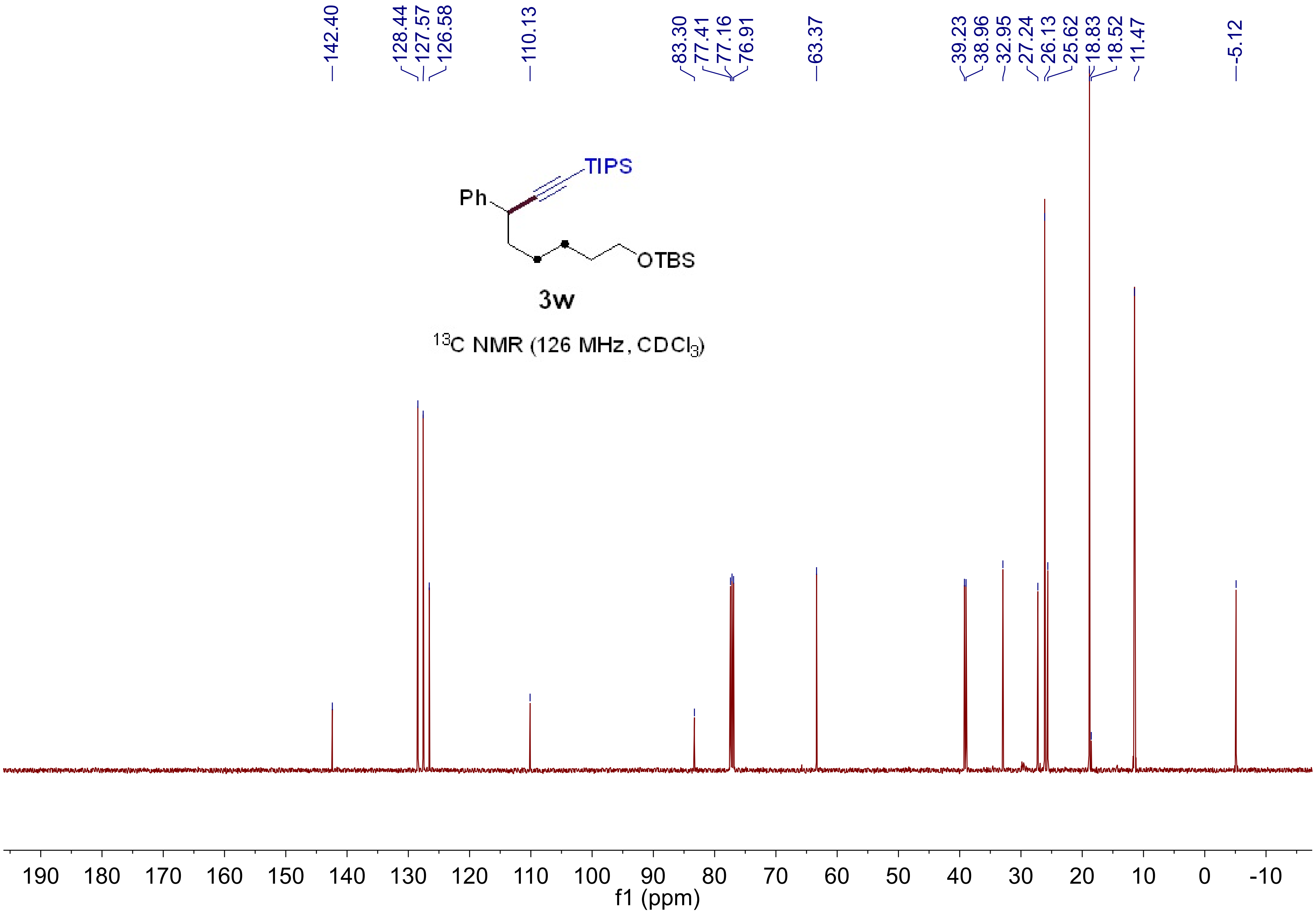

Supplementary Fig. 77. ^{13}C NMR (126 MHz, $CDCl_3$) spectra for compound **3w**

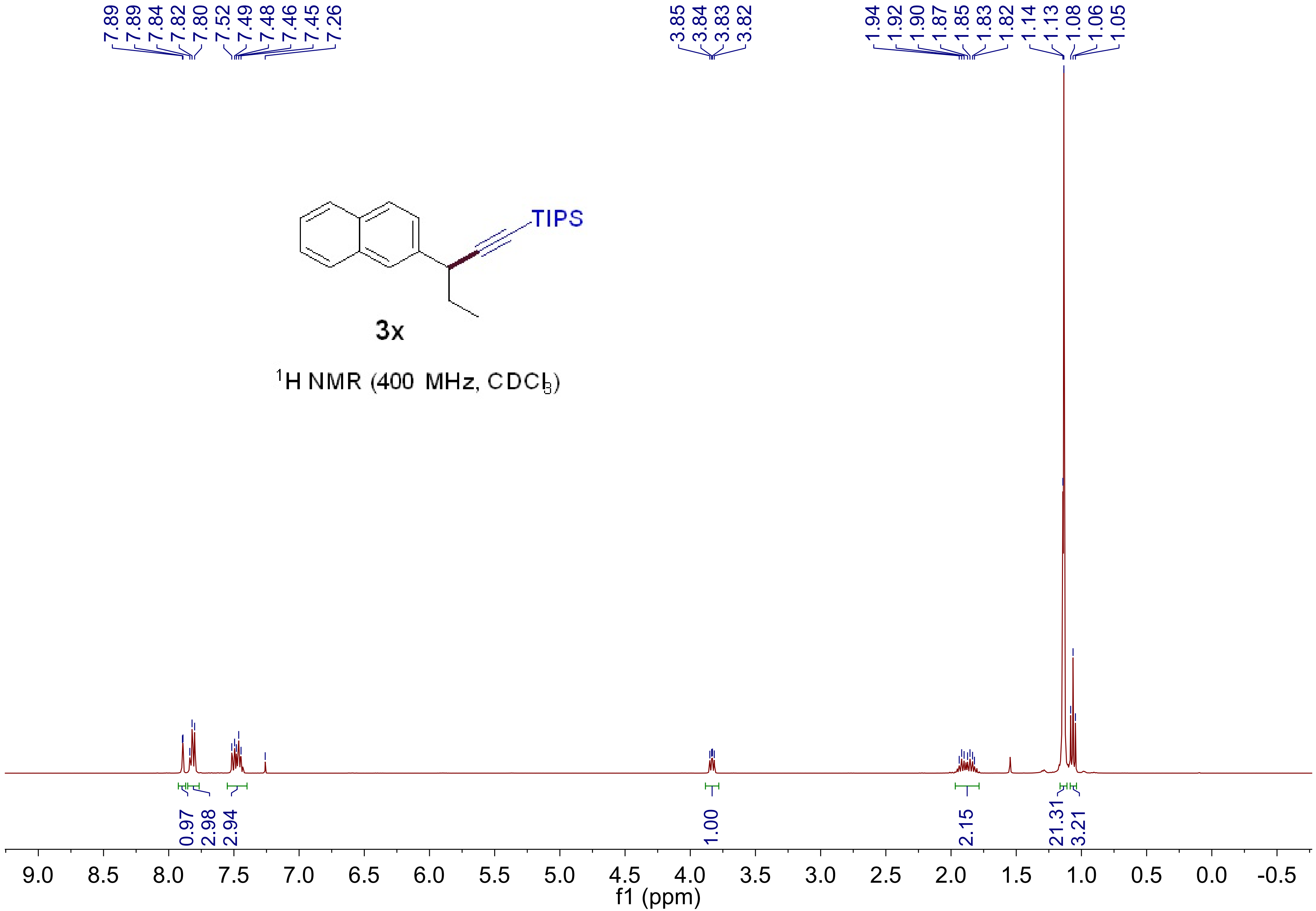

Supplementary Fig. 78. ^{1}H NMR (400 MHz, $CDCl_3$) spectra for compound **3x**

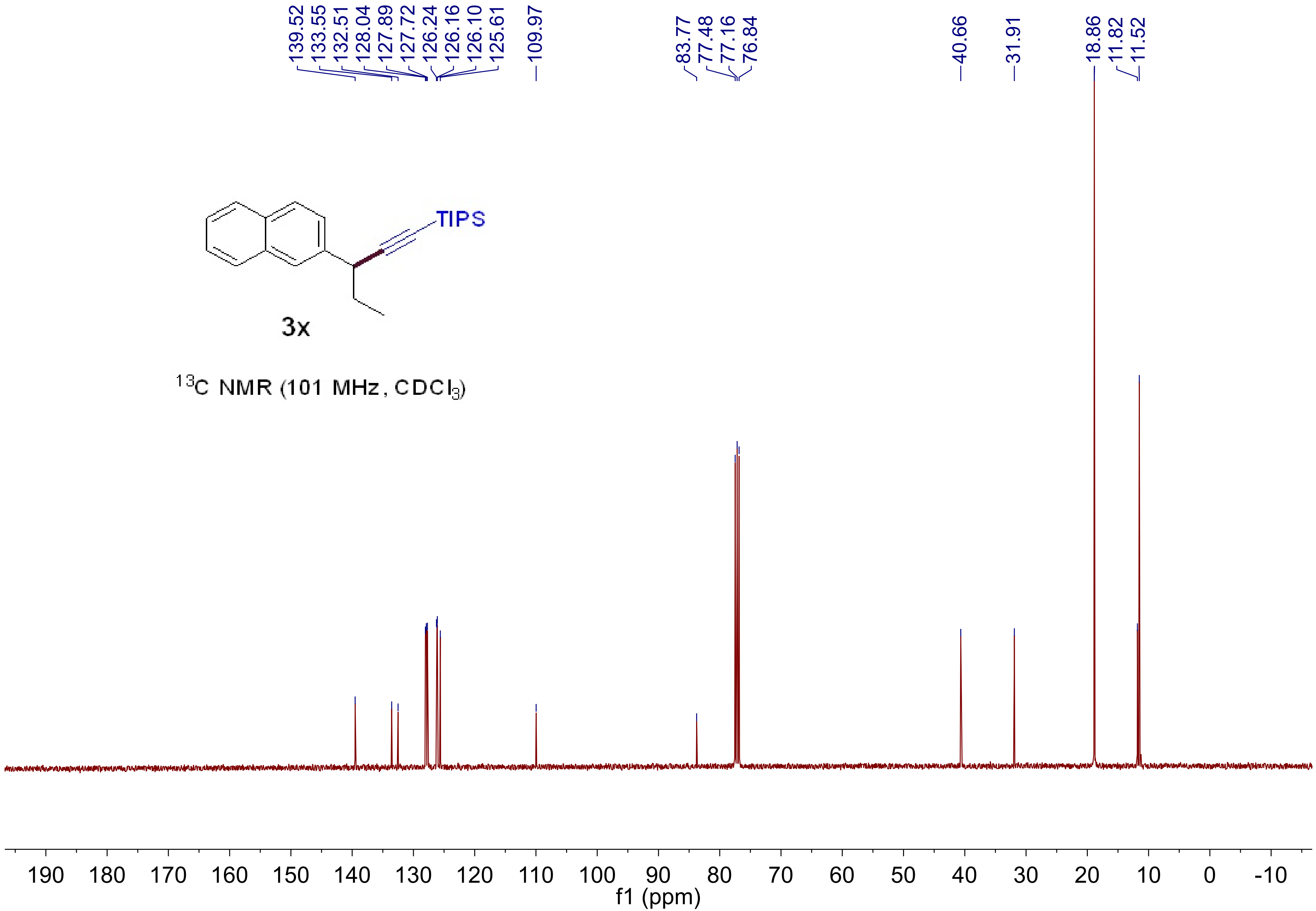

Supplementary Fig. 79. ^{13}C NMR (101 MHz, $CDCl_3$) spectra for compound **3x**

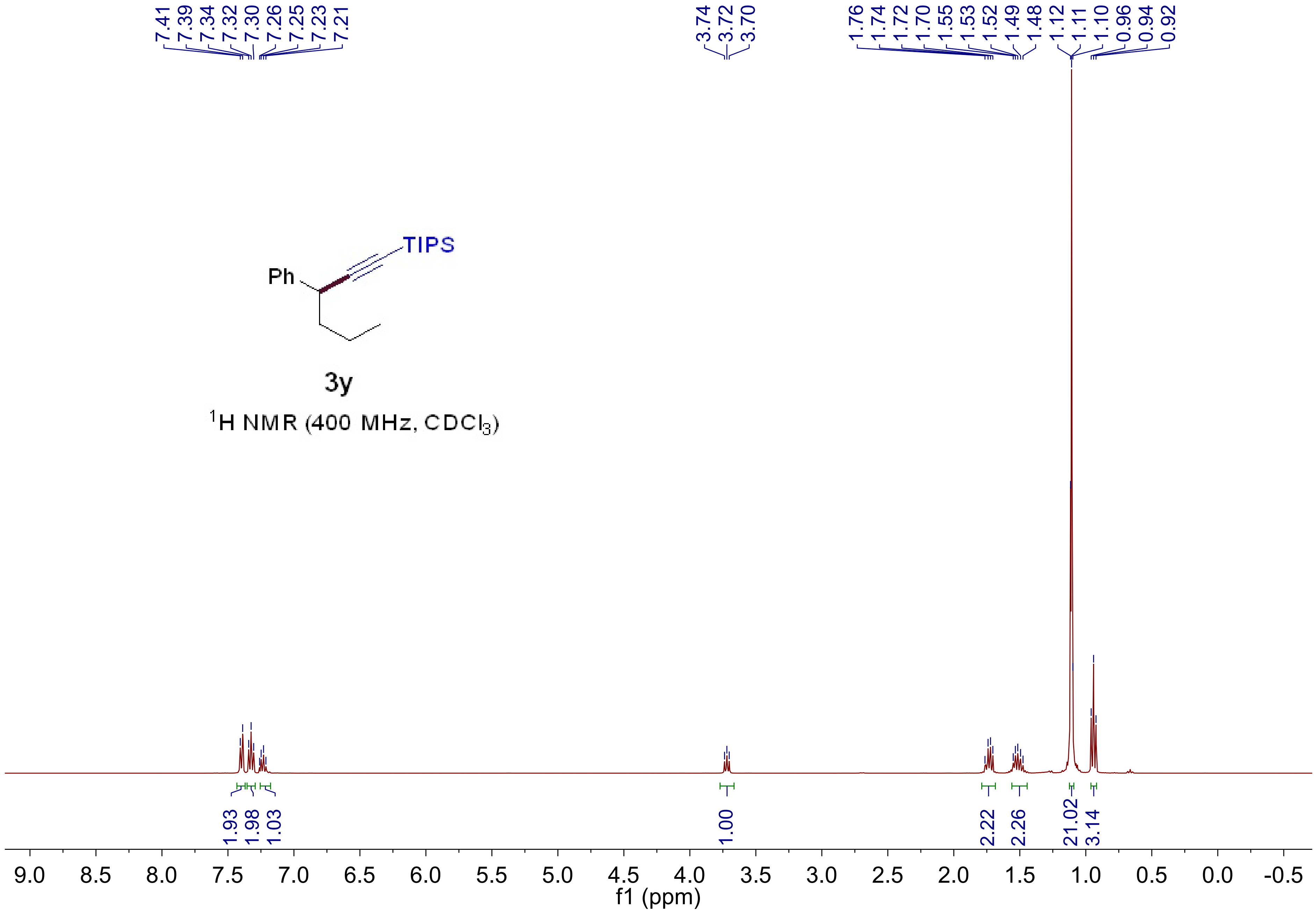

Supplementary Fig. 80. ^{1}H NMR (400 MHz, $CDCl_3$) spectra for compound **3y**

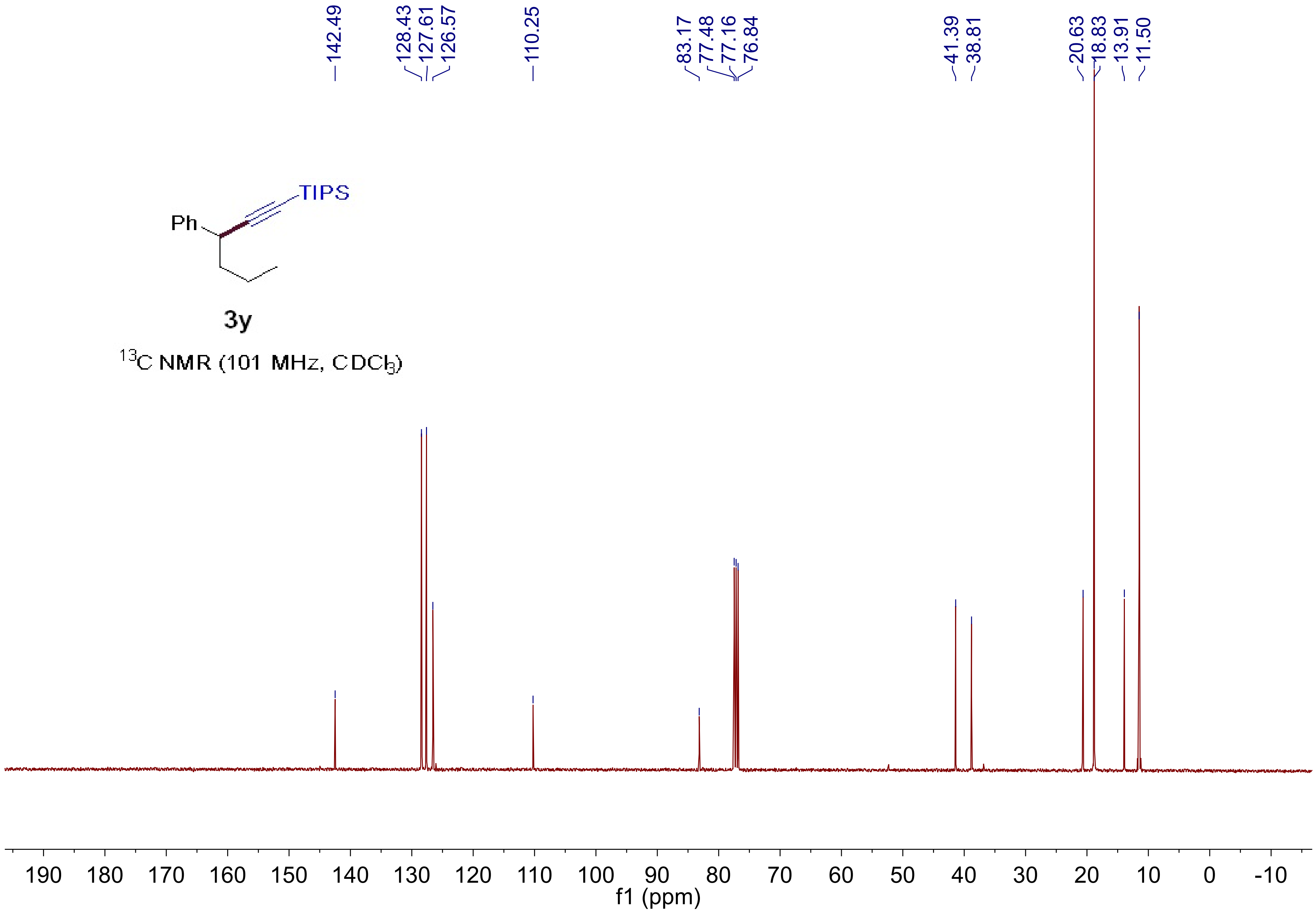

Supplementary Fig. 81. ^{13}C NMR (101 MHz, $CDCl_3$) spectra for compound **3y**

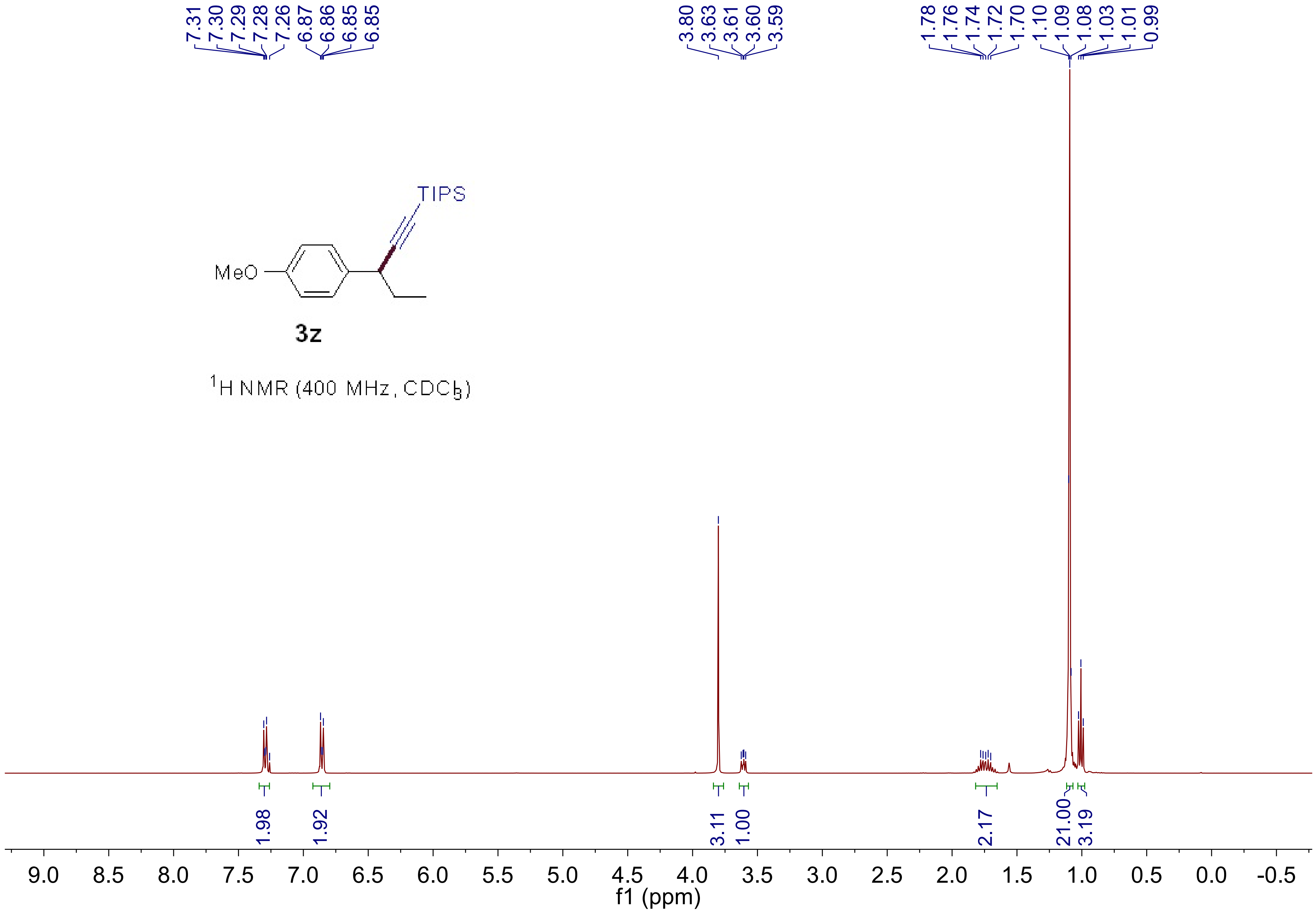

Supplementary Fig. 82. ^{1}H NMR (400 MHz, $CDCl_3$) spectra for compound **3z**

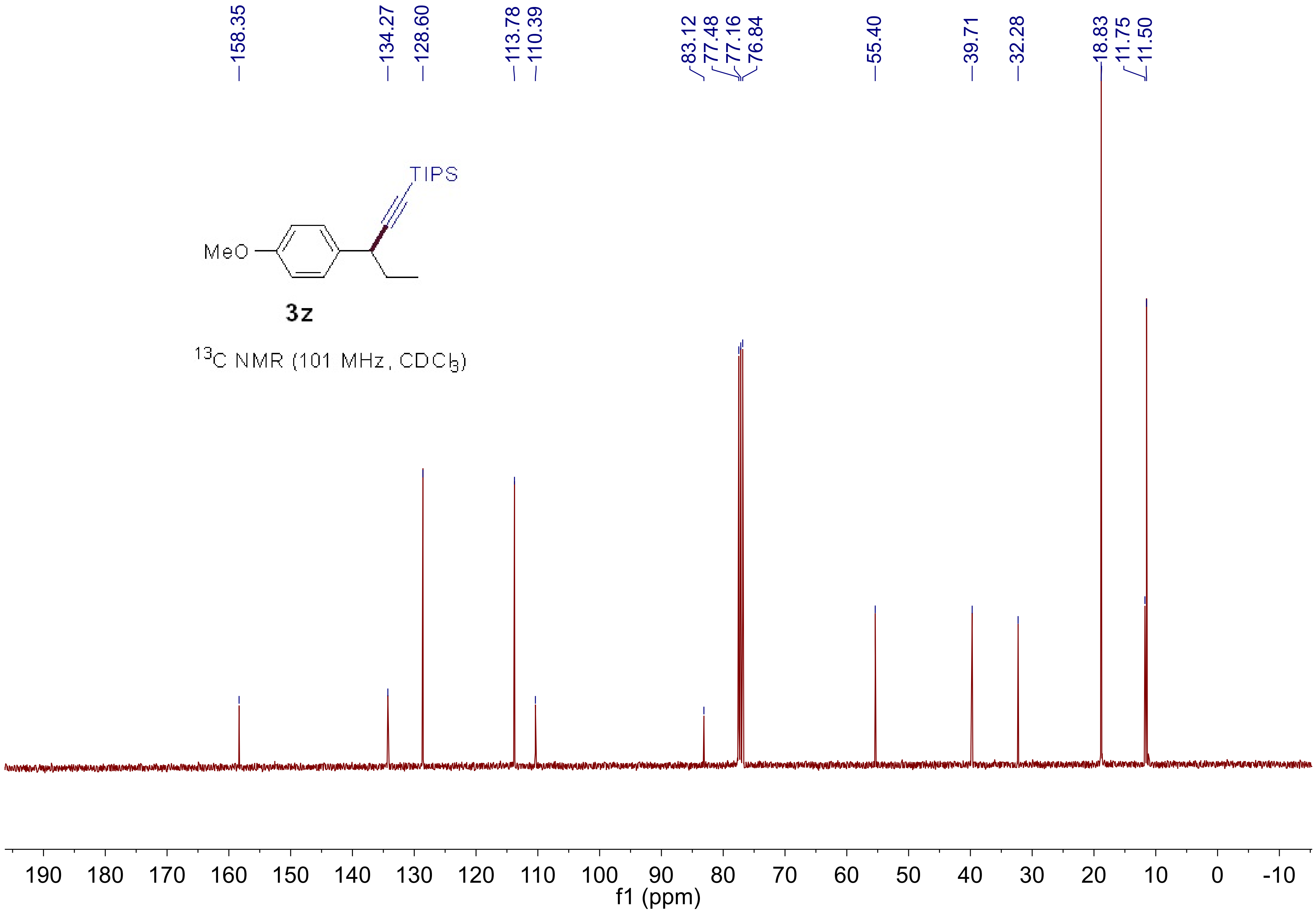

Supplementary Fig. 83. ^{13}C NMR (101 MHz, $CDCl_3$) spectra for compound **3z**

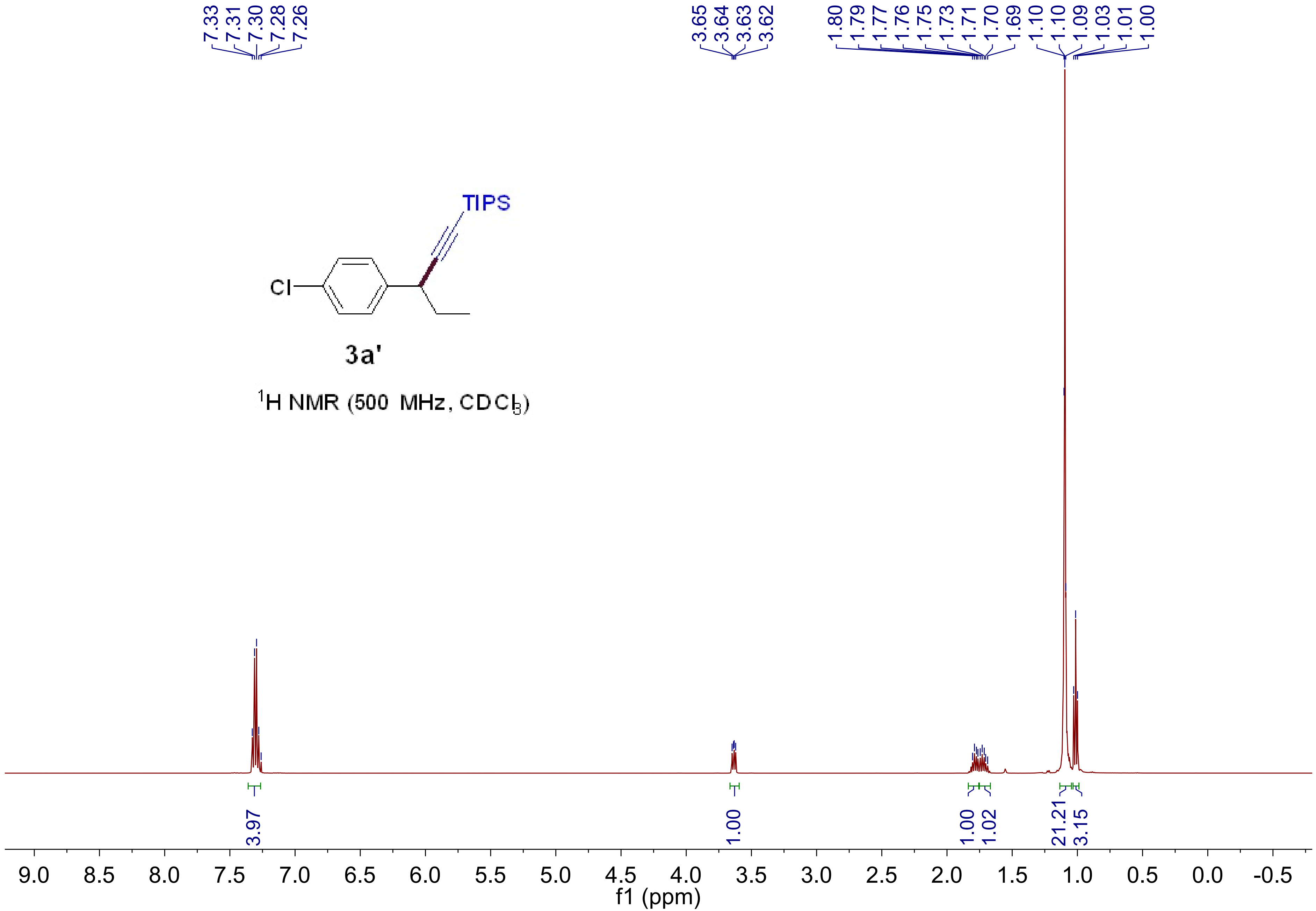

Supplementary Fig. 84. ^{1}H NMR (500 MHz, $CDCl_3$) spectra for compound **3a'**

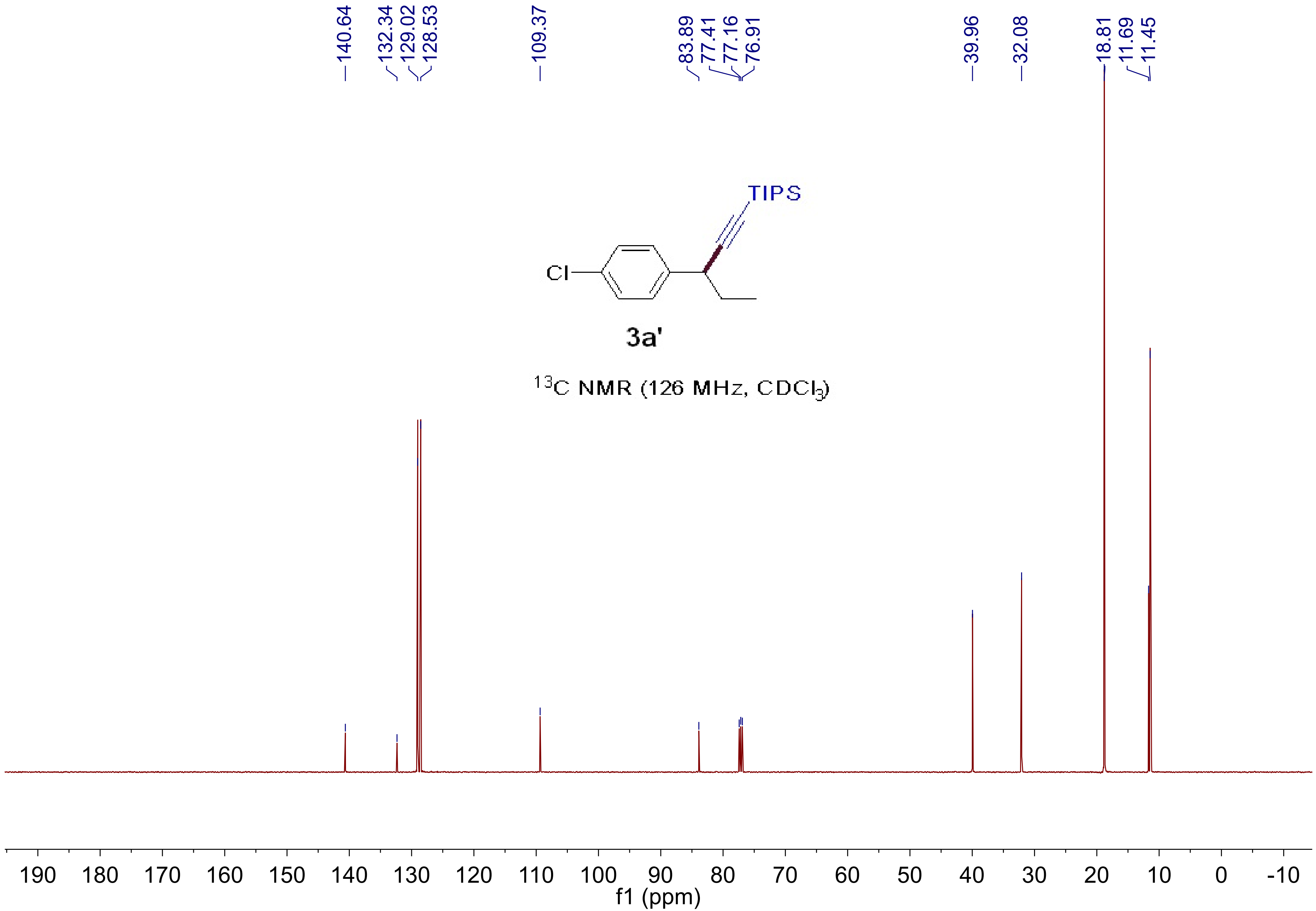

Supplementary Fig. 85. ^{13}C NMR (126 MHz, $CDCl_3$) spectra for compound **3a'**

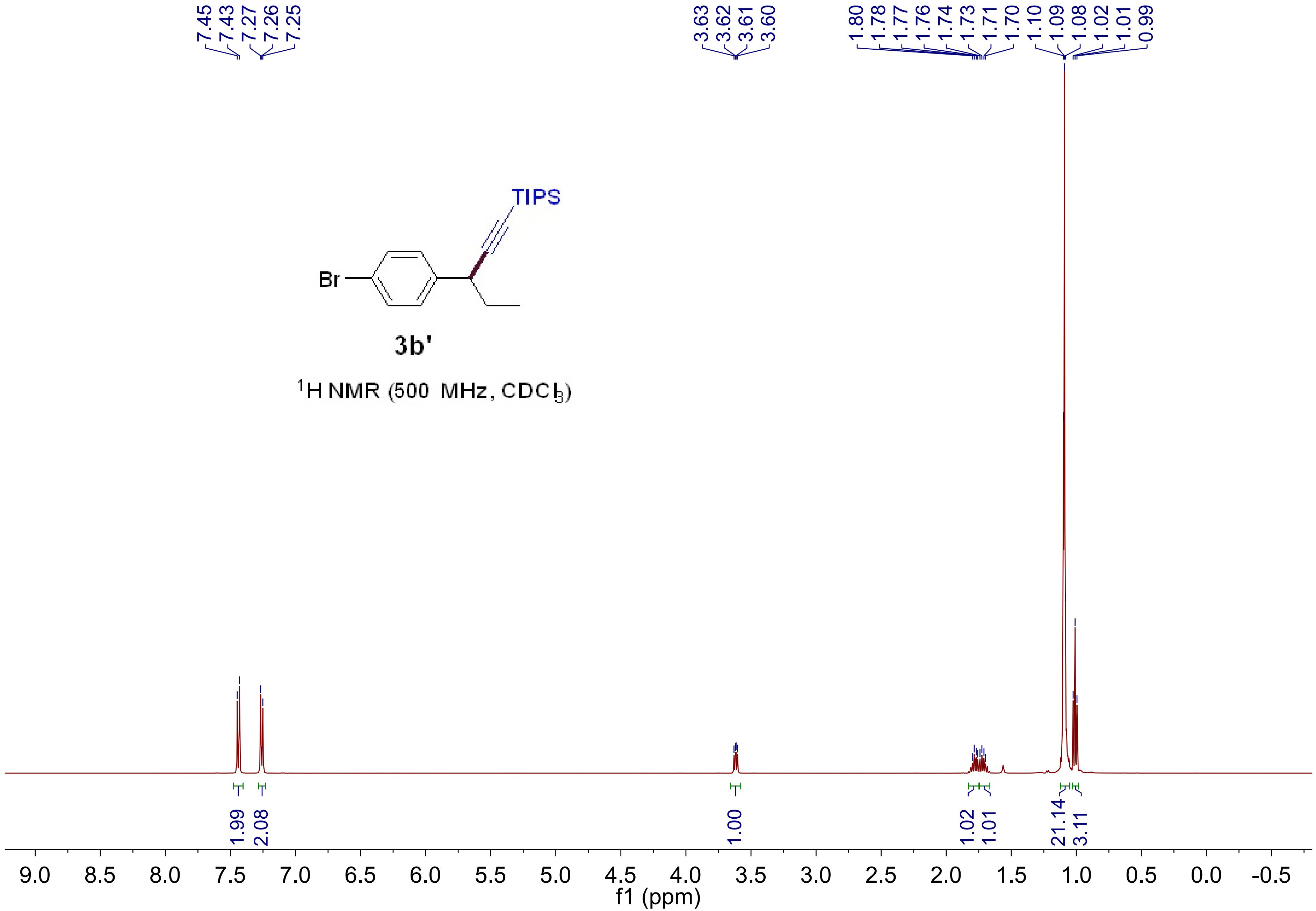

Supplementary Fig. 86. ^{1}H NMR (500 MHz, $CDCl_3$) spectra for compound **3b'**

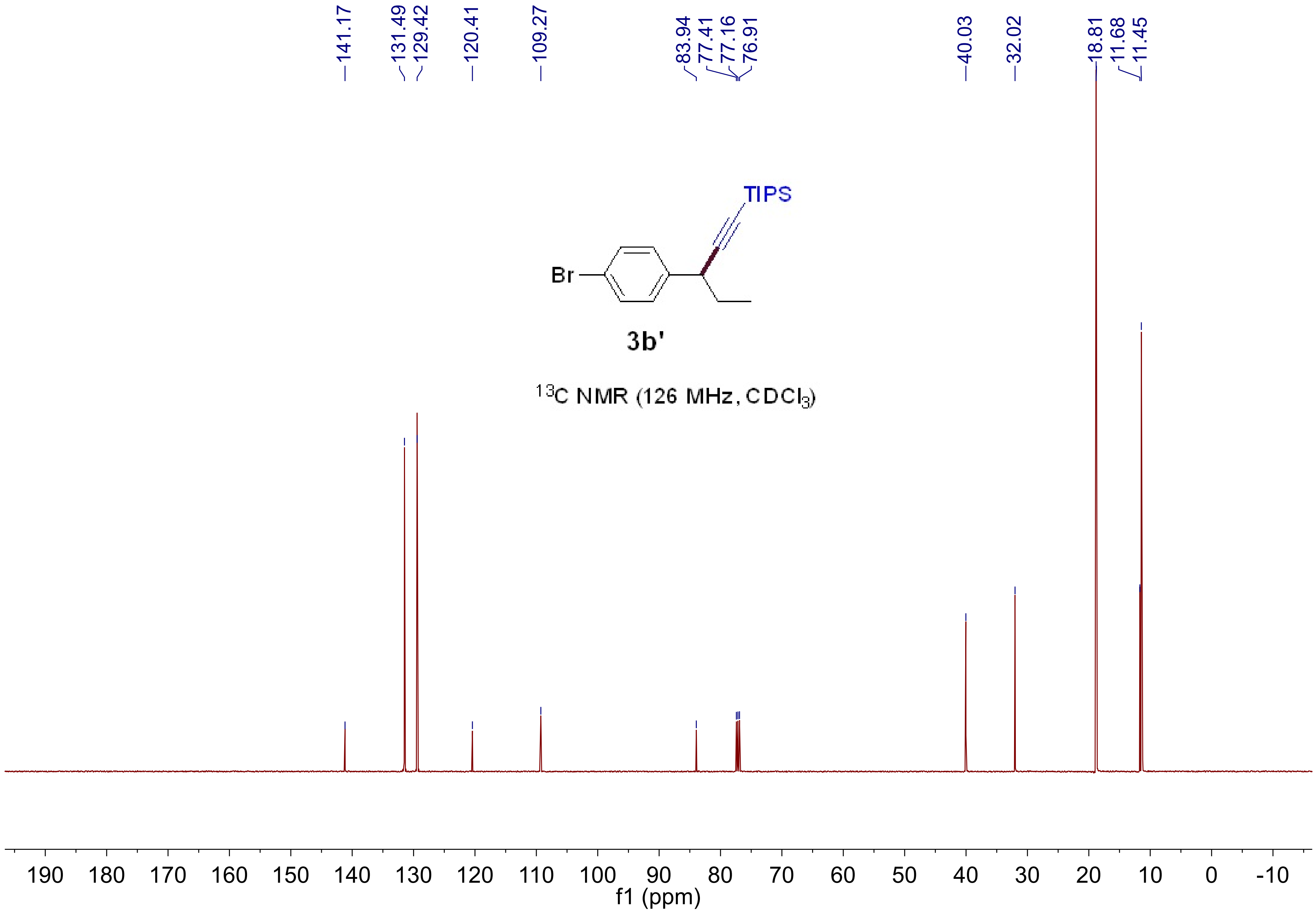

Supplementary Fig. 87. ^{13}C NMR (126 MHz, $CDCl_3$) spectra for compound **3b'**

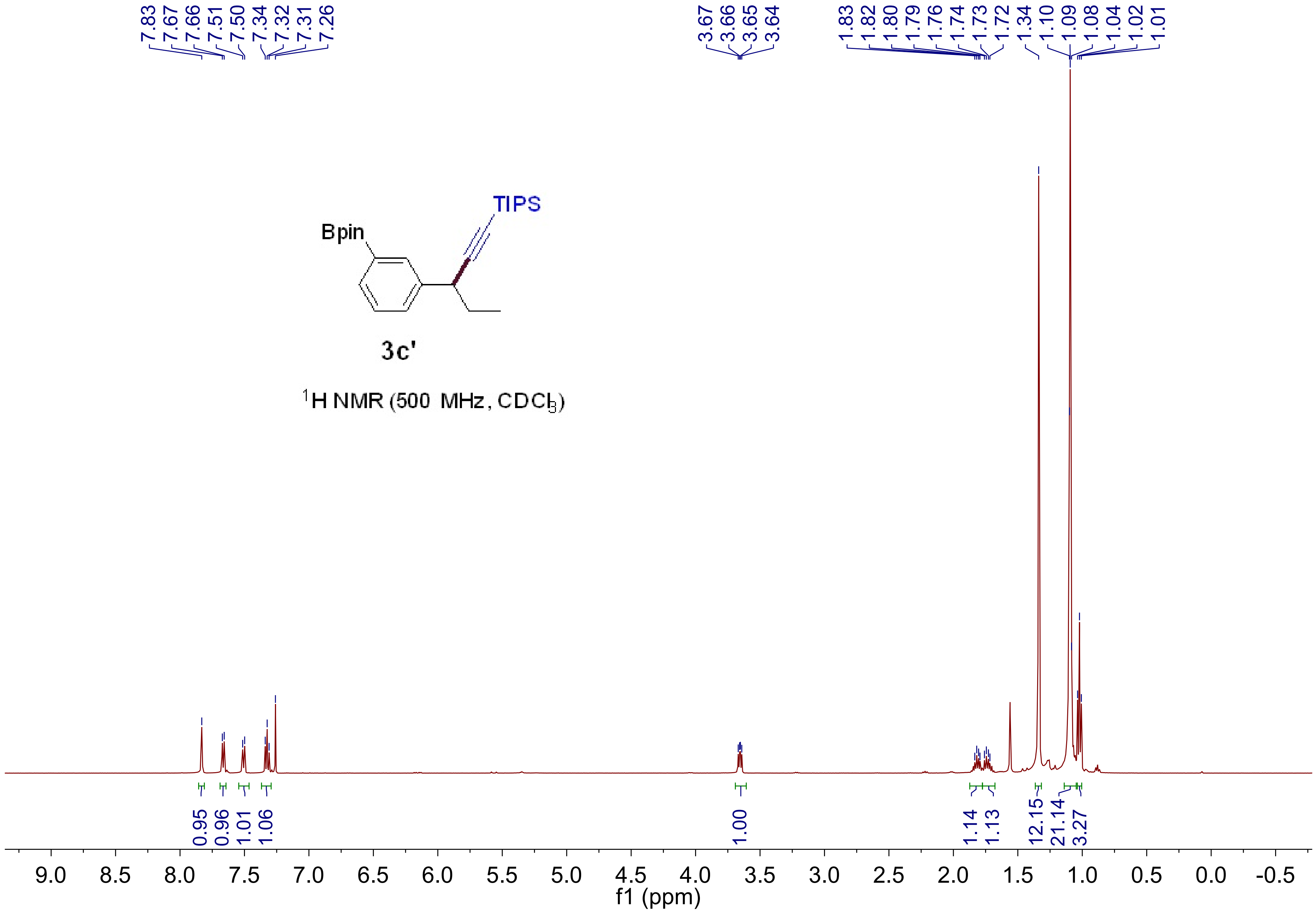

Supplementary Fig. 88. ^{1}H NMR (500 MHz, $CDCl_3$) spectra for compound **3c'**

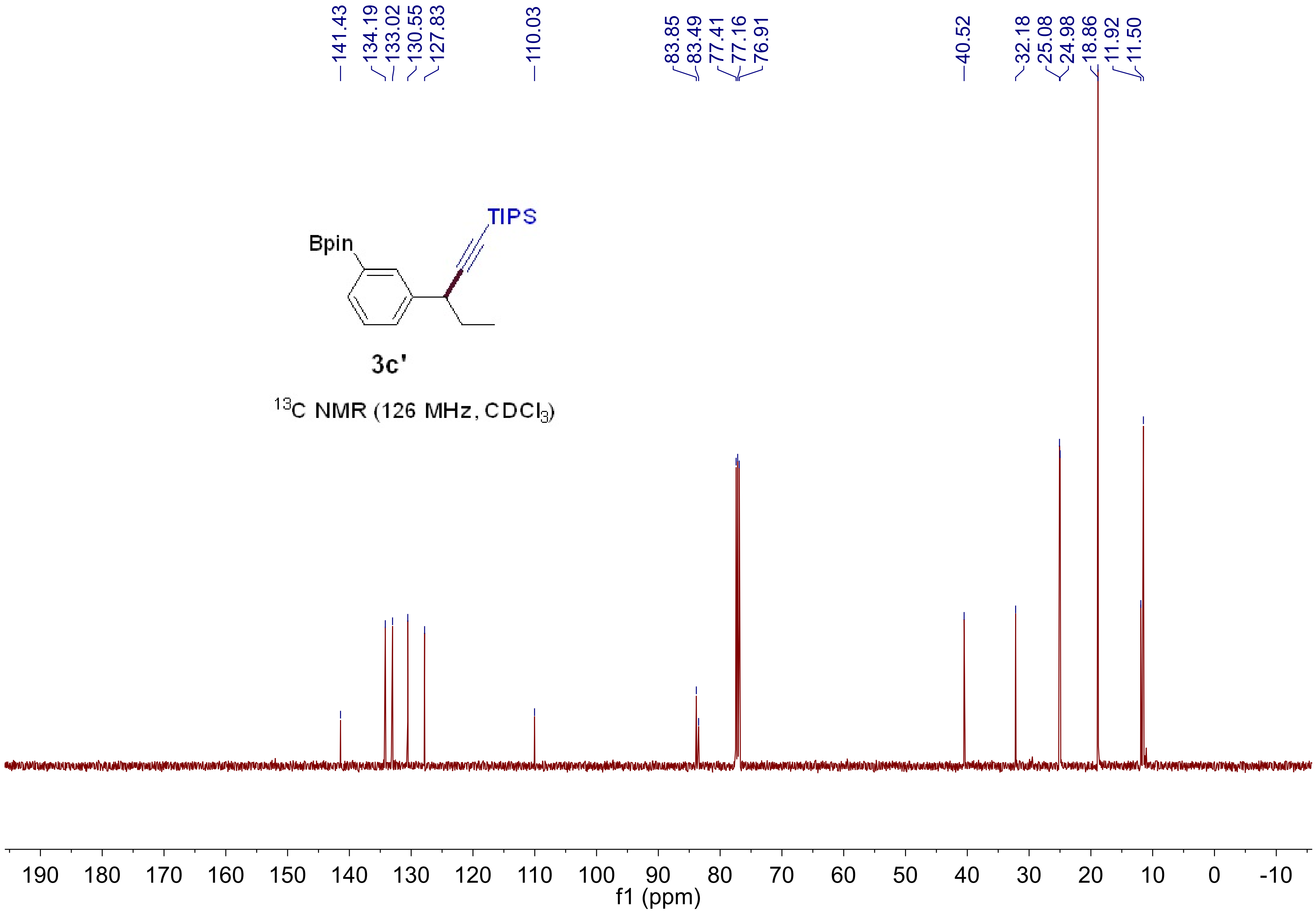

Supplementary Fig. 89. ^{13}C NMR (126 MHz, $CDCl_3$) spectra for compound **3c'**

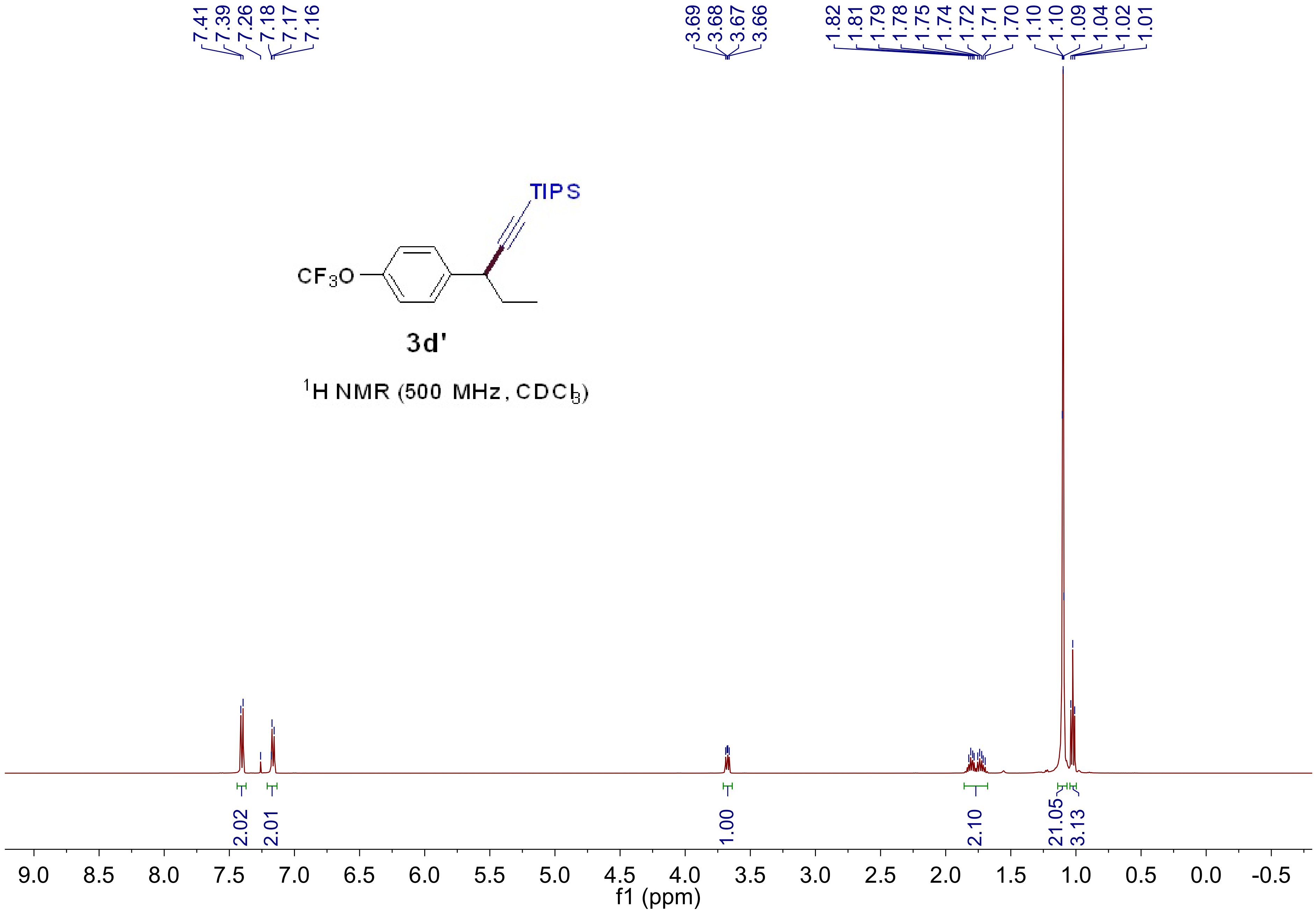

Supplementary Fig. 90. ^{1}H NMR (500 MHz, $CDCl_3$) spectra for compound **3d'**

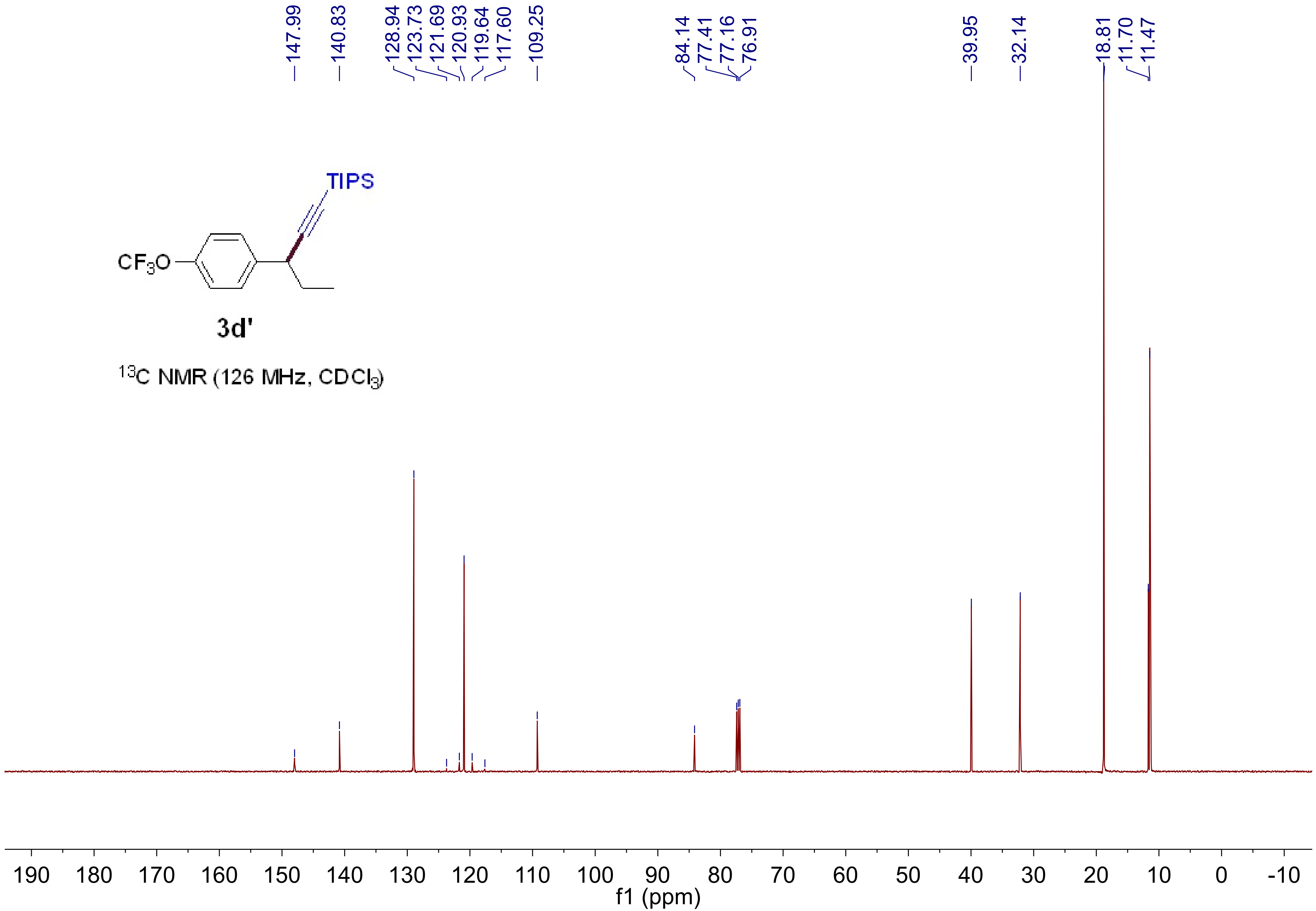

Supplementary Fig. 91. ^{13}C NMR (126 MHz, $CDCl_3$) spectra for compound **3d'**

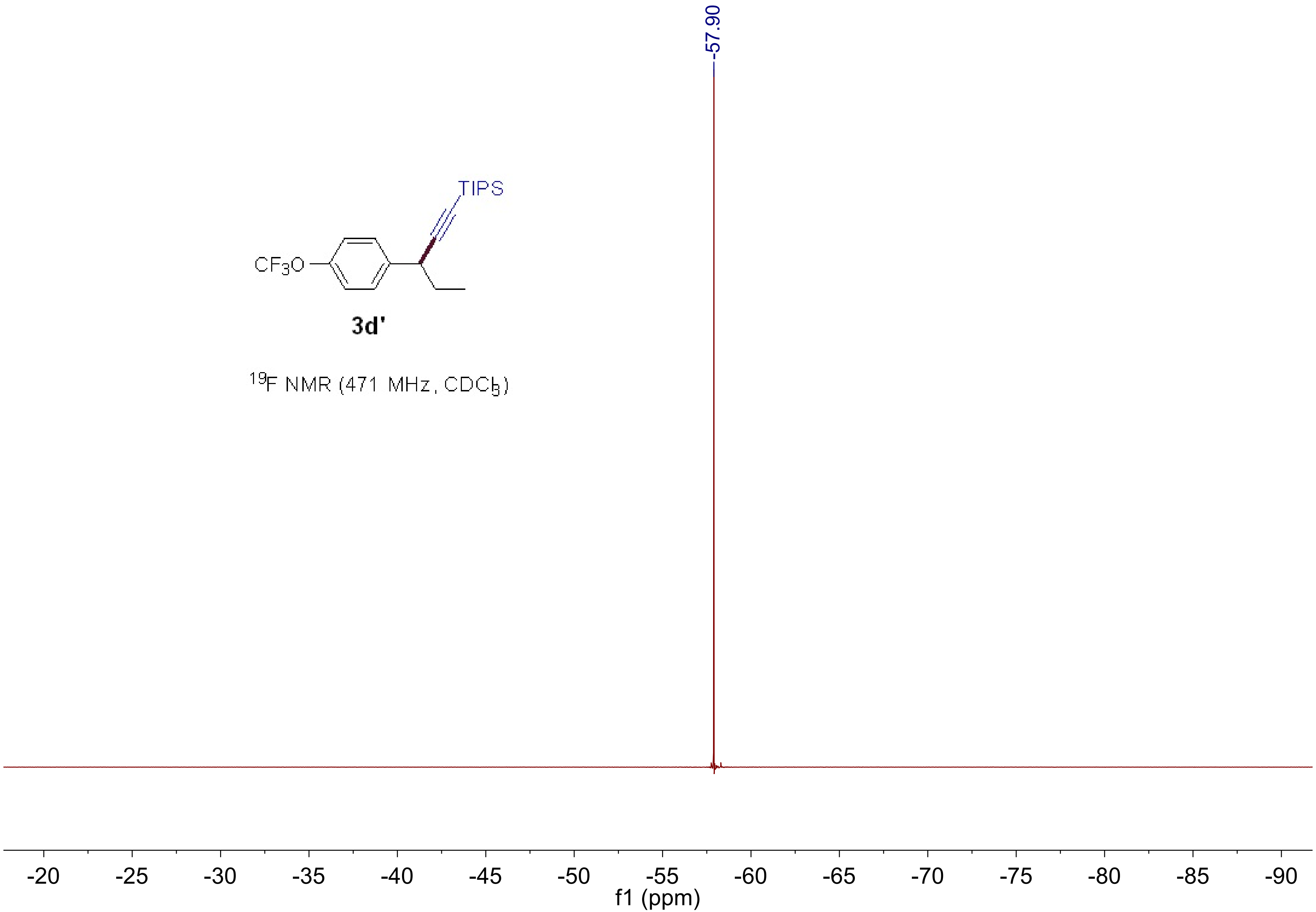

Supplementary Fig. 92. ^{19}F NMR (471 MHz, $CDCl_3$) spectra for compound **3d'**

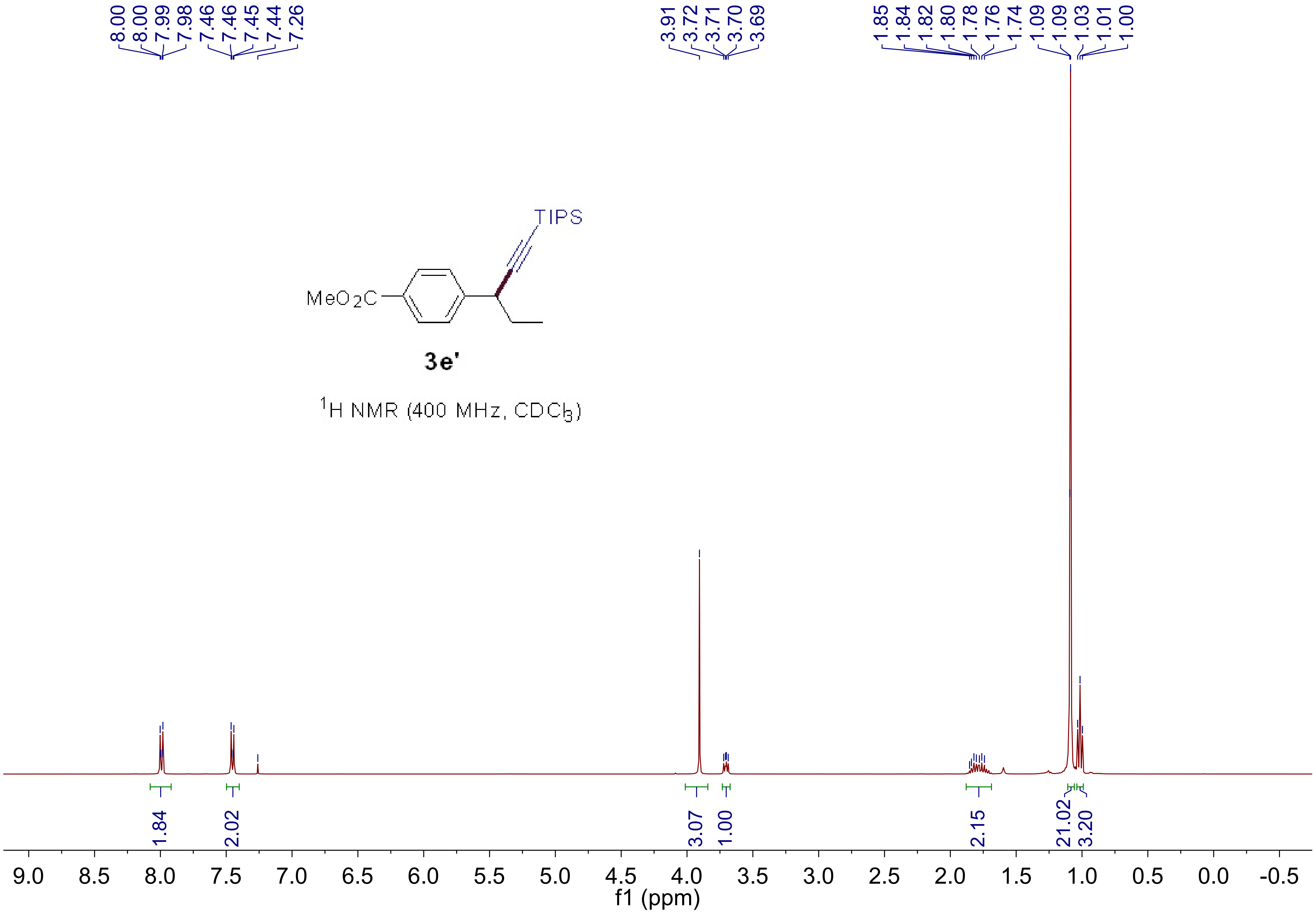

Supplementary Fig. 93. ^{1}H NMR (400 MHz, $CDCl_3$) spectra for compound **3e'**

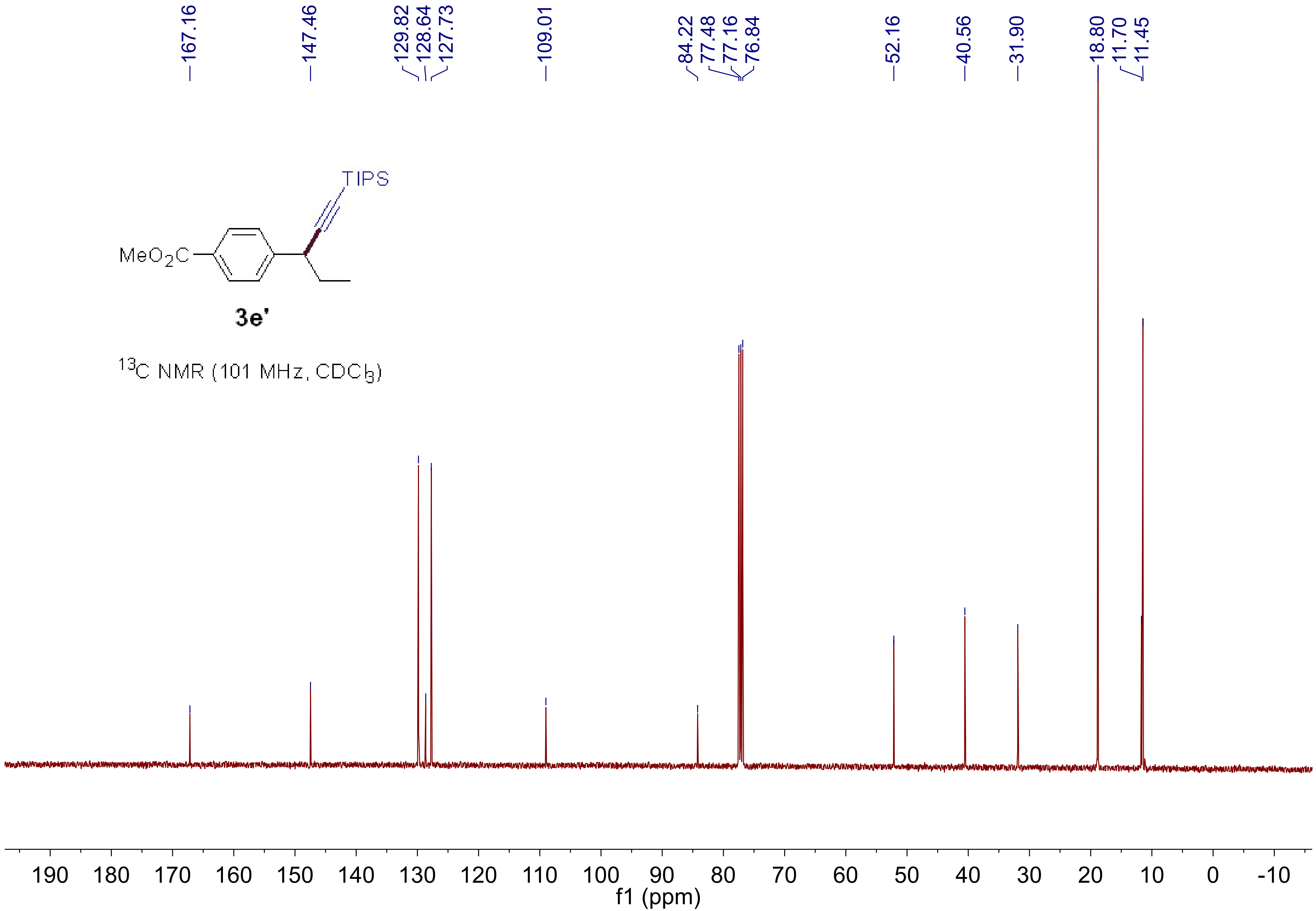

Supplementary Fig. 94. ^{13}C NMR (101 MHz, $CDCl_3$) spectra for compound **3e'**

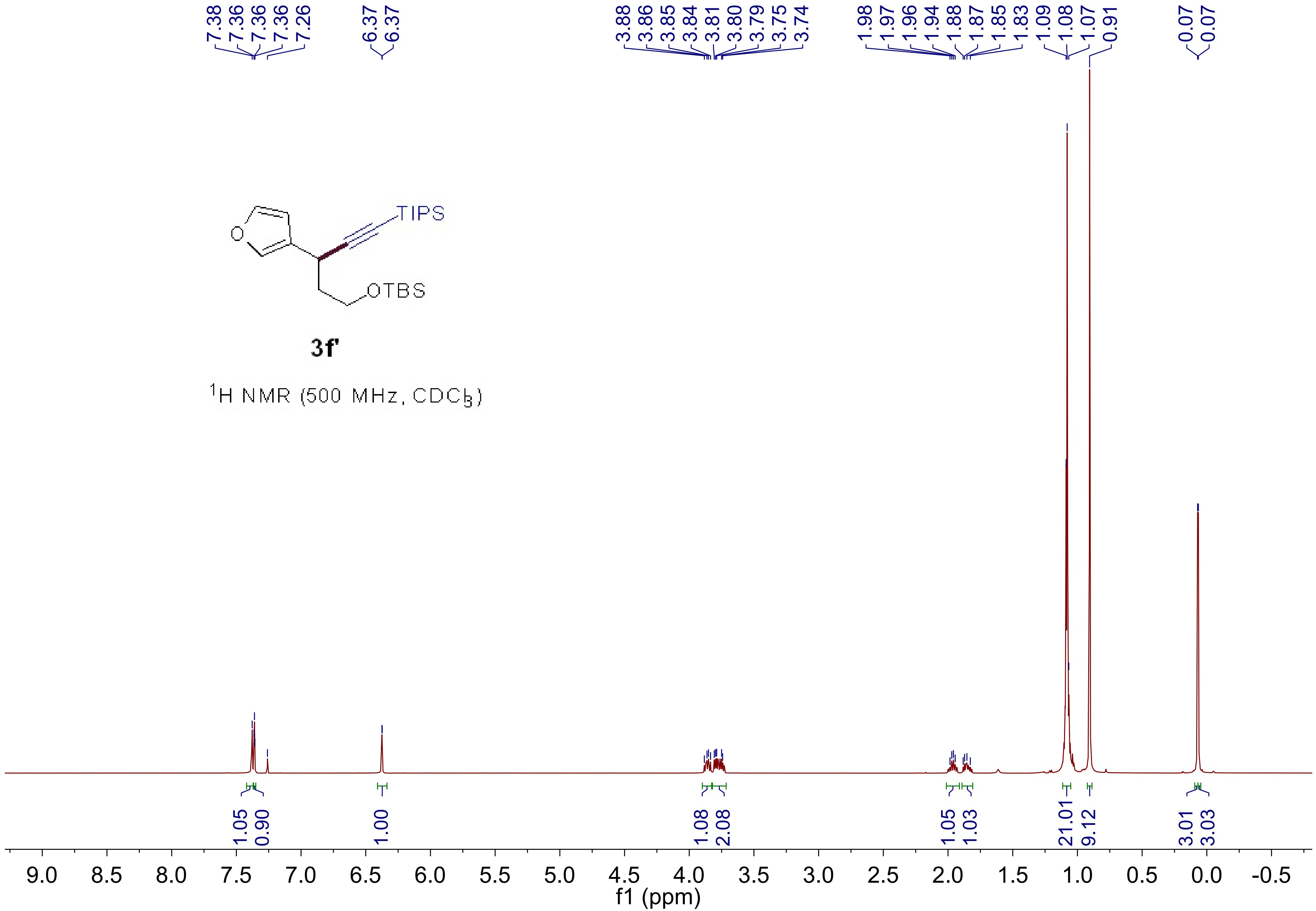

Supplementary Fig. 95. ^{1}H NMR (500 MHz, $CDCl_3$) spectra for compound **3f'**

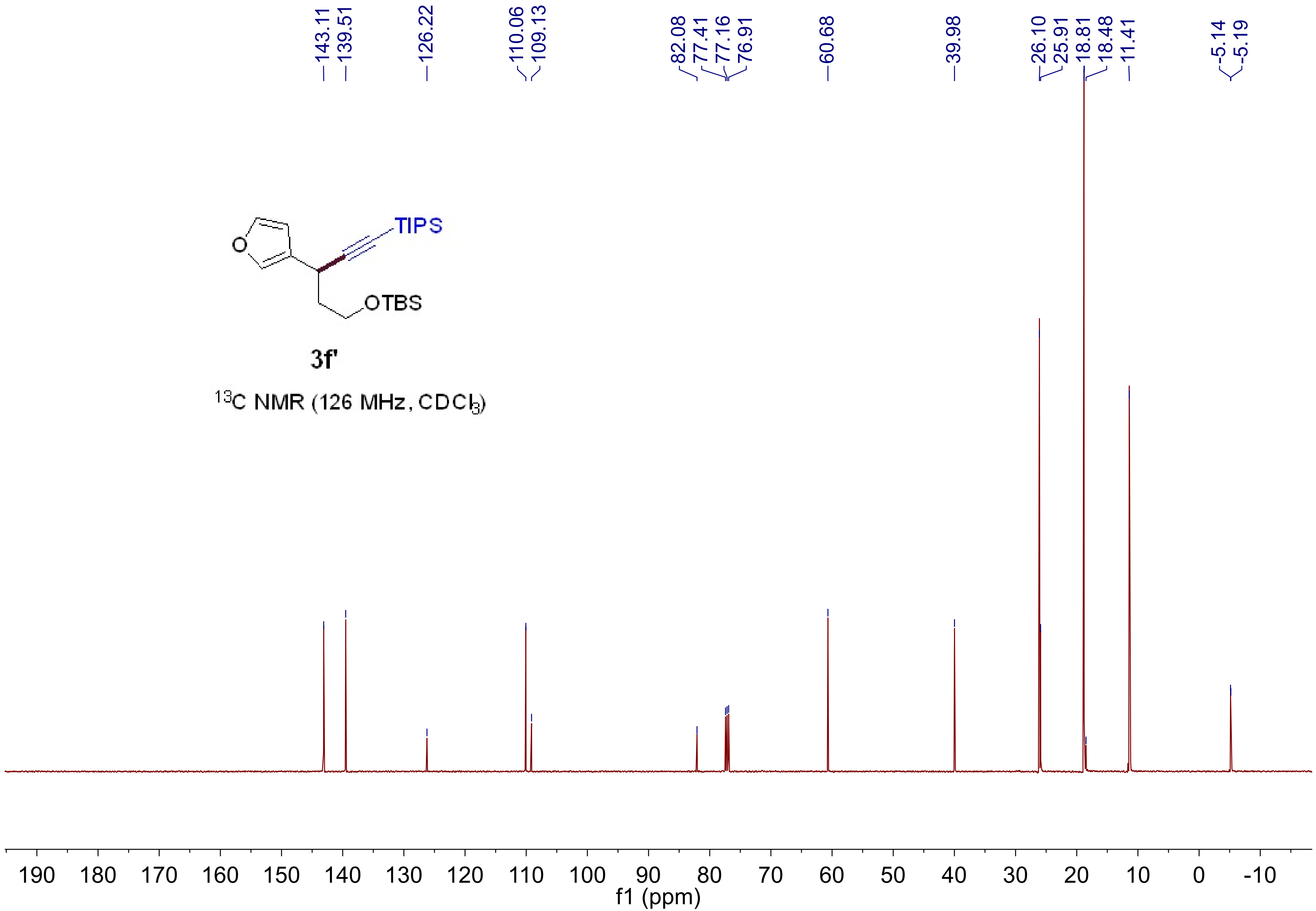

Supplementary Fig. 96. ^{13}C NMR (126 MHz, $CDCl_3$) spectra for compound **3f'**

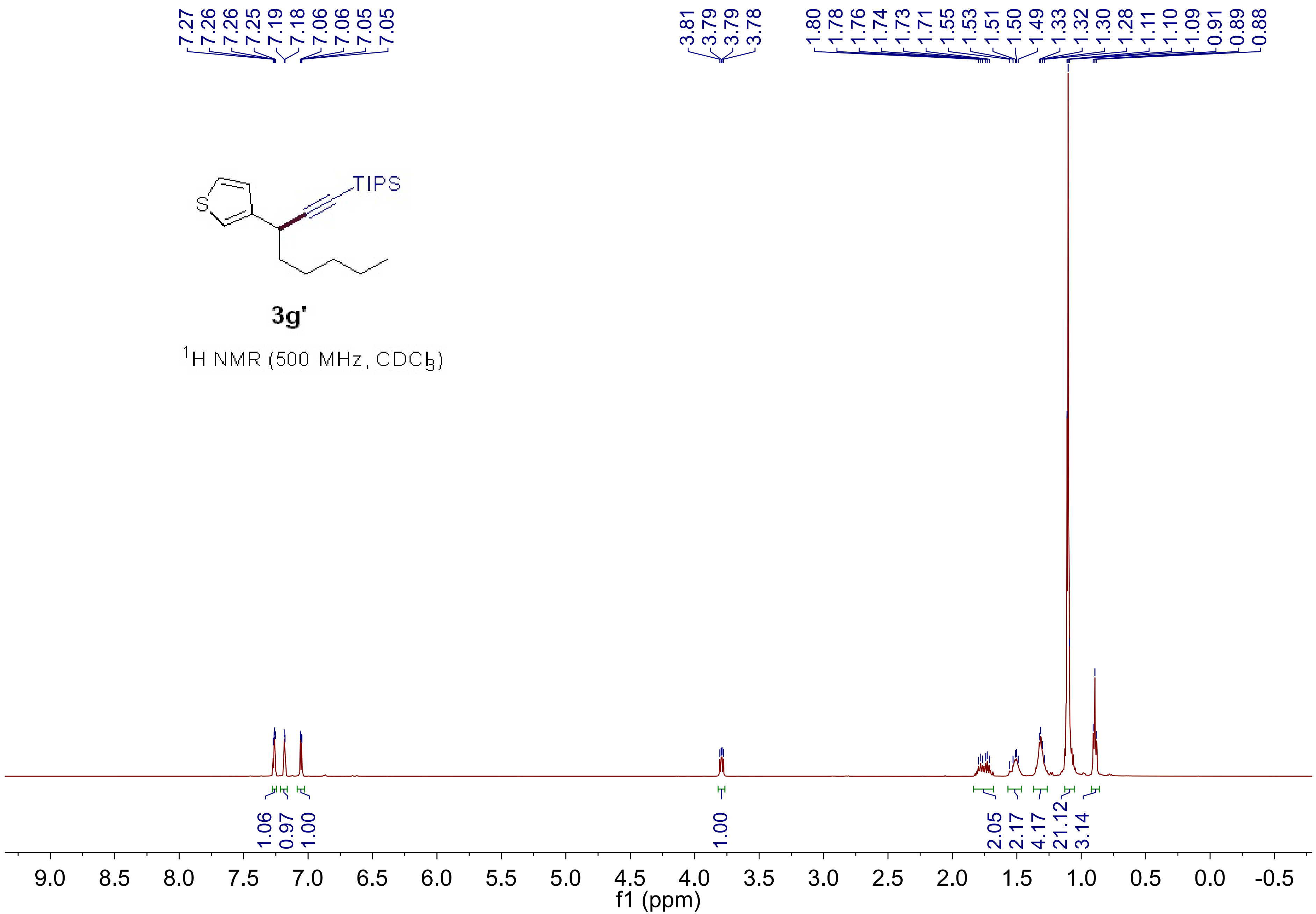

Supplementary Fig. 97. ^{1}H NMR (500 MHz, $CDCl_3$) spectra for compound **3g'**

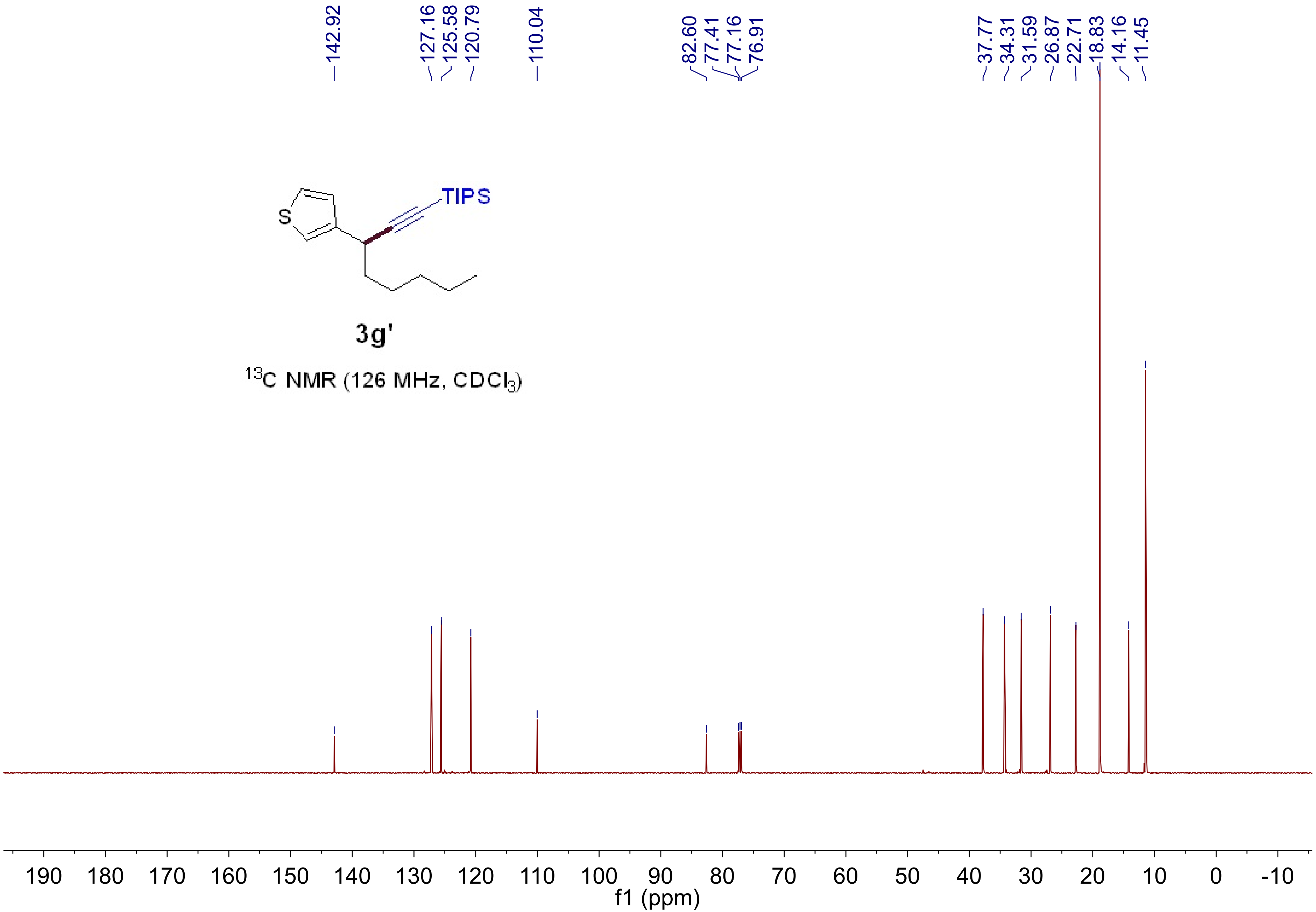

Supplementary Fig. 98. ^{13}C NMR (126 MHz, $CDCl_3$) spectra for compound **3g'**

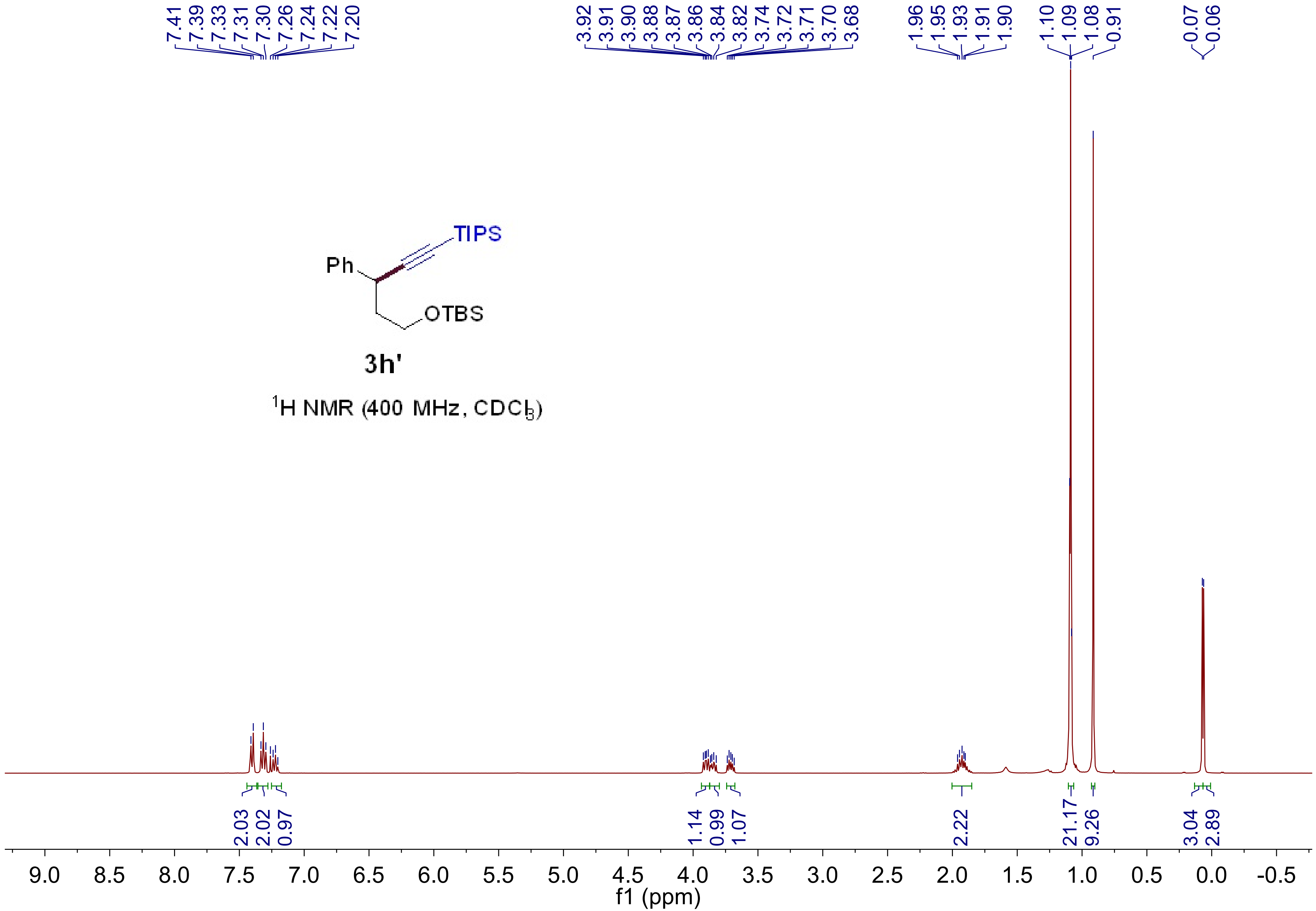

Supplementary Fig. 99. ^{1}H NMR (400 MHz, $CDCl_3$) spectra for compound **3h'**

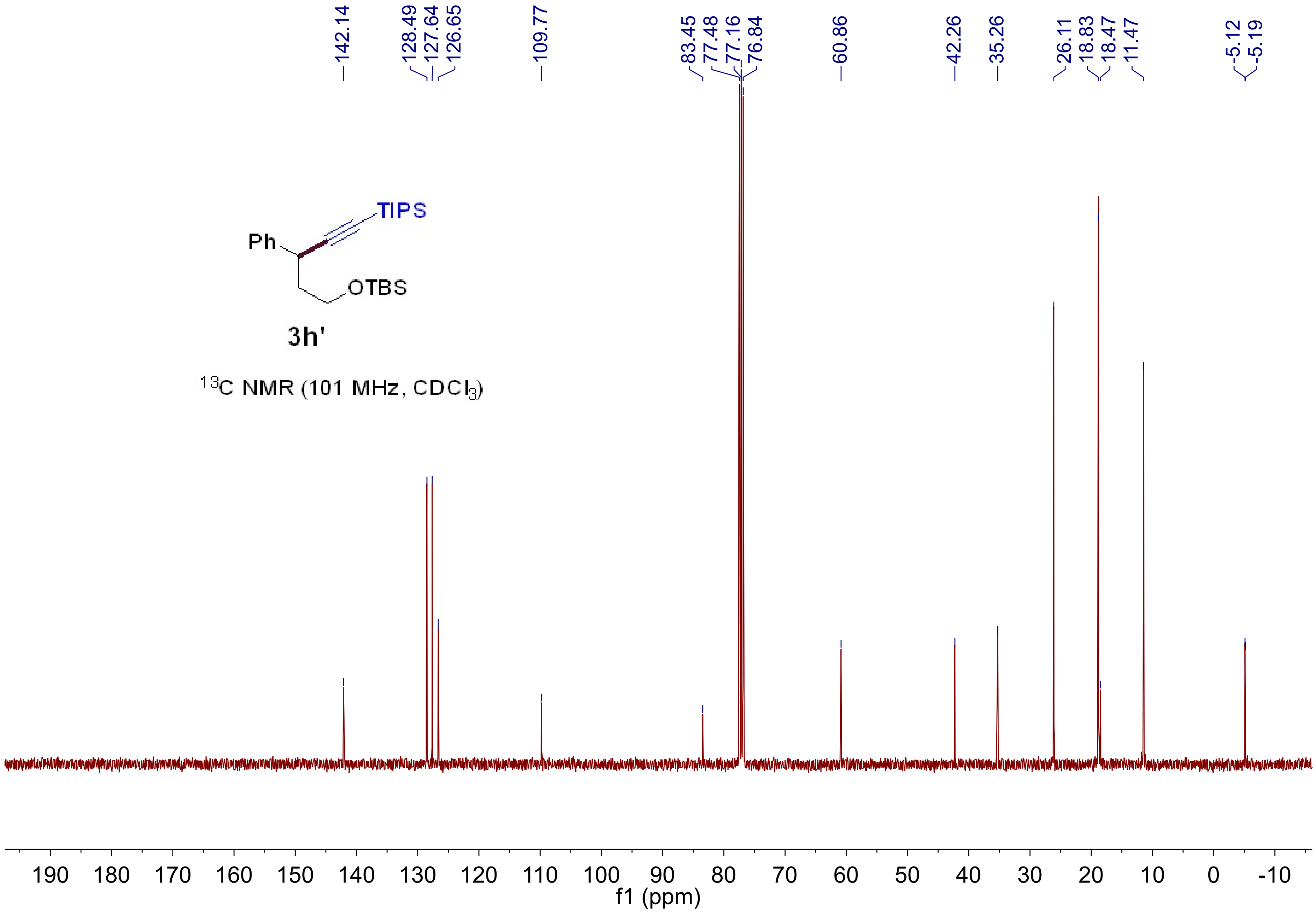

Supplementary Fig. 100. ^{13}C NMR (101 MHz, $CDCl_3$) spectra for compound **3h'**

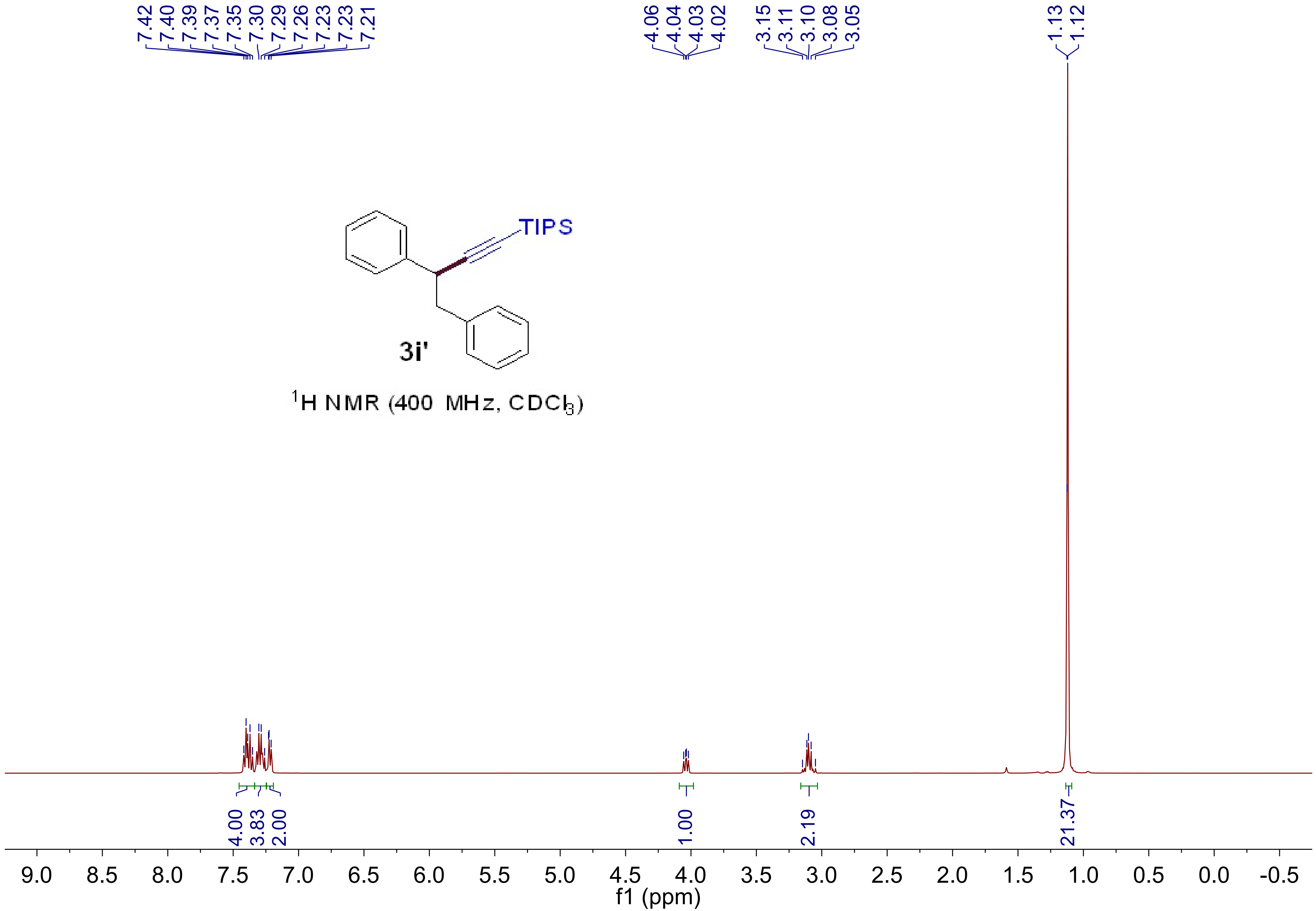

Supplementary Fig. 101. ^{1}H NMR (400 MHz, $CDCl_3$) spectra for compound **3i'**

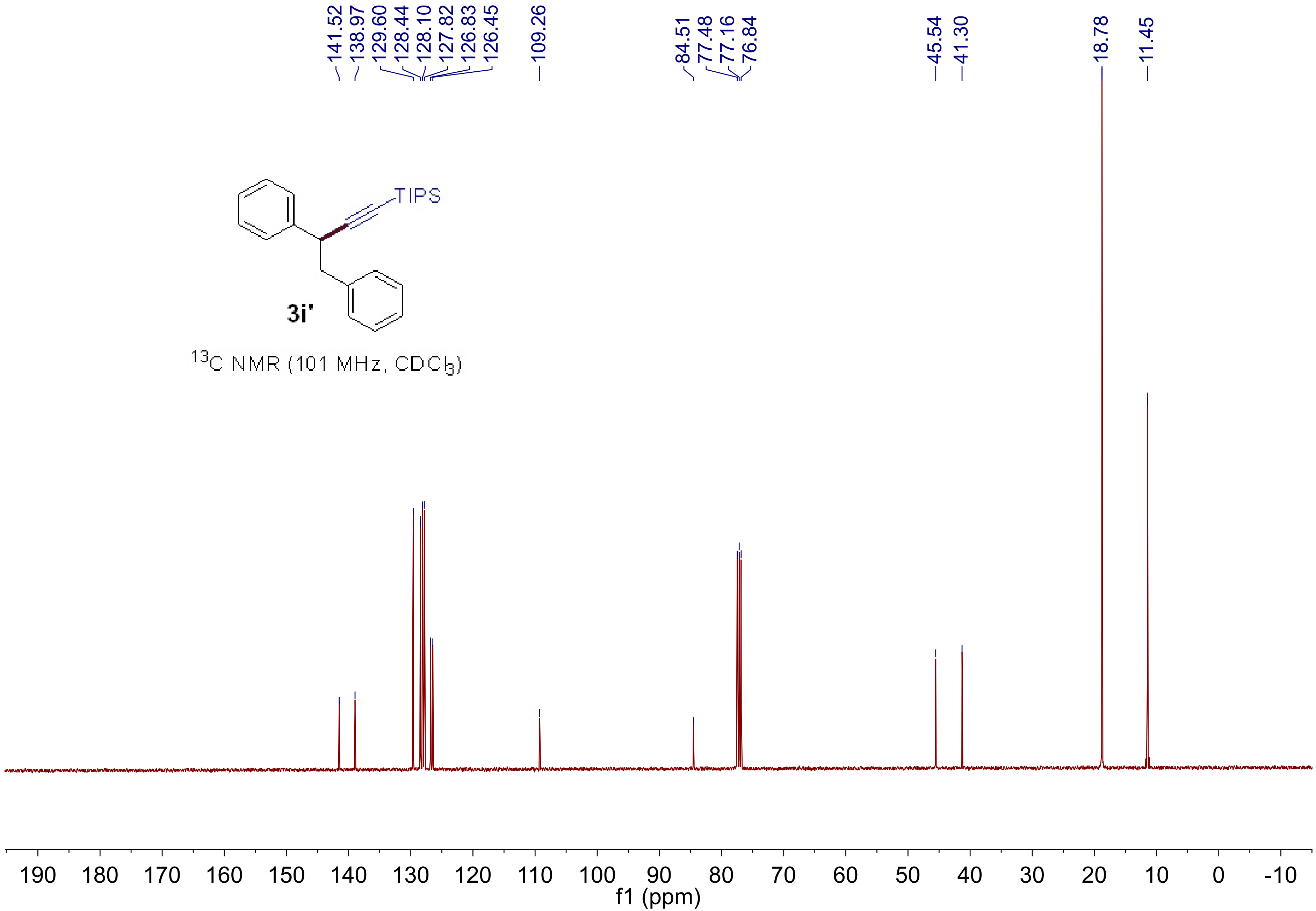

Supplementary Fig. 102. ^{13}C NMR (101 MHz, $CDCl_3$) spectra for compound **3i'**

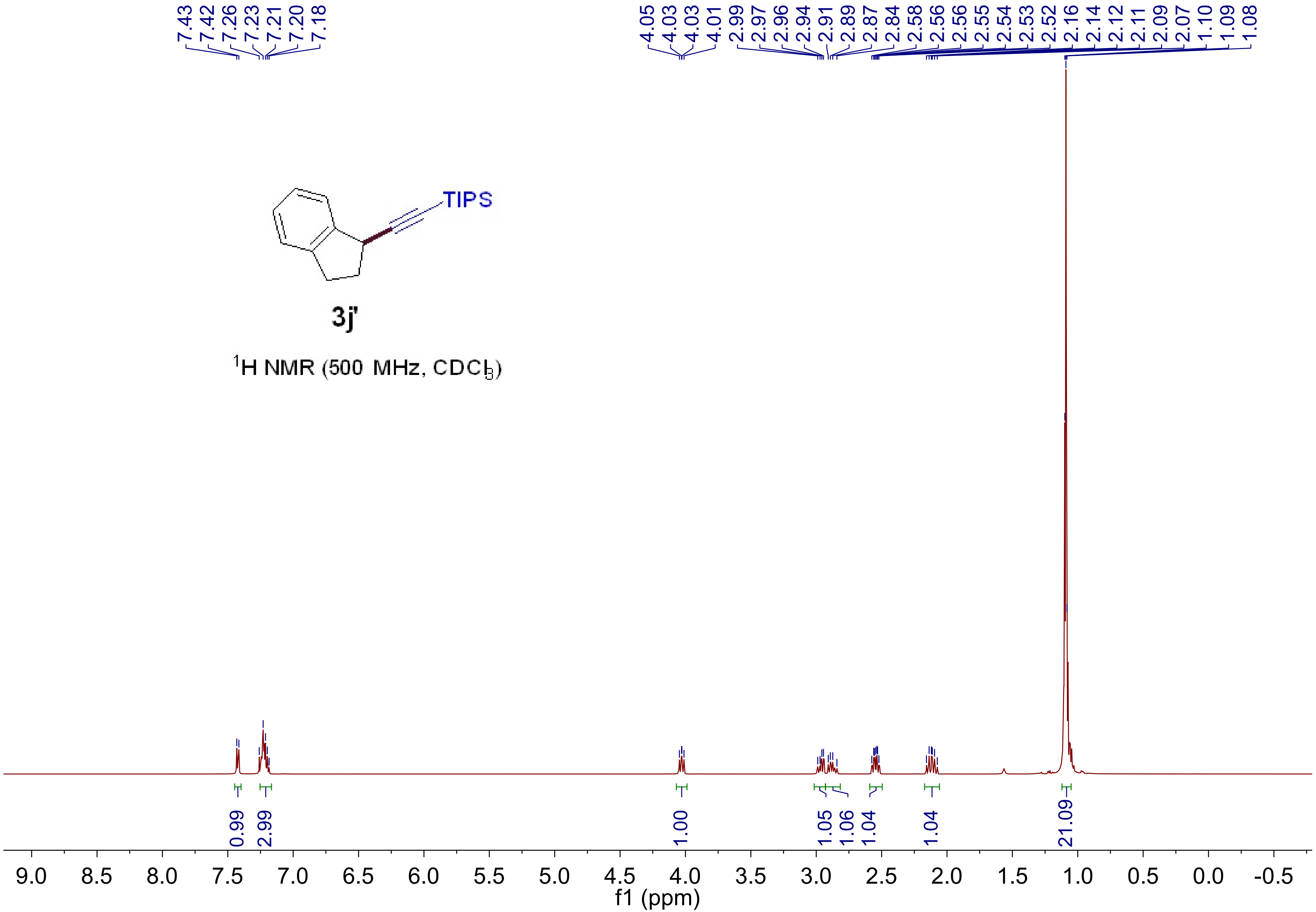

Supplementary Fig. 103. ^{1}H NMR (500 MHz, $CDCl_3$) spectra for compound **3j'**

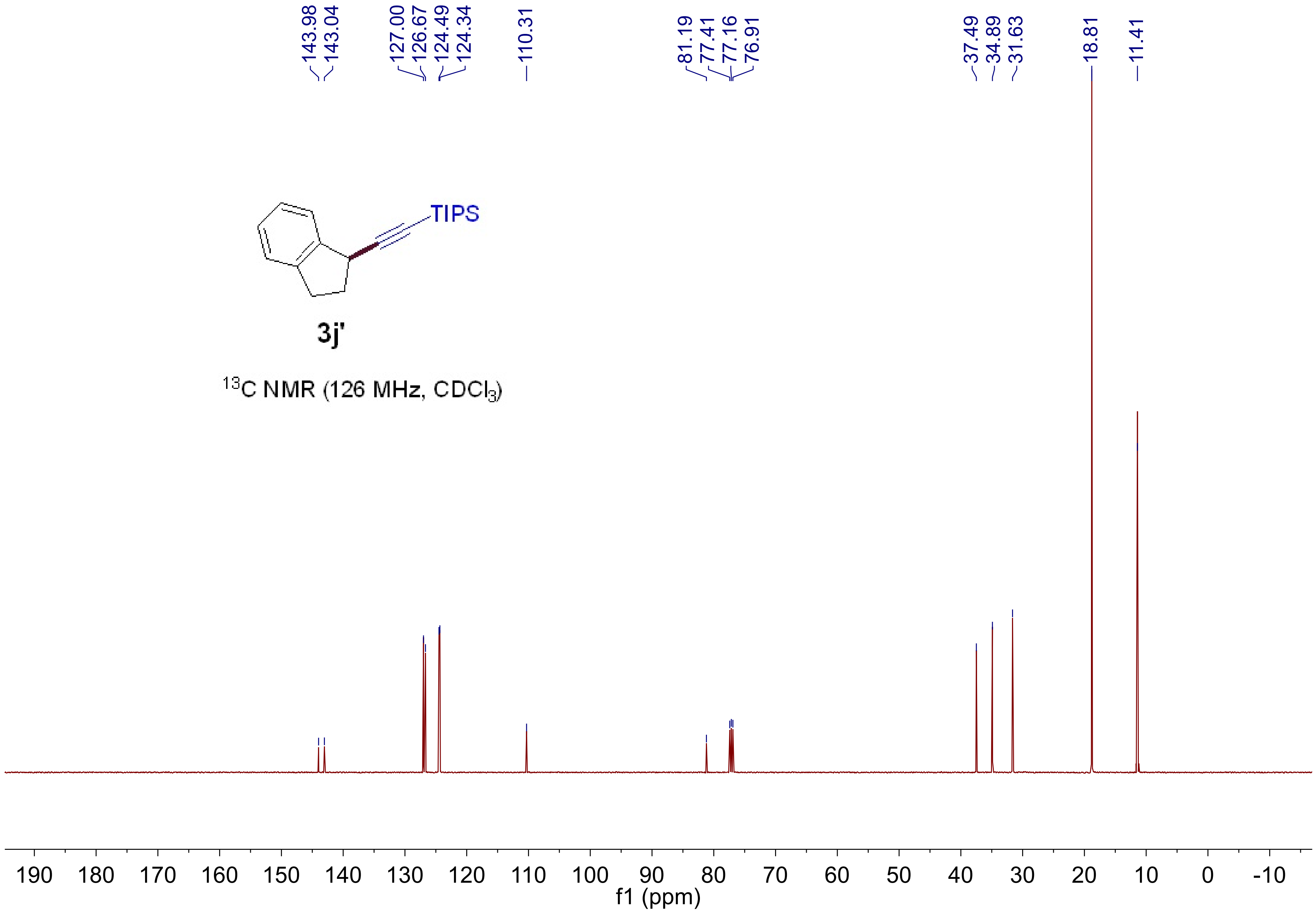

Supplementary Fig. 104. ^{13}C NMR (126 MHz, $CDCl_3$) spectra for compound **3j'**

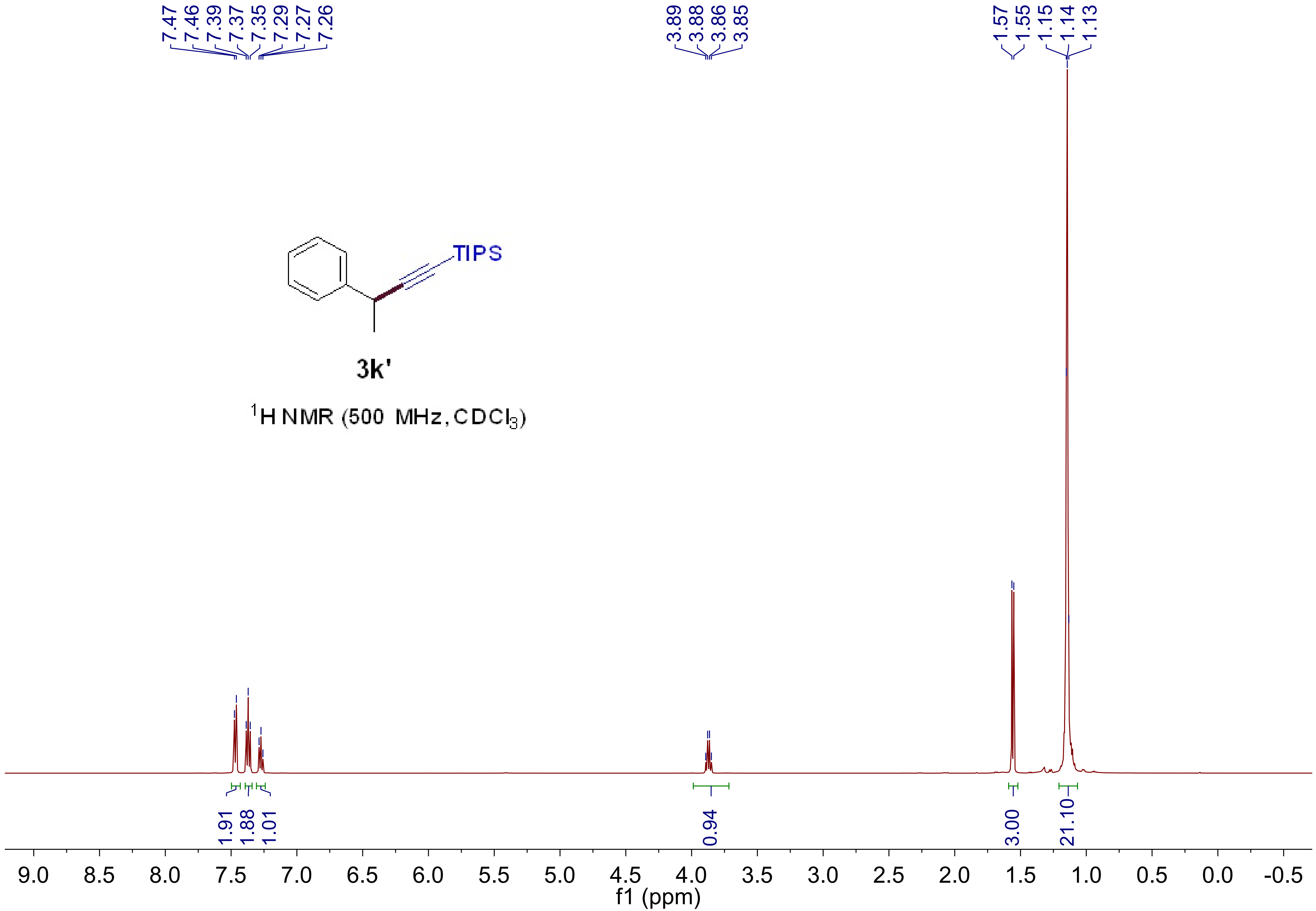

Supplementary Fig. 105. ^{1}H NMR (500 MHz, $CDCl_3$) spectra for compound **3k'**

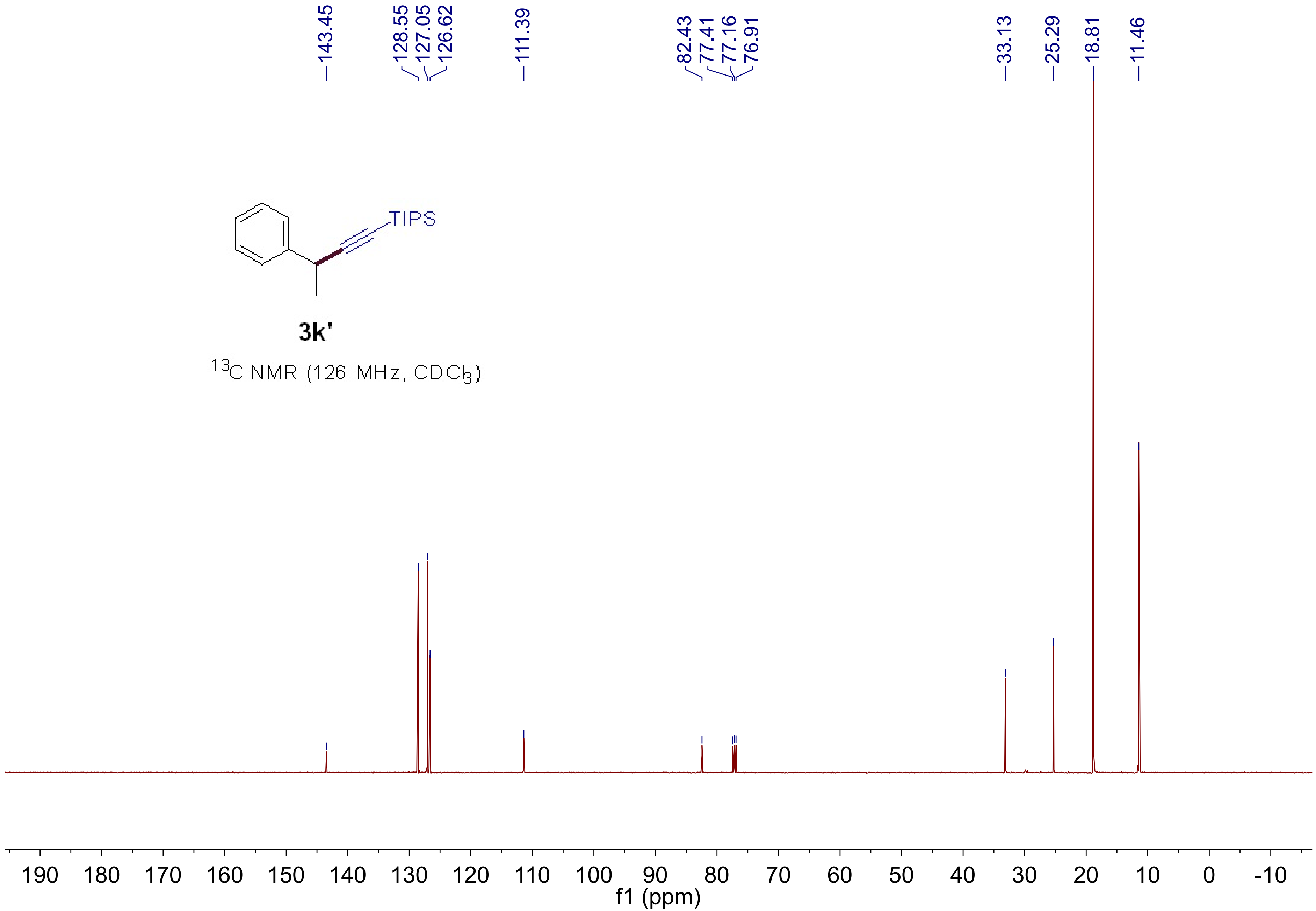

Supplementary Fig. 106. ^{13}C NMR (126 MHz, $CDCl_3$) spectra for compound **3k'**

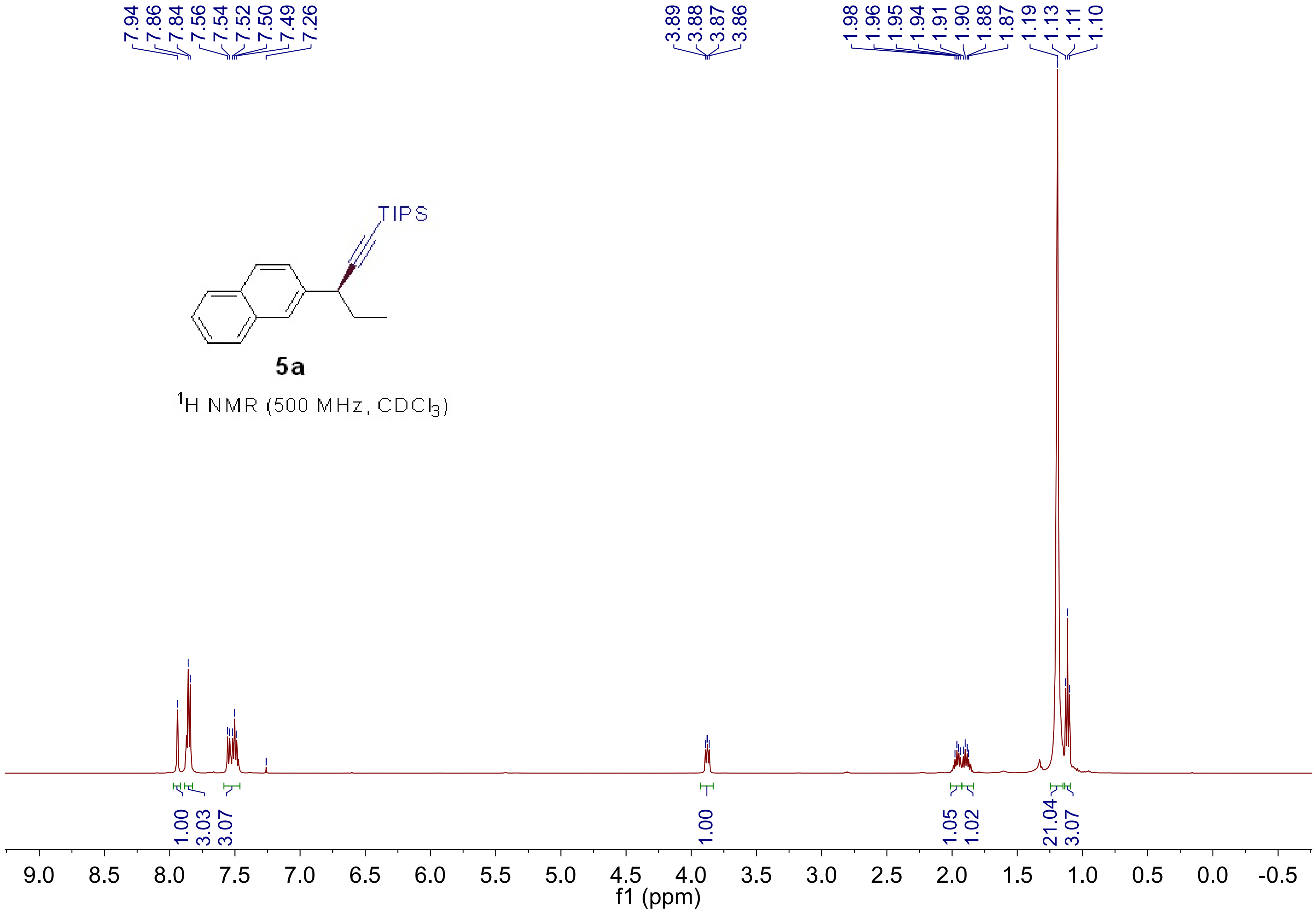

Supplementary Fig. 107. ^{1}H NMR (500 MHz, $CDCl_3$) spectra for compound **5a**

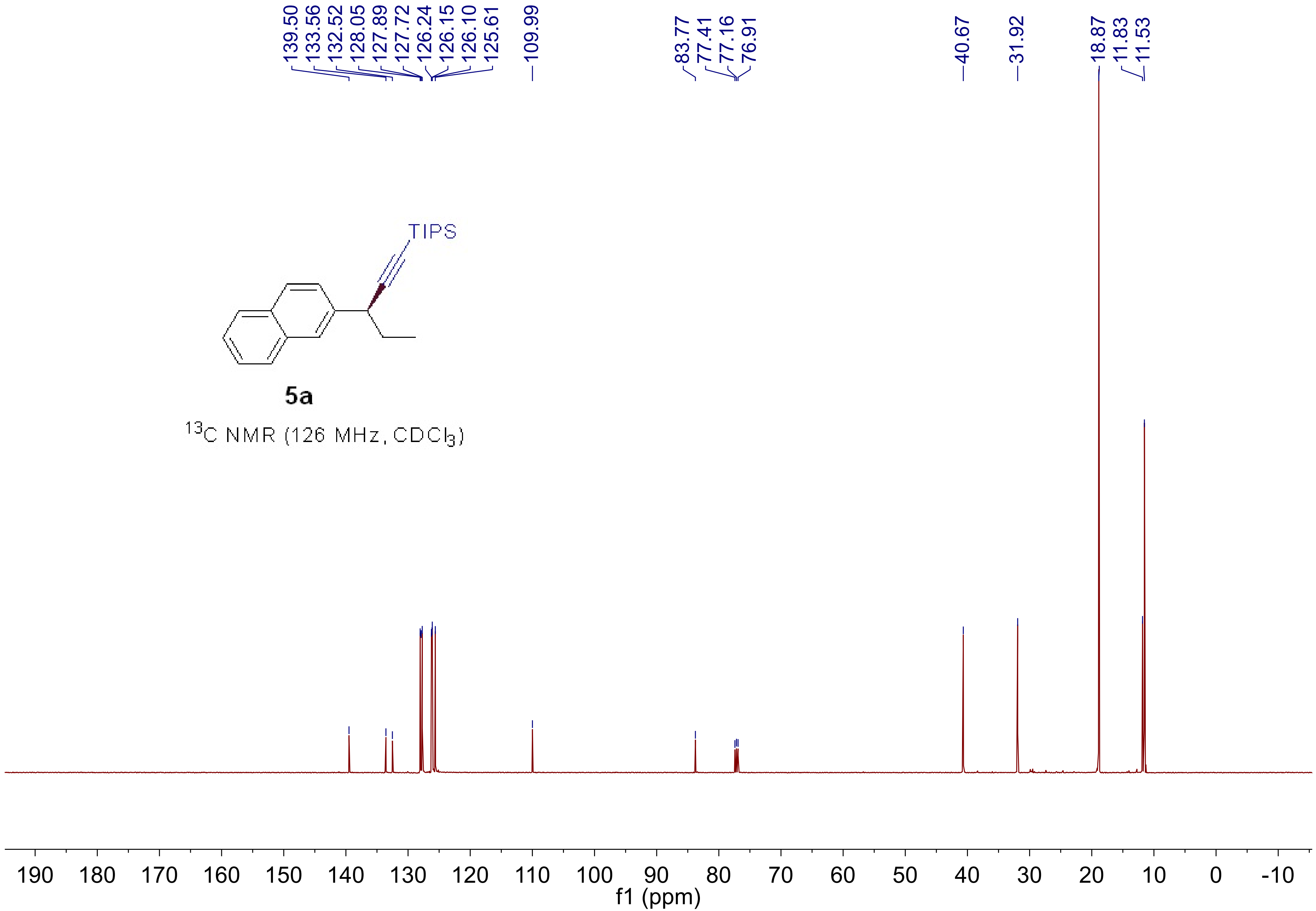

Supplementary Fig. 108. ^{13}C NMR (126 MHz, $CDCl_3$) spectra for compound **5a**

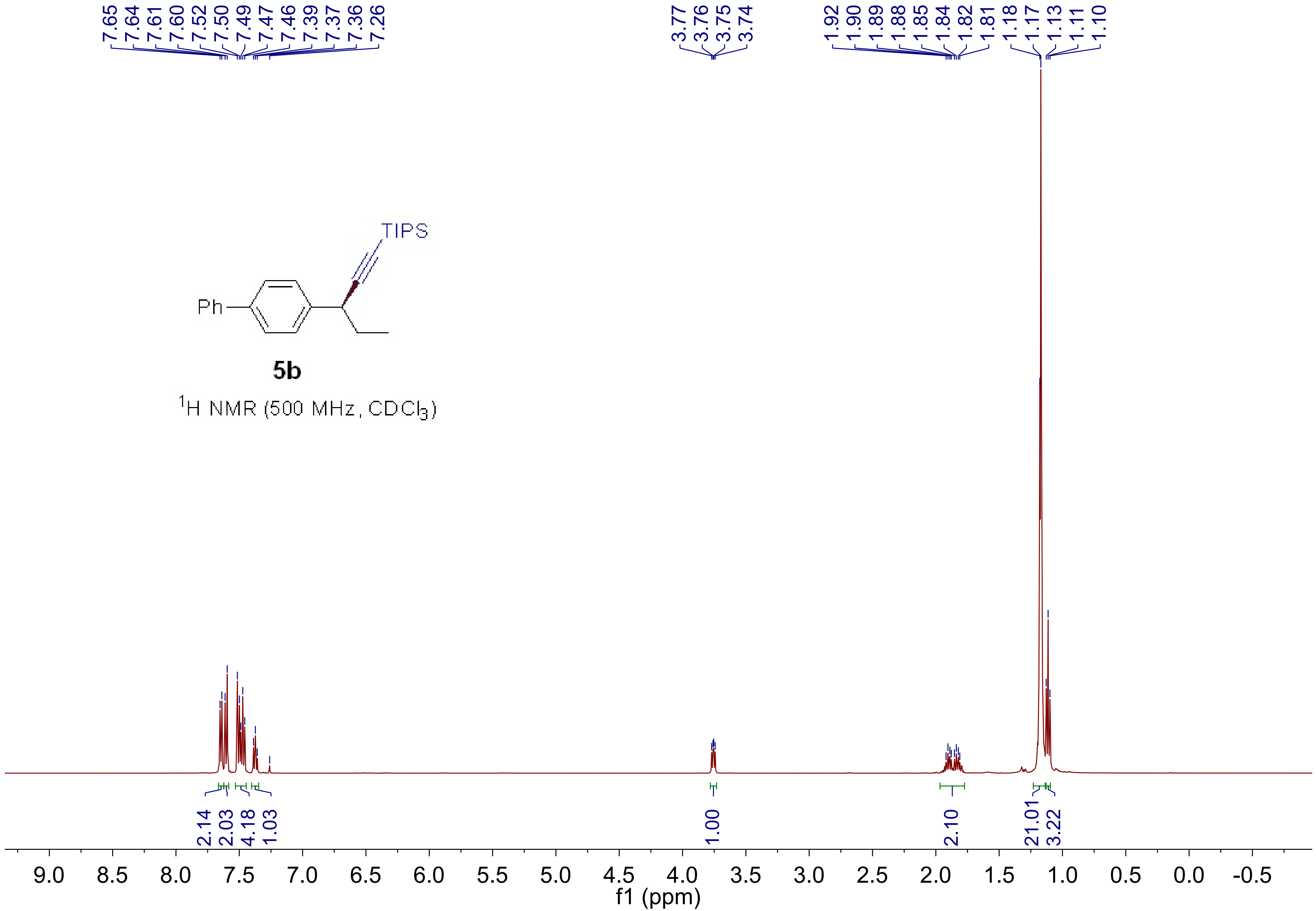

Supplementary Fig. 109. ^{1}H NMR (500 MHz, $CDCl_3$) spectra for compound **5b**

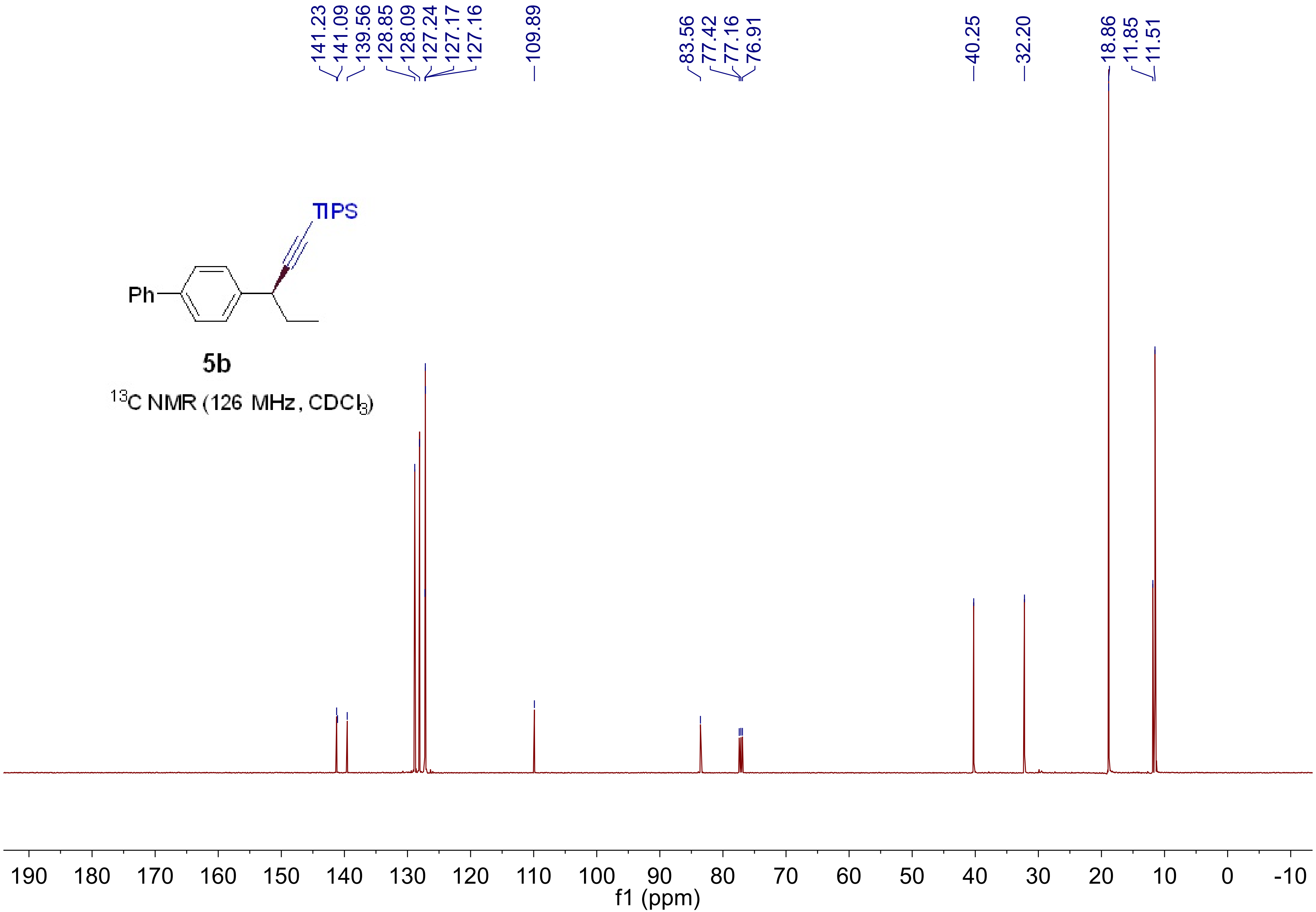

Supplementary Fig. 110. ^{13}C NMR (126 MHz, $CDCl_3$) spectra for compound **5b**

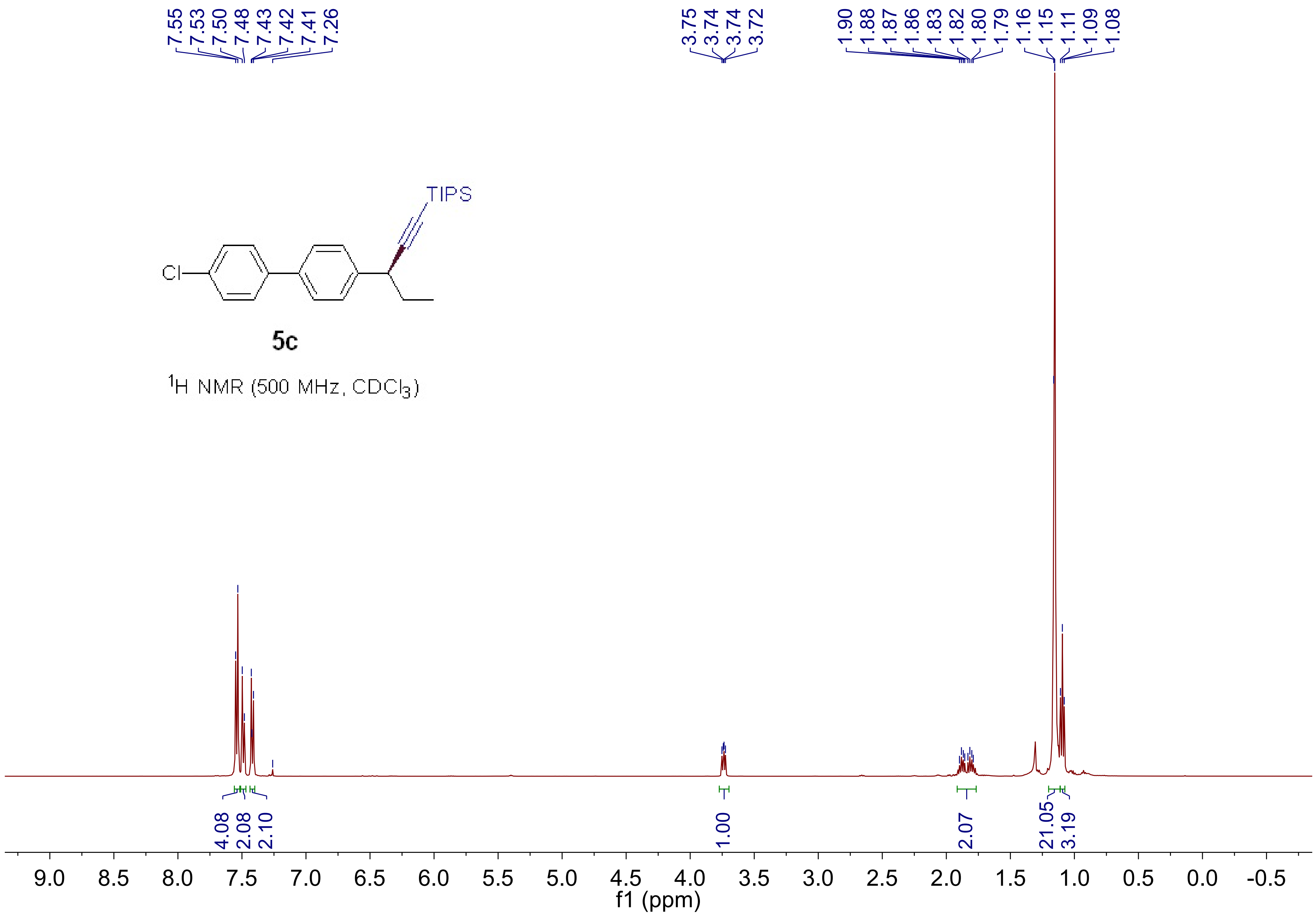

Supplementary Fig. 111. ^{1}H NMR (500 MHz, $CDCl_3$) spectra for compound **5c**

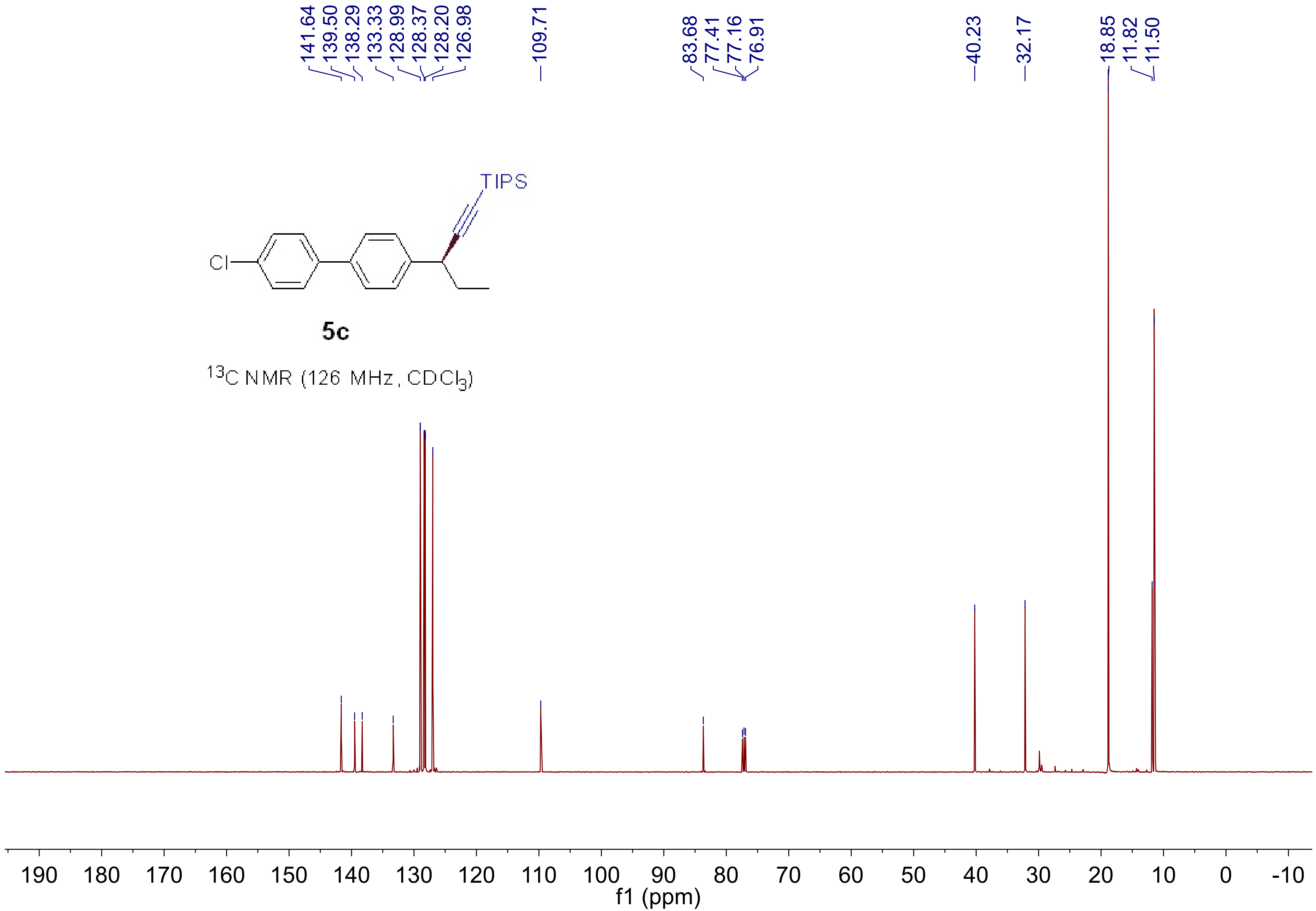

Supplementary Fig. 112. ^{13}C NMR (126 MHz, $CDCl_3$) spectra for compound **5c**

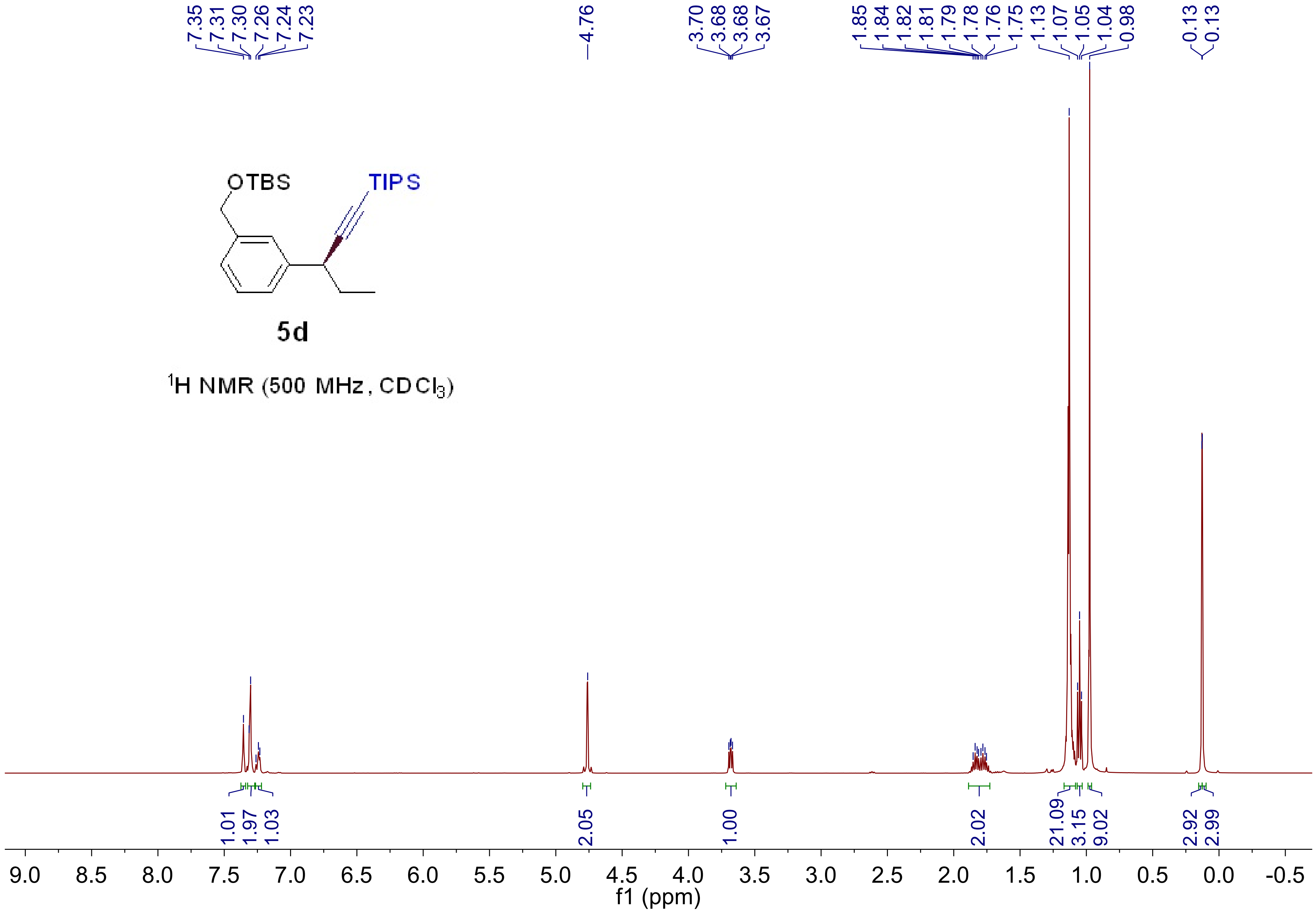

Supplementary Fig. 113. ^{1}H NMR (500 MHz, $CDCl_3$) spectra for compound **5d**

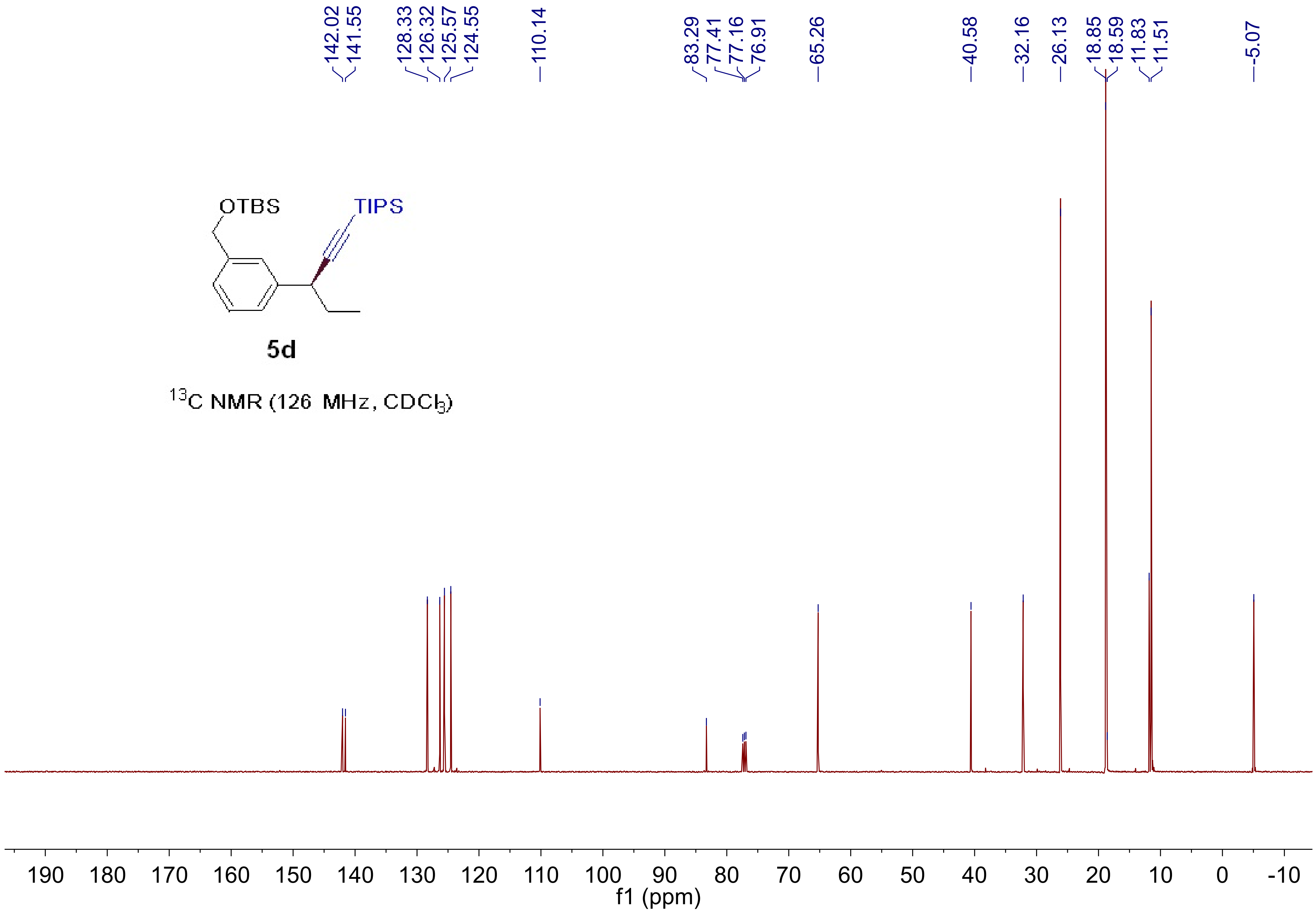

Supplementary Fig. 114. ^{13}C NMR (126 MHz, $CDCl_3$) spectra for compound **5d**

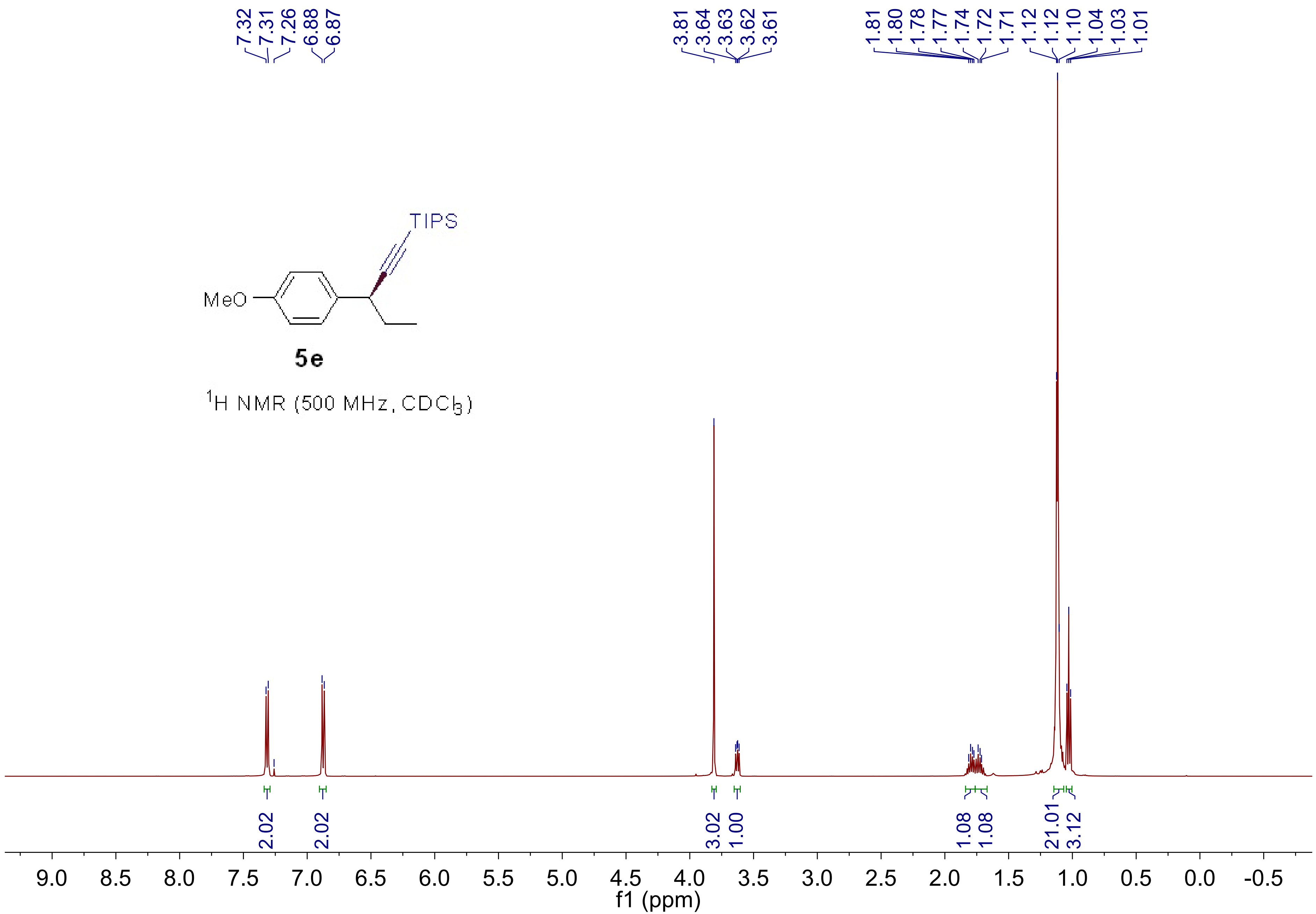

Supplementary Fig. 115. ^{1}H NMR (500 MHz, $CDCl_3$) spectra for compound **5e**

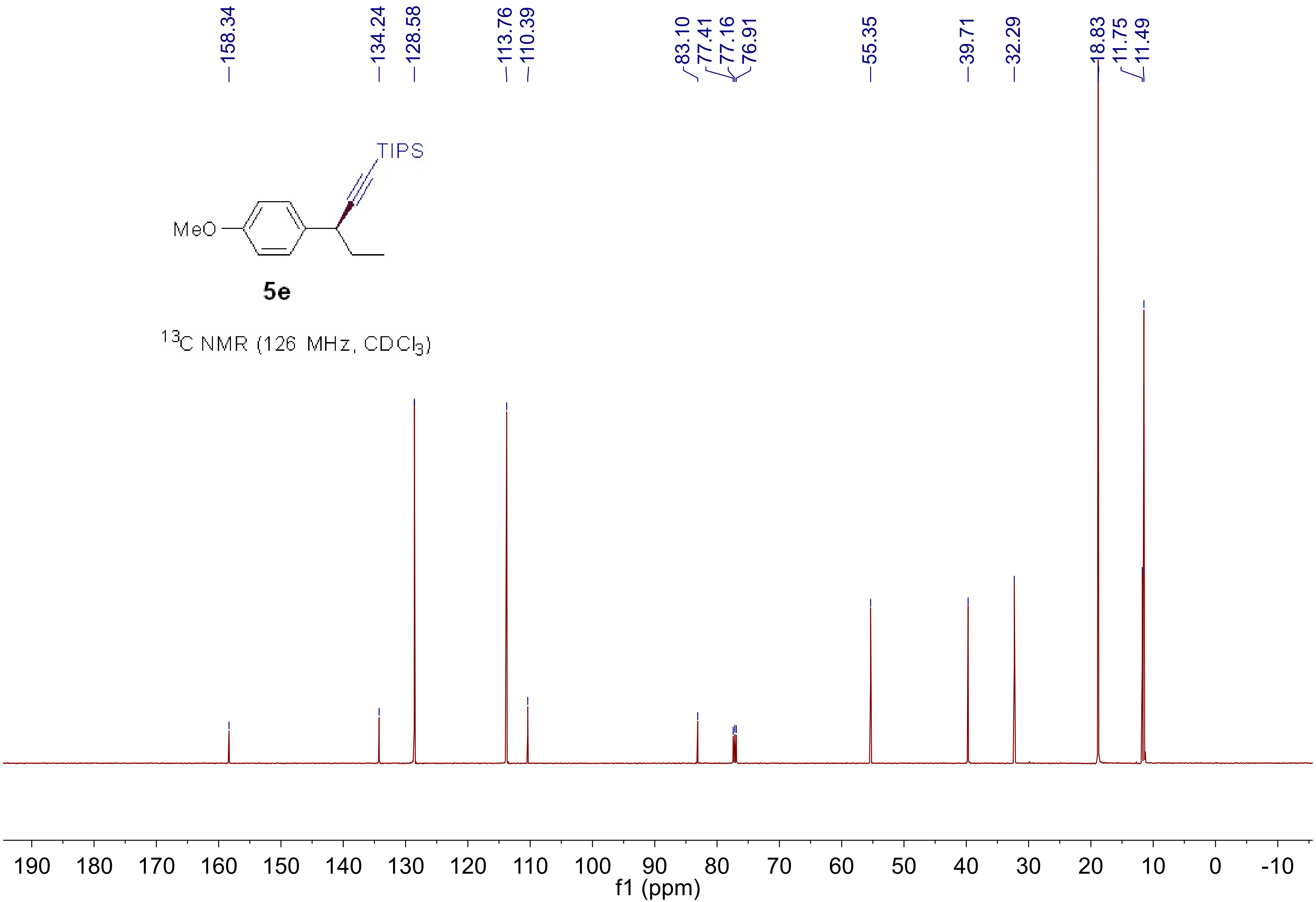

Supplementary Fig. 116. ^{13}C NMR (126 MHz, $CDCl_3$) spectra for compound **5e**

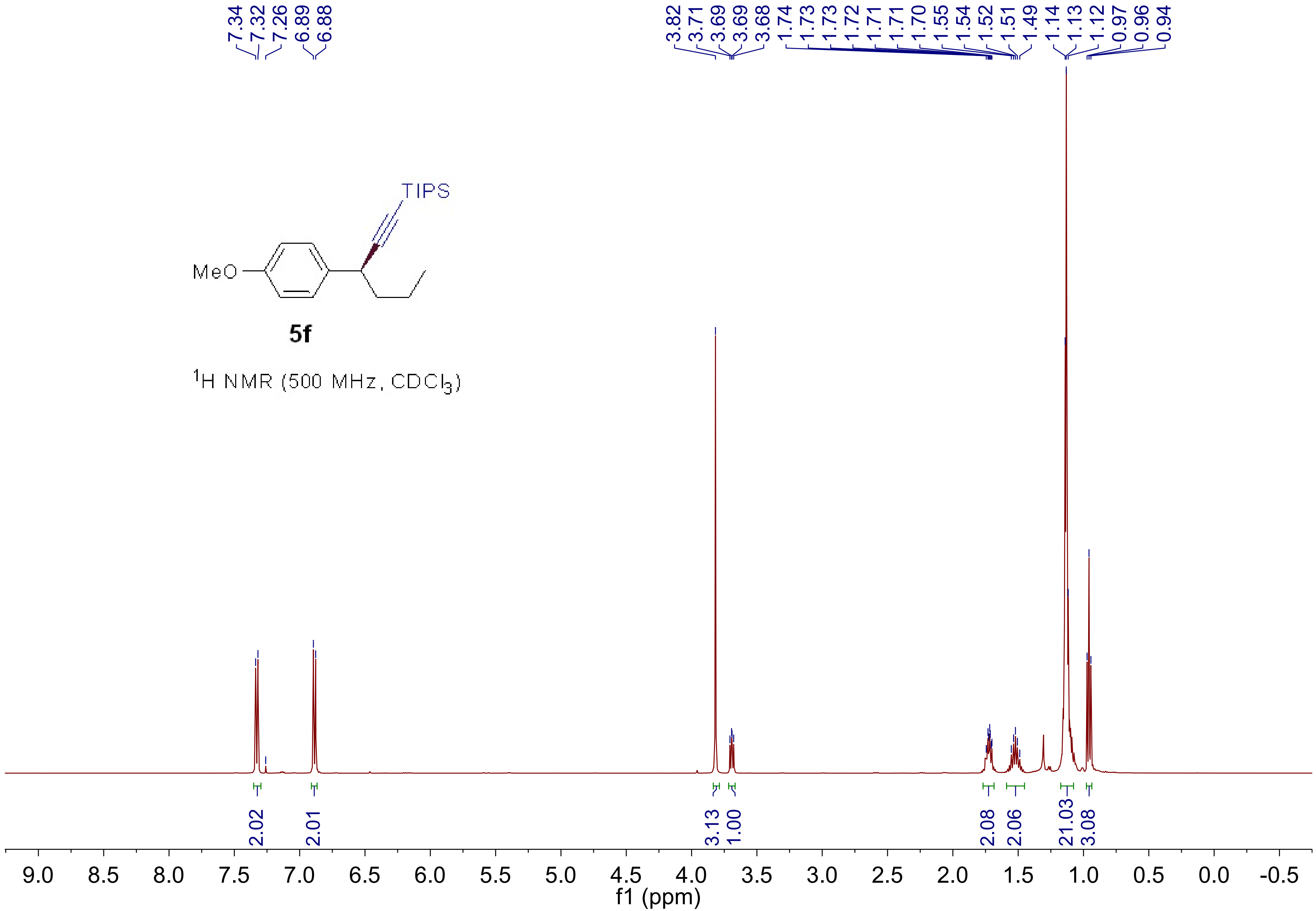

Supplementary Fig. 117. ^{1}H NMR (500 MHz, $CDCl_3$) spectra for compound **5f**

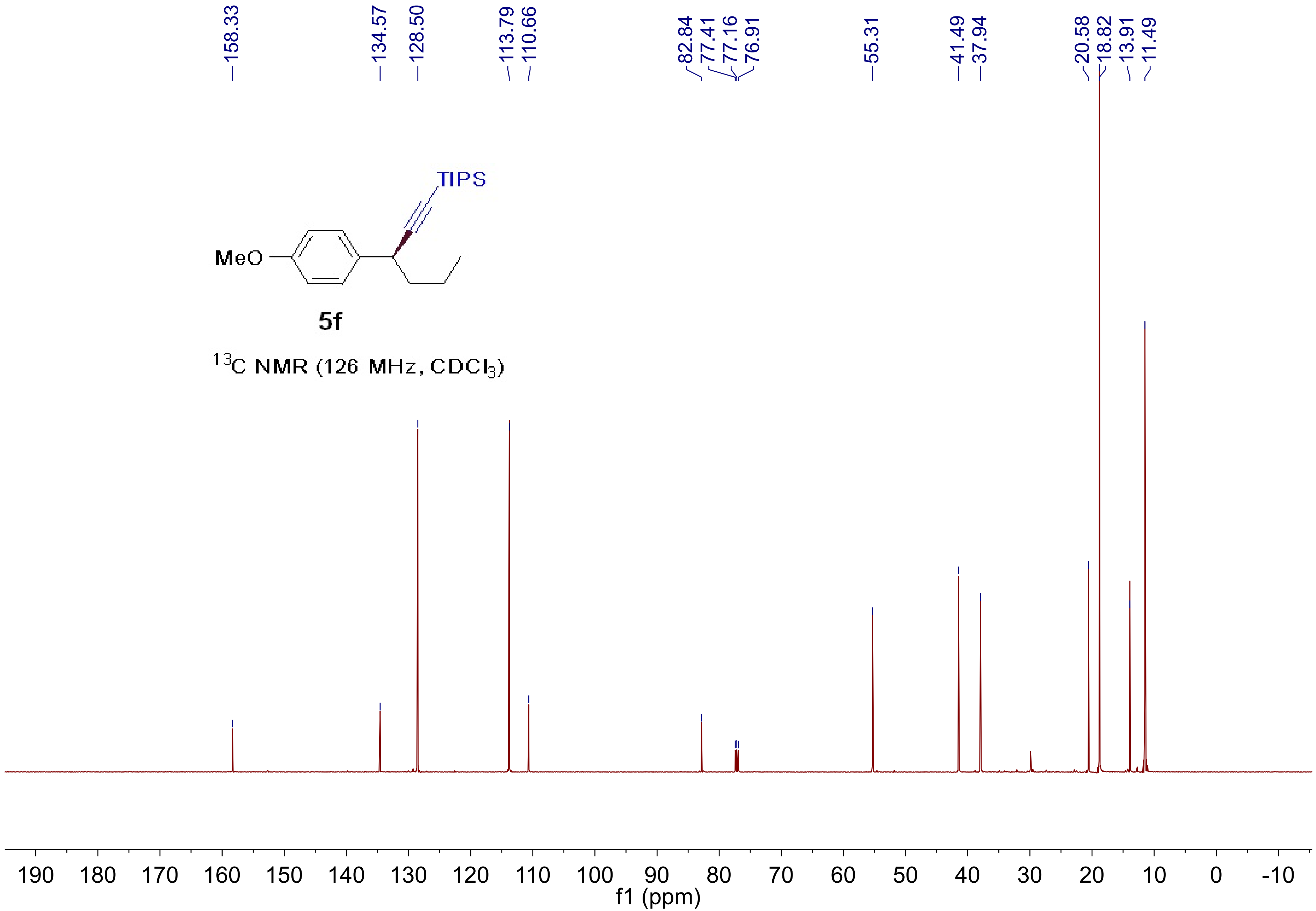

Supplementary Fig. 118. ^{13}C NMR (126 MHz, $CDCl_3$) spectra for compound **5f**

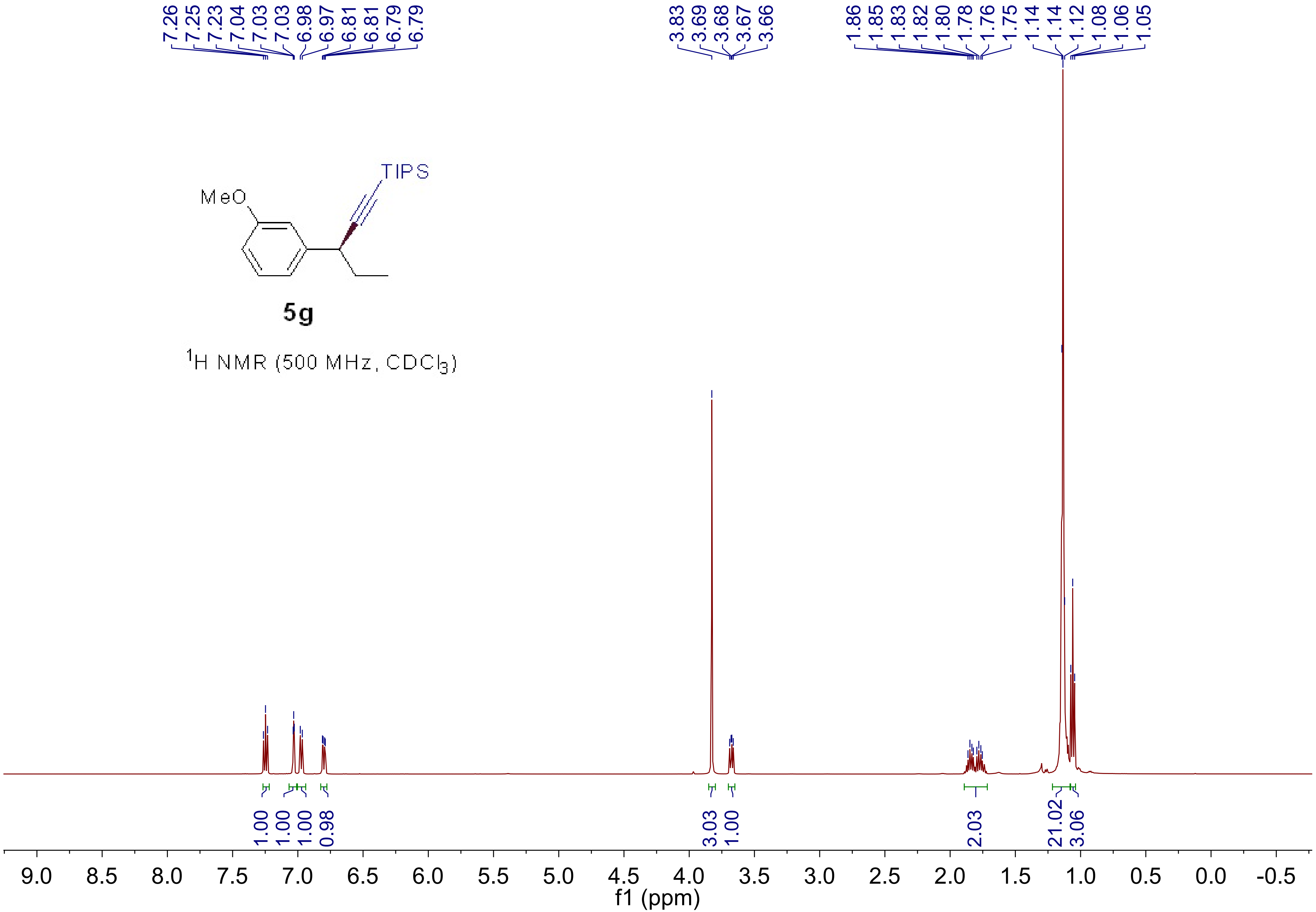

Supplementary Fig. 119. ^{1}H NMR (500 MHz, $CDCl_3$) spectra for compound **5g**

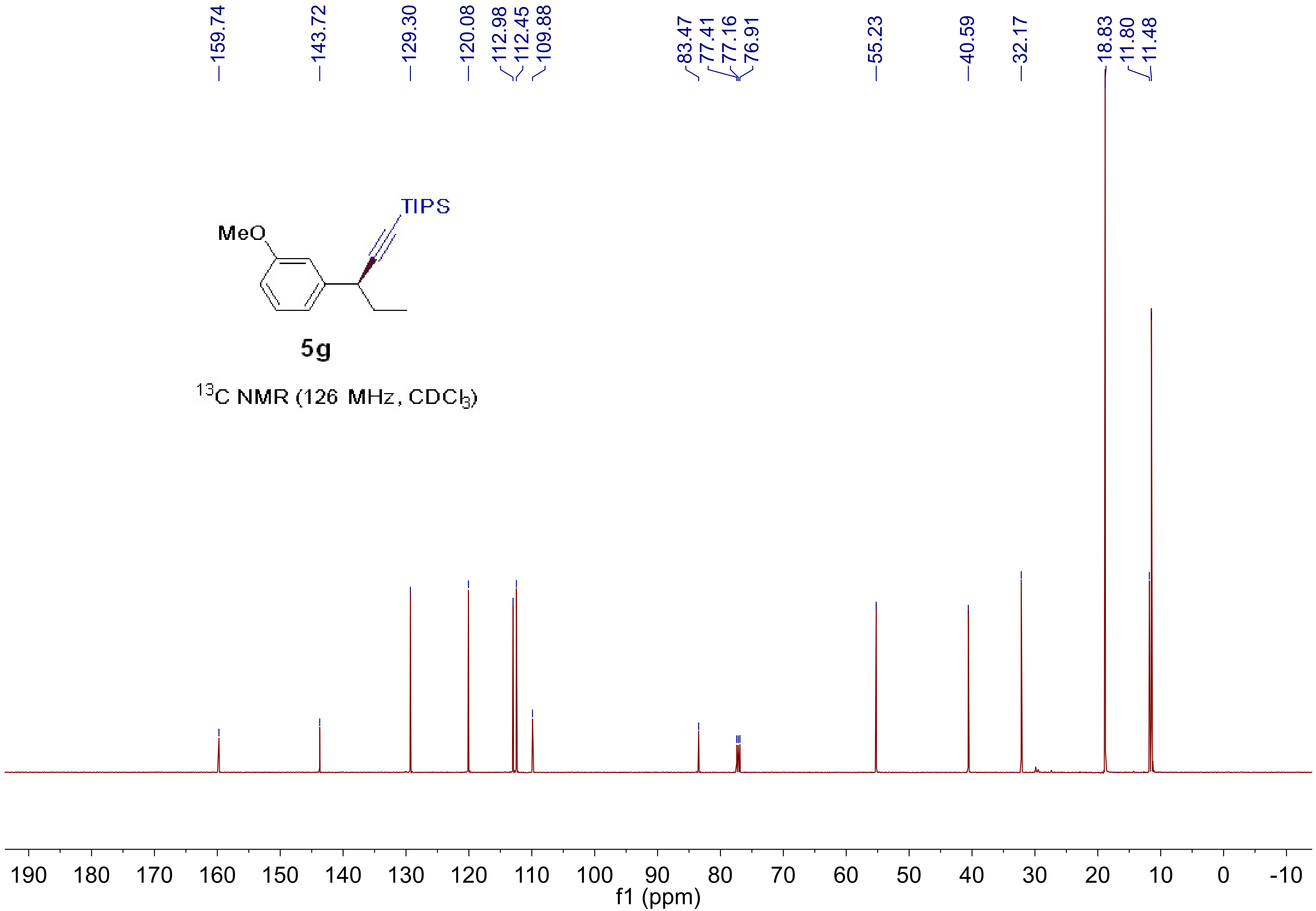

Supplementary Fig. 120. ^{13}C NMR (126 MHz, $CDCl_3$) spectra for compound **5g**

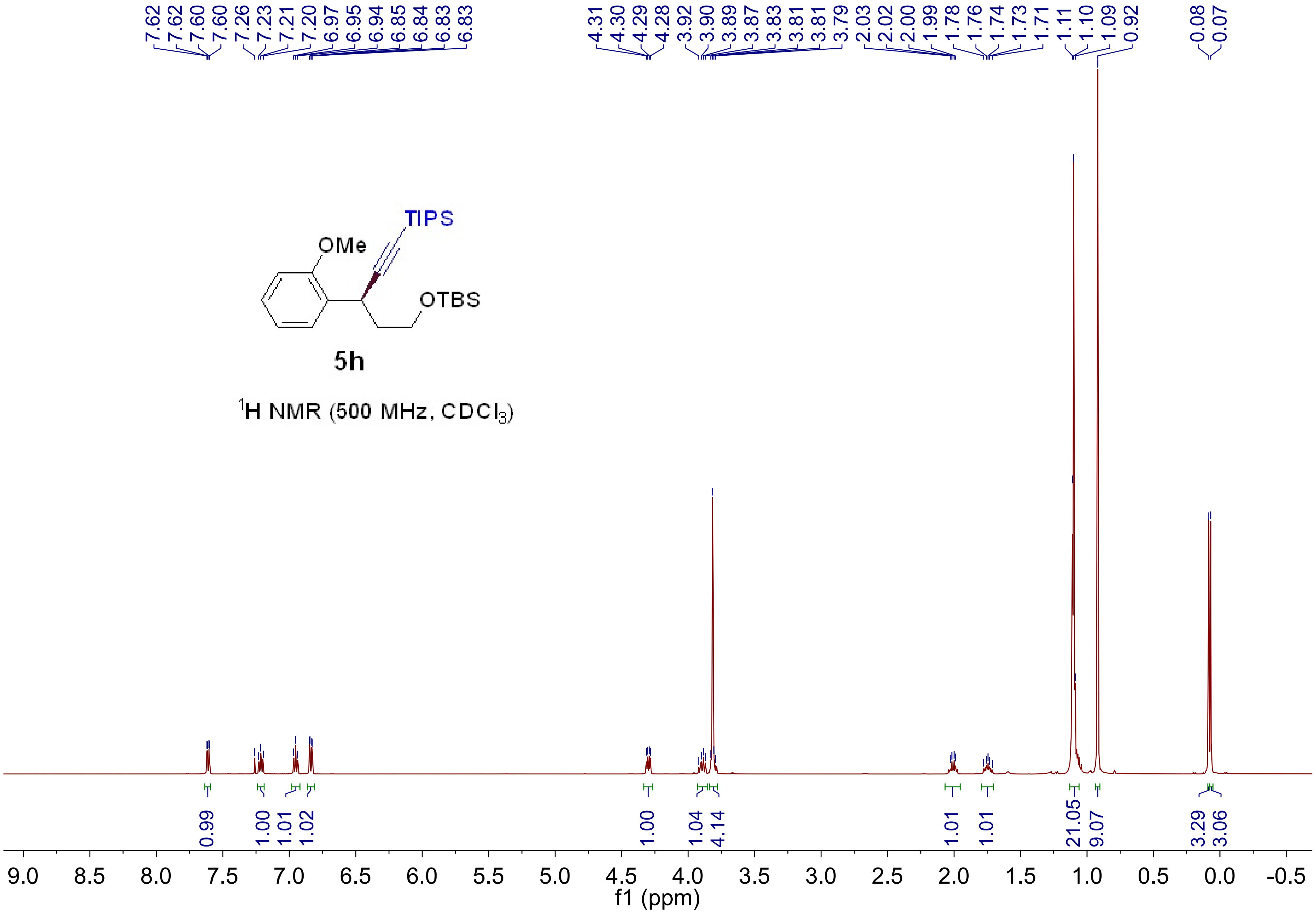

Supplementary Fig. 121. ^{1}H NMR (500 MHz, $CDCl_3$) spectra for compound **5h**

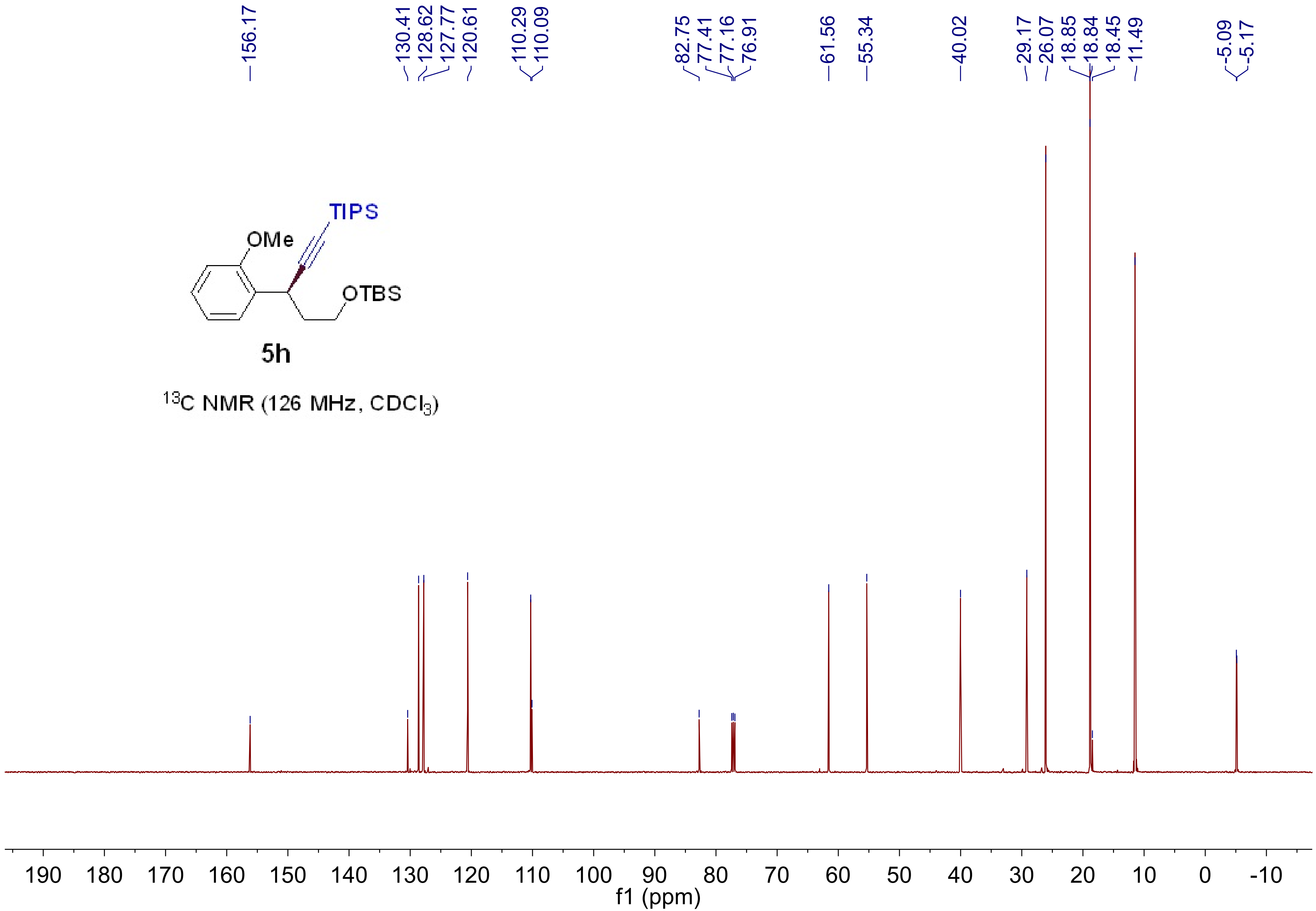

Supplementary Fig. 122. ^{13}C NMR (126 MHz, $CDCl_3$) spectra for compound **5h**

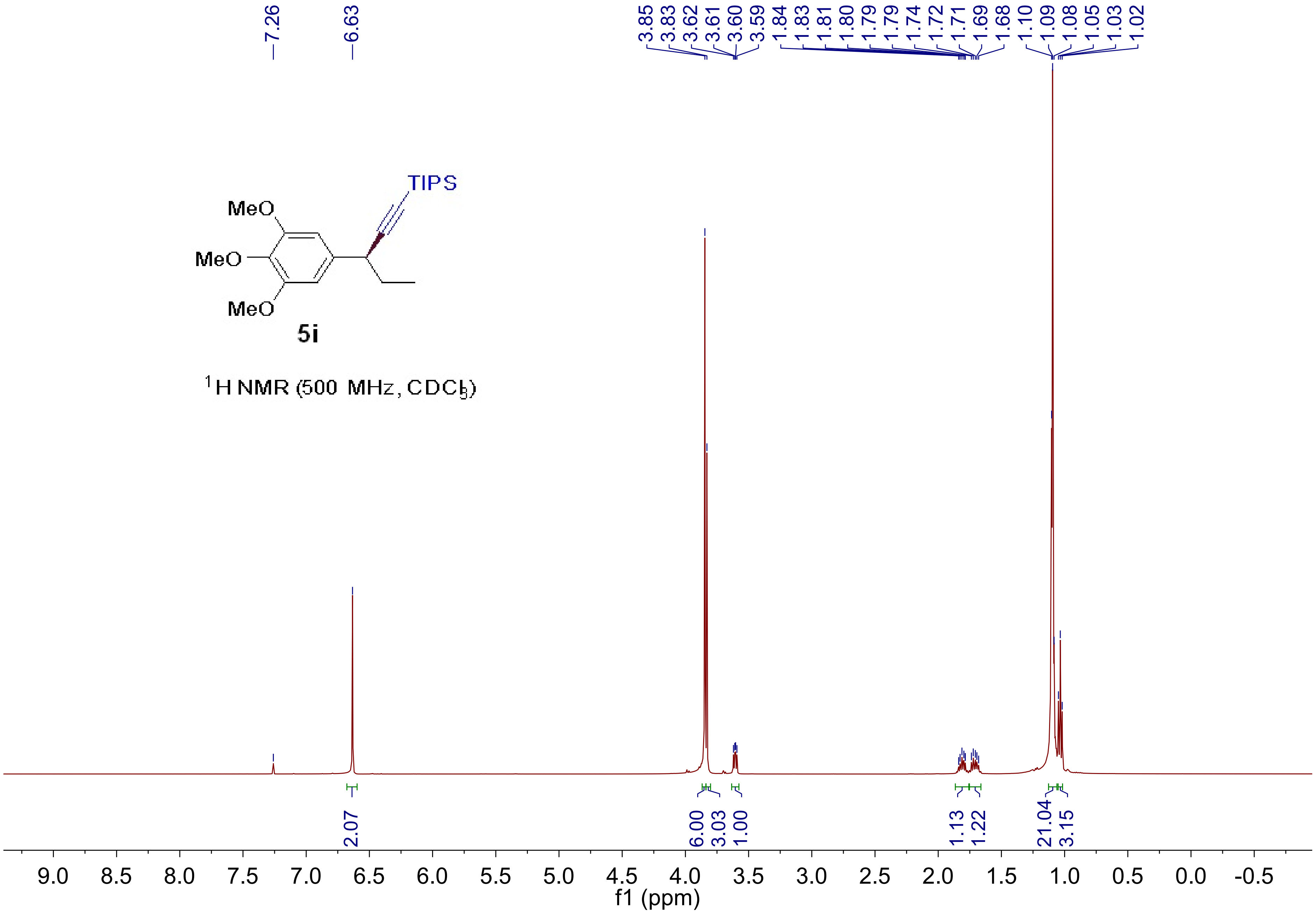

Supplementary Fig. 123. ^{1}H NMR (500 MHz, $CDCl_3$) spectra for compound **5i**

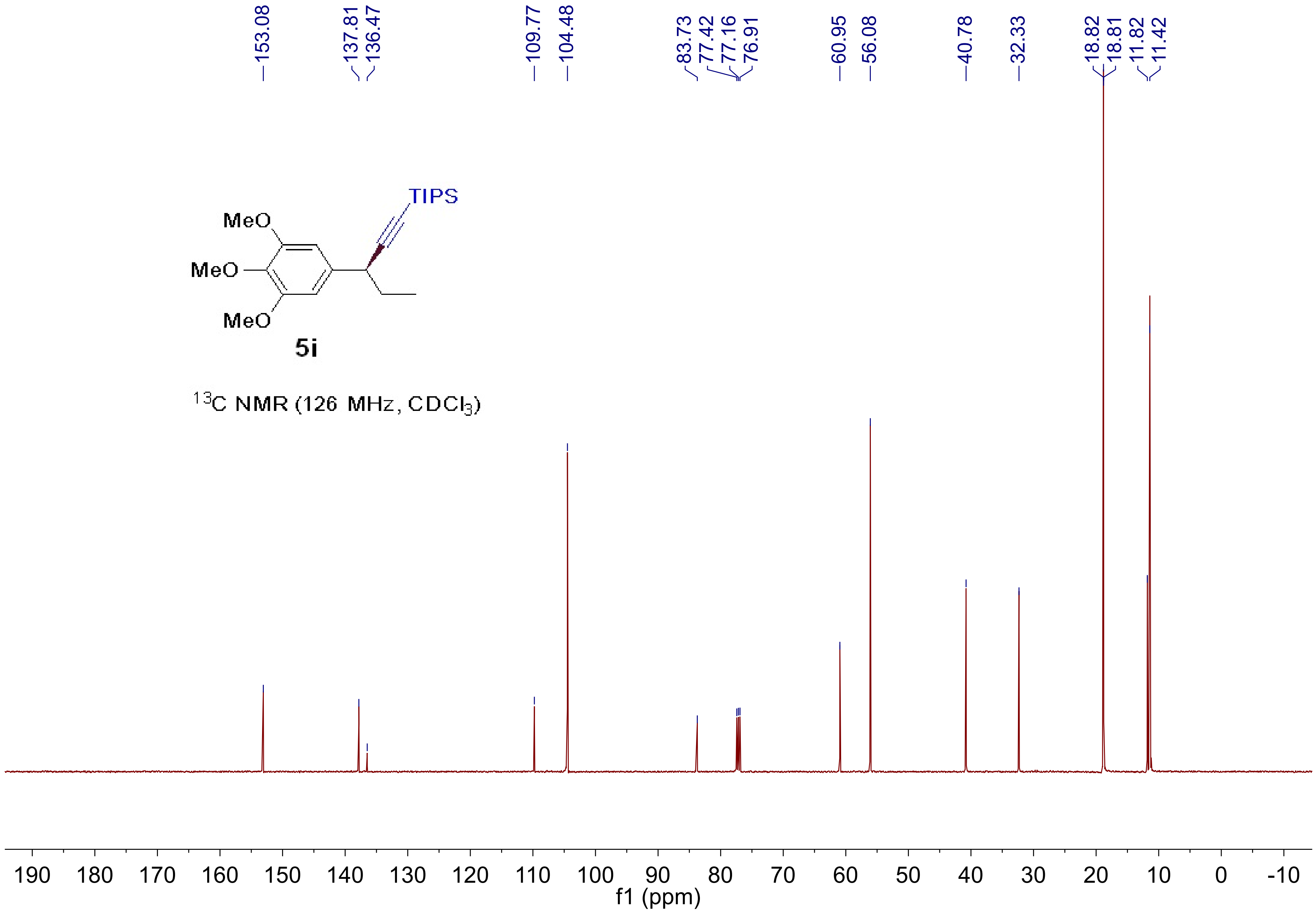

Supplementary Fig. 124. ^{13}C NMR (126 MHz, $CDCl_3$) spectra for compound **5i**

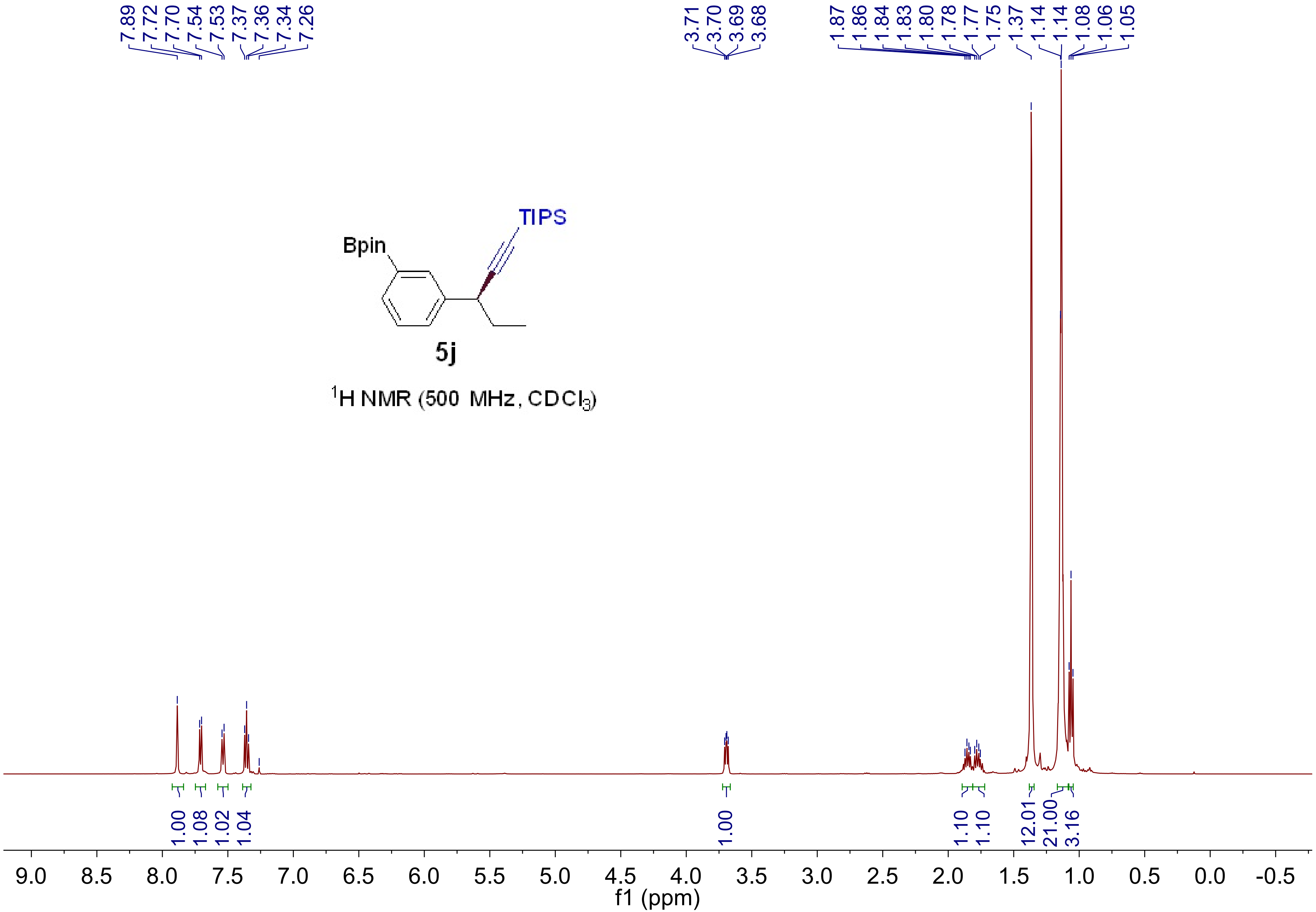

Supplementary Fig. 125. ^{1}H NMR (500 MHz, $CDCl_3$) spectra for compound **5j**

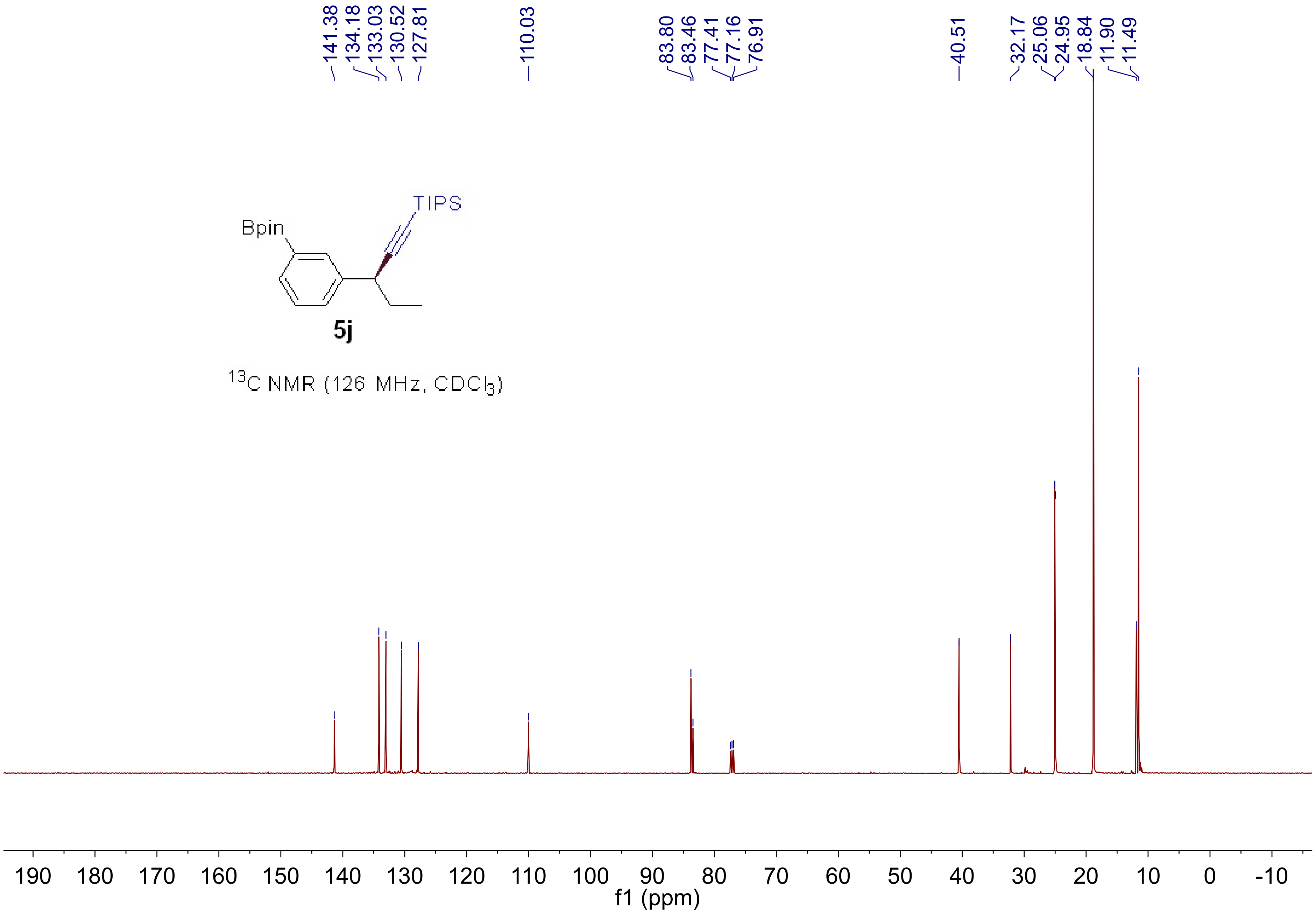

Supplementary Fig. 126. ^{13}C NMR (126 MHz, CDCl$_3$) spectra for compound **5j**

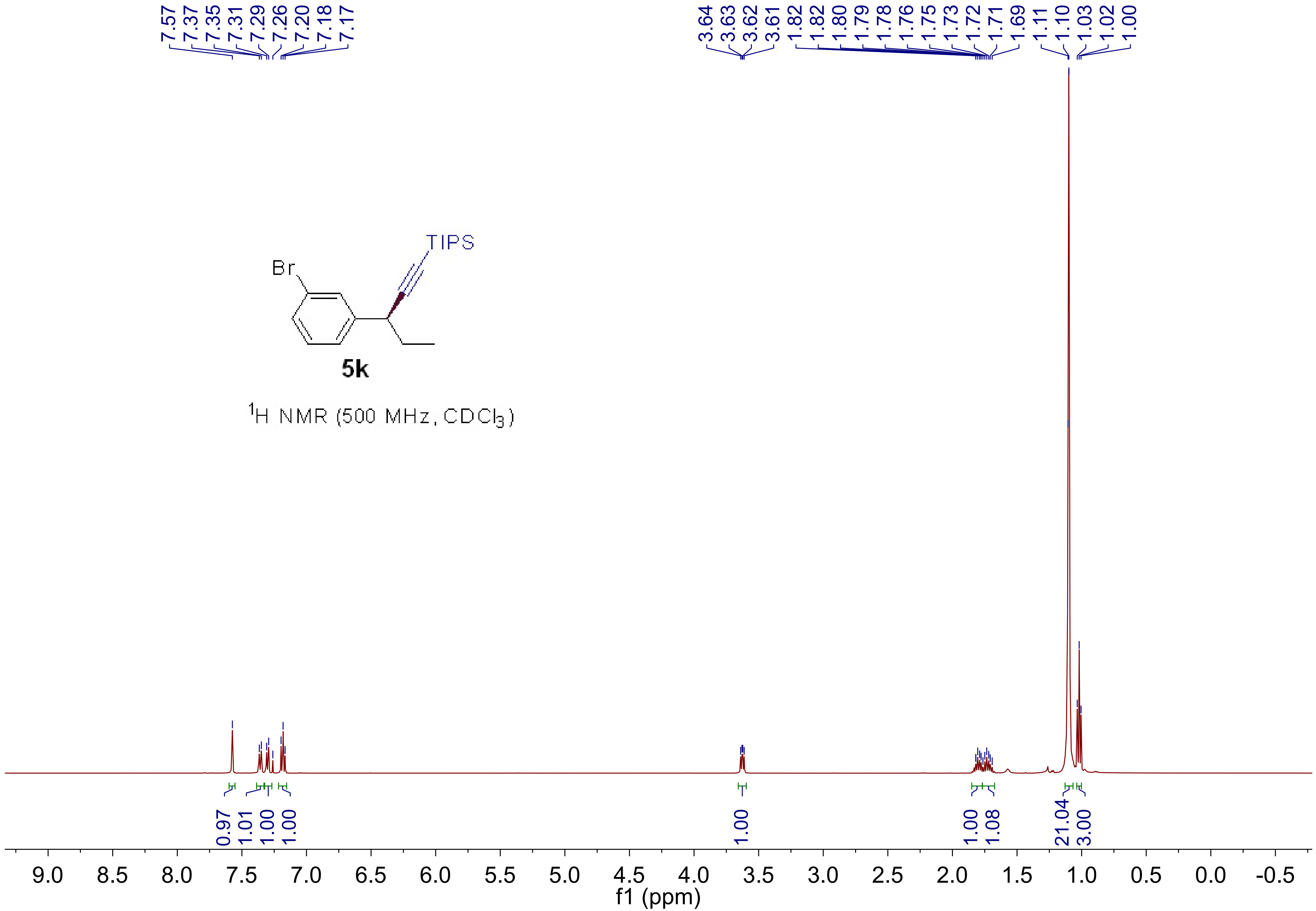

Supplementary Fig. 127. ^{1}H NMR (500 MHz, $CDCl_3$) spectra for compound **5k**

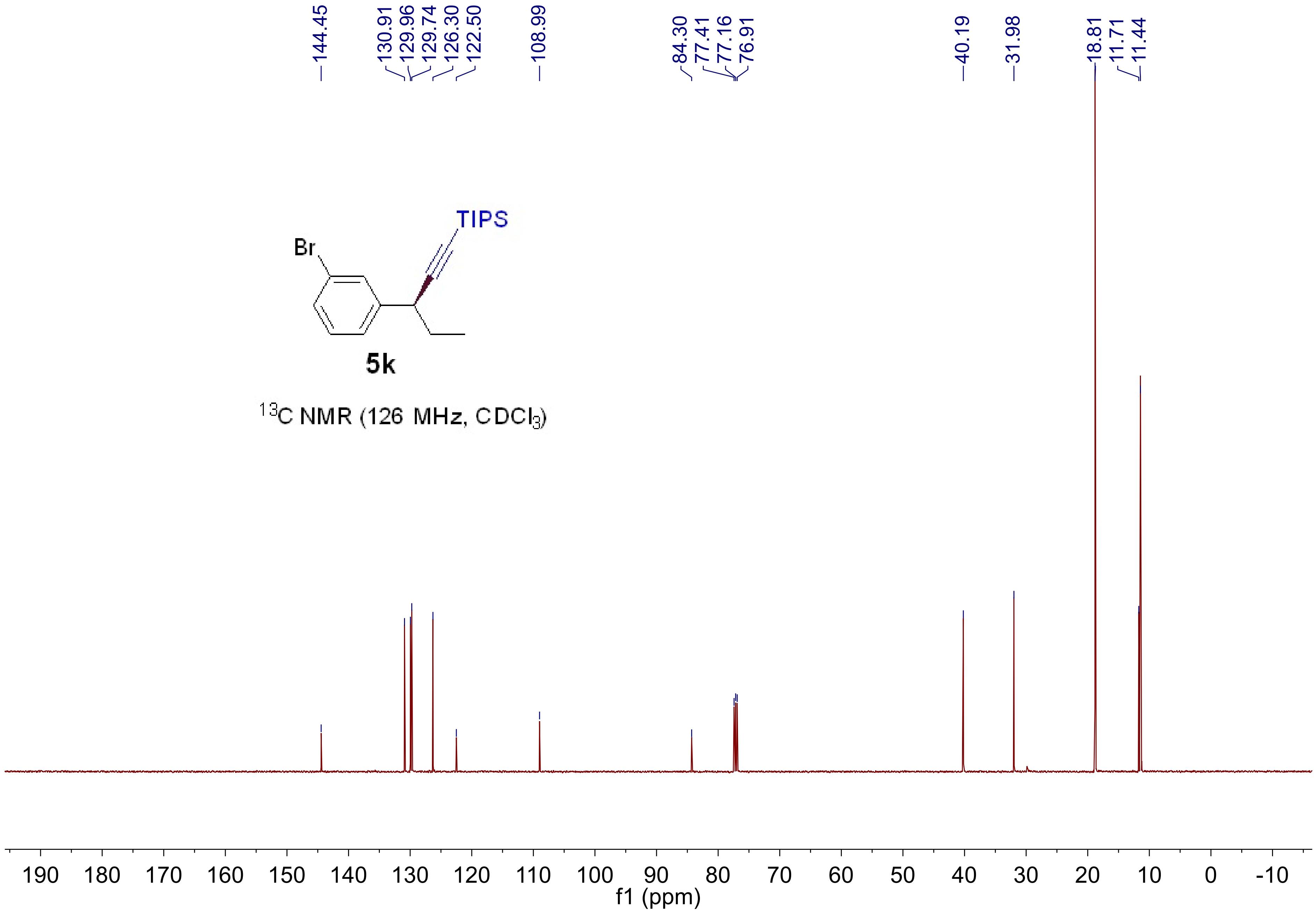

Supplementary Fig. 128. ^{13}C NMR (126 MHz, $CDCl_3$) spectra for compound **5k**

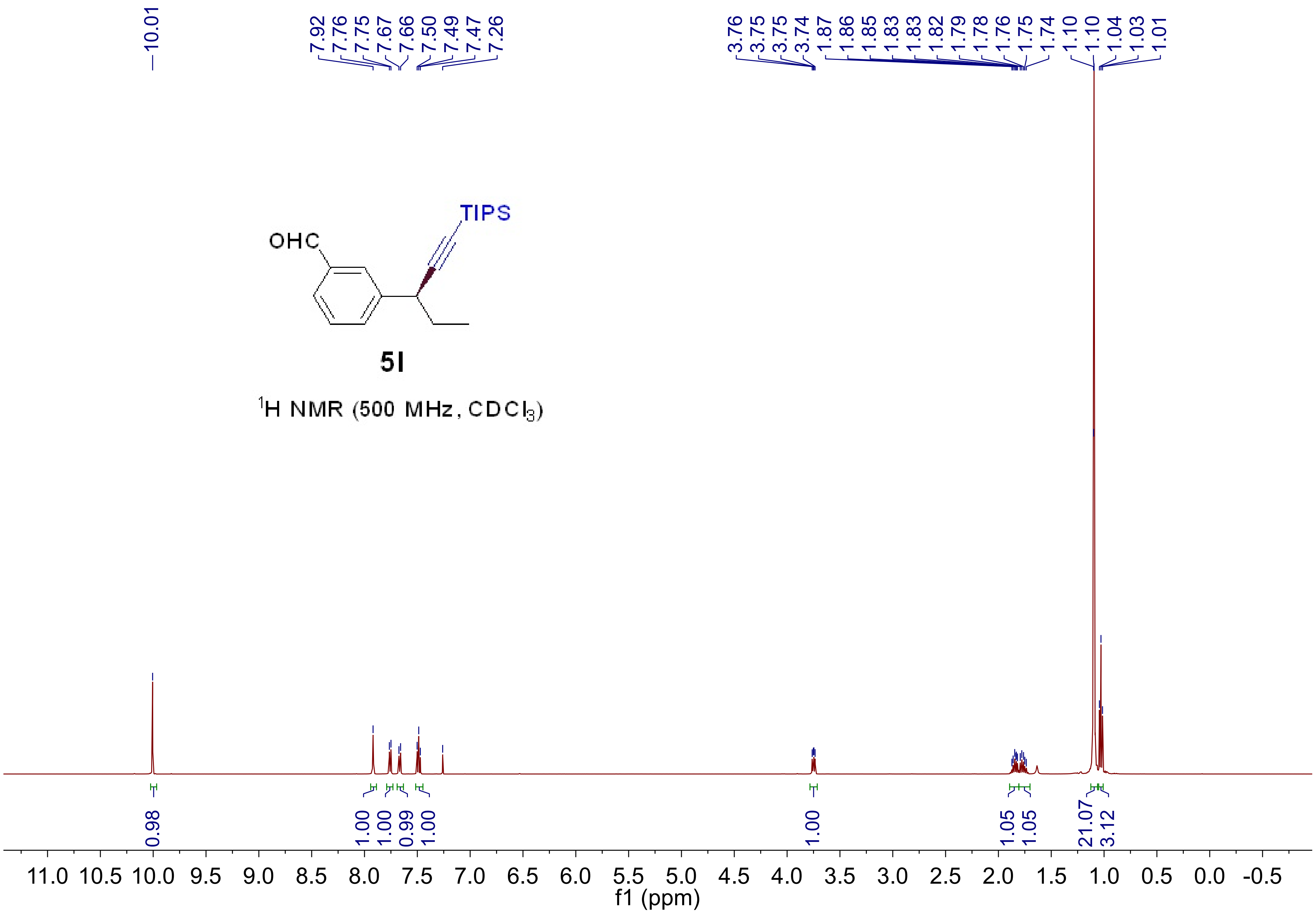

Supplementary Fig. 129. ^{1}H NMR (500 MHz, $CDCl_3$) spectra for compound **5l**

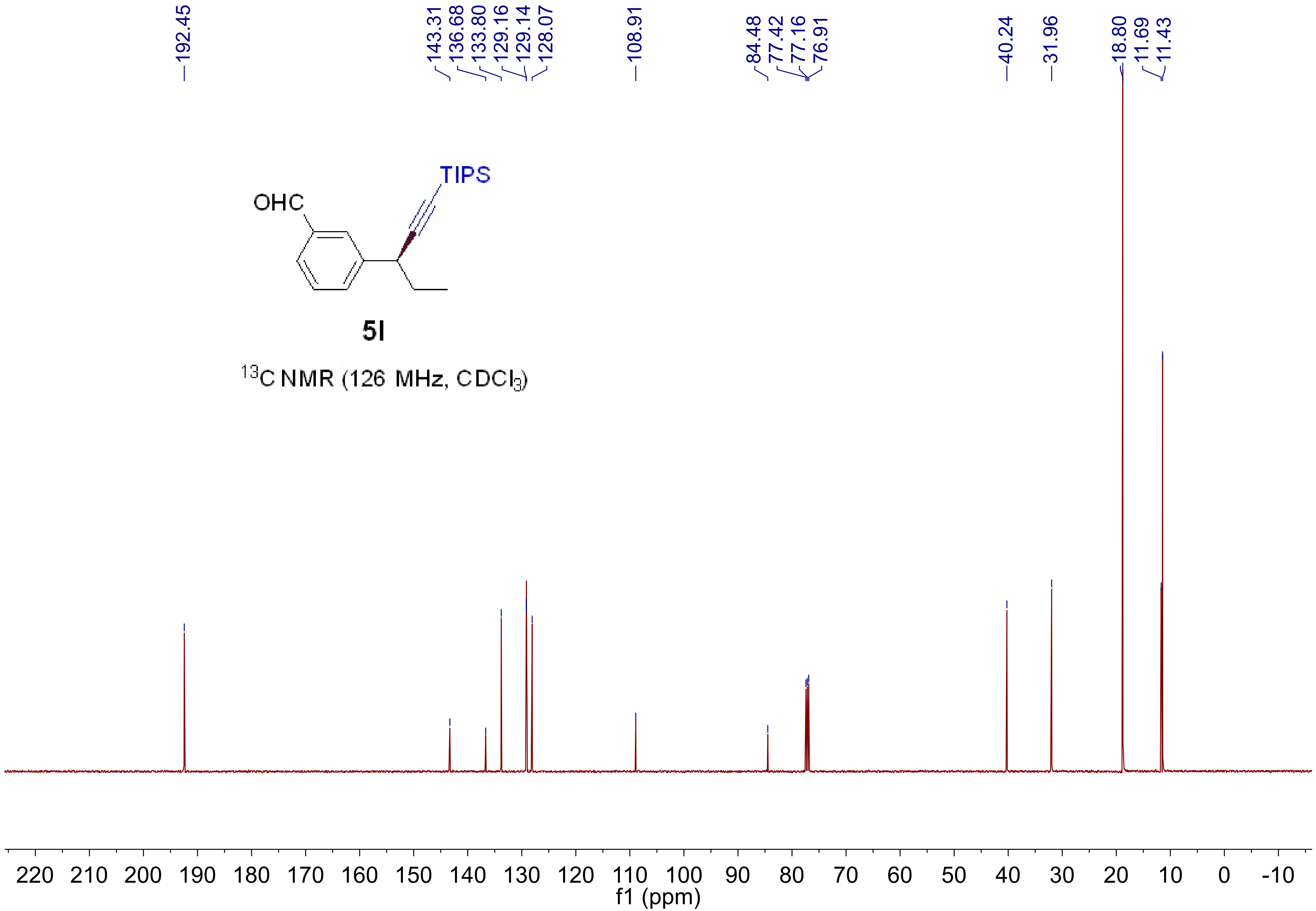

Supplementary Fig. 130. ^{13}C NMR (126 MHz, $CDCl_3$) spectra for compound **5l**

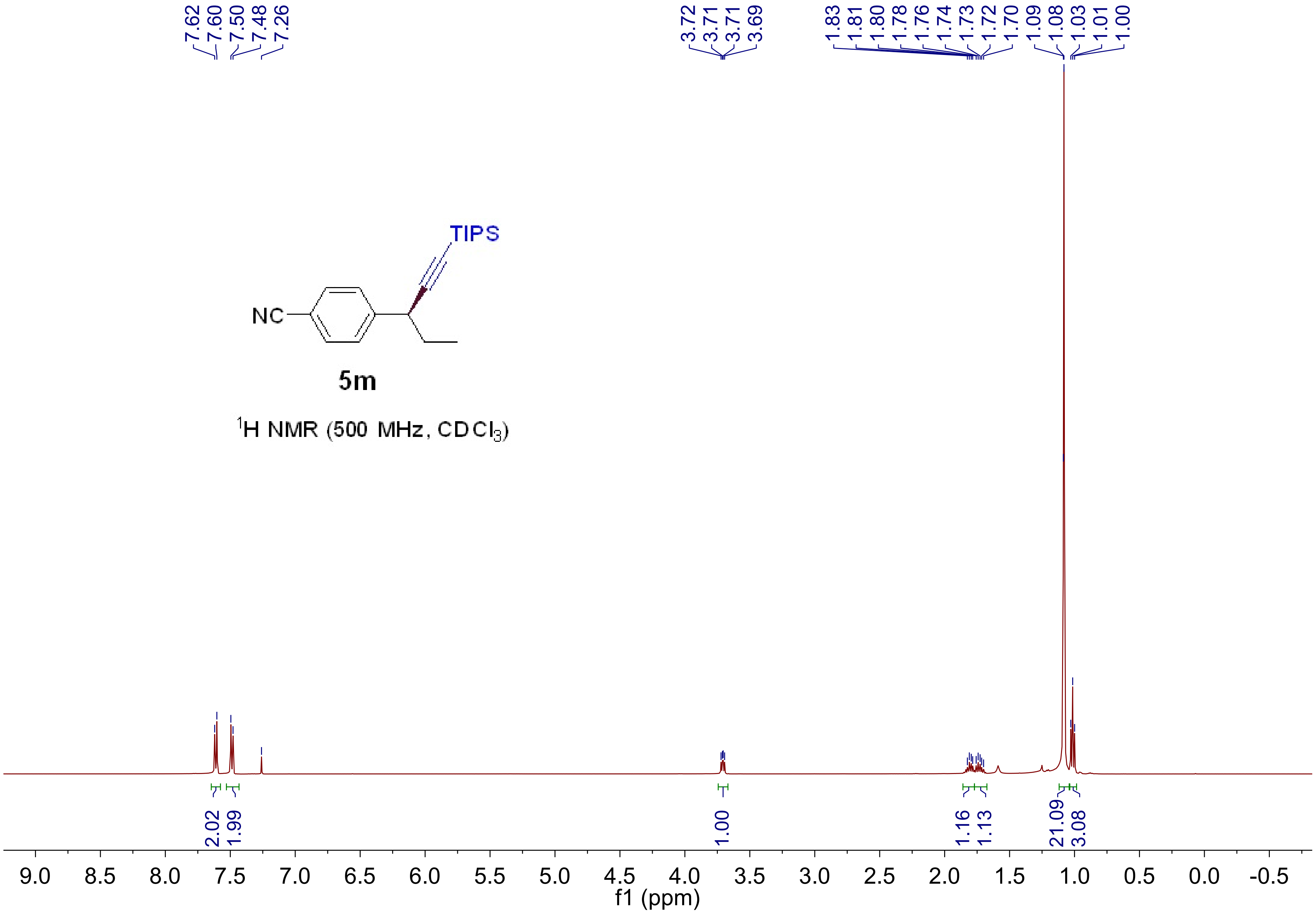

Supplementary Fig. 131. ^{1}H NMR (500 MHz, $CDCl_3$) spectra for compound **5m**

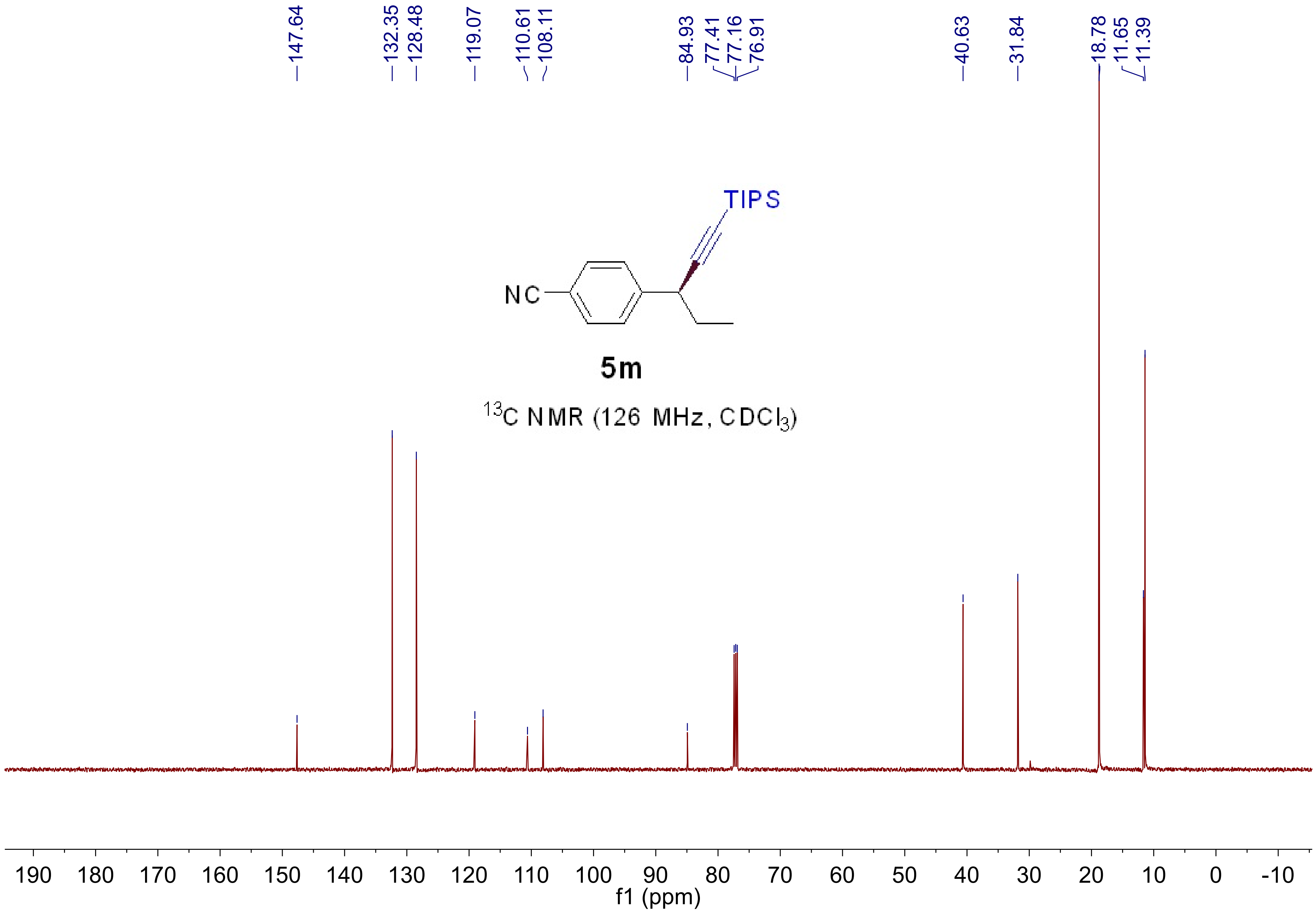

Supplementary Fig. 132. ^{13}C NMR (126 MHz, $CDCl_3$) spectra for compound **5m**

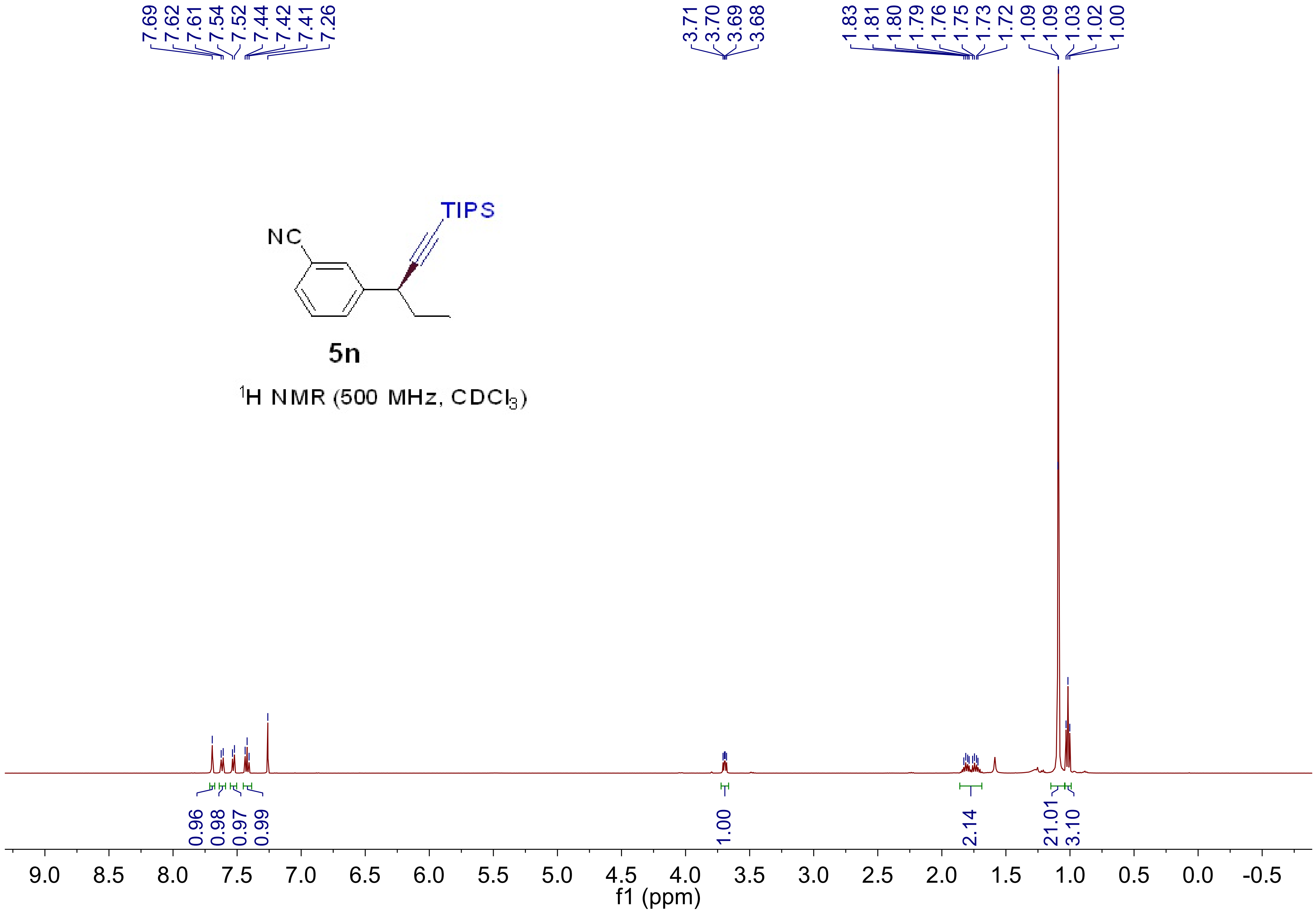

Supplementary Fig. 133. ^{1}H NMR (500 MHz, $CDCl_3$) spectra for compound **5n**

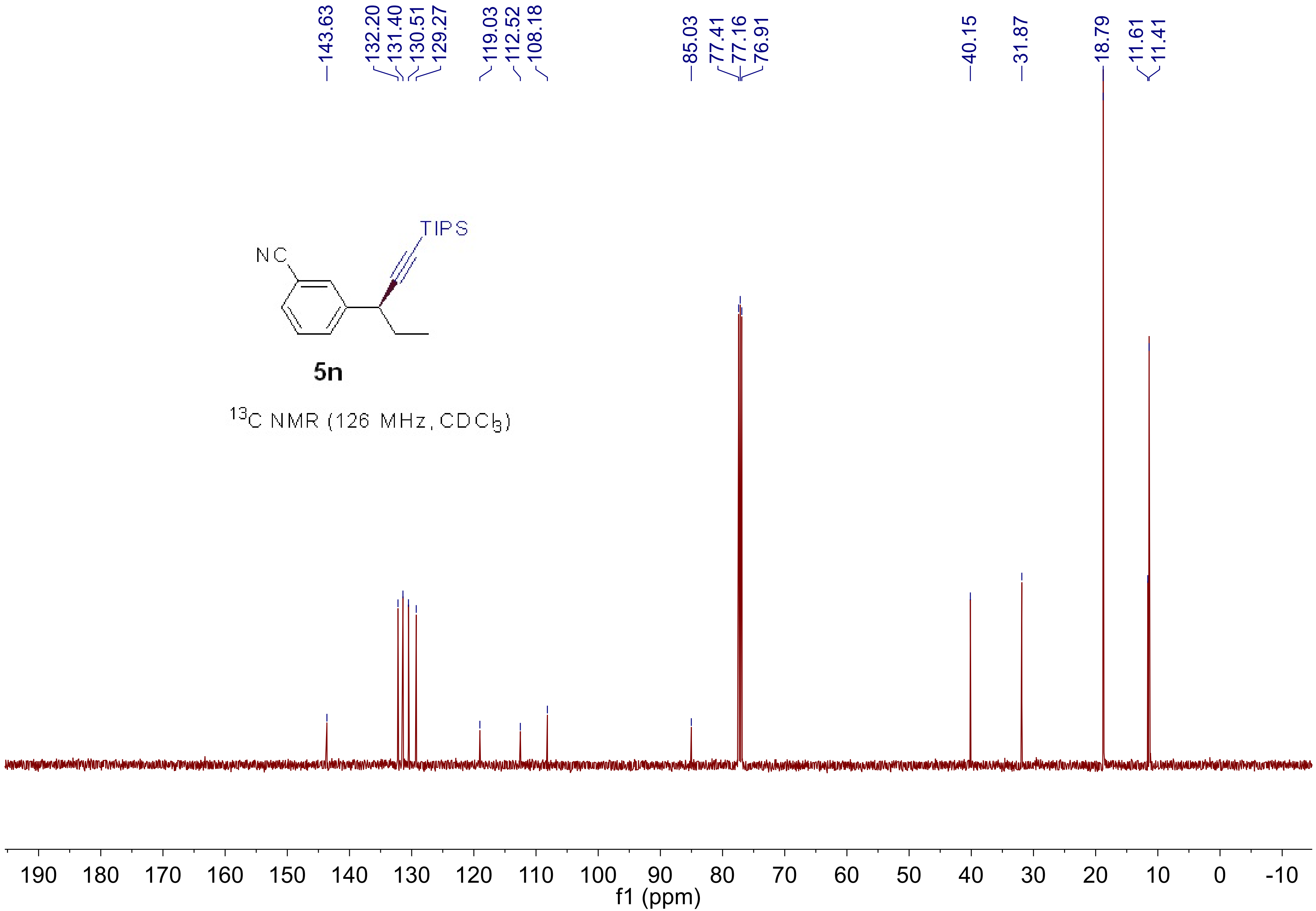

Supplementary Fig. 134. ^{13}C NMR (126 MHz, $CDCl_3$) spectra for compound **5n**

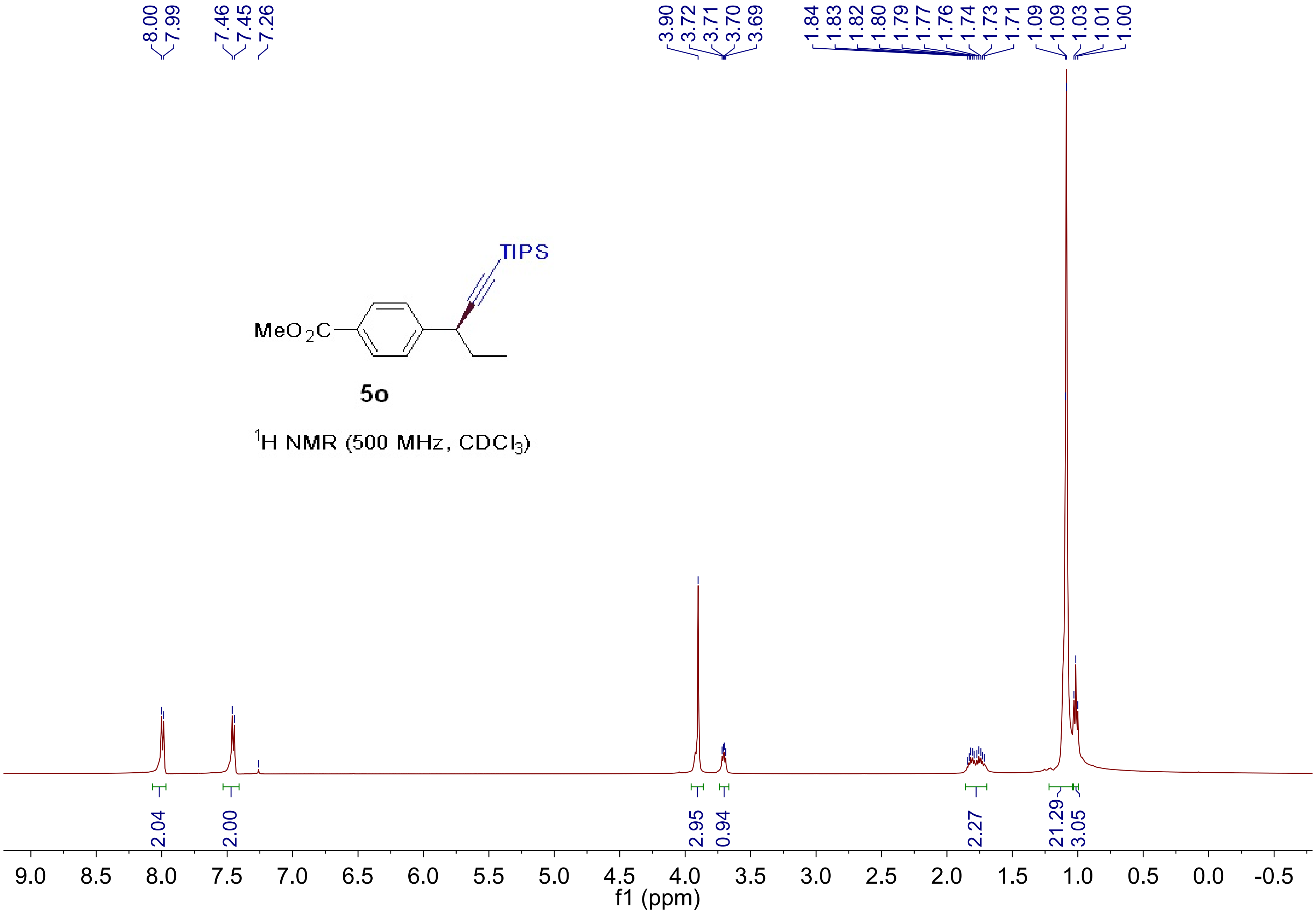

Supplementary Fig. 135. ^{1}H NMR (500 MHz, $CDCl_3$) spectra for compound **5o**

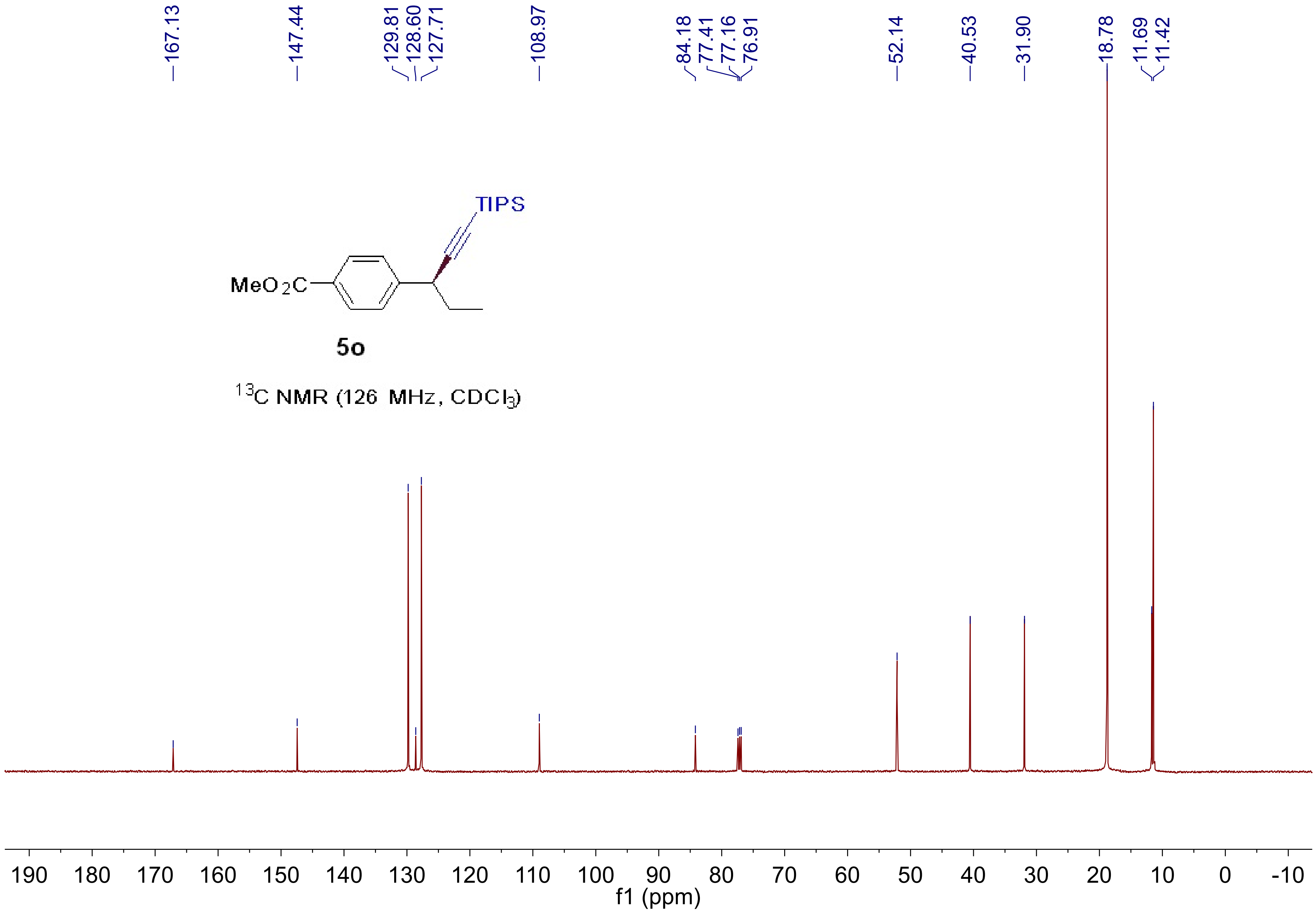

Supplementary Fig. 136. ^{13}C NMR (126 MHz, $CDCl_3$) spectra for compound **5o**

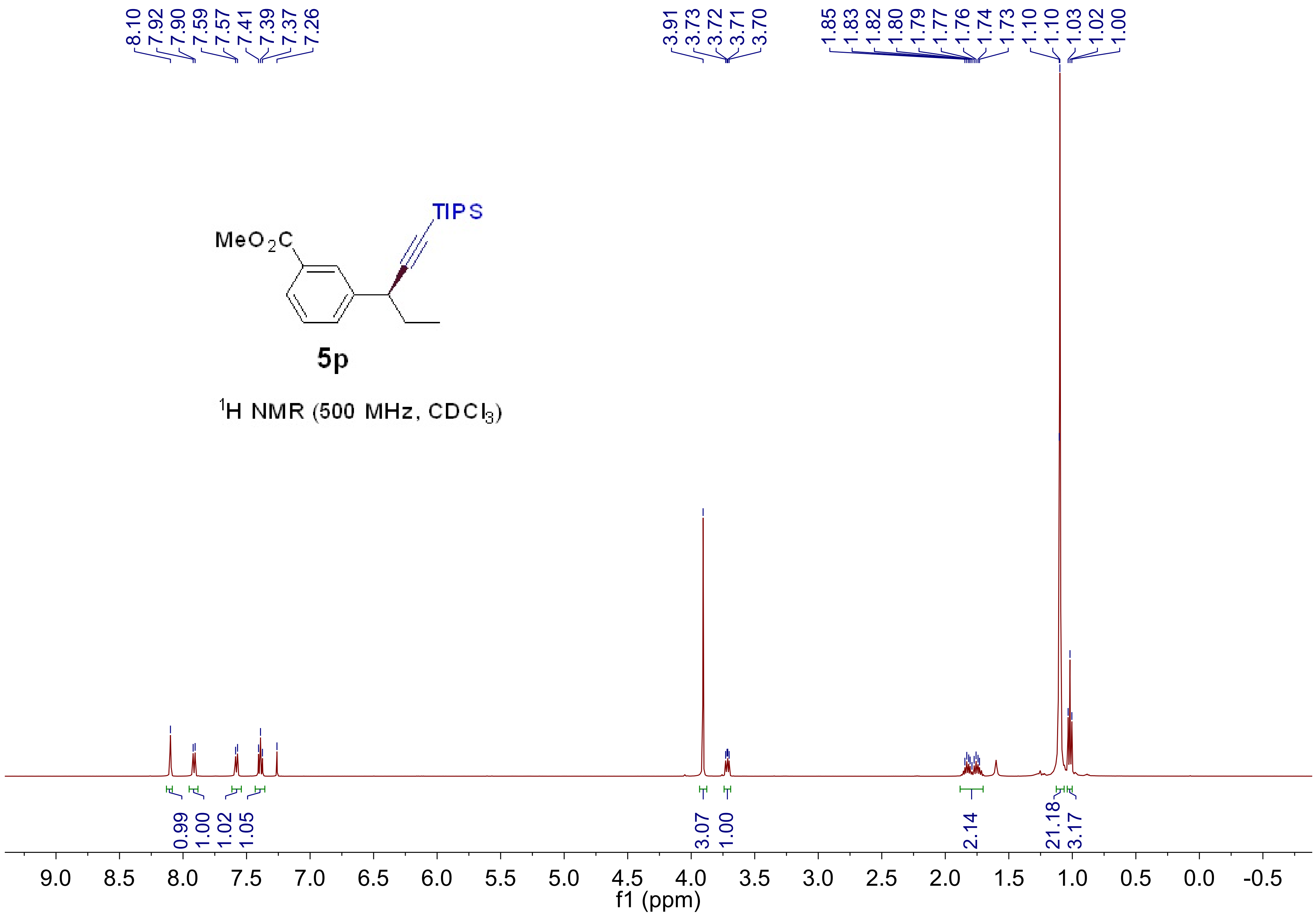

Supplementary Fig. 137. ^{1}H NMR (500 MHz, $CDCl_3$) spectra for compound **5p**

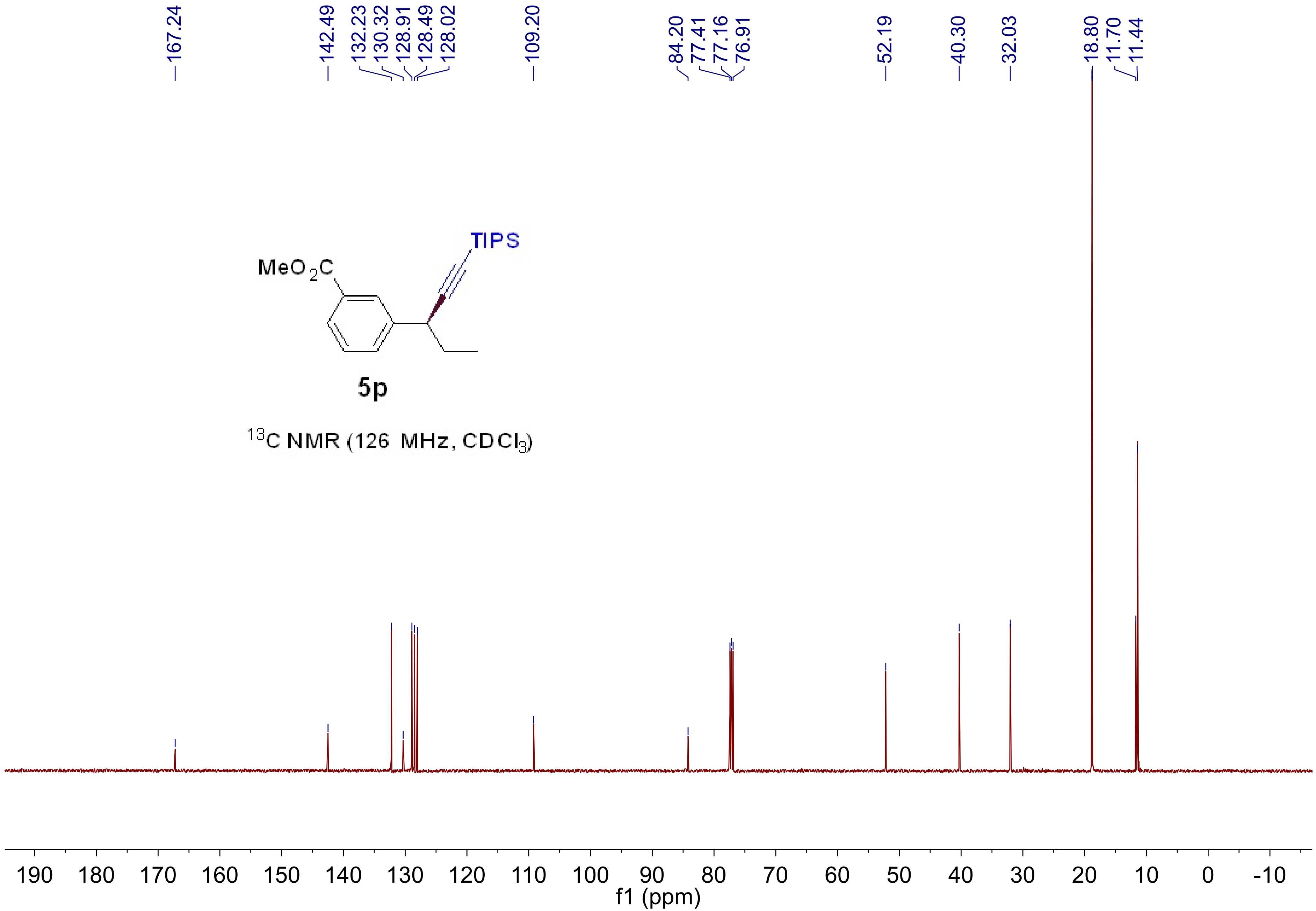

Supplementary Fig. 138. ^{13}C NMR (126 MHz, $CDCl_3$) spectra for compound **5p**

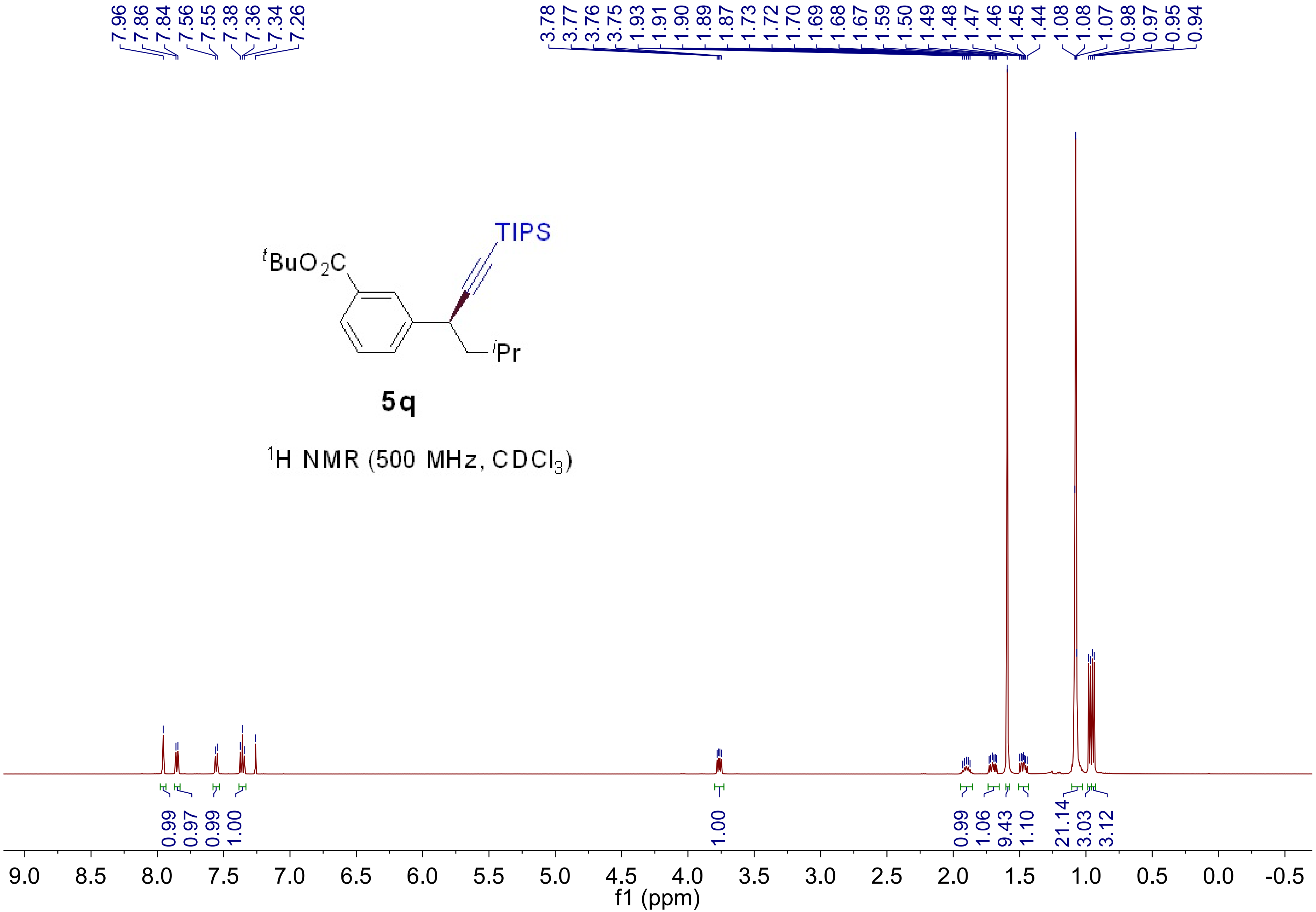

Supplementary Fig. 139. ^{1}H NMR (500 MHz, $CDCl_3$) spectra for compound **5q**

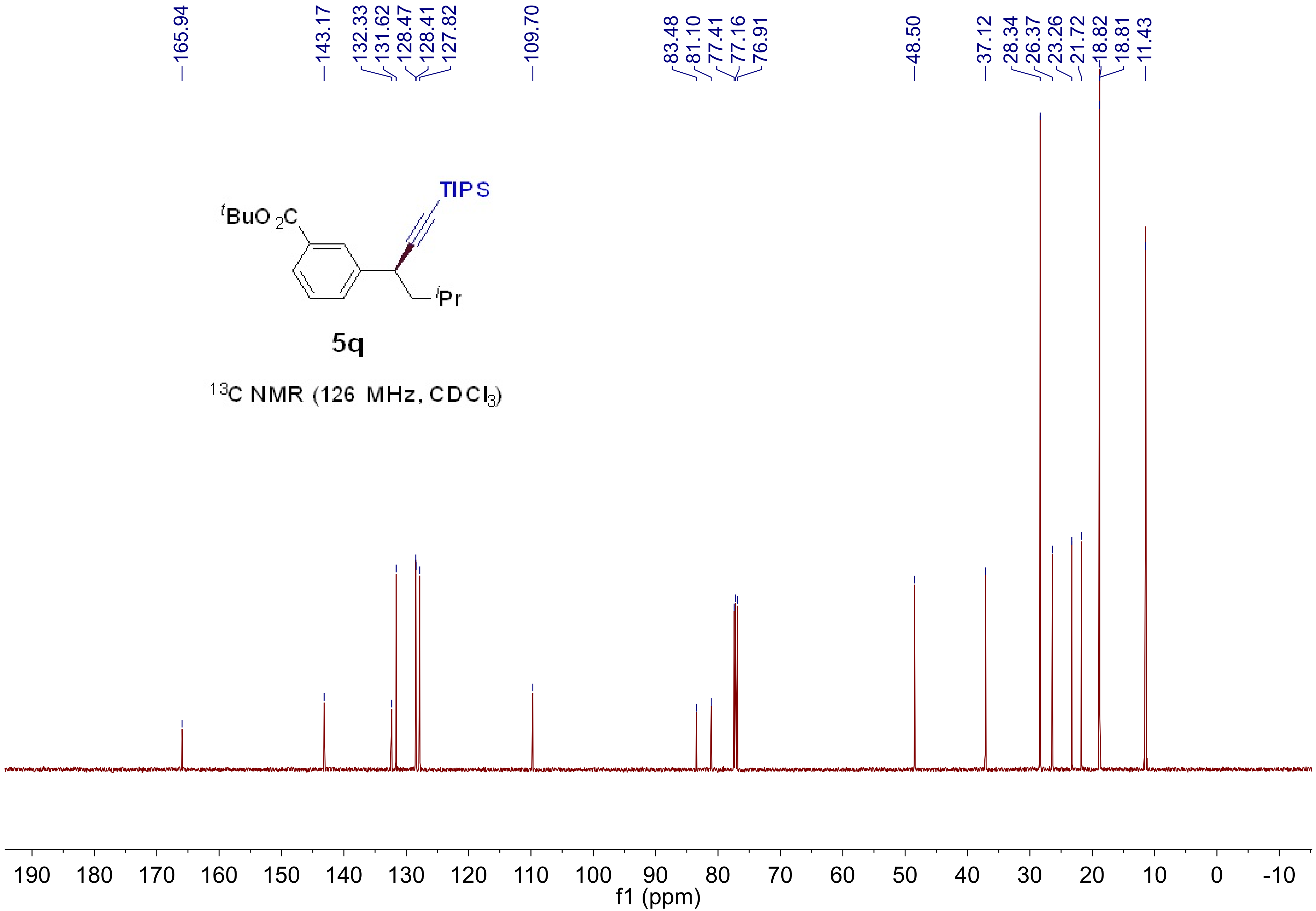

Supplementary Fig. 140. ^{13}C NMR (126 MHz, $CDCl_3$) spectra for compound **5q**

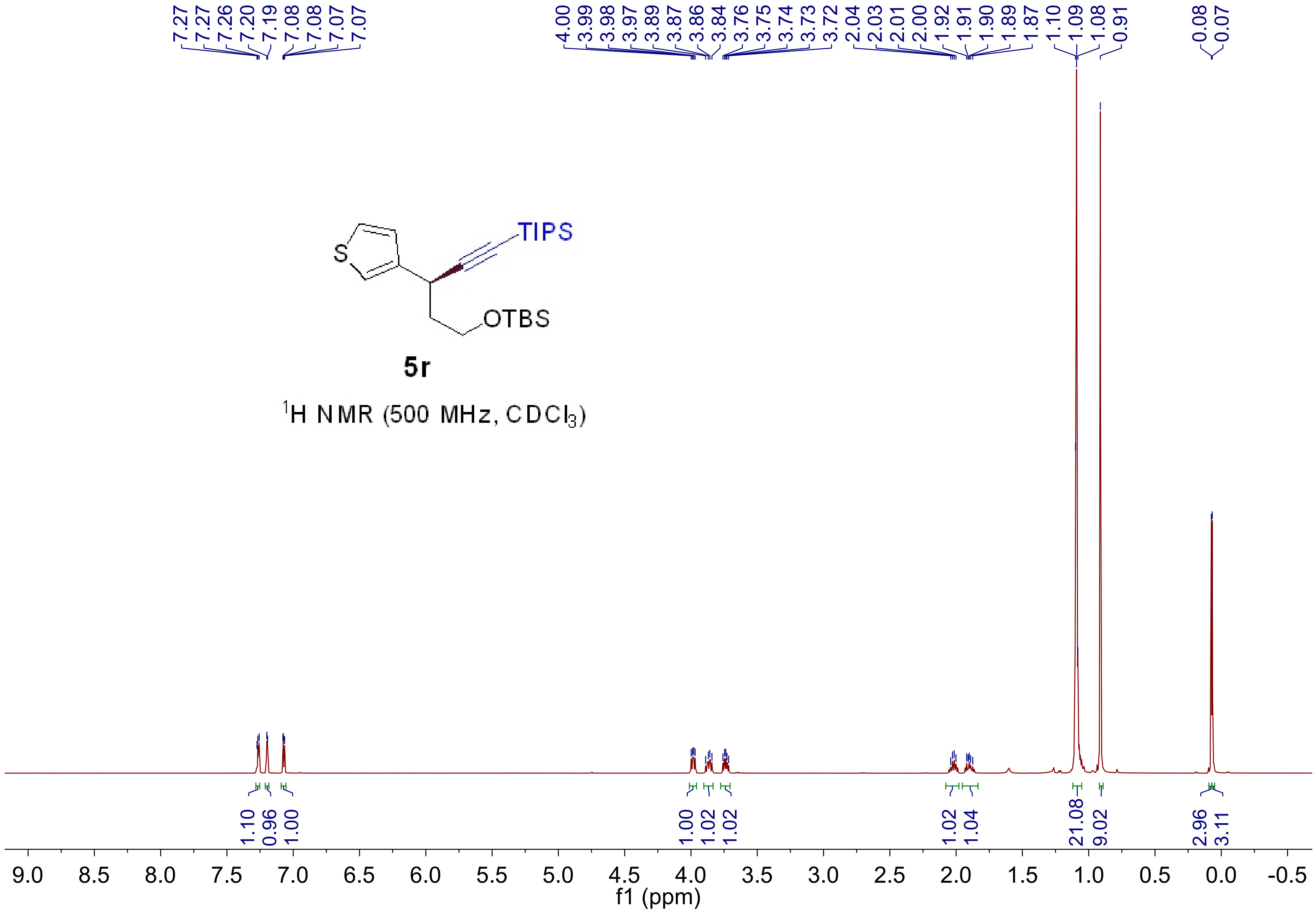

Supplementary Fig. 141. ^{1}H NMR (500 MHz, $CDCl_3$) spectra for compound **5r**

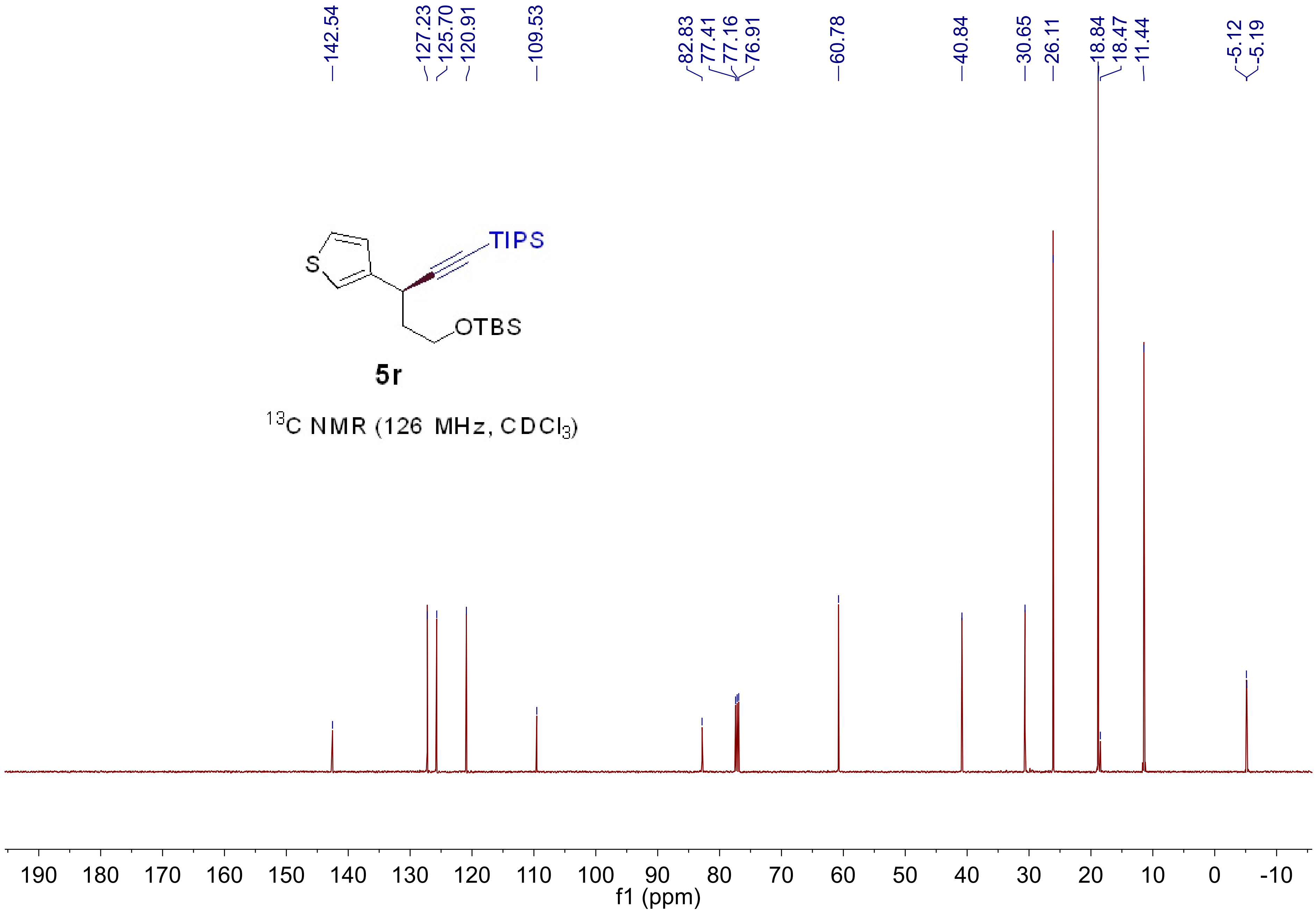

Supplementary Fig. 142. ^{13}C NMR (126 MHz, $CDCl_3$) spectra for compound **5r**

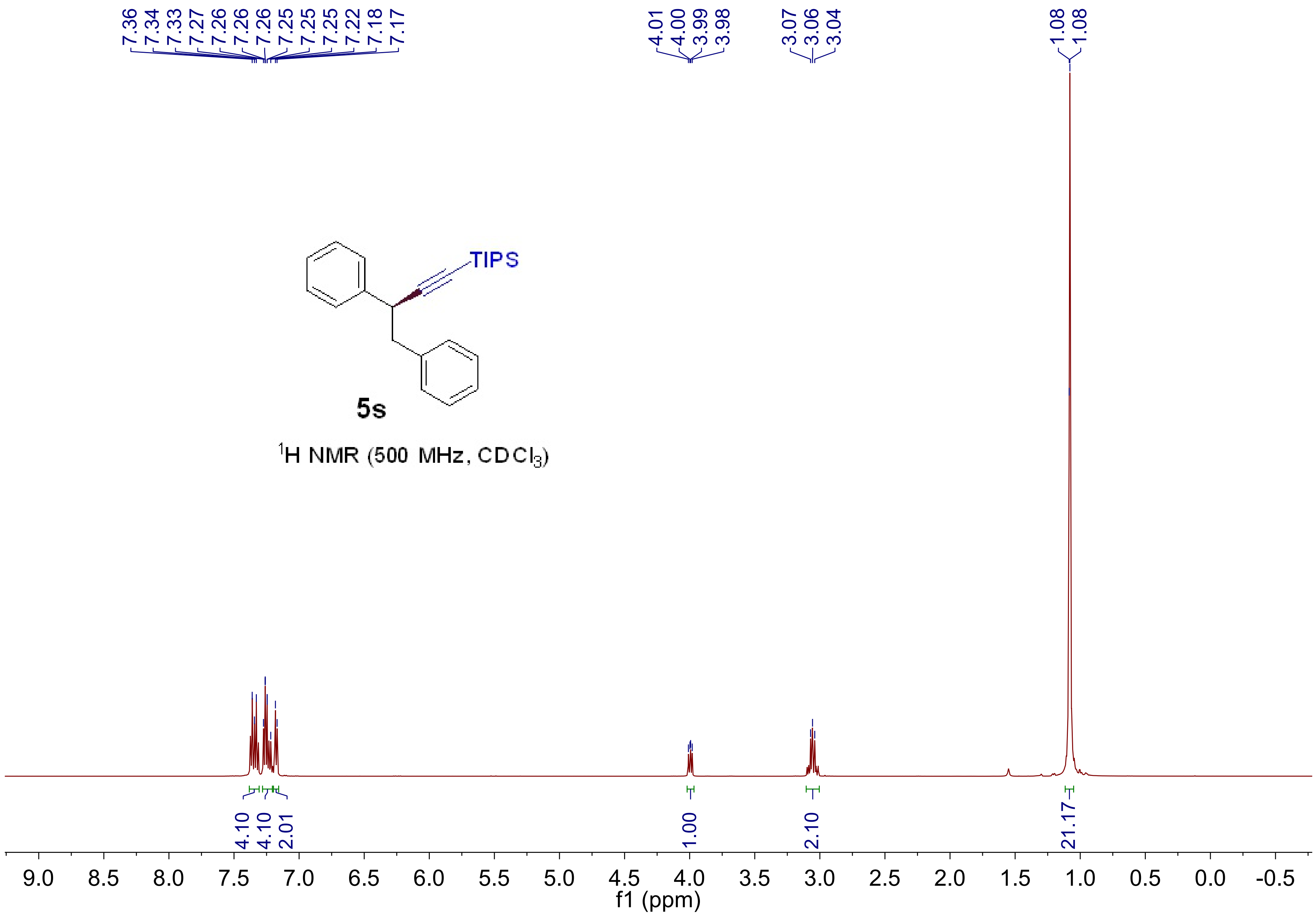

Supplementary Fig. 143. ^{1}H NMR (500 MHz, $CDCl_3$) spectra for compound **5s**

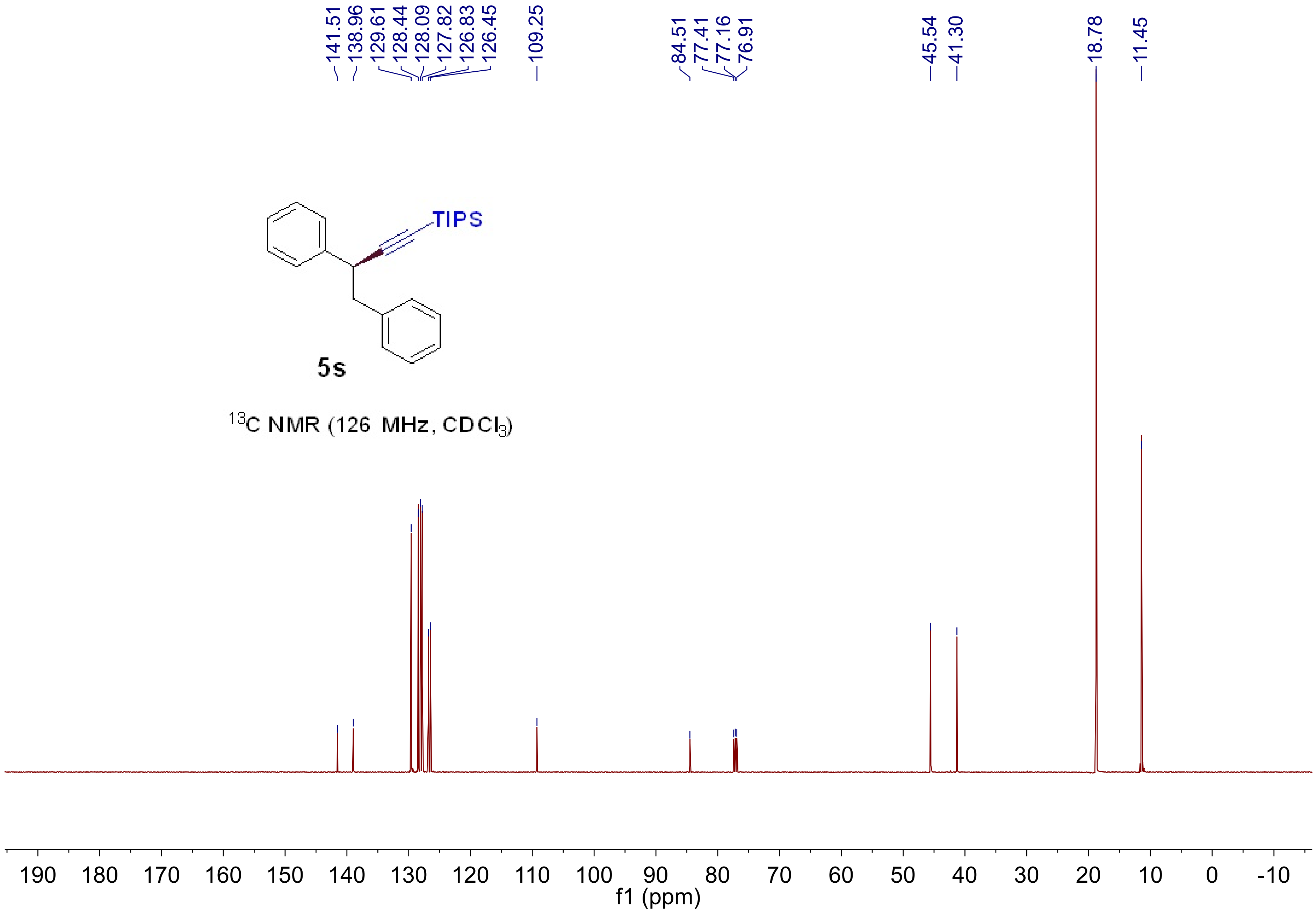

Supplementary Fig. 144. ^{13}C NMR (126 MHz, $CDCl_3$) spectra for compound **5s**

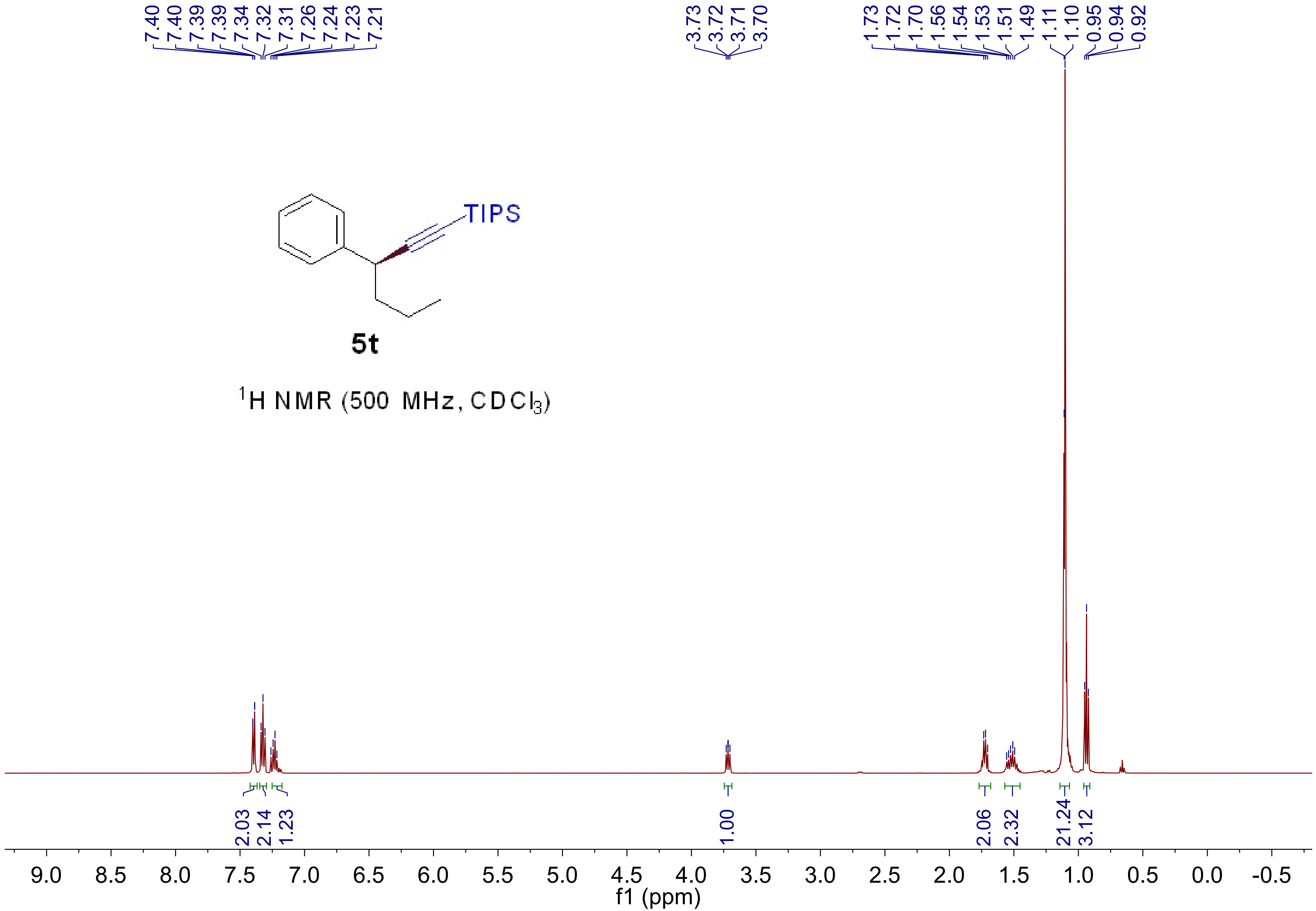

Supplementary Fig. 145. ^{1}H NMR (500 MHz, $CDCl_3$) spectra for compound **5t**

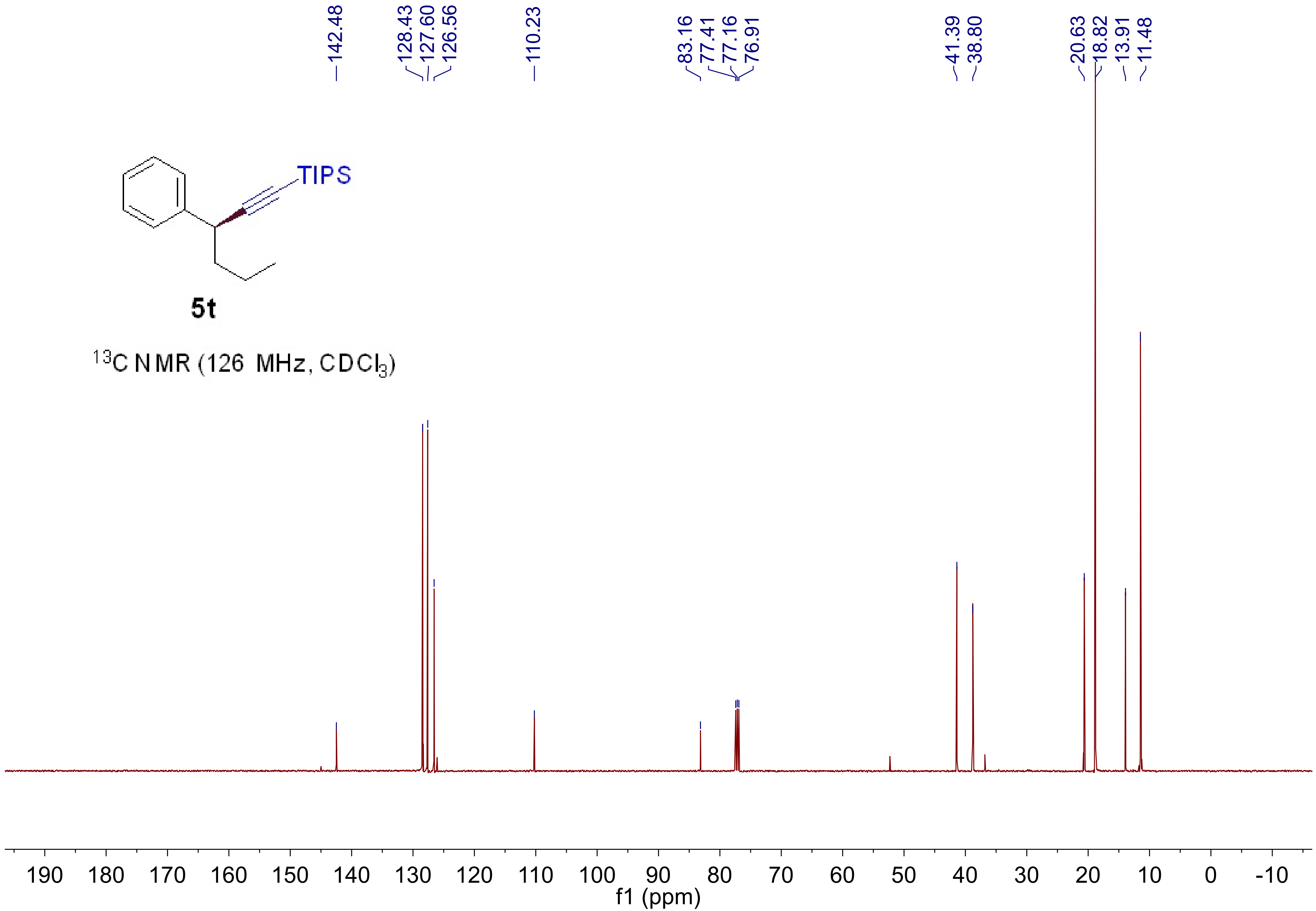

Supplementary Fig. 146. ^{13}C NMR (126 MHz, $CDCl_3$) spectra for compound **5t**

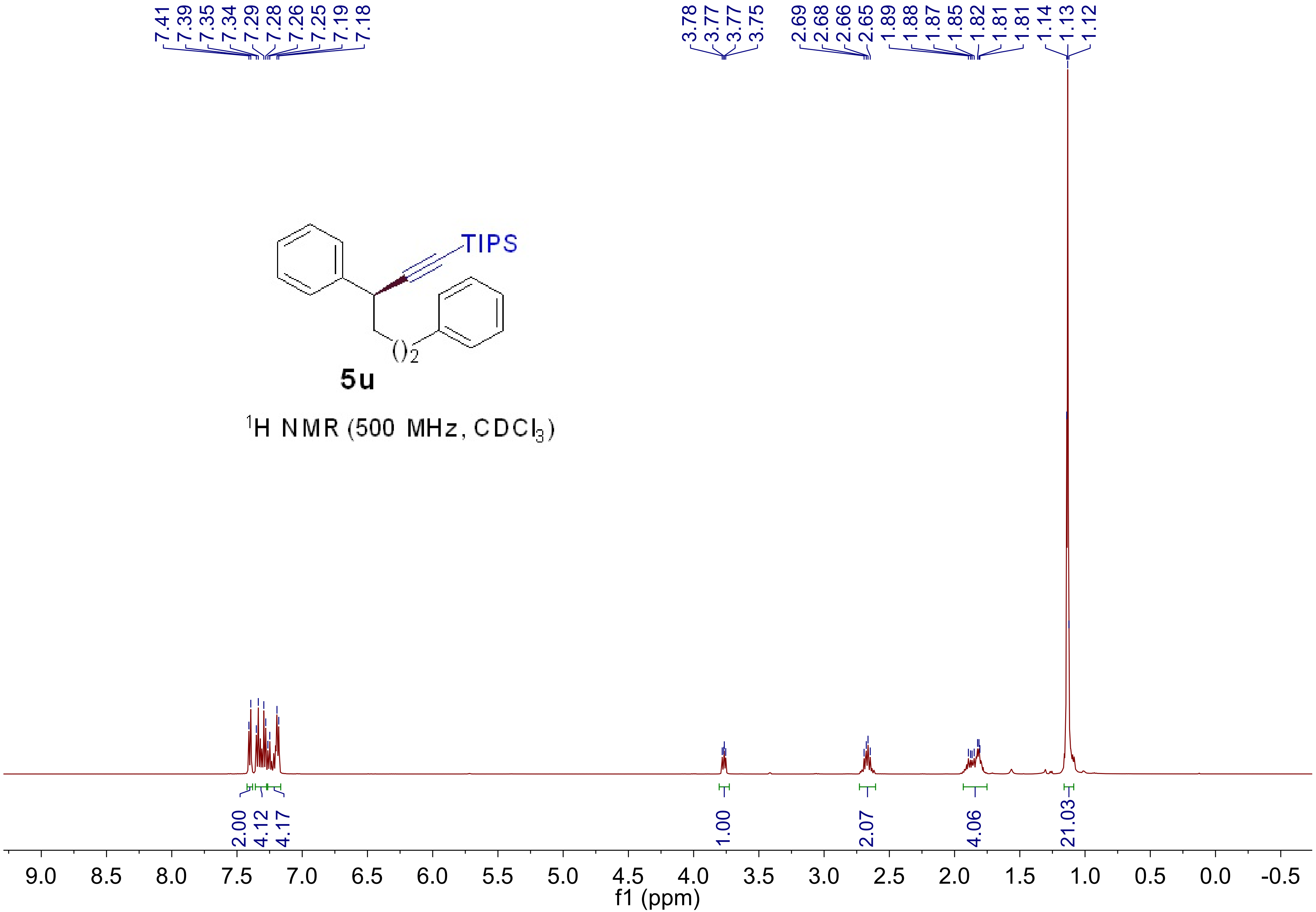

Supplementary Fig. 147. ^{1}H NMR (500 MHz, $CDCl_3$) spectra for compound **5u**

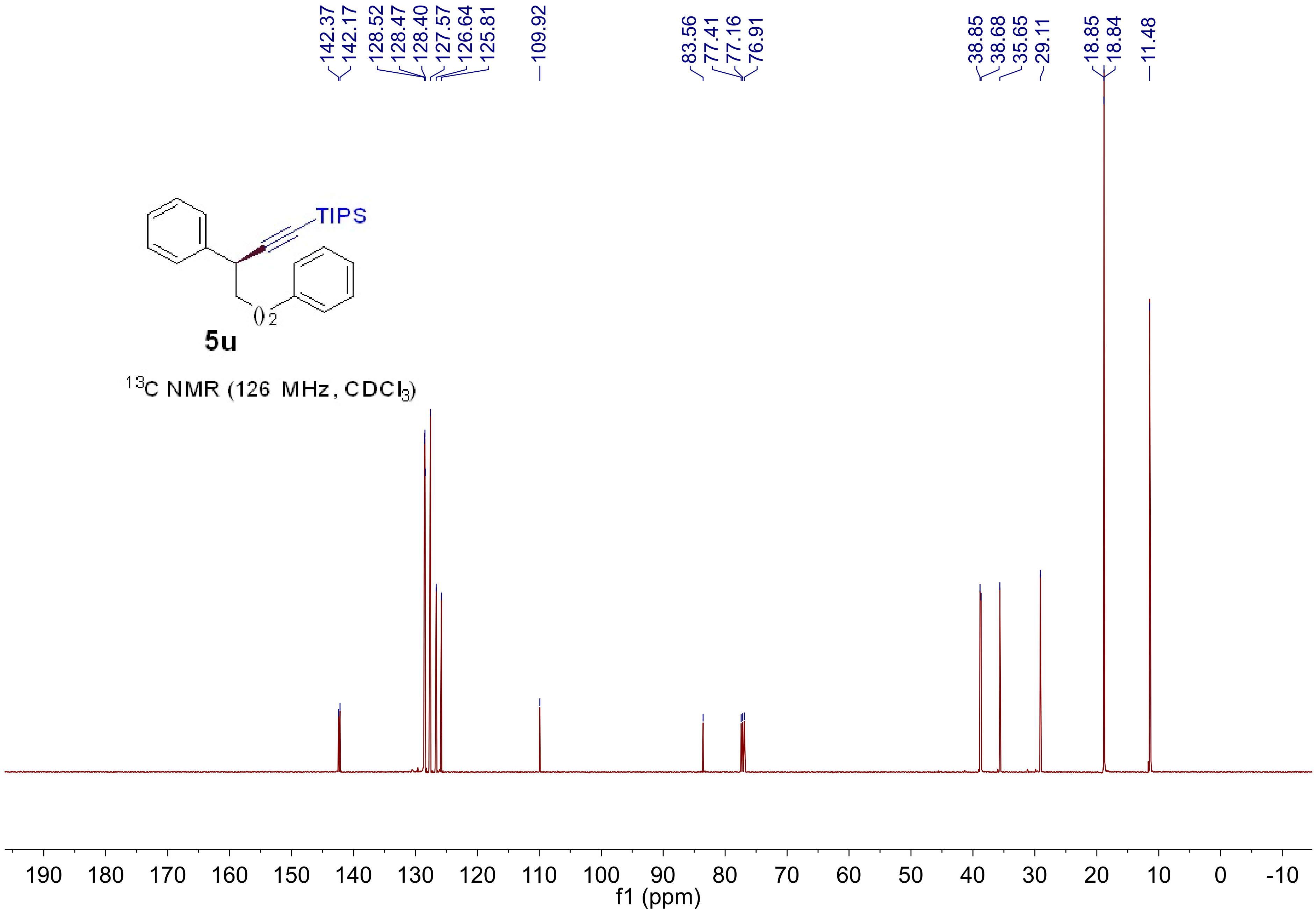

Supplementary Fig. 148. ^{13}C NMR (126 MHz, $CDCl_3$) spectra for compound **5u**

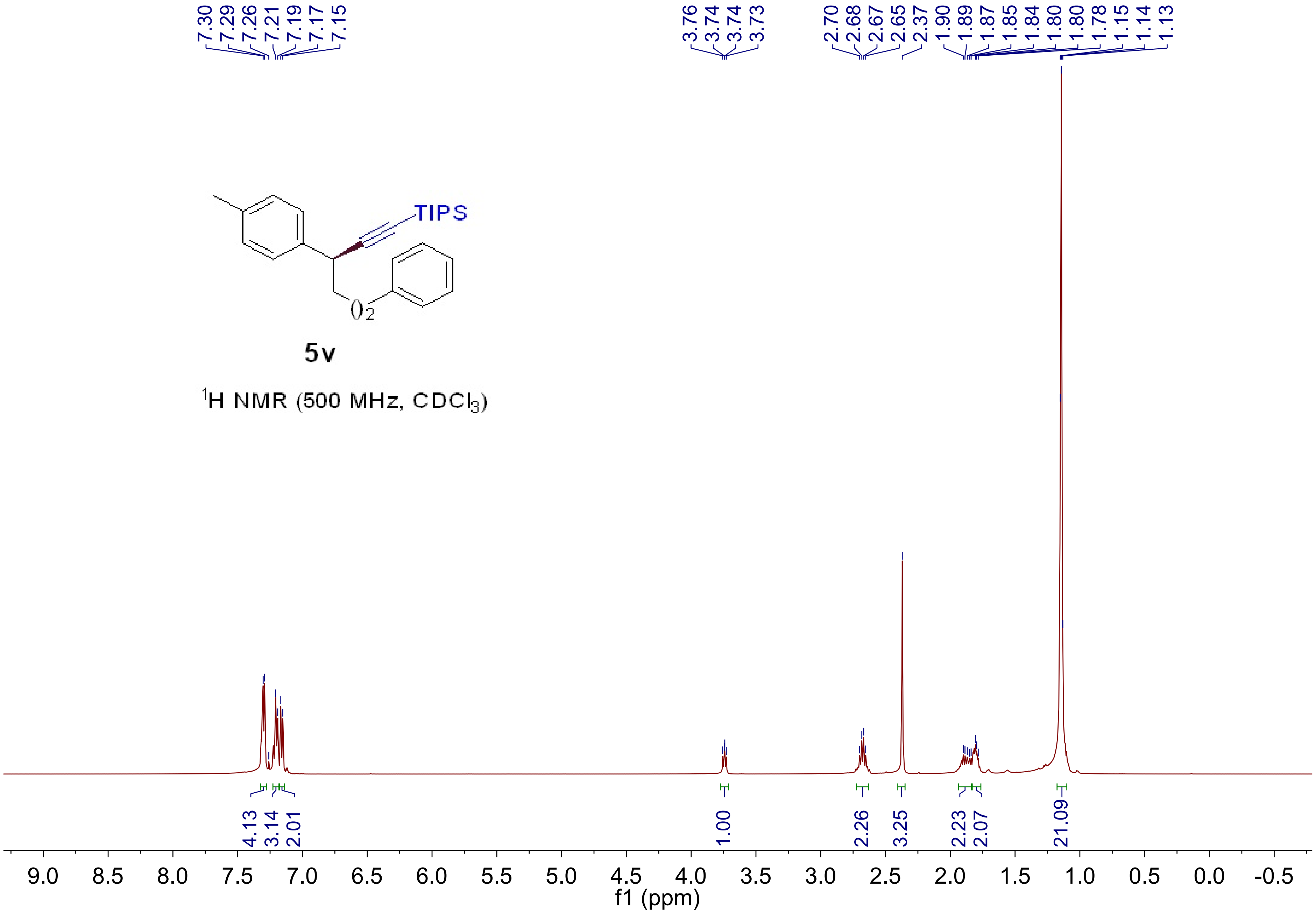

Supplementary Fig. 149. ^{1}H NMR (500 MHz, $CDCl_3$) spectra for compound **5v**

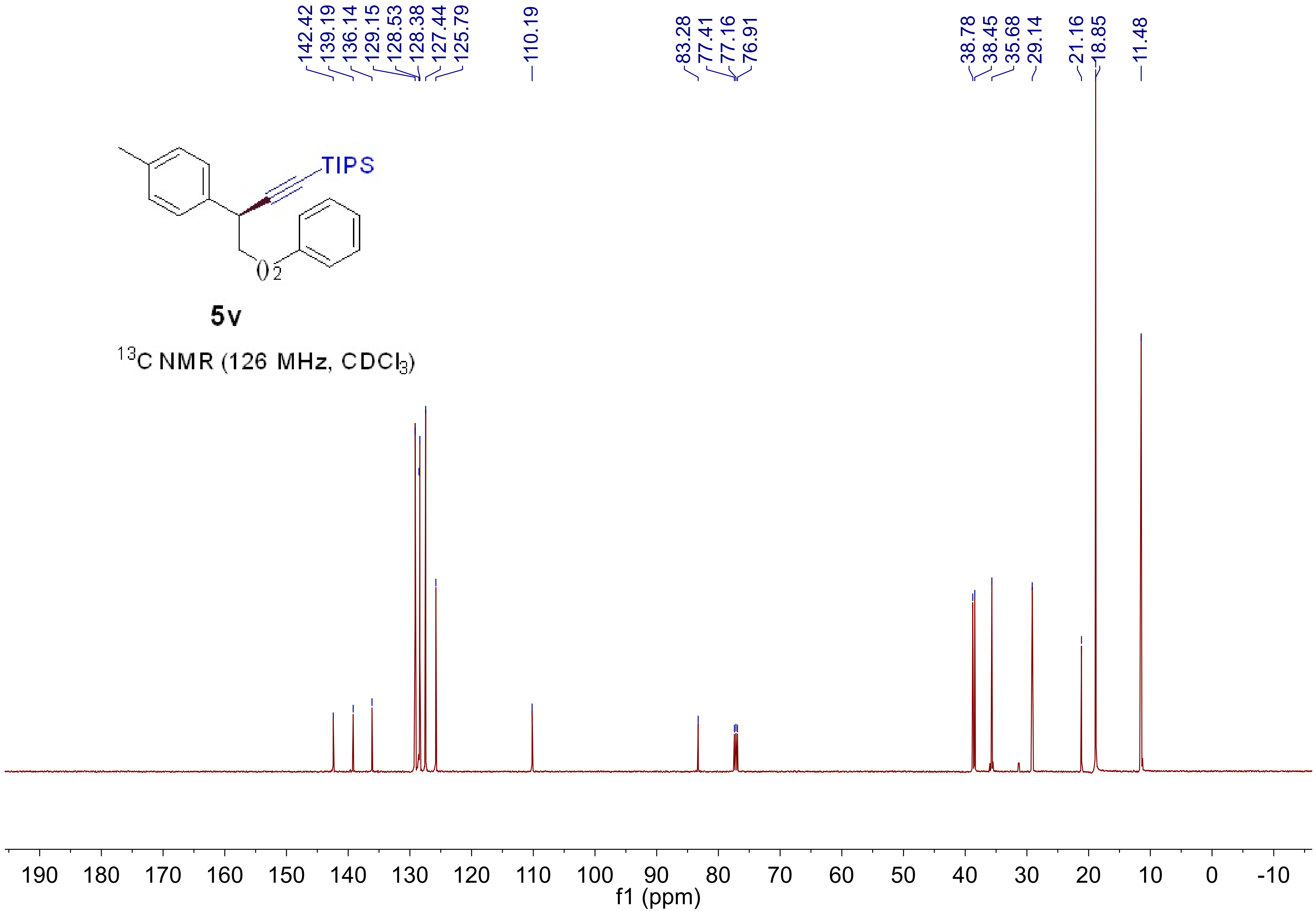

Supplementary Fig. 150. ^{13}C NMR (126 MHz, $CDCl_3$) spectra for compound **5v**

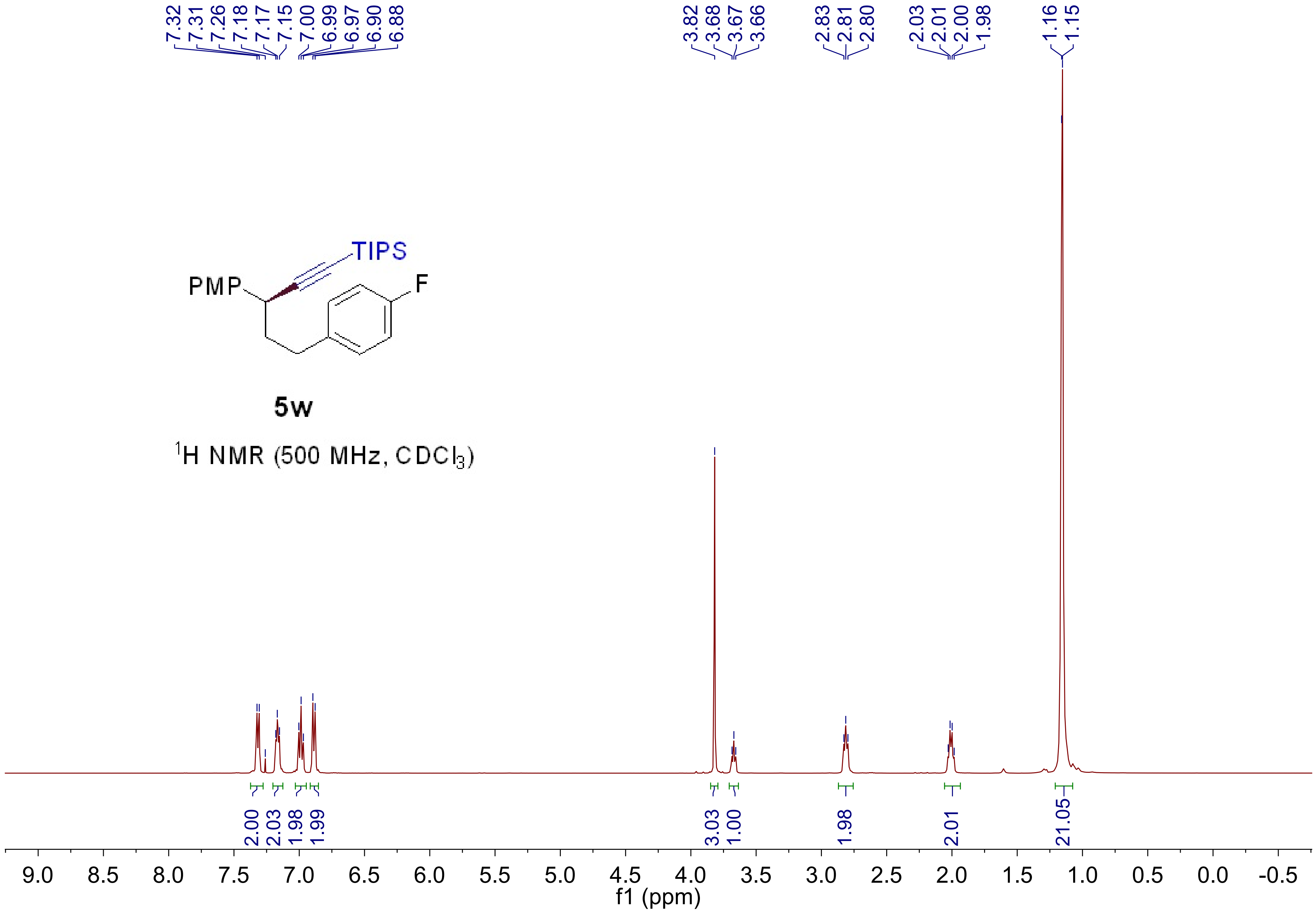

Supplementary Fig. 151. ^{1}H NMR (500 MHz, $CDCl_3$) spectra for compound **5w**

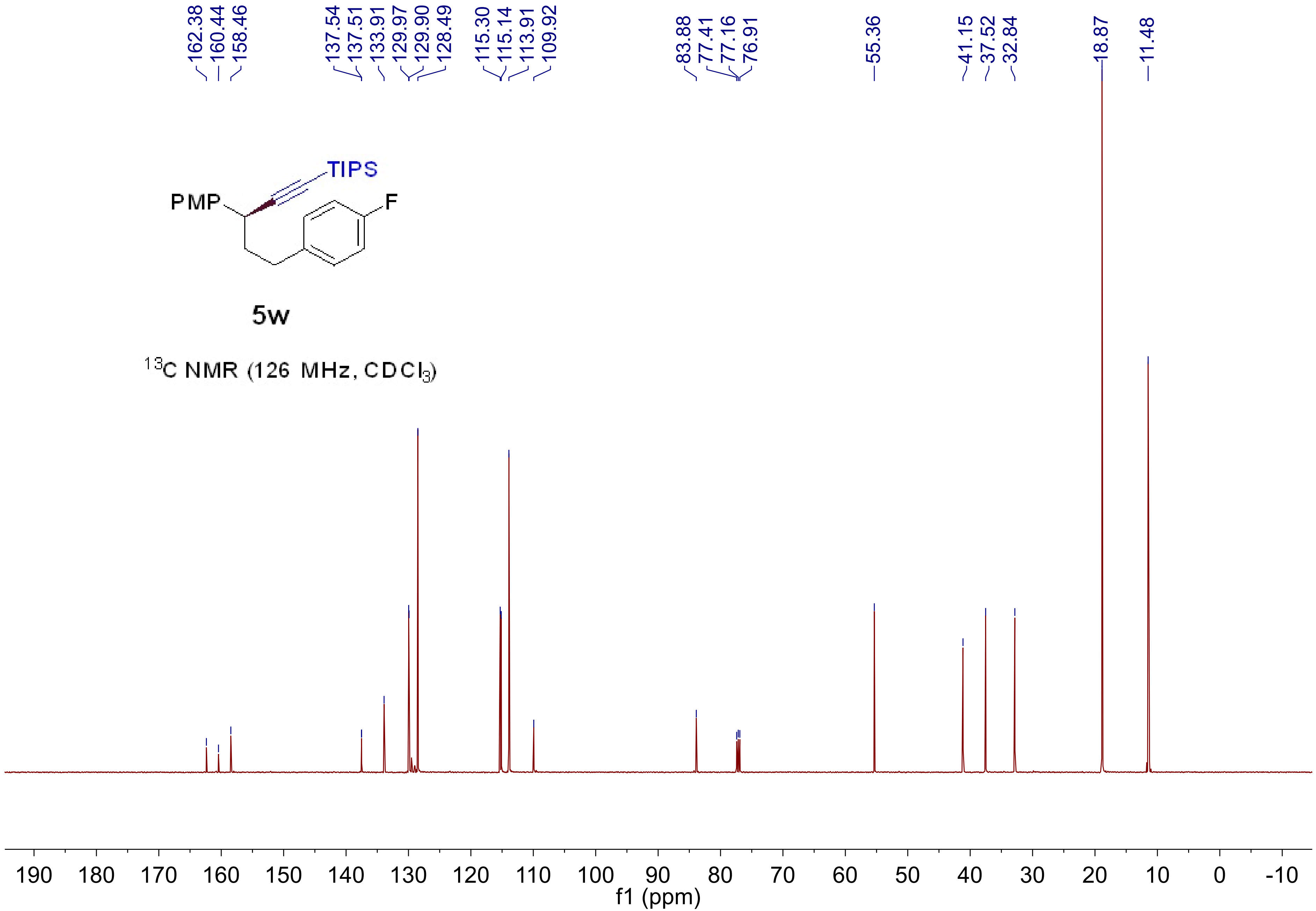

Supplementary Fig. 152. ^{13}C NMR (126 MHz, $CDCl_3$) spectra for compound **5w**

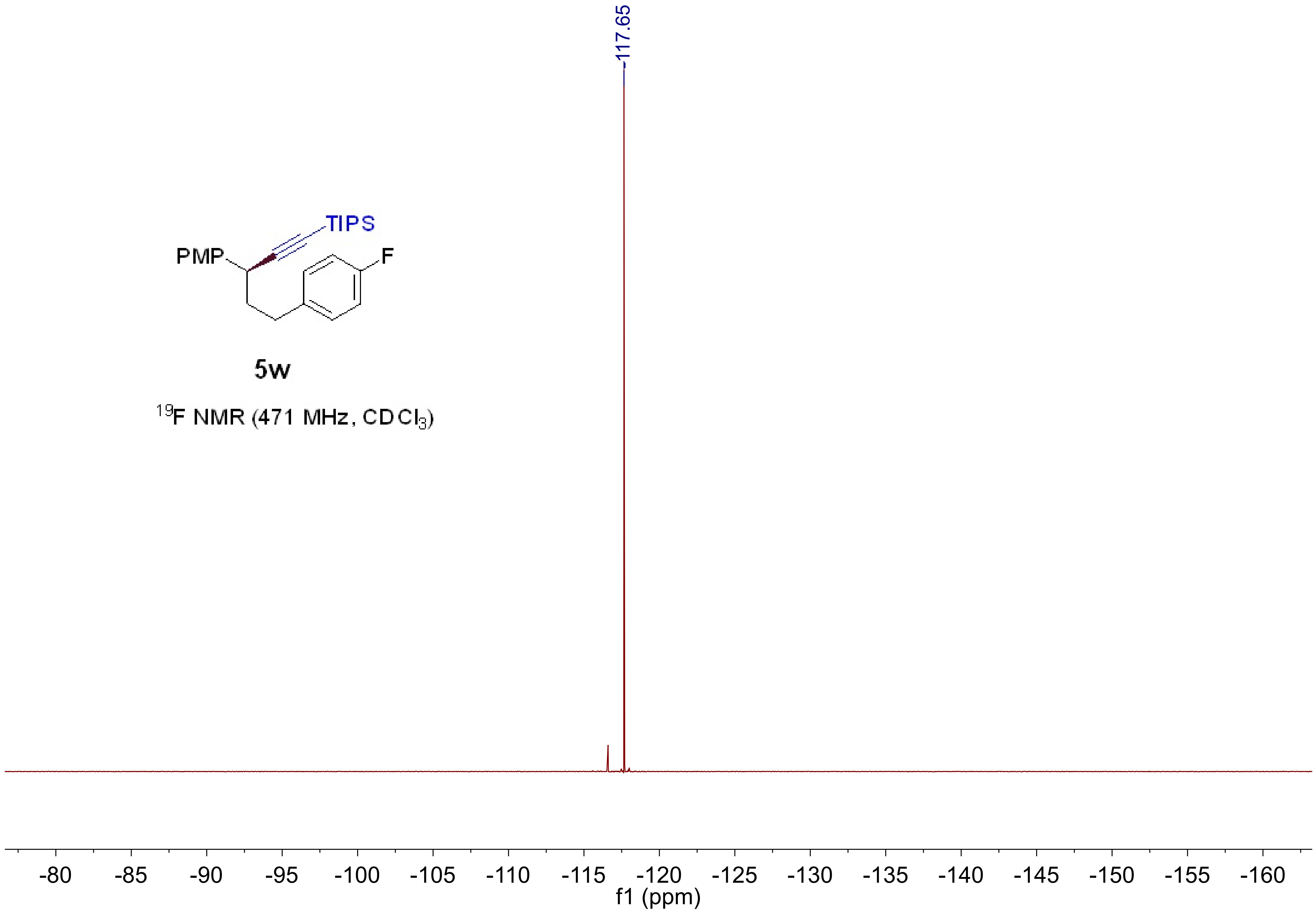

Supplementary Fig. 153. ^{19}F NMR (471 MHz, $CDCl_3$) spectra for compound **5w**

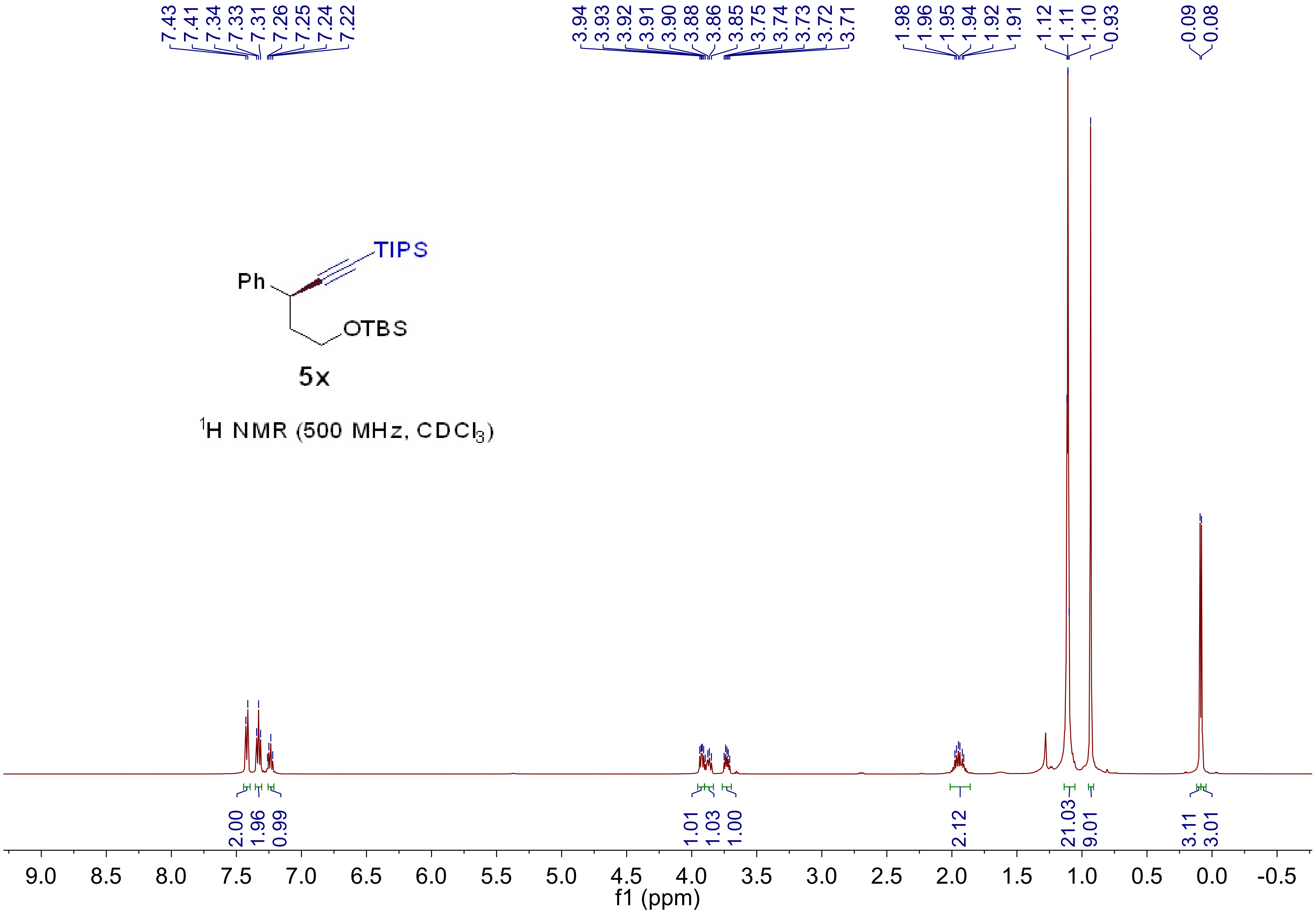

Supplementary Fig. 154. ^{1}H NMR (500 MHz, $CDCl_3$) spectra for compound **5x**

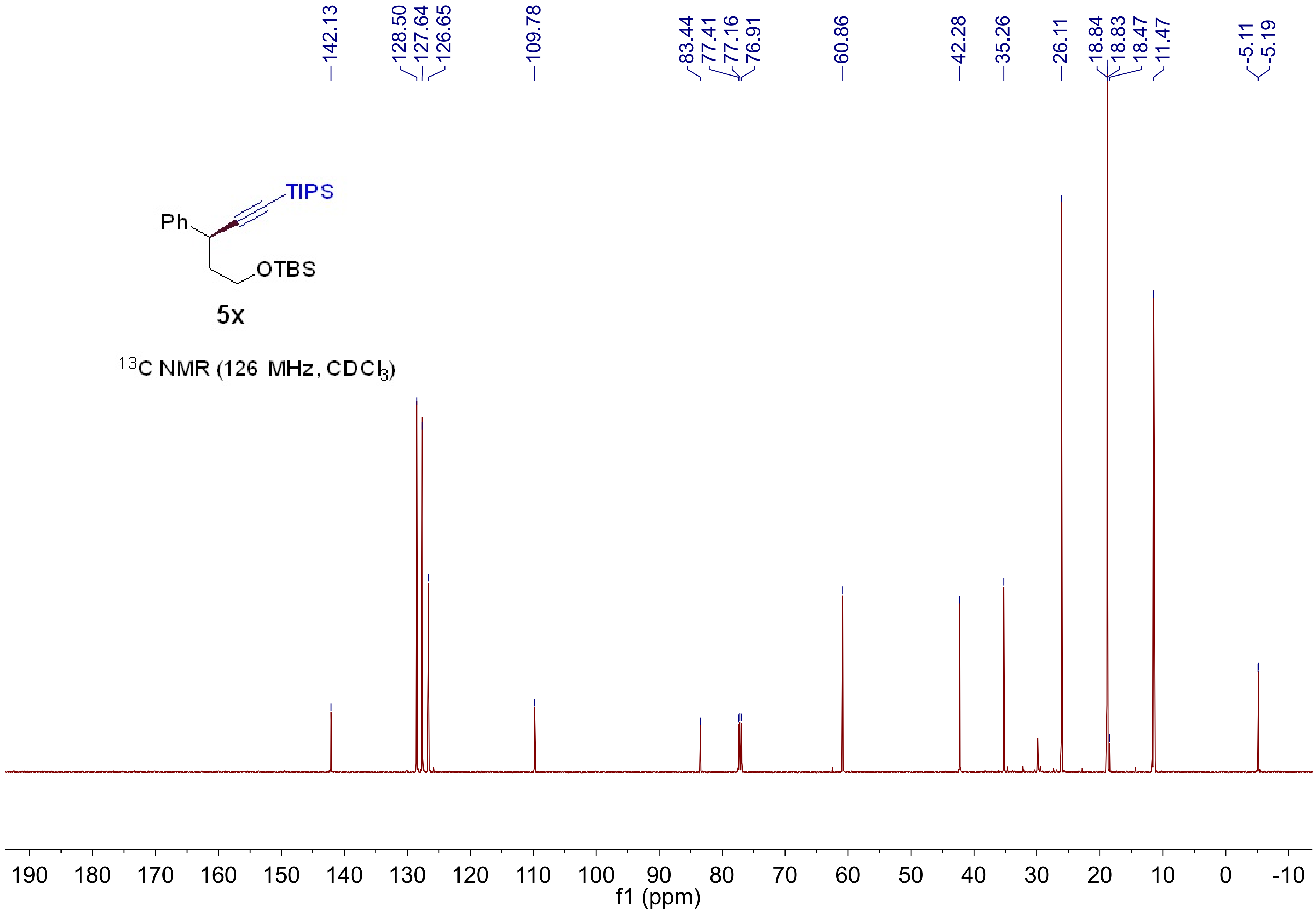

Supplementary Fig. 155. ^{13}C NMR (126 MHz, $CDCl_3$) spectra for compound **5x**

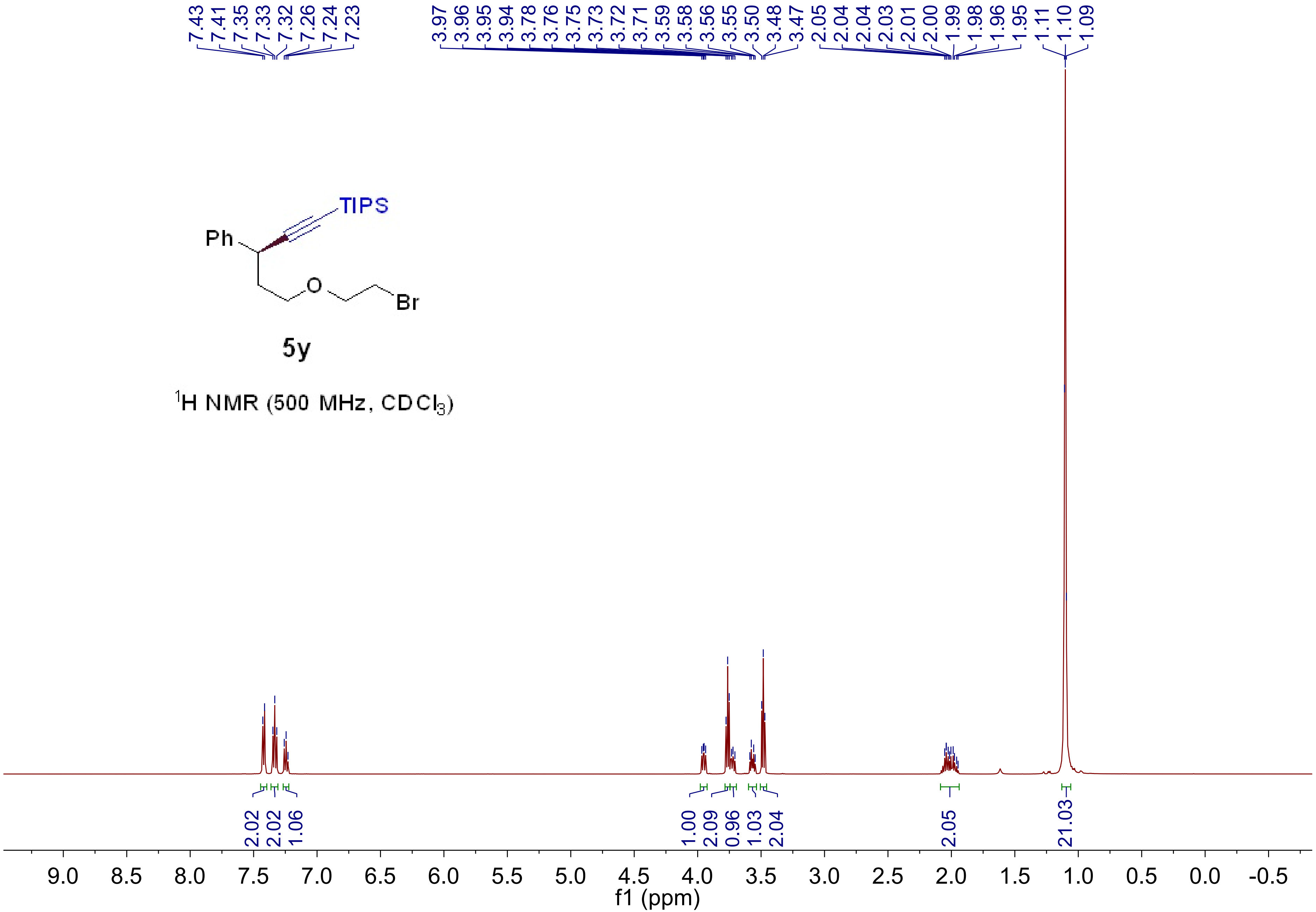

Supplementary Fig. 156. ^{1}H NMR (500 MHz, $CDCl_3$) spectra for compound **5y**

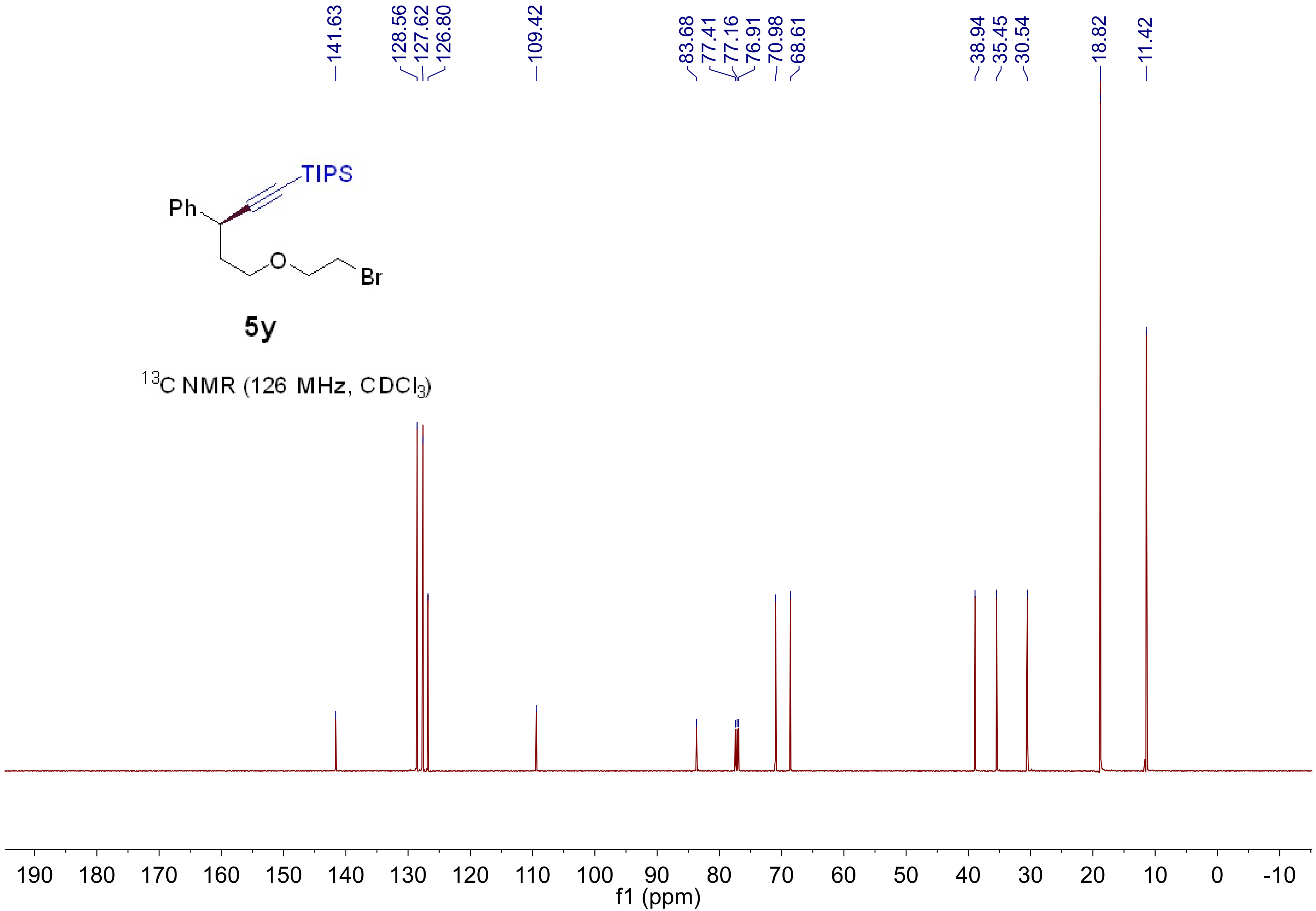

Supplementary Fig. 157. ^{13}C NMR (126 MHz, $CDCl_3$) spectra for compound **5y**

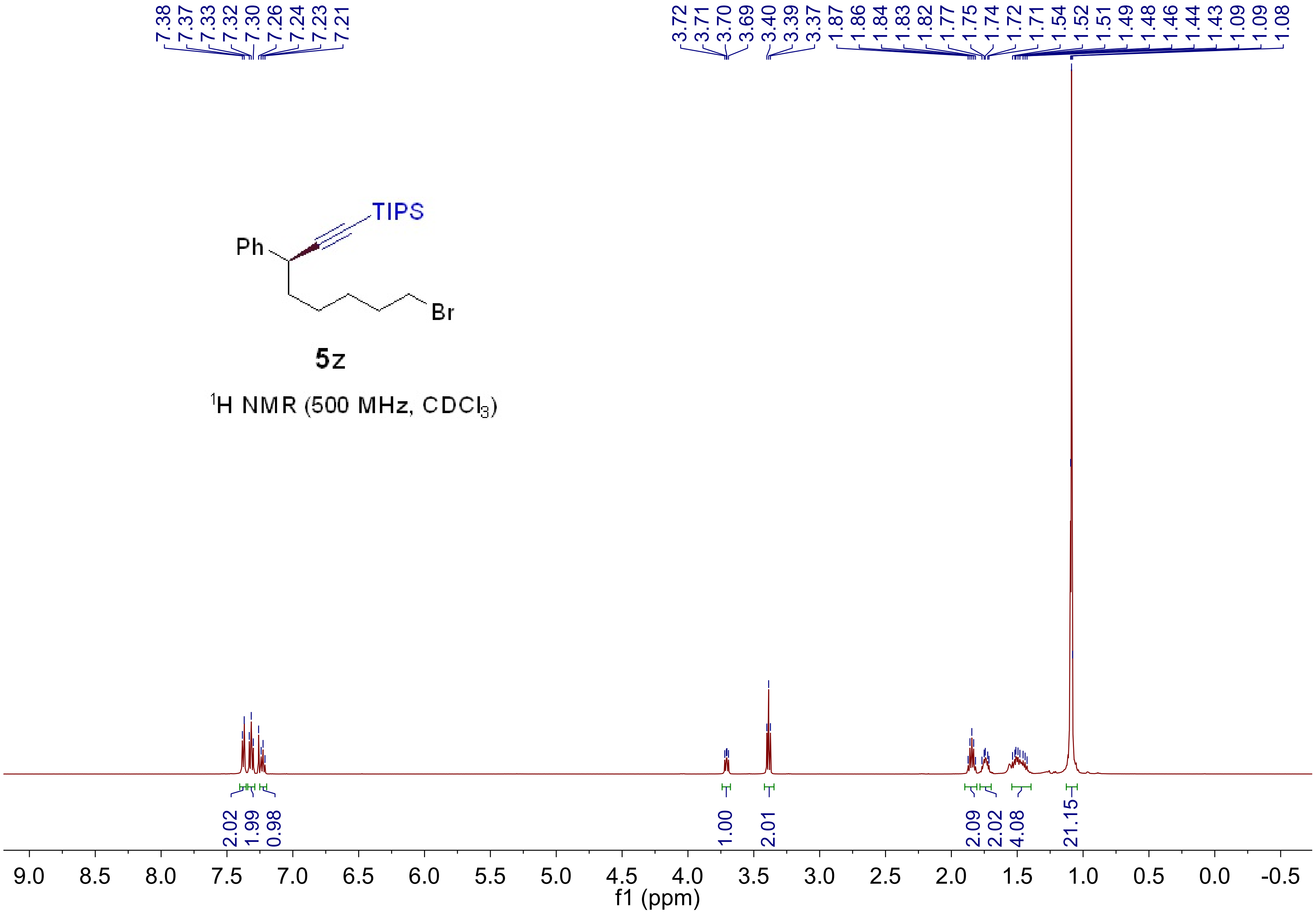

Supplementary Fig. 158. ^{1}H NMR (500 MHz, $CDCl_3$) spectra for compound **5z**

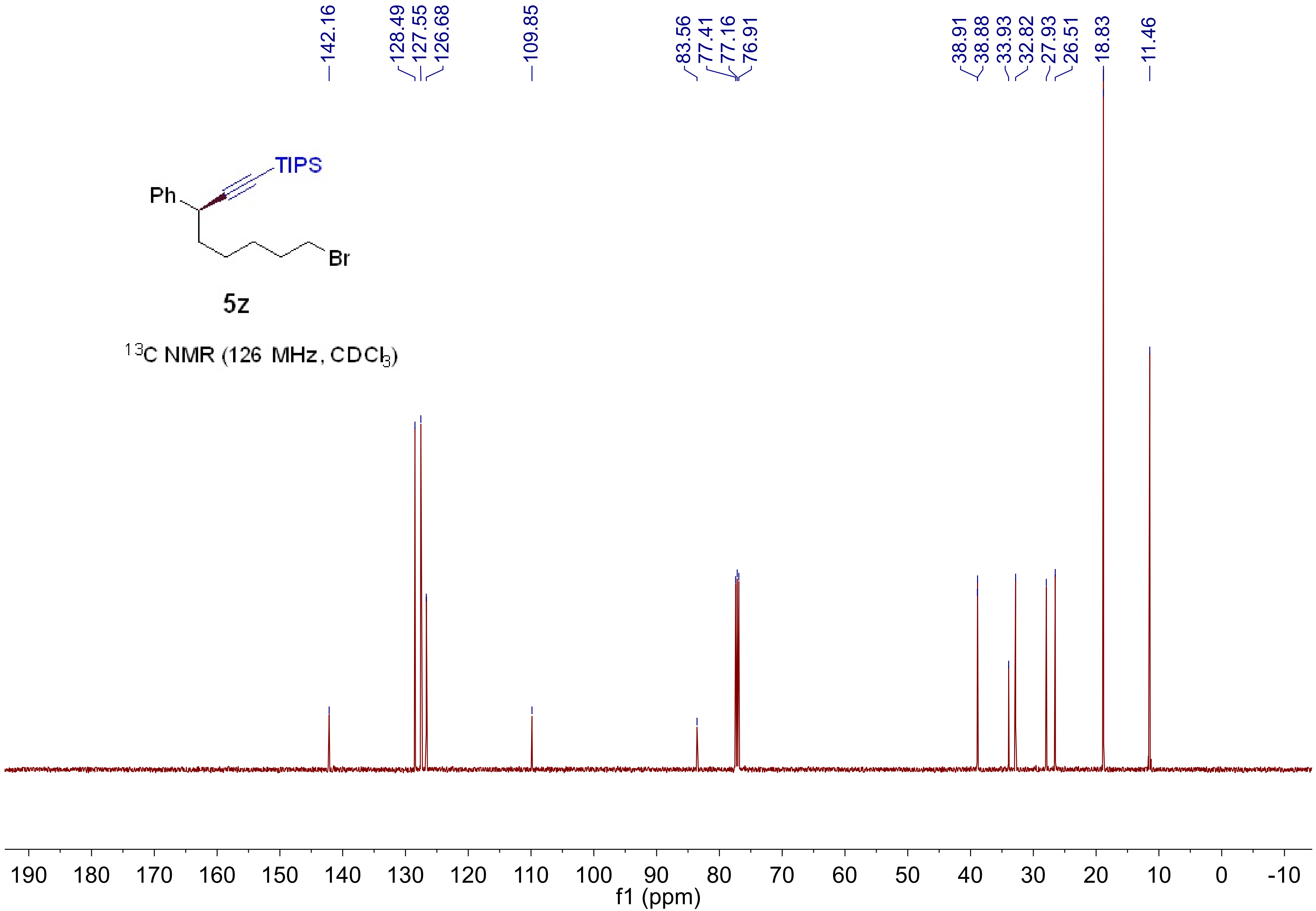

Supplementary Fig. 159. ^{13}C NMR (126 MHz, $CDCl_3$) spectra for compound **5z**

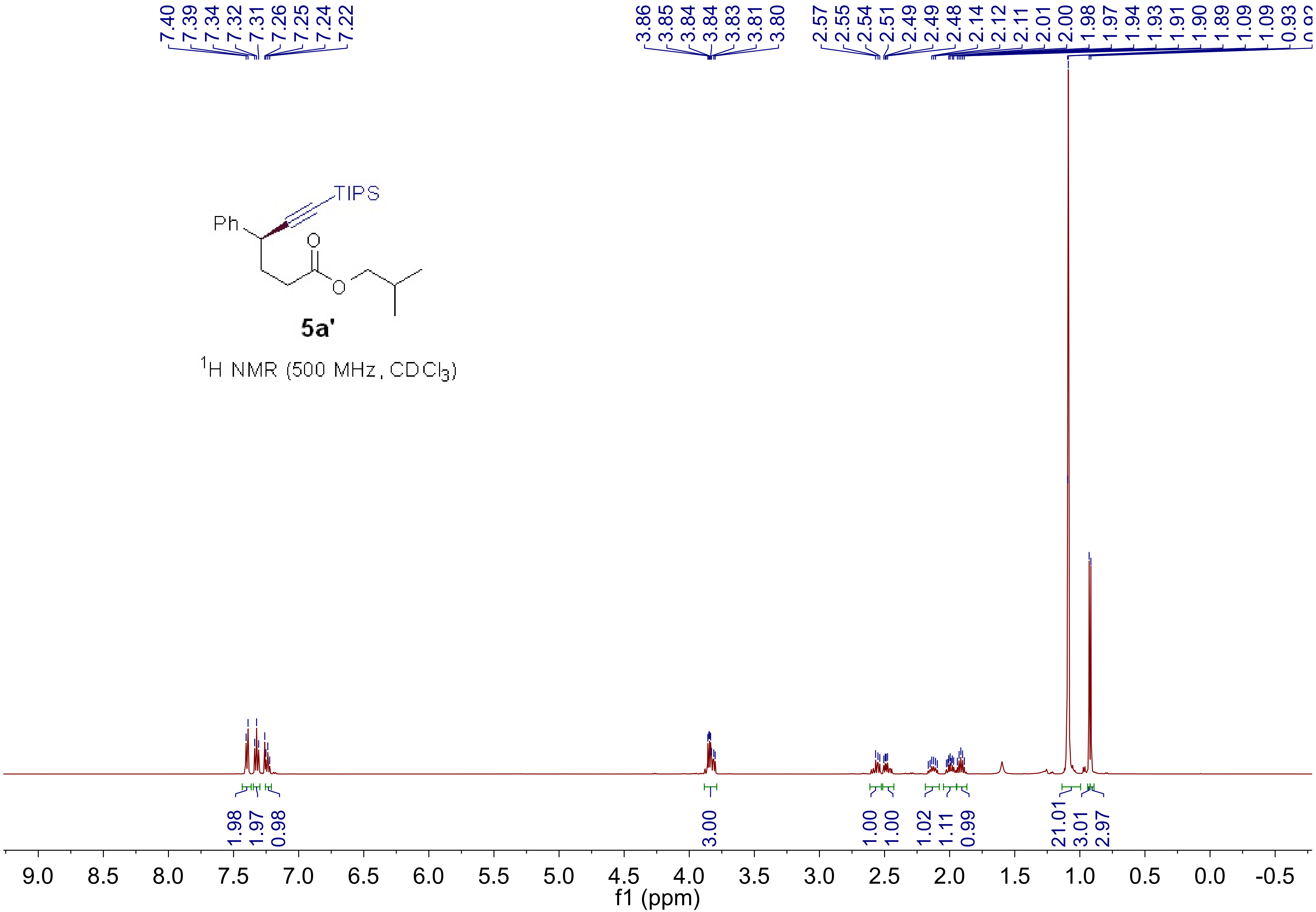

Supplementary Fig. 160. ^{1}H NMR (500 MHz, $CDCl_3$) spectra for compound **5a'**

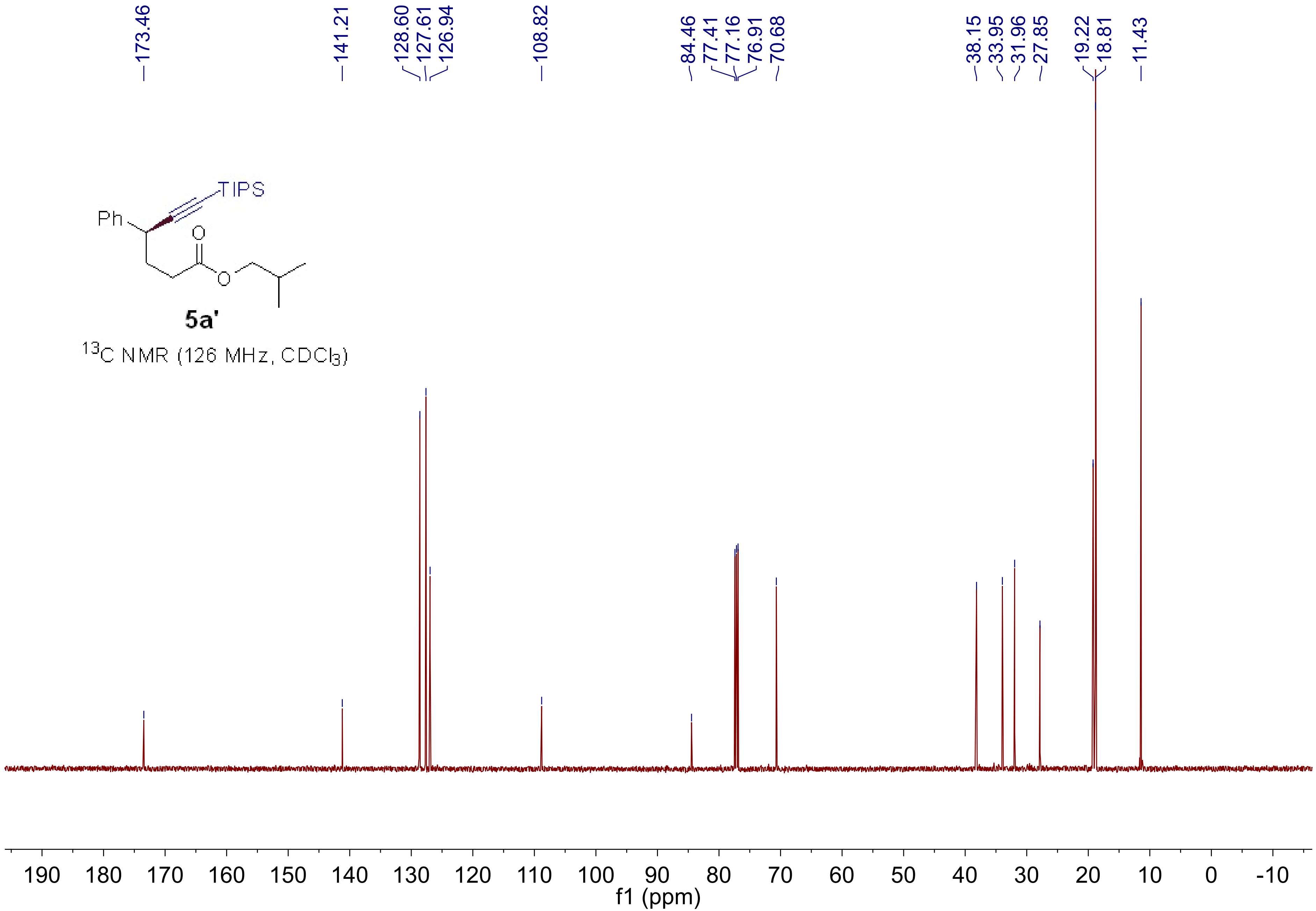

Supplementary Fig. 161. ^{13}C NMR (126 MHz, $CDCl_3$) spectra for compound **5a'**

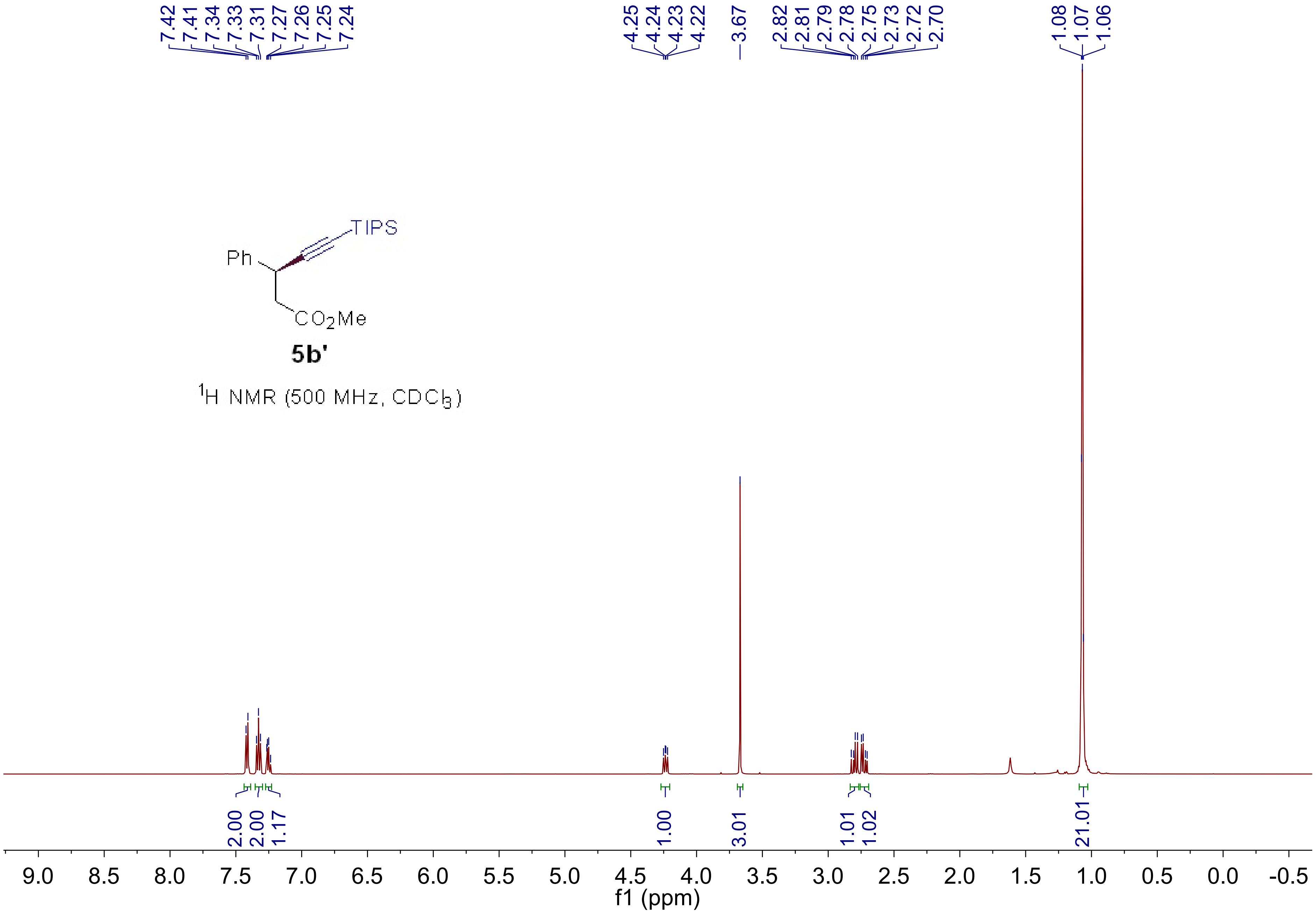

Supplementary Fig. 162. ^{1}H NMR (500 MHz, $CDCl_3$) spectra for compound **5b'**

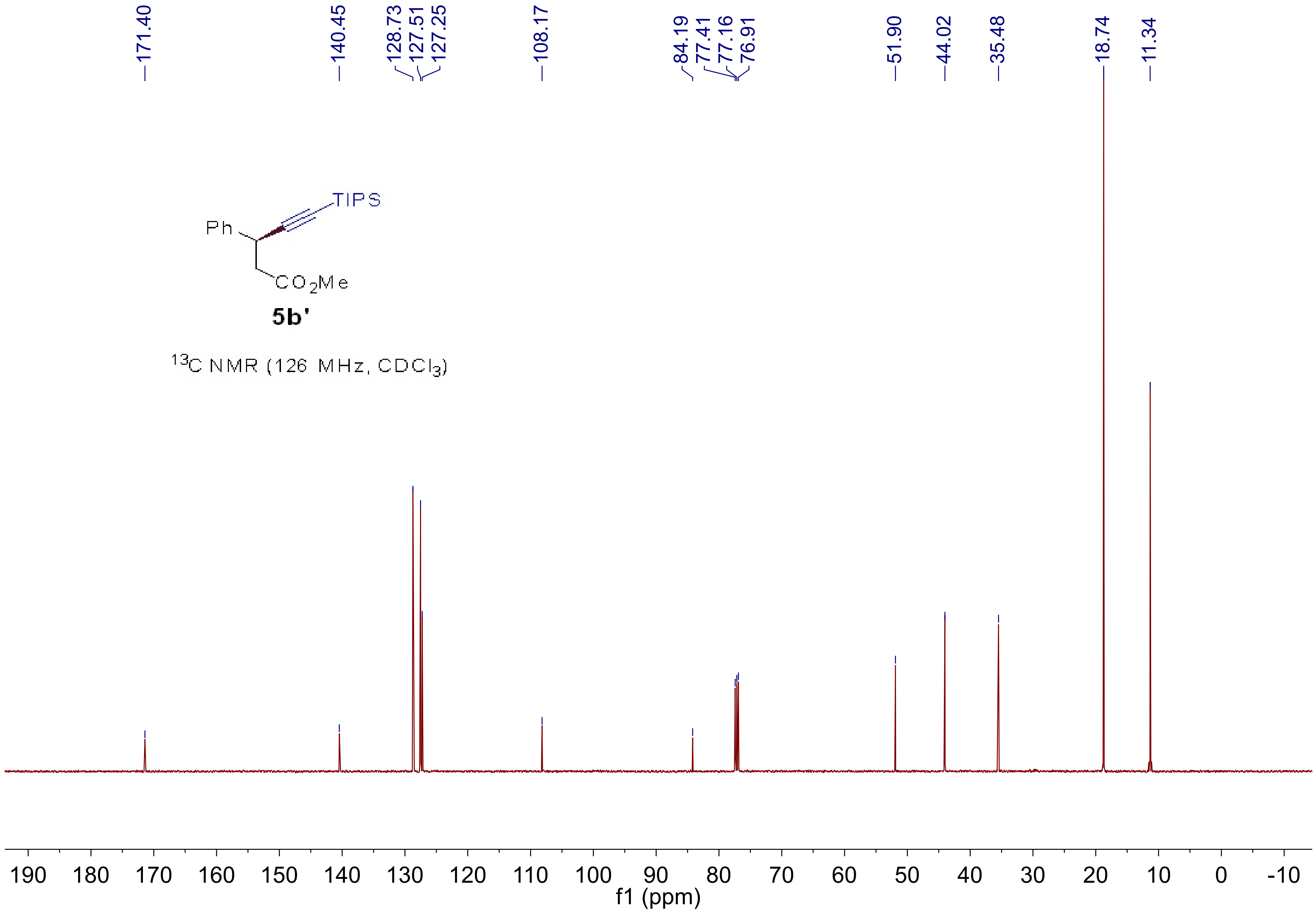

Supplementary Fig. 163. ^{13}C NMR (126 MHz, $CDCl_3$) spectra for compound **5b'**

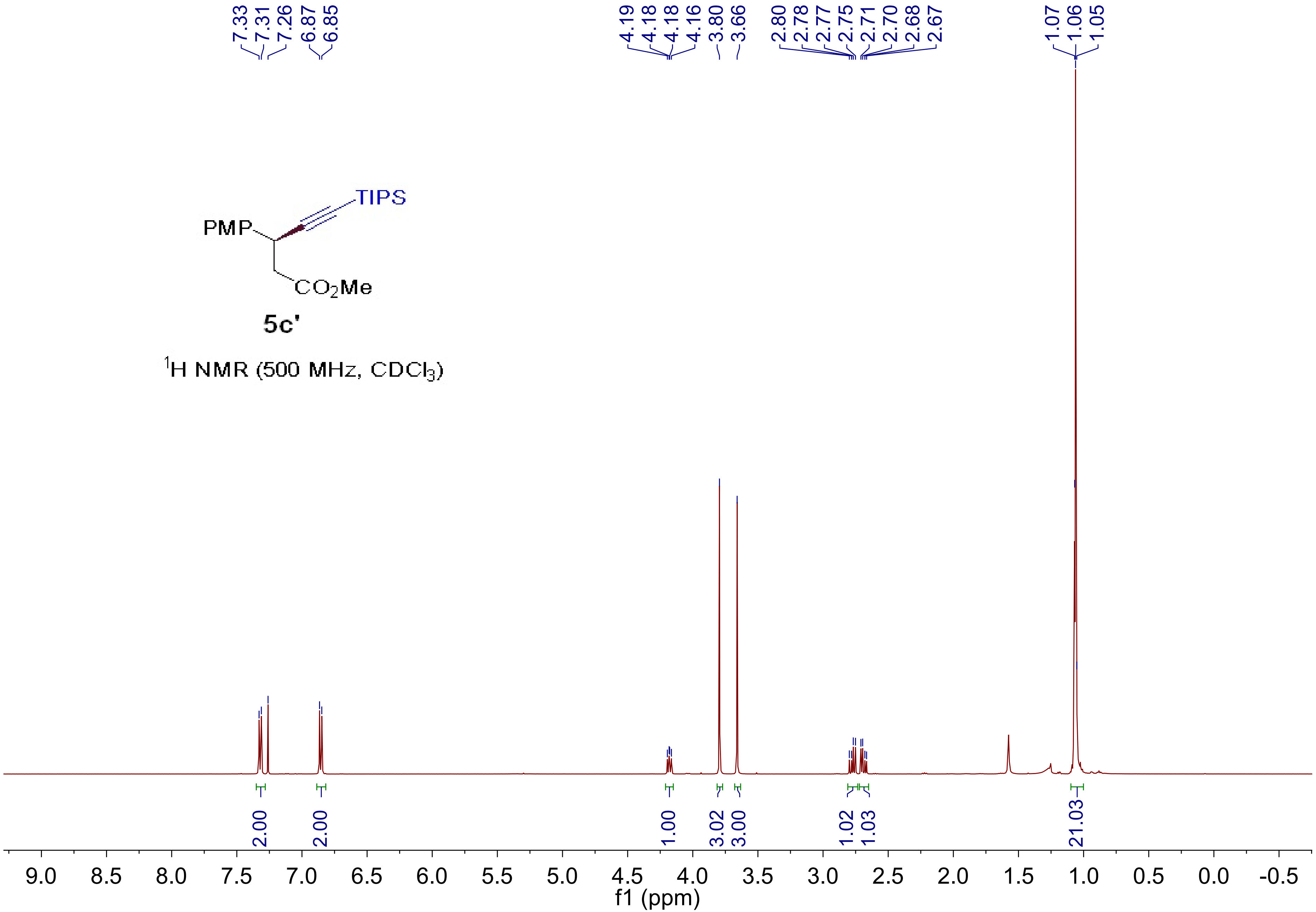

Supplementary Fig. 164. ^{1}H NMR (500 MHz, $CDCl_3$) spectra for compound **5c'**

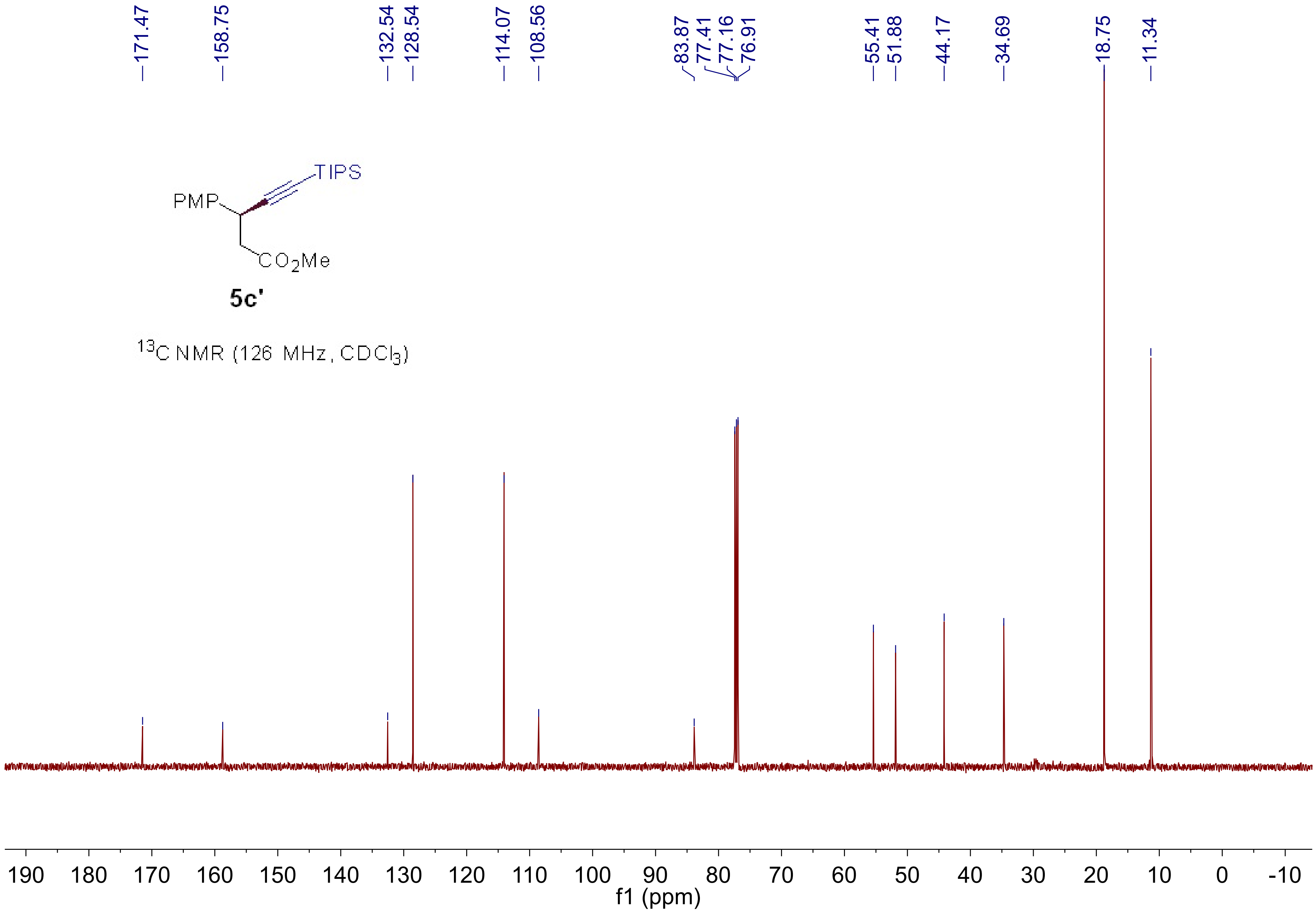

Supplementary Fig. 165. ^{13}C NMR (126 MHz, $CDCl_3$) spectra for compound **5c'**

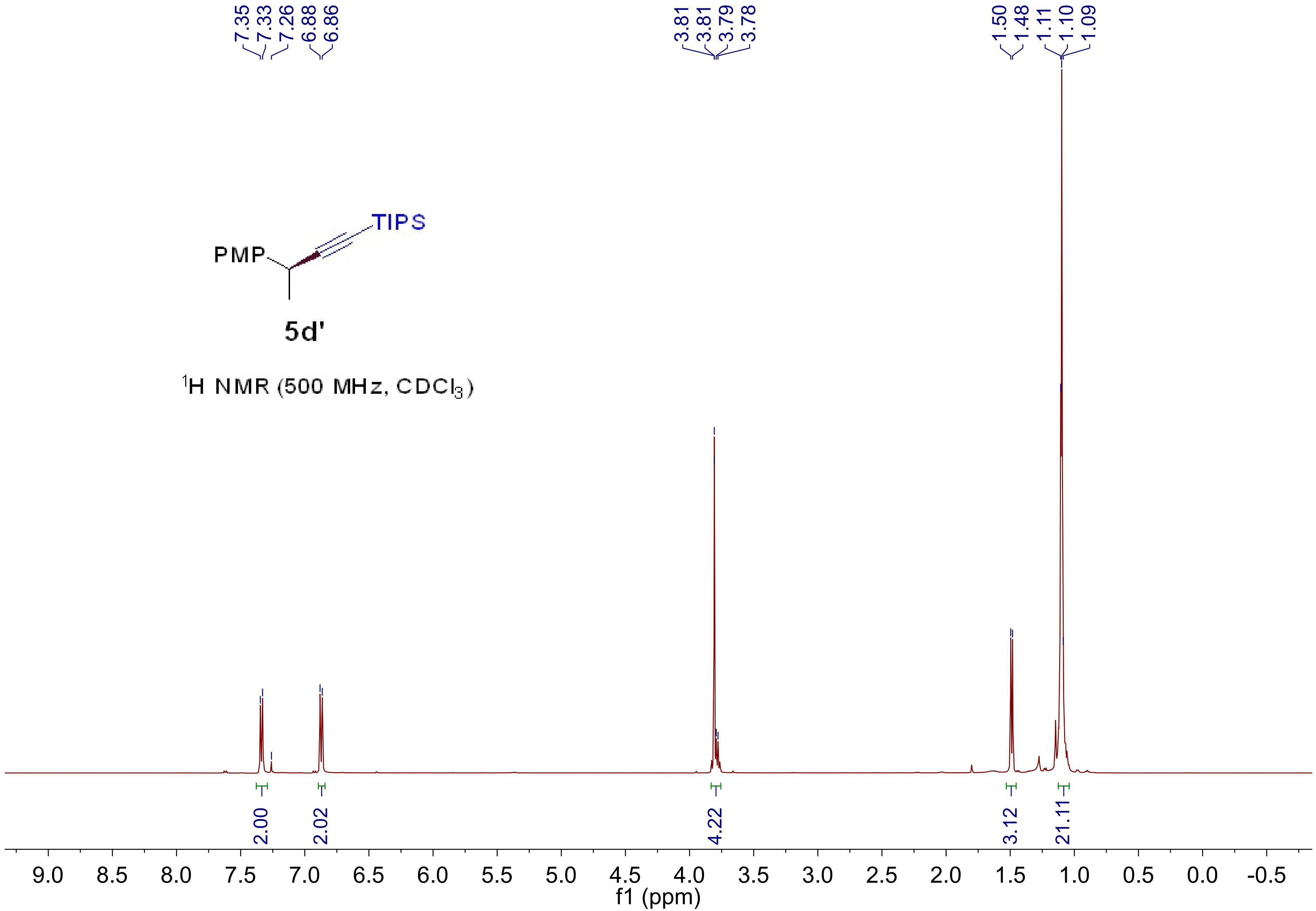

Supplementary Fig. 166. ^{1}H NMR (500 MHz, $CDCl_3$) spectra for compound **5d'**

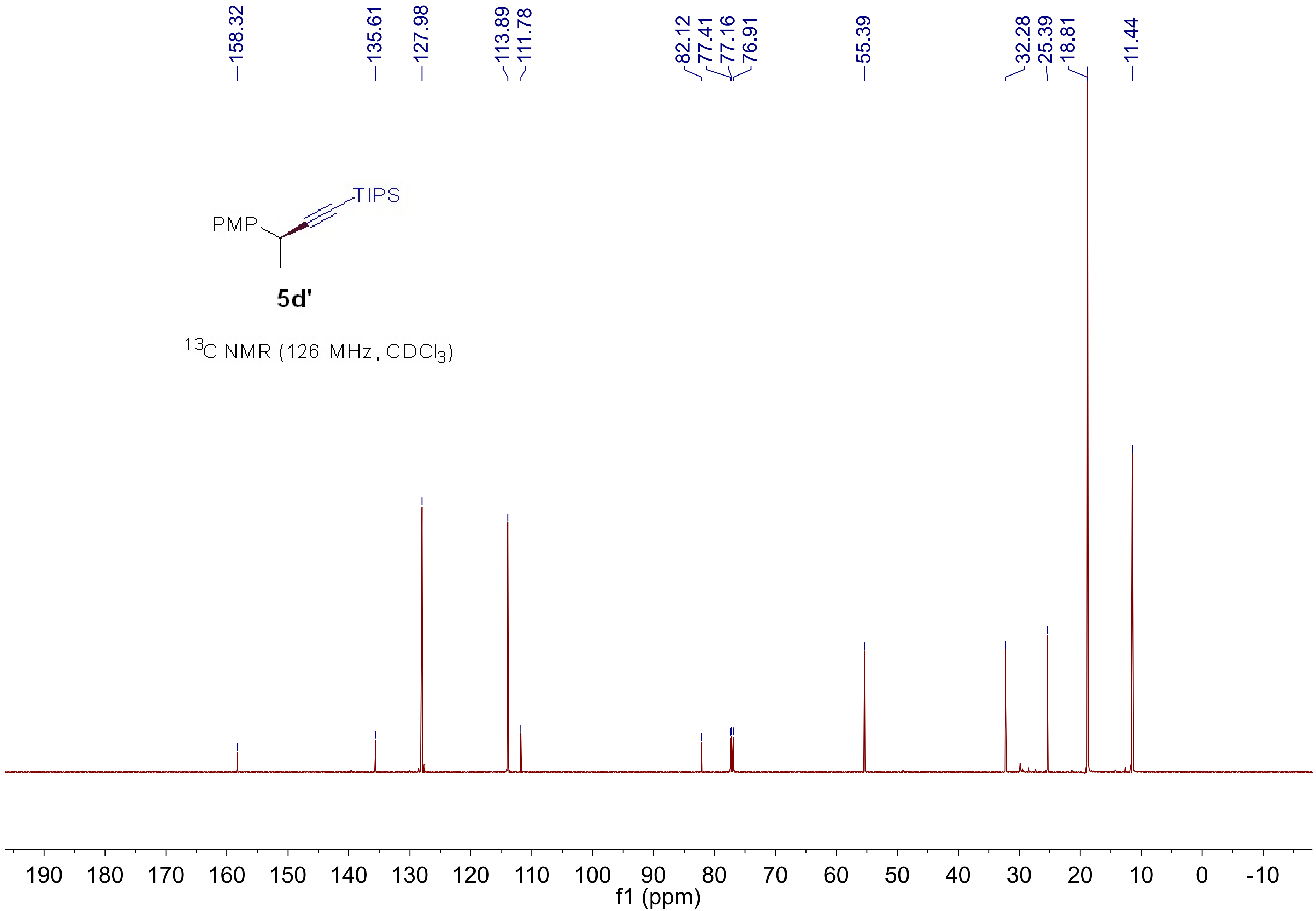

Supplementary Fig. 167. ^{13}C NMR (126 MHz, $CDCl_3$) spectra for compound **5d'**

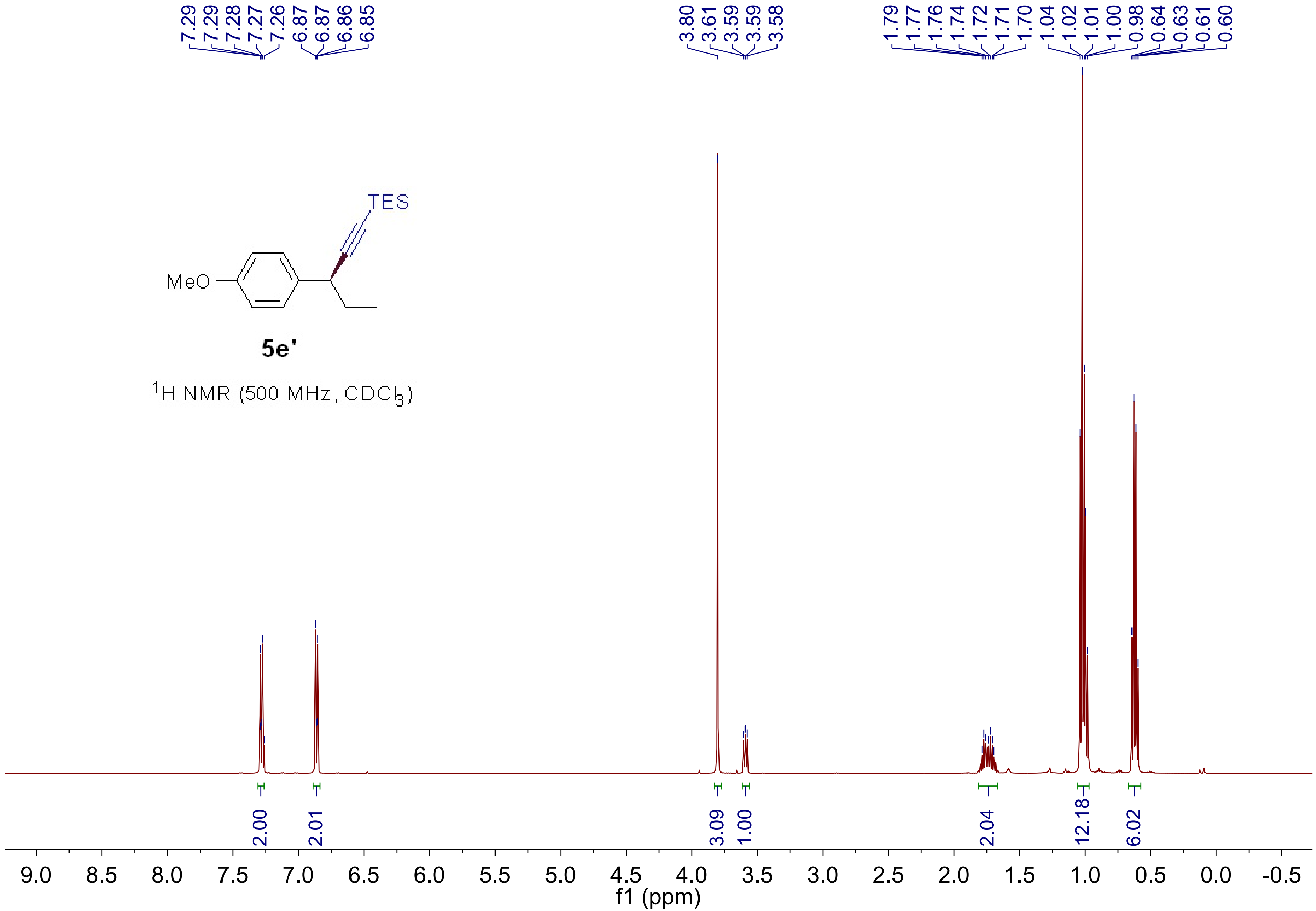

Supplementary Fig. 168. ^{1}H NMR (500 MHz, $CDCl_3$) spectra for compound **5e'**

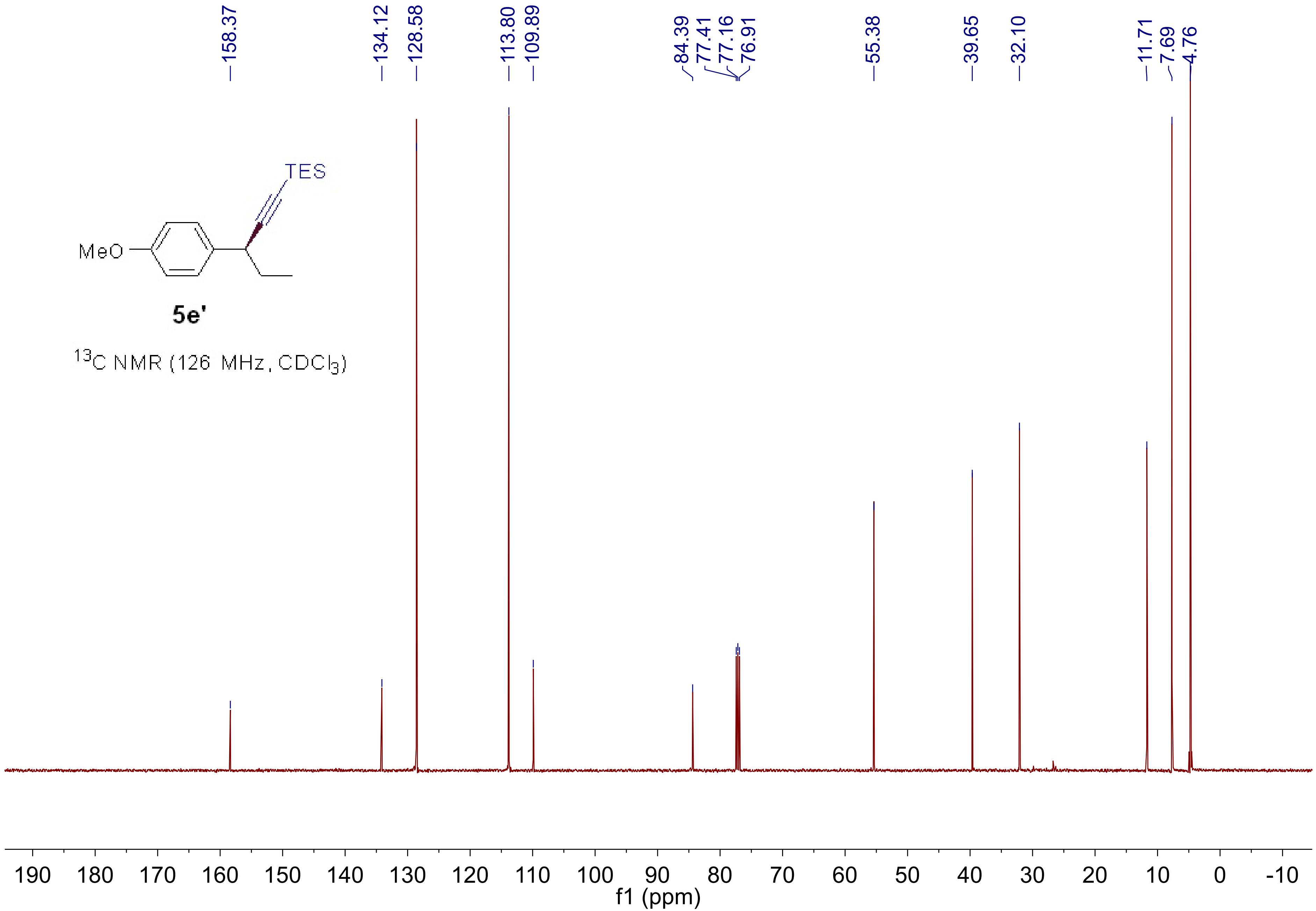

Supplementary Fig. 169. ^{13}C NMR (126 MHz, $CDCl_3$) spectra for compound **5e'**

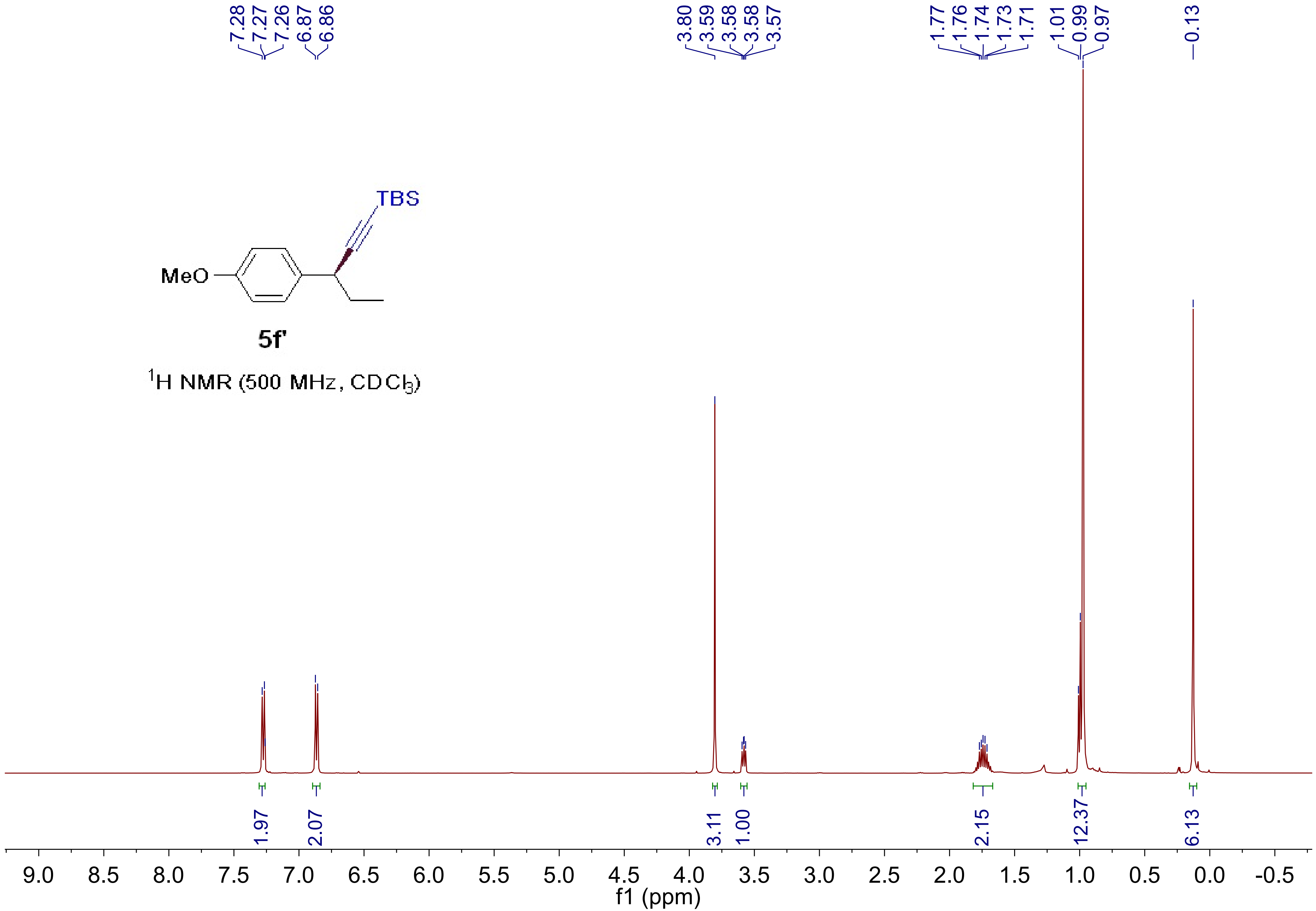

Supplementary Fig. 170. ^{1}H NMR (500 MHz, $CDCl_3$) spectra for compound **5f'**

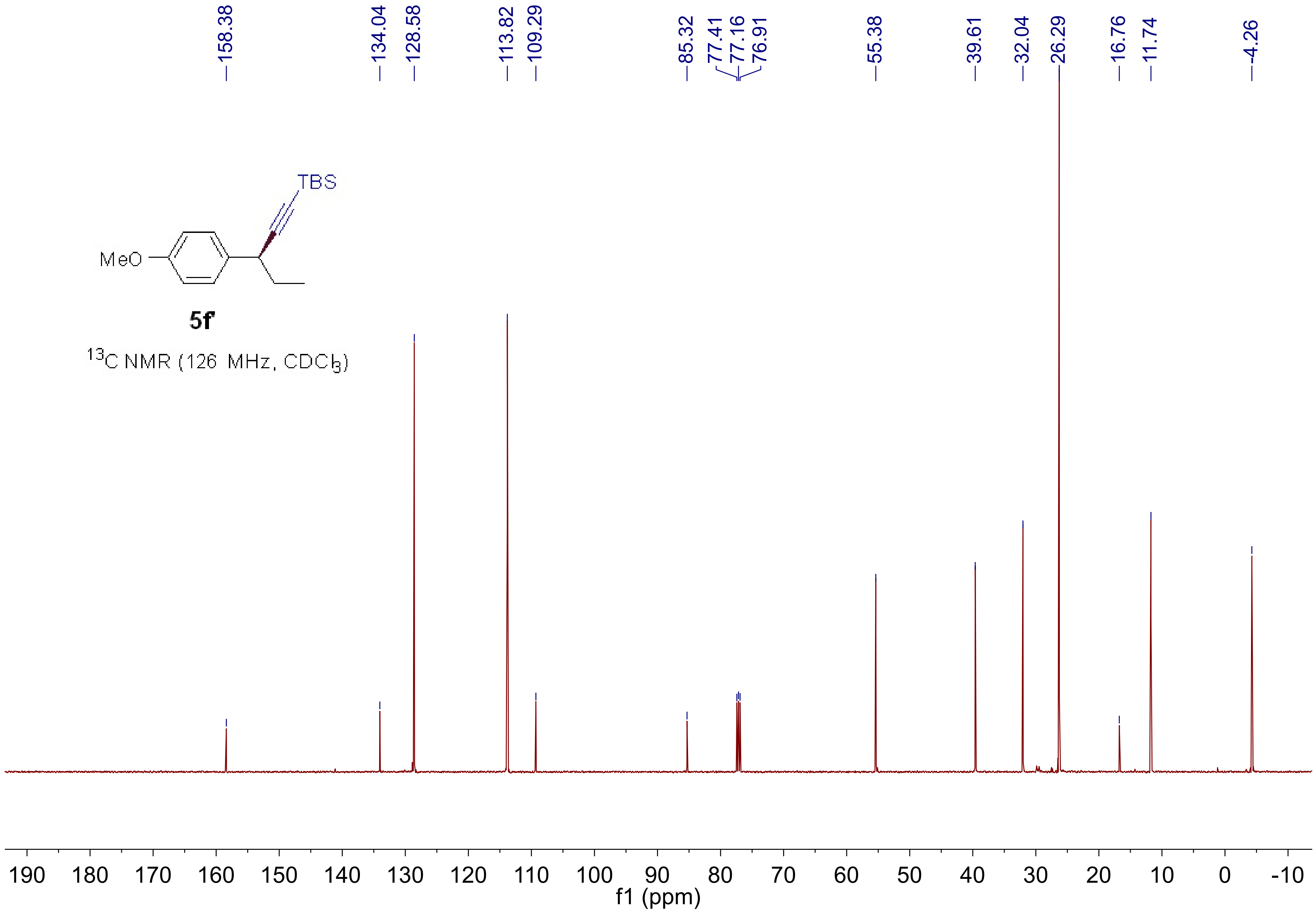

Supplementary Fig. 171. ^{13}C NMR (126 MHz, $CDCl_3$) spectra for compound **5f'**

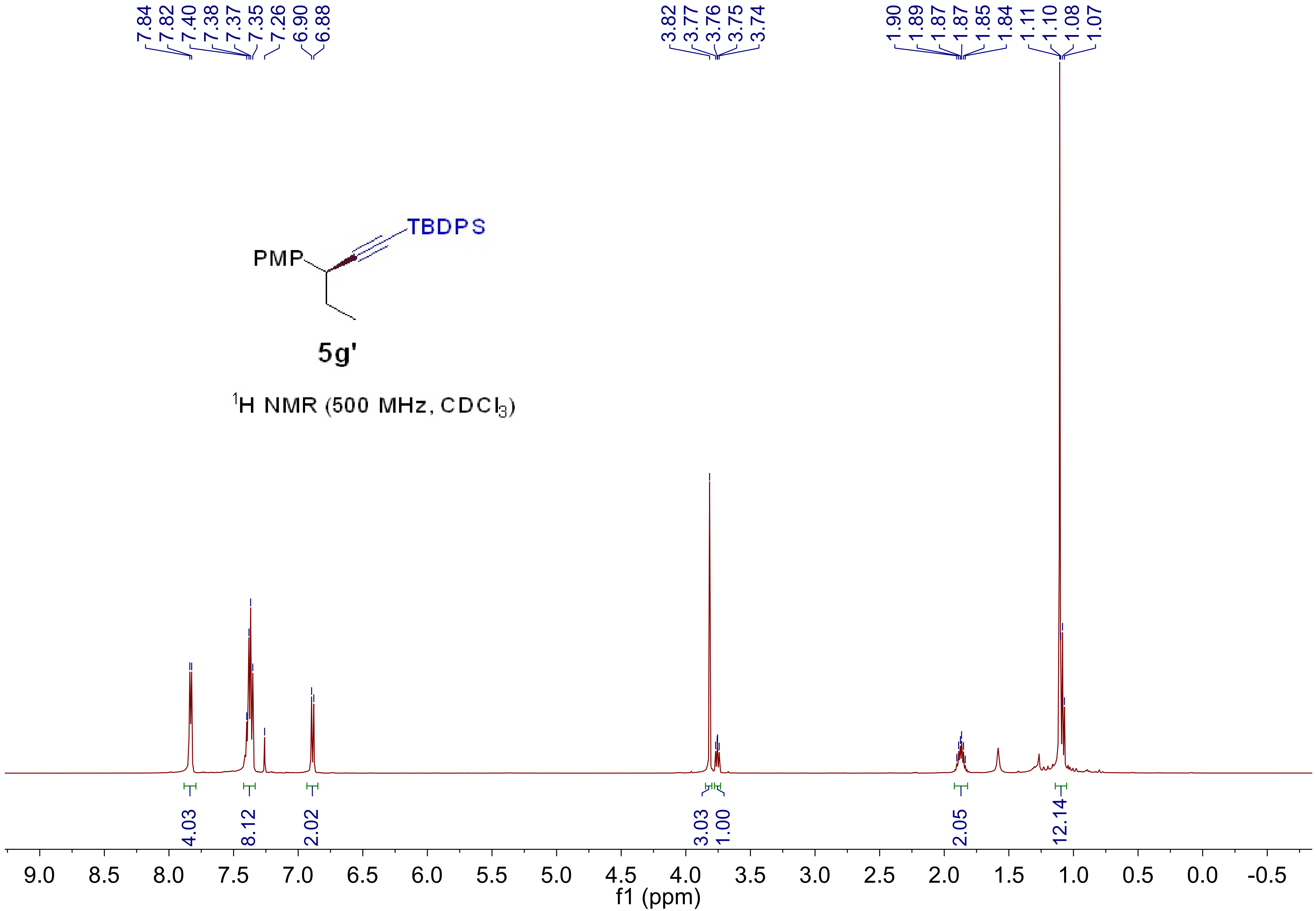

Supplementary Fig. 172. ^{1}H NMR (500 MHz, $CDCl_3$) spectra for compound **5g'**

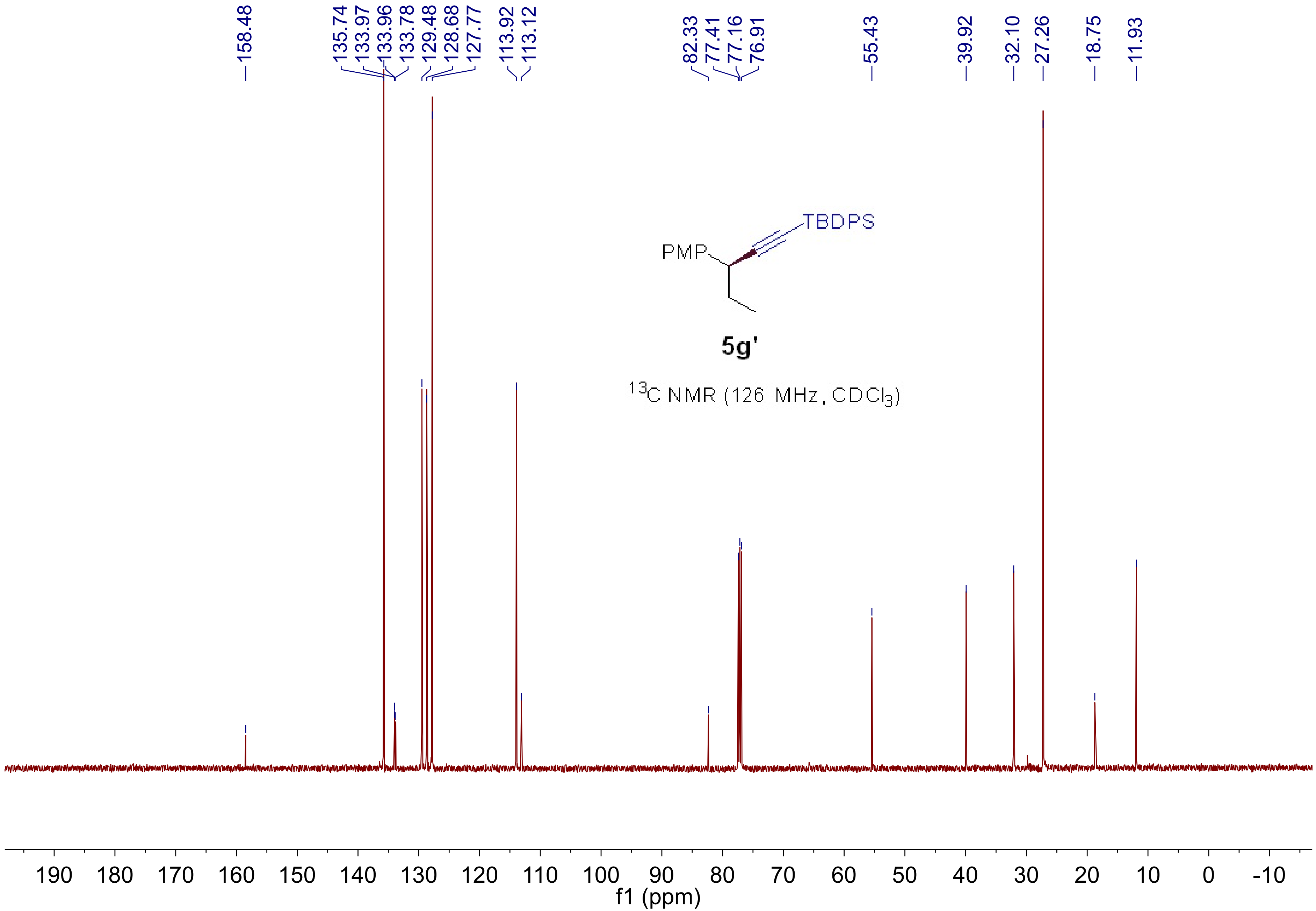

Supplementary Fig. 173. ^{13}C NMR (126 MHz, $CDCl_3$) spectra for compound **5g'**

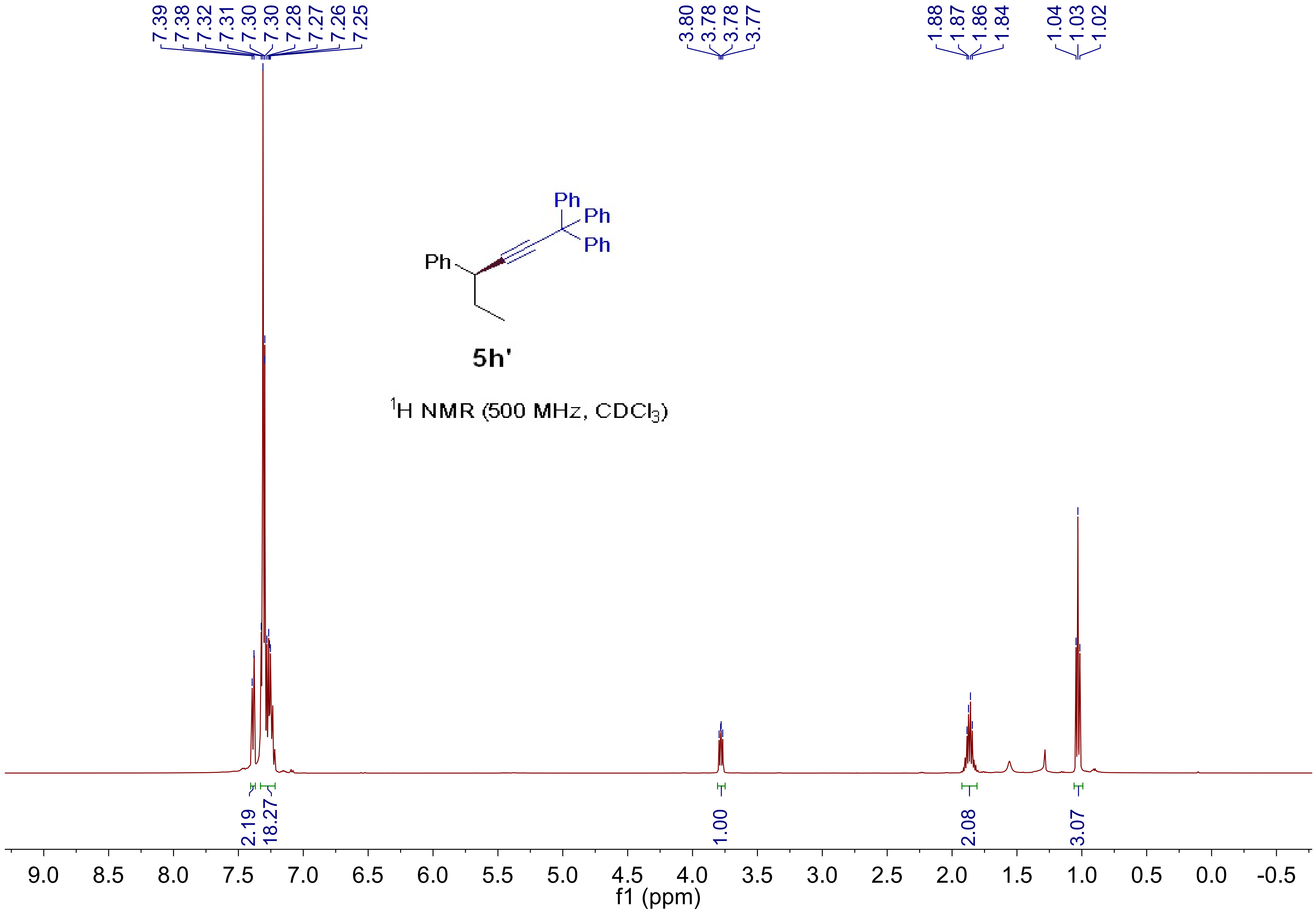

Supplementary Fig. 174. ^{1}H NMR (500 MHz, $CDCl_3$) spectra for compound **5h'**

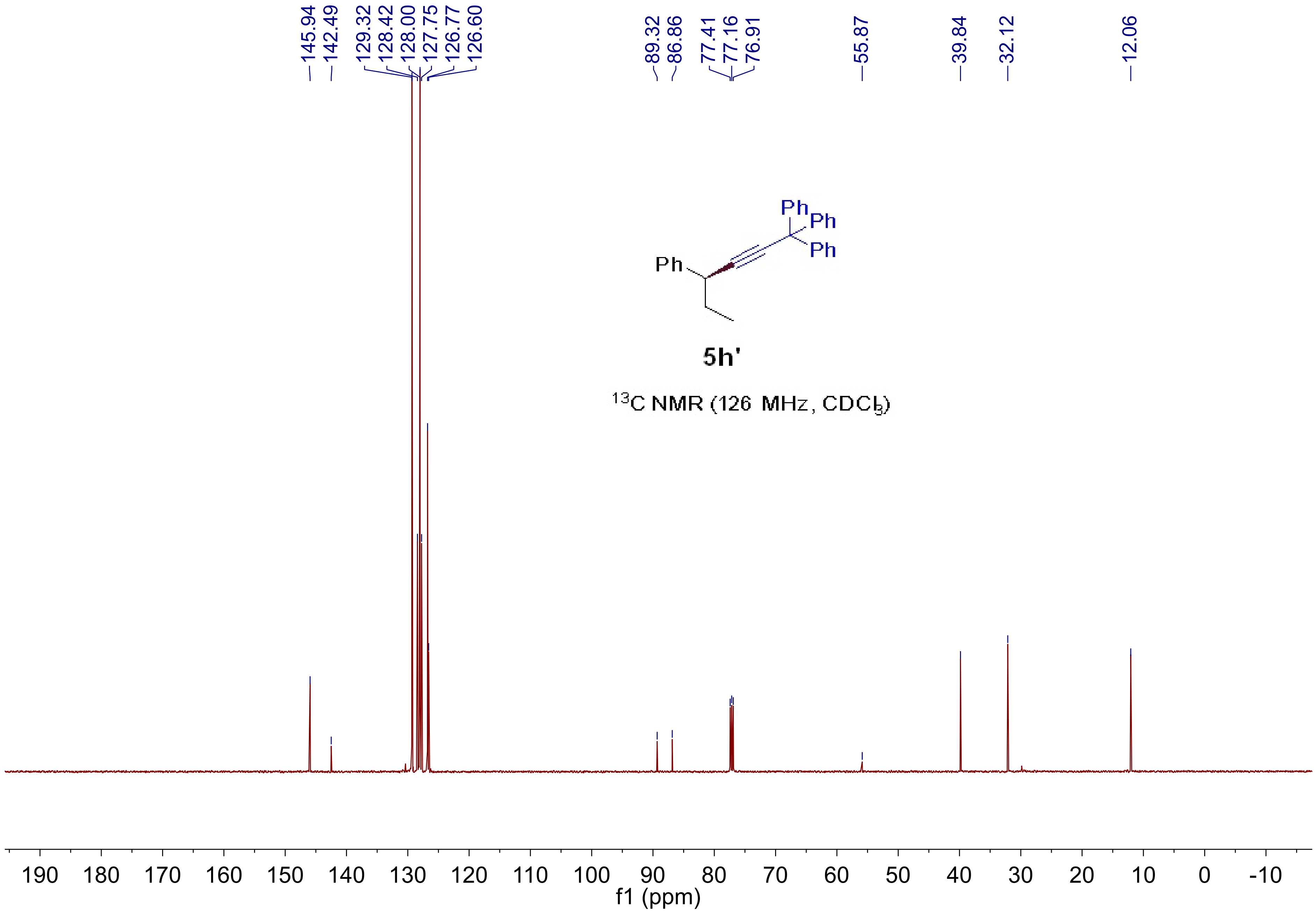

Supplementary Fig. 175. ^{13}C NMR (126 MHz, $CDCl_3$) spectra for compound **5h'**

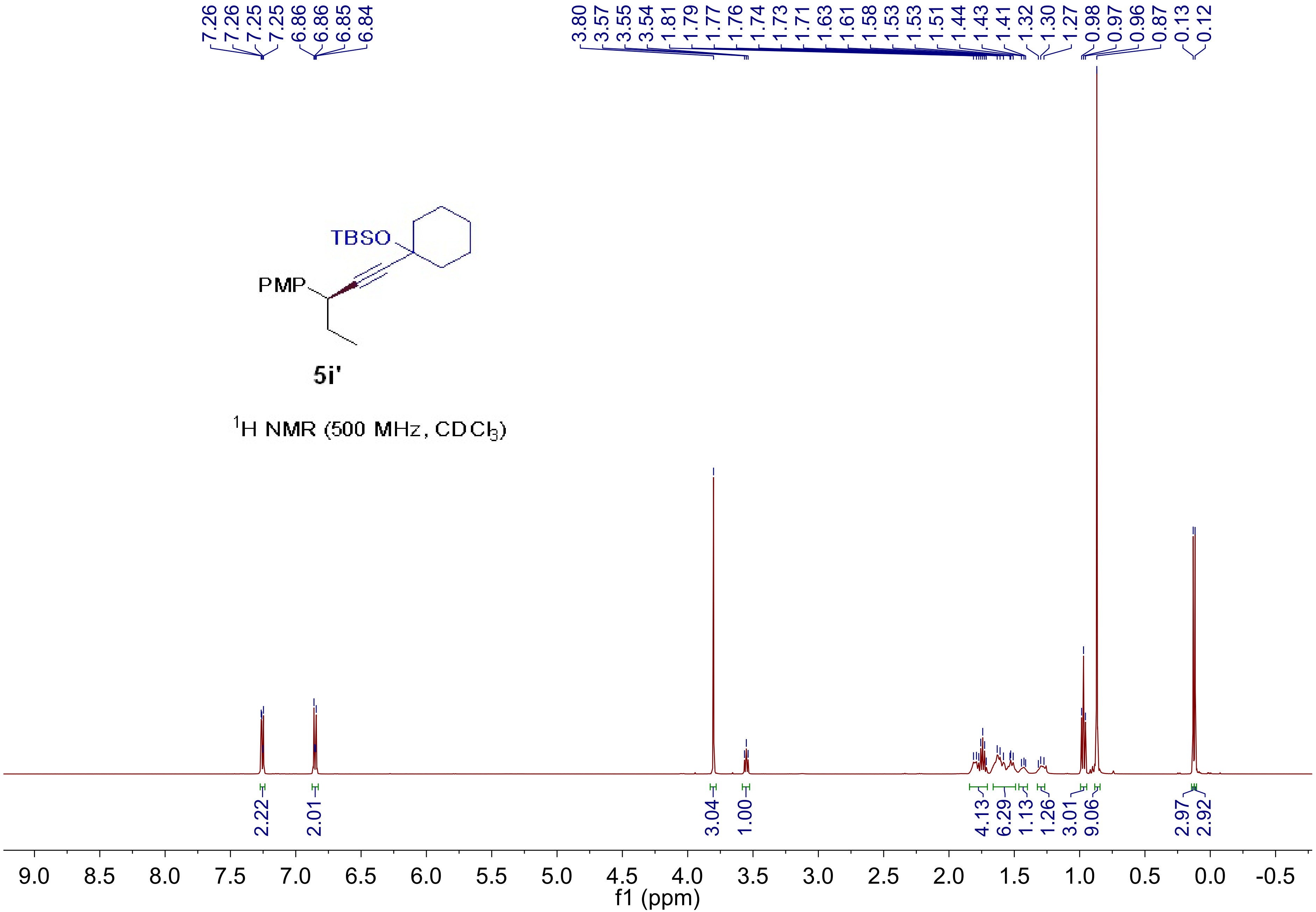

Supplementary Fig. 176. ^{1}H NMR (500 MHz, CDCl$_3$) spectra for compound **5i'**

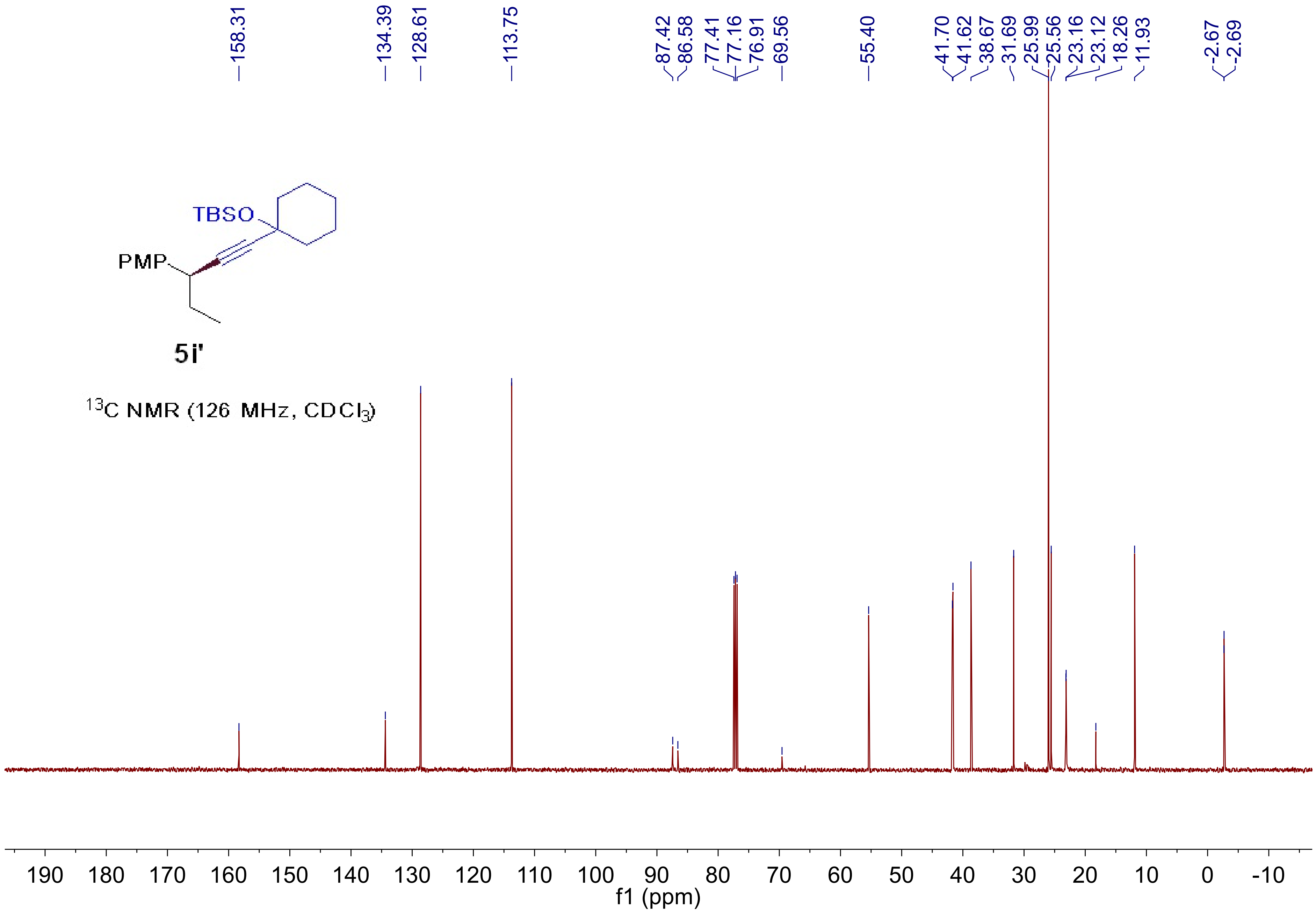

Supplementary Fig. 177. ^{13}C NMR (126 MHz, $CDCl_3$) spectra for compound **5i'**

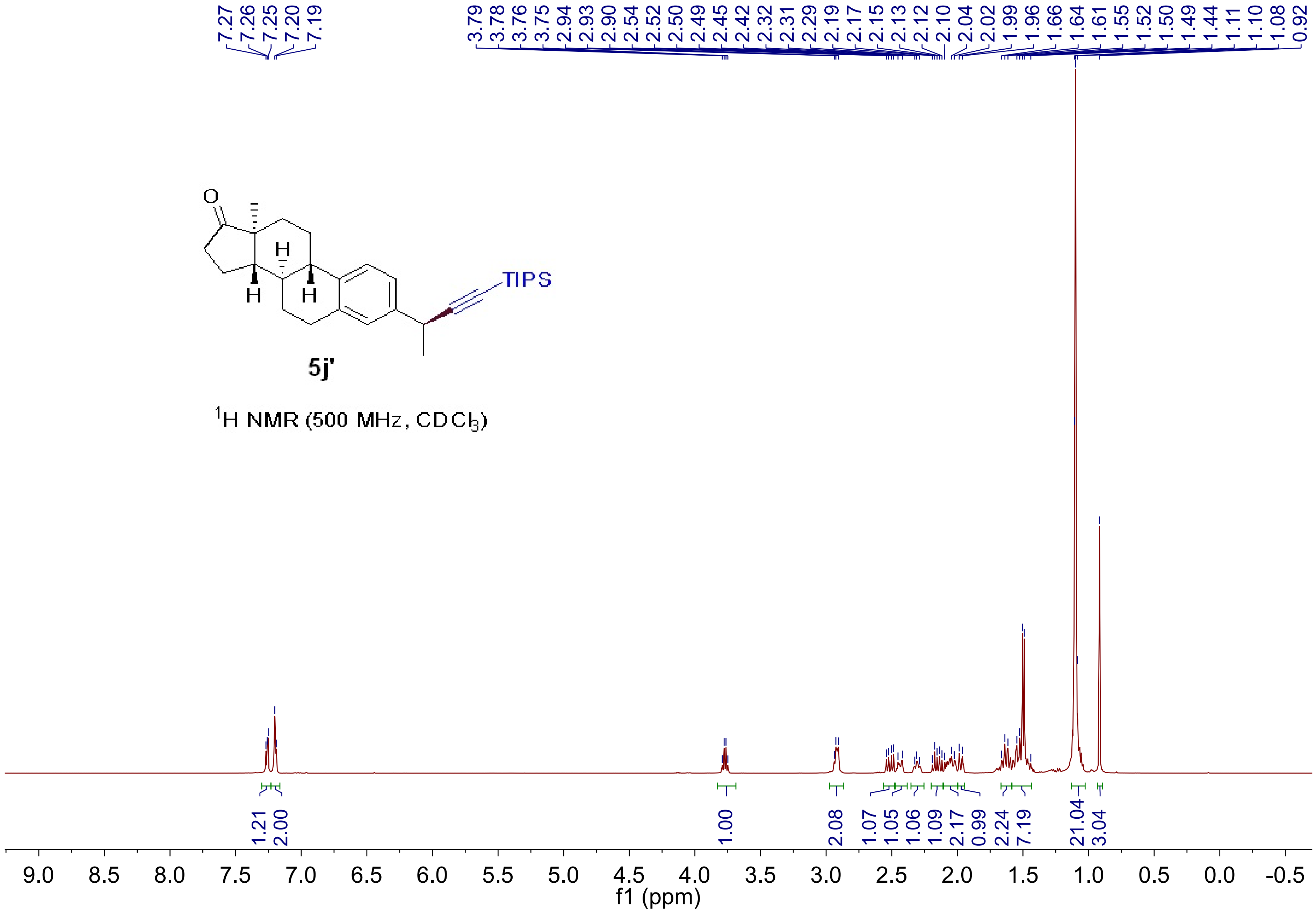

Supplementary Fig. 178. ^{1}H NMR (500 MHz, $CDCl_3$) spectra for compound **5j'**

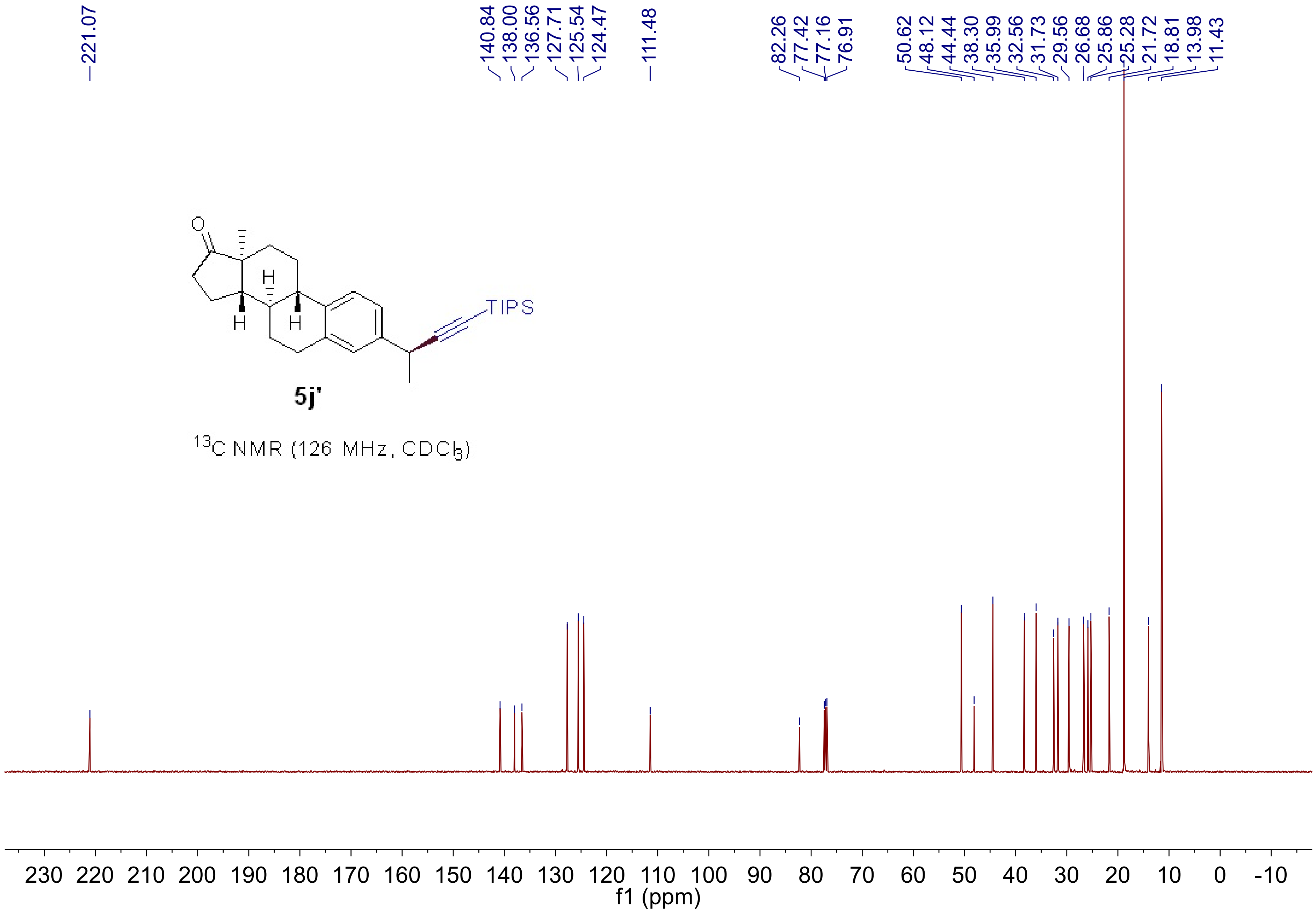

Supplementary Fig. 179. ^{13}C NMR (126 MHz, $CDCl_3$) spectra for compound **5j'**

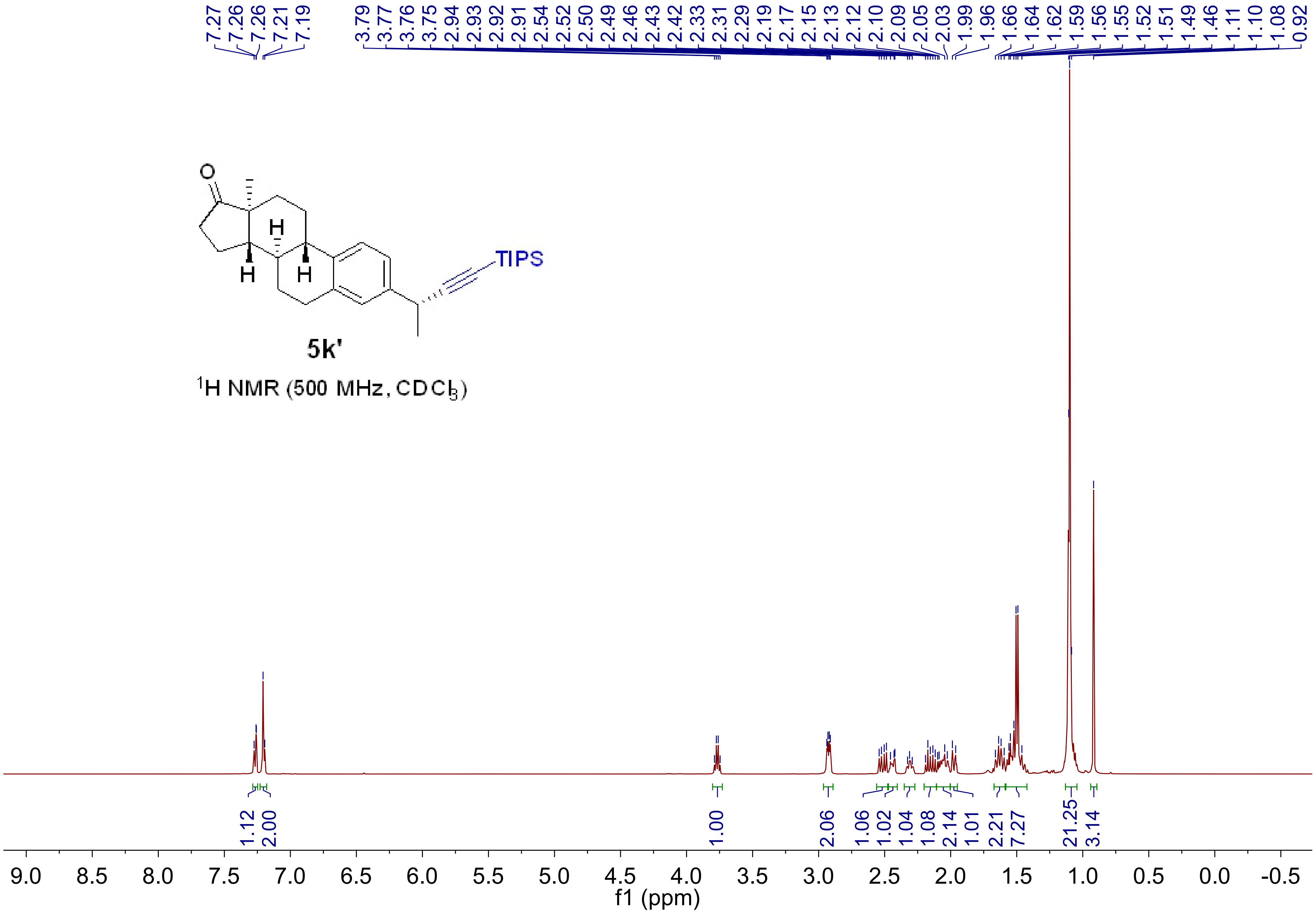

Supplementary Fig. 180. ^{1}H NMR (500 MHz, $CDCl_3$) spectra for compound **5k'**

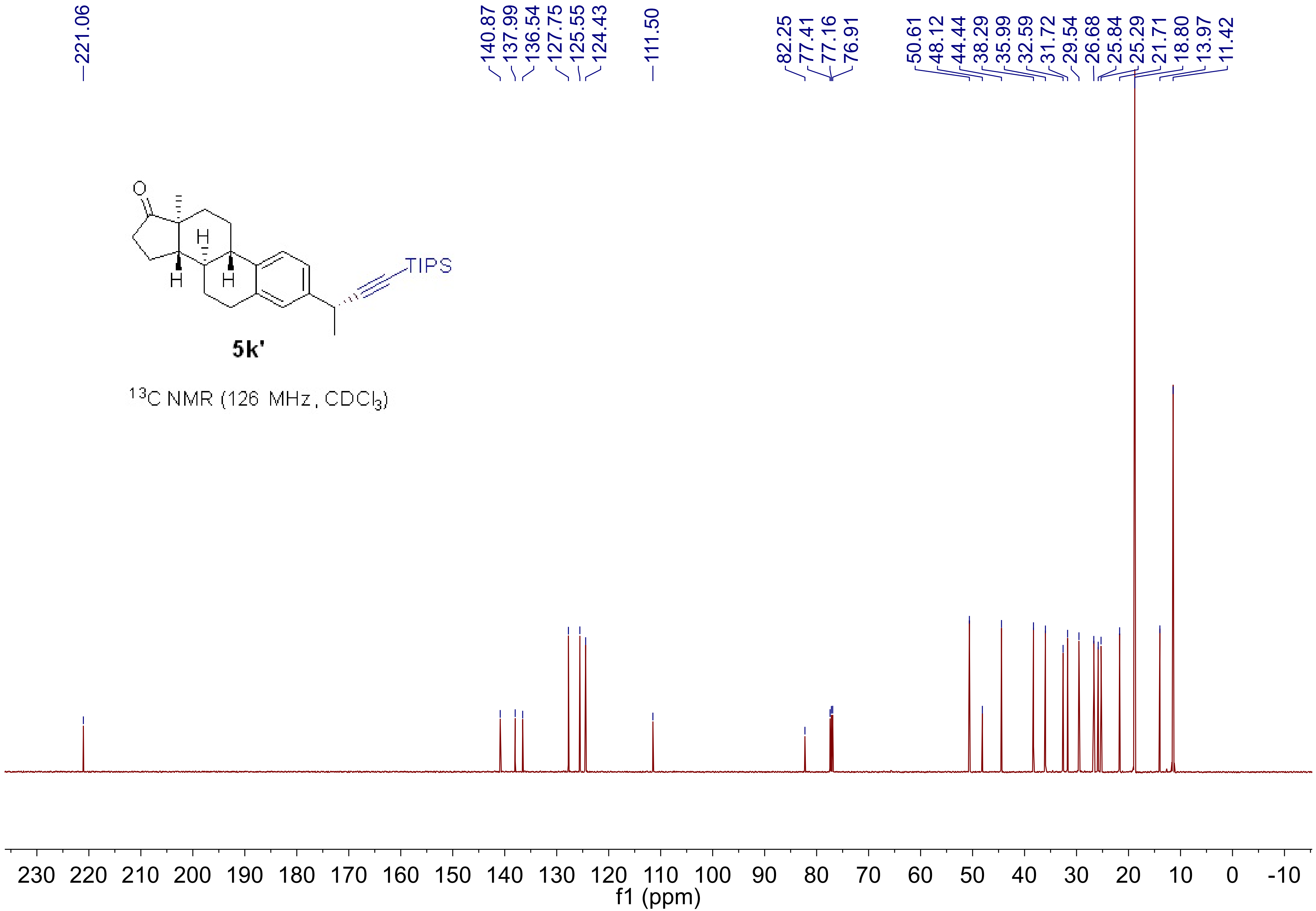

Supplementary Fig. 181. ^{13}C NMR (126 MHz, $CDCl_3$) spectra for compound **5k'**

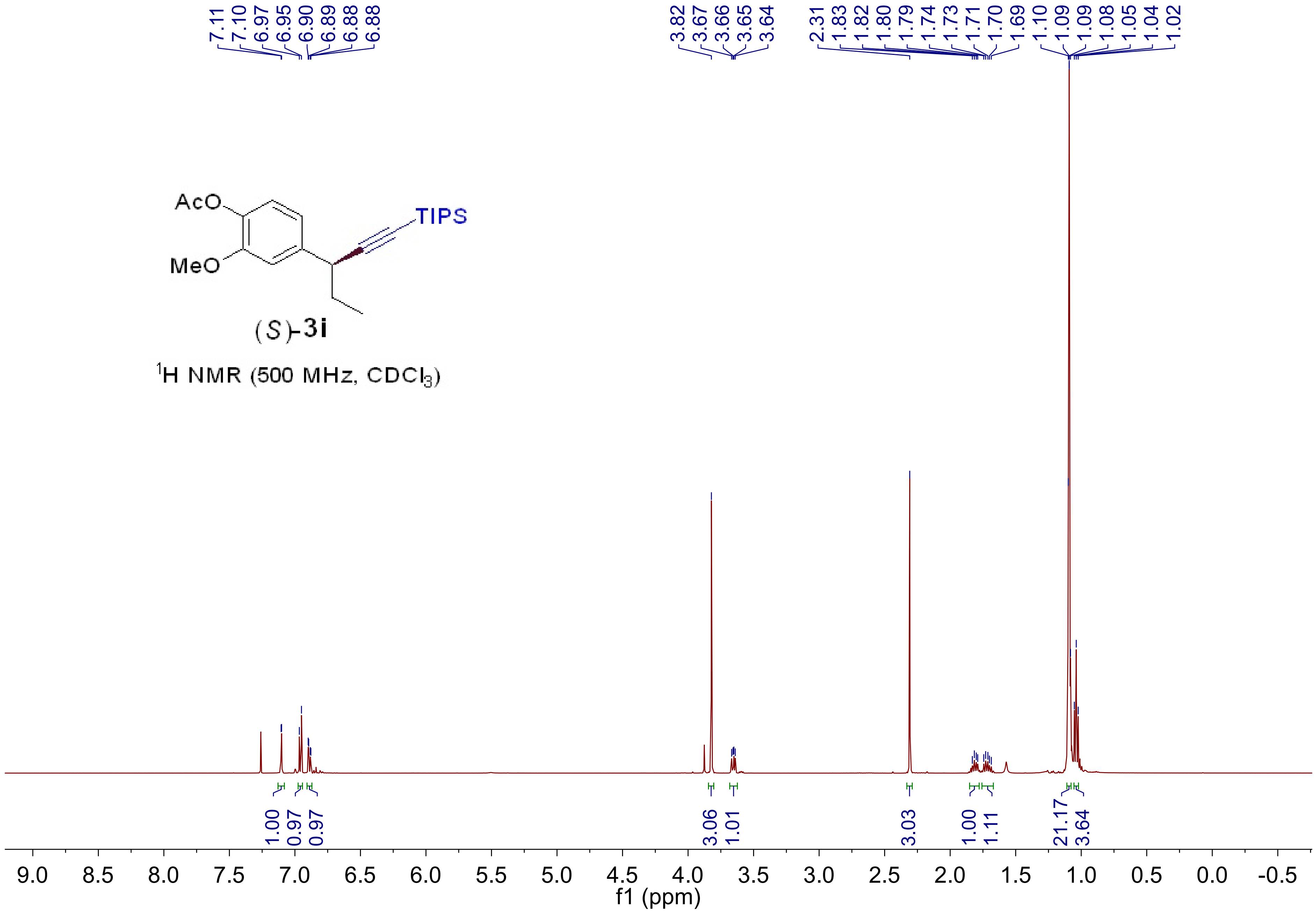

Supplementary Fig. 182. ^{1}H NMR (500 MHz, $CDCl_3$) spectra for compound (*S*)-**3i**

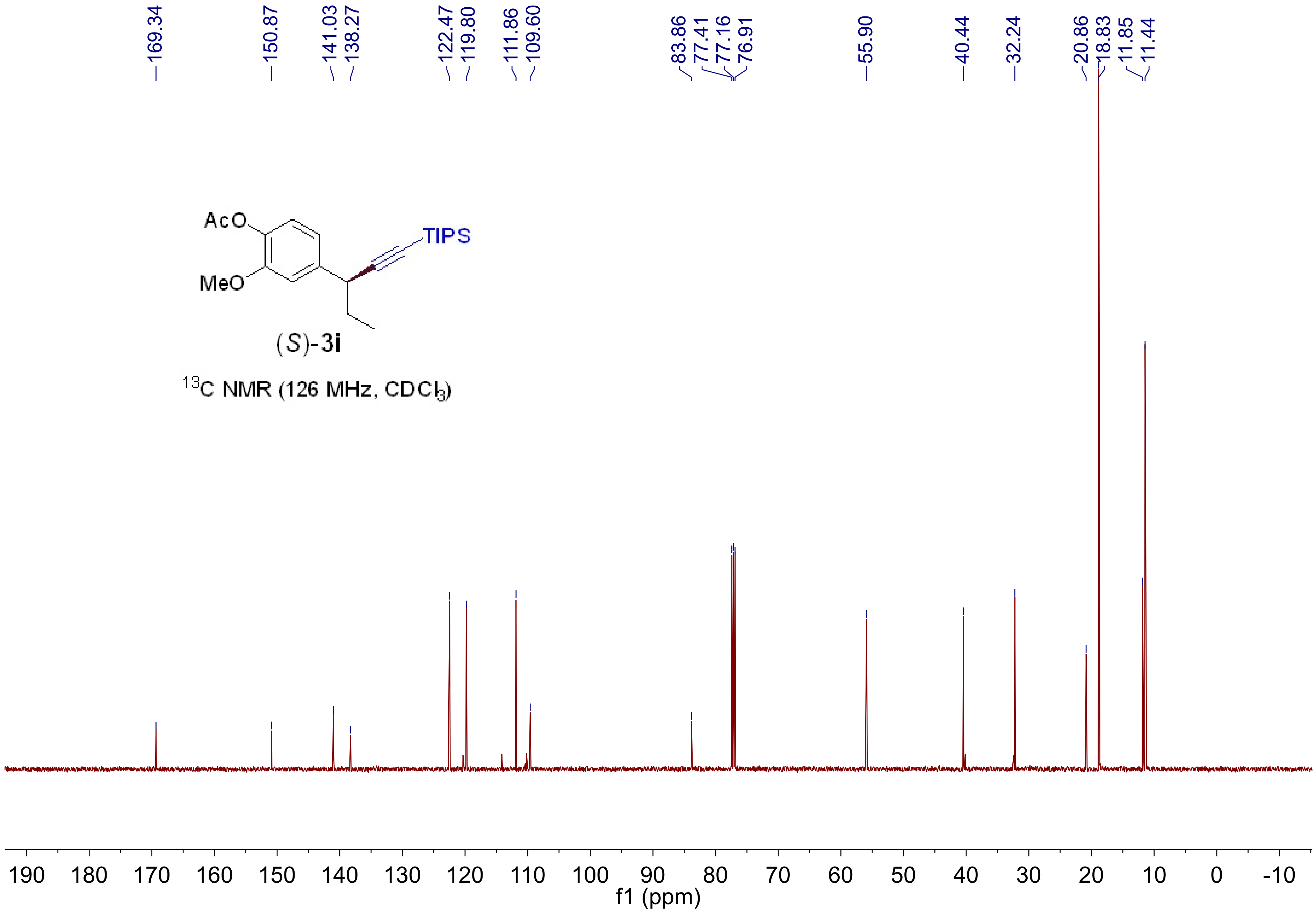

Supplementary Fig. 183. ^{13}C NMR (126 MHz, $CDCl_3$) spectra for compound (*S*)-**3i**

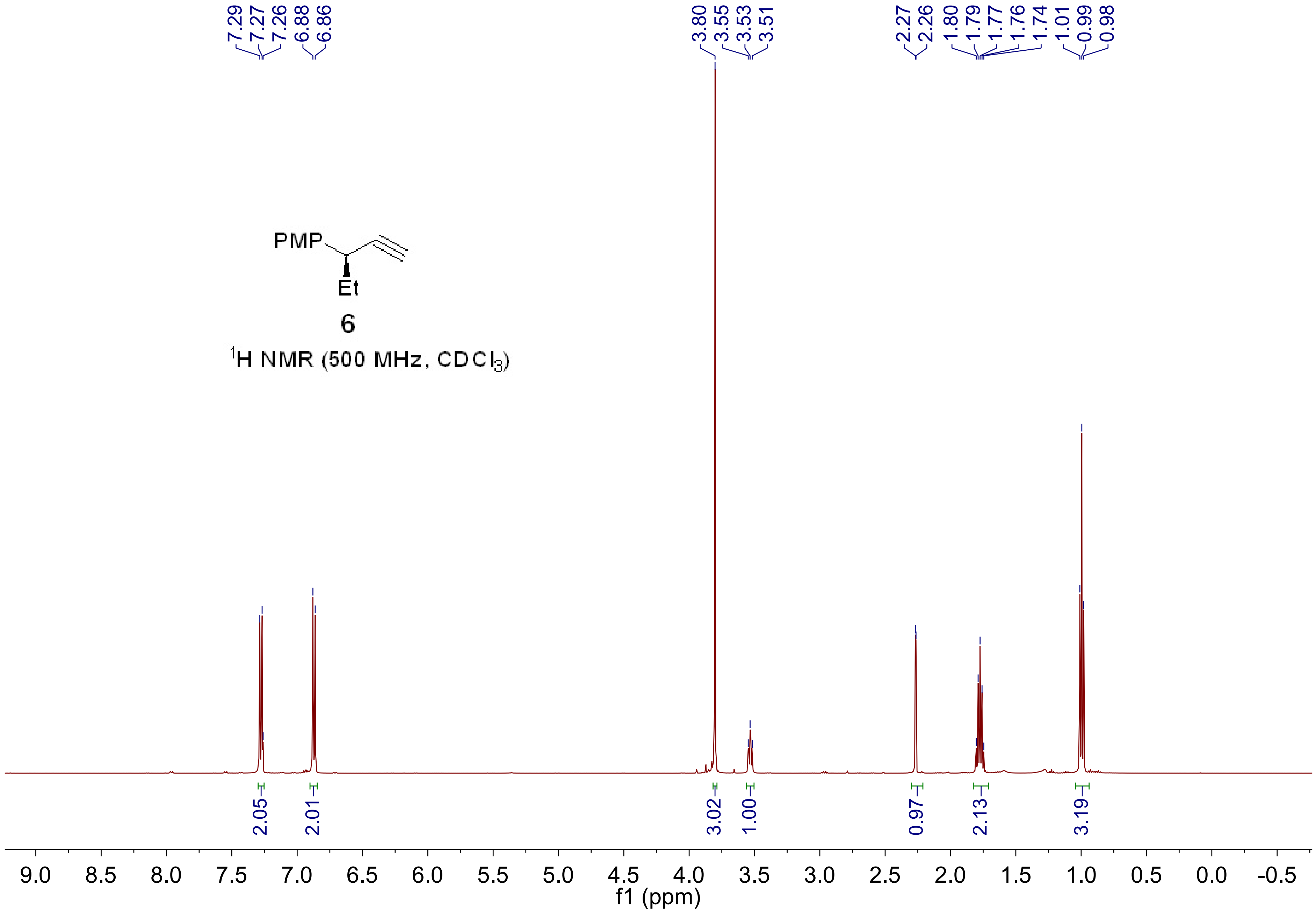

Supplementary Fig. 184. ^{1}H NMR (500 MHz, $CDCl_3$) spectra for compound **6**

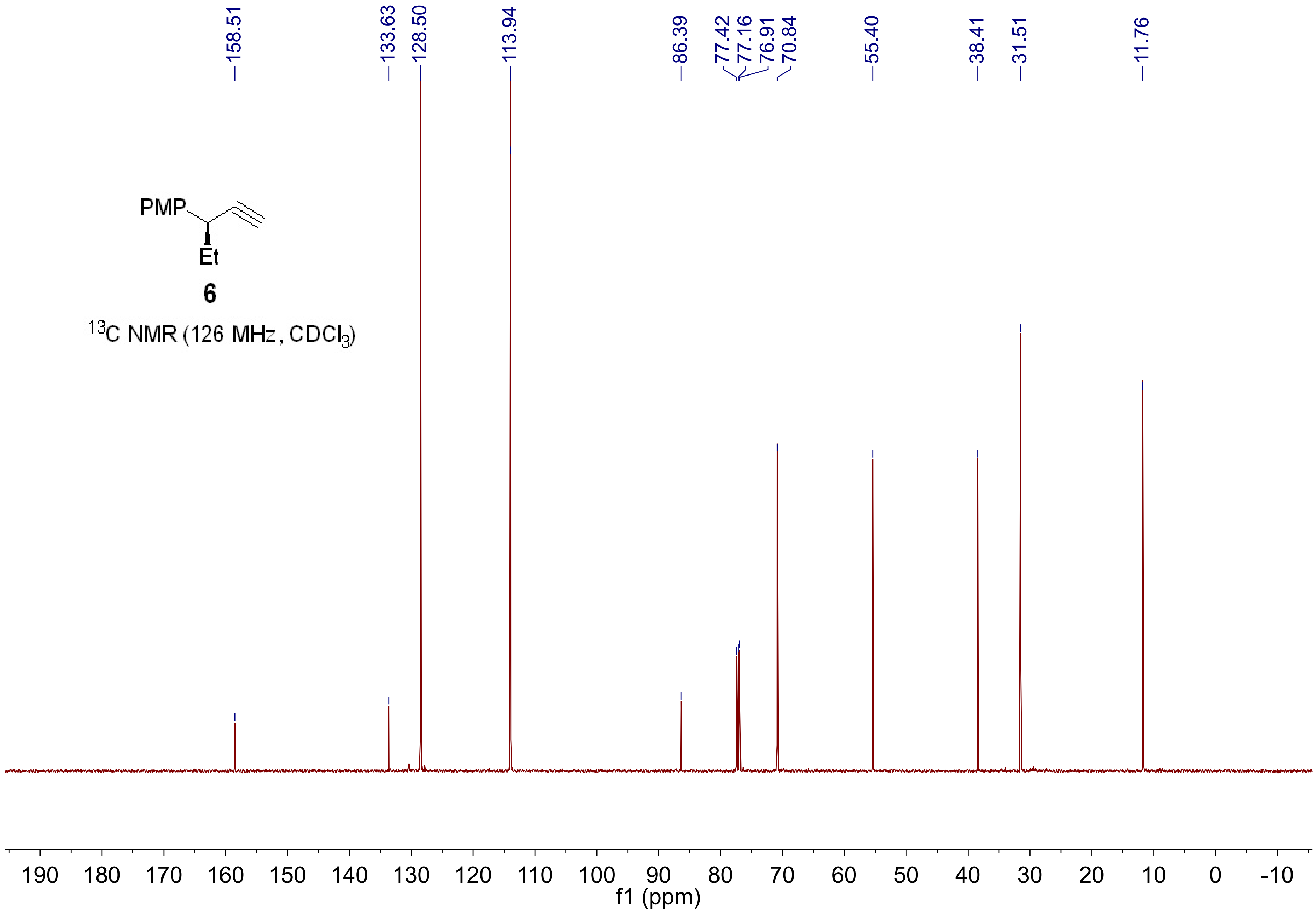

Supplementary Fig. 185. ^{13}C NMR (126 MHz, $CDCl_3$) spectra for compound **6**

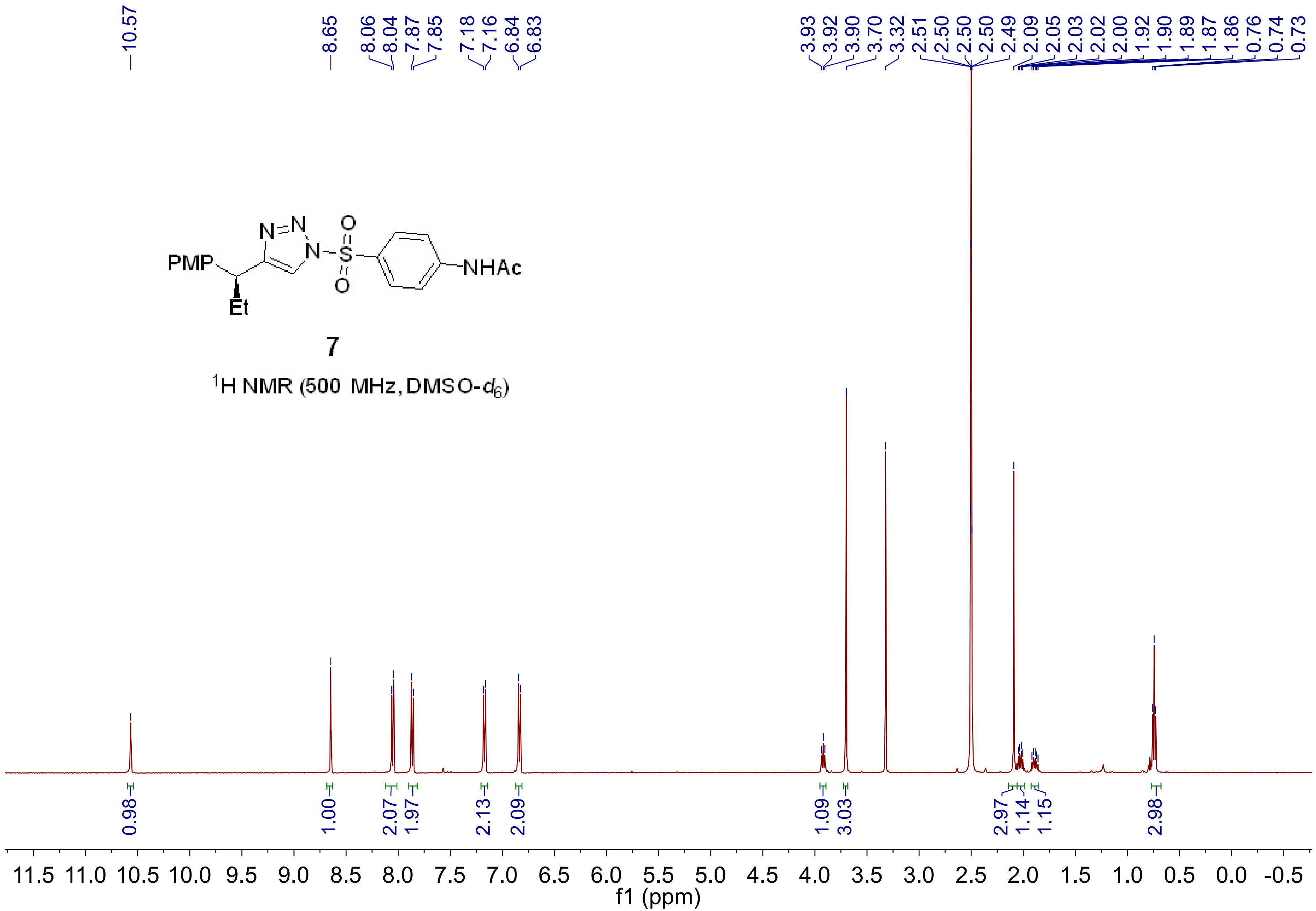

Supplementary Fig. 186. ^{1}H NMR (500 MHz, DMSO-d_6) spectra for compound **7**

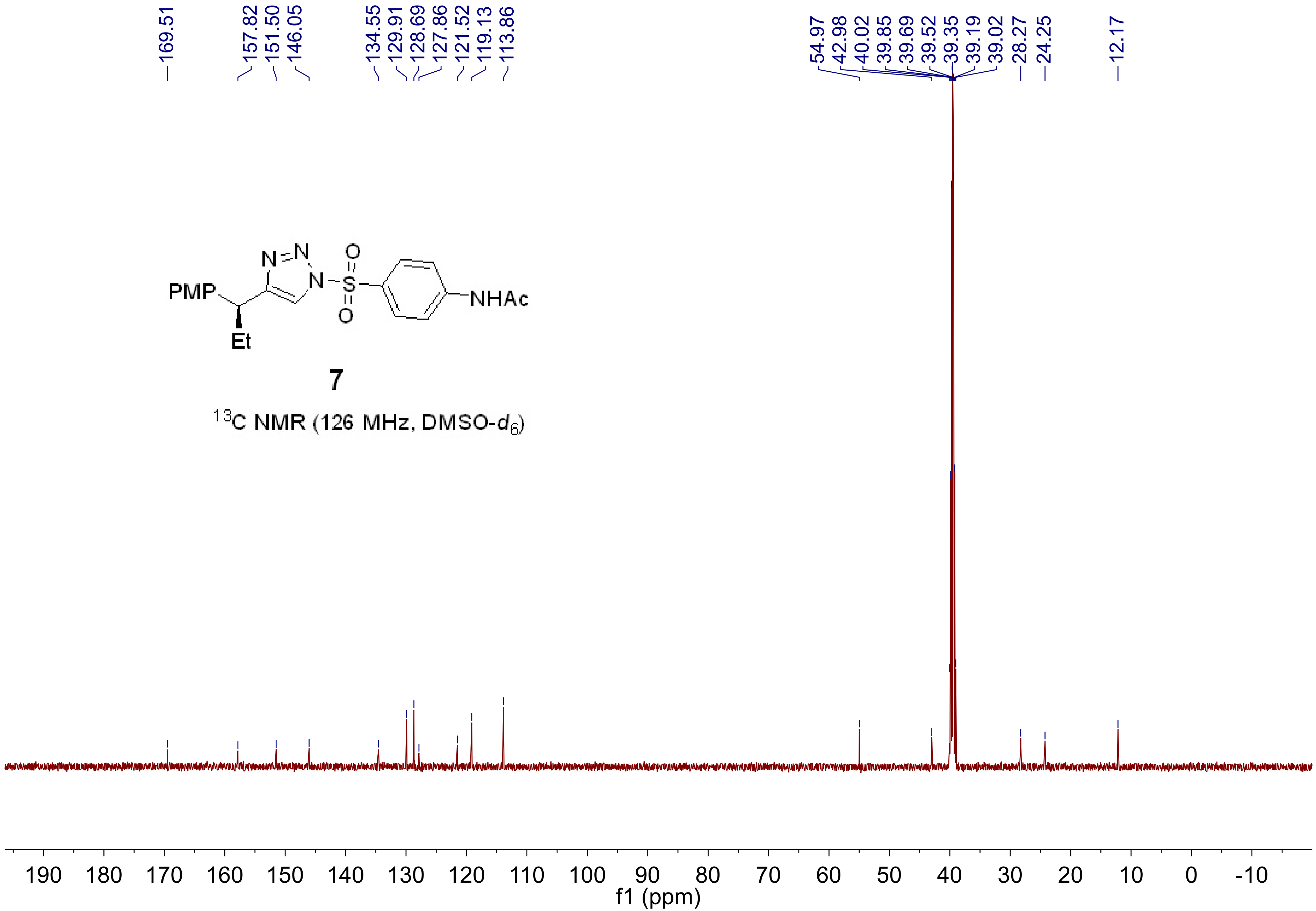

Supplementary Fig. 187. ^{13}C NMR (126 MHz, DMSO-d_6) spectra for compound **7**

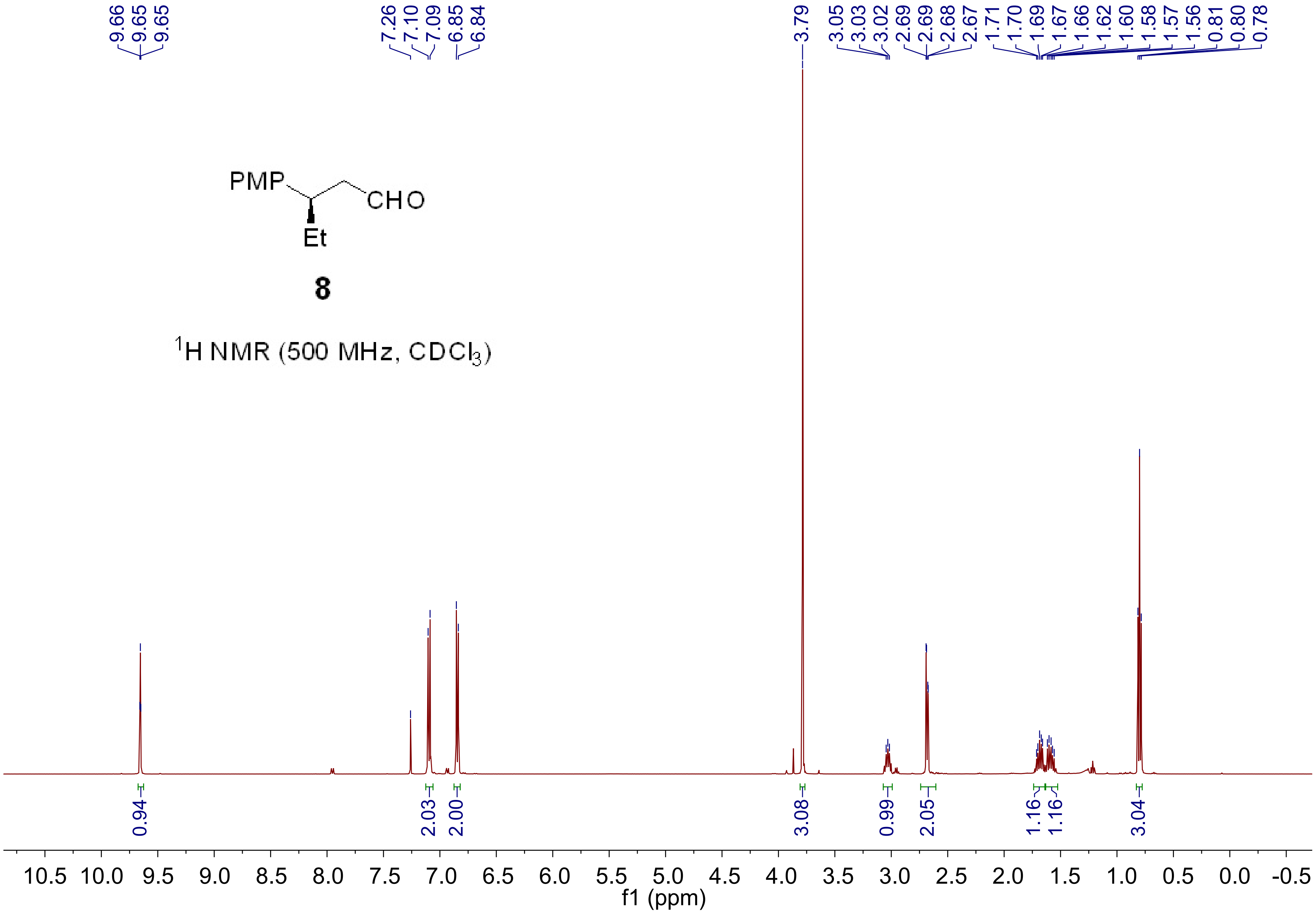

Supplementary Fig. 188. ^{1}H NMR (500 MHz, $CDCl_3$) spectra for compound **8**

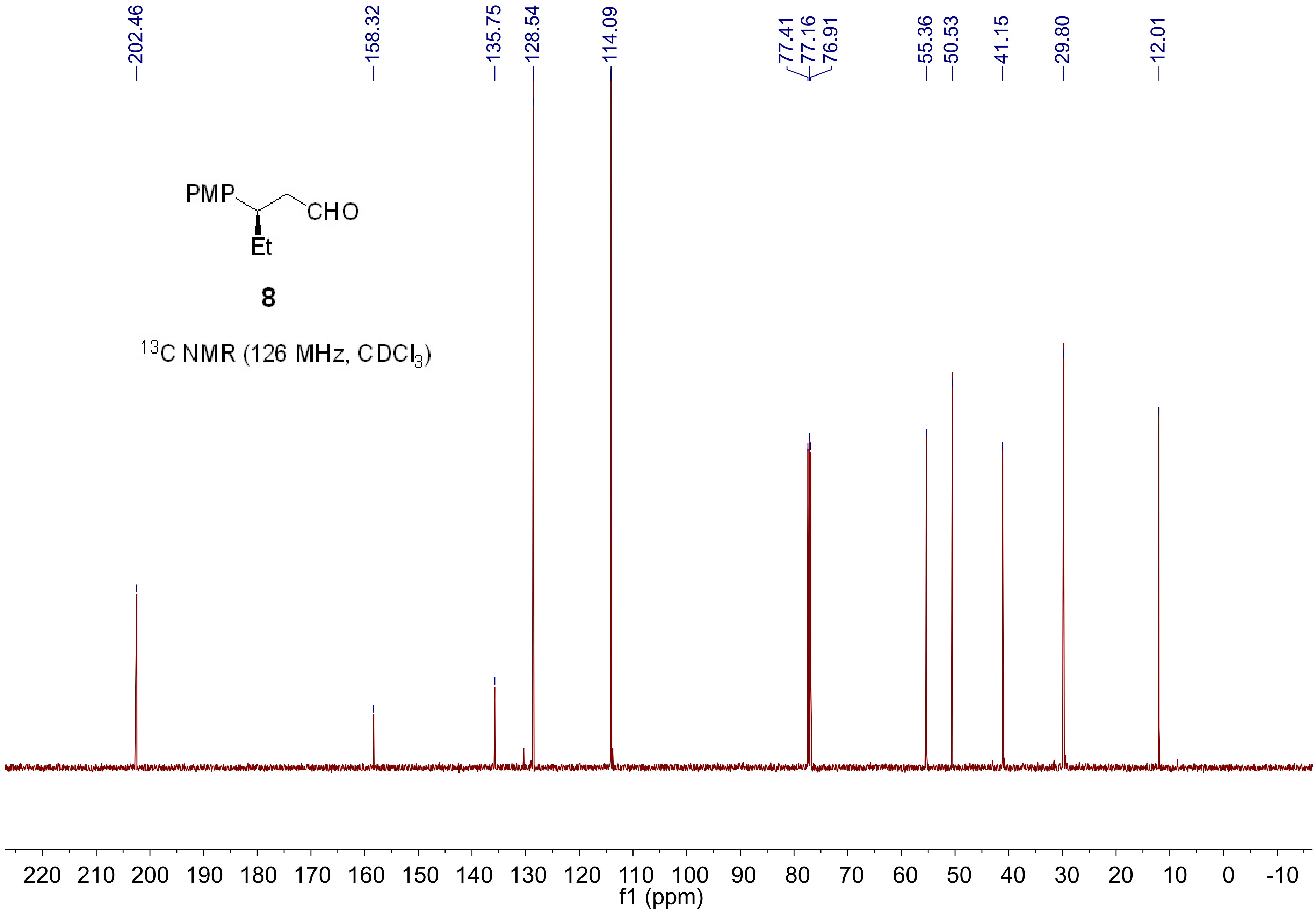

Supplementary Fig. 189. ^{13}C NMR (126 MHz, $CDCl_3$) spectra for compound **8**

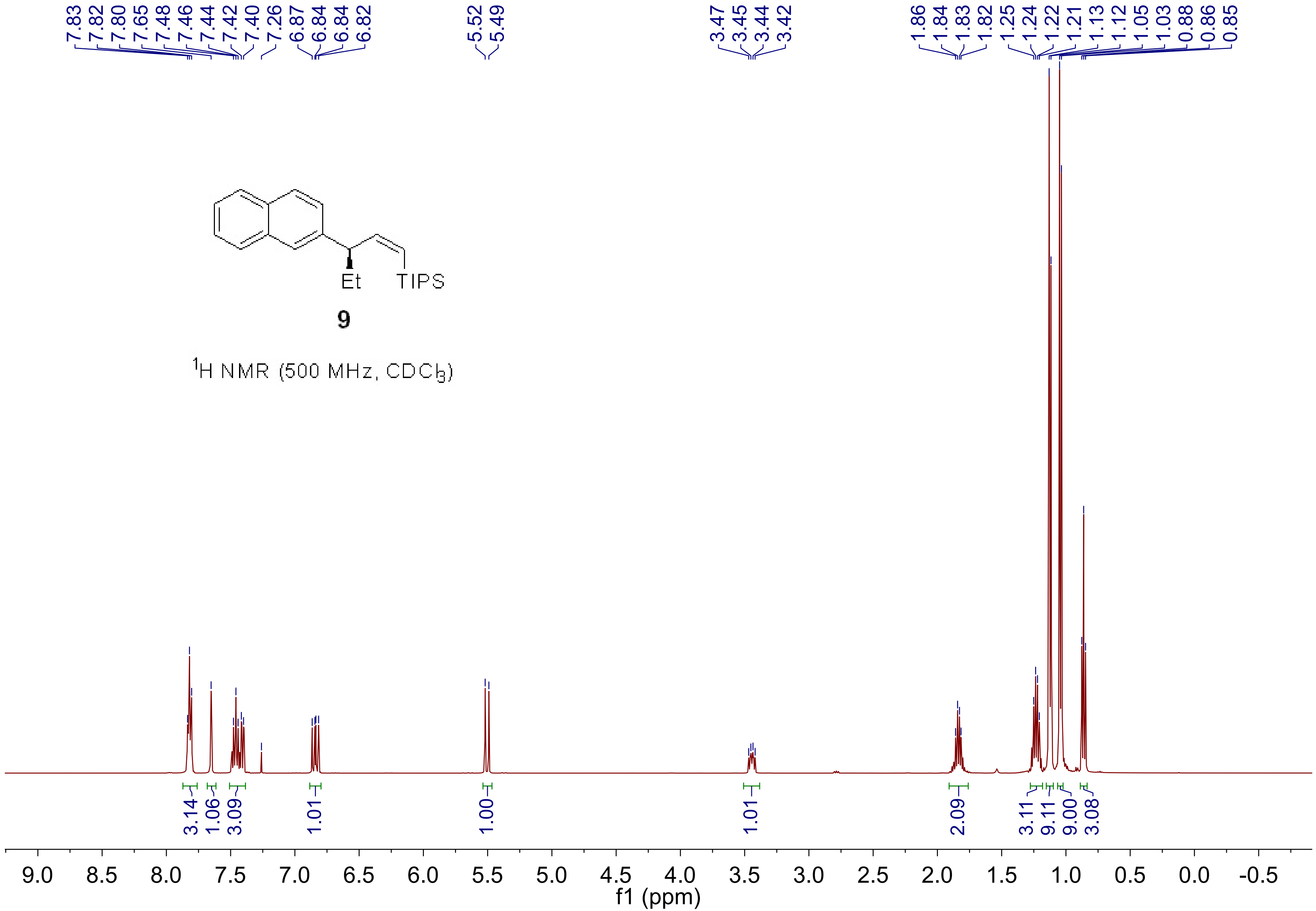

Supplementary Fig. 190. ^{1}H NMR (500 MHz, $CDCl_3$) spectra for compound **9**

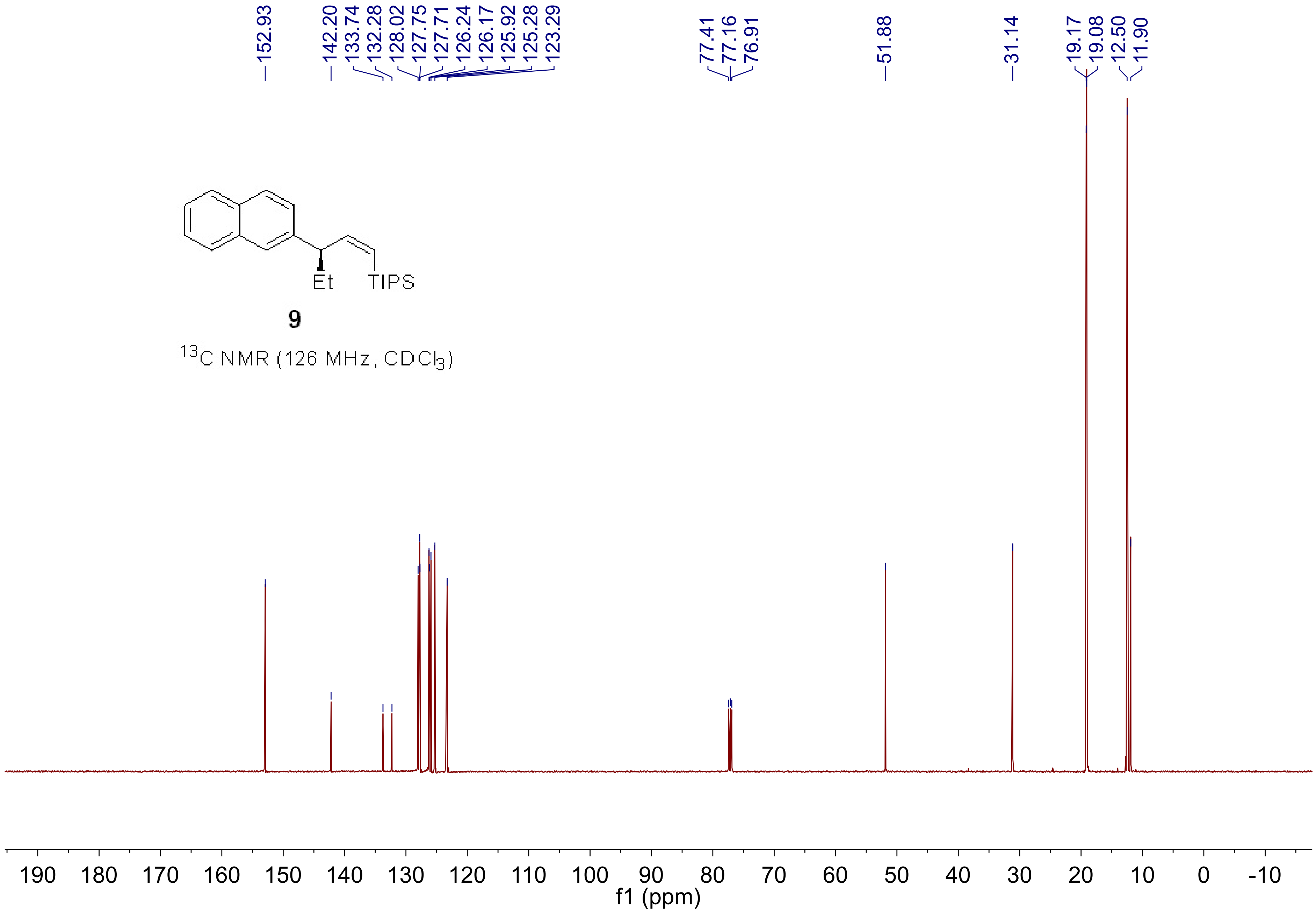

Supplementary Fig. 191. ^{13}C NMR (126 MHz, $CDCl_3$) spectra for compound **9**

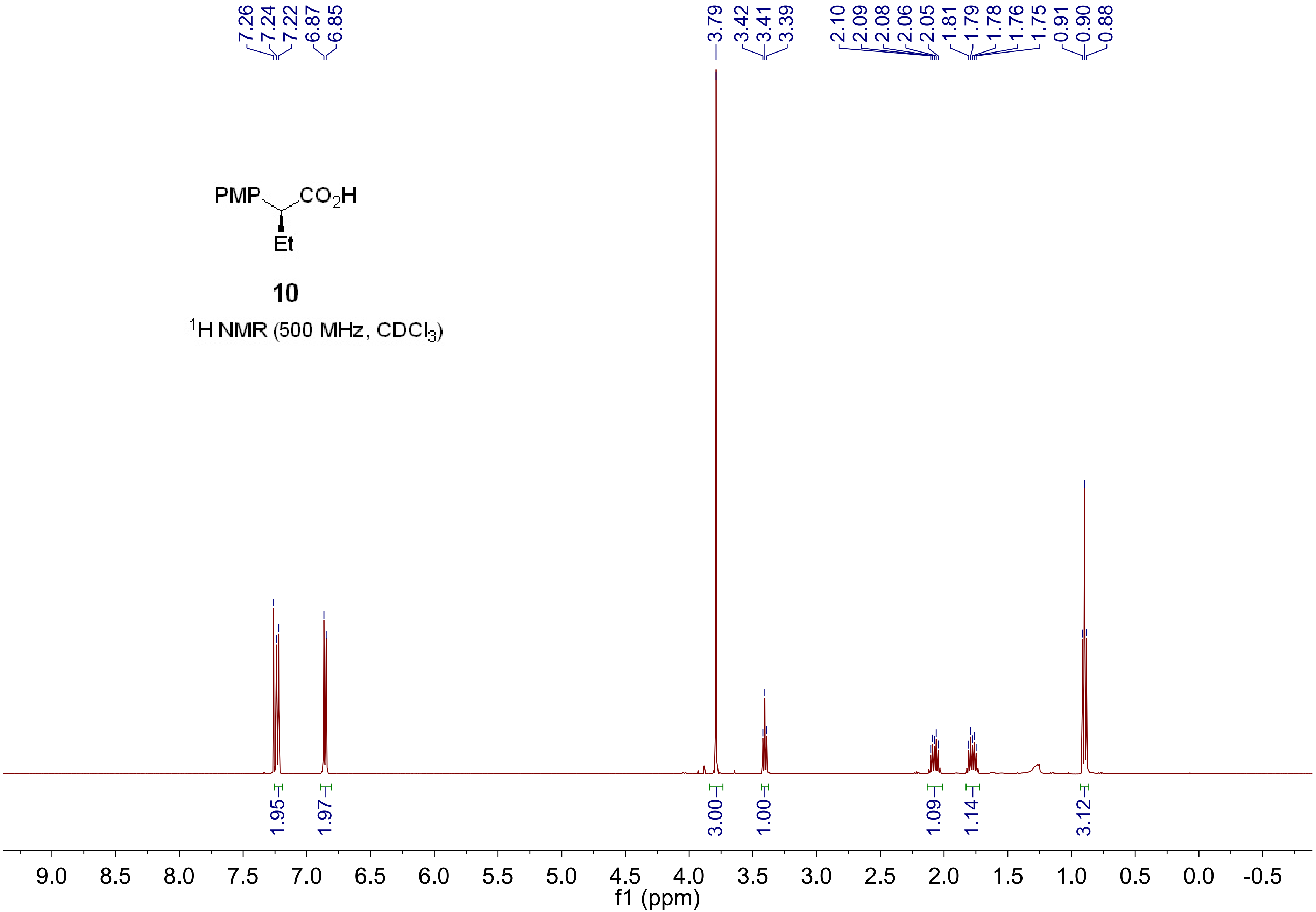

Supplementary Fig. 192. ^{1}H NMR (500 MHz, $CDCl_3$) spectra for compound **10**

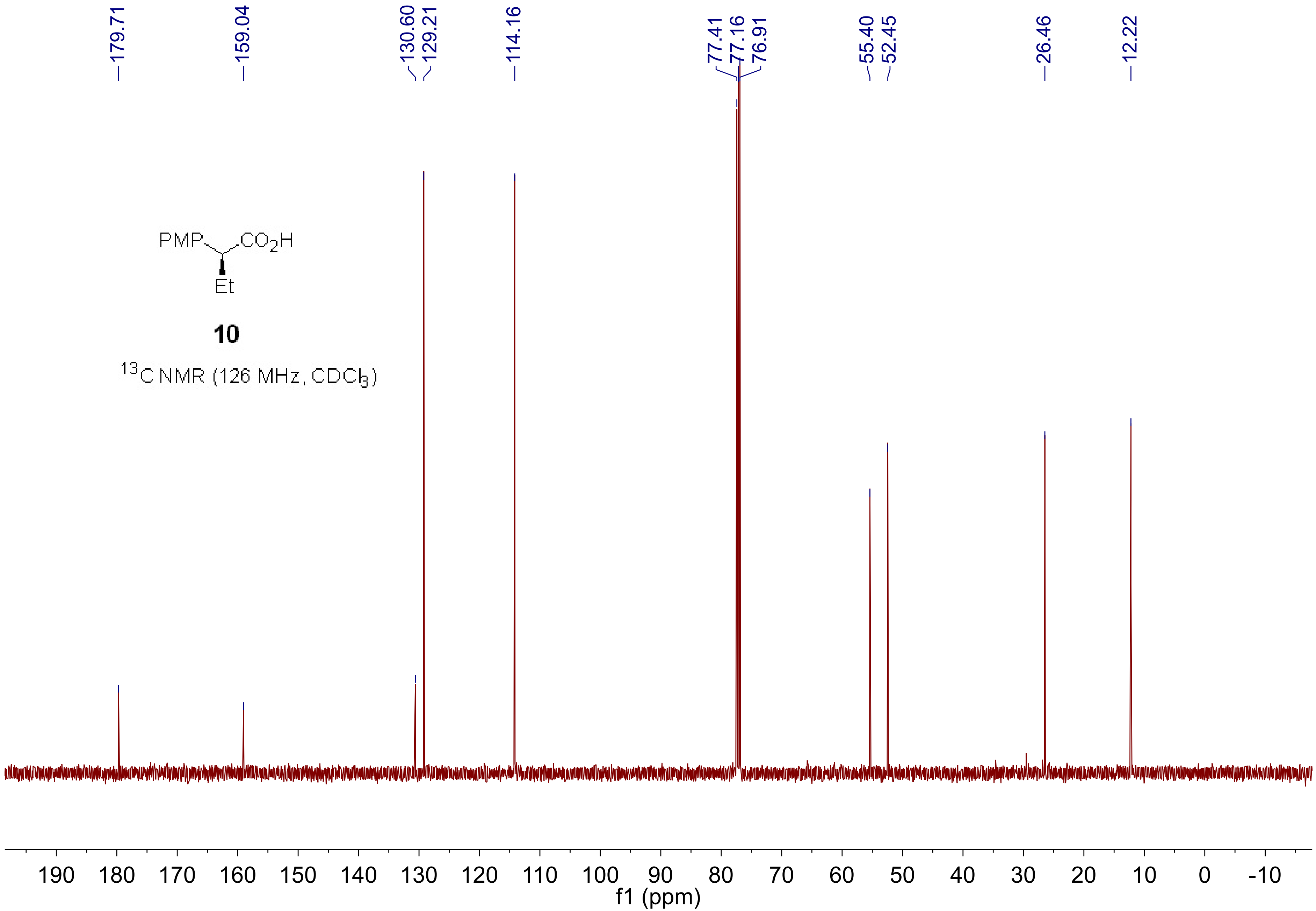

Supplementary Fig. 193. ^{13}C NMR (126 MHz, $CDCl_3$) spectra for compound **10**

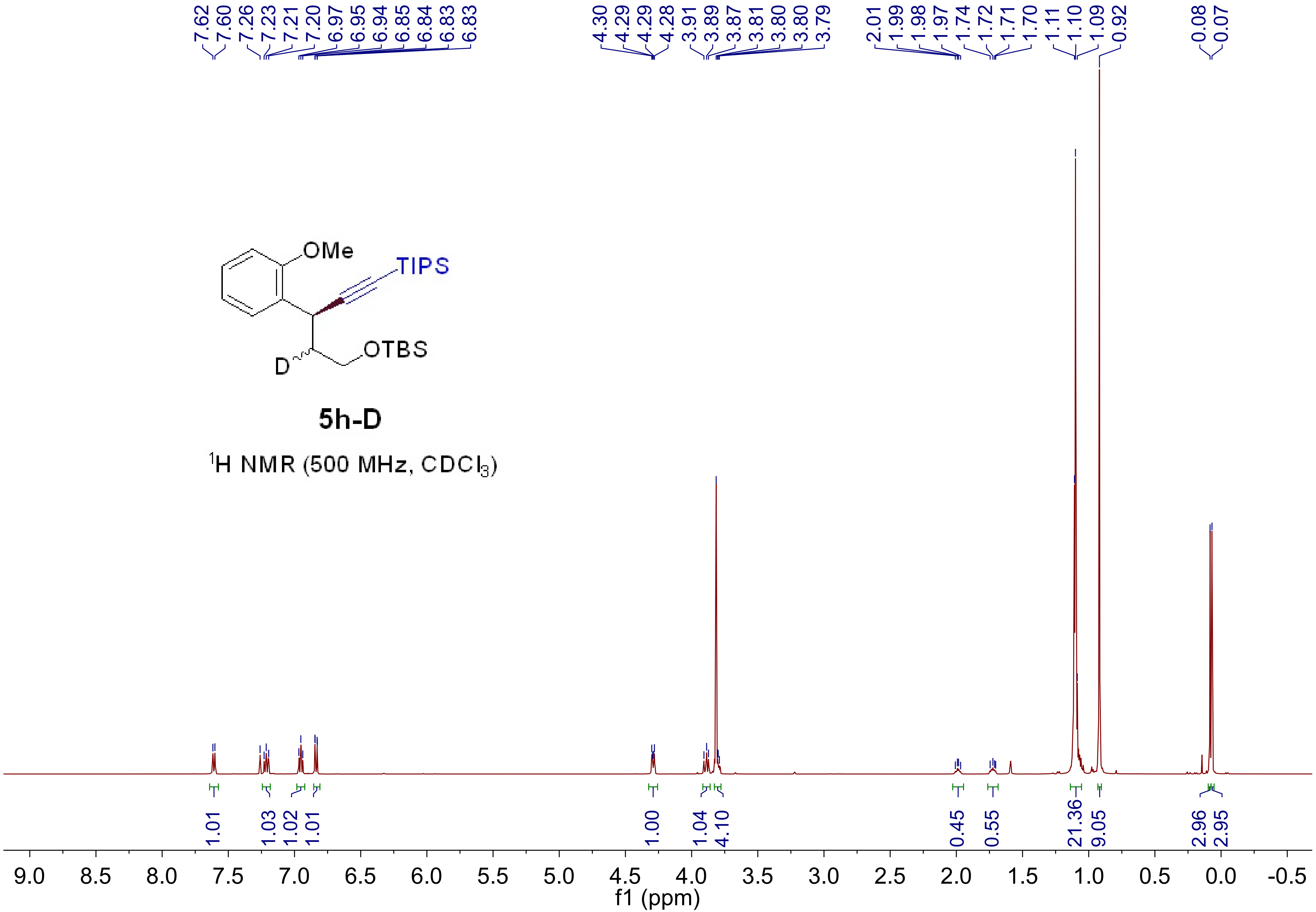

Supplementary Fig. 194. ^{1}H NMR (500 MHz, $CDCl_3$) spectra for compound **5h-D**

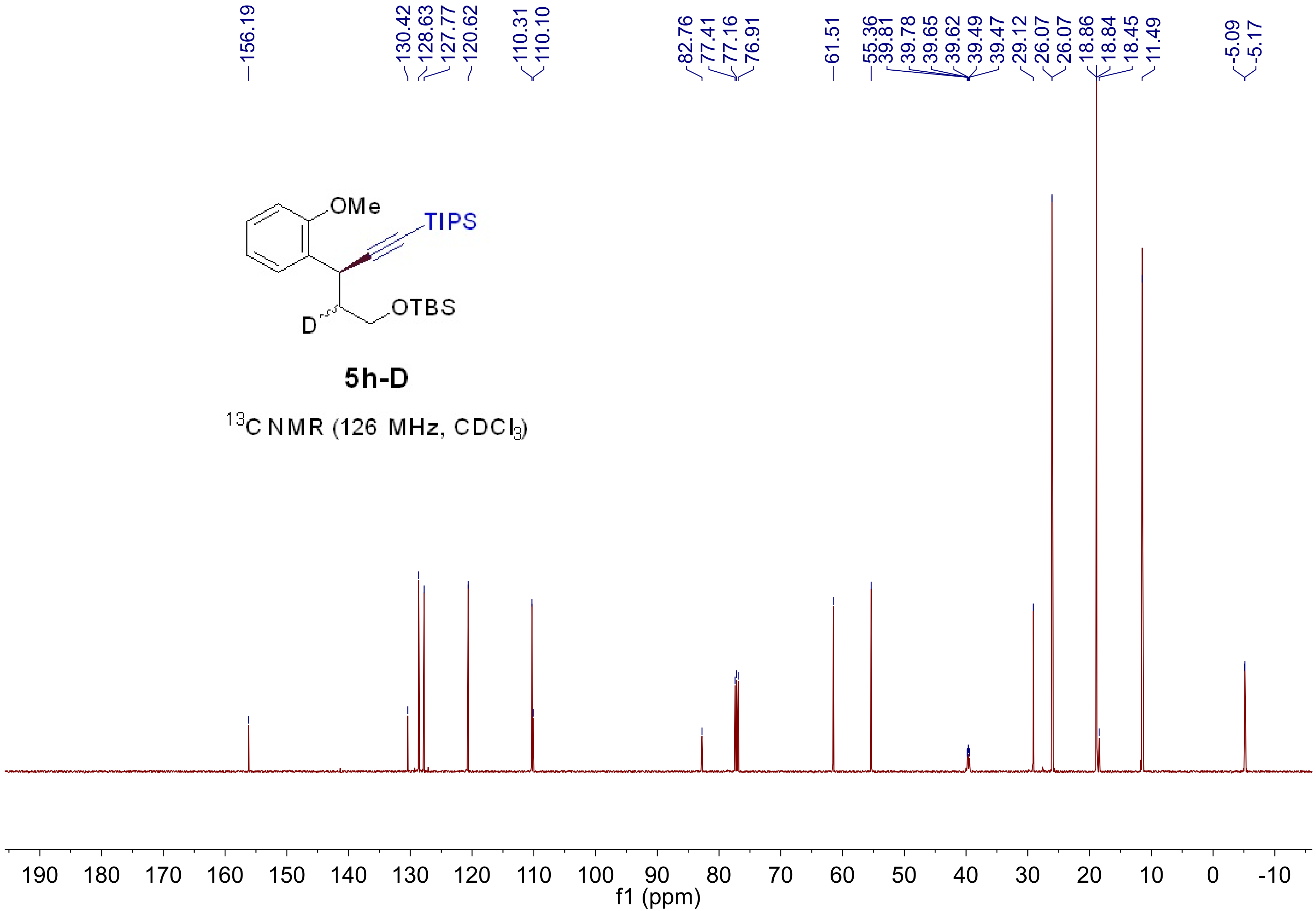

Supplementary Fig. 195. ^{13}C NMR (126 MHz, $CDCl_3$) spectra for compound **5h-D**

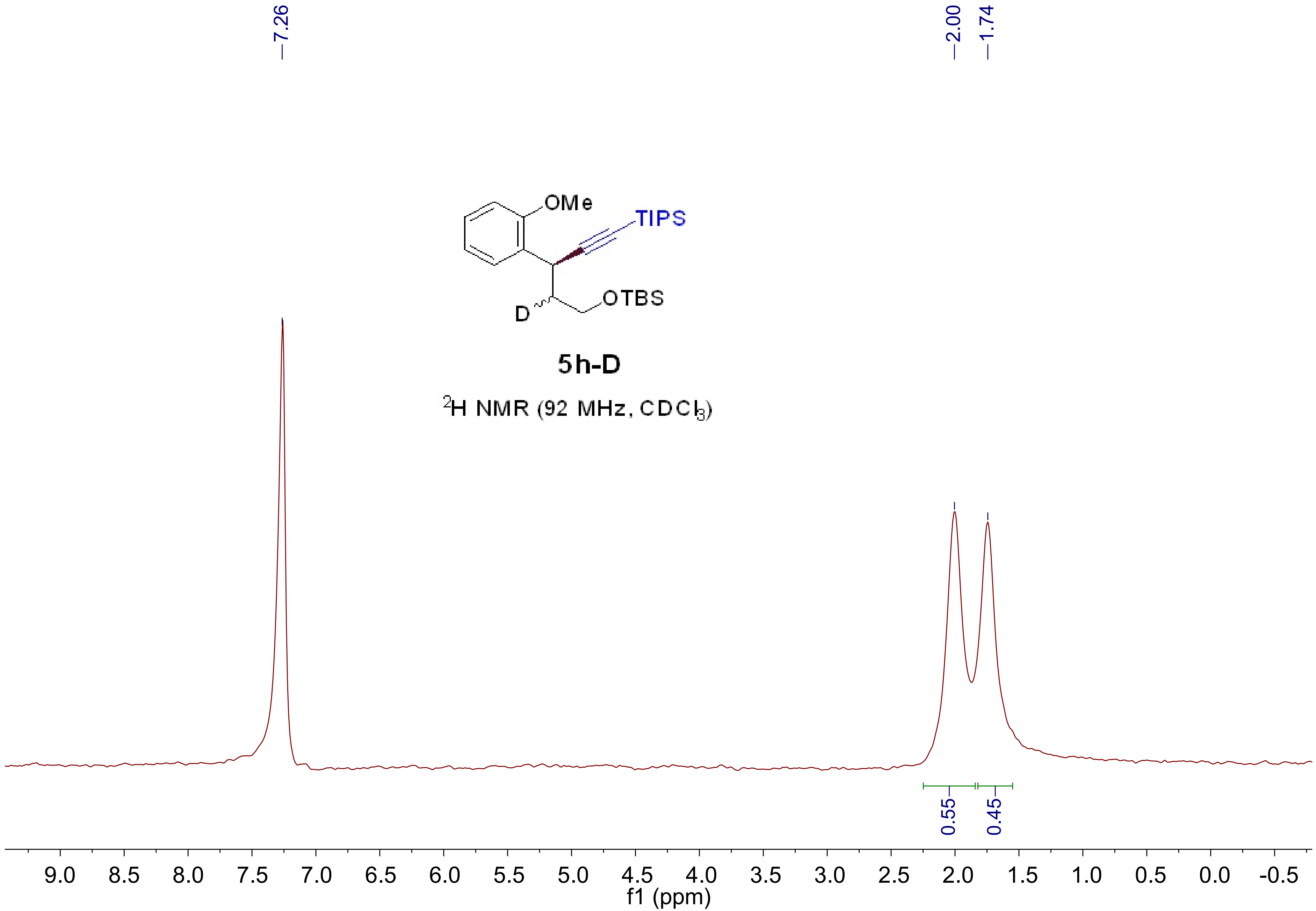

Supplementary Fig. 196. ^{2}H NMR (92 MHz, $CDCl_3$) spectra for compound **5h-D**

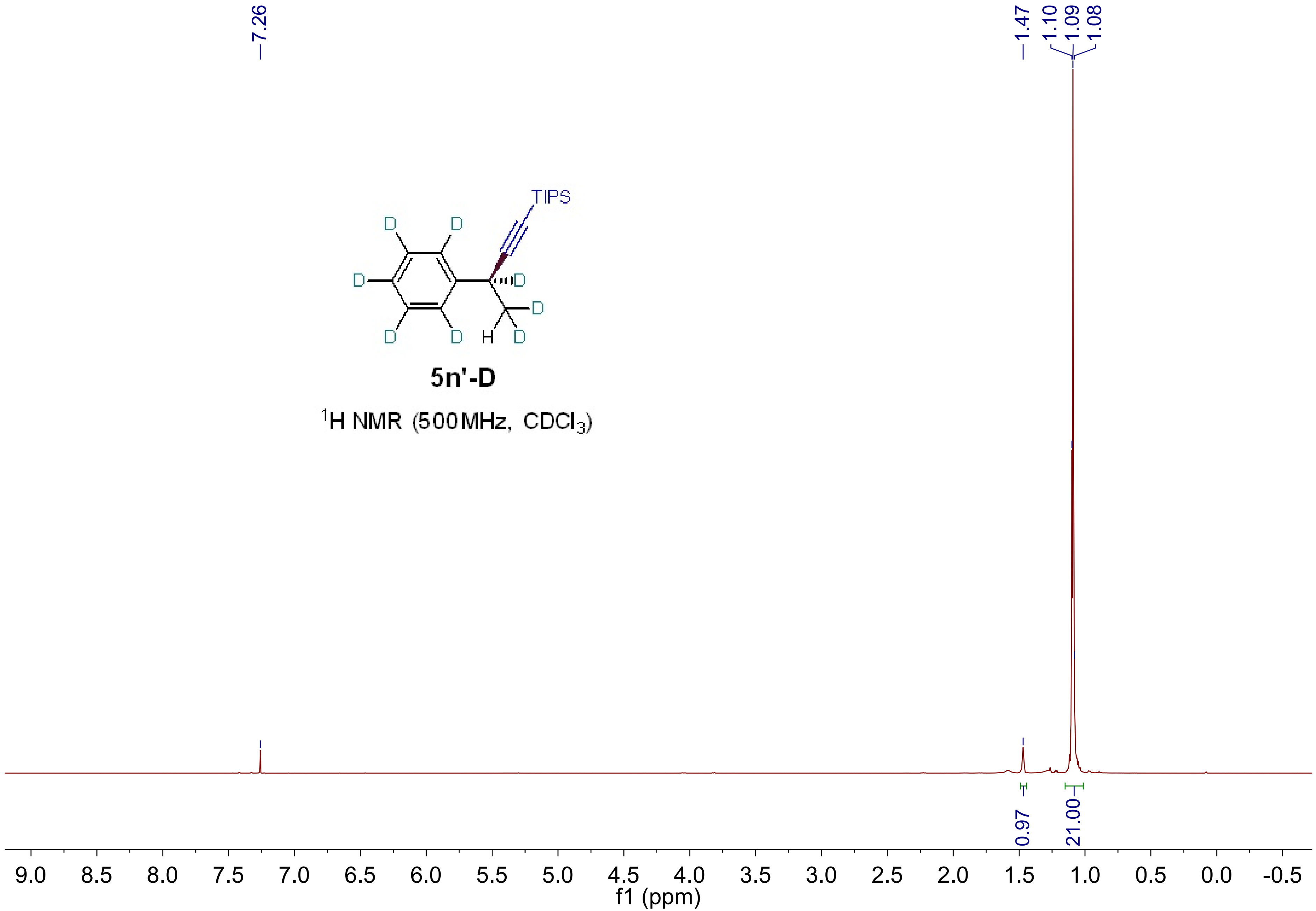

Supplementary Fig. 197. ^{1}H NMR (500 MHz, $CDCl_3$) spectra for compound **5n'-D**

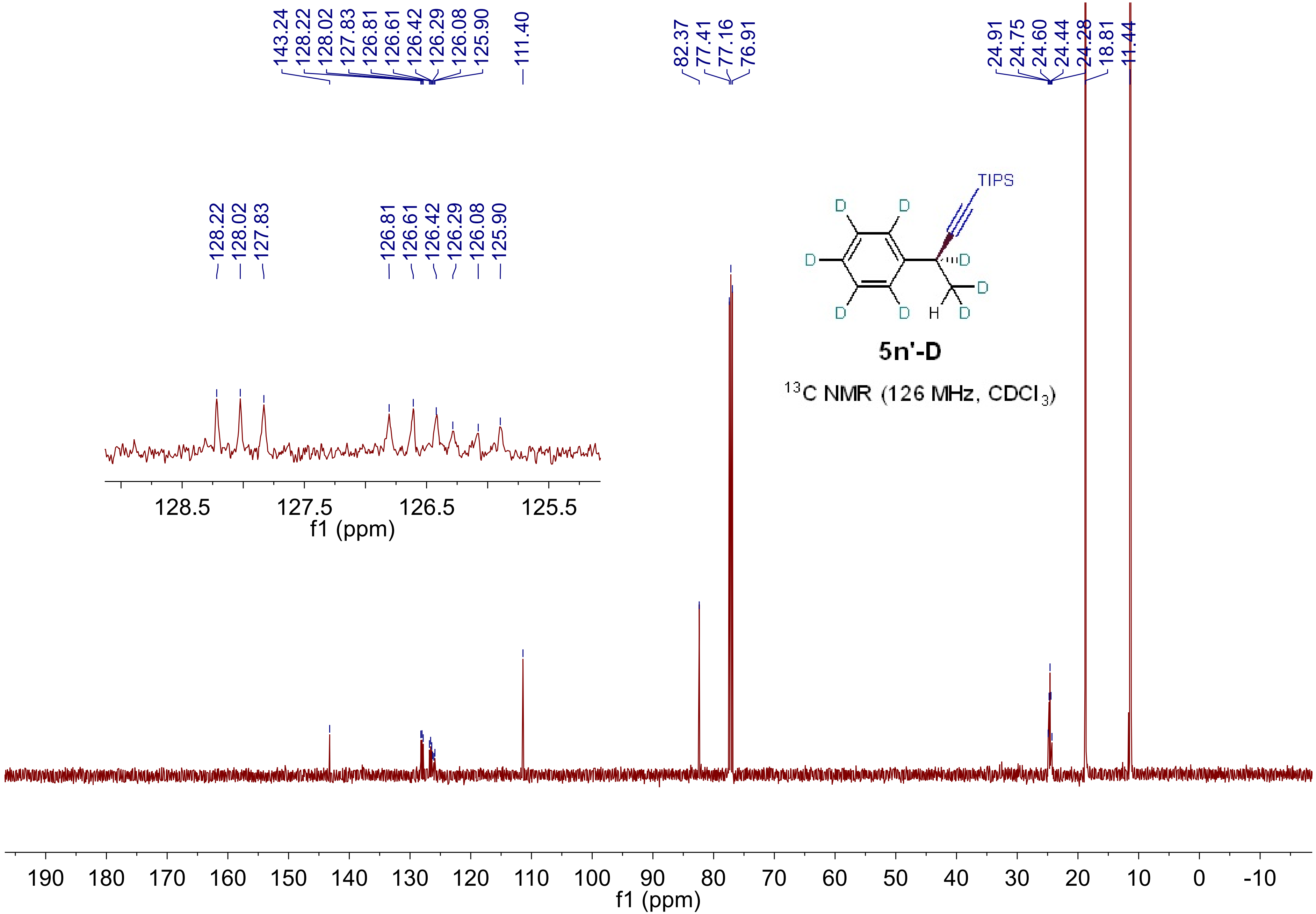

Supplementary Fig. 198. ^{13}C NMR (126 MHz, CDCl$_3$) spectra for compound **5n'-D**

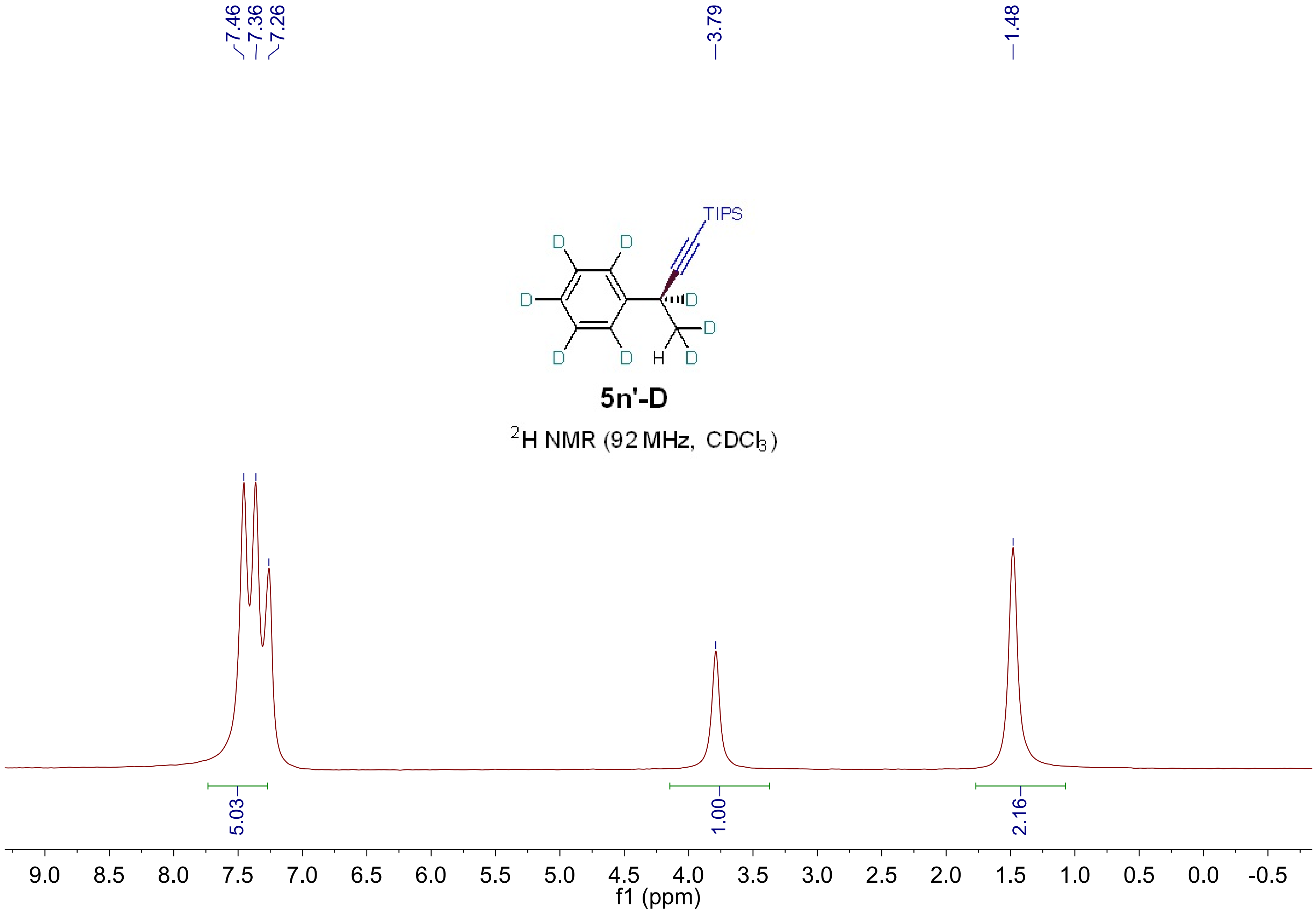

Supplementary Fig. 199. ^{2}H NMR (92 MHz, $CDCl_3$) spectra for compound **5n'-D**

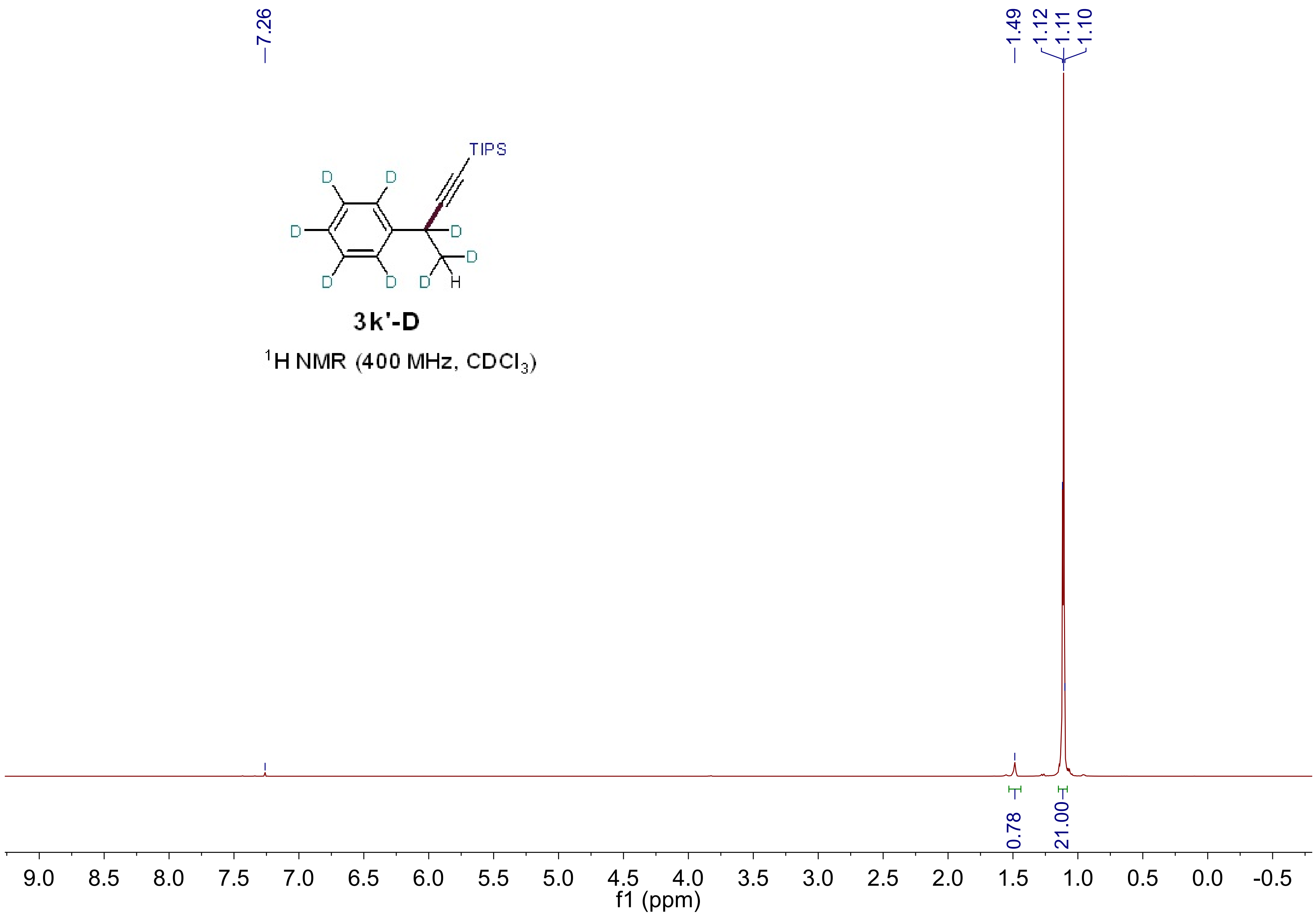

Supplementary Fig. 200. ^{1}H NMR (400 MHz, $CDCl_3$) spectra for compound **3k'-D**

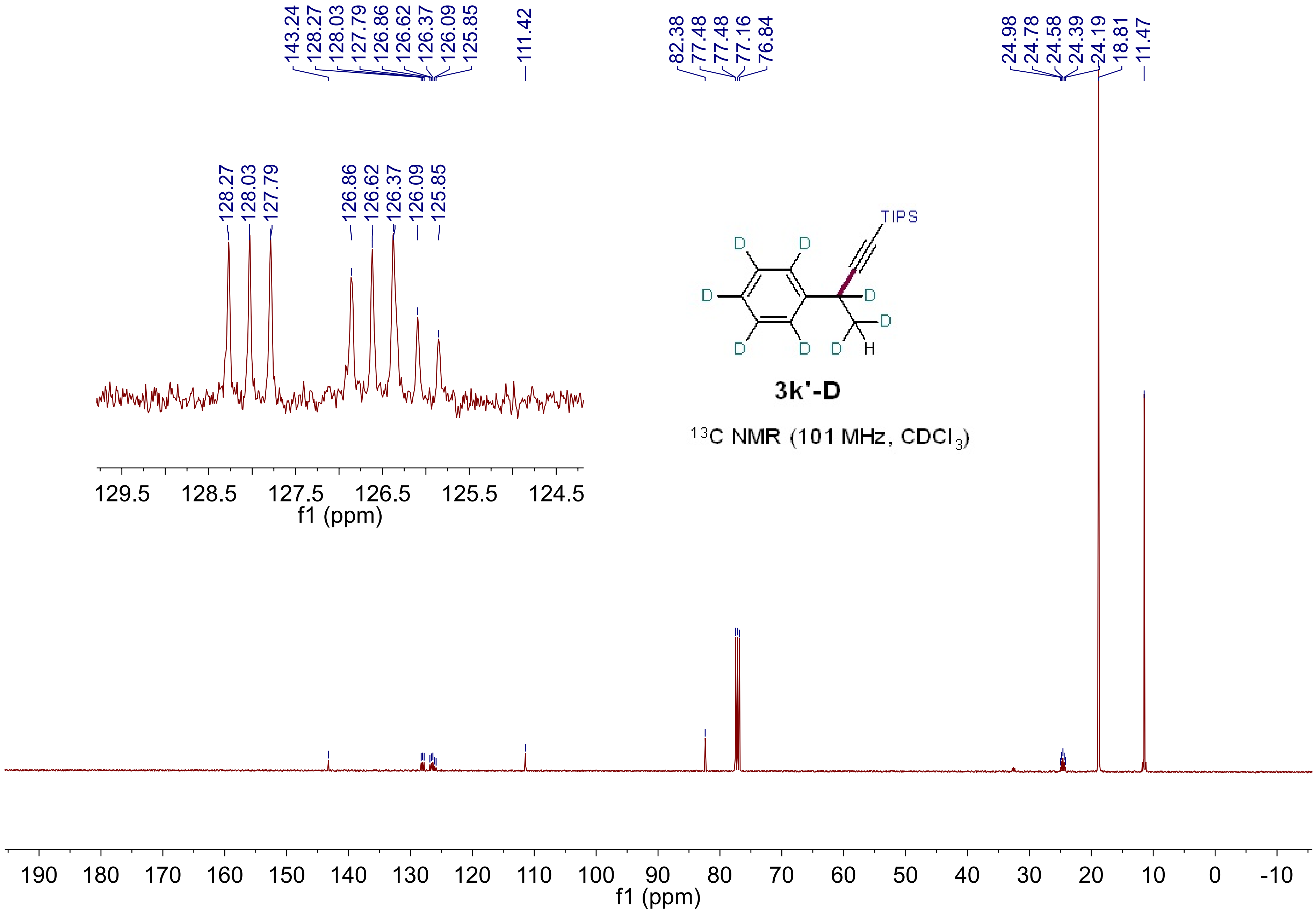

Supplementary Fig. 201. ^{13}C NMR (101 MHz, $CDCl_3$) spectra for compound **3k'-D**

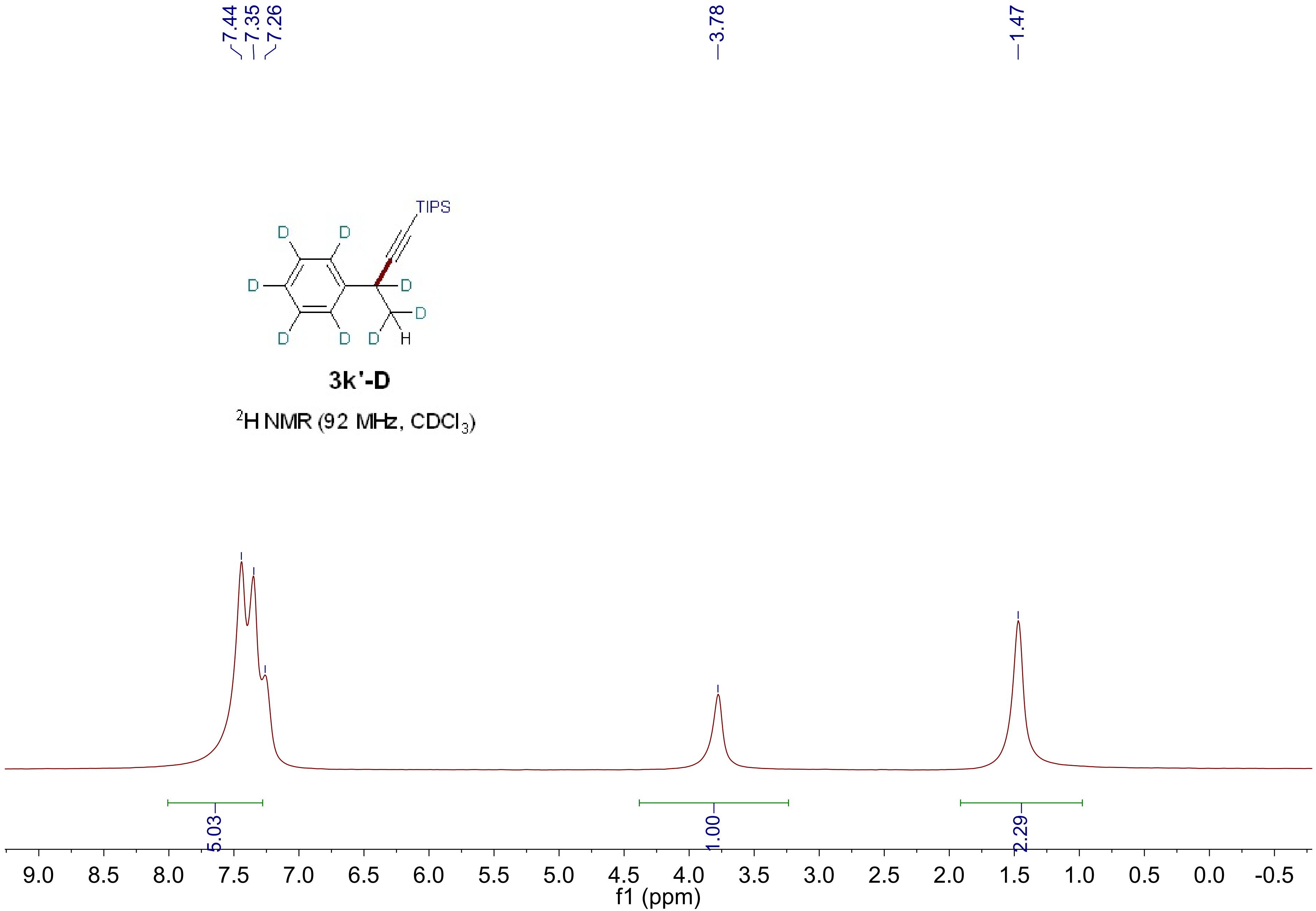

Supplementary Fig. 202. ^{2}H NMR (92 MHz, $CDCl_3$) spectra for compound **3k'-D**

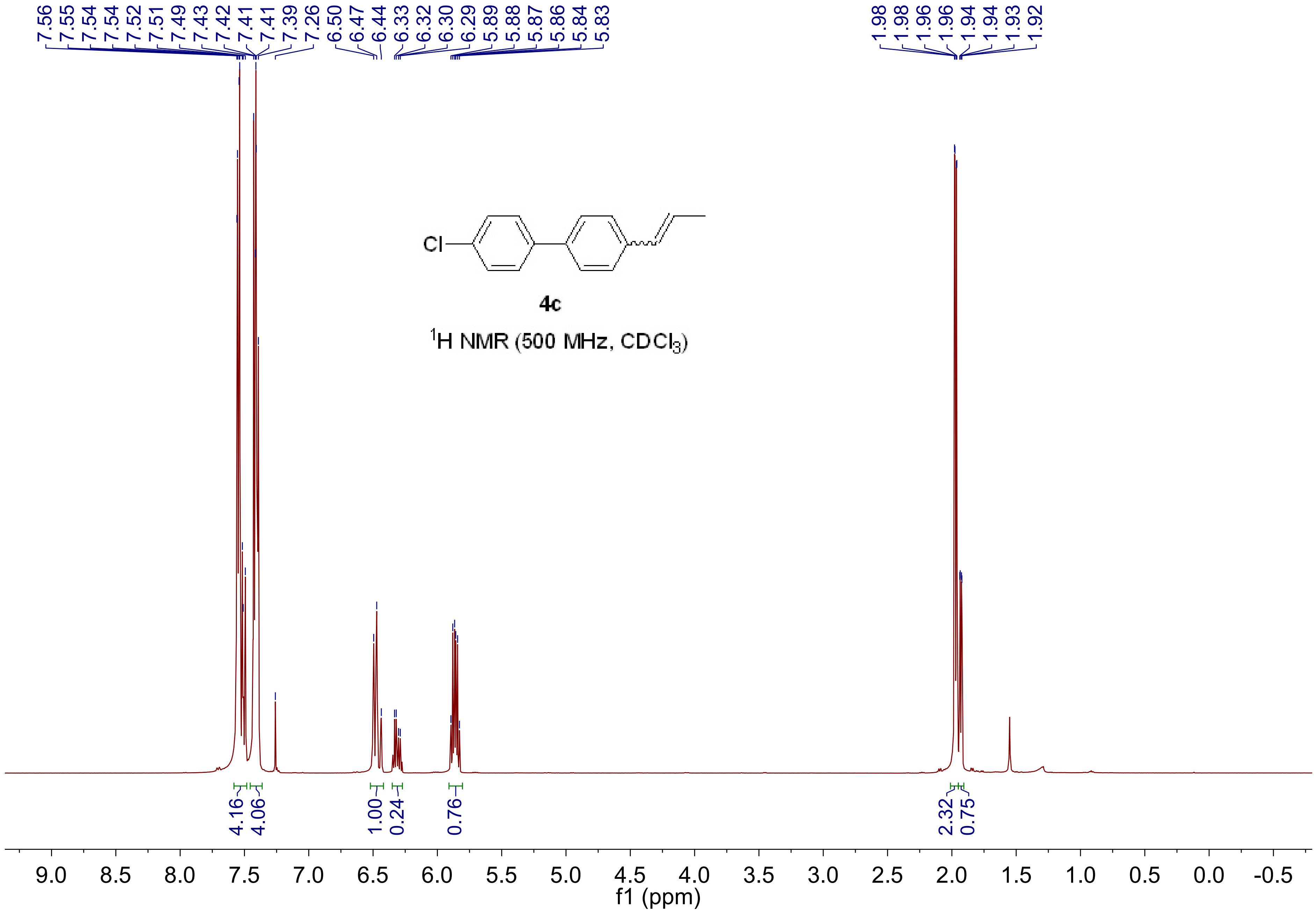

Supplementary Fig. 203. ^{1}H NMR (500 MHz, $CDCl_3$) spectra for compound **4c**

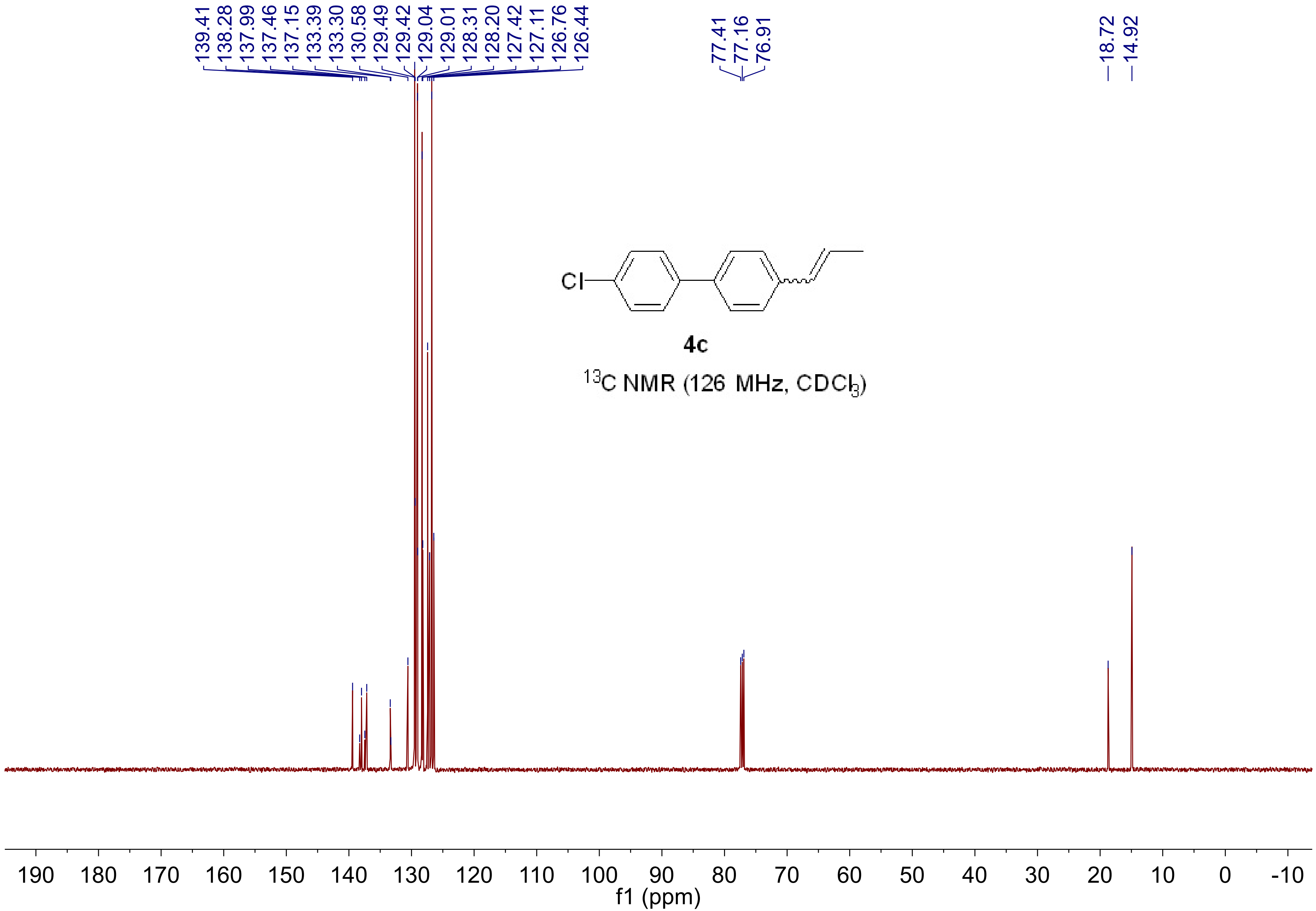

Supplementary Fig. 204. ^{13}C NMR (126 MHz, $CDCl_3$) spectra for compound **4c**

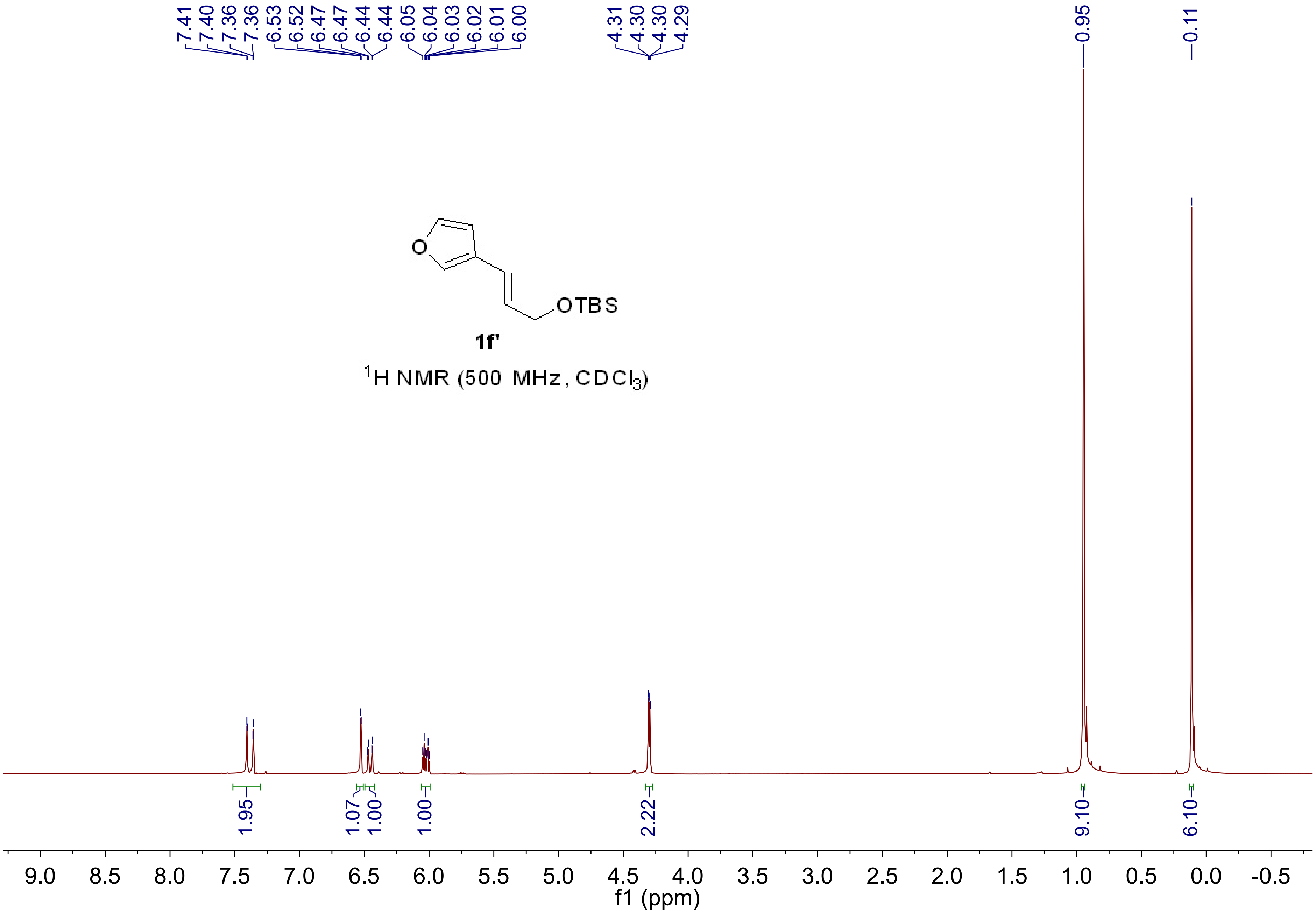

Supplementary Fig. 205. ^{1}H NMR (500 MHz, $CDCl_{3}$) spectra for compound **1f'**

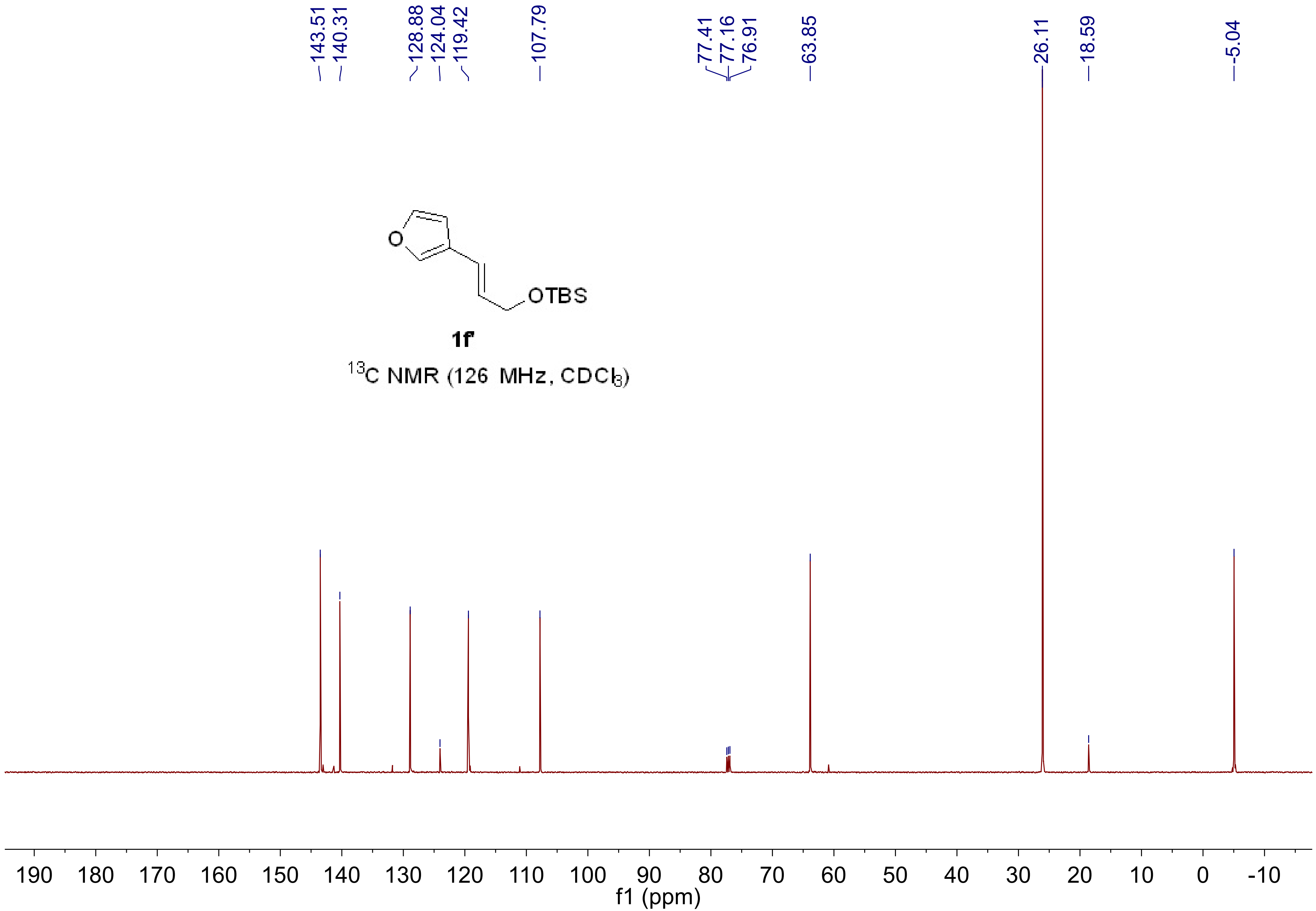

Supplementary Fig. 206. ^{1}H NMR (126 MHz, $CDCl_3$) spectra for compound **1f'**

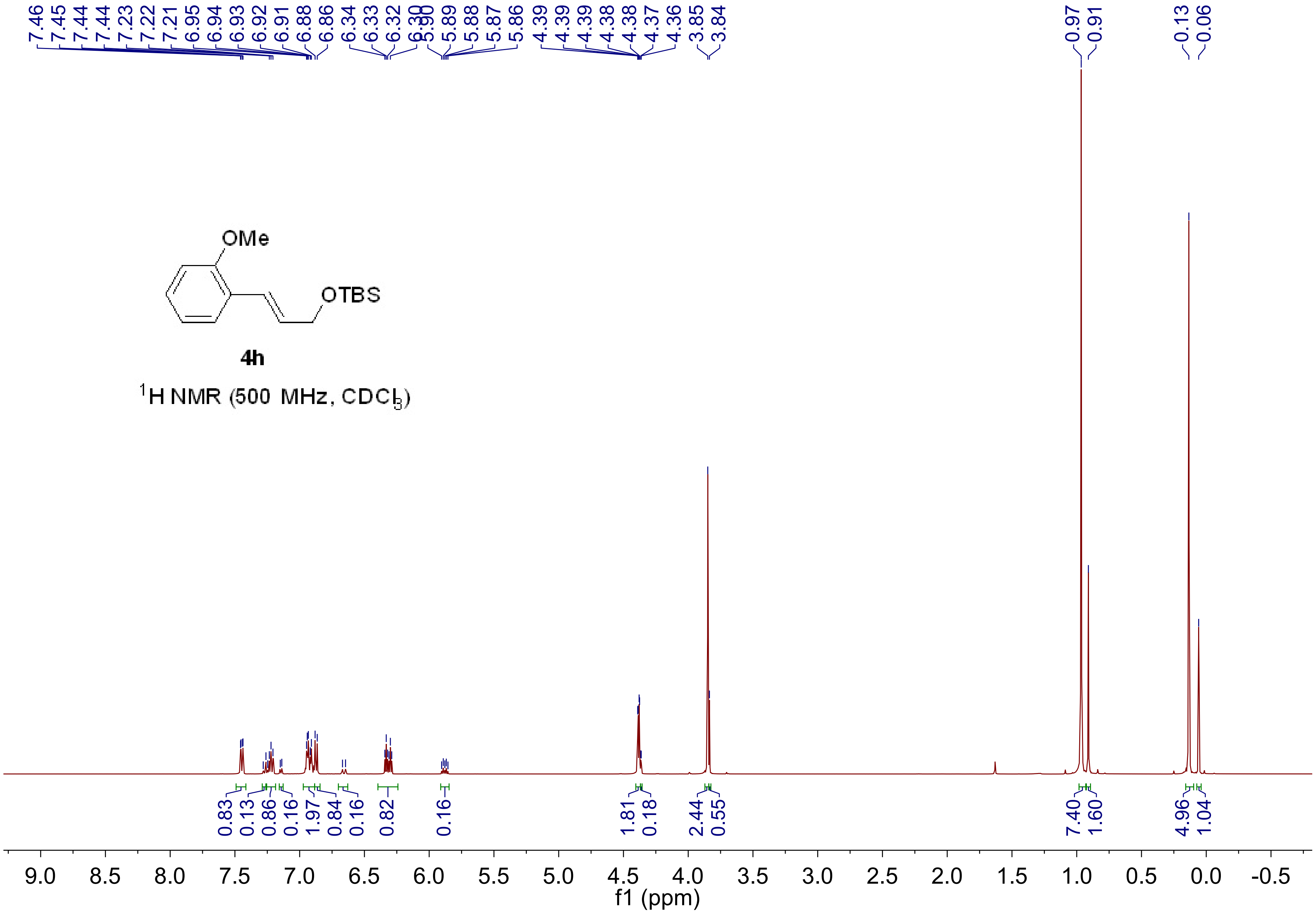

Supplementary Fig. 207. ^{1}H NMR (500 MHz, $CDCl_3$) spectra for compound **4h**

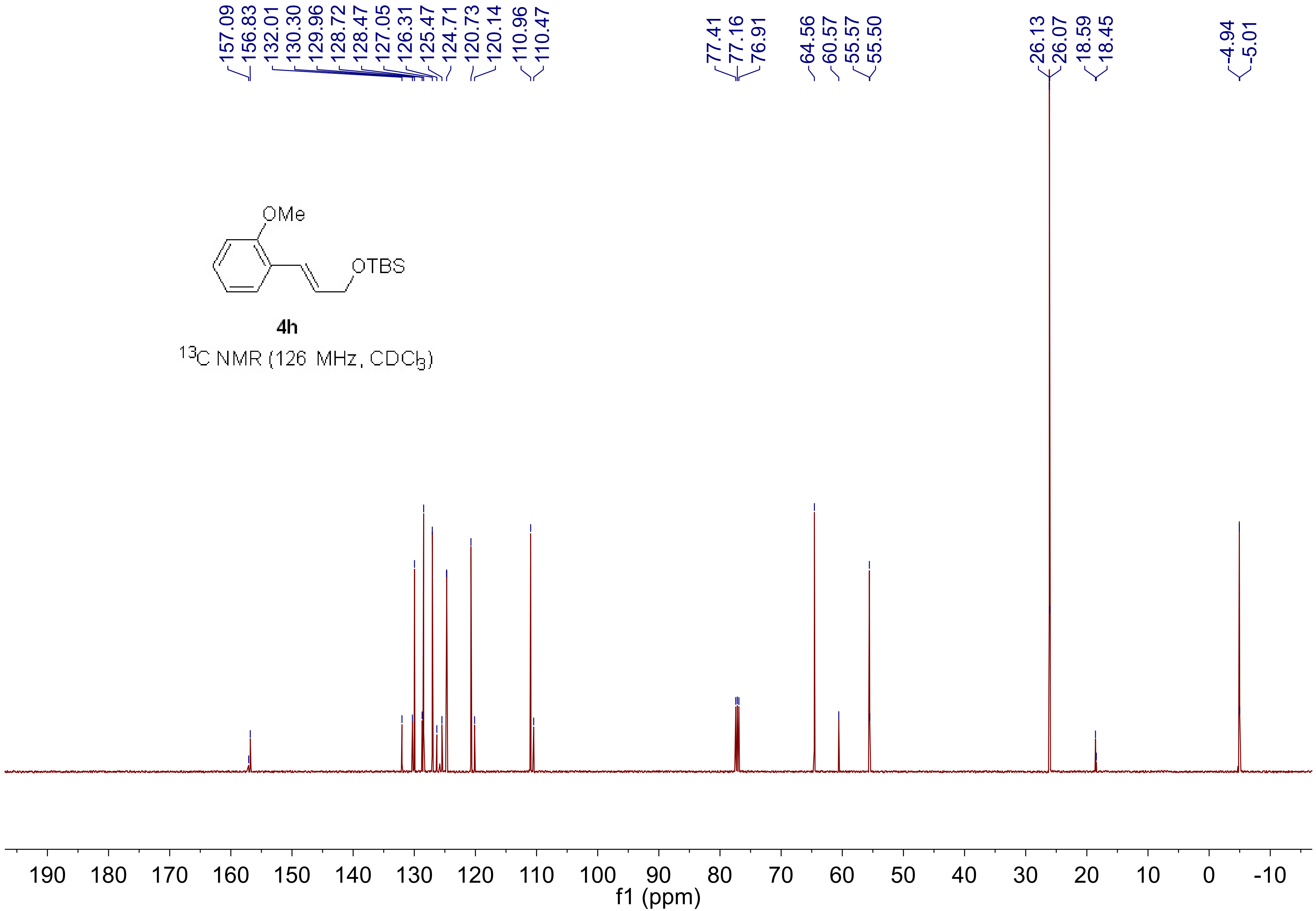

Supplementary Fig. 208. ^{13}C NMR (126 MHz, $CDCl_3$) spectra for compound **4h**

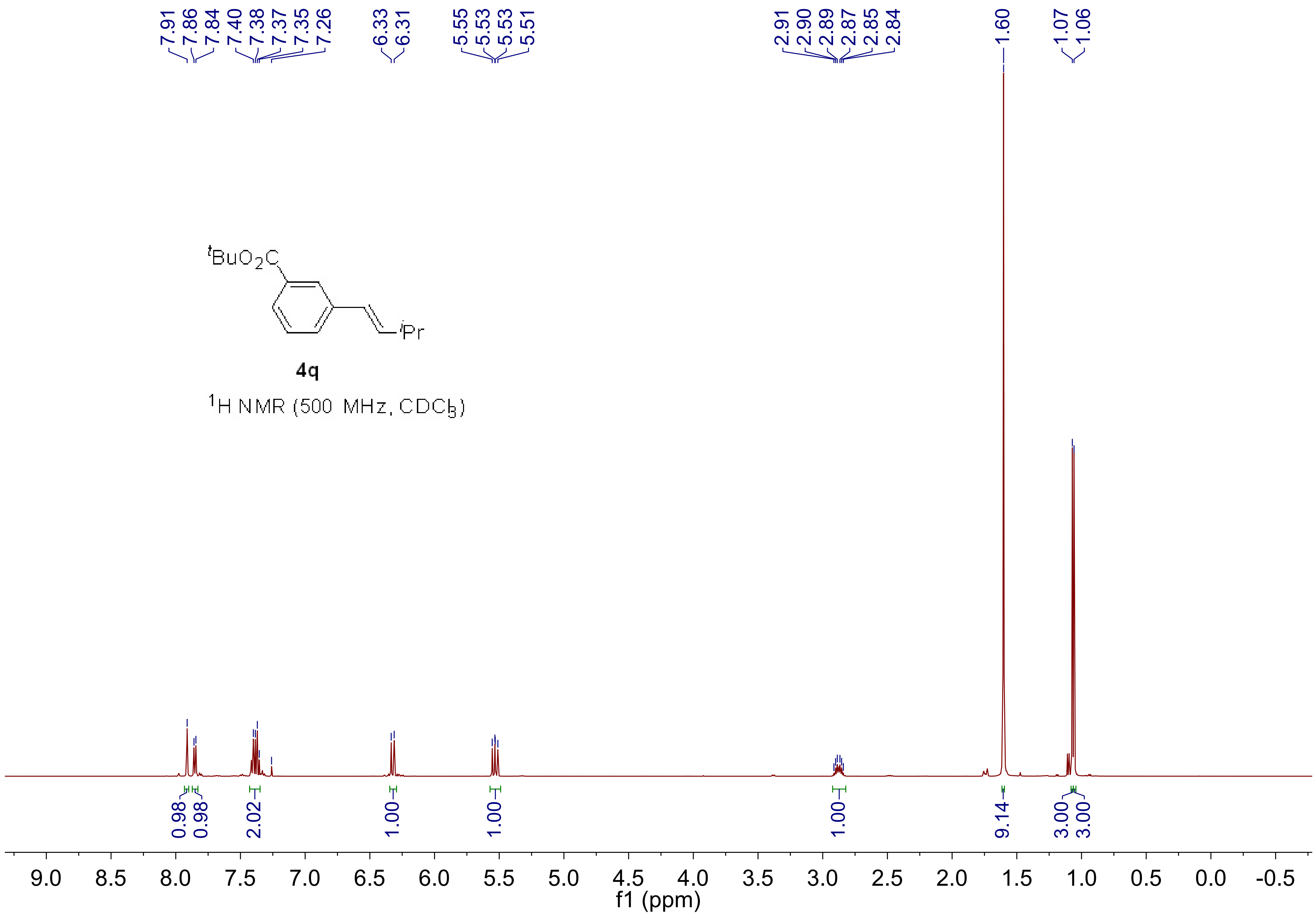

Supplementary Fig. 209. ^{1}H NMR (500 MHz, $CDCl_3$) spectra for compound **4q**

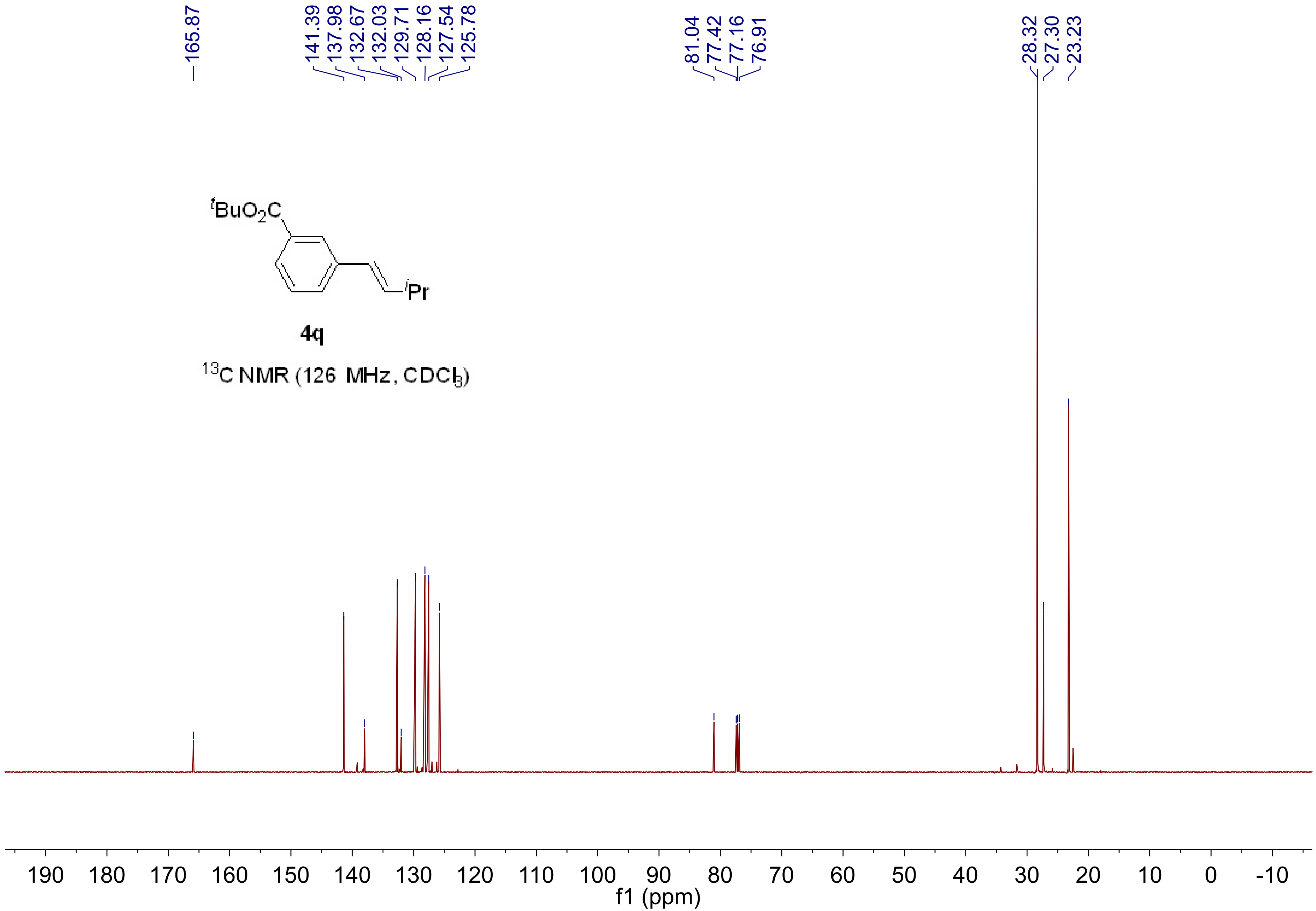

Supplementary Fig. 210. ^{13}C NMR (126 MHz, $CDCl_3$) spectra for compound **4q**

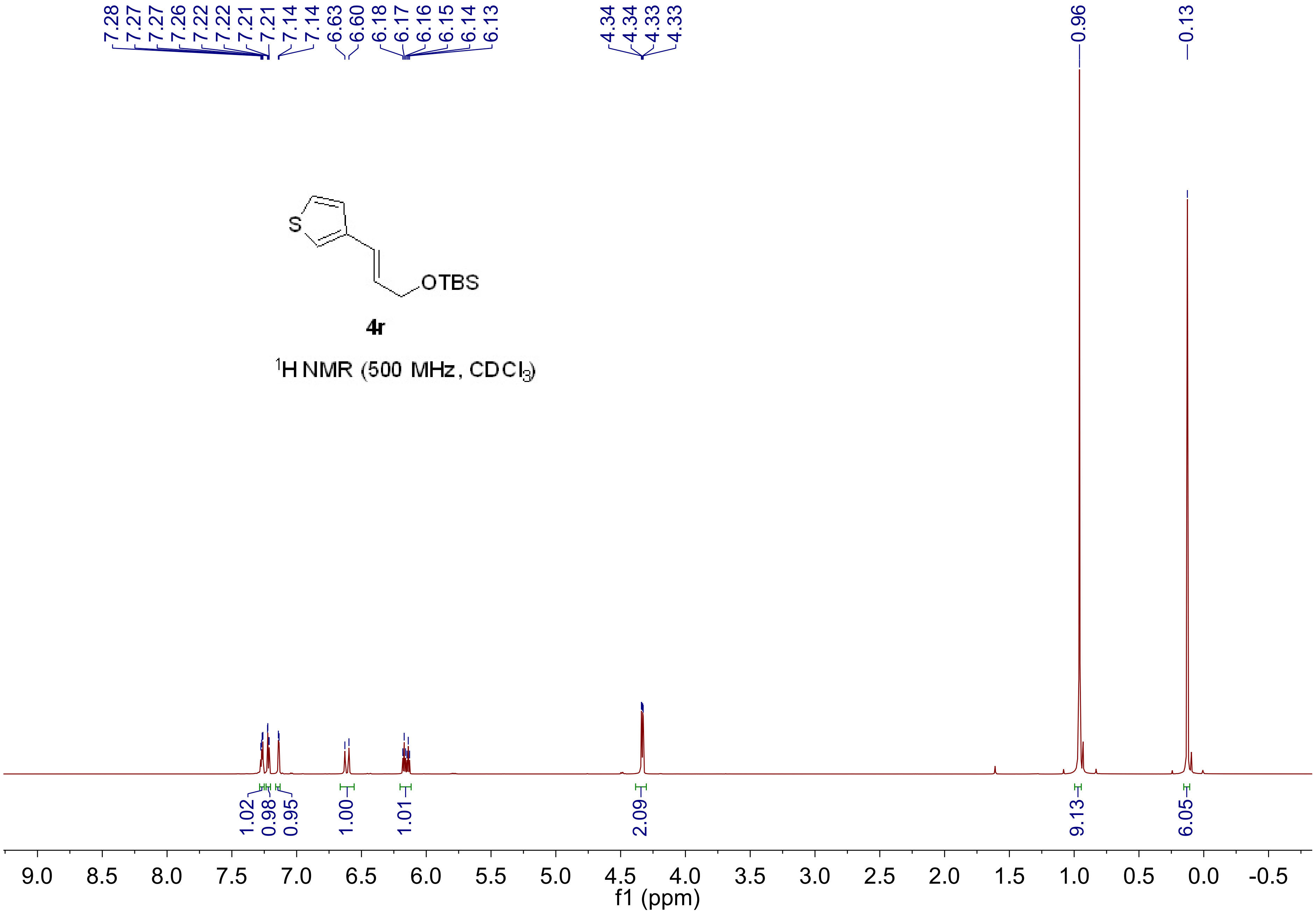

Supplementary Fig. 211. ^{1}H NMR (500 MHz, $CDCl_3$) spectra for compound **4r**

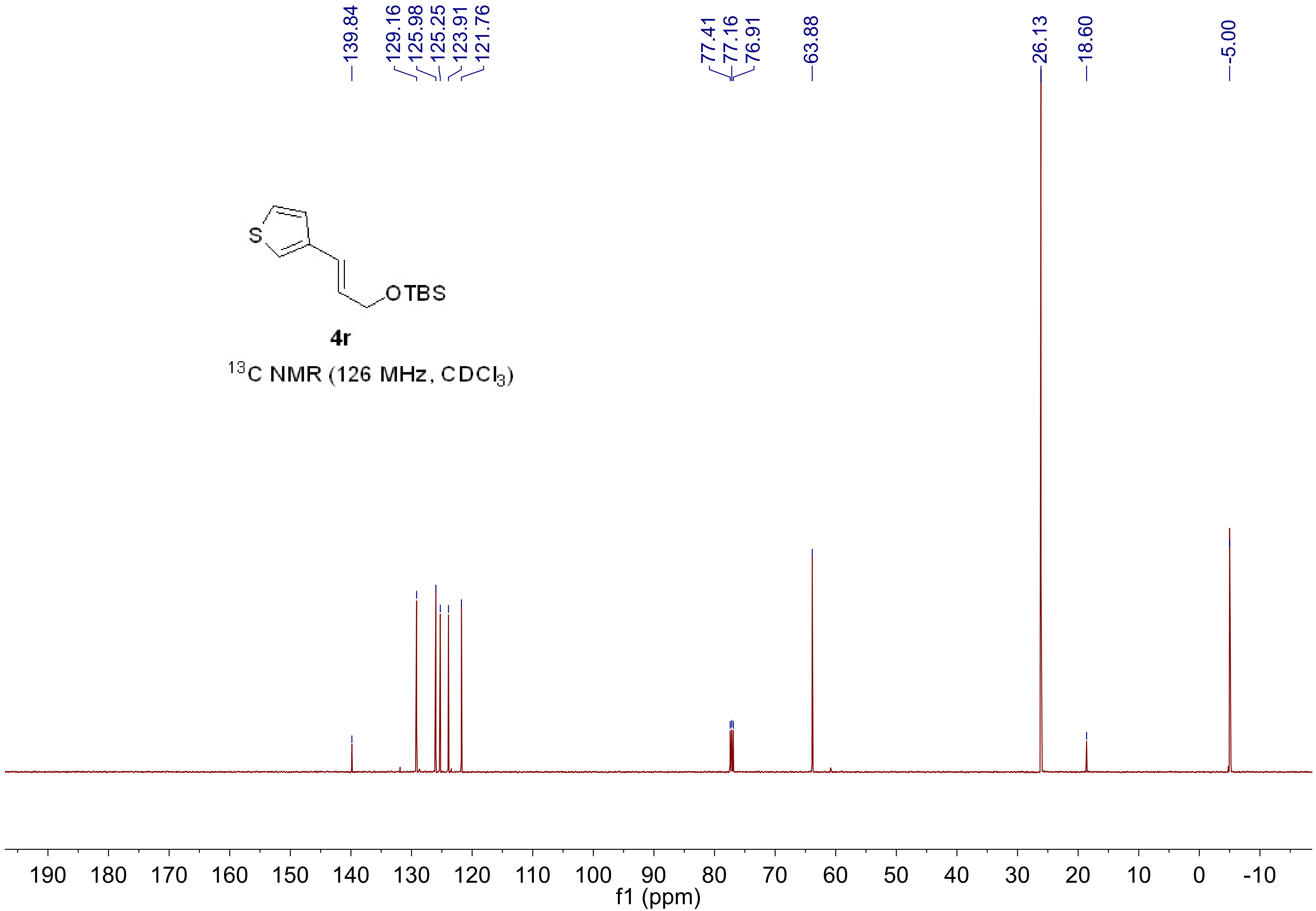

Supplementary Fig. 212. ^{13}C NMR (126 MHz, $CDCl_3$) spectra for compound **4r**

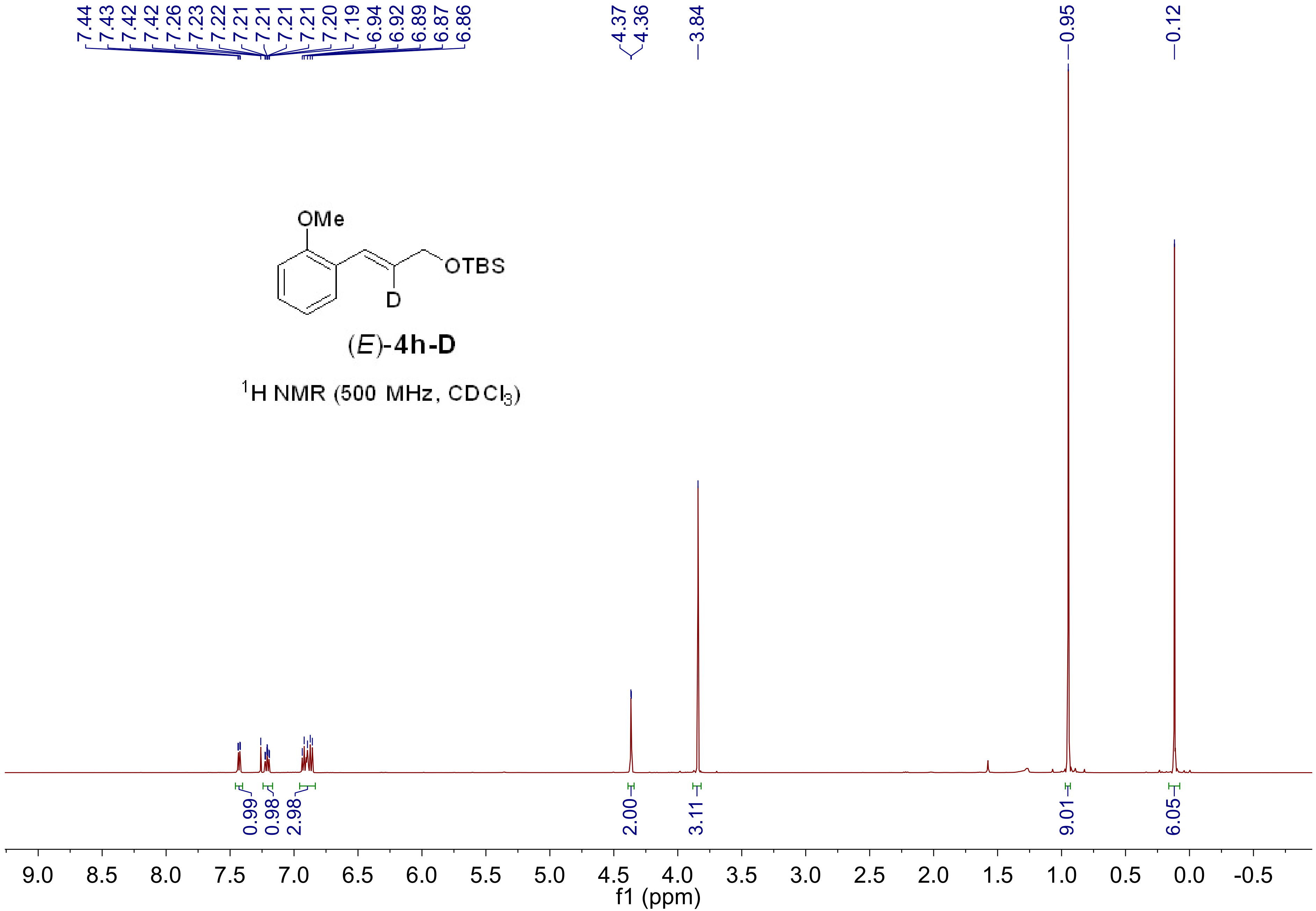

Supplementary Fig. 213. ^{1}H NMR (500 MHz, $CDCl_3$) spectra for compound (*E*)-**4h-D**

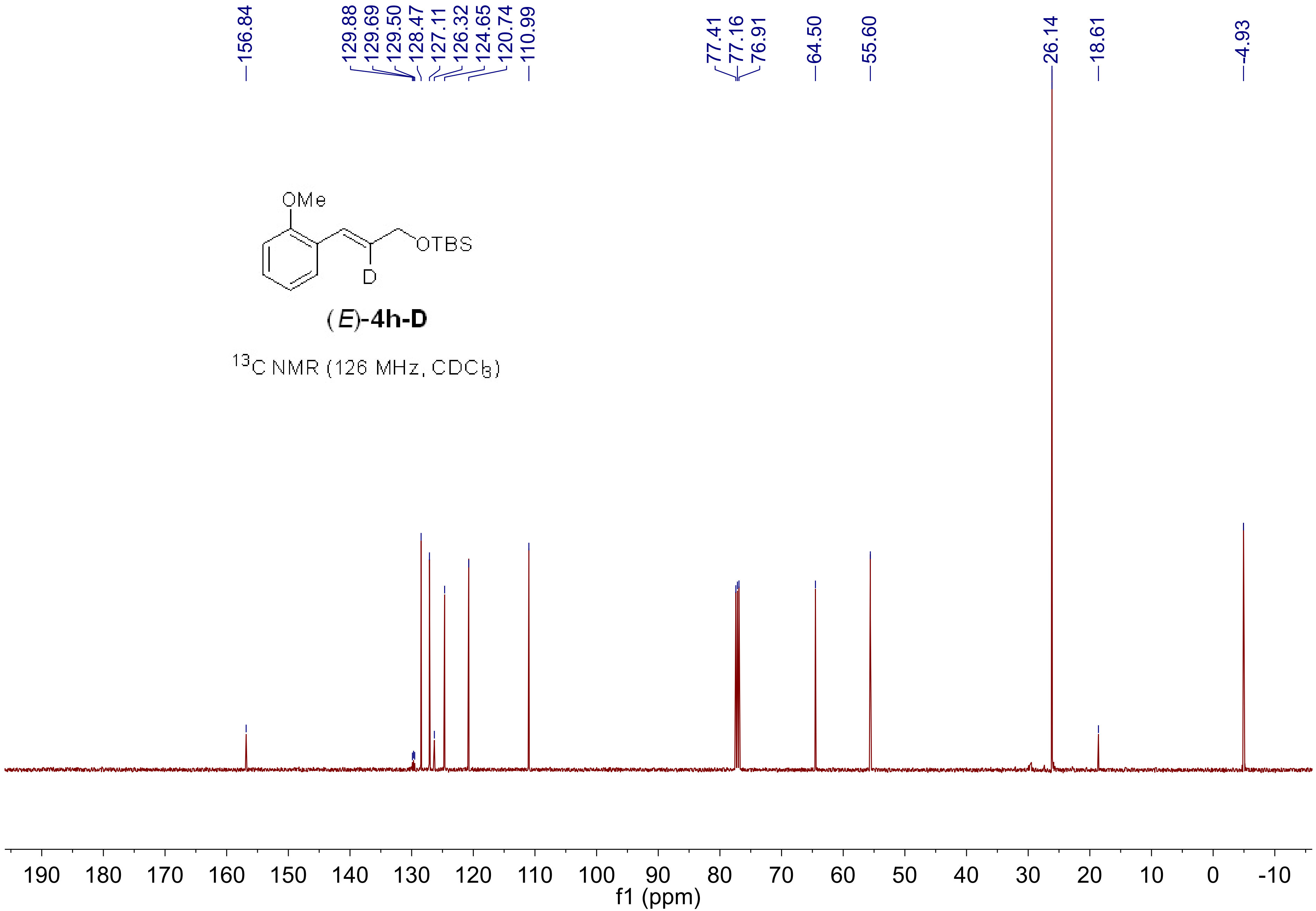

Supplementary Fig. 214. ^{1}H NMR (126 MHz, $CDCl_3$) spectra for compound (*E*)-**4h-D**

Data File H:\JXL HPLC\JXL-03-67-1-OD(1)-RAC.D
Sample Name: JXL-03-67-1-OD(1)

===

Acq. Operator : 系统 Seq. Line : 7
Acq. Instrument : HPLC-1260 Location : 93
Injection Date : 7/30/2020 7:51:24 PM Inj : 1
Inj Volume : 3.000 µl
Different Inj Volume from Sample Entry! Actual Inj Volume : 0.200 µl
Acq. Method : D:\JXL\20200730\YH 2020-07-30 16-30-45\0IPA-35-0.8-1-2-220-JXL.M
Last changed : 7/30/2020 7:54:31 PM by 系统
(modified after loading)
Analysis Method : E:\DATA\20201027\LC 2020-12-13 20-54-13\8IPA_40_0.8_4.M (Sequence Method)
Last changed : 12/18/2020 8:28:46 PM by SYSTEM
(modified after loading)
Additional Info : Peak(s) manually integrated

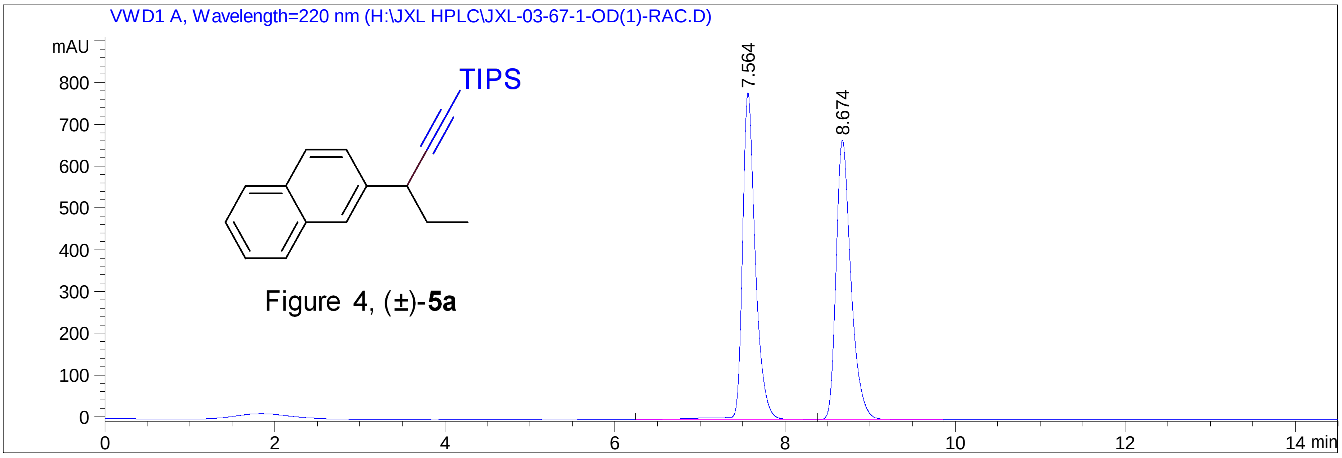

===

Area Percent Report

===

Sorted By : Signal
Multiplier : 1.0000
Dilution : 1.0000
Do not use Multiplier & Dilution Factor with ISTDs

Signal 1: VWD1 A, Wavelength=220 nm

Peak #	RetTime [min]	Type	Width [min]	Area [mAU*s]	Height [mAU]	Area %
1	7.564	VB R	0.1577	8214.19043	781.32666	50.3793
2	8.674	BB	0.1833	8090.48779	667.96808	49.6207

Totals : 1.63047e4 1449.29474

===

*** End of Report ***

Supplementary Fig. 215. HPLC spectra for compound (±)-**5a**

Data File H:\JXL HPLC\JXL-03-87-1-OD(1)-EE.D
Sample Name: JXL-03-87-1-OD(1)

===
Acq. Operator : 系统 Seq. Line : 14
Acq. Instrument : HPLC-1260 Location : 71
Injection Date : 8/13/2020 12:53:42 AM Inj : 2
Inj Volume : 3.000 µl
Different Inj Volume from Sample Entry! Actual Inj Volume : 0.300 µl
Acq. Method : D:\JXL\20200812\YH 2020-08-12 19-08-25\0IPA-15-0.8-1-2-220-JXL.M
Last changed : 8/12/2020 11:00:47 PM by 系统
Analysis Method : E:\DATA\20201027\LC 2020-12-15 14-02-17\6IPA_10_0.8_3.M (Sequence Method)
Last changed : 12/15/2020 7:33:03 PM by SYSTEM
(modified after loading)

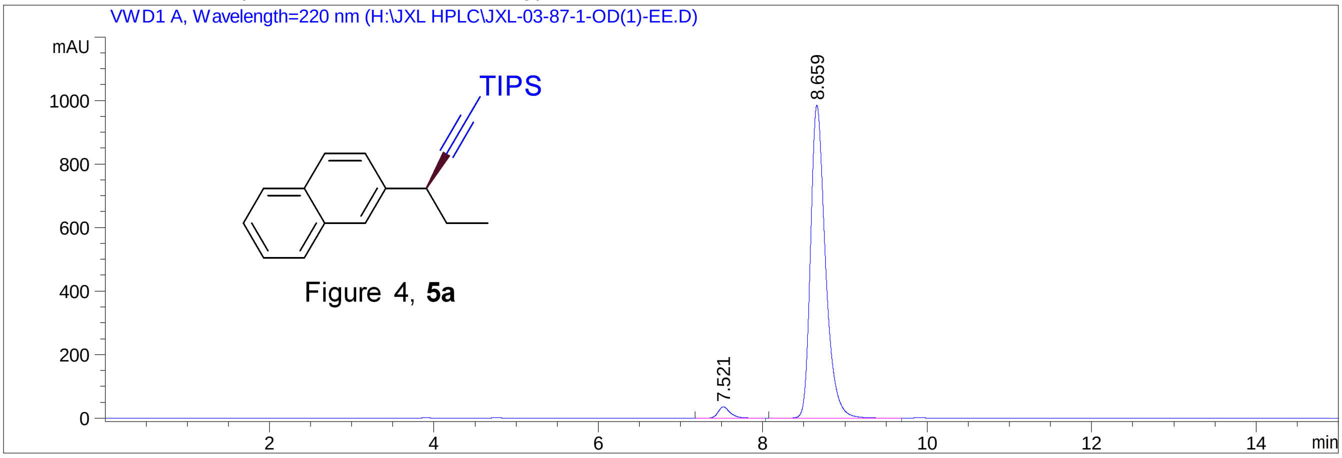

===
Area Percent Report
===

Sorted By : Signal
Multiplier : 1.0000
Dilution : 1.0000
Do not use Multiplier & Dilution Factor with ISTDs

Signal 1: VWD1 A, Wavelength=220 nm

Peak #	RetTime [min]	Type	Width [min]	Area [mAU*s]	Height [mAU]	Area %
1	7.521	BB	0.1554	368.12753	35.80718	2.8803
2	8.659	BB	0.1909	1.24129e4	985.56152	97.1197

Totals : 1.27811e4 1021.36870

===
*** End of Report ***

Supplementary Fig. 216. HPLC spectra for compound **5a**

Data File H:\JXL HPLC\JXL-03-52-1-OD(1)-RAC.D
Sample Name: JXL-03-52-1-OD(1)

===

Acq. Operator : 系统 Seq. Line : 4
Acq. Instrument : HPLC-1260 Location : 91
Injection Date : 7/29/2020 2:43:42 PM Inj : 2
Inj Volume : 3.000 µl
Different Inj Volume from Sample Entry! Actual Inj Volume : 0.300 µl
Acq. Method : D:\JXL\20200729\YH 2020-07-29 13-10-22\0IPA-40-0.8-1-2-220-JXL.M
Last changed : 7/29/2020 3:00:14 PM by 系统
(modified after loading)
Analysis Method : E:\DATA\20201027\LC 2020-12-15 14-02-17\6IPA_10_0.8_3.M (Sequence Method)
Last changed : 12/15/2020 7:34:31 PM by SYSTEM
(modified after loading)
Additional Info : Peak(s) manually integrated

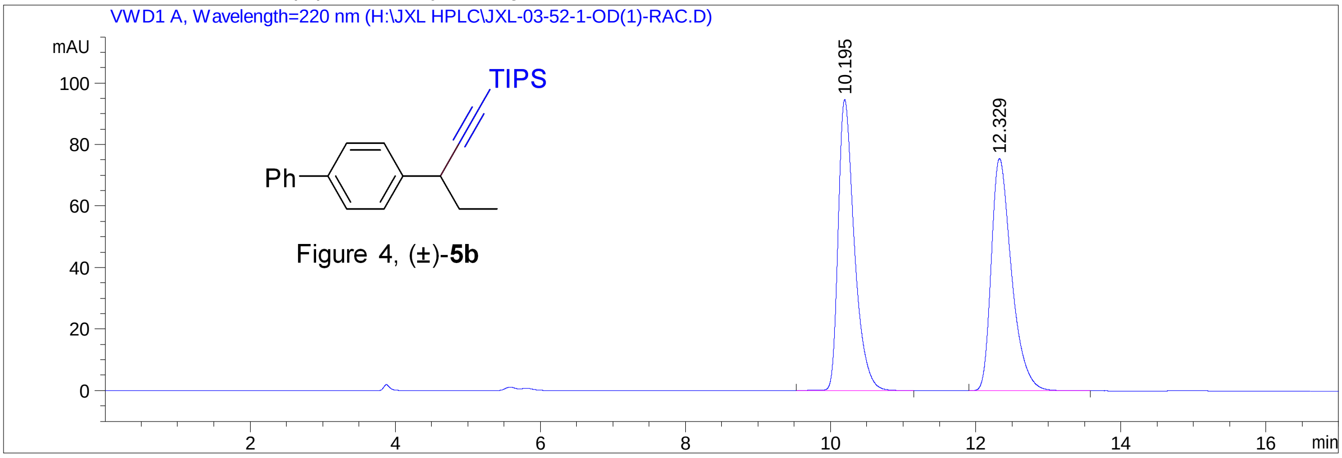

===

Area Percent Report

===

Sorted By : Signal
Multiplier : 1.0000
Dilution : 1.0000
Do not use Multiplier & Dilution Factor with ISTDs

Signal 1: VWD1 A, Wavelength=220 nm

Peak #	RetTime [min]	Type	Width [min]	Area [mAU*s]	Height [mAU]	Area %
1	10.195	BB	0.2340	1469.57141	94.70167	49.9464
2	12.329	BB	0.2973	1472.72339	75.57758	50.0536

Totals : 2942.29480 170.27924

===

*** End of Report ***

Supplementary Fig. 217. HPLC spectra for compound (±)-**5b**

Data File H:\JXL HPLC\JXL-03-90-1-OD(1)-EE.D
Sample Name: JXL-03-90-1-OD(1)

===
Acq. Operator : 系统 Seq. Line : 5
Acq. Instrument : HPLC-1260 Location : 81
Injection Date : 8/14/2020 1:17:53 AM Inj : 1
Inj Volume : 3.000 µl
Different Inj Volume from Sample Entry! Actual Inj Volume : 0.200 µl
Acq. Method : D:\JXL\20200813\YH 2020-08-13 23-35-04\0IPA-20-0.8-1-6-220-JXL.M
Last changed : 8/13/2020 11:36:14 PM by 系统
Analysis Method : E:\DATA\20201027\LC 2020-12-15 14-02-17\6IPA_10_0.8_3.M (Sequence Method)
Last changed : 12/15/2020 7:42:23 PM by SYSTEM
(modified after loading)
Additional Info : Peak(s) manually integrated

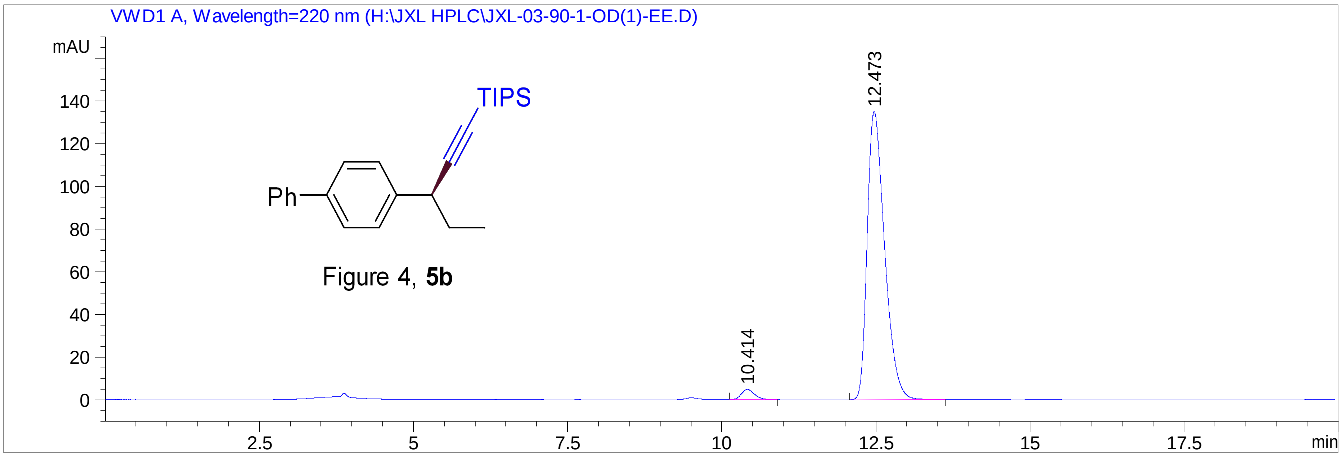

===
Area Percent Report
===

Sorted By : Signal
Multiplier : 1.0000
Dilution : 1.0000
Do not use Multiplier & Dilution Factor with ISTDs

Signal 1: VWD1 A, Wavelength=220 nm

Peak #	RetTime [min]	Type	Width [min]	Area [mAU*s]	Height [mAU]	Area %
1	10.414	BB	0.2247	71.15249	4.80578	2.6157
2	12.473	BB	0.3011	2649.05762	134.92877	97.3843

Totals : 2720.21011 139.73455

===
*** End of Report ***

Supplementary Fig. 218. HPLC spectra for compound **5b**

Data File H:\JXL HPLC\JXL-03-92-1-OD(2)-RAC.D
Sample Name: JXL-03-92-1-OD(2)

===

Acq. Operator : 系统 Seq. Line : 11
Acq. Instrument : HPLC-1260 Location : 85
Injection Date : 8/17/2020 3:32:40 PM Inj : 1
Inj Volume : 3.000 µl
Different Inj Volume from Sample Entry! Actual Inj Volume : 0.200 µl
Acq. Method : D:\JXL\20200817\YH 2020-08-17 08-55-41\0IPA-40-0.8-1-2-220-JXL.M
Last changed : 8/17/2020 3:53:54 PM by 系统
(modified after loading)
Analysis Method : C:\CHEM32\1\METHODS\15IPA_30_8_4.M
Last changed : 12/18/2020 9:34:33 PM by SYSTEM
(modified after loading)

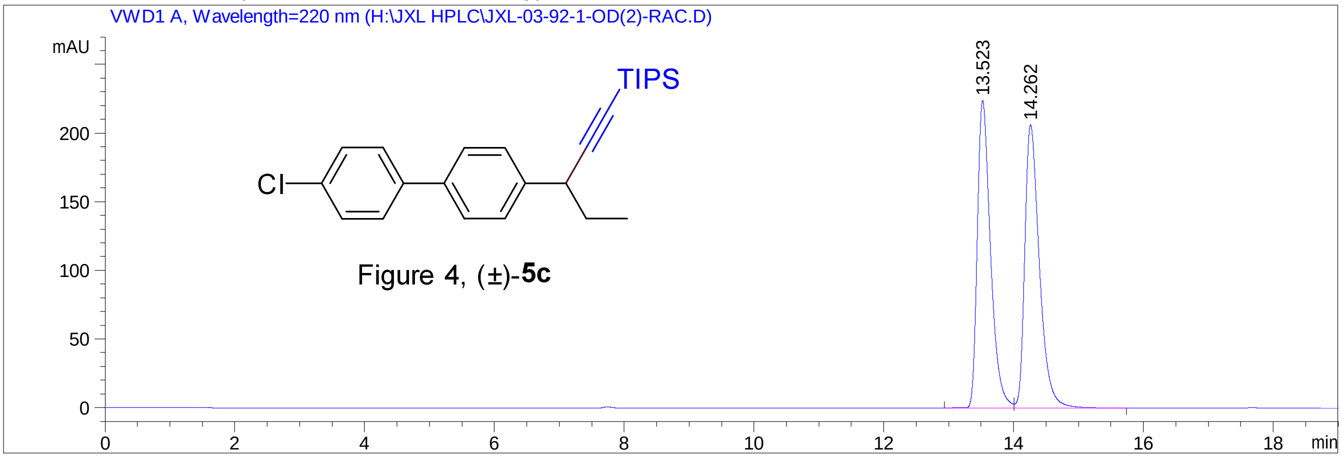

===

Area Percent Report

===

Sorted By : Signal
Multiplier : 1.0000
Dilution : 1.0000
Do not use Multiplier & Dilution Factor with ISTDs

Signal 1: VWD1 A, Wavelength=220 nm

Peak #	RetTime [min]	Type	Width [min]	Area [mAU*s]	Height [mAU]	Area %
1	13.523	VV R	0.2158	3176.07349	224.10931	49.4844
2	14.262	VB	0.2387	3242.26465	206.43098	50.5156

Totals : 6418.33813 430.54030

===

*** End of Report ***

HPLC1260 12/18/2020 9:34:42 PM SYSTEM Page 1 of 1

Supplementary Fig. 219. HPLC spectra for compound (±)-**5c**

Data File H:\JXL HPLC\JXL-03-118-1-OD(2)-EE.D
Sample Name: JXL-03-118-1-OD(2)

===

Acq. Operator : 系统 Seq. Line : 11
Acq. Instrument : HPLC-1260 Location : 83
Injection Date : 8/26/2020 1:43:16 PM Inj : 1
Inj Volume : 3.000 µl
Different Inj Volume from Sample Entry! Actual Inj Volume : 0.300 µl
Acq. Method : D:\JXL\20200826\YH 2020-08-26 09-02-52\0IPA-40-0.8-1-2-220-JXL.M
Last changed : 8/26/2020 1:59:25 PM by 系统
(modified after loading)
Analysis Method : E:\DATA\20201027\LC 2020-12-15 14-02-17\6IPA_10_0.8_3.M (Sequence Method)
Last changed : 12/15/2020 7:29:52 PM by SYSTEM
(modified after loading)

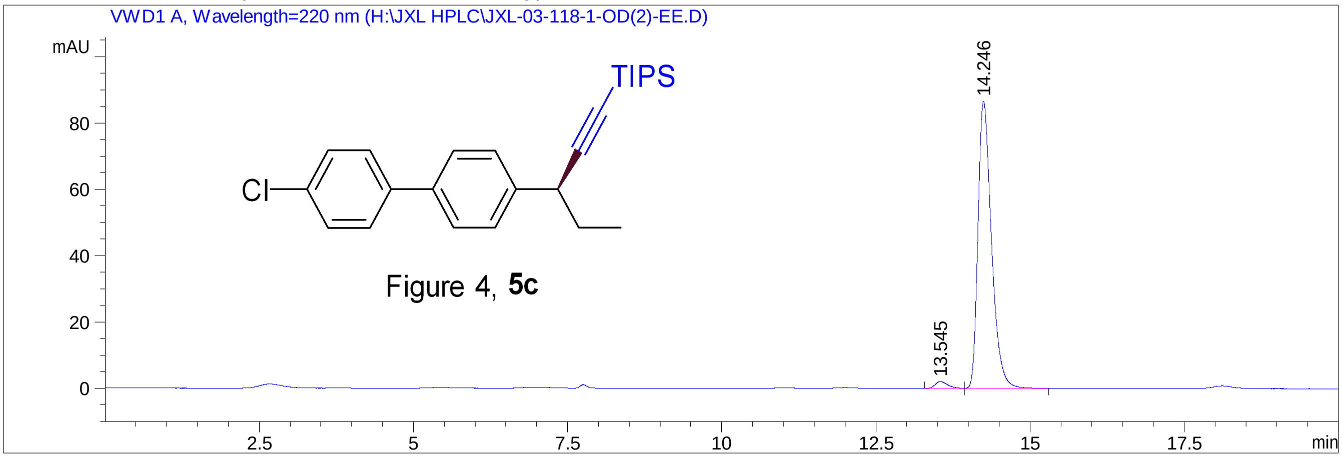

===

Area Percent Report

===

Sorted By : Signal
Multiplier : 1.0000
Dilution : 1.0000
Do not use Multiplier & Dilution Factor with ISTDs

Signal 1: VWD1 A, Wavelength=220 nm

Peak #	RetTime [min]	Type	Width [min]	Area [mAU*s]	Height [mAU]	Area %
1	13.545	BB	0.1861	29.73621	2.10822	2.2080
2	14.246	BB	0.2312	1317.04065	86.69881	97.7920

Totals : 1346.77686 88.80703

===

*** End of Report ***

Supplementary Fig. 220. HPLC spectra for compound **5c**

Data File H:\JXL HPLC XIUGAI\OnlineEdited--042.D
Sample Name: JXL-02-157-8-OD(2)

===

Acq. Operator : 系统 Seq. Line : 42
Acq. Instrument : HPLC-1260 Location : 77
Injection Date : 12/13/2020 8:10:48 PM Inj : 1
Inj Volume : 3.000 µl
Different Inj Volume from Sample Entry! Actual Inj Volume : 0.500 µl
Acq. Method : D:\G527\FZ\20201127\YH 2020-12-12 20-24-28\0IPA-30-0.5-1-6-220-JXL.M
Last changed : 12/13/2020 8:31:37 PM by 系统
(modified after loading)
Analysis Method : C:\CHEM32\1\METHODS\15IPA_30_8_4.M
Last changed : 12/18/2020 9:36:51 PM by SYSTEM
(modified after loading)
Additional Info : Peak(s) manually integrated

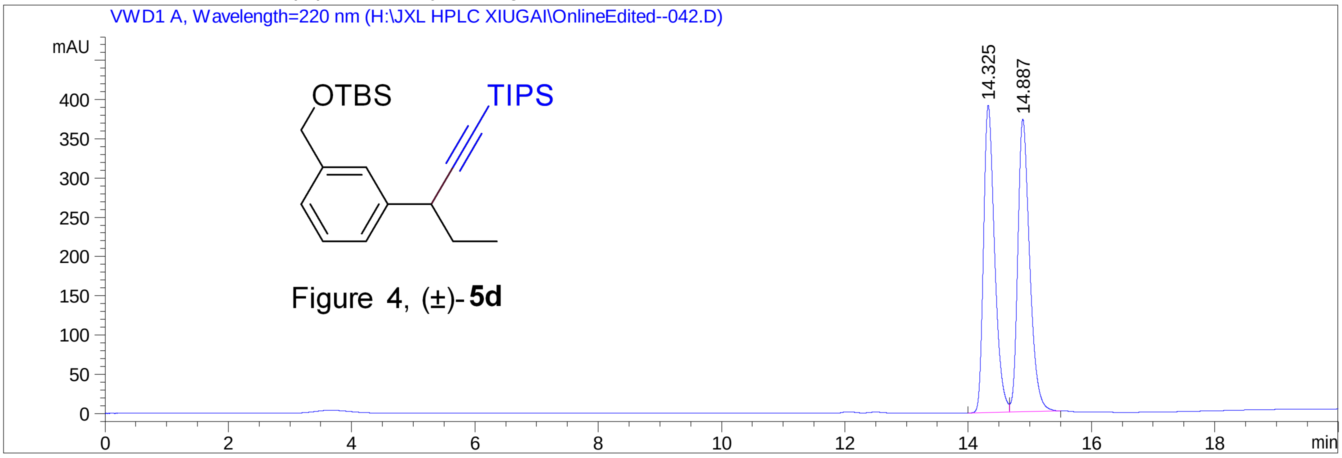

===

Area Percent Report

===

Sorted By : Signal
Multiplier : 1.0000
Dilution : 1.0000
Do not use Multiplier & Dilution Factor with ISTDs

Signal 1: VWD1 A, Wavelength=220 nm

Peak #	RetTime [min]	Type	Width [min]	Area [mAU*s]	Height [mAU]	Area %
1	14.325	BV	0.1908	4898.95654	392.01218	49.5196
2	14.887	VB	0.2036	4994.00635	373.24091	50.4804
Totals :				9892.96289	765.25308	

===

*** End of Report ***

Supplementary Fig. 221. HPLC spectra for compound (±)-**5d**

Data File H:\JXL HPLC XIUGAI\OnlineEdited--043.D
Sample Name: JXL-03-102-1-OD(2)

===

Acq. Operator : 系统 Seq. Line : 43
Acq. Instrument : HPLC-1260 Location : 78
Injection Date : 12/13/2020 8:33:02 PM Inj : 1
Inj Volume : 3.000 µl
Different Inj Volume from Sample Entry! Actual Inj Volume : 0.700 µl
Acq. Method : D:\G527\FZ\20201127\YH 2020-12-12 20-24-28\0IPA-30-0.5-1-6-220-JXL.M
Last changed : 12/13/2020 8:31:37 PM by 系统
Analysis Method : E:\DATA\20201027\LC 2020-12-16 12-53-00\8IPA_38_0.8_3.M (Sequence Method)
Last changed : 12/16/2020 9:48:22 PM by SYSTEM
(modified after loading)
Additional Info : Peak(s) manually integrated

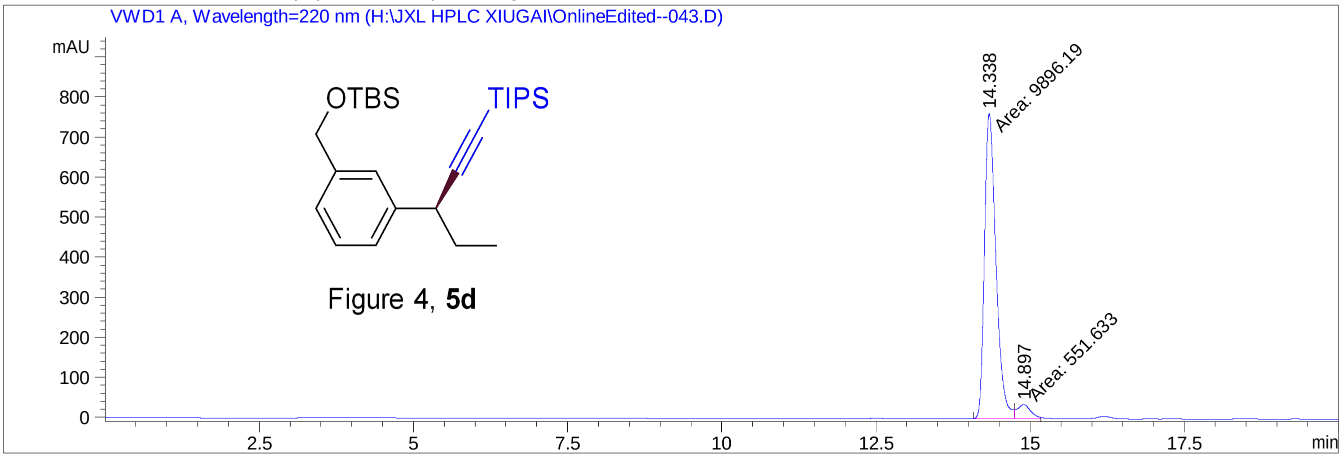

===

Area Percent Report

===

Sorted By : Signal
Multiplier : 1.0000
Dilution : 1.0000
Do not use Multiplier & Dilution Factor with ISTDs

Signal 1: VWD1 A, Wavelength=220 nm

Peak #	RetTime [min]	Type	Width [min]	Area [mAU*s]	Height [mAU]	Area %
1	14.338	FM	0.2162	9896.19434	763.04950	94.7201
2	14.897	MF	0.2551	551.63306	36.04580	5.2799

Totals : 1.04478e4 799.09530

===

*** End of Report ***

Supplementary Fig. 222. HPLC spectra for compound **5d**

Data File H:\JXL HPLC XIUGAI\OnlineEdited--223.D
Sample Name: JXL-02-132-OD(2)

===

Acq. Operator : 系统 Seq. Line : 223
Acq. Instrument : HPLC-1260 Location : 81
Injection Date : 12/17/2020 10:38:06 AM Inj : 1
Inj Volume : 3.000 µl
Different Inj Volume from Sample Entry! Actual Inj Volume : 2.000 µl
Acq. Method : D:\G527\FZ\20201127\YH 2020-12-12 20-24-28\0IPA-30-0.5-1-6-220-JXL.M
Last changed : 12/14/2020 9:39:45 AM by 系统
Analysis Method : E:\DATA\20201027\LC 2020-11-27 14-40-53\1EtOH-40-0.8-3-220-XYH.M (Sequence Method)
Last changed : 12/17/2020 11:25:28 PM by SYSTEM
(modified after loading)
Additional Info : Peak(s) manually integrated

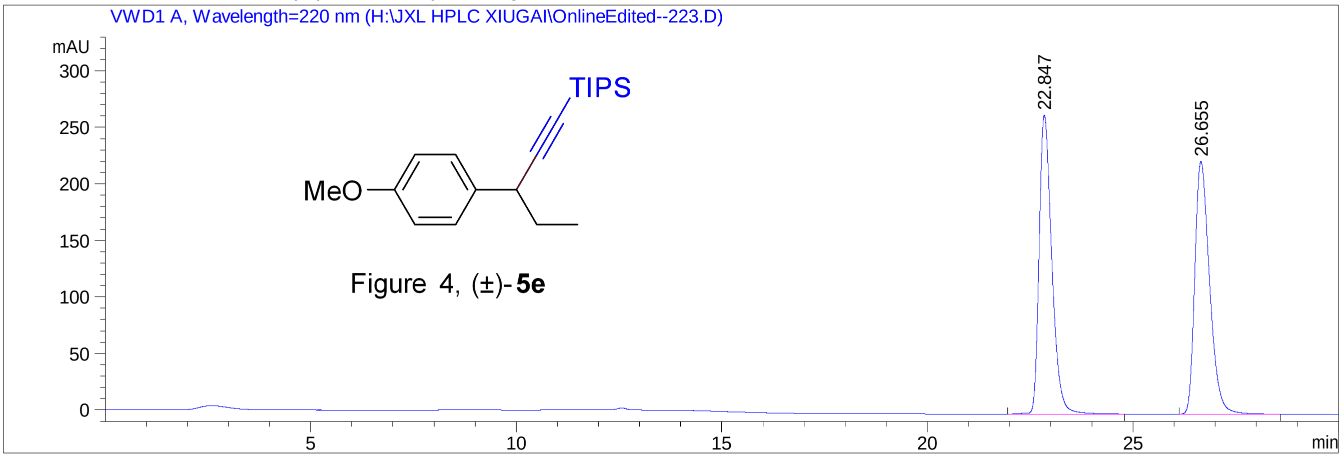

===
Area Percent Report
===

Sorted By : Signal
Multiplier : 1.0000
Dilution : 1.0000
Do not use Multiplier & Dilution Factor with ISTDs

Signal 1: VWD1 A, Wavelength=220 nm

Peak #	RetTime [min]	Type	Width [min]	Area [mAU*s]	Height [mAU]	Area %
1	22.847	BB	0.3280	5665.40869	264.39789	50.3487
2	26.655	BB	0.3807	5586.92920	223.48183	49.6513

Totals : 1.12523e4 487.87971

===
*** End of Report ***

Supplementary Fig. 223. HPLC spectra for compound (±)-**5e**

Data File H:\JXL HPLC XIUGAI\OnlineEdited--224.D
Sample Name: JXL-03-127-1-OD(2)

===

Acq. Operator : 系统 Seq. Line : 224
Acq. Instrument : HPLC-1260 Location : 79
Injection Date : 12/17/2020 11:09:29 AM Inj : 1
Inj Volume : 3.000 µl
Different Inj Volume from Sample Entry! Actual Inj Volume : 0.300 µl
Acq. Method : D:\G527\FZ\20201127\YH 2020-12-12 20-24-28\0IPA-30-0.5-1-6-220-JXL.M
Last changed : 12/14/2020 9:39:45 AM by 系统
Analysis Method : E:\DATA\20201027\LC 2020-11-27 14-40-53\1EtOH-40-0.8-3-220-XYH.M (Sequence Method)
Last changed : 12/17/2020 11:26:08 PM by SYSTEM
(modified after loading)

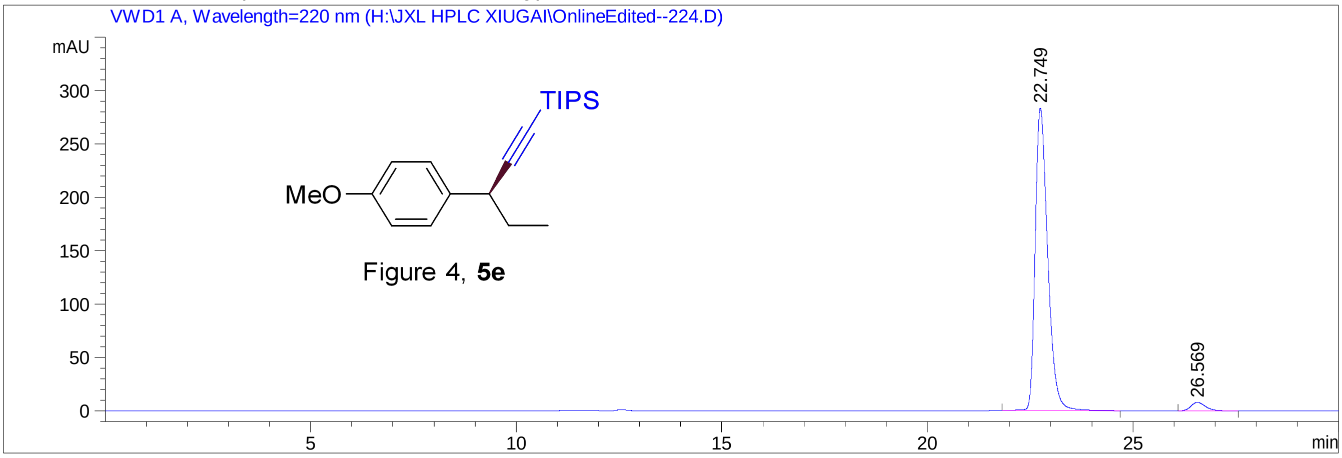

===

Area Percent Report

===

Sorted By : Signal
Multiplier : 1.0000
Dilution : 1.0000
Do not use Multiplier & Dilution Factor with ISTDs

Signal 1: VWD1 A, Wavelength=220 nm

Peak #	RetTime [min]	Type	Width [min]	Area [mAU*s]	Height [mAU]	Area %
1	22.749	BB	0.3245	5984.18018	283.18457	96.8689
2	26.569	BB	0.3349	193.42569	7.92140	3.1311

Totals : 6177.60587 291.10598

===

*** End of Report ***

Supplementary Fig. 224. HPLC spectra for compound **5e**

Data File H:\JXL HPLC\JXL-03-92-2-OD(2)-RAC.D
Sample Name: JXL-03-92-2-OD(2)

===

Acq. Operator : 系统 Seq. Line : 4
Acq. Instrument : HPLC-1260 Location : 81
Injection Date : 8/17/2020 10:26:59 AM Inj : 1
Inj Volume : 3.000 µl
Different Inj Volume from Sample Entry! Actual Inj Volume : 0.200 µl
Acq. Method : D:\JXL\20200817\YH 2020-08-17 08-55-41\0IPA-40-0.8-1-2-220-JXL.M
Last changed : 8/17/2020 10:49:51 AM by 系统
(modified after loading)
Analysis Method : C:\CHEM32\1\METHODS\15IPA_30_8_4.M
Last changed : 12/18/2020 9:38:07 PM by SYSTEM
(modified after loading)

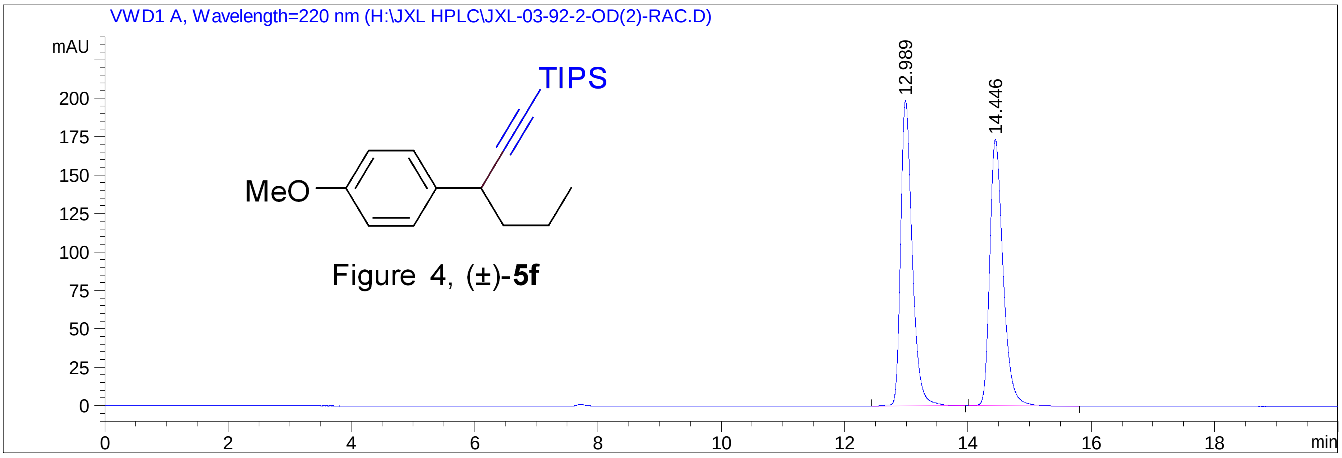

===

Area Percent Report

===

Sorted By : Signal
Multiplier : 1.0000
Dilution : 1.0000
Do not use Multiplier & Dilution Factor with ISTDs

Signal 1: VWD1 A, Wavelength=220 nm

Peak #	RetTime [min]	Type	Width [min]	Area [mAU*s]	Height [mAU]	Area %
1	12.989	BB	0.1981	2590.69507	198.68257	49.8213
2	14.446	BB	0.2291	2609.28149	173.36870	50.1787

Totals : 5199.97656 372.05127

===

*** End of Report ***

Supplementary Fig. 225. HPLC spectra for compound (±)-**5f**

Data File H:\JXL HPLC\JXL-03-105-1-OD(2)-EE.D
Sample Name: JXL-03-105-1-OD(2)

===

Acq. Operator : 系统 Seq. Line : 28
Acq. Instrument : HPLC-1260 Location : 95
Injection Date : 8/22/2020 12:54:36 AM Inj : 2
Inj Volume : 3.000 µl
Different Inj Volume from Sample Entry! Actual Inj Volume : 0.300 µl
Acq. Method : D:\YH\20200820\YH 2020-08-21 08-21-12\0IPA-20-0.8-1-6-220-JXL.M
Last changed : 8/21/2020 11:21:53 PM by 系统
Analysis Method : E:\DATA\20201027\LC 2020-12-15 14-02-17\6IPA_10_0.8_3.M (Sequence Method)
Last changed : 12/15/2020 8:19:17 PM by SYSTEM
(modified after loading)

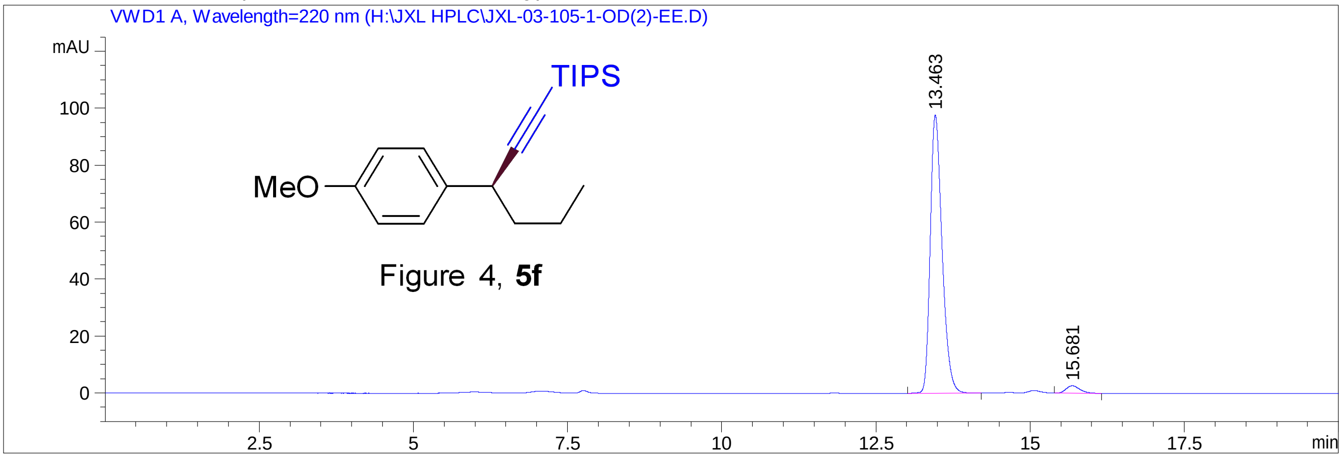

===

Area Percent Report

===

Sorted By : Signal
Multiplier : 1.0000
Dilution : 1.0000
Do not use Multiplier & Dilution Factor with ISTDs

Signal 1: VWD1 A, Wavelength=220 nm

Peak #	RetTime [min]	Type	Width [min]	Area [mAU*s]	Height [mAU]	Area %
1	13.463	BB	0.2036	1303.97400	97.74081	96.9901
2	15.681	BB	0.2027	40.46679	2.62005	3.0099

Totals : 1344.44079 100.36086

===

*** End of Report ***

Supplementary Fig. 226. HPLC spectra for compound **5f**

Data File H:\JXL HPLC XIUGAI\OnlineEdited--022.D
Sample Name: JXL-03-5-4-OD(2)

===

Acq. Operator : 系统 Seq. Line : 22
Acq. Instrument : HPLC-1260 Location : 81
Injection Date : 12/13/2020 5:23:48 AM Inj : 1
Inj Volume : 3.000 µl
Different Inj Volume from Sample Entry! Actual Inj Volume : 2.500 µl
Acq. Method : D:\G527\FZ\20201127\YH 2020-12-12 20-24-28\0IPA-30-0.8-1-6-220-JXL.M
Last changed : 12/13/2020 4:39:41 AM by 系统
Analysis Method : E:\DATA\20201027\LC 2020-11-24 17-29-49\1IPA-40-0.5-220-3-JXL.M (Sequence Method)
Last changed : 12/16/2020 11:20:01 AM by SYSTEM
(modified after loading)
Additional Info : Peak(s) manually integrated

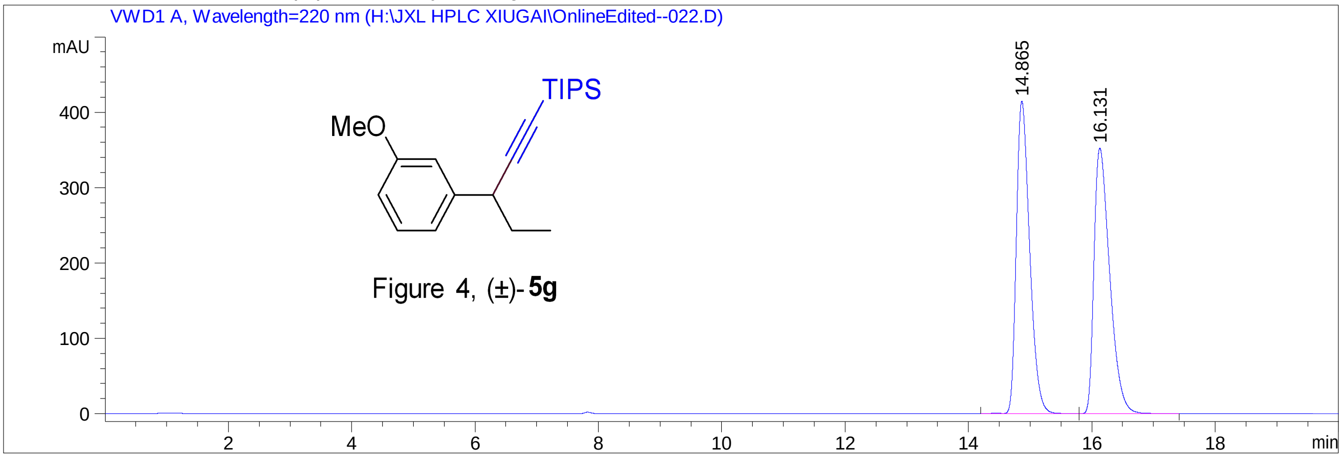

===

Area Percent Report

===

Sorted By : Signal
Multiplier : 1.0000
Dilution : 1.0000
Do not use Multiplier & Dilution Factor with ISTDs

Signal 1: VWD1 A, Wavelength=220 nm

Peak #	RetTime [min]	Type	Width [min]	Area [mAU*s]	Height [mAU]	Area %
1	14.865	VB R	0.2318	6233.67725	414.96182	49.9749
2	16.131	BB	0.2736	6239.94482	352.28775	50.0251

Totals : 1.24736e4 767.24957

===

*** End of Report ***

Supplementary Fig. 227. HPLC spectra for compound (±)-**5g**

Data File H:\JXL HPLC XIUGAI\OnlineEdited--021.D
Sample Name: JXL-03-93-3-OD(2)

===

Acq. Operator : 系统 Seq. Line : 21
Acq. Instrument : HPLC-1260 Location : 82
Injection Date : 12/13/2020 5:02:26 AM Inj : 1
Inj Volume : 3.000 µl
Different Inj Volume from Sample Entry! Actual Inj Volume : 1.000 µl
Acq. Method : D:\G527\FZ\20201127\YH 2020-12-12 20-24-28\0IPA-30-0.8-1-6-220-JXL.M
Last changed : 12/13/2020 4:39:41 AM by 系统
Analysis Method : E:\DATA\20201027\LC 2020-11-24 17-29-49\1IPA-40-0.5-220-3-JXL.M (Sequence Method)
Last changed : 12/16/2020 11:27:48 AM by SYSTEM
(modified after loading)
Additional Info : Peak(s) manually integrated

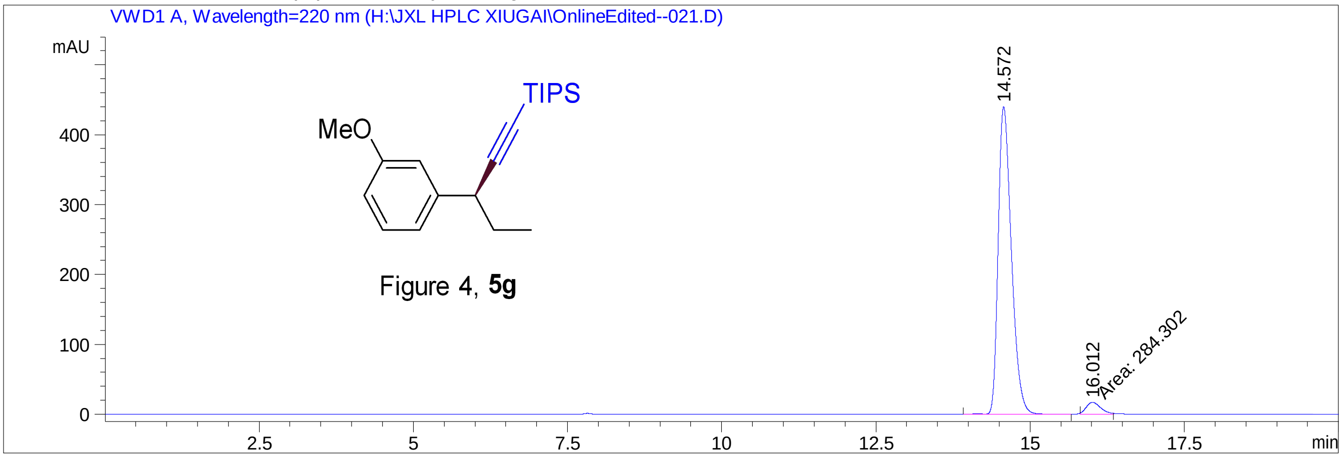

===

Area Percent Report

===

Sorted By : Signal
Multiplier : 1.0000
Dilution : 1.0000
Do not use Multiplier & Dilution Factor with ISTDs

Signal 1: VWD1 A, Wavelength=220 nm

Peak #	RetTime [min]	Type	Width [min]	Area [mAU*s]	Height [mAU]	Area %
1	14.572	VB R	0.2298	6559.40625	440.30060	95.8458
2	16.012	FM	0.2754	284.30161	17.20478	4.1542

Totals : 6843.70786 457.50538

===

*** End of Report ***

Supplementary Fig. 228. HPLC spectra for compound **5g**

Data File H:\JXL HPLC\JXL-03-157-1-OD(2)-RAC.D
Sample Name: JXL-03-157-1-OD(2)

===

Acq. Operator : 系统 Seq. Line : 165
Acq. Instrument : HPLC-1260 Location : 96
Injection Date : 9/18/2020 3:32:54 AM Inj : 2
Inj Volume : 3.000 µl
Different Inj Volume from Sample Entry! Actual Inj Volume : 0.300 µl
Acq. Method : D:\G527\FZ\20200822\YH 2020-09-15 11-31-01\0IPA-30-0.5-1-6-220-JXL.M
Last changed : 9/17/2020 11:59:29 PM by 系统
Analysis Method : E:\DATA\20201027\LC 2020-12-15 14-02-17\6IPA_10_0.8_3.M (Sequence Method)
Last changed : 12/15/2020 8:25:11 PM by SYSTEM
(modified after loading)
Additional Info : Peak(s) manually integrated

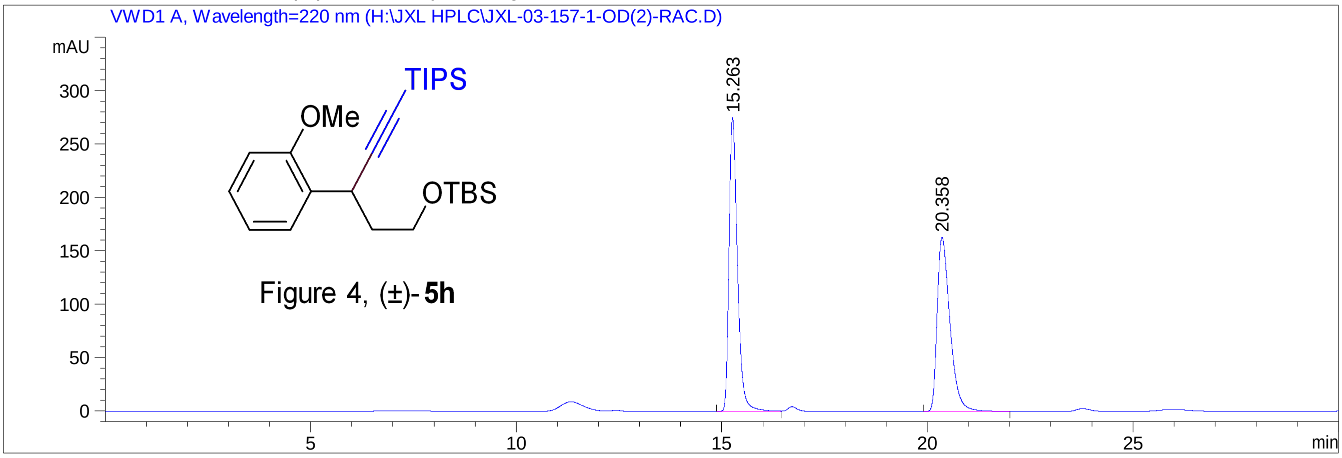

===

Area Percent Report

===

Sorted By : Signal
Multiplier : 1.0000
Dilution : 1.0000
Do not use Multiplier & Dilution Factor with ISTDs

Signal 1: VWD1 A, Wavelength=220 nm

Peak #	RetTime [min]	Type	Width [min]	Area [mAU*s]	Height [mAU]	Area %
1	15.263	BB	0.2178	3914.83374	275.44077	52.5555
2	20.358	BB	0.3282	3534.11255	163.15683	47.4445

Totals : 7448.94629 438.59760

===

*** End of Report ***

Supplementary Fig. 229. HPLC spectra for compound (±)-**5h**

Data File H:\JXL HPLC XIUGAI\OnlineEdited--039.D
Sample Name: JXL-04-22-5-OD(2)

===

Acq. Operator : 系统 Seq. Line : 39
Acq. Instrument : HPLC-1260 Location : 73
Injection Date : 10/30/2020 5:15:41 AM Inj : 1
Inj Volume : 3.000 µl
Different Inj Volume from Sample Entry! Actual Inj Volume : 0.300 µl
Acq. Method : D:\G527\FZ\20201026\YH 2020-10-29 08-35-49\0IPA-30-0.5-1-6-220-JXL.M
Last changed : 10/29/2020 6:19:06 PM by 系统
Analysis Method : E:\DATA\20201027\LC 2020-12-16 12-53-00\15IPA_30_0.8_4.M (Sequence Method)
Last changed : 12/16/2020 3:05:37 PM by SYSTEM
(modified after loading)
Additional Info : Peak(s) manually integrated

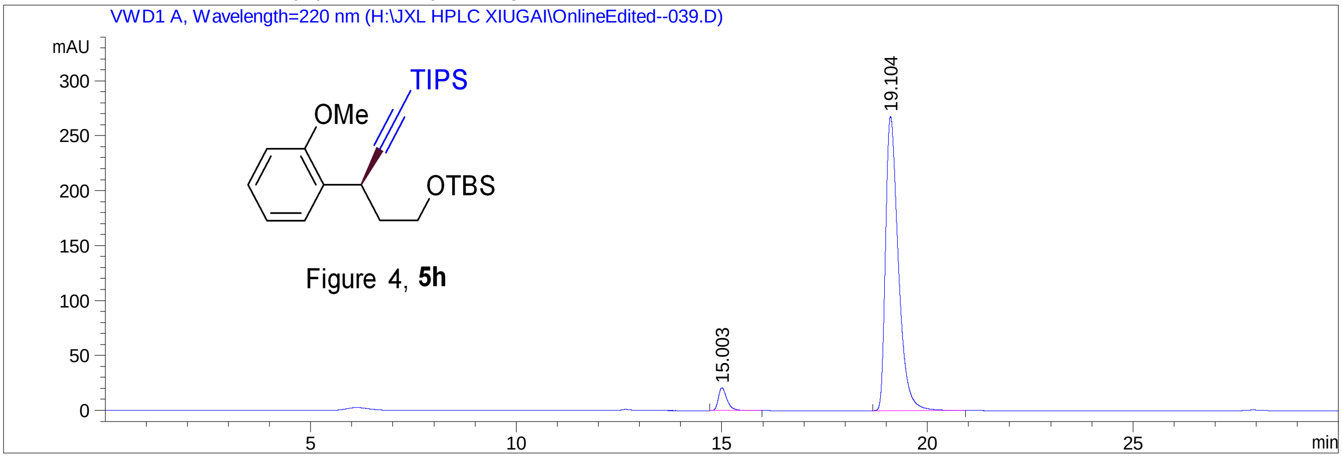

===

Area Percent Report

===

Sorted By : Signal
Multiplier : 1.0000
Dilution : 1.0000
Do not use Multiplier & Dilution Factor with ISTDs

Signal 1: VWD1 A, Wavelength=220 nm

Peak #	RetTime [min]	Type	Width [min]	Area [mAU*s]	Height [mAU]	Area %
1	15.003	BB	0.2210	303.79639	20.79279	4.9837
2	19.104	BB	0.3307	5791.94336	267.88892	95.0163

Totals : 6095.73975 288.68170

===

*** End of Report ***

Supplementary Fig. 230. HPLC spectra for compound **5h**

Data File H:\JXL HPLC XIUGAI\091-0402.D
Sample Name: JXL-03-44-1-OD(1)

===

Acq. Operator : 系统 Seq. Line : 4
Acq. Instrument : HPLC-1260 Location : 91
Injection Date : 12/12/2020 9:23:04 PM Inj : 1
Inj Volume : 3.000 µl
Different Inj Volume from Sample Entry! Actual Inj Volume : 2.000 µl
Acq. Method : D:\G527\FZ\20201127\YH 2020-12-12 20-24-28\1IPA-20-0.5-1-6-220-JXL.M
Last changed : 12/12/2020 9:21:20 PM by 系统
Analysis Method : E:\DATA\20201027\LC 2020-12-15 14-02-17\6IPA_10_0.8_3.M (Sequence Method)
Last changed : 12/15/2020 8:38:05 PM by SYSTEM
(modified after loading)
Additional Info : Peak(s) manually integrated

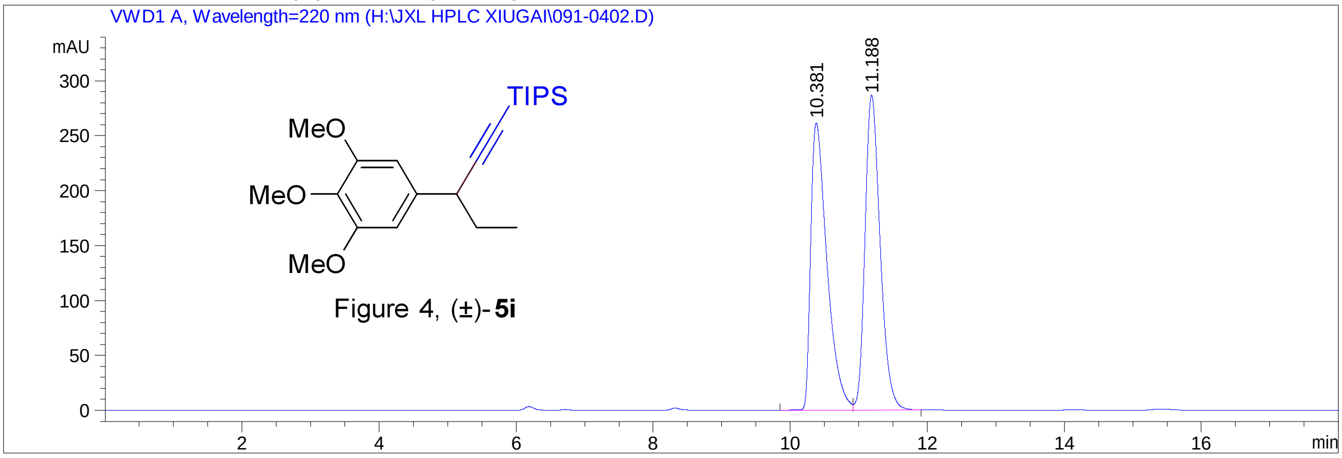

===

Area Percent Report

===

Sorted By : Signal
Multiplier : 1.0000
Dilution : 1.0000
Do not use Multiplier & Dilution Factor with ISTDs

Signal 1: VWD1 A, Wavelength=220 nm

Peak #	RetTime [min]	Type	Width [min]	Area [mAU*s]	Height [mAU]	Area %
1	10.381	VV R	0.2500	4336.35254	261.50546	49.5476
2	11.188	VB	0.2371	4415.54150	286.78824	50.4524

Totals : 8751.89404 548.29370

===

*** End of Report ***

Supplementary Fig. 231. HPLC spectra for compound (±)-**5i**

Data File H:\JXL HPLC XIUGAI\092-0501.D
Sample Name: JXL-03-96-1-OD(1)

===
Acq. Operator : 系统 Seq. Line : 5
Acq. Instrument : HPLC-1260 Location : 92
Injection Date : 12/12/2020 9:42:29 PM Inj : 1
Inj Volume : 3.000 µl
Different Inj Volume from Sample Entry! Actual Inj Volume : 0.800 µl
Acq. Method : D:\G527\FZ\20201127\YH 2020-12-12 20-24-28\1IPA-20-0.5-1-6-220-JXL.M
Last changed : 12/12/2020 10:04:54 PM by 系统
(modified after loading)
Analysis Method : C:\CHEM32\1\METHODS\15IPA_30_8_4.M
Last changed : 12/18/2020 9:40:44 PM by SYSTEM
(modified after loading)
Additional Info : Peak(s) manually integrated

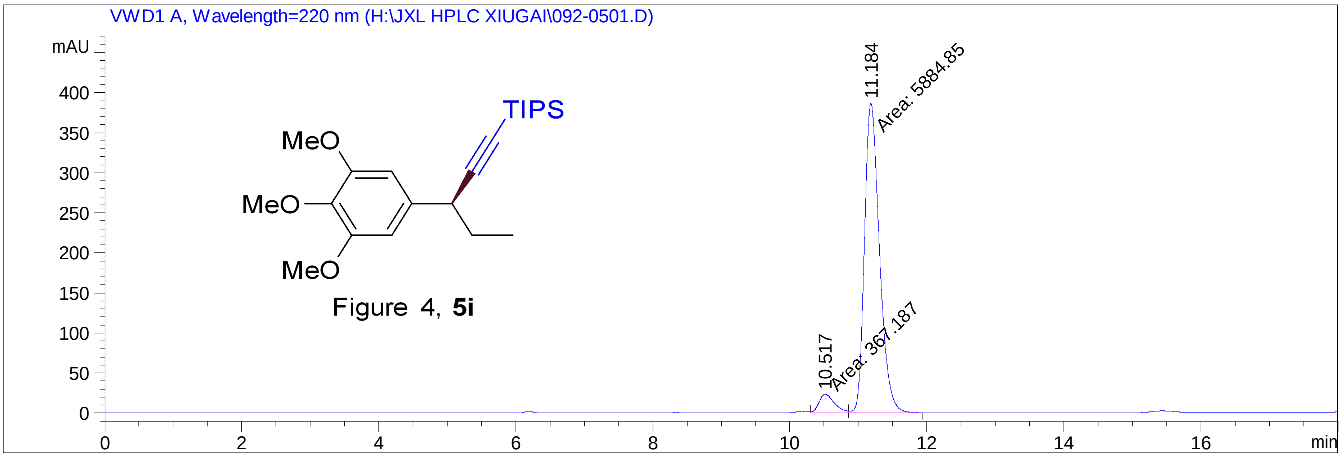

===
Area Percent Report
===

Sorted By : Signal
Multiplier : 1.0000
Dilution : 1.0000
Do not use Multiplier & Dilution Factor with ISTDs

Signal 1: VWD1 A, Wavelength=220 nm

Peak #	RetTime [min]	Type	Width [min]	Area [mAU*s]	Height [mAU]	Area %
1	10.517	MF	0.2625	367.18674	23.31229	5.8731
2	11.184	FM	0.2538	5884.84668	386.49524	94.1269

Totals : 6252.03342 409.80753

===
*** End of Report ***

Supplementary Fig. 232. HPLC spectra for compound **5i**

Data File H:\JXL HPLC\JXL-02-164-9-OD(2)-RAC.D
Sample Name: JXL-02-164-9-OD(2)

===

Acq. Operator : 系统 Seq. Line : 34
Acq. Instrument : HPLC-1260 Location : 81
Injection Date : 8/16/2020 6:01:09 AM Inj : 1
Inj Volume : 3.000 µl
Different Inj Volume from Sample Entry! Actual Inj Volume : 0.500 µl
Acq. Method : D:\G527\FZ\20200814\YH 2020-08-15 15-49-29\0IPA-40-0.3-1-2-220-JXL.M
Last changed : 8/15/2020 10:39:12 PM by 系统
Analysis Method : E:\DATA\20201027\LC 2020-12-15 14-02-17\6IPA_10_0.8_3.M (Sequence Method)
Last changed : 12/15/2020 8:43:43 PM by SYSTEM
(modified after loading)
Additional Info : Peak(s) manually integrated

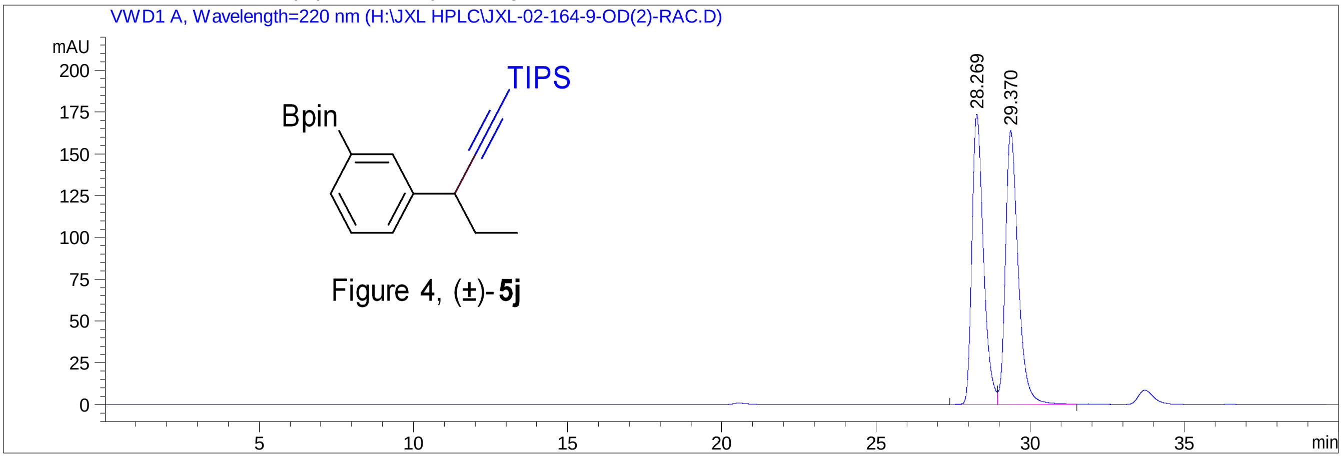

===

Area Percent Report

===

Sorted By : Signal
Multiplier : 1.0000
Dilution : 1.0000
Do not use Multiplier & Dilution Factor with ISTDs

Signal 1: VWD1 A, Wavelength=220 nm

Peak #	RetTime [min]	Type	Width [min]	Area [mAU*s]	Height [mAU]	Area %
1	28.269	BV	0.3981	4526.81055	173.69307	49.1311
2	29.370	VB	0.4353	4686.92578	163.81421	50.8689

Totals : 9213.73633 337.50728

===

*** End of Report ***

Supplementary Fig. 233. HPLC spectra for compound (±)-**5j**

Data File H:\JXL HPLC\JXL-03-95-1-OD(2)-EE.D
Sample Name: JXL-03-95-1-OD(2)

===

Acq. Operator : 系统 Seq. Line : 35
Acq. Instrument : HPLC-1260 Location : 85
Injection Date : 8/16/2020 6:42:35 AM Inj : 1
Inj Volume : 3.000 µl
Different Inj Volume from Sample Entry! Actual Inj Volume : 0.300 µl
Acq. Method : D:\G527\FZ\20200814\YH 2020-08-15 15-49-29\0IPA-40-0.3-1-2-220-JXL.M
Last changed : 8/15/2020 10:39:12 PM by 系统
Analysis Method : E:\DATA\20201027\LC 2020-12-15 14-02-17\6IPA_10_0.8_3.M (Sequence Method)
Last changed : 12/15/2020 8:44:39 PM by SYSTEM
(modified after loading)
Additional Info : Peak(s) manually integrated

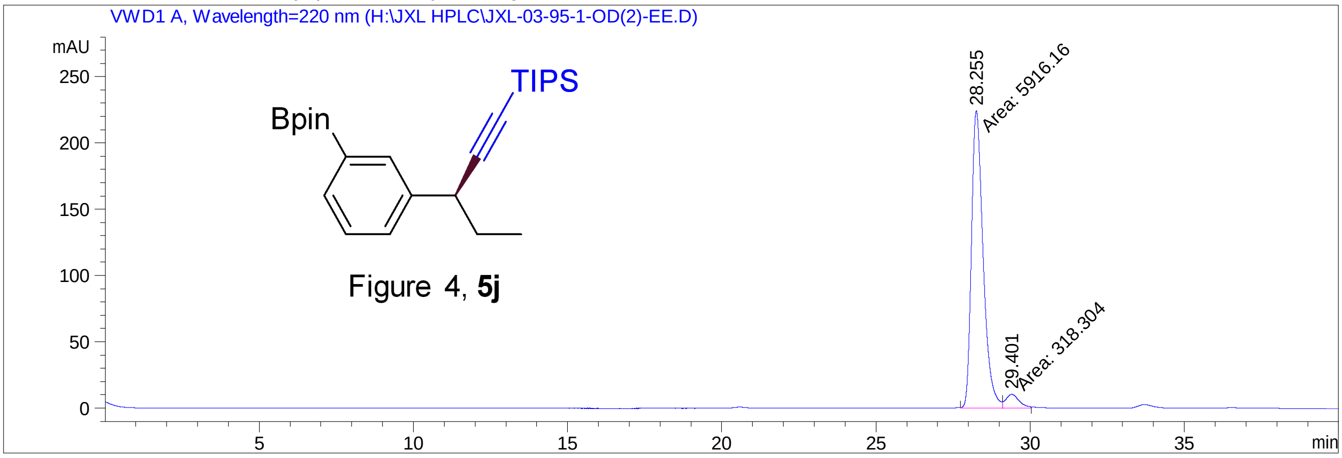

===

Area Percent Report

===

Sorted By : Signal
Multiplier : 1.0000
Dilution : 1.0000
Do not use Multiplier & Dilution Factor with ISTDs

Signal 1: VWD1 A, Wavelength=220 nm

Peak #	RetTime [min]	Type	Width [min]	Area [mAU*s]	Height [mAU]	Area %
1	28.255	MF	0.4394	5916.15869	224.40263	94.8945
2	29.401	MF	0.5088	318.30356	10.42563	5.1055

Totals : 6234.46225 234.82827

===

*** End of Report ***

Supplementary Fig. 234. HPLC spectra for compound **5j**

Data File H:\JXL HPLC\JXL-03-167-3-IE(1)-RAC.D
Sample Name: JXL-03-167-3-IE(1)

===

Acq. Operator : 系统 Seq. Line : 59
Acq. Instrument : HPLC-1260 Location : 84
Injection Date : 9/22/2020 6:09:20 PM Inj : 1
Inj Volume : 3.000 µl
Different Inj Volume from Sample Entry! Actual Inj Volume : 0.300 µl
Acq. Method : D:\G527\FZ\20200822\YH 2020-09-21 18-04-54\0IPA-35-0.3-1-6-220-JXL.M
Last changed : 9/22/2020 5:03:45 PM by 系统
Analysis Method : E:\DATA\20201027\LC 2020-12-15 14-02-17\6IPA_10_0.8_3.M (Sequence Method)
Last changed : 12/15/2020 8:47:09 PM by SYSTEM
(modified after loading)
Additional Info : Peak(s) manually integrated

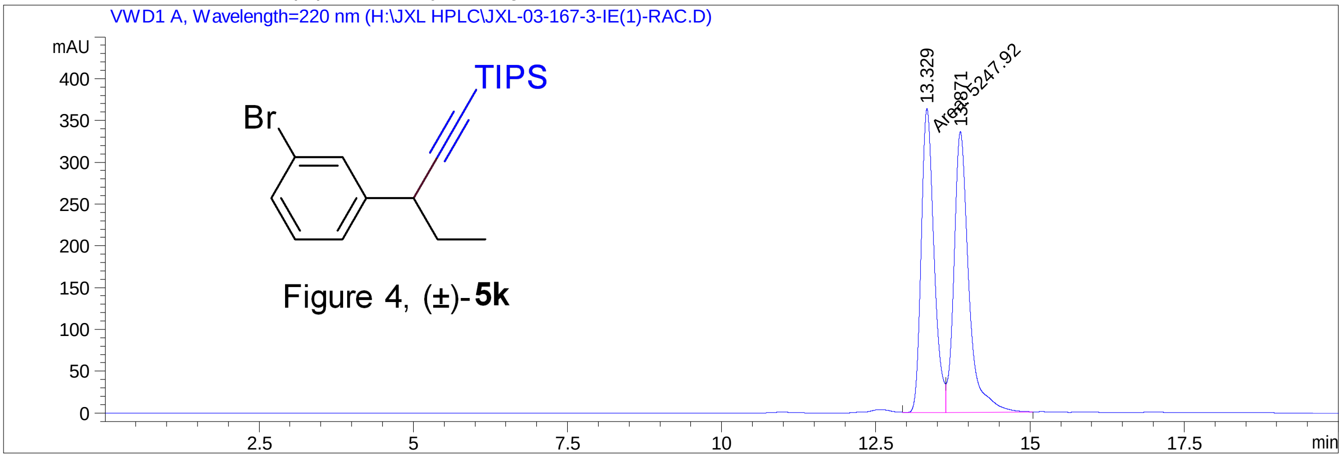

===

Area Percent Report

===

Sorted By : Signal
Multiplier : 1.0000
Dilution : 1.0000
Do not use Multiplier & Dilution Factor with ISTDs

Signal 1: VWD1 A, Wavelength=220 nm

Peak #	RetTime [min]	Type	Width [min]	Area [mAU*s]	Height [mAU]	Area %
1	13.329	FM	0.2407	5247.91748	363.43359	48.2757
2	13.871	VB	0.2509	5622.79443	335.64539	51.7243

Totals : 1.08707e4 699.07898

===

*** End of Report ***

Supplementary Fig. 235. HPLC spectra for compound (±)-**5k**

Data File H:\JXL HPLC\JXL-03-167-1-IE(1)-EE.D
Sample Name: JXL-03-167-1-IE(1)

===

Acq. Operator : 系统 Seq. Line : 56
Acq. Instrument : HPLC-1260 Location : 85
Injection Date : 9/22/2020 5:05:09 PM Inj : 1
Inj Volume : 3.000 µl
Different Inj Volume from Sample Entry! Actual Inj Volume : 0.500 µl
Acq. Method : D:\G527\FZ\20200822\YH 2020-09-21 18-04-54\0IPA-35-0.3-1-6-220-JXL.M
Last changed : 9/22/2020 5:03:45 PM by 系统
Analysis Method : E:\DATA\20201027\LC 2020-12-15 14-02-17\6IPA_10_0.8_3.M (Sequence Method)
Last changed : 12/15/2020 8:48:19 PM by SYSTEM
(modified after loading)
Additional Info : Peak(s) manually integrated

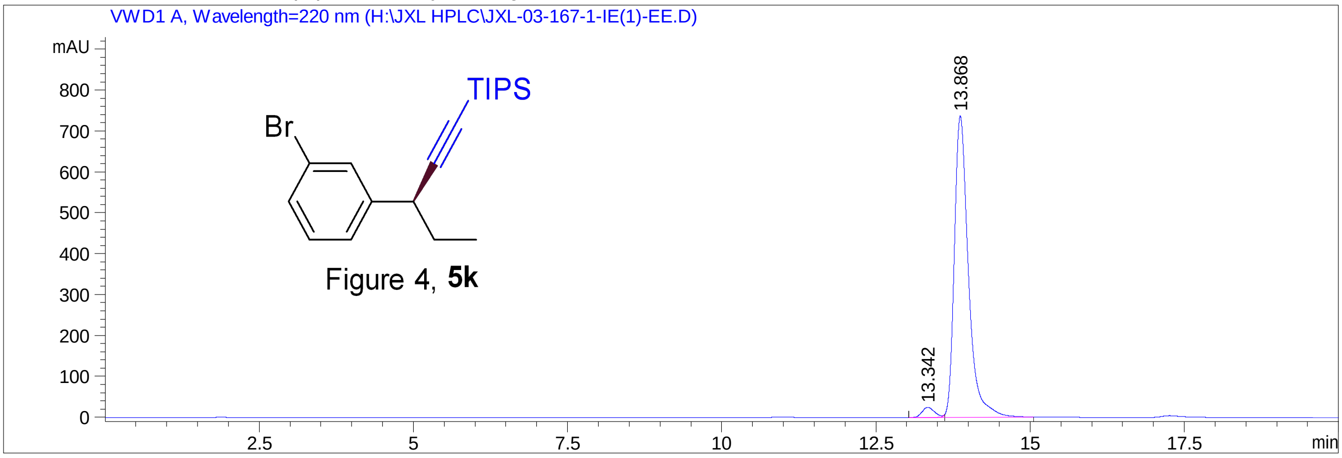

===

Area Percent Report

===

Sorted By : Signal
Multiplier : 1.0000
Dilution : 1.0000
Do not use Multiplier & Dilution Factor with ISTDs

Signal 1: VWD1 A, Wavelength=220 nm

Peak #	RetTime [min]	Type	Width [min]	Area [mAU*s]	Height [mAU]	Area %
1	13.342	BV E	0.2140	345.04163	24.92293	2.8670
2	13.868	VB R	0.2396	1.16897e4	736.77887	97.1330

Totals : 1.20347e4 761.70180

===

*** End of Report ***

Supplementary Fig. 236. HPLC spectra for compound **5k**

Data File H:\JXL HPLC XIUGAI\084-0301--004.D
Sample Name: JXL-03-92-5-OD(2)

===
Acq. Operator : 系统 Seq. Line : 4
Acq. Instrument : HPLC-1260 Location : 84
Injection Date : 8/17/2020 8:42:43 PM Inj : 1
Inj Volume : 3.000 µl
Different Inj Volume from Sample Entry! Actual Inj Volume : 2.500 µl
Acq. Method : D:\JXL\20200817\YH 2020-08-17 19-27-56\0IPA-40-0.5-1-2-220-JXL.M
Last changed : 8/17/2020 9:37:40 PM by 系统
(modified after loading)
Analysis Method : E:\DATA\20201027\LC 2020-12-28 19-00-53\20IPA_20_10_3.M (Sequence Method)
Last changed : 12/29/2020 3:26:14 PM by SYSTEM
(modified after loading)
Additional Info : Peak(s) manually integrated

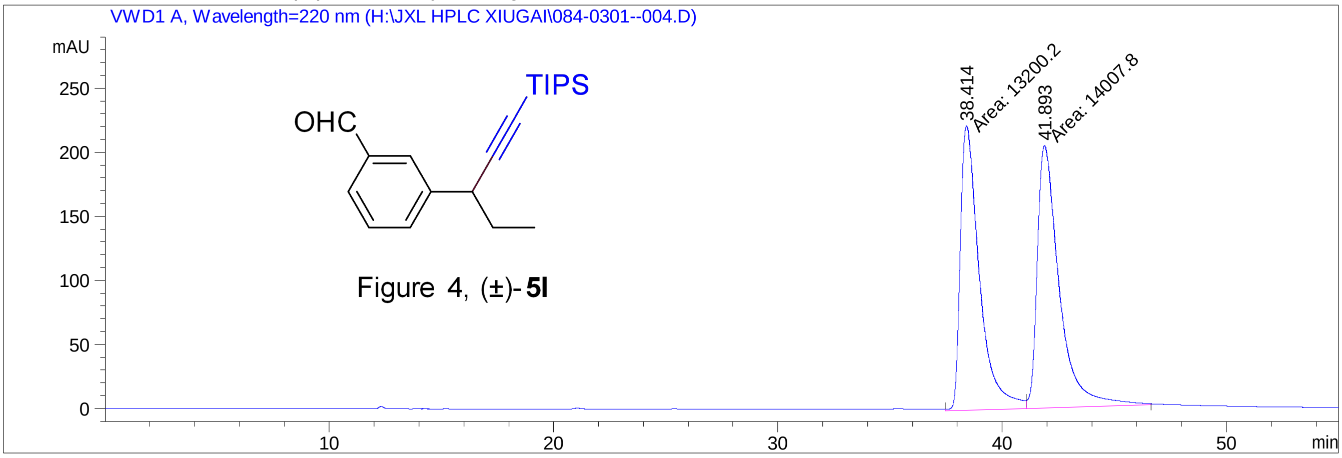

===
Area Percent Report
===

Sorted By : Signal
Multiplier : 1.0000
Dilution : 1.0000
Do not use Multiplier & Dilution Factor with ISTDs

Signal 1: VWD1 A, Wavelength=220 nm

Peak #	RetTime [min]	Type	Width [min]	Area [mAU*s]	Height [mAU]	Area %
1	38.414	MF	0.9917	1.32002e4	221.85463	48.5158
2	41.893	FM	1.1406	1.40078e4	204.67603	51.4842

Totals : 2.72080e4 426.53065

===
*** End of Report ***

Supplementary Fig. 237. HPLC spectra for compound (±)-**5l**

Data File H:\JXL HPLC XIUGAI\OnlineEdited--228.D
Sample Name: JXL-04-13-1-OD(2)

===
Acq. Operator   : 系统   Seq. Line : 228
Acq. Instrument : HPLC-1260   Location :   65
Injection Date : 12/17/2020 12:59:39 PM   Inj :   1
                                               Inj Volume : 3.000 µl
Different Inj Volume from Sample Entry! Actual Inj Volume : 4.000 µl
Acq. Method   : D:\G527\FZ\20201127\YH 2020-12-12 20-24-28\0IPA-75-0.5-1-6-220-JXL.M
Last changed   : 12/16/2020 10:14:40 PM by 系统
Analysis Method : C:\CHEM32\1\METHODS\10IPA_35_0.8_254_1.M
Last changed   : 12/29/2020 3:32:19 PM by SYSTEM
                  (modified after loading)
Additional Info : Peak(s) manually integrated

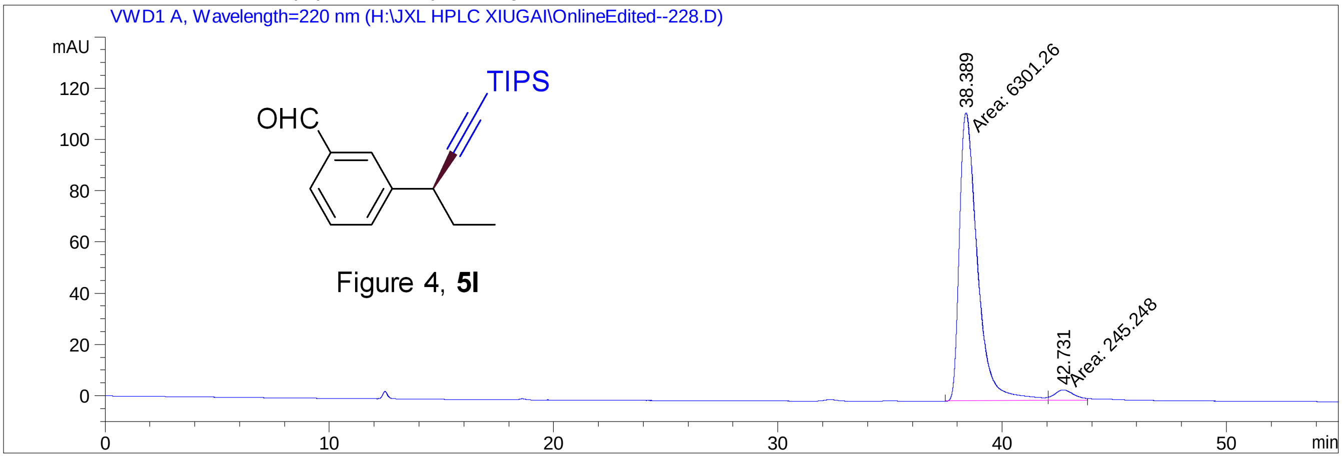

===
Area Percent Report
===

Sorted By   :   Signal
Multiplier   :   1.0000
Dilution   :   1.0000
Do not use Multiplier & Dilution Factor with ISTDs

Signal 1: VWD1 A, Wavelength=220 nm

Peak #	RetTime [min]	Type	Width [min]	Area [mAU*s]	Height [mAU]	Area %
1	38.389	MF	0.9346	6301.26416	112.37509	96.2538
2	42.731	MF	1.0123	245.24841	4.03794	3.7462

Totals :   6546.51257 116.41303

===
*** End of Report ***

Supplementary Fig. 238. HPLC spectra for compound **5l**

Data File H:\JXL HPLC XIUGAI\OnlineEdited--008.D
Sample Name: JXL-03-156-2-OD(2)

===

Acq. Operator : 系统 Seq. Line : 8
Acq. Instrument : HPLC-1260 Location : 93
Injection Date : 9/5/2020 12:26:45 PM Inj : 2
Inj Volume : 3.000 µl
Different Inj Volume from Sample Entry! Actual Inj Volume : 0.600 µl
Acq. Method : D:\JXL\20200905\YH 2020-09-05 08-53-26\0IPA-50-0.5-1-6-220-JXL.M
Last changed : 9/5/2020 11:44:07 AM by 系统
Analysis Method : E:\DATA\20201027\LC 2020-12-16 12-53-00\15IPA_30_0.8_4.M (Sequence Method)
Last changed : 12/16/2020 3:20:04 PM by SYSTEM
(modified after loading)

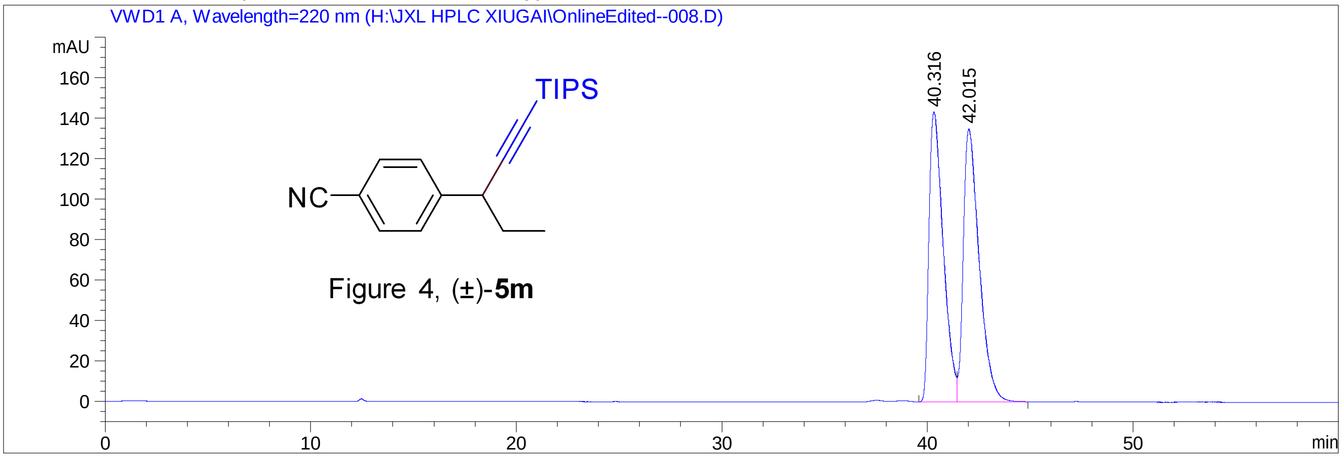

===

Area Percent Report

===

Sorted By : Signal
Multiplier : 1.0000
Dilution : 1.0000
Do not use Multiplier & Dilution Factor with ISTDs

Signal 1: VWD1 A, Wavelength=220 nm

Peak #	RetTime [min]	Type	Width [min]	Area [mAU*s]	Height [mAU]	Area %
1	40.316	BV	0.7029	6916.47607	143.22227	48.8690
2	42.015	VB	0.7842	7236.61133	134.82007	51.1310

Totals : 1.41531e4 278.04234

===

*** End of Report ***

Supplementary Fig. 239. HPLC spectra for compound (±)-**5m**

```
Data File H:\JXL HPLC\JXL-03-140-3-OD(2)-EE.D
Sample Name: JXL-03-140-3-OD(2)

=====================================================================
Acq. Operator   : 系统                          Seq. Line :  77
Acq. Instrument : HPLC-1260                      Location :   97
Injection Date  : 9/7/2020 3:28:25 PM                 Inj :   1
                                             Inj Volume : 3.000 µl
Different Inj Volume from Sample Entry!  Actual Inj Volume : 0.300 µl
Acq. Method     : D:\zy\20200906\YH 2020-09-06 12-21-16\0IPA-50-0.5-1-6-220-JXL.M
Last changed    : 9/7/2020 4:35:33 PM by 系统
                  (modified after loading)
Analysis Method : E:\DATA\20201027\LC 2020-12-16 12-53-00\15IPA_30_0.8_4.M (Sequence Method)
Last changed    : 12/16/2020 3:14:40 PM by SYSTEM
                  (modified after loading)
```

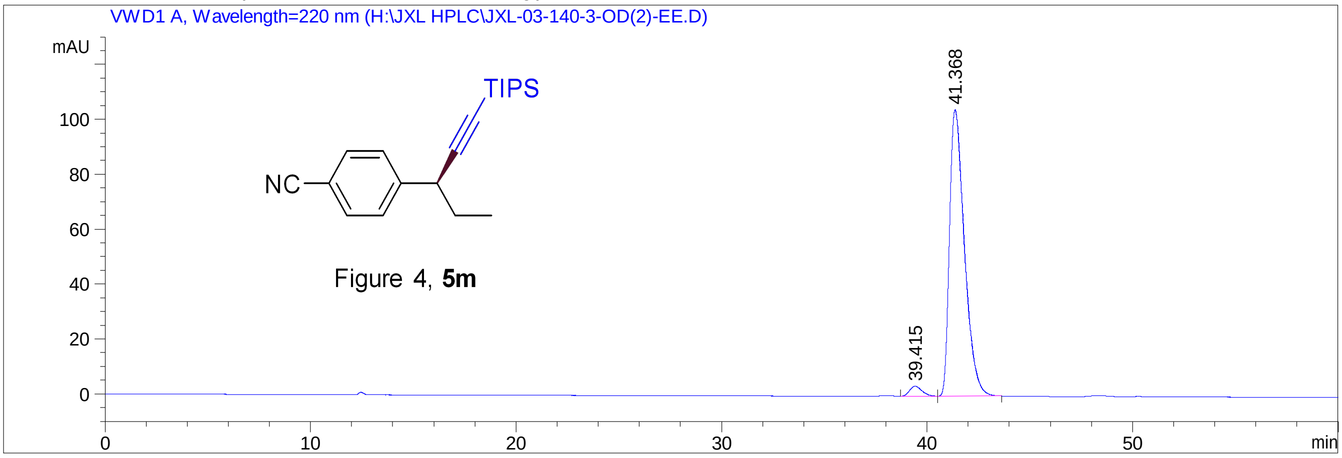

===

Area Percent Report

===

```
Sorted By             :      Signal
Multiplier            :      1.0000
Dilution              :      1.0000
Do not use Multiplier & Dilution Factor with ISTDs


Signal 1: VWD1 A, Wavelength=220 nm
```

Peak #	RetTime [min]	Type	Width [min]	Area [mAU*s]	Height [mAU]	Area %
1	39.415	BB	0.4882	151.74007	3.64863	2.8049
2	41.368	BB	0.7510	5258.08691	104.41154	97.1951

Totals : 5409.82698 108.06016

===

*** End of Report ***

Supplementary Fig. 240. HPLC spectra for compound **5m**

Data File H:\JXL HPLC XIUGAI\OnlineEdited--020.D
Sample Name: JXL-03-44-2-AD(1)

===

Acq. Operator : 系统 Seq. Line : 20
Acq. Instrument : HPLC-1260 Location : 81
Injection Date : 12/12/2020 4:57:42 PM Inj : 1
Inj Volume : 3.000 µl
Different Inj Volume from Sample Entry! Actual Inj Volume : 2.000 µl
Acq. Method : D:\G527\FZ\20201127\YH 2020-12-12 09-12-36\0IPA-40-0.8-1-2-220-JXL.M
Last changed : 12/12/2020 5:33:13 PM by 系统
(modified after loading)
Analysis Method : E:\DATA\20201027\LC 2020-12-15 14-02-17\6IPA_10_0.8_3.M (Sequence Method)
Last changed : 12/15/2020 8:50:28 PM by SYSTEM
(modified after loading)
Additional Info : Peak(s) manually integrated

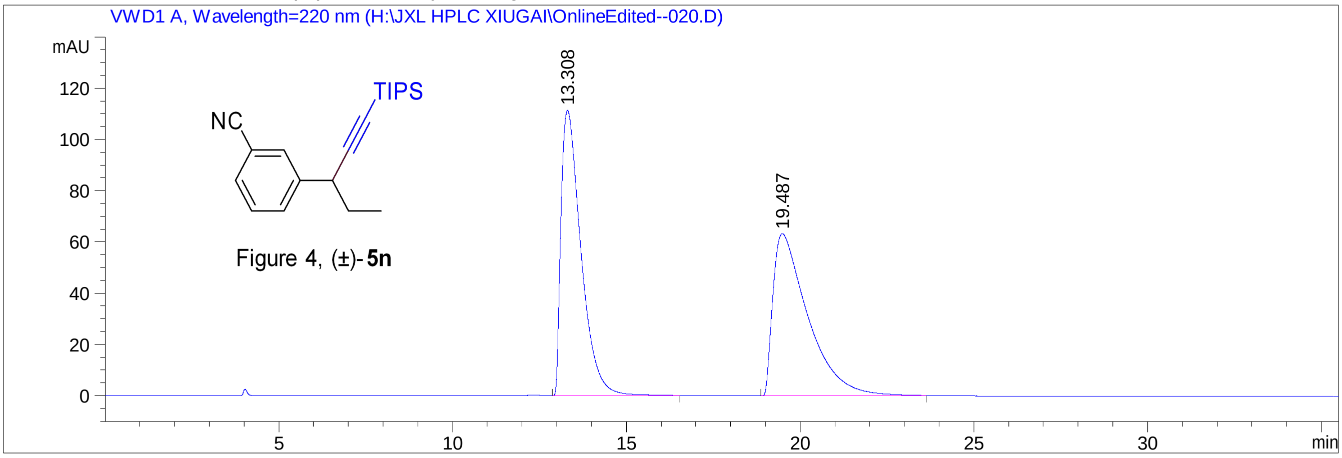

===

Area Percent Report

===

Sorted By : Signal
Multiplier : 1.0000
Dilution : 1.0000
Do not use Multiplier & Dilution Factor with ISTDs

Signal 1: VWD1 A, Wavelength=220 nm

Peak #	RetTime [min]	Type	Width [min]	Area [mAU*s]	Height [mAU]	Area %
1	13.308	BB	0.6101	4459.22852	111.38471	50.2351
2	19.487	BB	0.9239	4417.49512	63.20845	49.7649
Totals :				8876.72363	174.59316	

===

*** End of Report ***

Supplementary Fig. 241. HPLC spectra for compound (±)-**5n**

Data File H:\JXL HPLC XIUGAI\OnlineEdited--021.D
Sample Name: JXL-03-119-AD(1)

===

Acq. Operator : 系统 Seq. Line : 21
Acq. Instrument : HPLC-1260 Location : 82
Injection Date : 12/12/2020 5:34:37 PM Inj : 1
Inj Volume : 3.000 µl
Acq. Method : D:\G527\FZ\20201127\YH 2020-12-12 09-12-36\0IPA-40-0.8-1-2-220-JXL.M
Last changed : 12/12/2020 5:33:13 PM by 系统
Analysis Method : E:\DATA\20201027\LC 2020-12-15 14-02-17\6IPA_10_0.8_3.M (Sequence Method)
Last changed : 12/15/2020 8:53:09 PM by SYSTEM
(modified after loading)
Additional Info : Peak(s) manually integrated

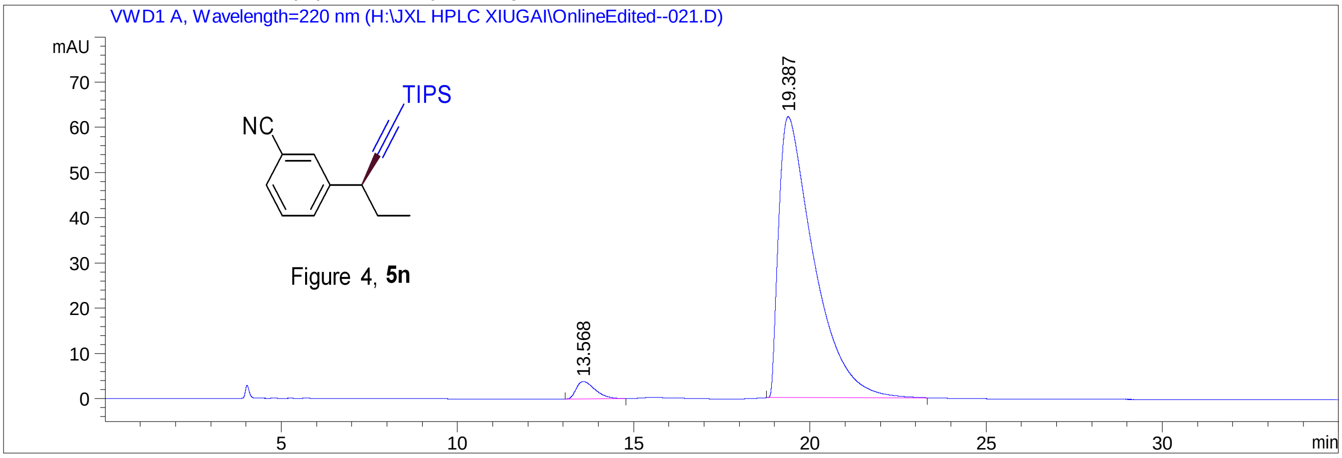

===

Area Percent Report

===

Sorted By : Signal
Multiplier : 1.0000
Dilution : 1.0000
Do not use Multiplier & Dilution Factor with ISTDs

Signal 1: VWD1 A, Wavelength=220 nm

Peak #	RetTime [min]	Type	Width [min]	Area [mAU*s]	Height [mAU]	Area %
1	13.568	BB	0.4392	144.75229	3.87164	3.2444
2	19.387	BB	0.9460	4316.88574	62.13744	96.7556

Totals : 4461.63803 66.00907

===

*** End of Report ***

Supplementary Fig. 242. HPLC spectra for compound **5n**

Data File H:\JXL HPLC XIUGAI\OnlineEdited--110.D
Sample Name: JXL-02-149-5-OD(2)

===

Acq. Operator : 系统 Seq. Line : 110
Acq. Instrument : HPLC-1260 Location : 61
Injection Date : 12/15/2020 1:31:57 AM Inj : 2
Inj Volume : 3.000 µl
Different Inj Volume from Sample Entry! Actual Inj Volume : 5.000 µl
Acq. Method : D:\G527\FZ\20201127\YH 2020-12-12 20-24-28\0IPA-40-0.8-1-6-220-JXL.M
Last changed : 12/14/2020 9:36:57 AM by 系统
Analysis Method : E:\DATA\20201027\LC 2020-12-15 14-02-17\6IPA_10_0.8_3.M (Sequence Method)
Last changed : 12/15/2020 9:00:19 PM by SYSTEM
(modified after loading)
Additional Info : Peak(s) manually integrated

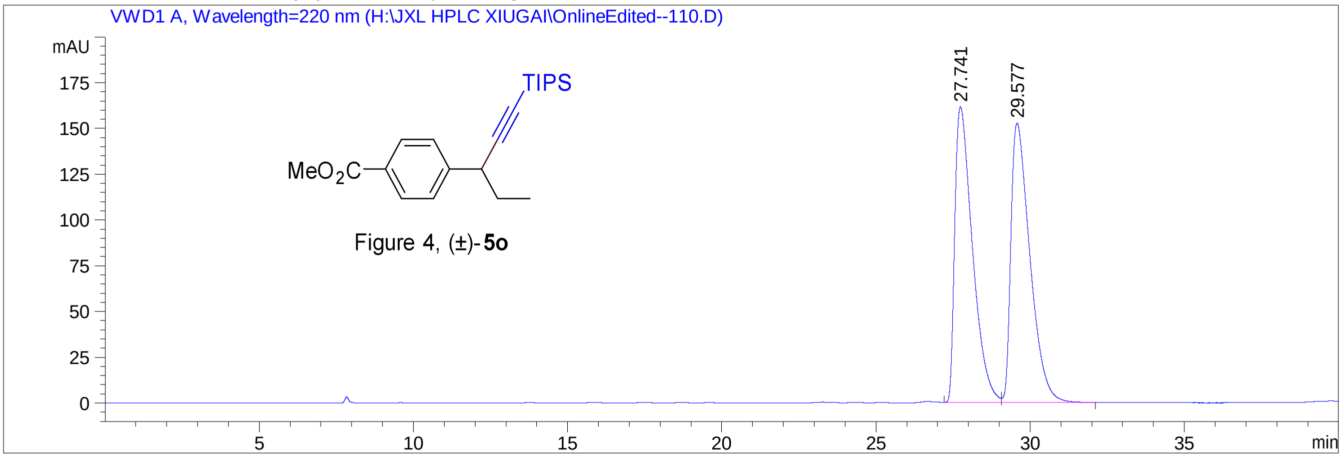

===

Area Percent Report

===

Sorted By : Signal
Multiplier : 1.0000
Dilution : 1.0000
Do not use Multiplier & Dilution Factor with ISTDs

Signal 1: VWD1 A, Wavelength=220 nm

Peak #	RetTime [min]	Type	Width [min]	Area [mAU*s]	Height [mAU]	Area %
1	27.741	BV	0.5997	6440.34473	161.52284	49.5692
2	29.577	VB	0.6333	6552.29932	152.65790	50.4308

Totals : 1.29926e4 314.18074

===

*** End of Report ***

Supplementary Fig. 243. HPLC spectra for compound (±)-**5o**

Data File H:\JXL HPLC XIUGAI\OnlineEdited--111.D
Sample Name: JXL-DB-OD(2)

===
Acq. Operator   : 系统   Seq. Line : 111
Acq. Instrument : HPLC-1260   Location :   62
Injection Date : 12/15/2020 2:13:22 AM   Inj :   1
Inj Volume : 3.000 µl
Different Inj Volume from Sample Entry! Actual Inj Volume : 1.500 µl
Acq. Method   : D:\G527\FZ\20201127\YH 2020-12-12 20-24-28\0IPA-40-0.8-1-6-220-JXL.M
Last changed   : 12/14/2020 9:36:57 AM by 系统
Analysis Method : E:\DATA\20201027\LC 2020-12-15 14-02-17\6IPA_10_0.8_3.M (Sequence Method)
Last changed   : 12/15/2020 8:59:06 PM by SYSTEM
(modified after loading)

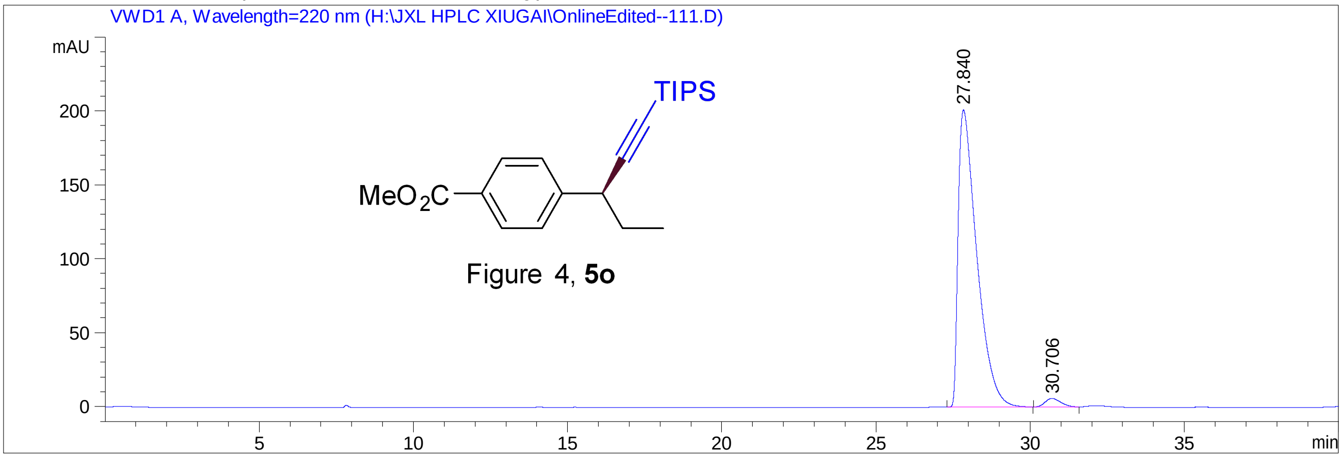

===
Area Percent Report
===

Sorted By : Signal
Multiplier : 1.0000
Dilution : 1.0000
Do not use Multiplier & Dilution Factor with ISTDs

Signal 1: VWD1 A, Wavelength=220 nm

Peak #	RetTime [min]	Type	Width [min]	Area [mAU*s]	Height [mAU]	Area %
1	27.840	BB	0.6234	8452.31738	201.06433	97.6544
2	30.706	BB	0.4168	203.01605	5.77937	2.3456

Totals : 8655.33344 206.84370

===
*** End of Report ***

Supplementary Fig. 244. HPLC spectra for compound **5o**

Data File H:\JXL HPLC\JXL-02-149-6-OD(1)-RAC.D
Sample Name: JXL-02-149-6-OD

===

Acq. Operator : 系统 Seq. Line : 31
Acq. Instrument : HPLC-1260 Location : 95
Injection Date : 6/15/2020 8:54:24 PM Inj : 1
Inj Volume : 3.000 µl
Different Inj Volume from Sample Entry! Actual Inj Volume : 0.500 µl
Acq. Method : D:\zy\20200615\YH 2020-06-15 08-21-52\0IPA-30-0.5-1-2-220-JXL.M
Last changed : 6/15/2020 8:52:56 PM by 系统
Analysis Method : C:\CHEM32\1\METHODS\15IPA_30_8_4.M
Last changed : 12/18/2020 9:43:32 PM by SYSTEM
(modified after loading)
Additional Info : Peak(s) manually integrated

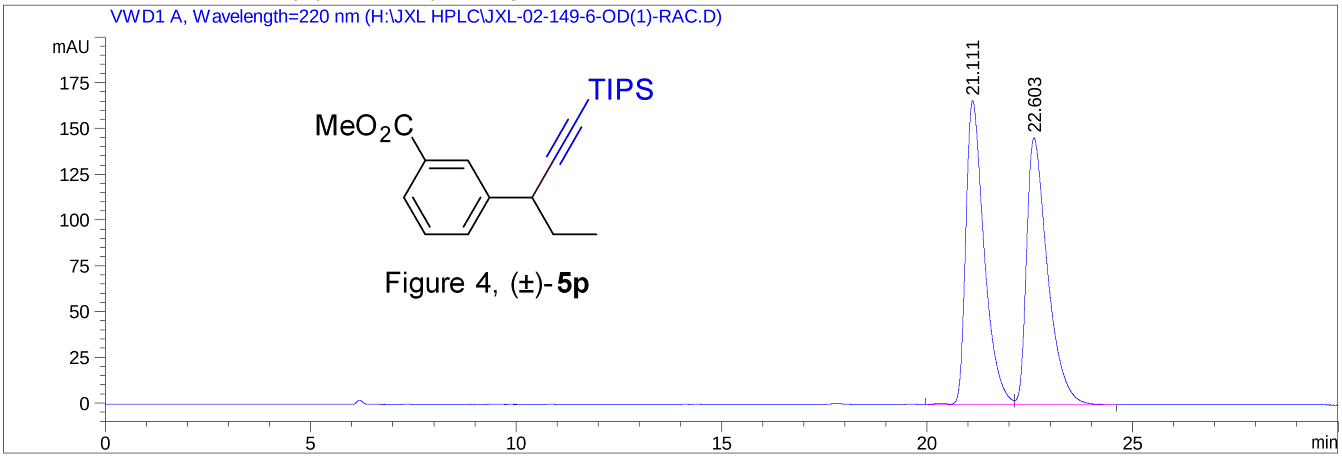

===

Area Percent Report

===

Sorted By : Signal
Multiplier : 1.0000
Dilution : 1.0000
Do not use Multiplier & Dilution Factor with ISTDs

Signal 1: VWD1 A, Wavelength=220 nm

Peak #	RetTime [min]	Type	Width [min]	Area [mAU*s]	Height [mAU]	Area %
1	21.111	VV R	0.4701	5171.46680	165.94023	50.0069
2	22.603	VB	0.5322	5170.04248	145.63780	49.9931

Totals : 1.03415e4 311.57803

===

*** End of Report ***

Supplementary Fig. 245. HPLC spectra for compound (±)-**5p**

Data File H:\JXL HPLC\JXL-02-149-2-OD-EE.D
Sample Name: JXL-02-149-2-OD

===
Acq. Operator : 系统 Seq. Line : 49
Acq. Instrument : HPLC-1260 Location : 96
Injection Date : 6/16/2020 4:59:14 PM Inj : 1
Inj Volume : 3.000 µl
Different Inj Volume from Sample Entry! Actual Inj Volume : 0.400 µl
Acq. Method : D:\G527\FZ\20200611\YH 2020-06-15 23-14-24\0IPA-30-0.5-1-2-220-JXL.M
Last changed : 6/16/2020 8:53:54 AM by 系统
Analysis Method : E:\DATA\20201027\LC 2020-11-24 17-29-49\1IPA-40-0.5-220-3-JXL.M (Sequence Method)
Last changed : 12/16/2020 11:40:15 AM by SYSTEM
(modified after loading)
Additional Info : Peak(s) manually integrated

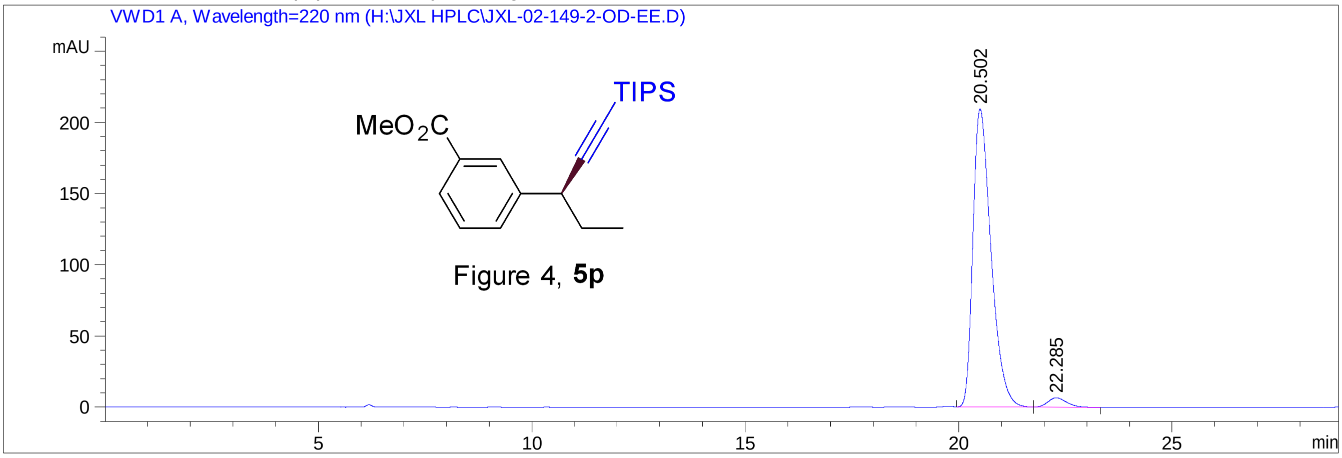

===
Area Percent Report
===

Sorted By : Signal
Multiplier : 1.0000
Dilution : 1.0000
Do not use Multiplier & Dilution Factor with ISTDs

Signal 1: VWD1 A, Wavelength=220 nm

Peak #	RetTime [min]	Type	Width [min]	Area [mAU*s]	Height [mAU]	Area %
1	20.502	BB	0.4508	6165.68359	209.24425	96.8537
2	22.285	BB	0.3691	200.29059	6.55361	3.1463

Totals : 6365.97418 215.79785

===
*** End of Report ***

Supplementary Fig. 246. HPLC spectra for compound **5p**

Data File H:\JXL HPLC\JXL-02-157-2-OD(2)-RAC.D
Sample Name: JXL-02-157-2-OD(2)

===

Acq. Operator : 系统 Seq. Line : 87
Acq. Instrument : HPLC-1260 Location : 62
Injection Date : 6/21/2020 8:45:38 AM Inj : 1
Inj Volume : 3.000 µl
Different Inj Volume from Sample Entry! Actual Inj Volume : 0.500 µl
Acq. Method : D:\zy\20200619\YH 2020-06-19 10-26-40\0IPA-60-0.5-1-2-220-JXL.M
Last changed : 6/20/2020 11:08:48 PM by 系统
Analysis Method : E:\DATA\20201027\LC 2020-12-19 17-11-35\0.6IPA_15_0.8_3.CJ.M (Sequence Method)
Last changed : 12/20/2020 3:24:03 PM by SYSTEM
(modified after loading)
Additional Info : Peak(s) manually integrated

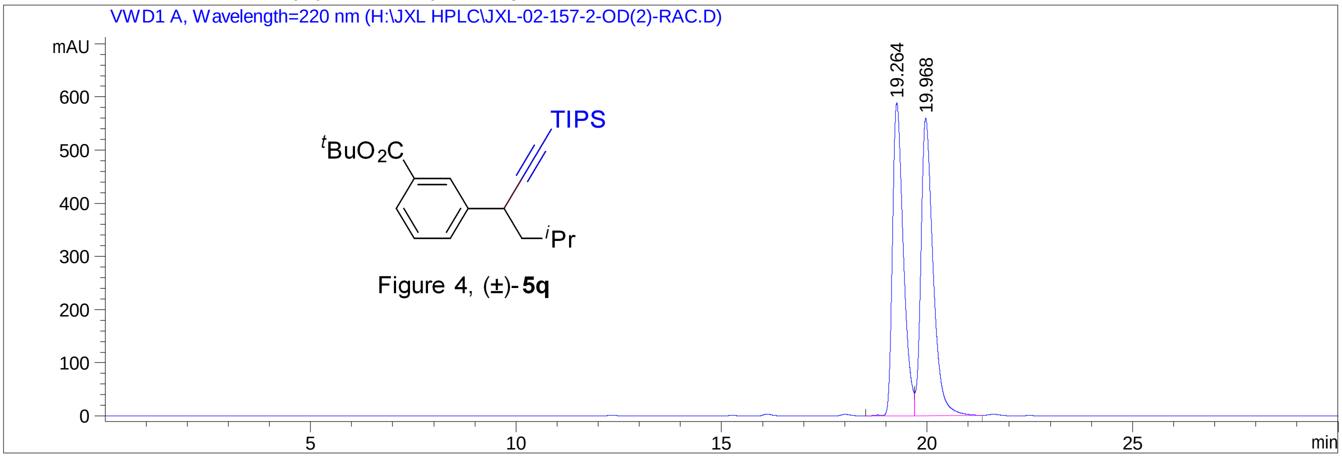

===
Area Percent Report
===

Sorted By : Signal
Multiplier : 1.0000
Dilution : 1.0000
Do not use Multiplier & Dilution Factor with ISTDs

Signal 1: VWD1 A, Wavelength=220 nm

Peak #	RetTime [min]	Type	Width [min]	Area [mAU*s]	Height [mAU]	Area %
1	19.264	VV R	0.2823	1.08682e4	588.87714	48.5491
2	19.968	VB	0.3102	1.15178e4	559.51123	51.4509

Totals : 2.23860e4 1148.38837

===
*** End of Report ***

Supplementary Fig. 247. HPLC spectra for compound (±)-**5q**

Data File H:\JXL HPLC\JXL-03-115-OD(2)-EE.D
Sample Name: JXL-115-OD(2)

===

Acq. Operator : 系统 Seq. Line : 124
Acq. Instrument : HPLC-1260 Location : 86
Injection Date : 11/1/2020 6:47:32 AM Inj : 1
Inj Volume : 3.000 µl
Different Inj Volume from Sample Entry! Actual Inj Volume : 0.800 µl
Acq. Method : D:\G527\FZ\20201026\YH 2020-10-29 08-35-49\0IPA-40-0.5-1-6-220-JXL.M
Last changed : 10/31/2020 10:15:10 PM by 系统
Analysis Method : E:\DATA\20201027\LC 2020-12-15 14-02-17\6IPA_10_0.8_3.M (Sequence Method)
Last changed : 12/15/2020 9:24:30 PM by SYSTEM
(modified after loading)
Additional Info : Peak(s) manually integrated

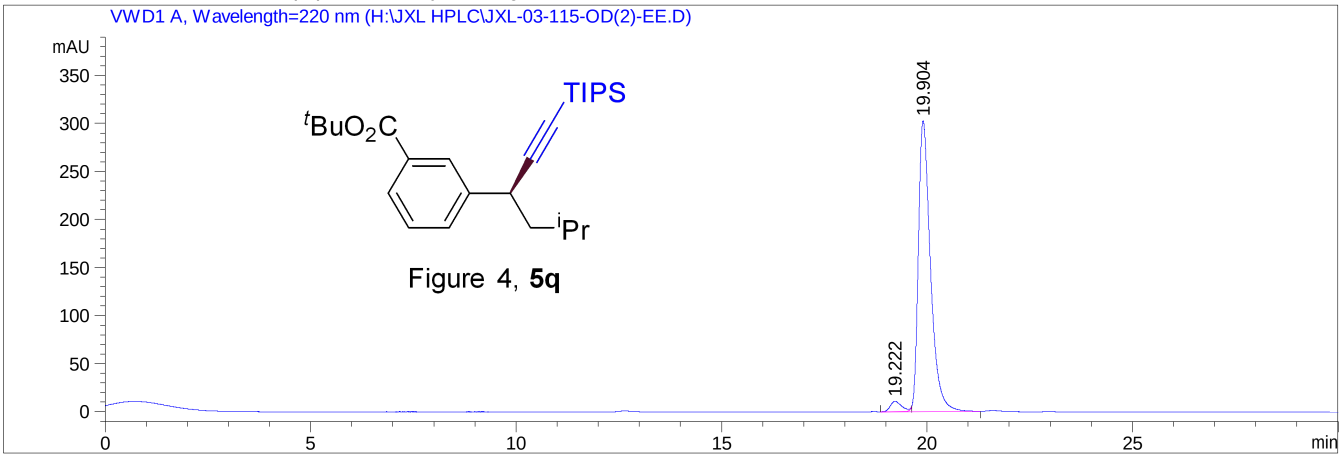

===

Area Percent Report

===

Sorted By : Signal
Multiplier : 1.0000
Dilution : 1.0000
Do not use Multiplier & Dilution Factor with ISTDs

Signal 1: VWD1 A, Wavelength=220 nm

Peak #	RetTime [min]	Type	Width [min]	Area [mAU*s]	Height [mAU]	Area %
1	19.222	BV E	0.2773	208.75603	10.95368	3.2626
2	19.904	VB R	0.3090	6189.72070	302.73431	96.7374

Totals : 6398.47673 313.68800

===

*** End of Report ***

Supplementary Fig. 248. HPLC spectra for compound **5q**

Data File H:\JXL HPLC\JXL-03-157-2-OD(2)-RAC.D
Sample Name: JXL-03-157-2-OD(2)

===

Acq. Operator : 系统 Seq. Line : 35
Acq. Instrument : HPLC-1260 Location : 94
Injection Date : 9/22/2020 8:11:49 AM Inj : 1
Inj Volume : 3.000 µl
Different Inj Volume from Sample Entry! Actual Inj Volume : 0.500 µl
Acq. Method : D:\G527\FZ\20200822\YH 2020-09-21 18-04-54\0IPA-35-0.3-1-6-220-JXL.M
Last changed : 9/21/2020 10:01:05 PM by 系统
Analysis Method : E:\DATA\20201027\LC 2020-12-15 14-02-17\6IPA_10_0.8_3.M (Sequence Method)
Last changed : 12/15/2020 9:26:51 PM by SYSTEM
(modified after loading)
Additional Info : Peak(s) manually integrated

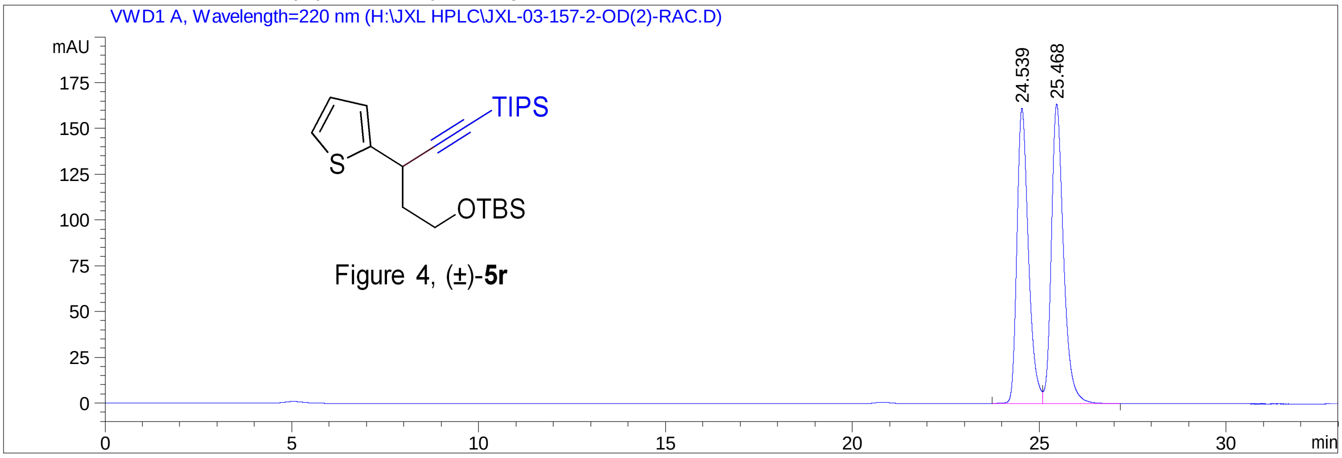

===

Area Percent Report

===

Sorted By : Signal
Multiplier : 1.0000
Dilution : 1.0000
Do not use Multiplier & Dilution Factor with ISTDs

Signal 1: VWD1 A, Wavelength=220 nm

Peak #	RetTime [min]	Type	Width [min]	Area [mAU*s]	Height [mAU]	Area %
1	24.539	BV	0.3261	3451.30762	161.34341	48.1006
2	25.468	VB	0.3434	3723.87671	163.69365	51.8994

Totals : 7175.18433 325.03706

===

*** End of Report ***

Supplementary Fig. 249. HPLC spectra for compound (±)-**5r**

Data File H:\JXL HPLC\JXL-03-166-1-OD(2)-EE.D
Sample Name: JXL-03-166-1-OD(2)

===

Acq. Operator : 系统 Seq. Line : 38
Acq. Instrument : HPLC-1260 Location : 95
Injection Date : 9/22/2020 9:58:01 AM Inj : 1
Inj Volume : 3.000 µl
Different Inj Volume from Sample Entry! Actual Inj Volume : 0.300 µl
Acq. Method : D:\G527\FZ\20200822\YH 2020-09-21 18-04-54\0IPA-35-0.3-1-6-220-JXL.M
Last changed : 9/22/2020 9:56:24 AM by 系统
Analysis Method : E:\DATA\20201027\LC 2020-12-15 14-02-17\6IPA_10_0.8_3.M (Sequence Method)
Last changed : 12/15/2020 9:28:34 PM by SYSTEM
(modified after loading)
Additional Info : Peak(s) manually integrated

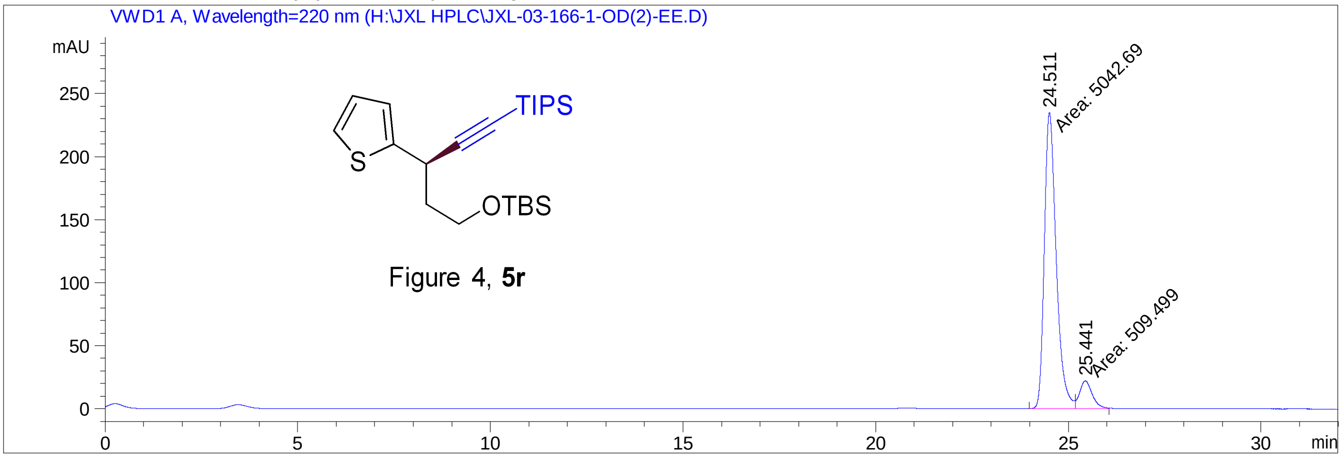

===

Area Percent Report

===

Sorted By : Signal
Multiplier : 1.0000
Dilution : 1.0000
Do not use Multiplier & Dilution Factor with ISTDs

Signal 1: VWD1 A, Wavelength=220 nm

Peak #	RetTime [min]	Type	Width [min]	Area [mAU*s]	Height [mAU]	Area %
1	24.511	FM	0.3576	5042.69092	235.04404	90.8235
2	25.441	MF	0.3822	509.49850	22.21989	9.1765

Totals : 5552.18942 257.26392

===

*** End of Report ***

Supplementary Fig. 250. HPLC spectra for compound **5r**

Data File H:\JXL HPLC XIUGAI\OnlineEdited--116.D
Sample Name: JXL-02-157-3-OD(2)

===

Acq. Operator : 系统 Seq. Line : 116
Acq. Instrument : HPLC-1260 Location : 83
Injection Date : 12/15/2020 5:00:41 AM Inj : 1
Inj Volume : 3.000 µl
Different Inj Volume from Sample Entry! Actual Inj Volume : 1.000 µl
Acq. Method : D:\G527\FZ\20201127\YH 2020-12-12 20-24-28\0IPA-30-0.8-1-6-220-JXL.M
Last changed : 12/14/2020 9:37:57 AM by 系统
Analysis Method : E:\DATA\20201027\LC 2020-11-24 17-29-49\1IPA-40-0.5-220-3-JXL.M (Sequence Method)
Last changed : 12/16/2020 11:43:39 AM by SYSTEM
(modified after loading)

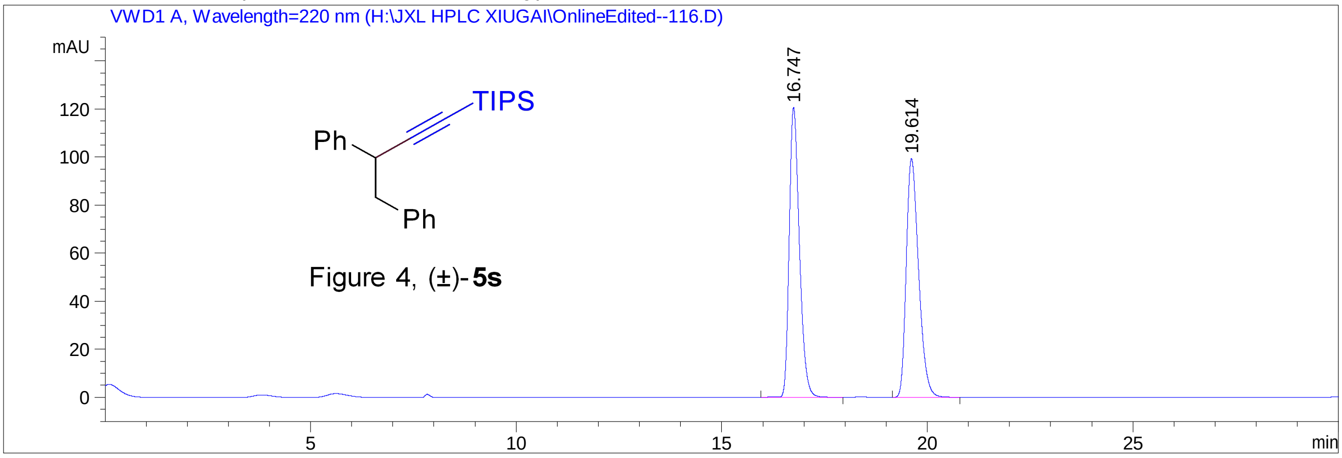

===

Area Percent Report

===

Sorted By : Signal
Multiplier : 1.0000
Dilution : 1.0000
Do not use Multiplier & Dilution Factor with ISTDs

Signal 1: VWD1 A, Wavelength=220 nm

Peak #	RetTime [min]	Type	Width [min]	Area [mAU*s]	Height [mAU]	Area %
1	16.747	VB R	0.2644	2085.05396	120.80421	50.0368
2	19.614	BB	0.3213	2081.99048	99.44037	49.9632

Totals : 4167.04443 220.24458

===

*** End of Report ***

Supplementary Fig. 251. HPLC spectra for compound (±)-**5s**

Data File H:\JXL HPLC XIUGAI\OnlineEdited--117.D
Sample Name: JXL-02-167-2-OD(2)

===

Acq. Operator : 系统 Seq. Line : 117
Acq. Instrument : HPLC-1260 Location : 84
Injection Date : 12/15/2020 5:32:11 AM Inj : 1
Inj Volume : 3.000 µl
Different Inj Volume from Sample Entry! Actual Inj Volume : 1.000 µl
Acq. Method : D:\G527\FZ\20201127\YH 2020-12-12 20-24-28\0IPA-30-0.8-1-6-220-JXL.M
Last changed : 12/14/2020 9:37:57 AM by 系统
Analysis Method : E:\DATA\20201027\LC 2020-11-24 17-29-49\1IPA-40-0.5-220-3-JXL.M (Sequence Method)
Last changed : 12/16/2020 11:45:08 AM by SYSTEM
(modified after loading)
Additional Info : Peak(s) manually integrated

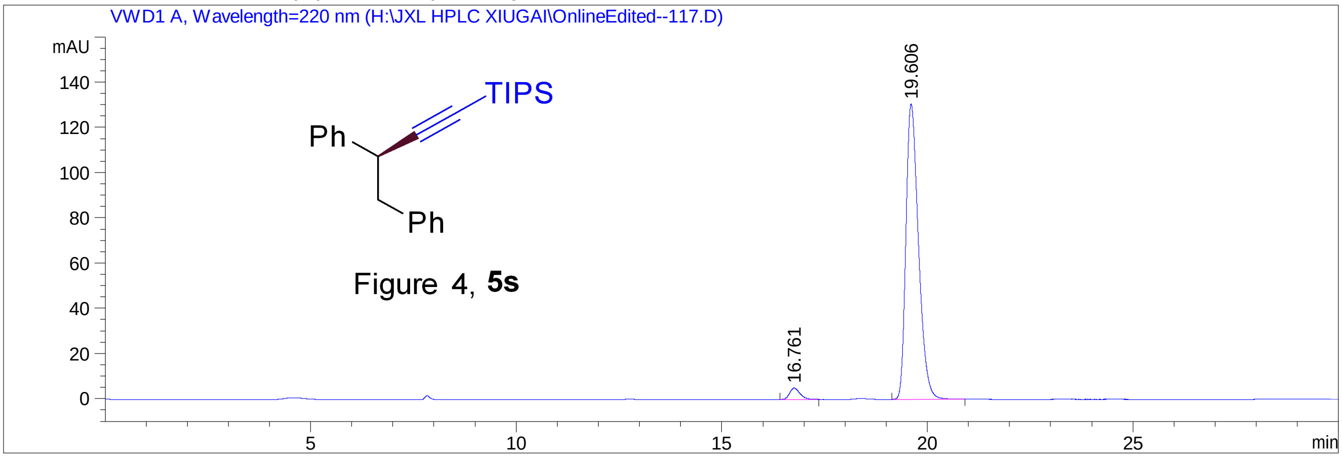

===

Area Percent Report

===

Sorted By : Signal
Multiplier : 1.0000
Dilution : 1.0000
Do not use Multiplier & Dilution Factor with ISTDs

Signal 1: VWD1 A, Wavelength=220 nm

Peak #	RetTime [min]	Type	Width [min]	Area [mAU*s]	Height [mAU]	Area %
1	16.761	BB	0.2505	86.25835	5.04446	3.0187
2	19.606	BB	0.3264	2771.22437	130.70523	96.9813

Totals : 2857.48272 135.74969

===

*** End of Report ***

Supplementary Fig. 252. HPLC spectra for compound **5s**

Data File H:\JXL HPLC XIUGAI\091-0802--010.D
Sample Name: JXL-03-31-1-OD(2)

===

Acq. Operator : 系统 Seq. Line : 10
Acq. Instrument : HPLC-1260 Location : 91
Injection Date : 8/8/2020 8:42:21 PM Inj : 2
Inj Volume : 3.000 µl
Different Inj Volume from Sample Entry! Actual Inj Volume : 0.500 µl
Acq. Method : D:\JXL\20200808\YH 2020-08-08 16-05-24\0IPA-40-0.3-1-2-220-JXL.M
Last changed : 8/8/2020 9:16:15 PM by 系统
(modified after loading)
Analysis Method : E:\DATA\20201027\LC 2020-12-19 17-11-35\10IPA_45_0.8_4.M (Sequence Method)
Last changed : 12/20/2020 1:16:54 PM by SYSTEM
(modified after loading)
Additional Info : Peak(s) manually integrated

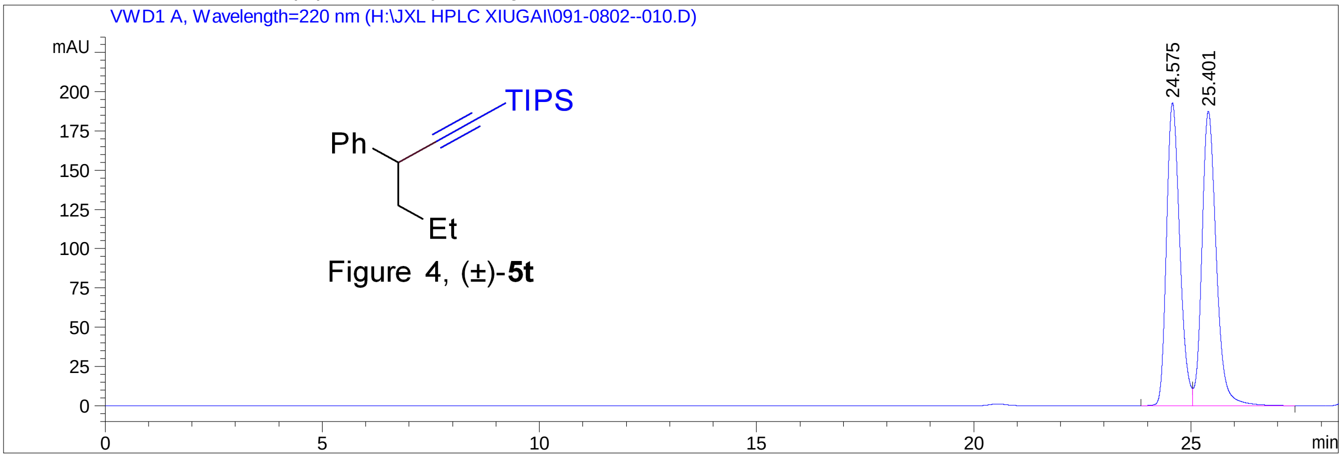

===

Area Percent Report

===

Sorted By : Signal
Multiplier : 1.0000
Dilution : 1.0000
Do not use Multiplier & Dilution Factor with ISTDs

Signal 1: VWD1 A, Wavelength=220 nm

Peak #	RetTime [min]	Type	Width [min]	Area [mAU*s]	Height [mAU]	Area %
1	24.575	BV	0.3294	4100.78613	193.01289	48.7329
2	25.401	VB	0.3520	4314.04102	187.80188	51.2671

Totals : 8414.82715 380.81477

===

*** End of Report ***

Supplementary Fig. 253. HPLC spectra for compound (±)-**5t**

Data File H:\JXL HPLC\JXL-03-101-1-OD(2).D
Sample Name: JXL-03-101-1-OD(2)

===
Acq. Operator : 系统 Seq. Line : 13
Acq. Instrument : HPLC-1260 Location : 95
Injection Date : 8/20/2020 3:34:30 AM Inj : 1
Inj Volume : 3.000 µl
Different Inj Volume from Sample Entry! Actual Inj Volume : 0.200 µl
Acq. Method : D:\JXL\20200819\YH 2020-08-19 21-39-43\0IPA-30-0.3-1-6-220-JXL.M
Last changed : 8/19/2020 9:39:43 PM by 系统
Analysis Method : C:\CHEM32\1\METHODS\1IPA_20_1.0_3.CJM.M
Last changed : 12/16/2020 2:45:22 PM by SYSTEM
(modified after loading)
Additional Info : Peak(s) manually integrated

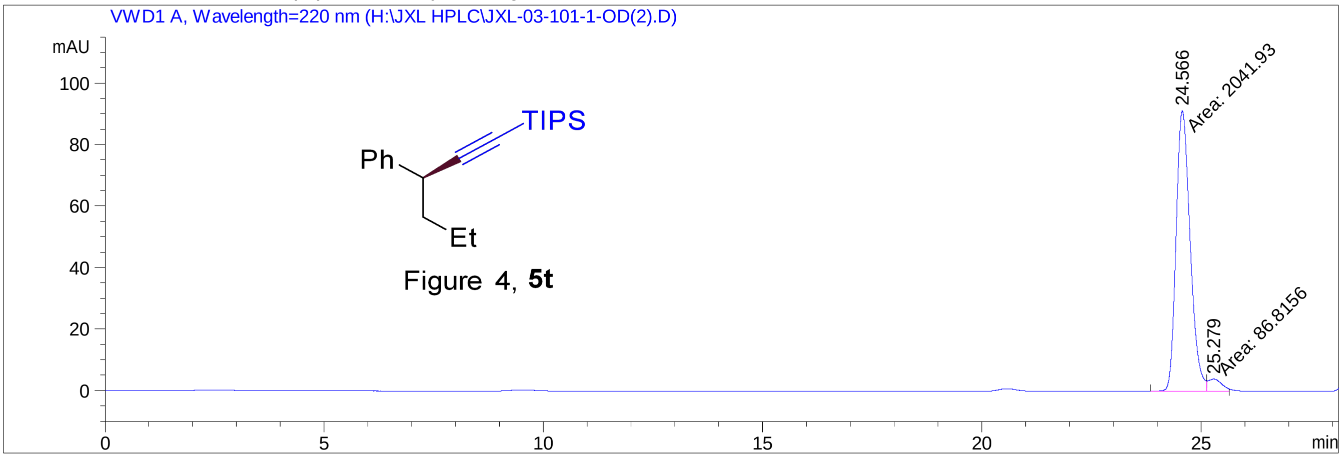

===
Area Percent Report
===

Sorted By : Signal
Multiplier : 1.0000
Dilution : 1.0000
Do not use Multiplier & Dilution Factor with ISTDs

Signal 1: VWD1 A, Wavelength=220 nm

Peak #	RetTime [min]	Type	Width [min]	Area [mAU*s]	Height [mAU]	Area %
1	24.566	MF	0.3732	2041.93396	91.18871	95.9218
2	25.279	MF	0.3618	86.81557	3.99962	4.0782

Totals : 2128.74953 95.18834

===
*** End of Report ***

Supplementary Fig. 254. HPLC spectra for compound **5t**

Data File H:\JXL HPLC XIUGAI\OnlineEdited--019.D
Sample Name: JXL-02-164-6-OD(2)

===

Acq. Operator : 系统 Seq. Line : 19
Acq. Instrument : HPLC-1260 Location : 91
Injection Date : 8/23/2020 1:24:20 AM Inj : 1
Inj Volume : 3.000 µl
Different Inj Volume from Sample Entry! Actual Inj Volume : 0.200 µl
Acq. Method : D:\G527\FZ\20200822\YH 2020-08-22 18-57-44\0IPA-40-0.8-1-2-220-JXL.M
Last changed : 8/22/2020 9:23:07 PM by 系统
Analysis Method : E:\DATA\20201027\LC 2020-12-18 10-52-42\20IPA_15_10_3.M (Sequence Method)
Last changed : 12/18/2020 3:03:04 PM by SYSTEM
(modified after loading)
Additional Info : Peak(s) manually integrated

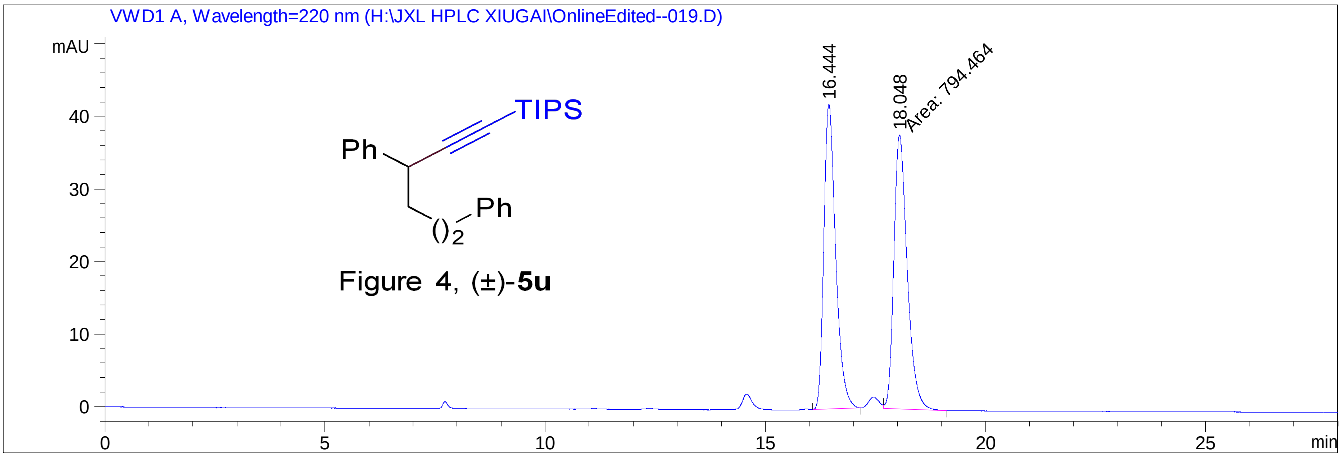

===

Area Percent Report

===

Sorted By : Signal
Multiplier : 1.0000
Dilution : 1.0000
Do not use Multiplier & Dilution Factor with ISTDs

Signal 1: VWD1 A, Wavelength=220 nm

Peak #	RetTime [min]	Type	Width [min]	Area [mAU*s]	Height [mAU]	Area %
1	16.444	BB	0.2856	786.78638	41.99541	49.7572
2	18.048	FM	0.3504	794.46375	37.78609	50.2428

Totals : 1581.25012 79.78150

===

*** End of Report ***

Supplementary Fig. 255. HPLC spectra for compound (±)-**5u**

Data File H:\JXL HPLC\JXL-03-108-1-OD(2)-EE.D
Sample Name: JXL-03-108-1

===

Acq. Operator : 系统 Seq. Line : 3
Acq. Instrument : HPLC-1260 Location : 92
Injection Date : 8/23/2020 8:08:34 PM Inj : 1
Inj Volume : 3.000 µl
Different Inj Volume from Sample Entry! Actual Inj Volume : 0.600 µl
Acq. Method : D:\JXL\20200823\YH 2020-08-23 19-08-47\0IPA-40-0.8-1-2-220-JXL.M
Last changed : 8/23/2020 8:45:56 PM by 系统
(modified after loading)
Analysis Method : E:\DATA\20201027\LC 2020-12-18 10-52-42\20IPA_15_10_3.M (Sequence Method)
Last changed : 12/18/2020 3:05:03 PM by SYSTEM
(modified after loading)
Additional Info : Peak(s) manually integrated

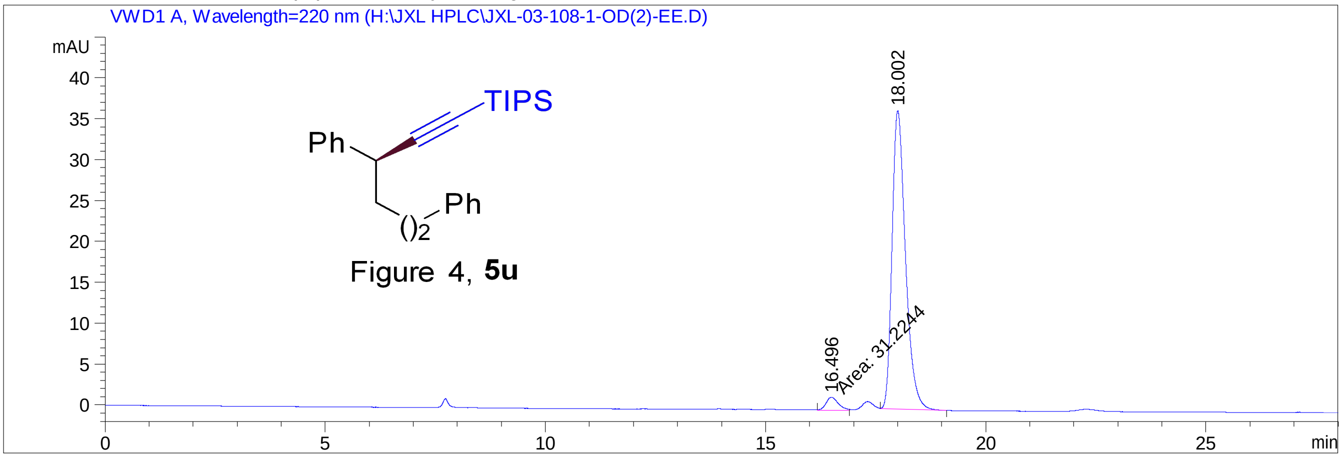

===

Area Percent Report

===

Sorted By : Signal
Multiplier : 1.0000
Dilution : 1.0000
Do not use Multiplier & Dilution Factor with ISTDs

Signal 1: VWD1 A, Wavelength=220 nm

Peak #	RetTime [min]	Type	Width [min]	Area [mAU*s]	Height [mAU]	Area %
1	16.496	MM	0.3235	31.22440	1.60860	3.8895
2	18.002	BB	0.3203	771.57056	36.47853	96.1105

Totals : 802.79495 38.08713

===

*** End of Report ***

Supplementary Fig. 256. HPLC spectra for compound **5u**

Data File H:\JXL HPLC\JXL-03-117-2-OD(2)-RAC.D
Sample Name: JXL-03-117-2-OD(2)

===

Acq. Operator : 系统 Seq. Line : 5
Acq. Instrument : HPLC-1260 Location : 92
Injection Date : 8/28/2020 1:12:31 AM Inj : 1
Inj Volume : 3.000 µl
Different Inj Volume from Sample Entry! Actual Inj Volume : 0.300 µl
Acq. Method : D:\JXL\20200827\YH 2020-08-27 23-15-17\0IPA-40-0.8-1-2-220-JXL.M
Last changed : 8/27/2020 11:27:50 PM by 系统
Analysis Method : E:\DATA\20201027\LC 2020-12-15 14-02-17\6IPA_10_0.8_3.M (Sequence Method)
Last changed : 12/15/2020 10:07:12 PM by SYSTEM
(modified after loading)
Additional Info : Peak(s) manually integrated

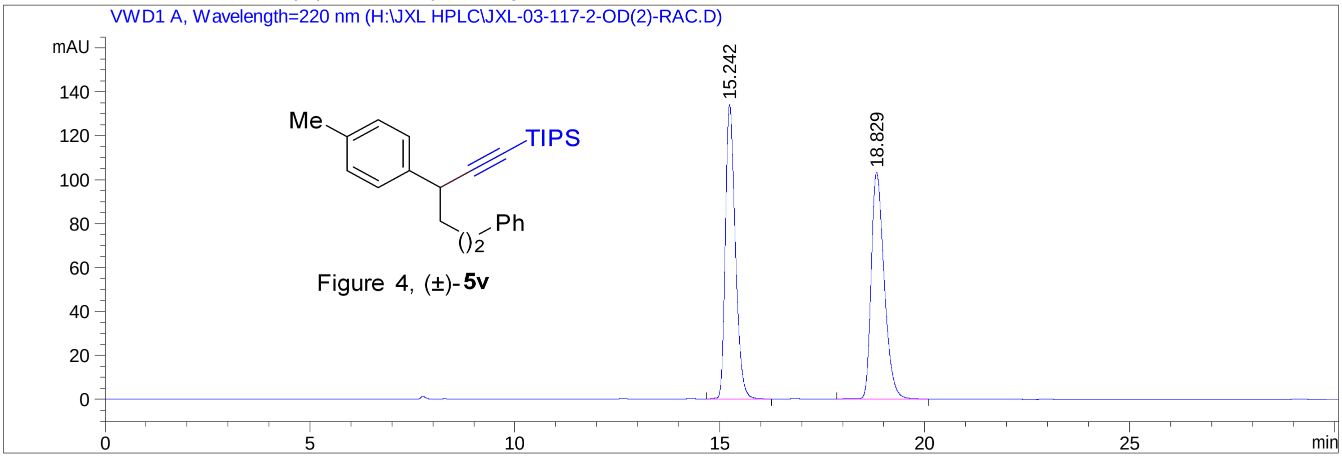

===

Area Percent Report

===

Sorted By : Signal
Multiplier : 1.0000
Dilution : 1.0000
Do not use Multiplier & Dilution Factor with ISTDs

Signal 1: VWD1 A, Wavelength=220 nm

Peak #	RetTime [min]	Type	Width [min]	Area [mAU*s]	Height [mAU]	Area %
1	15.242	BB	0.2599	2274.47729	134.07082	49.9280
2	18.829	BB	0.3388	2281.03564	103.21310	50.0720

Totals : 4555.51294 237.28392

===

*** End of Report ***

Supplementary Fig. 257. HPLC spectra for compound (±)-**5v**

Data File H:\JXL HPLC XIUGAI\OnlineEdited--067.D
Sample Name: JXL-03-133-1-XIA-OD(2)

===

Acq. Operator : 系统 Seq. Line : 67
Acq. Instrument : HPLC-1260 Location : 88
Injection Date : 9/2/2020 10:39:15 AM Inj : 1
Inj Volume : 3.000 µl
Different Inj Volume from Sample Entry! Actual Inj Volume : 0.500 µl
Acq. Method : D:\zy\20200901\YH 2020-09-01 09-59-23\0IPA-40-0.8-1-6-220-JXL.M
Last changed : 9/1/2020 10:11:54 PM by 系统
Analysis Method : C:\CHEM32\1\METHODS\1IPA_20_1.0_3.CJM.M
Last changed : 12/16/2020 2:48:23 PM by SYSTEM
(modified after loading)
Additional Info : Peak(s) manually integrated

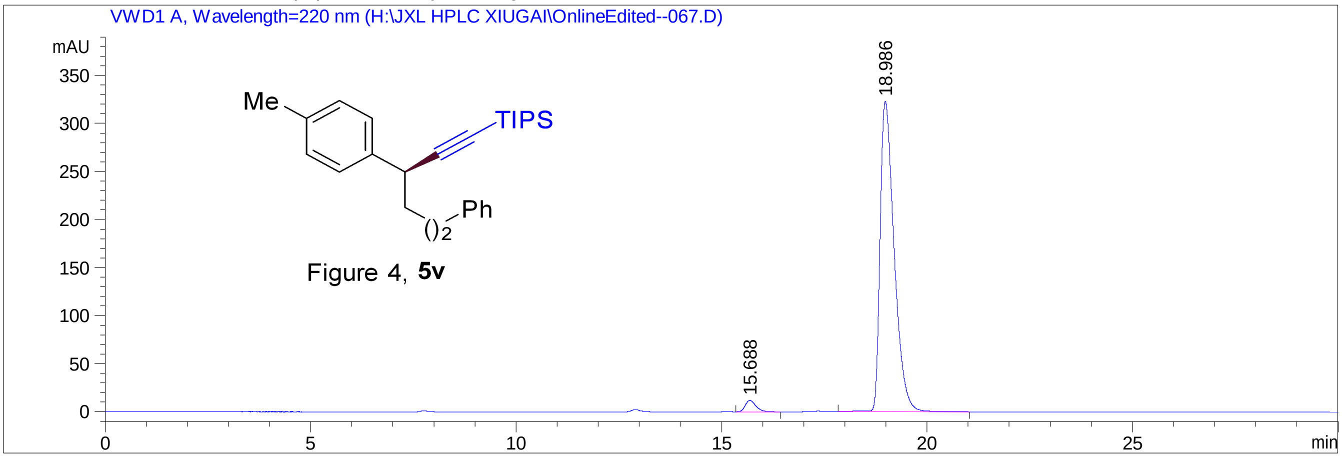

===

Area Percent Report

===

Sorted By : Signal
Multiplier : 1.0000
Dilution : 1.0000
Do not use Multiplier & Dilution Factor with ISTDs

Signal 1: VWD1 A, Wavelength=220 nm

Peak #	RetTime [min]	Type	Width [min]	Area [mAU*s]	Height [mAU]	Area %
1	15.688	BB	0.2716	211.30690	11.79120	2.6762
2	18.986	VB R	0.3611	7684.48779	322.94391	97.3238

Totals : 7895.79469 334.73511

===

*** End of Report ***

HPLC1260 12/16/2020 2:48:33 PM SYSTEM Page 1 of 1

Supplementary Fig. 258. HPLC spectra for compound **5v**

Data File H:\JXL HPLC\JXL-03-131-4-OD(2)-1.D
Sample Name: JXL-03-131-4-OD(2)

===

Acq. Operator : 系统 Seq. Line : 36
Acq. Instrument : HPLC-1260 Location : 89
Injection Date : 9/3/2020 11:05:09 PM Inj : 1
Inj Volume : 3.000 µl
Different Inj Volume from Sample Entry! Actual Inj Volume : 2.000 µl
Acq. Method : D:\JXL\20200902\YH 2020-09-02 16-57-48\0IPA-90-0.5-1-6-220-JXL.M
Last changed : 9/3/2020 11:09:41 PM by 系统
(modified after loading)
Analysis Method : E:\DATA\20201027\LC 2020-12-15 14-02-17\6IPA_10_0.8_3.M (Sequence Method)
Last changed : 12/15/2020 10:10:58 PM by SYSTEM
(modified after loading)
Additional Info : Peak(s) manually integrated

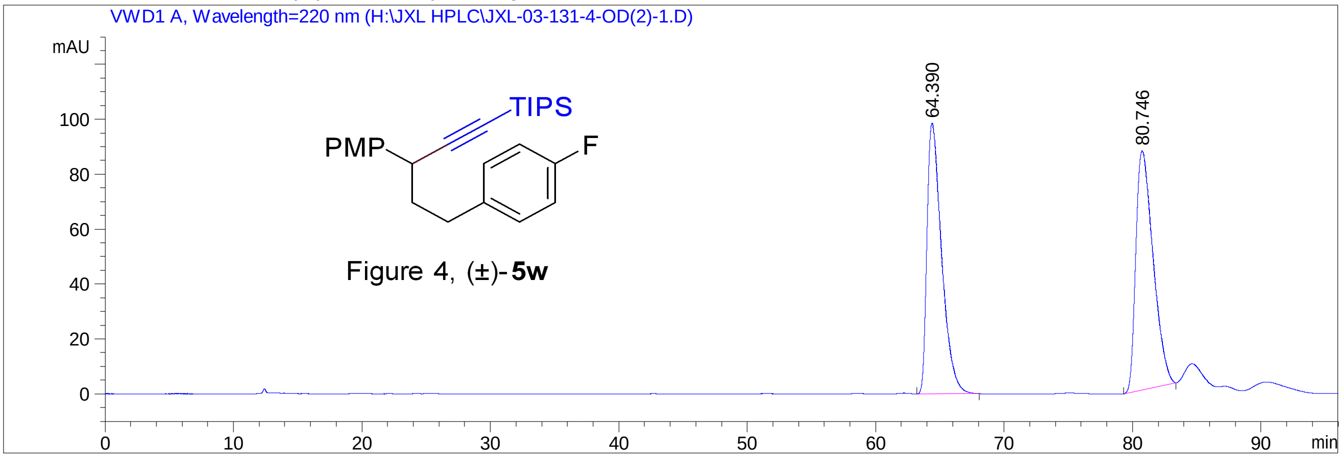

===

Area Percent Report

===

Sorted By : Signal
Multiplier : 1.0000
Dilution : 1.0000
Do not use Multiplier & Dilution Factor with ISTDs

Signal 1: VWD1 A, Wavelength=220 nm

Peak #	RetTime [min]	Type	Width [min]	Area [mAU*s]	Height [mAU]	Area %
1	64.390	BB	1.0778	7801.03662	98.67648	48.9508
2	80.746	BB	1.2471	8135.43311	87.11023	51.0492

Totals : 1.59365e4 185.78671

===

*** End of Report ***

Supplementary Fig. 259. HPLC spectra for compound (±)-**5w**

Data File H:\JXL HPLC\JXL-03-123-1-XIA-OD(2).D
Sample Name: JXL-03-123-1-XIA-OD(2)

===

Acq. Operator : 系统 Seq. Line : 35
Acq. Instrument : HPLC-1260 Location : 72
Injection Date : 9/3/2020 9:33:43 PM Inj : 1
Inj Volume : 3.000 µl
Different Inj Volume from Sample Entry! Actual Inj Volume : 2.000 µl
Acq. Method : D:\JXL\20200902\YH 2020-09-02 16-57-48\0IPA-90-0.5-1-6-220-JXL.M
Last changed : 9/3/2020 11:02:41 PM by 系统
(modified after loading)
Analysis Method : E:\DATA\20201027\LC 2020-12-15 14-02-17\6IPA_10_0.8_3.M (Sequence Method)
Last changed : 12/15/2020 10:12:40 PM by SYSTEM
(modified after loading)

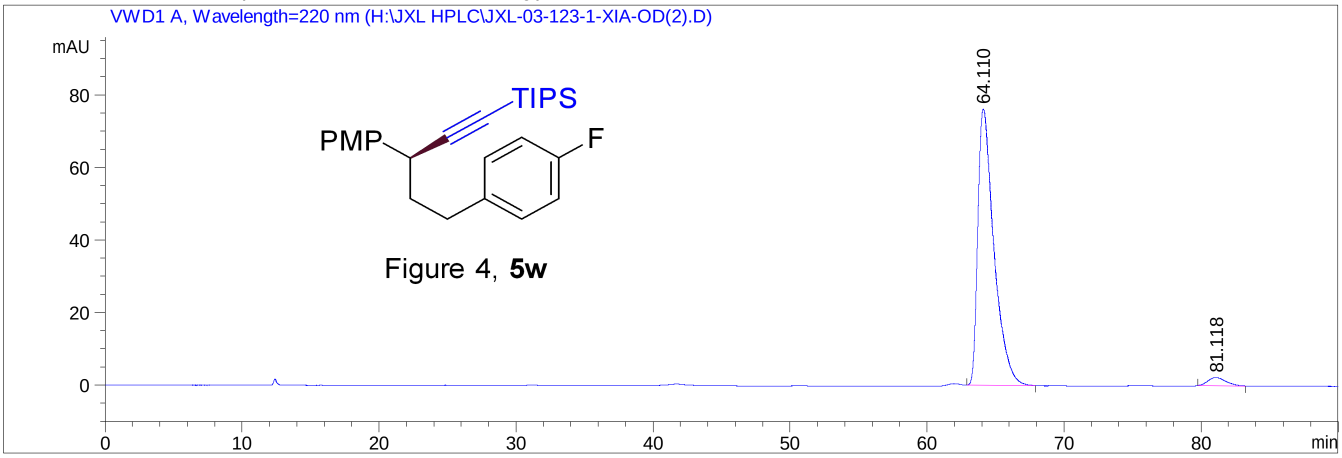

===

Area Percent Report

===

Sorted By : Signal
Multiplier : 1.0000
Dilution : 1.0000
Do not use Multiplier & Dilution Factor with ISTDs

Signal 1: VWD1 A, Wavelength=220 nm

Peak #	RetTime [min]	Type	Width [min]	Area [mAU*s]	Height [mAU]	Area %
1	64.110	BB	1.1146	6215.83203	76.20796	96.8170
2	81.118	BB	1.0648	204.35680	2.24434	3.1830

Totals : 6420.18883 78.45230

===

*** End of Report ***

Supplementary Fig. 260. HPLC spectra for compound **5w**

Data File H:\JXL HPLC\JXL-03-126-2-OD(2)-RAC.D
Sample Name: JXL-03-126-2-OD(2)

===

Acq. Operator : 系统 Seq. Line : 9
Acq. Instrument : HPLC-1260 Location : 81
Injection Date : 9/3/2020 1:06:15 AM Inj : 1
Inj Volume : 3.000 µl
Different Inj Volume from Sample Entry! Actual Inj Volume : 0.800 µl
Acq. Method : D:\JXL\20200902\YH 2020-09-02 16-57-48\0IPA-30-0.5-1-6-220-JXL.M
Last changed : 9/2/2020 11:43:41 PM by 系统
Analysis Method : E:\DATA\20201027\LC 2020-12-15 14-02-17\6IPA_10_0.8_3.M (Sequence Method)
Last changed : 12/15/2020 10:13:54 PM by SYSTEM
(modified after loading)

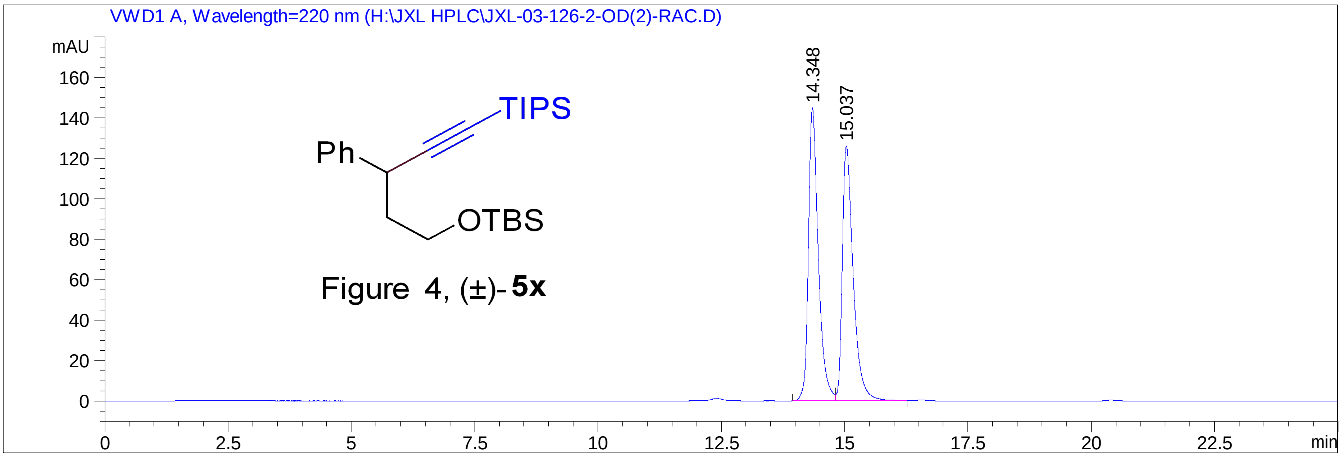

===

Area Percent Report

===

Sorted By : Signal
Multiplier : 1.0000
Dilution : 1.0000
Do not use Multiplier & Dilution Factor with ISTDs

Signal 1: VWD1 A, Wavelength=220 nm

Peak #	RetTime [min]	Type	Width [min]	Area [mAU*s]	Height [mAU]	Area %
1	14.348	BV	0.2091	2025.82507	144.89226	51.1203
2	15.037	VB	0.2303	1937.03552	126.01382	48.8797

Totals : 3962.86060 270.90607

===

*** End of Report ***

Supplementary Fig. 261. HPLC spectra for compound (±)-**5x**

Data File H:\JXL HPLC\JXL-03-135-1-OD(2)-EE.D
Sample Name: JXL-03-135-1-OD(2)

===

Acq. Operator : 系统 Seq. Line : 10
Acq. Instrument : HPLC-1260 Location : 82
Injection Date : 9/3/2020 1:37:40 AM Inj : 1
Inj Volume : 3.000 µl
Different Inj Volume from Sample Entry! Actual Inj Volume : 0.800 µl
Acq. Method : D:\JXL\20200902\YH 2020-09-02 16-57-48\0IPA-30-0.5-1-6-220-JXL.M
Last changed : 9/2/2020 11:43:41 PM by 系统
Analysis Method : E:\DATA\20201027\LC 2020-12-15 14-02-17\6IPA_10_0.8_3.M (Sequence Method)
Last changed : 12/15/2020 10:15:20 PM by SYSTEM
(modified after loading)
Additional Info : Peak(s) manually integrated

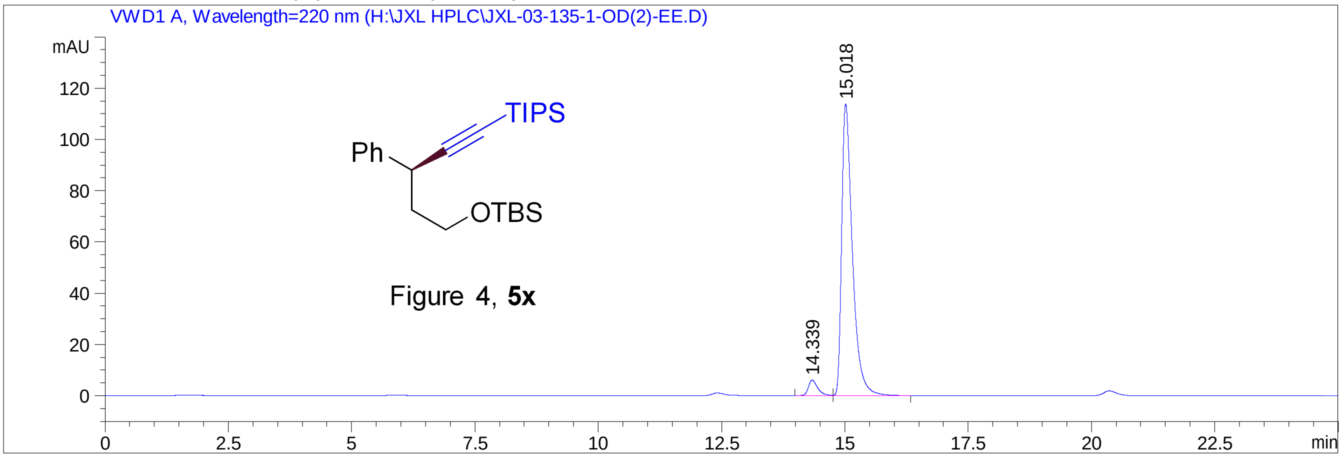

===

Area Percent Report

===

Sorted By : Signal
Multiplier : 1.0000
Dilution : 1.0000
Do not use Multiplier & Dilution Factor with ISTDs

Signal 1: VWD1 A, Wavelength=220 nm

Peak #	RetTime [min]	Type	Width [min]	Area [mAU*s]	Height [mAU]	Area %
1	14.339	BV	0.1966	81.53291	6.11455	4.5107
2	15.018	VB	0.2278	1726.00415	113.87477	95.4893

Totals : 1807.53706 119.98932

===

*** End of Report ***

Supplementary Fig. 262. HPLC spectra for compound **5x**

Data File H:\JXL HPLC\JXL-03-150-4-OD(1)-RAC.D
Sample Name: JXL-03-150-4-OD(1)

===

Acq. Operator : 系统 Seq. Line : 192
Acq. Instrument : HPLC-1260 Location : 95
Injection Date : 9/15/2020 9:49:37 AM Inj : 3
Inj Volume : 3.000 µl
Different Inj Volume from Sample Entry! Actual Inj Volume : 1.000 µl
Acq. Method : D:\G527\FZ\20200822\YH 2020-09-11 23-16-57\1IPA-30-0.5-1-2-220-JXL.M
Last changed : 9/15/2020 9:11:51 AM by 系统
Analysis Method : E:\DATA\20201027\LC 2020-12-15 14-02-17\6IPA_10_0.8_3.M (Sequence Method)
Last changed : 12/15/2020 10:17:14 PM by SYSTEM
(modified after loading)
Additional Info : Peak(s) manually integrated

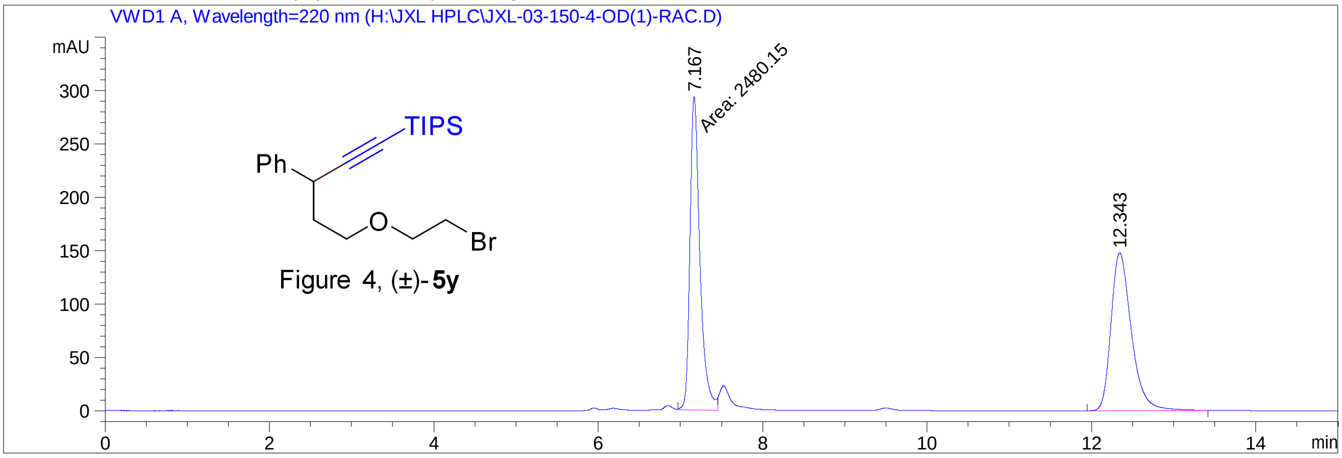

===

Area Percent Report

===

Sorted By : Signal
Multiplier : 1.0000
Dilution : 1.0000
Do not use Multiplier & Dilution Factor with ISTDs

Signal 1: VWD1 A, Wavelength=220 nm

Peak #	RetTime [min]	Type	Width [min]	Area [mAU*s]	Height [mAU]	Area %
1	7.167	FM	0.1409	2480.15137	293.46741	49.4393
2	12.343	BB	0.2629	2536.40210	147.66779	50.5607

Totals : 5016.55347 441.13519

===

*** End of Report ***

Supplementary Fig. 263. HPLC spectra for compound (±)-**5y**

Data File H:\JXL HPLC\JXL-03-153-1-OD(1)-EE.D
Sample Name: JXL-03-153-1-OD(1)

===

Acq. Operator : 系统 Seq. Line : 102
Acq. Instrument : HPLC-1260 Location : 96
Injection Date : 9/13/2020 8:02:04 PM Inj : 1
Inj Volume : 3.000 µl
Different Inj Volume from Sample Entry! Actual Inj Volume : 0.500 µl
Acq. Method : D:\G527\FZ\20200822\YH 2020-09-11 23-16-57\1IPA-30-0.5-1-2-220-JXL.M
Last changed : 9/13/2020 7:58:29 PM by 系统
Analysis Method : E:\DATA\20201027\LC 2020-12-15 14-02-17\6IPA_10_0.8_3.M (Sequence Method)
Last changed : 12/15/2020 10:19:23 PM by SYSTEM
(modified after loading)
Additional Info : Peak(s) manually integrated

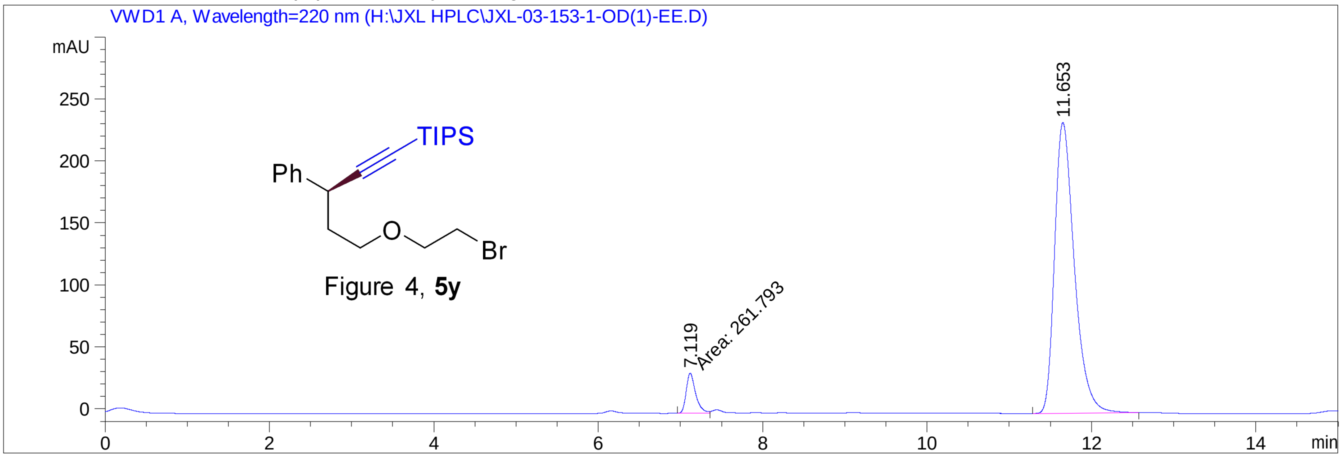

===

Area Percent Report

===

Sorted By : Signal
Multiplier : 1.0000
Dilution : 1.0000
Do not use Multiplier & Dilution Factor with ISTDs

Signal 1: VWD1 A, Wavelength=220 nm

Peak #	RetTime [min]	Type	Width [min]	Area [mAU*s]	Height [mAU]	Area %
1	7.119	FM	0.1345	261.79251	32.42925	6.2619
2	11.653	BB	0.2565	3918.89722	234.48160	93.7381

Totals : 4180.68973 266.91085

===

*** End of Report ***

HPLC1260 12/15/2020 10:19:35 PM SYSTEM Page 1 of 1

Supplementary Fig. 264. HPLC spectra for compound **5y**

Data File H:\JXL HPLC XIUGAI\OnlineEdited--029.D
Sample Name: JXL-03-126-3-OD(2)

===

Acq. Operator : 系统 Seq. Line : 29
Acq. Instrument : HPLC-1260 Location : 89
Injection Date : 12/13/2020 11:16:29 AM Inj : 1
Inj Volume : 3.000 µl
Different Inj Volume from Sample Entry! Actual Inj Volume : 4.000 µl
Acq. Method : D:\G527\FZ\20201127\YH 2020-12-12 20-24-28\0IPA-45-0.5-1-6-220-JXL.M
Last changed : 12/12/2020 9:24:33 PM by 系统
Analysis Method : E:\DATA\20201027\LC 2020-12-18 10-52-42\15IPA_30_8_4.M (Sequence Method)
Last changed : 12/18/2020 3:13:02 PM by SYSTEM
(modified after loading)
Additional Info : Peak(s) manually integrated

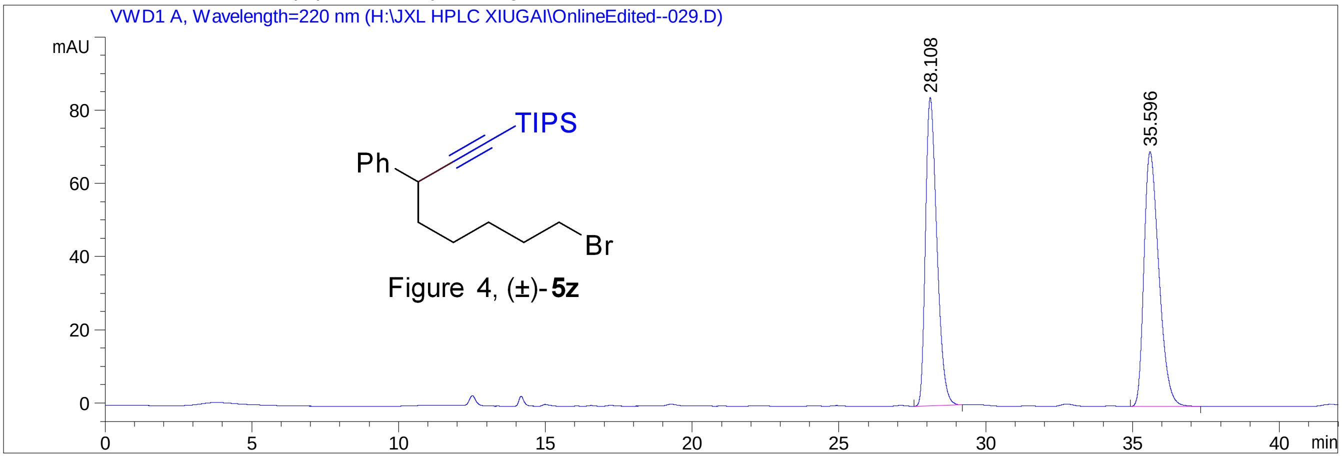

===

Area Percent Report

===

Sorted By : Signal
Multiplier : 1.0000
Dilution : 1.0000
Do not use Multiplier & Dilution Factor with ISTDs

Signal 1: VWD1 A, Wavelength=220 nm

Peak #	RetTime [min]	Type	Width [min]	Area [mAU*s]	Height [mAU]	Area %
1	28.108	BB	0.4169	2276.74316	84.21875	48.0828
2	35.596	BB	0.5323	2458.30249	69.55943	51.9172

Totals : 4735.04565 153.77818

===

*** End of Report ***

Supplementary Fig. 265. HPLC spectra for compound (±)-**5z**

Data File H:\JXL HPLC XIUGAI\OnlineEdited--044.D
Sample Name: JXL-03-138-2-OD(2)

===

Acq. Operator : 系统 Seq. Line : 44
Acq. Instrument : HPLC-1260 Location : 90
Injection Date : 12/13/2020 8:54:29 PM Inj : 1
Inj Volume : 3.000 µl
Acq. Method : D:\G527\FZ\20201127\YH 2020-12-12 20-24-28\0IPA-45-0.5-1-6-220-JXL.M
Last changed : 12/12/2020 9:24:33 PM by 系统
Analysis Method : E:\DATA\20201027\LC 2020-12-15 14-02-17\6IPA_10_0.8_3.M (Sequence Method)
Last changed : 12/15/2020 10:22:57 PM by SYSTEM
(modified after loading)
Additional Info : Peak(s) manually integrated

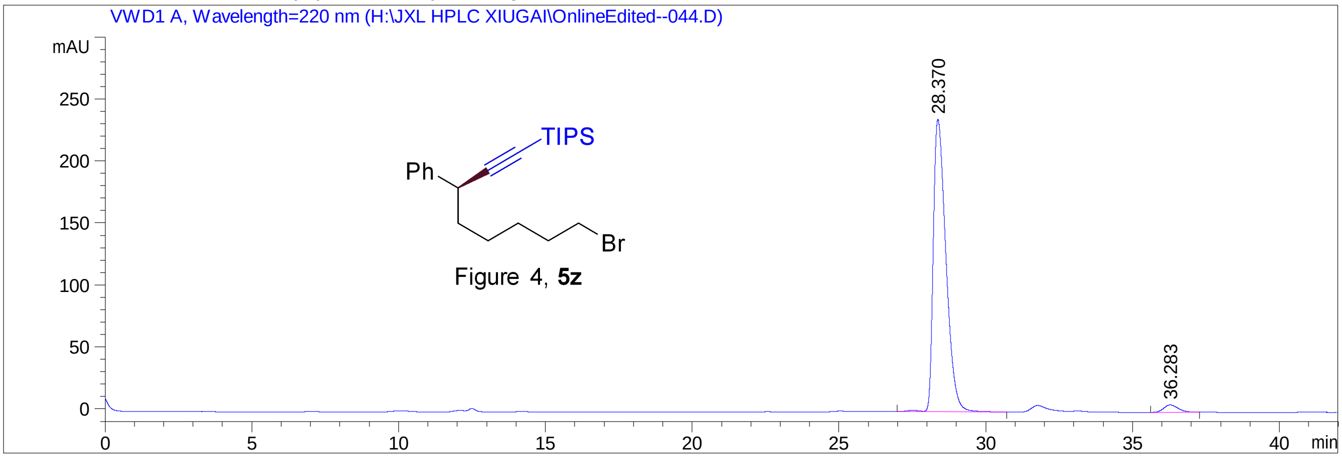

===

Area Percent Report

===

Sorted By : Signal
Multiplier : 1.0000
Dilution : 1.0000
Do not use Multiplier & Dilution Factor with ISTDs

Signal 1: VWD1 A, Wavelength=220 nm

Peak #	RetTime [min]	Type	Width [min]	Area [mAU*s]	Height [mAU]	Area %
1	28.370	VB R	0.4587	7076.32129	236.06902	97.1948
2	36.283	BB	0.4036	204.23074	5.95462	2.8052

Totals : 7280.55203 242.02364

===

*** End of Report ***

Supplementary Fig. 266. HPLC spectra for compound **5z**

Data File H:\JXL HPLC\JXL-02-164-4-AD(2)-RAC.D
Sample Name: JXL-02-164-4-AD(2)

===

Acq. Operator : 系统 Seq. Line : 6
Acq. Instrument : HPLC-1260 Location : 81
Injection Date : 9/20/2020 5:35:17 PM Inj : 1
Inj Volume : 3.000 µl
Different Inj Volume from Sample Entry! Actual Inj Volume : 2.000 µl
Acq. Method : D:\JXL\20200920\YH 2020-09-20 14-51-06\0IPA-45-0.5-1-6-220-JXL.M
Last changed : 9/20/2020 3:40:59 PM by 系统
Analysis Method : E:\DATA\20210127\LC 2021-01-27 19-42-16\10IPA_20_1.0_3.MZWJ.M (Sequence Method)
Last changed : 1/29/2021 4:34:03 PM by SYSTEM
(modified after loading)

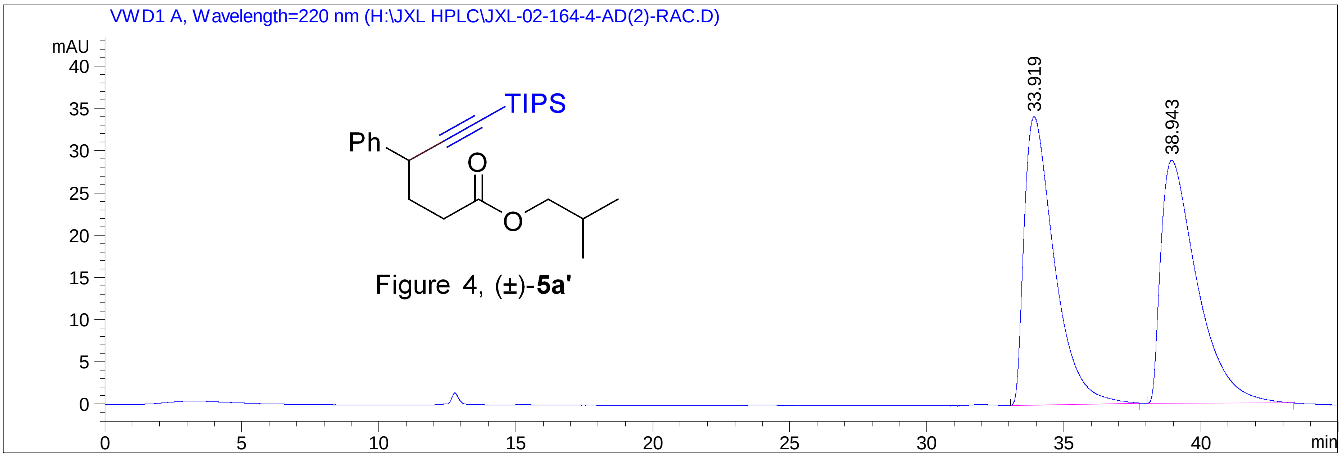

===

Area Percent Report

===

Sorted By : Signal
Multiplier : 1.0000
Dilution : 1.0000
Do not use Multiplier & Dilution Factor with ISTDs

Signal 1: VWD1 A, Wavelength=220 nm

Peak #	RetTime [min]	Type	Width [min]	Area [mAU*s]	Height [mAU]	Area %
1	33.919	BB	0.9033	2623.93433	34.13412	49.6876
2	38.943	BB	1.0816	2656.93164	28.77937	50.3124

Totals : 5280.86597 62.91348

===

*** End of Report ***

Supplementary Fig. 267. HPLC spectra for compound (±)-**5a'**

Data File H:\JXL HPLC\OnlineEdited--008.D
Sample Name: JXL-03-116-1-AD(2)

===

Acq. Operator : 系统 Seq. Line : 8
Acq. Instrument : HPLC-1260 Location : 92
Injection Date : 1/23/2021 1:14:05 PM Inj : 1
Inj Volume : 3.000 µl
Different Inj Volume from Sample Entry! Actual Inj Volume : 4.000 µl
Acq. Method : D:\JXL\20210123\YH 2021-01-23 09-26-26\0IPA-45-0.5-1-2-220-JXL.M
Last changed : 1/23/2021 9:58:41 AM by 系统
Analysis Method : E:\DATA\20210127\LC 2021-01-27 19-42-16\10IPA_20_1.0_3.MZWJ.M (Sequence Method)
Last changed : 1/29/2021 4:32:32 PM by SYSTEM
(modified after loading)
Additional Info : Peak(s) manually integrated

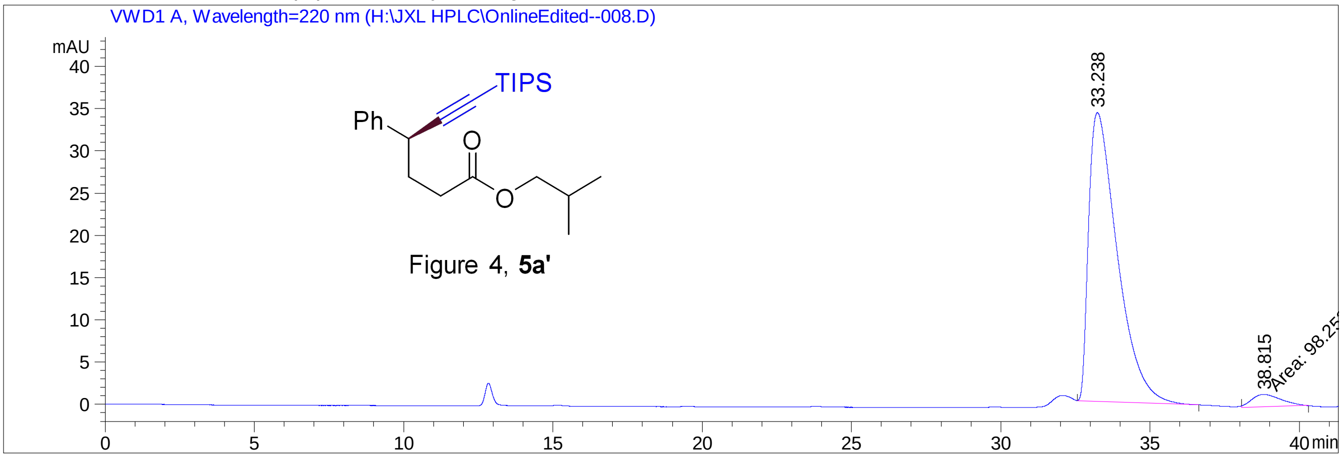

===

Area Percent Report

===

Sorted By : Signal
Multiplier : 1.0000
Dilution : 1.0000
Do not use Multiplier & Dilution Factor with ISTDs

Signal 1: VWD1 A, Wavelength=220 nm

Peak #	RetTime [min]	Type	Width [min]	Area [mAU*s]	Height [mAU]	Area %
1	33.238	BB	0.7824	2280.43457	34.18036	95.8695
2	38.815	MM	1.1224	98.25251	1.45902	4.1305

Totals : 2378.68708 35.63938

===

*** End of Report ***

Supplementary Fig. 268. HPLC spectra for compound **5a'**

Data File H:\JXL HPLC XIUGAI\OnlineEdited--010.D
Sample Name: JXL-03-153-5-OD(1)

===

Acq. Operator : 系统 Seq. Line : 10
Acq. Instrument : HPLC-1260 Location : 95
Injection Date : 12/12/2020 11:47:56 PM Inj : 1
Inj Volume : 3.000 µl
Acq. Method : D:\G527\FZ\20201127\YH 2020-12-12 20-24-28\1IPA-20-0.5-1-6-220-JXL.M
Last changed : 12/12/2020 11:16:10 PM by 系统
Analysis Method : E:\DATA\20201027\LC 2020-12-15 14-02-17\6IPA_10_0.8_3.M (Sequence Method)
Last changed : 12/15/2020 10:41:58 PM by SYSTEM
(modified after loading)
Additional Info : Peak(s) manually integrated

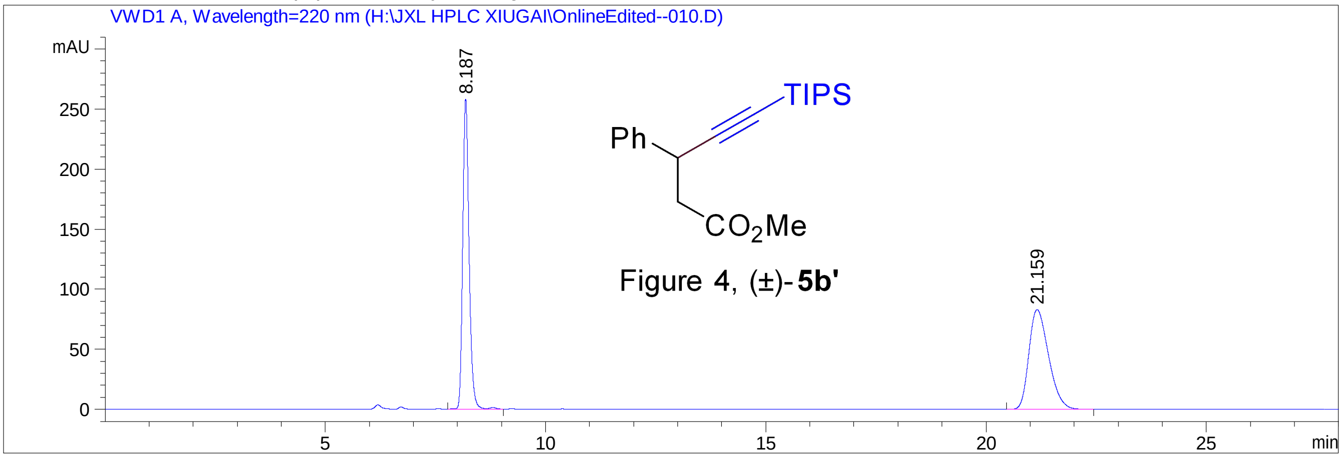

===

Area Percent Report

===

Sorted By : Signal
Multiplier : 1.0000
Dilution : 1.0000
Do not use Multiplier & Dilution Factor with ISTDs

Signal 1: VWD1 A, Wavelength=220 nm

Peak #	RetTime [min]	Type	Width [min]	Area [mAU*s]	Height [mAU]	Area %
1	8.187	BV R	0.1524	2576.23950	258.17044	49.9962
2	21.159	BB	0.4717	2576.62598	83.11460	50.0038

Totals : 5152.86548 341.28504

===

*** End of Report ***

Supplementary Fig. 269. HPLC spectra for compound (±)-**5b'**

Data File H:\JXL HPLC XIUGAI\OnlineEdited--011.D
Sample Name: JXL-03-158-3-OD(1)

===

```
Acq. Operator   : 系统                          Seq. Line :  11
Acq. Instrument : HPLC-1260                      Location :   96
Injection Date  : 12/13/2020 12:17:20 AM              Inj :   1
                                               Inj Volume : 3.000 µl
Acq. Method     : D:\G527\FZ\20201127\YH 2020-12-12 20-24-28\1IPA-20-0.5-1-6-220-JXL.M
Last changed    : 12/12/2020 11:16:10 PM by 系统
Analysis Method : E:\DATA\20201027\LC 2020-12-15 14-02-17\6IPA_10_0.8_3.M (Sequence Method)
Last changed    : 12/15/2020 10:42:45 PM by SYSTEM
                  (modified after loading)
Additional Info : Peak(s) manually integrated
```

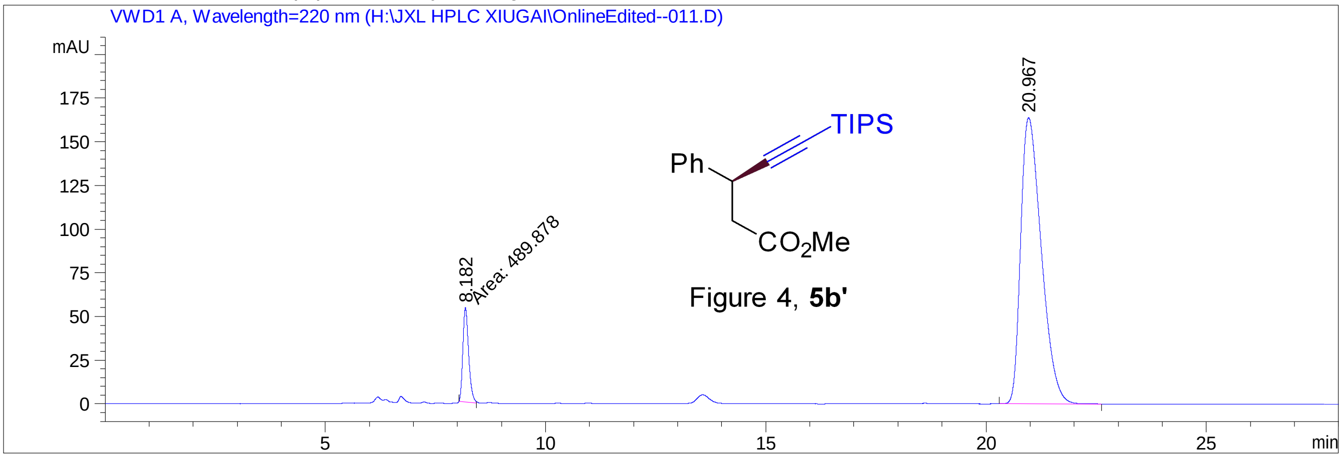

===

Area Percent Report

===

Sorted By : Signal
Multiplier : 1.0000
Dilution : 1.0000
Do not use Multiplier & Dilution Factor with ISTDs

Signal 1: VWD1 A, Wavelength=220 nm

Peak #	RetTime [min]	Type	Width [min]	Area [mAU*s]	Height [mAU]	Area %
1	8.182	PM	0.1516	489.87790	53.86531	8.4159
2	20.967	BB	0.4982	5331.00342	163.80722	91.5841

Totals : 5820.88132 217.67253

===

*** End of Report ***

Supplementary Fig. 270. HPLC spectra for compound **5b'**

Data File H:\JXL HPLC XIUGAI\OnlineEdited--006.D
Sample Name: JXL-03-131-5-OD(1)

===

Acq. Operator : 系统 Seq. Line : 6
Acq. Instrument : HPLC-1260 Location : 93
Injection Date : 12/12/2020 10:06:17 PM Inj : 1
Inj Volume : 3.000 µl
Different Inj Volume from Sample Entry! Actual Inj Volume : 1.000 µl
Acq. Method : D:\G527\FZ\20201127\YH 2020-12-12 20-24-28\1IPA-20-0.5-1-6-220-JXL.M
Last changed : 12/12/2020 10:25:23 PM by 系统
(modified after loading)
Analysis Method : E:\DATA\20201027\LC 2020-12-15 14-02-17\6IPA_10_0.8_3.M (Sequence Method)
Last changed : 12/15/2020 10:50:20 PM by SYSTEM
(modified after loading)
Additional Info : Peak(s) manually integrated

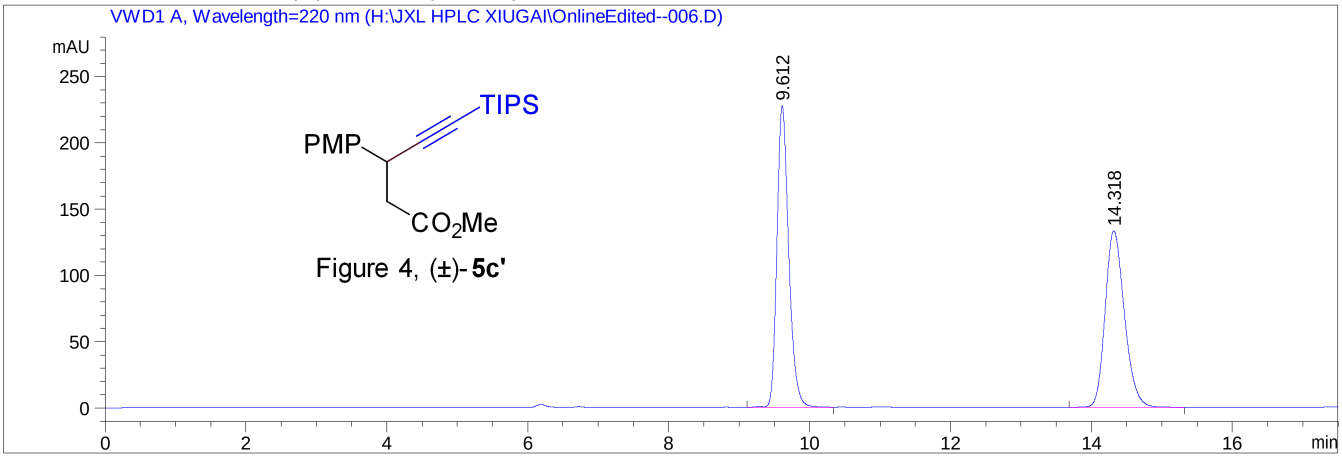

===

Area Percent Report

===

Sorted By : Signal
Multiplier : 1.0000
Dilution : 1.0000
Do not use Multiplier & Dilution Factor with ISTDs

Signal 1: VWD1 A, Wavelength=220 nm

Peak #	RetTime [min]	Type	Width [min]	Area [mAU*s]	Height [mAU]	Area %
1	9.612	VB R	0.1765	2641.20361	227.43277	51.0177
2	14.318	BB	0.2906	2535.82764	133.21794	48.9823

Totals : 5177.03125 360.65071

===

*** End of Report ***

Supplementary Fig. 271. HPLC spectra for compound (±)-**5c'**

Data File H:\JXL HPLC XIUGAI\OnlineEdited--007.D
Sample Name: JXL-03-137-1-OD(1)

===

Acq. Operator : 系统 Seq. Line : 7
Acq. Instrument : HPLC-1260 Location : 94
Injection Date : 12/12/2020 10:27:40 PM Inj : 1
Inj Volume : 3.000 µl
Different Inj Volume from Sample Entry! Actual Inj Volume : 1.000 µl
Acq. Method : D:\G527\FZ\20201127\YH 2020-12-12 20-24-28\1IPA-20-0.5-1-6-220-JXL.M
Last changed : 12/12/2020 10:25:23 PM by 系统
Analysis Method : E:\DATA\20201027\LC 2020-12-15 14-02-17\6IPA_10_0.8_3.M (Sequence Method)
Last changed : 12/15/2020 10:51:28 PM by SYSTEM
(modified after loading)
Additional Info : Peak(s) manually integrated

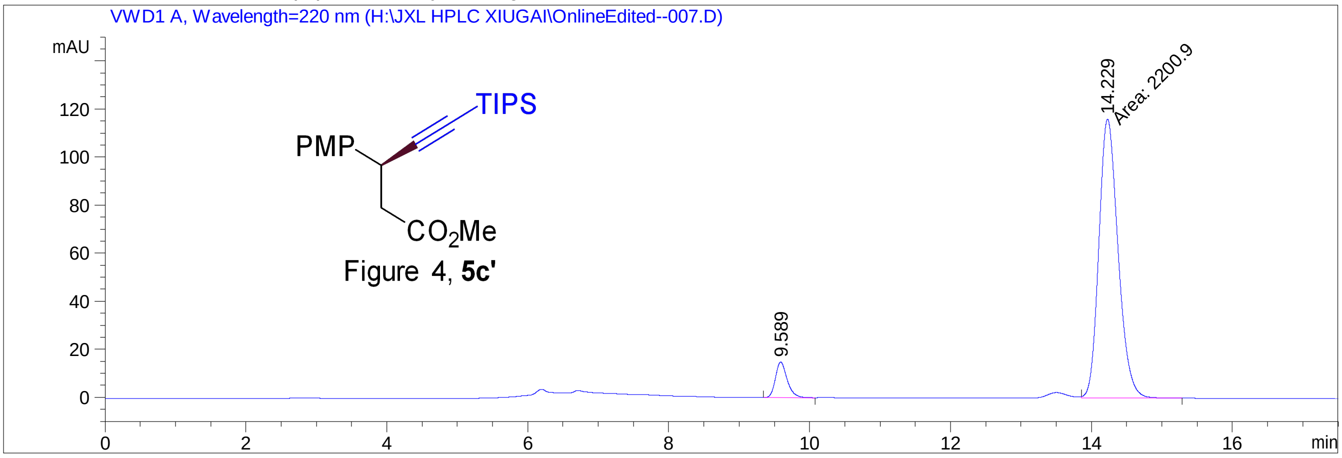

===

Area Percent Report

===

Sorted By : Signal
Multiplier : 1.0000
Dilution : 1.0000
Do not use Multiplier & Dilution Factor with ISTDs

Signal 1: VWD1 A, Wavelength=220 nm

Peak #	RetTime [min]	Type	Width [min]	Area [mAU*s]	Height [mAU]	Area %
1	9.589	BB	0.1754	170.47371	14.79596	7.1888
2	14.229	FM	0.3158	2200.89990	116.16302	92.8112

Totals : 2371.37361 130.95898

===

*** End of Report ***

Supplementary Fig. 272. HPLC spectra for compound **5c'**

Data File H:\JXL HPLC XIUGAI\OnlineEdited--032.D
Sample Name: JXL-02-95-3-OD(2)

===

Acq. Operator : 系统 Seq. Line : 32
Acq. Instrument : HPLC-1260 Location : 71
Injection Date : 12/13/2020 1:51:07 PM Inj : 1
Inj Volume : 3.000 µl
Different Inj Volume from Sample Entry! Actual Inj Volume : 2.000 µl
Acq. Method : D:\G527\FZ\20201127\YH 2020-12-12 20-24-28\0IPA-40-0.5-1-6-220-JXL.M
Last changed : 12/12/2020 9:26:27 PM by 系统
Analysis Method : E:\DATA\20201027\LC 2020-12-18 10-52-42\15IPA_30_8_4.M (Sequence Method)
Last changed : 12/18/2020 3:18:06 PM by SYSTEM
(modified after loading)
Additional Info : Peak(s) manually integrated

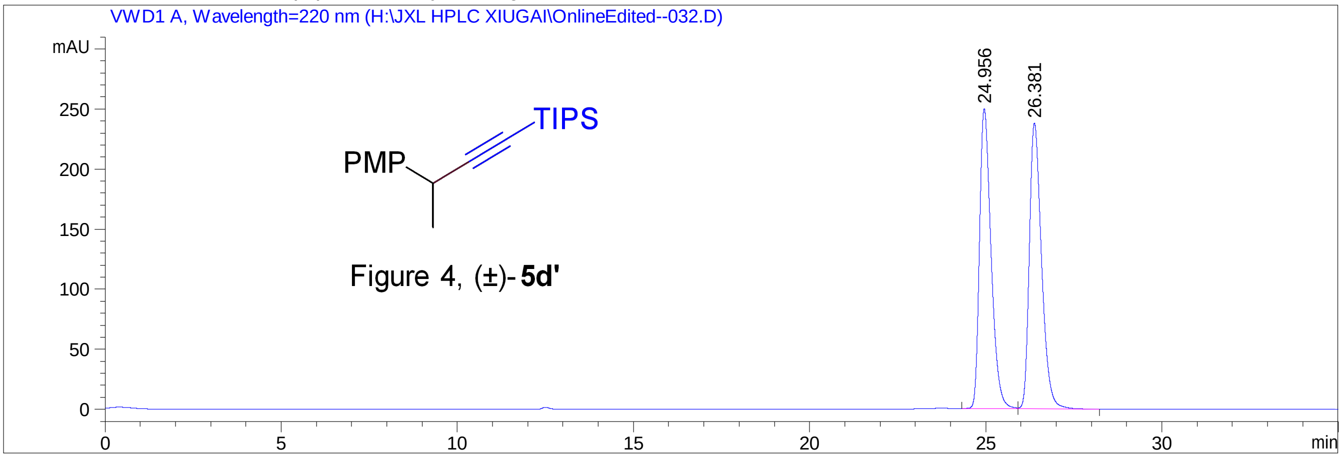

===
Area Percent Report
===

Sorted By : Signal
Multiplier : 1.0000
Dilution : 1.0000
Do not use Multiplier & Dilution Factor with ISTDs

Signal 1: VWD1 A, Wavelength=220 nm

Peak #	RetTime [min]	Type	Width [min]	Area [mAU*s]	Height [mAU]	Area %
1	24.956	BV	0.3465	5686.15039	249.83354	49.7768
2	26.381	VB	0.3651	5737.15039	238.07536	50.2232

Totals : 1.14233e4 487.90891

===
*** End of Report ***

Supplementary Fig. 273. HPLC spectra for compound (±)-**5d'**

Data File H:\JXL HPLC XIUGAI\OnlineEdited--041.D
Sample Name: JXL-03-132-1-OD(2)

===

Acq. Operator : 系统 Seq. Line : 41
Acq. Instrument : HPLC-1260 Location : 72
Injection Date : 12/13/2020 7:34:17 PM Inj : 1
Inj Volume : 3.000 µl
Different Inj Volume from Sample Entry! Actual Inj Volume : 1.000 µl
Acq. Method : D:\G527\FZ\20201127\YH 2020-12-12 20-24-28\0IPA-40-0.5-1-6-220-JXL.M
Last changed : 12/13/2020 8:07:11 PM by 系统
(modified after loading)
Analysis Method : E:\DATA\20201027\LC 2020-12-15 14-02-17\6IPA_10_0.8_3.M (Sequence Method)
Last changed : 12/15/2020 10:54:24 PM by SYSTEM
(modified after loading)
Additional Info : Peak(s) manually integrated

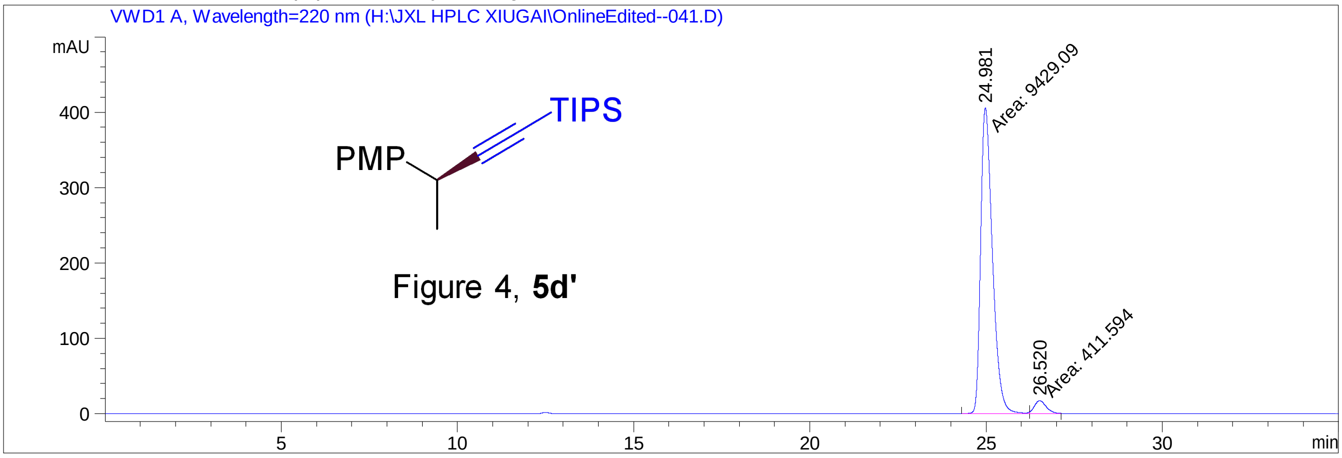

===

Area Percent Report

===

Sorted By : Signal
Multiplier : 1.0000
Dilution : 1.0000
Do not use Multiplier & Dilution Factor with ISTDs

Signal 1: VWD1 A, Wavelength=220 nm

Peak #	RetTime [min]	Type	Width [min]	Area [mAU*s]	Height [mAU]	Area %
1	24.981	MF	0.3870	9429.09473	406.02753	95.8174
2	26.520	FM	0.3954	411.59427	17.34928	4.1826

Totals : 9840.68900 423.37681

===

*** End of Report ***

Supplementary Fig. 274. HPLC spectra for compound **5d'**

Data File H:\HPLC20210310\OnlineEdited--027.D
Sample Name: JXL-04-125-3-1-OD(2)

===

Acq. Operator : 系统 Seq. Line : 27
Acq. Instrument : HPLC-1260 Location : 81
Injection Date : 3/5/2021 8:58:56 PM Inj : 1
Inj Volume : 3.000 µl
Different Inj Volume from Sample Entry! Actual Inj Volume : 2.000 µl
Acq. Method : D:\ZHH\20210304\YH 2021-03-05 08-58-35\0IPA-45-0.5-1-2-220-JXL.M
Last changed : 3/5/2021 9:34:52 PM by 系统
(modified after loading)
Analysis Method : E:\DATA\20210310\LC 2021-03-10 20-00-42\0IPA_30_0.5_2 H.M (Sequence Method)
Last changed : 3/10/2021 10:33:44 PM by SYSTEM
(modified after loading)
Additional Info : Peak(s) manually integrated

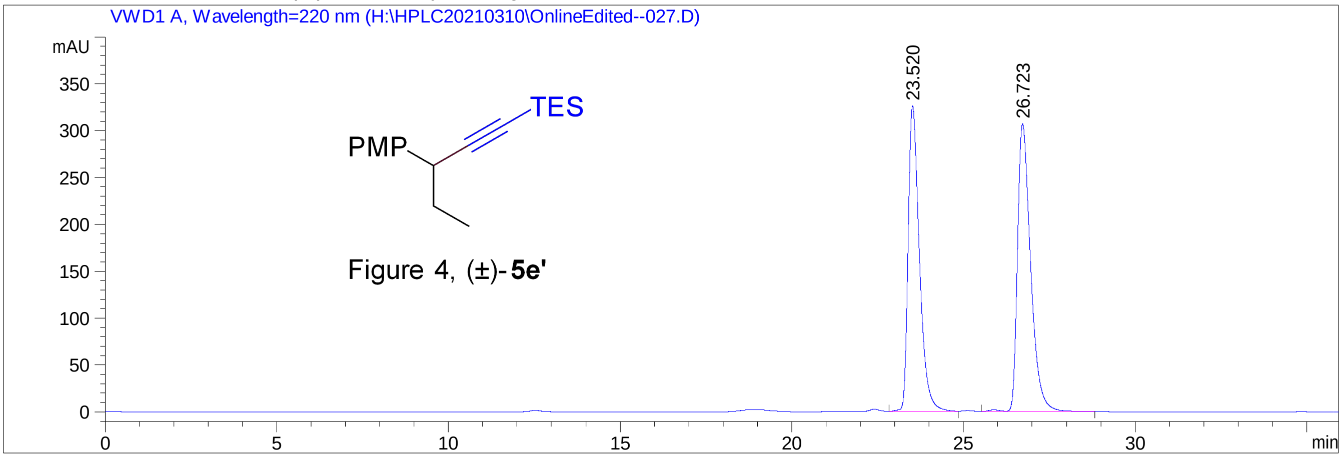

===

Area Percent Report

===

Sorted By : Signal
Multiplier : 1.0000
Dilution : 1.0000
Do not use Multiplier & Dilution Factor with ISTDs

Signal 1: VWD1 A, Wavelength=220 nm

Peak #	RetTime [min]	Type	Width [min]	Area [mAU*s]	Height [mAU]	Area %
1	23.520	BB	0.3475	7443.00439	325.79547	47.6595
2	26.723	VB R	0.3985	8174.02979	307.09445	52.3405

Totals : 1.56170e4 632.88992

===

*** End of Report ***

Supplementary Fig. 275. HPLC spectra for compound (±)-**5e'**

Data File H:\HPLC20210310\OnlineEdited--024.D
Sample Name: JXL-04-124-3-OD(2)

===

Acq. Operator : 系统 Seq. Line : 24
Acq. Instrument : HPLC-1260 Location : 93
Injection Date : 3/5/2021 7:09:43 PM Inj : 1
Inj Volume : 3.000 µl
Different Inj Volume from Sample Entry! Actual Inj Volume : 0.200 µl
Acq. Method : D:\ZHH\20210304\YH 2021-03-05 08-58-35\0IPA-45-0.5-1-2-220-JXL.M
Last changed : 3/5/2021 7:39:28 PM by 系统
(modified after loading)
Analysis Method : E:\DATA\20210310\LC 2021-03-10 20-00-42\0IPA_30_0.5_2 H.M (Sequence Method)
Last changed : 3/10/2021 10:35:06 PM by SYSTEM
(modified after loading)
Additional Info : Peak(s) manually integrated

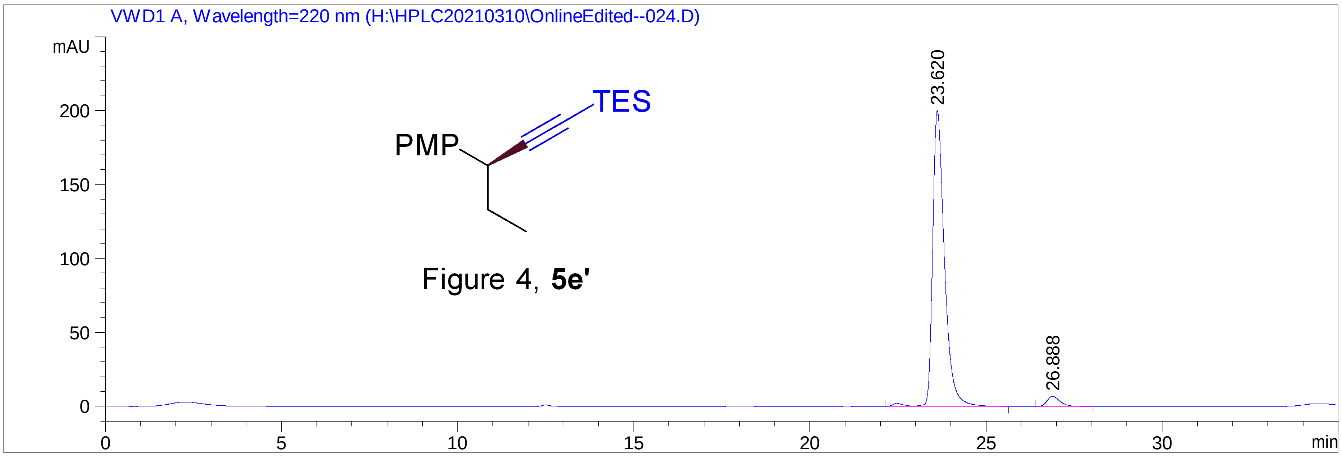

===

Area Percent Report

===

Sorted By : Signal
Multiplier : 1.0000
Dilution : 1.0000
Do not use Multiplier & Dilution Factor with ISTDs

Signal 1: VWD1 A, Wavelength=220 nm

Peak #	RetTime [min]	Type	Width [min]	Area [mAU*s]	Height [mAU]	Area %
1	23.620	VB R	0.3499	4690.87891	200.07715	96.2894
2	26.888	BB	0.3508	180.76852	6.95428	3.7106

Totals : 4871.64743 207.03143

===

*** End of Report ***

Supplementary Fig. 276. HPLC spectra for compound **5e'**

```
Data File H:\HPLC20210310\OnlineEdited--025.D
Sample Name: JXL-04-125-5-1-OD(2)

=====================================================================
Acq. Operator   : 系统                            Seq. Line :  25
Acq. Instrument : HPLC-1260                        Location :   82
Injection Date  : 3/5/2021 7:46:06 PM                   Inj :   1
                                                 Inj Volume : 3.000 µl
Different Inj Volume from Sample Entry!  Actual Inj Volume : 0.700 µl
Acq. Method     : D:\ZHH\20210304\YH 2021-03-05 08-58-35\0IPA-45-0.5-1-2-220-JXL.M
Last changed    : 3/5/2021 7:39:28 PM by 系统
Analysis Method : E:\DATA\20210310\LC 2021-03-10 20-00-42\0IPA_30_0.5_2 H.M (Sequence Method)
Last changed    : 3/10/2021 10:36:25 PM by SYSTEM
                  (modified after loading)
```

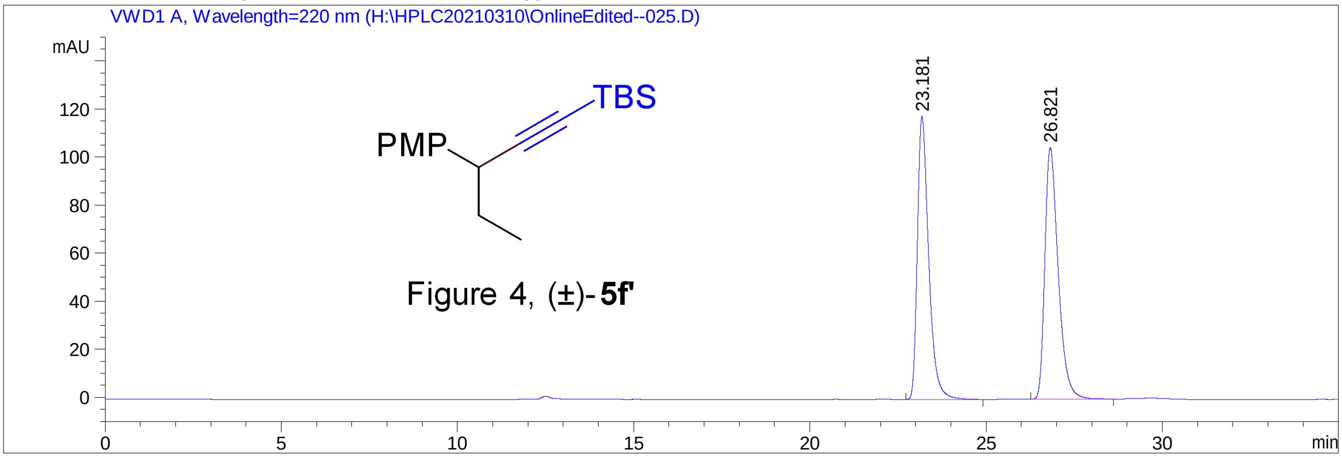


```
=====================================================================
                         Area Percent Report
=====================================================================

Sorted By             :      Signal
Multiplier            :      1.0000
Dilution              :      1.0000
Do not use Multiplier & Dilution Factor with ISTDs


Signal 1: VWD1 A, Wavelength=220 nm
```

Peak #	RetTime [min]	Type	Width [min]	Area [mAU*s]	Height [mAU]	Area %
1	23.181	BB	0.3379	2628.45190	117.75947	48.7145
2	26.821	BB	0.4016	2767.17554	104.77796	51.2855

Totals : 5395.62744 222.53743

```
=====================================================================
                         *** End of Report ***
```

HPLC1260 3/10/2021 10:36:36 PM SYSTEM   Page 1 of 1

Supplementary Fig. 277. HPLC spectra for compound (±)-**5f'**

Data File H:\HPLC20210310\OnlineEdited--026.D
Sample Name: JXL-04-124-7-OD(2)

===

Acq. Operator : 系统 Seq. Line : 26
Acq. Instrument : HPLC-1260 Location : 94
Injection Date : 3/5/2021 8:22:32 PM Inj : 1
Inj Volume : 3.000 µl
Different Inj Volume from Sample Entry! Actual Inj Volume : 0.300 µl
Acq. Method : D:\ZHH\20210304\YH 2021-03-05 08-58-35\0IPA-45-0.5-1-2-220-JXL.M
Last changed : 3/5/2021 7:39:28 PM by 系统
Analysis Method : E:\DATA\20210310\LC 2021-03-10 20-00-42\0IPA_30_0.5_2 H.M (Sequence Method)
Last changed : 3/10/2021 10:37:09 PM by SYSTEM
(modified after loading)

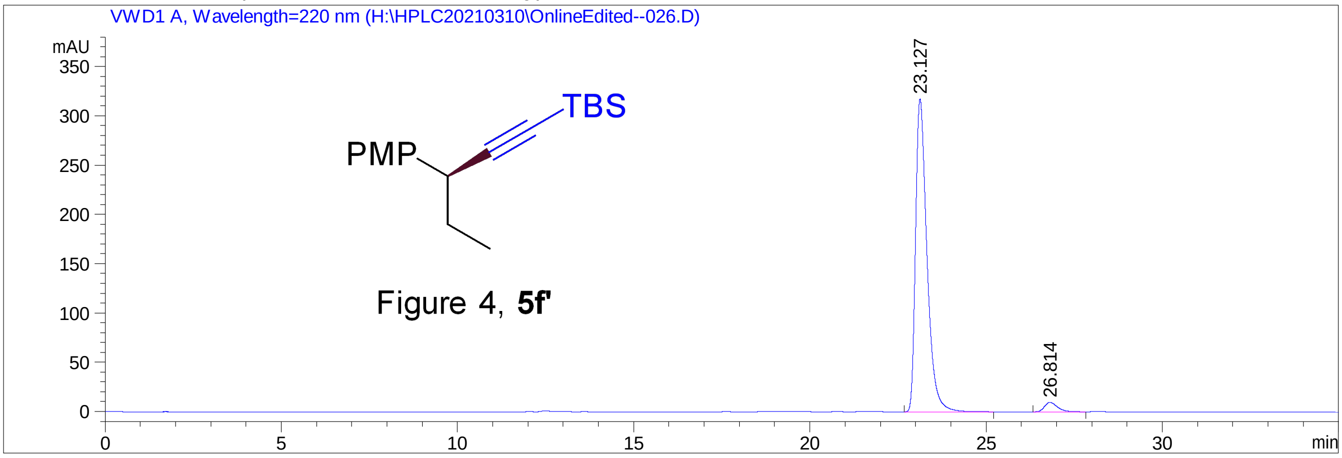

===

Area Percent Report

===

Sorted By : Signal
Multiplier : 1.0000
Dilution : 1.0000
Do not use Multiplier & Dilution Factor with ISTDs

Signal 1: VWD1 A, Wavelength=220 nm

Peak #	RetTime [min]	Type	Width [min]	Area [mAU*s]	Height [mAU]	Area %
1	23.127	BB	0.3407	7151.65332	317.55710	96.6746
2	26.814	BB	0.3599	245.99886	9.61679	3.3254

Totals : 7397.65218 327.17389

===

*** End of Report ***

HPLC1260 3/10/2021 10:37:16 PM SYSTEM Page 1 of 1

Supplementary Fig. 278. HPLC spectra for compound **5f'**

Data File H:\JXL HPLC XIUGAI\OnlineEdited--015.D
Sample Name: JXL-03-148-1-OD(1)

===
Acq. Operator : 系统 Seq. Line : 15
Acq. Instrument : HPLC-1260 Location : 97
Injection Date : 12/13/2020 2:21:41 AM Inj : 1
Inj Volume : 3.000 µl
Different Inj Volume from Sample Entry! Actual Inj Volume : 8.000 µl
Acq. Method : D:\G527\FZ\20201127\YH 2020-12-12 20-24-28\0IPA-40-0.8-1-6-220-JXL.M
Last changed : 12/13/2020 1:42:46 AM by 系统
Analysis Method : E:\DATA\20201027\LC 2020-12-18 10-52-42\15IPA_30_8_4.M (Sequence Method)
Last changed : 12/18/2020 3:22:43 PM by SYSTEM
(modified after loading)
Additional Info : Peak(s) manually integrated

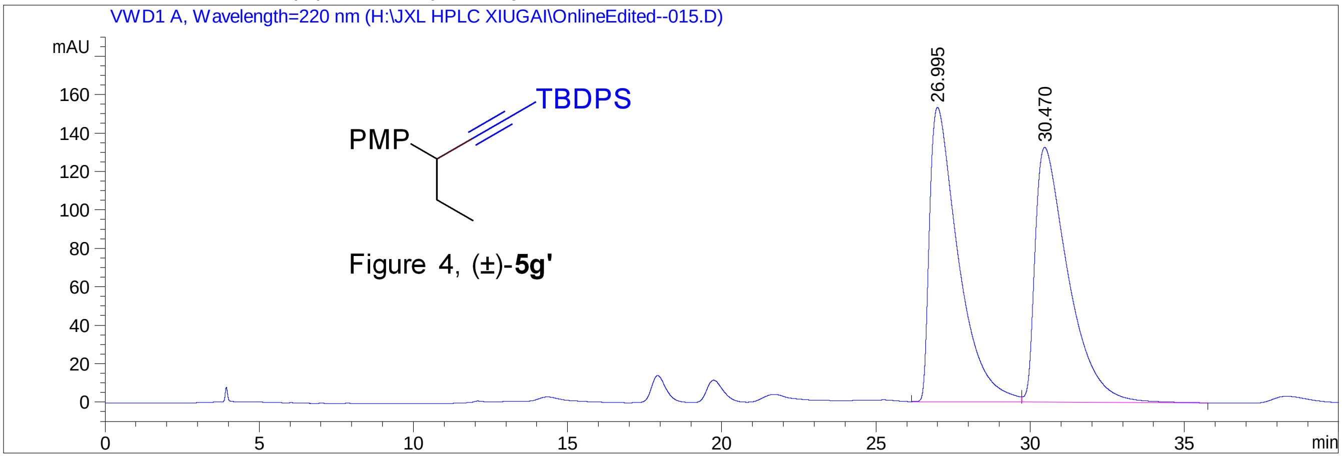

===
Area Percent Report
===

Sorted By : Signal
Multiplier : 1.0000
Dilution : 1.0000
Do not use Multiplier & Dilution Factor with ISTDs

Signal 1: VWD1 A, Wavelength=220 nm

Peak #	RetTime [min]	Type	Width [min]	Area [mAU*s]	Height [mAU]	Area %
1	26.995	BV	0.9186	9964.35254	153.24321	50.1070
2	30.470	VB	1.0340	9921.79297	132.51718	49.8930

Totals : 1.98861e4 285.76039

===
*** End of Report ***

Supplementary Fig. 279. HPLC spectra for compound (±)-**5g'**

Data File H:\JXL HPLC XIUGAI\OnlineEdited--014.D
Sample Name: JXL-03-154-2-OD(1)

===

Acq. Operator : 系统 Seq. Line : 14
Acq. Instrument : HPLC-1260 Location : 98
Injection Date : 12/13/2020 1:40:07 AM Inj : 1
Inj Volume : 3.000 µl
Different Inj Volume from Sample Entry! Actual Inj Volume : 5.000 µl
Acq. Method : D:\G527\FZ\20201127\YH 2020-12-12 20-24-28\0IPA-40-0.8-1-6-220-JXL.M
Last changed : 12/13/2020 1:42:46 AM by 系统
(modified after loading)
Analysis Method : E:\DATA\20201027\LC 2020-12-15 14-02-17\6IPA_10_0.8_3.M (Sequence Method)
Last changed : 12/15/2020 11:00:35 PM by SYSTEM
(modified after loading)
Additional Info : Peak(s) manually integrated

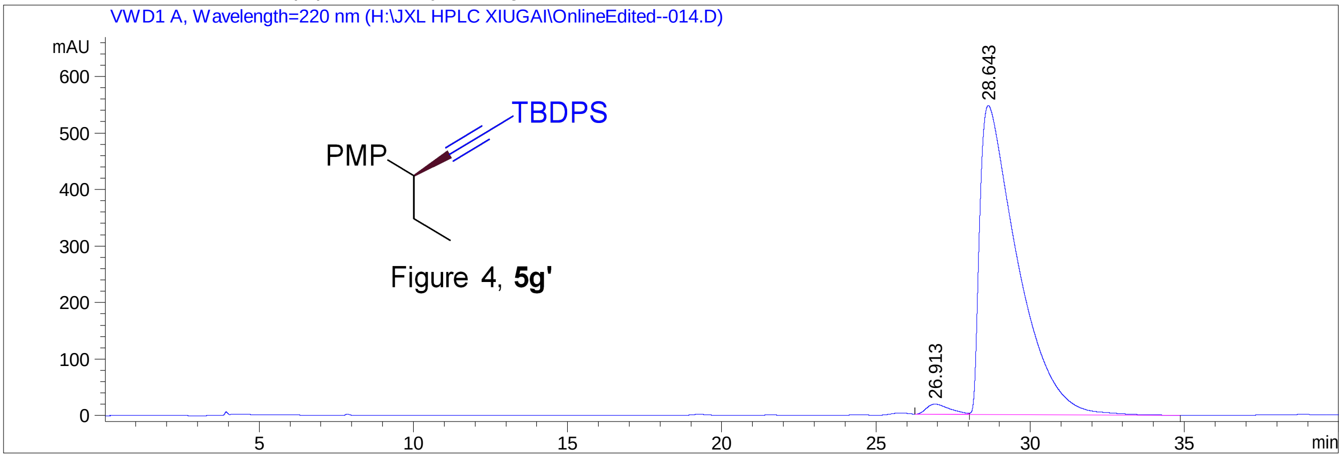

===
Area Percent Report
===

Sorted By : Signal
Multiplier : 1.0000
Dilution : 1.0000
Do not use Multiplier & Dilution Factor with ISTDs

Signal 1: VWD1 A, Wavelength=220 nm

Peak #	RetTime [min]	Type	Width [min]	Area [mAU*s]	Height [mAU]	Area %
1	26.913	BV E	0.6800	925.29993	17.77107	1.9496
2	28.643	VB R	1.1901	4.65365e4	546.39746	98.0504

Totals : 4.74618e4 564.16853

===
*** End of Report ***

Supplementary Fig. 280. HPLC spectra for compound **5g'**

Data File H:\JXL HPLC XIUGAI\OnlineEdited--027.D
Sample Name: JXL-03-126-7-OD(2)

===

Acq. Operator : 系统 Seq. Line : 27
Acq. Instrument : HPLC-1260 Location : 75
Injection Date : 12/13/2020 9:15:35 AM Inj : 1
Inj Volume : 3.000 µl
Acq. Method : D:\G527\FZ\20201127\YH 2020-12-12 20-24-28\0IPA-60-0.5-1-6-220-JXL.M
Last changed : 12/13/2020 10:16:07 AM by 系统
(modified after loading)
Analysis Method : C:\CHEM32\1\METHODS\15IPA_30_8_4.M
Last changed : 12/19/2020 9:38:39 AM by SYSTEM
(modified after loading)
Additional Info : Peak(s) manually integrated

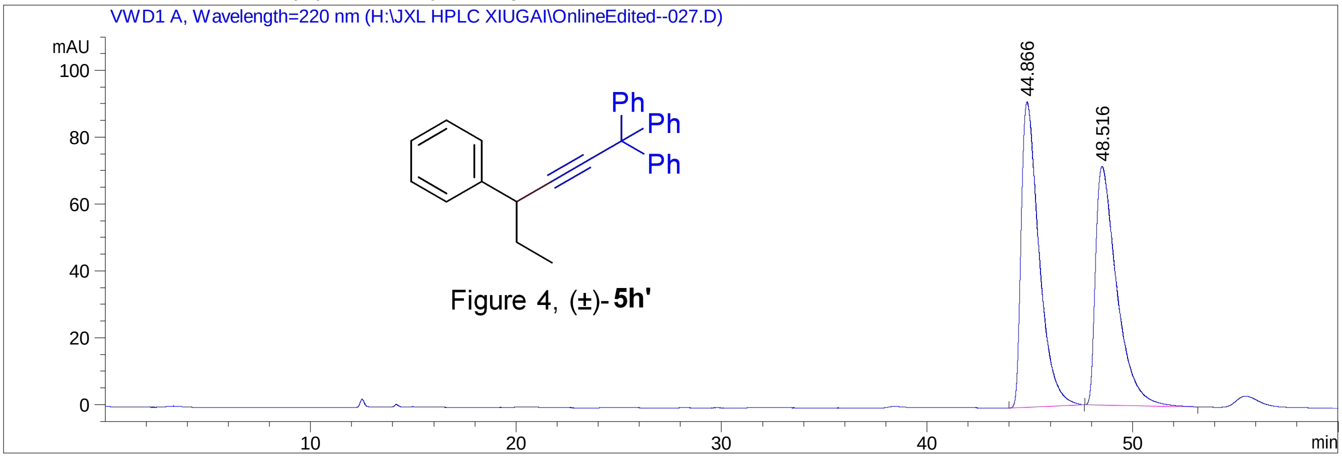

===

Area Percent Report

===

Sorted By : Signal
Multiplier : 1.0000
Dilution : 1.0000
Do not use Multiplier & Dilution Factor with ISTDs

Signal 1: VWD1 A, Wavelength=220 nm

Peak #	RetTime [min]	Type	Width [min]	Area [mAU*s]	Height [mAU]	Area %
1	44.866	BB	0.8906	5530.87402	91.31907	51.8735
2	48.516	BB	1.0003	5131.35156	71.41467	48.1265

Totals : 1.06622e4 162.73374

===

*** End of Report ***

HPLC1260 12/19/2020 9:38:53 AM SYSTEM Page 1 of 1

Supplementary Fig. 281. HPLC spectra for compound (±)-**5h'**

Data File H:\JXL HPLC XIUGAI\OnlineEdited--038.D
Sample Name: JXL-03-145-1-OD(2)

===

Acq. Operator : 系统 Seq. Line : 38
Acq. Instrument : HPLC-1260 Location : 76
Injection Date : 12/13/2020 5:29:55 PM Inj : 1
Inj Volume : 3.000 µl
Different Inj Volume from Sample Entry! Actual Inj Volume : 6.000 µl
Acq. Method : D:\G527\FZ\20201127\YH 2020-12-12 20-24-28\0IPA-60-0.5-1-6-220-JXL.M
Last changed : 12/13/2020 11:18:35 AM by 系统
Analysis Method : E:\DATA\20201027\LC 2020-12-15 14-02-17\10IPA_20_1.0_4.ZWJ.M (Sequence Method)
Last changed : 12/16/2020 10:24:22 AM by SYSTEM
(modified after loading)
Additional Info : Peak(s) manually integrated

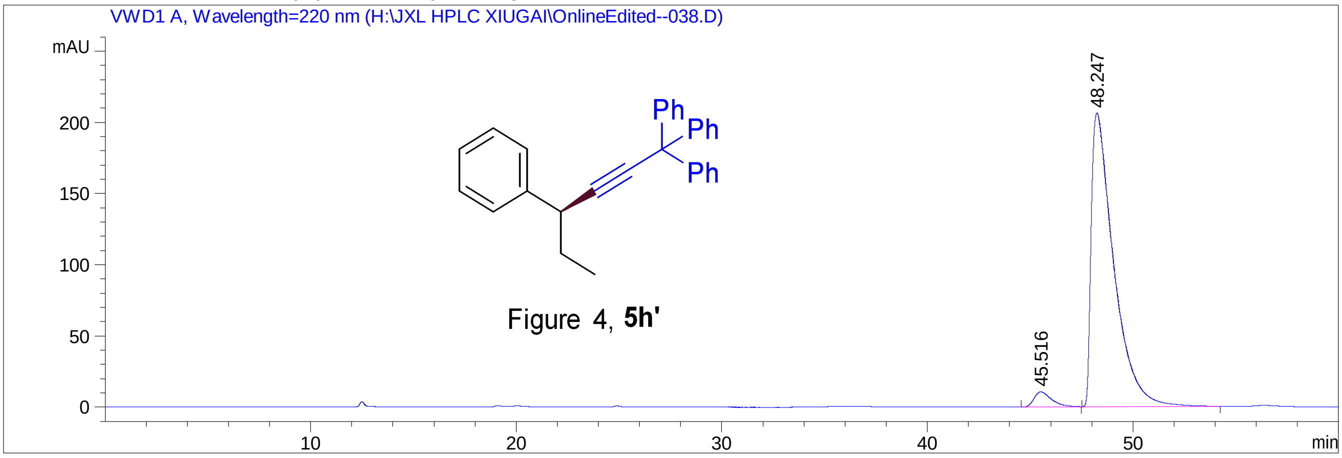

===

Area Percent Report

===

Sorted By : Signal
Multiplier : 1.0000
Dilution : 1.0000
Do not use Multiplier & Dilution Factor with ISTDs

Signal 1: VWD1 A, Wavelength=220 nm

Peak #	RetTime [min]	Type	Width [min]	Area [mAU*s]	Height [mAU]	Area %
1	45.516	BB	0.6972	631.42139	10.64241	3.8816
2	48.247	BB	1.0764	1.56356e4	206.36812	96.1184

Totals : 1.62670e4 217.01053

===

*** End of Report ***

Supplementary Fig. 282. HPLC spectra for compound **5h'**

Data File H:\JXL HPLC\JXL-03-136-2-OD(2)-RAC.D
Sample Name: JXL-03-136-2-OD(2)

===

Acq. Operator : 系统 Seq. Line : 51
Acq. Instrument : HPLC-1260 Location : 76
Injection Date : 9/4/2020 10:05:01 AM Inj : 1
Inj Volume : 3.000 µl
Different Inj Volume from Sample Entry! Actual Inj Volume : 0.600 µl
Acq. Method : D:\JXL\20200902\YH 2020-09-02 16-57-48\0IPA-50-0.5-1-6-220-JXL.M
Last changed : 9/4/2020 10:33:34 AM by 系统
(modified after loading)
Analysis Method : E:\DATA\20201027\LC 2020-12-15 14-02-17\10IPA_20_1.0_4.ZWJ.M (Sequence Method)
Last changed : 12/16/2020 10:29:15 AM by SYSTEM
(modified after loading)
Additional Info : Peak(s) manually integrated

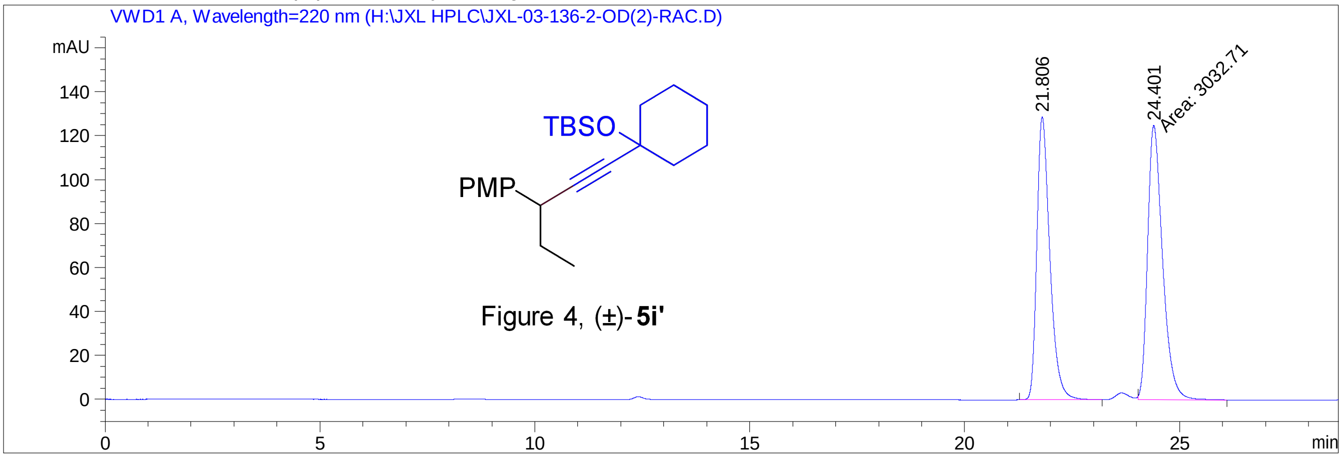

===

Area Percent Report

===

Sorted By : Signal
Multiplier : 1.0000
Dilution : 1.0000
Do not use Multiplier & Dilution Factor with ISTDs

Signal 1: VWD1 A, Wavelength=220 nm

Peak #	RetTime [min]	Type	Width [min]	Area [mAU*s]	Height [mAU]	Area %
1	21.806	BB	0.3197	2708.47876	128.85146	47.1763
2	24.401	FM	0.4045	3032.70752	124.94582	52.8237

Totals : 5741.18628 253.79727

===

*** End of Report ***

Supplementary Fig. 283. HPLC spectra for compound (±)-**5i'**

Data File H:\JXL HPLC\JXL-03-146-1-OD(2)-EE.D
Sample Name: JXL-03-146-1-OD(2)

===

Acq. Operator : 系统 Seq. Line : 90
Acq. Instrument : HPLC-1260 Location : 81
Injection Date : 9/8/2020 1:06:59 AM Inj : 1
Inj Volume : 3.000 µl
Different Inj Volume from Sample Entry! Actual Inj Volume : 0.300 µl
Acq. Method : D:\zy\20200906\YH 2020-09-06 12-21-16\0IPA-50-0.5-1-6-220-JXL.M
Last changed : 9/8/2020 12:10:11 AM by 系统
Analysis Method : E:\DATA\20201027\LC 2020-12-18 10-52-42\15IPA_30_8_4.M (Sequence Method)
Last changed : 12/18/2020 3:26:55 PM by SYSTEM
(modified after loading)
Additional Info : Peak(s) manually integrated

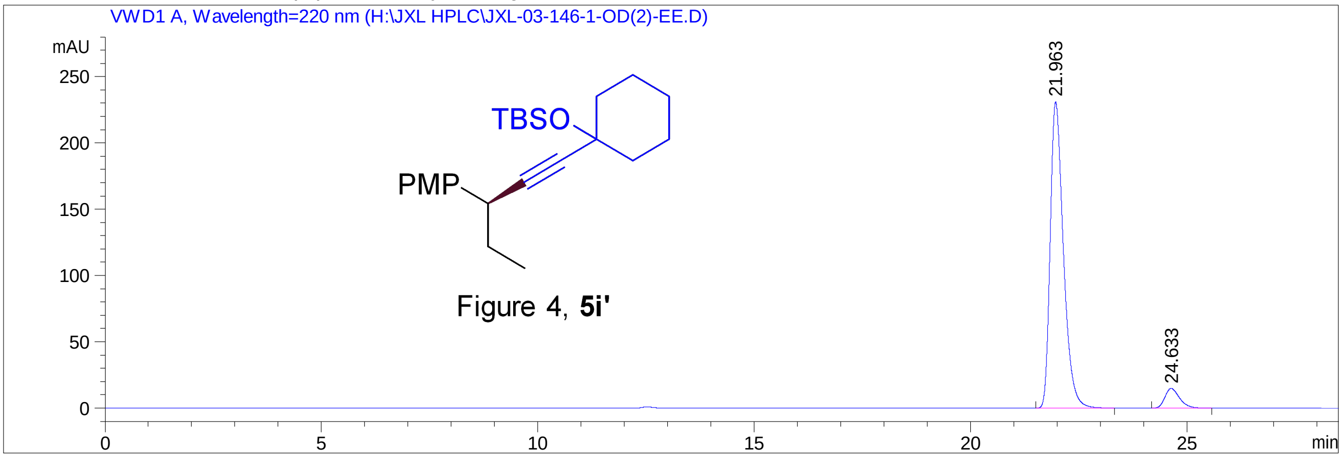

===
Area Percent Report
===

Sorted By : Signal
Multiplier : 1.0000
Dilution : 1.0000
Do not use Multiplier & Dilution Factor with ISTDs

Signal 1: VWD1 A, Wavelength=220 nm

Peak #	RetTime [min]	Type	Width [min]	Area [mAU*s]	Height [mAU]	Area %
1	21.963	BB	0.3228	4895.04541	230.91272	93.3120
2	24.633	BB	0.3485	350.84473	14.96423	6.6880

Totals : 5245.89014 245.87695

===
*** End of Report ***

Supplementary Fig. 284. HPLC spectra for compound **5i'**

Data File H:\JXL HPLC\JXL-03-183-2-CHUNHUA-RAC-OD(2).D
Sample Name: JXL-03-183-2-CHUNHUA-OD(2)

===

Acq. Operator : 系统 Seq. Line : 69
Acq. Instrument : HPLC-1260 Location : 73
Injection Date : 10/31/2020 4:30:26 AM Inj : 1
Inj Volume : 3.000 µl
Different Inj Volume from Sample Entry! Actual Inj Volume : 0.800 µl
Acq. Method : D:\G527\FZ\20201026\YH 2020-10-29 08-35-49\0.5IPA-60-0.5-1-6-220-JXL.M
Last changed : 10/30/2020 11:25:02 PM by 系统
Analysis Method : E:\DATA\20201027\LC 2020-12-18 10-52-42\15IPA_30_8_4.M (Sequence Method)
Last changed : 12/18/2020 3:29:11 PM by SYSTEM
(modified after loading)

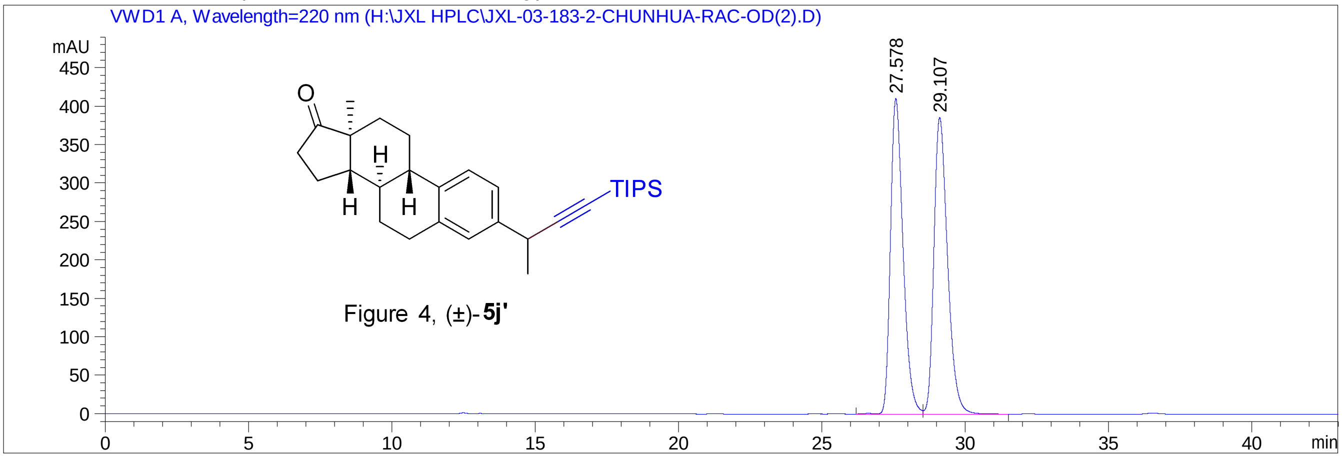

===

Area Percent Report

===

Sorted By : Signal
Multiplier : 1.0000
Dilution : 1.0000
Do not use Multiplier & Dilution Factor with ISTDs

Signal 1: VWD1 A, Wavelength=220 nm

Peak #	RetTime [min]	Type	Width [min]	Area [mAU*s]	Height [mAU]	Area %
1	27.578	VV R	0.4689	1.25377e4	410.39276	49.5354
2	29.107	VB	0.5077	1.27729e4	385.89130	50.4646

Totals : 2.53106e4 796.28406

===

*** End of Report ***

Supplementary Fig. 285. HPLC spectra for compound (±)-**5j'**

Data File H:\JXL HPLC\JXL-03-186-1-OD(2)-dr.D
Sample Name: JXL-03-186-1-OD(2)

===
Acq. Operator : 系统 Seq. Line : 70
Acq. Instrument : HPLC-1260 Location : 71
Injection Date : 10/31/2020 5:31:56 AM Inj : 1
Inj Volume : 3.000 µl
Different Inj Volume from Sample Entry! Actual Inj Volume : 0.500 µl
Acq. Method : D:\G527\FZ\20201026\YH 2020-10-29 08-35-49\0.5IPA-60-0.5-1-6-220-JXL.M
Last changed : 10/30/2020 11:25:02 PM by 系统
Analysis Method : E:\DATA\20201027\LC 2020-12-15 14-02-17\10IPA_20_1.0_4.ZWJ.M (Sequence Method)
Last changed : 12/16/2020 10:35:31 AM by SYSTEM
(modified after loading)
Additional Info : Peak(s) manually integrated

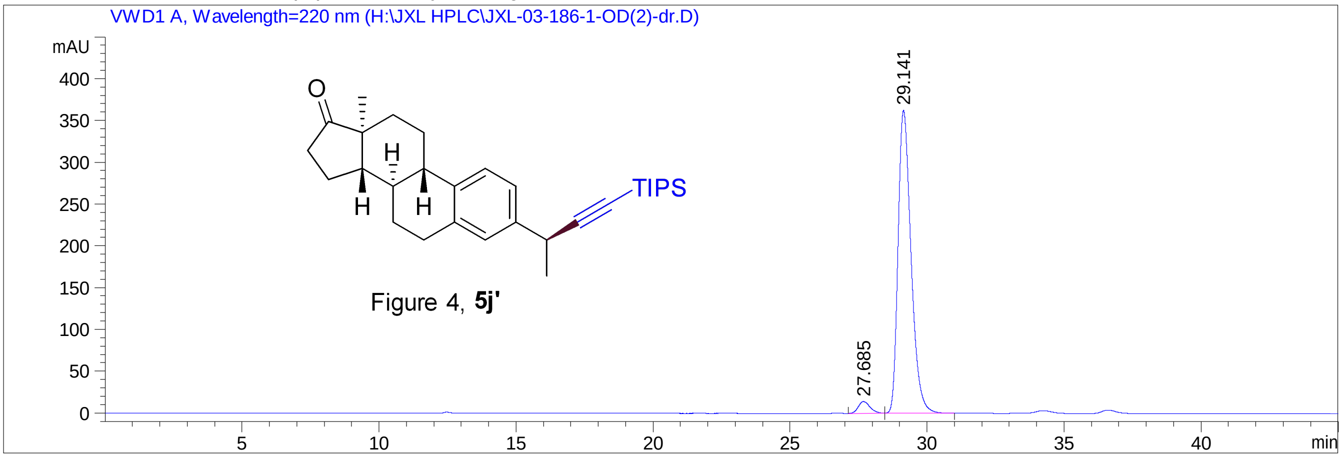

===
Area Percent Report
===

Sorted By : Signal
Multiplier : 1.0000
Dilution : 1.0000
Do not use Multiplier & Dilution Factor with ISTDs

Signal 1: VWD1 A, Wavelength=220 nm

Peak #	RetTime [min]	Type	Width [min]	Area [mAU*s]	Height [mAU]	Area %
1	27.685	BB	0.4149	415.06985	14.10046	3.3649
2	29.141	BB	0.5068	1.19203e4	362.34137	96.6351

Totals : 1.23354e4 376.44183

===
*** End of Report ***

Supplementary Fig. 286. HPLC spectra for compound **5j'**

Data File H:\JXL HPLC\JXL-03-189-1-OD(2)-dr.D
Sample Name: JXL-03-189-1-OD(2)

===

Acq. Operator : 系统 Seq. Line : 71
Acq. Instrument : HPLC-1260 Location : 72
Injection Date : 10/31/2020 6:33:24 AM Inj : 1
Inj Volume : 3.000 µl
Different Inj Volume from Sample Entry! Actual Inj Volume : 0.500 µl
Acq. Method : D:\G527\FZ\20201026\YH 2020-10-29 08-35-49\0.5IPA-60-0.5-1-6-220-JXL.M
Last changed : 10/30/2020 11:25:02 PM by 系统
Analysis Method : E:\DATA\20201027\LC 2020-12-15 14-02-17\10IPA_20_1.0_4.ZWJ.M (Sequence Method)
Last changed : 12/16/2020 10:38:02 AM by SYSTEM (modified after loading)
Additional Info : Peak(s) manually integrated

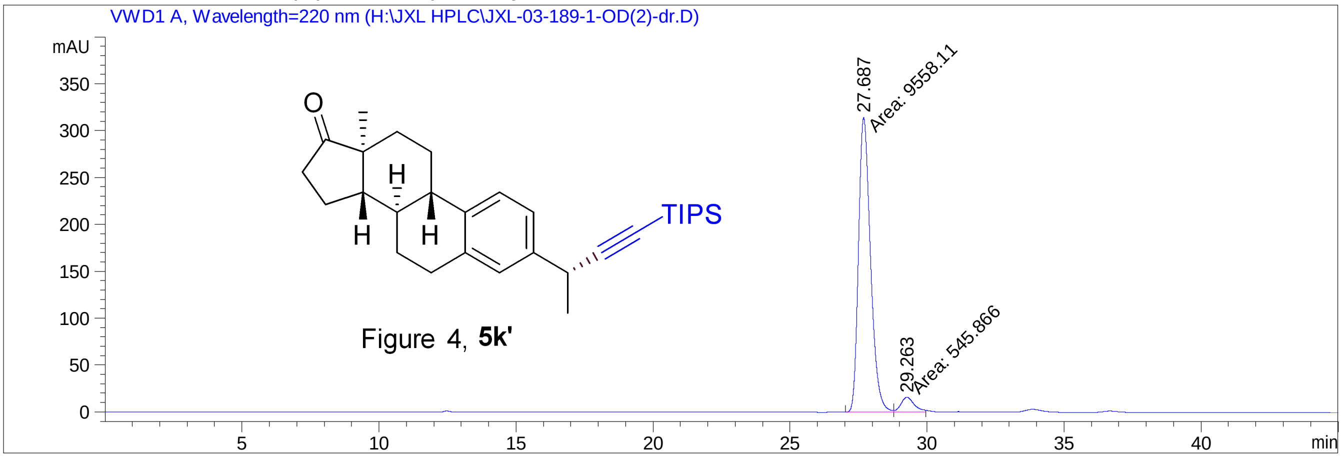

===

Area Percent Report

===

Sorted By : Signal
Multiplier : 1.0000
Dilution : 1.0000
Do not use Multiplier & Dilution Factor with ISTDs

Signal 1: VWD1 A, Wavelength=220 nm

Peak #	RetTime [min]	Type	Width [min]	Area [mAU*s]	Height [mAU]	Area %
1	27.687	MF	0.5074	9558.11328	313.93344	94.5975
2	29.263	MF	0.5734	545.86591	15.86660	5.4025
Totals :				1.01040e4	329.80004	

===

*** End of Report ***

Supplementary Fig. 287. HPLC spectra for compound (±)-**5k'**

```
Data File H:\HPLC20210310\OnlineEdited--020.D
Sample Name: HB-04-48-OD(1)

     =====================================================================
     Acq. Operator   : 系统                            Seq. Line :  20
     Acq. Instrument : HPLC-1260                        Location :   91
     Injection Date  : 3/9/2021 4:31:46 PM                   Inj :   1
                                                      Inj Volume : 3.000 µl
     Different Inj Volume from Sample Entry!  Actual Inj Volume : 0.300 µl
     Acq. Method     : D:\ZHH\20210309\YH 2021-03-09 10-26-38\1IPA-30-0.5-1-2-220-JXL .M
     Last changed    : 3/9/2021 4:13:51 PM by 系统
     Analysis Method : E:\DATA\20210310\LC 2021-03-10 20-00-42\0IPA_30_0.5_2 H.M (Sequence Method)
     Last changed    : 3/10/2021 10:29:41 PM by SYSTEM
                       (modified after loading)
     Additional Info : Peak(s) manually integrated
```

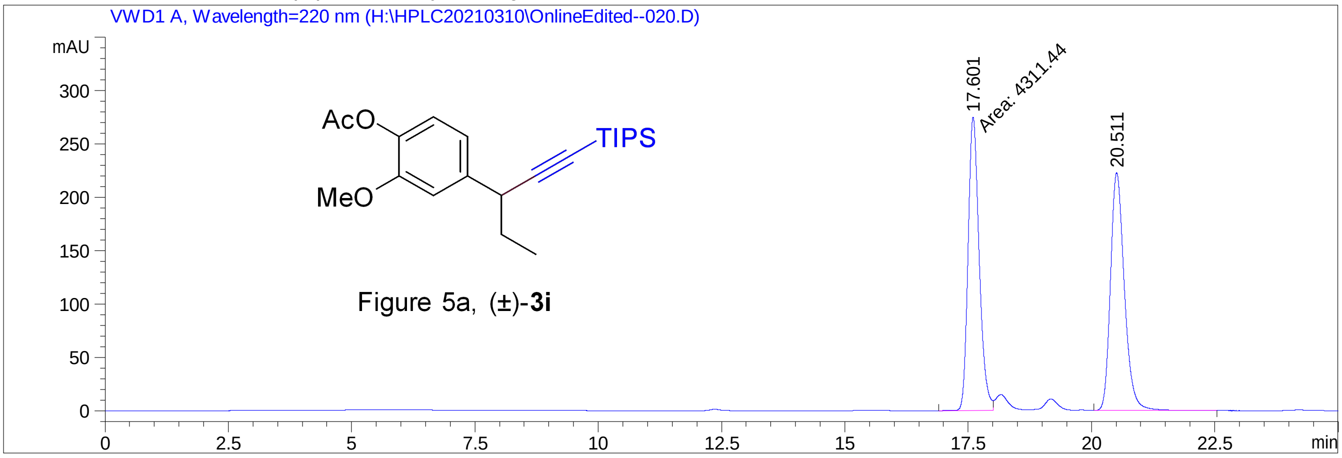


```
     =====================================================================
                              Area Percent Report
     =====================================================================

     Sorted By             :      Signal
     Multiplier            :      1.0000
     Dilution              :      1.0000
     Do not use Multiplier & Dilution Factor with ISTDs


     Signal 1: VWD1 A, Wavelength=220 nm

     Peak RetTime Type  Width     Area      Height     Area
       #   [min]        [min]   [mAU*s]     [mAU]       %
     ----|-------|----|-------|----------|----------|--------|
        1  17.601 MF    0.2615 4311.43701  274.81775  49.6585
        2  20.511 BB    0.3011 4370.74121  222.61029  50.3415

     Totals :                  8682.17822  497.42804


     =====================================================================
                              *** End of Report ***
```


Supplementary Fig. 288. HPLC spectra for compound (±)-**3i**

Data File H:\HPLC20210310\OnlineEdited--022.D
Sample Name: JXL-04-128-5-OD(2)

===

Acq. Operator : 系统 Seq. Line : 22
Acq. Instrument : HPLC-1260 Location : 92
Injection Date : 3/9/2021 5:34:34 PM Inj : 1
Inj Volume : 3.000 µl
Different Inj Volume from Sample Entry! Actual Inj Volume : 0.800 µl
Acq. Method : D:\ZHH\20210309\YH 2021-03-09 10-26-38\1IPA-30-0.5-1-2-220-JXL .M
Last changed : 3/9/2021 4:13:51 PM by 系统
Analysis Method : E:\DATA\20210310\LC 2021-03-10 20-00-42\0IPA_30_0.5_2 H.M (Sequence Method)
Last changed : 3/10/2021 10:31:15 PM by SYSTEM
(modified after loading)
Additional Info : Peak(s) manually integrated

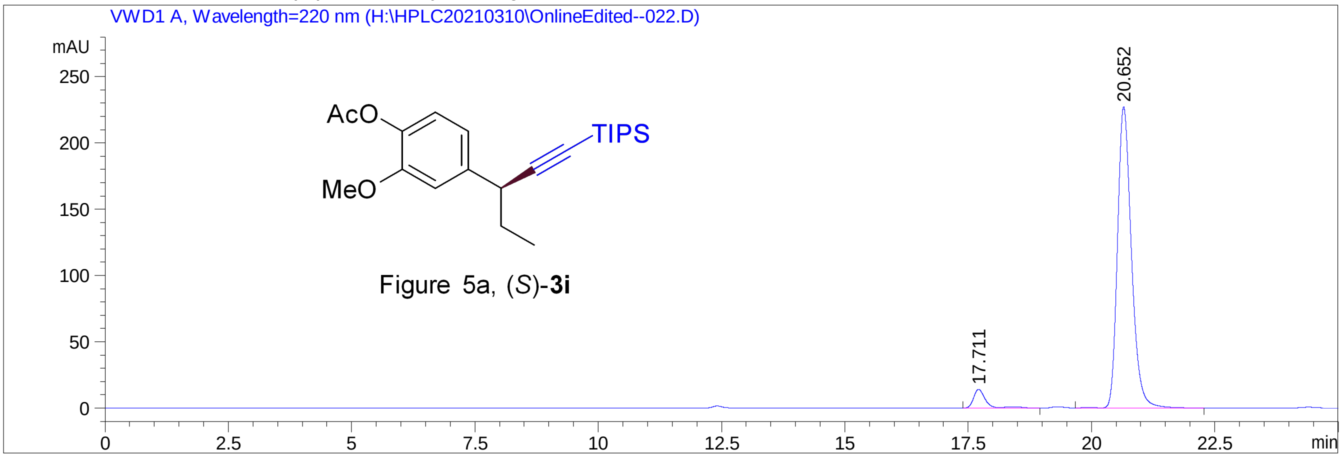

===

Area Percent Report

===

Sorted By : Signal
Multiplier : 1.0000
Dilution : 1.0000
Do not use Multiplier & Dilution Factor with ISTDs

Signal 1: VWD1 A, Wavelength=220 nm

Peak #	RetTime [min]	Type	Width [min]	Area [mAU*s]	Height [mAU]	Area %
1	17.711	BV R	0.2603	247.38464	14.19770	5.2441
2	20.652	VB R	0.3020	4470.03857	227.23535	94.7559

Totals : 4717.42322 241.43305

===

*** End of Report ***

Supplementary Fig. 289. HPLC spectra for compound (*S*)-**3i**

Data File H:\JXL HPLC\JXL-03-180-1-OD(1)-RAC.D
Sample Name: JXL-03-180-1-OD(1)

===
Acq. Operator : 系统 Seq. Line : 13
Acq. Instrument : HPLC-1260 Location : 82
Injection Date : 10/1/2020 7:46:18 PM Inj : 1
Inj Volume : 3.000 µl
Different Inj Volume from Sample Entry! Actual Inj Volume : 2.000 µl
Acq. Method : D:\zy\20200930\YH 2020-10-01 14-39-42\0IPA-80-0.5-1-6-220-JXL.M
Last changed : 10/1/2020 8:42:50 PM by 系统
(modified after loading)
Analysis Method : E:\DATA\20201027\LC 2020-11-25 23-35-05\10IPA-60-0.5-220-4-JXL TFA.M (
Sequence Method)
Last changed : 12/16/2020 10:51:17 AM by SYSTEM
(modified after loading)
Additional Info : Peak(s) manually integrated

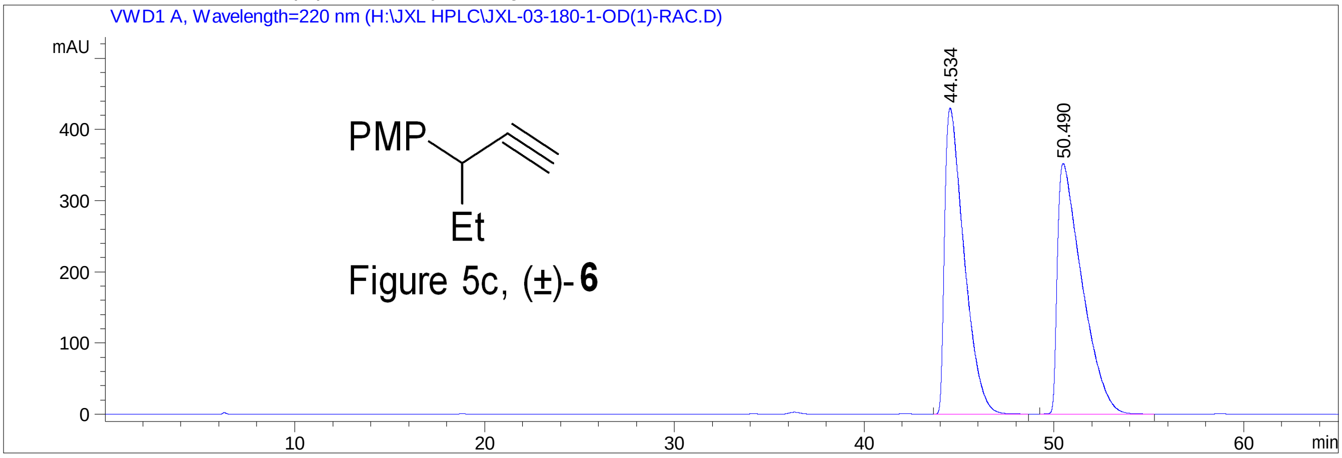

===
Area Percent Report
===

Sorted By : Signal
Multiplier : 1.0000
Dilution : 1.0000
Do not use Multiplier & Dilution Factor with ISTDs

Signal 1: VWD1 A, Wavelength=220 nm

Peak #	RetTime [min]	Type	Width [min]	Area [mAU*s]	Height [mAU]	Area %
1	44.534	BB	1.0287	3.06876e4	430.47202	49.1524
2	50.490	BB	1.2161	3.17460e4	352.04309	50.8476

Totals : 6.24336e4 782.51511

===
*** End of Report ***

Supplementary Fig. 290. HPLC spectra for compound (±)-**6**

Data File H:\JXL HPLC\JXL-03-181-1-OD(1)-EE.D
Sample Name: JXL-03-181-1-OD(1)

===

Acq. Operator : 系统 Seq. Line : 14
Acq. Instrument : HPLC-1260 Location : 83
Injection Date : 10/1/2020 8:52:42 PM Inj : 1
Inj Volume : 3.000 µl
Different Inj Volume from Sample Entry! Actual Inj Volume : 1.500 µl
Acq. Method : D:\zy\20200930\YH 2020-10-01 14-39-42\0IPA-80-0.5-1-6-220-JXL.M
Last changed : 10/1/2020 8:42:50 PM by 系统
Analysis Method : E:\DATA\20201027\LC 2020-11-25 23-35-05\10IPA-60-0.5-220-4-JXL TFA.M (Sequence Method)
Last changed : 12/16/2020 10:54:09 AM by SYSTEM
(modified after loading)
Additional Info : Peak(s) manually integrated

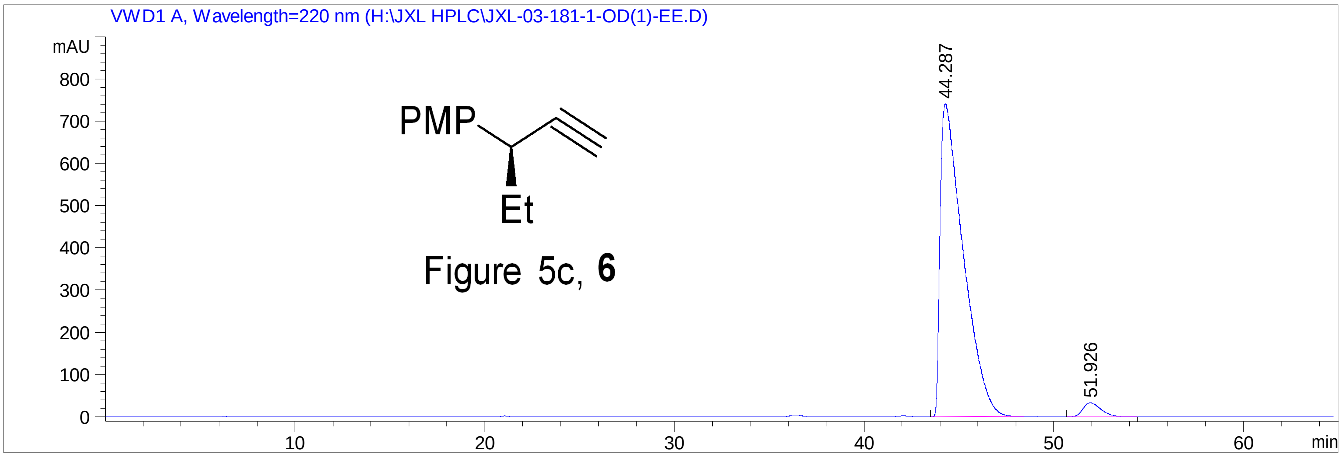

===

Area Percent Report

===

Sorted By : Signal
Multiplier : 1.0000
Dilution : 1.0000
Do not use Multiplier & Dilution Factor with ISTDs

Signal 1: VWD1 A, Wavelength=220 nm

Peak #	RetTime [min]	Type	Width [min]	Area [mAU*s]	Height [mAU]	Area %
1	44.287	BB	1.1729	6.26115e4	741.29761	96.5824
2	51.926	BB	0.7805	2215.55591	33.26823	3.4176

Totals : 6.48271e4 774.56584

===

*** End of Report ***

Supplementary Fig. 291. HPLC spectra for compound **6**

Data File H:\JXL HPLC\JXL-04-20-1-Click-RAC-IC(1).D
Sample Name: JXL-04-20-1-Click-RAC-IC(1)

===

Acq. Operator : 系统 Seq. Line : 157
Acq. Instrument : HPLC-1260 Location : 93
Injection Date : 11/1/2020 10:51:34 PM Inj : 1
Inj Volume : 3.000 µl
Different Inj Volume from Sample Entry! Actual Inj Volume : 8.000 µl
Acq. Method : D:\G527\FZ\20201026\YH 2020-10-29 08-35-49\20IPA-60-0.8-1-2-254-JXL.M
Last changed : 10/30/2020 11:10:37 PM by 系统
Analysis Method : E:\DATA\20201027\LC 2020-11-25 23-35-05\10IPA-60-0.5-220-4-JXL TFA.M (Sequence Method)
Last changed : 12/16/2020 10:55:36 AM by SYSTEM
(modified after loading)
Additional Info : Peak(s) manually integrated

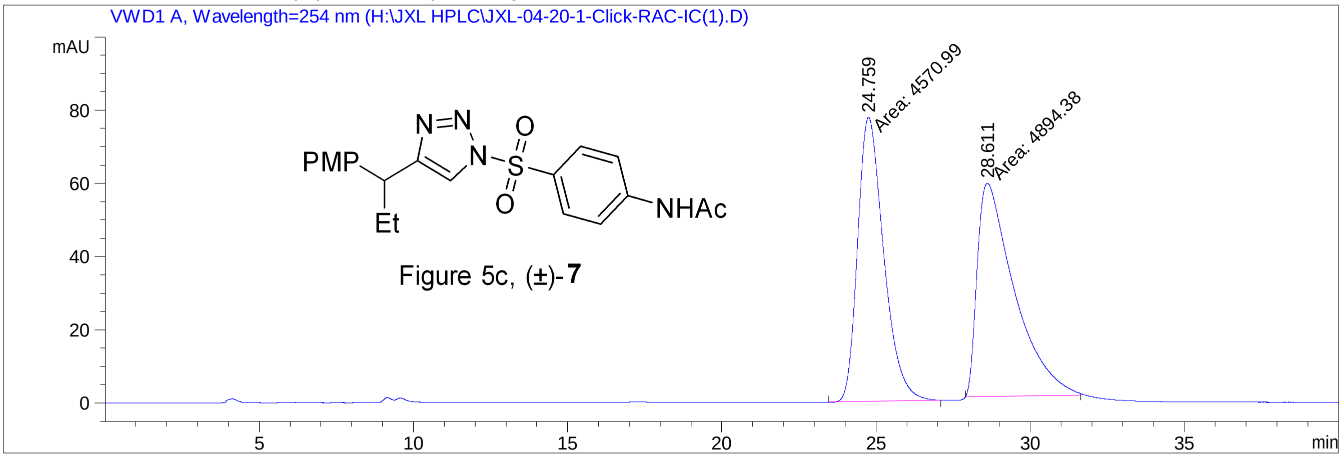

===
Area Percent Report
===

Sorted By : Signal
Multiplier : 1.0000
Dilution : 1.0000
Do not use Multiplier & Dilution Factor with ISTDs

Signal 1: VWD1 A, Wavelength=254 nm

Peak #	RetTime [min]	Type	Width [min]	Area [mAU*s]	Height [mAU]	Area %
1	24.759	MM	0.9815	4570.99170	77.61681	48.2917
2	28.611	MM	1.3984	4894.38379	58.33504	51.7083

Totals : 9465.37549 135.95186

===
*** End of Report ***

Supplementary Fig. 292. HPLC spectra for compound (±)-**7**

Data File H:\JXL HPLC\JXL-04-20-1-Click-EE-IC(1).D
Sample Name: JXL-04-20-1-Click-EE-IC(1)

===

Acq. Operator : 系统 Seq. Line : 158
Acq. Instrument : HPLC-1260 Location : 94
Injection Date : 11/1/2020 11:33:06 PM Inj : 1
Inj Volume : 3.000 µl
Different Inj Volume from Sample Entry! Actual Inj Volume : 8.000 µl
Acq. Method : D:\G527\FZ\20201026\YH 2020-10-29 08-35-49\20IPA-60-0.8-1-2-254-JXL.M
Last changed : 10/30/2020 11:10:37 PM by 系统
Analysis Method : E:\DATA\20201027\LC 2020-12-29 20-00-48\15IPA_20_8_4.M (Sequence Method)
Last changed : 12/31/2020 11:53:19 PM by SYSTEM
(modified after loading)
Additional Info : Peak(s) manually integrated

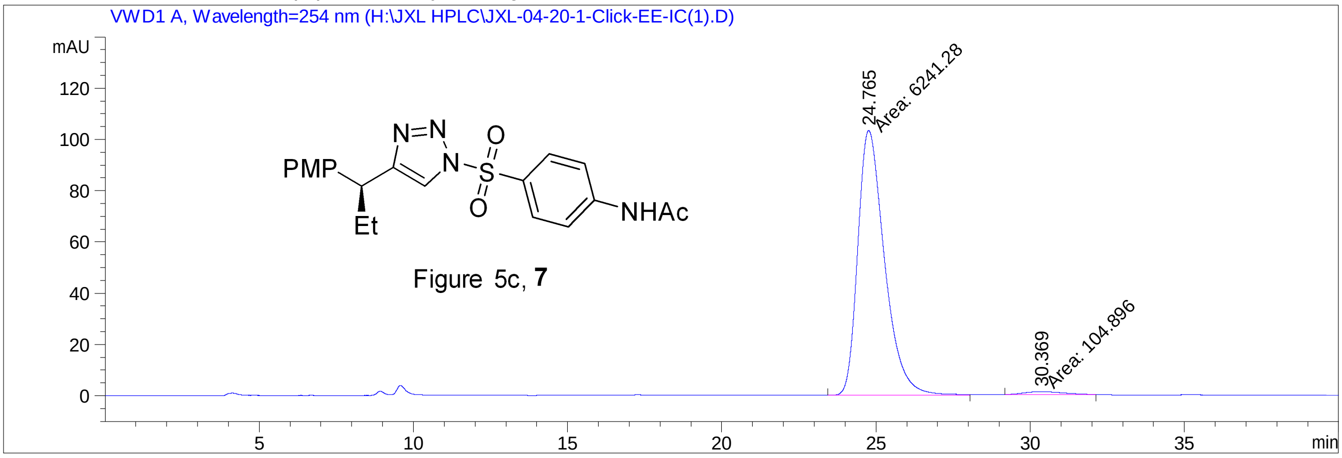

===

Area Percent Report

===

Sorted By : Signal
Multiplier : 1.0000
Dilution : 1.0000
Do not use Multiplier & Dilution Factor with ISTDs

Signal 1: VWD1 A, Wavelength=254 nm

Peak #	RetTime [min]	Type	Width [min]	Area [mAU*s]	Height [mAU]	Area %
1	24.765	MF	1.0067	6241.28369	103.32697	98.3471
2	30.369	MM	1.4662	104.89571	1.19238	1.6529

Totals : 6346.17941 104.51934

===

*** End of Report ***

Supplementary Fig. 293. HPLC spectra for compound **7**

Data File E:\DATA\20201027\LC 2020-11-24 17-29-49\OnlineEdited--059.D
Sample Name: JXL-04-47-1-IC(1)-RAC

===

Acq. Operator : SYSTEM Seq. Line : 59
Acq. Instrument : HPLC1260 Location : P2-B1
Injection Date : 11/25/2020 4:28:55 PM Inj : 1
Inj Volume : 3.000 µl
Different Inj Volume from Sample Entry! Actual Inj Volume : 2.000 µl
Acq. Method : E:\DATA\20201027\LC 2020-11-24 17-29-49\1IPA-40-0.5-220-3-JXL.M
Last changed : 11/25/2020 5:10:38 PM by SYSTEM
(modified after loading)
Analysis Method : E:\DATA\20201027\LC 2020-11-24 17-29-49\1IPA-40-0.5-220-3-JXL.M (Sequence Method)
Last changed : 12/16/2020 11:01:36 AM by SYSTEM
(modified after loading)
Additional Info : Peak(s) manually integrated

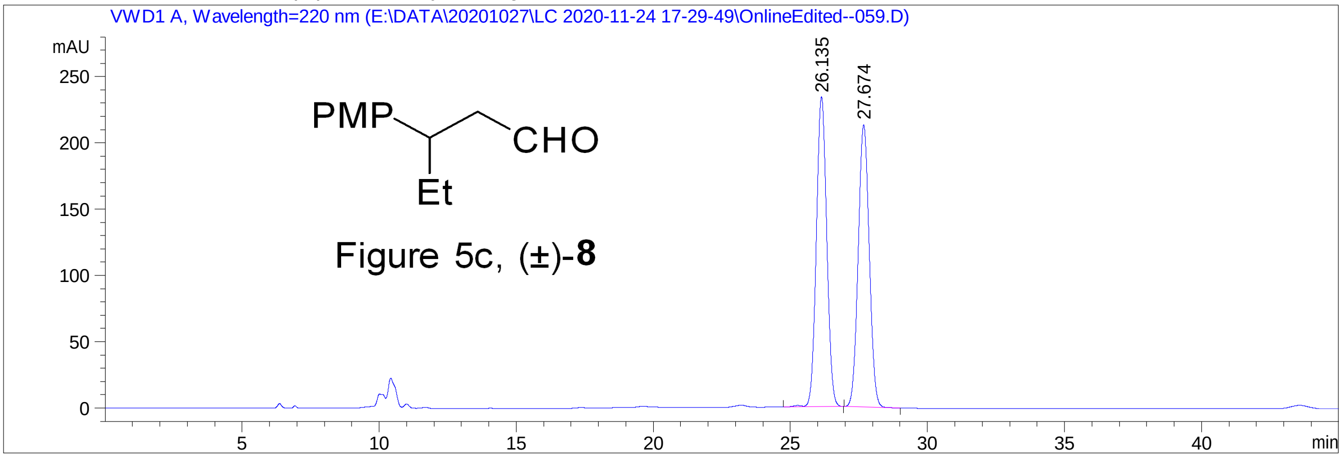

===

Area Percent Report

===

Sorted By : Signal
Multiplier : 1.0000
Dilution : 1.0000
Do not use Multiplier & Dilution Factor with ISTDs

Signal 1: VWD1 A, Wavelength=220 nm

Peak #	RetTime [min]	Type	Width [min]	Area [mAU*s]	Height [mAU]	Area %
1	26.135	VB R	0.4109	6170.07764	233.78003	50.8392
2	27.674	BB	0.4379	5966.36768	212.94882	49.1608

Totals : 1.21364e4 446.72885

===

*** End of Report ***

Supplementary Fig. 294. HPLC spectra for compound (±)-**8**

Data File E:\DATA\20201027\LC 2020-11-24 17-29-49\OnlineEdited--060.D
Sample Name: JXL-04-47-2-IC(1)-EE

===

Acq. Operator   : SYSTEM   Seq. Line : 60
Acq. Instrument : HPLC1260   Location :   P2-B2
Injection Date : 11/25/2020 5:14:38 PM   Inj :   1
                                                Inj Volume : 3.000 µl
Different Inj Volume from Sample Entry! Actual Inj Volume : 0.300 µl
Acq. Method   : E:\DATA\20201027\LC 2020-11-24 17-29-49\1IPA-40-0.5-220-3-JXL.M
Last changed   : 11/25/2020 5:10:38 PM by SYSTEM
Analysis Method : E:\DATA\20201027\LC 2020-11-24 17-29-49\1IPA-40-0.5-220-3-JXL.M (Sequence Method)
Last changed   : 12/16/2020 11:01:36 AM by SYSTEM
(modified after loading)
Additional Info : Peak(s) manually integrated

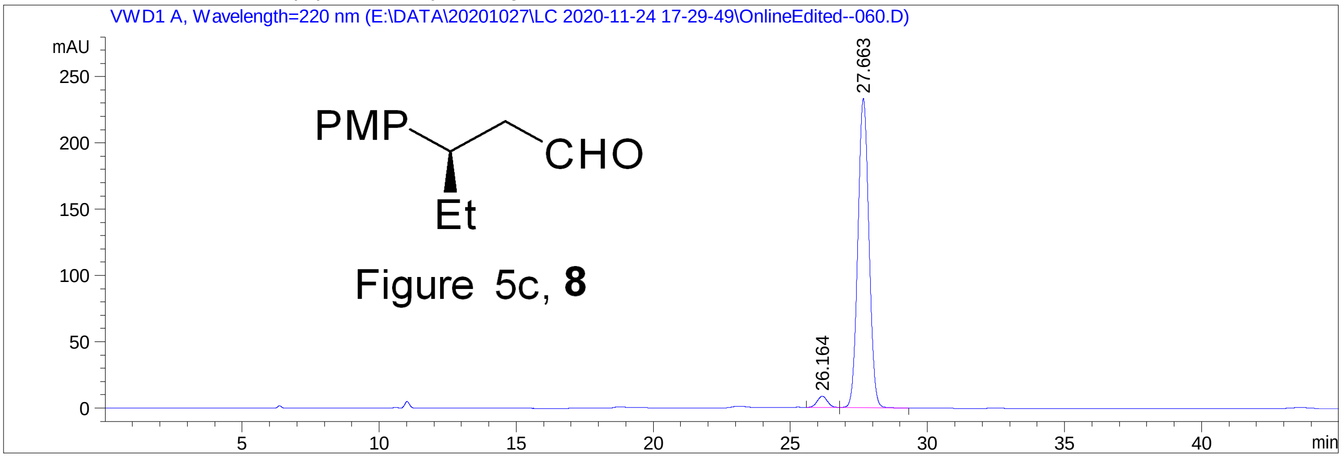

===

Area Percent Report

===

Sorted By : Signal
Multiplier : 1.0000
Dilution : 1.0000
Do not use Multiplier & Dilution Factor with ISTDs

Signal 1: VWD1 A, Wavelength=220 nm

Peak #	RetTime [min]	Type	Width [min]	Area [mAU*s]	Height [mAU]	Area %
1	26.164	BB	0.4043	217.16803	8.40917	3.2146
2	27.663	BB	0.4369	6538.50342	233.36398	96.7854

Totals : 6755.67145 241.77315

===

*** End of Report ***

Supplementary Fig. 295. HPLC spectra for compound **8**

Data File H:\JXL HPLC\JXL-DIBAL-OD(2)-RAC.D
Sample Name: JXL-DIBAL-OD(2)

===

Acq. Operator : 系统 Seq. Line : 111
Acq. Instrument : HPLC-1260 Location : 91
Injection Date : 10/16/2020 2:02:41 AM Inj : 1
Inj Volume : 3.000 µl
Different Inj Volume from Sample Entry! Actual Inj Volume : 0.200 µl
Acq. Method : D:\G527\FZ\20201013\YH 2020-10-13 08-36-19\0IPA-40-0.5-1-2-220-JXL.M
Last changed : 10/15/2020 11:27:15 PM by 系统
Analysis Method : E:\DATA\20201027\LC 2020-12-15 14-02-17\10IPA_20_1.0_4.ZWJ.M (Sequence Method)
Last changed : 12/16/2020 10:39:43 AM by SYSTEM
(modified after loading)
Additional Info : Peak(s) manually integrated

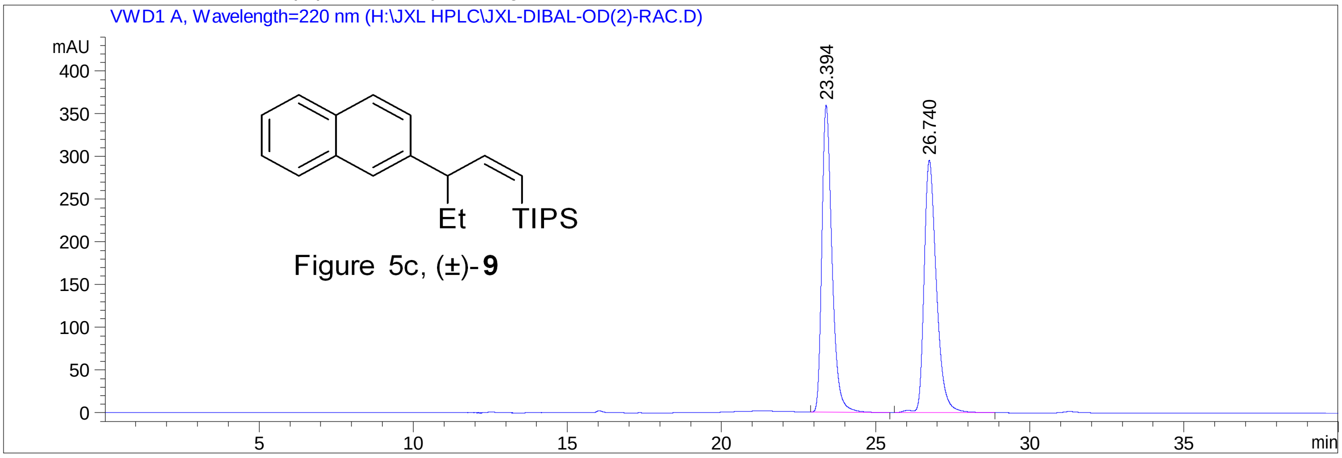

===

Area Percent Report

===

Sorted By : Signal
Multiplier : 1.0000
Dilution : 1.0000
Do not use Multiplier & Dilution Factor with ISTDs

Signal 1: VWD1 A, Wavelength=220 nm

Peak #	RetTime [min]	Type	Width [min]	Area [mAU*s]	Height [mAU]	Area %
1	23.394	BB	0.3493	8235.24219	359.38779	50.0443
2	26.740	VB R	0.4200	8220.66504	295.71948	49.9557

Totals : 1.64559e4 655.10727

===

*** End of Report ***

Supplementary Fig. 296. HPLC spectra for compound (±)-**9**

Data File H:\JXL HPLC\DIBAL-H(XIN)-OD(2).D
Sample Name: DIBAL-H(XIN)-OD(2)

===

Acq. Operator : 系统 Seq. Line : 26
Acq. Instrument : HPLC-1260 Location : 93
Injection Date : 11/6/2020 10:32:36 PM Inj : 1
Inj Volume : 3.000 µl
Different Inj Volume from Sample Entry! Actual Inj Volume : 0.200 µl
Acq. Method : D:\G527\FZ\20201106\YH 2020-11-06 09-03-38\0IPA-40-0.5-1-6-220-JXL.M
Last changed : 11/6/2020 11:03:43 PM by 系统
(modified after loading)
Analysis Method : E:\DATA\20201027\LC 2020-12-15 14-02-17\10IPA_20_1.0_4.ZWJ.M (Sequence Method)
Last changed : 12/16/2020 10:41:24 AM by SYSTEM
(modified after loading)
Additional Info : Peak(s) manually integrated

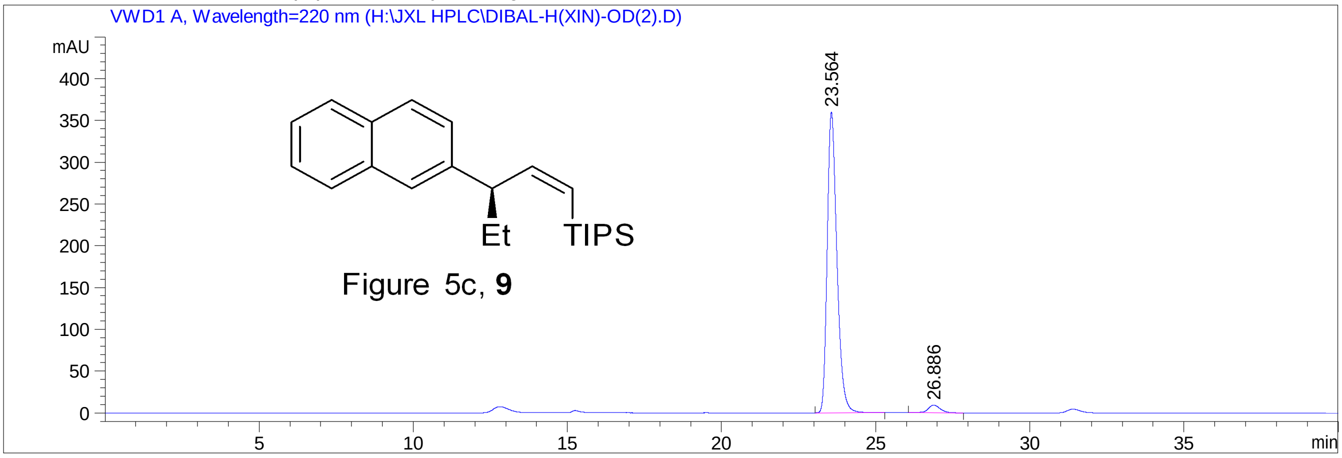

===

Area Percent Report

===

Sorted By : Signal
Multiplier : 1.0000
Dilution : 1.0000
Do not use Multiplier & Dilution Factor with ISTDs

Signal 1: VWD1 A, Wavelength=220 nm

Peak #	RetTime [min]	Type	Width [min]	Area [mAU*s]	Height [mAU]	Area %
1	23.564	BB	0.3336	7883.77832	359.83118	96.8967
2	26.886	BB	0.3775	252.49660	9.36688	3.1033

Totals : 8136.27492 369.19806

===

*** End of Report ***

Supplementary Fig. 297. HPLC spectra for compound **9**

Data File E:\DATA\20201027\LC 2020-11-25 23-35-05\OnlineEdited--009.D
Sample Name: JXL-XYH-02-24-1-AD(1)-RAC

===

Acq. Operator : SYSTEM Seq. Line : 9
Acq. Instrument : HPLC1260 Location : P2-B5
Injection Date : 11/26/2020 4:53:04 AM Inj : 1
Inj Volume : 3.000 µl
Different Inj Volume from Sample Entry! Actual Inj Volume : 6.000 µl
Acq. Method : E:\DATA\20201027\LC 2020-11-25 23-35-05\10IPA-60-0.5-220-4-JXL TFA.M
Last changed : 11/26/2020 12:35:05 AM by SYSTEM
Analysis Method : E:\DATA\20201027\LC 2020-11-25 23-35-05\10IPA-60-0.5-220-4-JXL TFA.M (Sequence Method)
Last changed : 12/18/2020 3:32:57 PM by SYSTEM
(modified after loading)
Additional Info : Peak(s) manually integrated

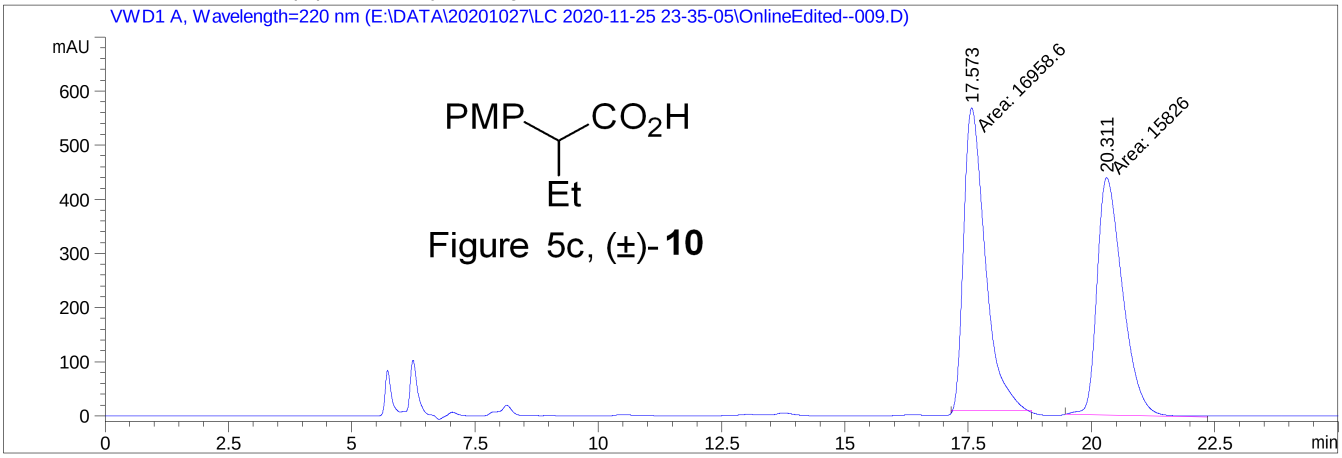

===

Area Percent Report

===

Sorted By : Signal
Multiplier : 1.0000
Dilution : 1.0000
Do not use Multiplier & Dilution Factor with ISTDs

Signal 1: VWD1 A, Wavelength=220 nm

Peak #	RetTime [min]	Type	Width [min]	Area [mAU*s]	Height [mAU]	Area %
1	17.573	MM	0.5064	1.69586e4	558.18024	51.7273
2	20.311	PM	0.6016	1.58260e4	438.45648	48.2727

Totals : 3.27845e4 996.63672

===

*** End of Report ***

Supplementary Fig. 298. HPLC spectra for compound (±)-**10**

Data File E:\DATA\20201027\LC 2020-11-25 23-35-05\OnlineEdited--003.D
Sample Name: JXL-XYH-02-27-AD(1)-EE

===

Acq. Operator : SYSTEM Seq. Line : 3
Acq. Instrument : HPLC1260 Location : P2-B1
Injection Date : 11/26/2020 12:48:22 AM Inj : 1
Inj Volume : 3.000 µl
Different Inj Volume from Sample Entry! Actual Inj Volume : 2.000 µl
Acq. Method : E:\DATA\20201027\LC 2020-11-25 23-35-05\10IPA-60-0.5-220-4-JXL TFA.M
Last changed : 11/26/2020 12:35:05 AM by SYSTEM
Analysis Method : E:\DATA\20201027\LC 2020-11-25 23-35-05\10IPA-60-0.5-220-4-JXL TFA.M (Sequence Method)
Last changed : 12/16/2020 10:49:52 AM by SYSTEM
(modified after loading)
Additional Info : Peak(s) manually integrated

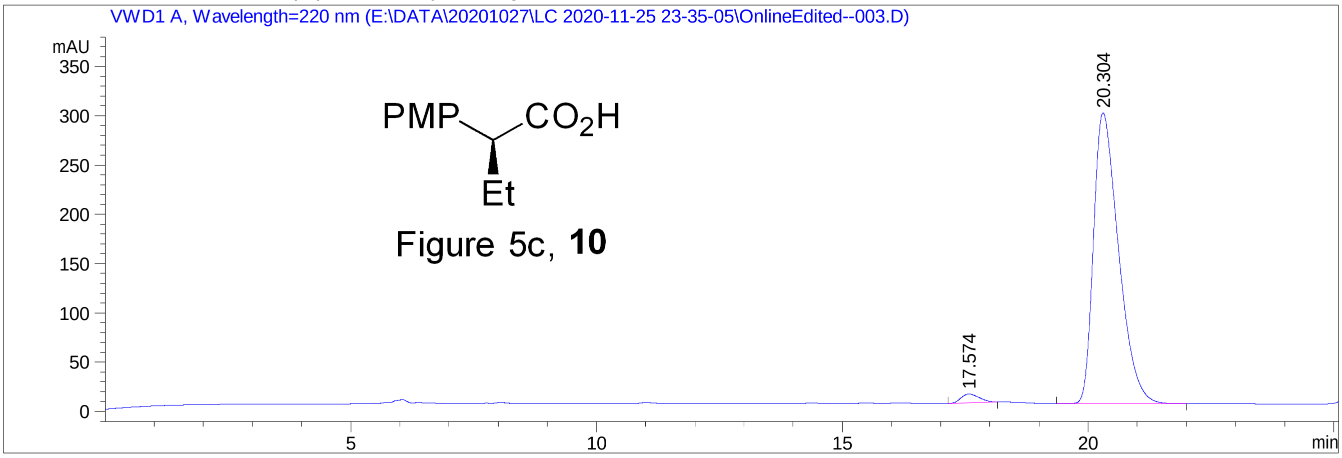

===
Area Percent Report
===

Sorted By : Signal
Multiplier : 1.0000
Dilution : 1.0000
Do not use Multiplier & Dilution Factor with ISTDs

Signal 1: VWD1 A, Wavelength=220 nm

Peak #	RetTime [min]	Type	Width [min]	Area [mAU*s]	Height [mAU]	Area %
1	17.574	BB	0.4057	230.44807	9.00215	2.1554
2	20.304	BB	0.5433	1.04610e4	295.26358	97.8446

Totals : 1.06914e4 304.26573

===
*** End of Report ***

Supplementary Fig. 299. HPLC spectra for compound **10**

Data File H:\JXL HPLC\JXL-04-55-2-OD(2)-RAC.D
Sample Name: JXL--04-55-2-OD(2)

===

Acq. Operator : 系统 Seq. Line : 62
Acq. Instrument : HPLC-1260 Location : 91
Injection Date : 12/11/2020 6:39:59 AM Inj : 1
Inj Volume : 3.000 µl
Different Inj Volume from Sample Entry! Actual Inj Volume : 0.300 µl
Acq. Method : D:\G527\FZ\20201127\YH 2020-12-10 10-05-55\0IPA-40-0.5-1-6-220-JXL.M
Last changed : 12/11/2020 12:15:19 AM by 系统
Analysis Method : E:\DATA\20201027\LC 2020-11-24 17-29-49\1IPA-40-0.5-220-3-JXL.M (Sequence Method)
Last changed : 12/16/2020 11:05:22 AM by SYSTEM
(modified after loading)

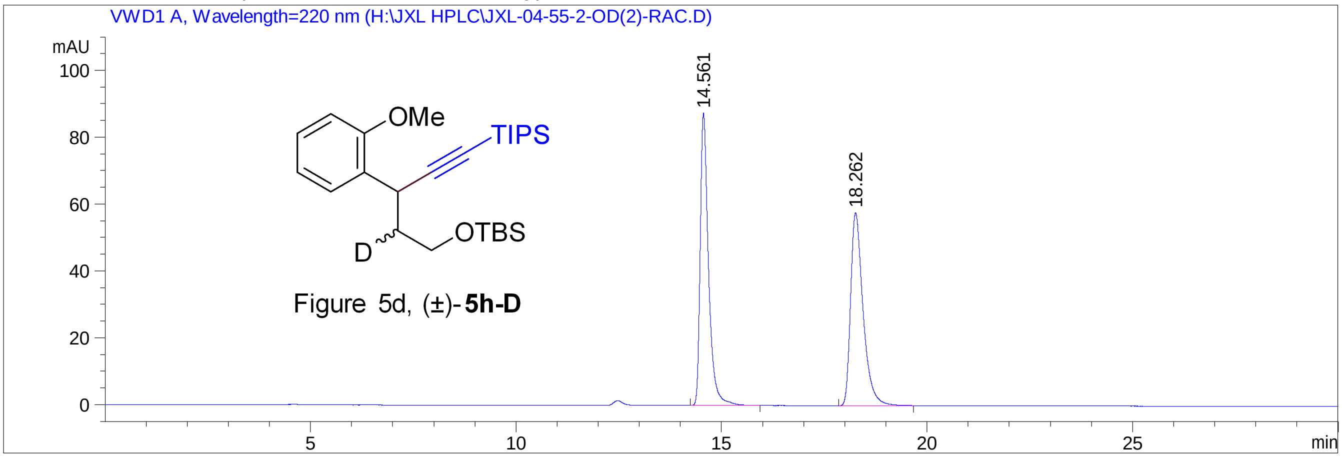

===

Area Percent Report

===

Sorted By : Signal
Multiplier : 1.0000
Dilution : 1.0000
Do not use Multiplier & Dilution Factor with ISTDs

Signal 1: VWD1 A, Wavelength=220 nm

Peak #	RetTime [min]	Type	Width [min]	Area [mAU*s]	Height [mAU]	Area %
1	14.561	BB	0.2087	1226.21692	87.40794	50.1898
2	18.262	BB	0.3189	1216.94397	57.72718	49.8102

Totals : 2443.16089 145.13512

===

*** End of Report ***

Supplementary Fig. 300. HPLC spectra for compound (±)-**5h-D**

Data File H:\JXL HPLC\JXL-04-49-1-OD(2)-EE-DAODAIDIWU.D
Sample Name: JXL-04-49-1-OD(2)

===

Acq. Operator : 系统 Seq. Line : 54
Acq. Instrument : HPLC-1260 Location : 91
Injection Date : 11/25/2020 3:53:25 AM Inj : 1
Inj Volume : 3.000 µl
Different Inj Volume from Sample Entry! Actual Inj Volume : 0.300 µl
Acq. Method : D:\G527\FZ\20201124\YH 2020-11-24 08-36-13\0IPA-40-0.5-1-6-220-JXL.M
Last changed : 11/25/2020 12:09:48 AM by 系统
Analysis Method : E:\DATA\20201027\LC 2020-11-24 17-29-49\1IPA-40-0.5-220-3-JXL.M (Sequence Method)
Last changed : 12/16/2020 11:06:48 AM by SYSTEM
(modified after loading)
Additional Info : Peak(s) manually integrated

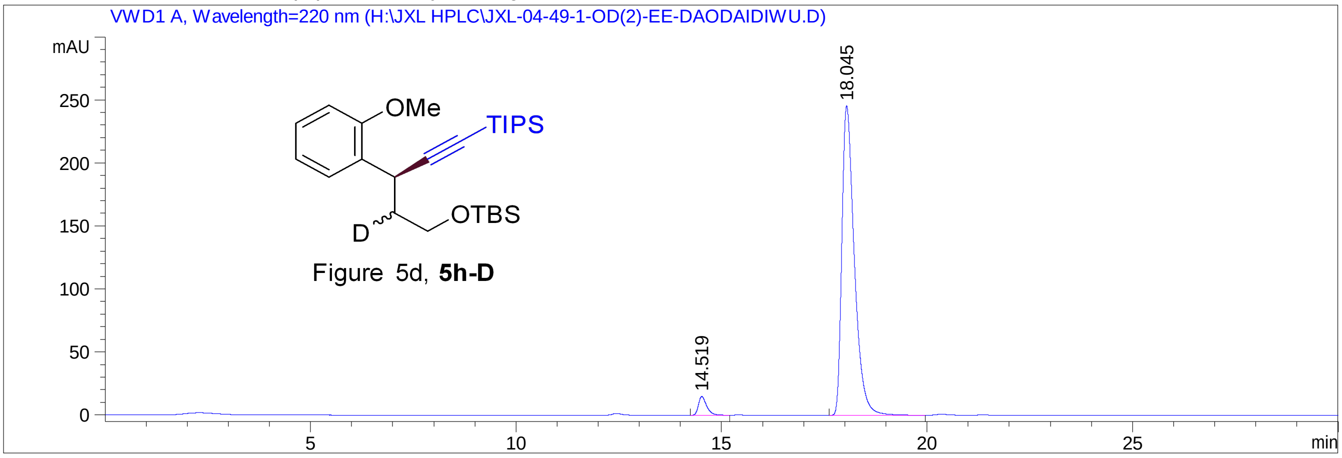

===
Area Percent Report
===

Sorted By : Signal
Multiplier : 1.0000
Dilution : 1.0000
Do not use Multiplier & Dilution Factor with ISTDs

Signal 1: VWD1 A, Wavelength=220 nm

Peak #	RetTime [min]	Type	Width [min]	Area [mAU*s]	Height [mAU]	Area %
1	14.519	BB	0.2058	205.12283	14.88075	3.8076
2	18.045	BB	0.3213	5182.03369	245.47847	96.1924

Totals : 5387.15652 260.35922

===
*** End of Report ***

Supplementary Fig. 301. HPLC spectra for compound (±)-**5h-D**

Supplementary References

[1] Berk, S. C., Kreutzer, K. A. & Buchwald, S. L. A catalytic method for the reduction of esters to alcohols. *J. Am. Chem. Soc.* **113**, 5093–5095 (1991).

[2] Berk, S. C. & Buchwald, S. L. An air-stable catalyst system for the conversion of esters to alcohols. *J. Org. Chem.* **57**, 3751–3753 (1992).

[3] Einaru, S., Shitamichi, K., Nagano, T., Matsumoto, A., Asano, K. & Matsubara, S. *trans*-Cyclooctenes as halolactonization catalysts. *Angew. Chem. Int. Ed.* **57**, 13863–13867 (2018).

[4] Benoit, G. & Charette, A. B. Diastereoselective borocyclopropanation of allylic ethers using a boromethylzinc carbenoid. *J. Am. Chem. Soc.* **139**, 1364–1367 (2017).

[5] Chen, Z., Liang, P., Xu, F., Deng, Z., Long, L., Luo, G. & Ye, M. Metal-free aminothiation of alkynes: three-component tandem annulation toward indolizine thiones from 2-alkylpyridines, ynals, and elemental sulfur. *J. Org. Chem.* **84**, 12639–12647 (2019).

[6] Kim, S. W., Wurm, T., Brito, G. A., Jung, W.-O., Zbieg, J. R., Stivala, C. E. & Krische, M. J. Hydroamination versus allylic amination in iridium-catalyzed reactions of allylic acetates with amines: 1,3-aminoalcohols via ester-directed regioselectivity. *J. Am. Chem. Soc.* **140**, 9087–9099 (2018).

[7] Hatakeyama, T., Hashimoto, T., Kondo, Y., Fujiwara, Y., Seike, H., Takaya, H., Tamada, Y., Ono, T. & Nakamura, M. Iron-catalyzed suzuki−miyaura coupling of alkyl halides. *J. Am. Chem. Soc.* **132,** 10674–10676 (2010).

[8] Wang, Y.-F., Gao, Y.-R., Mao, S., Zhang, Y.-L., Guo, D.-D., Yan, Z.-L., Guo, S.-H. & Wang, Y.-Q. Wacker-type oxidation and dehydrogenation of terminal olefins using molecular oxygen as the sole oxidant without adding ligand. *Org. Lett.* **16**, 1610–1613 (2014).

[9] Zhu, D. Lv, L., Li, C.-C., Ung, S., Gao, J. & Li, C.-J. Umpolung of carbonyl groups as alkyl organometallic reagent surrogates for palladium-catalyzed allylic alkylation.

Angew. Chem. Int. Ed. **57**, 16520–16524 (2018).

[10] Chen, F., Xu, X., He, Y., Huang, G. & Zhu, S. NiH-catalyzed migratory defluorinative olefin cross-coupling: trifluoromethyl-substituted alkenes as acceptor olefins to form gem-difluoroalkenes. *Angew. Chem. Int. Ed.* **59**, 5398–5402 (2020).

[11] Cosgrove, S. C., Hussain, S., Turner, N. J. & Marsden, S. P. Synergistic chemo/biocatalytic synthesis of alkaloidal tetrahydroquinolines. *ACS Catal.* **8**, 5570–5573 (2018).

[12] Álvarez-Calero, J. M., Jorge, Z. D. & Massanet, G. M. $TiCl_4/Et_3N$-mediated condensation of acetate and formate esters: direct access to β-alkoxy- and β-aryloxyacrylates. *Org. Lett.* **18**, 6344–6347 (2016).

[13] Taeufer, T. & Pospech, J. Palladium-catalyzed synthesis of N,N-dimethylanilines via Buchwald–Hartwig amination of (hetero)aryl triflates. *J. Org. Chem.* **85**, 7097–7111 (2020).

[14] Liu, X., Liu, B. & Liu, Q. Migratory hydrogenation of terminal alkynes by base/cobalt relay catalysis. *Angew. Chem. Int. Ed.* **59**, 6750–6755 (2020).

[15] Chen, Z.-M., Liu, J., Guo, J.-Y., Loch, M., DeLuca, R. J. & Sigman, M. S. Palladium-catalyzed enantioselective alkenylation of alkenylbenzene derivatives. *Chem. Sci.* **10**, 7246–7250 (2019).

[16] He, J., Song, P., Xu, X., Zhu, S. & Wang, Y. Migratory reductive acylation between alkyl halides or alkenes and alkyl carboxylic acids by nickel catalysis. *ACS Catal.* **9**, 3253–3259 (2019).

[17] He, Y., Cai, Y. & Zhu, S. Mild and regioselective benzylic C–H functionalization: Ni-catalyzed reductive arylation of remote and proximal olefins. *J. Am. Chem. Soc.* **139**, 1061–1064 (2017).

[18] Shimogaki, M., Fujita, M. & Sugimura, T. Metal-free enantioselective oxidative arylation of alkenes: hypervalent-iodine-promoted oxidative C−C bond formation. *Angew. Chem. Int. Ed.* **55**, 15797–15801 (2016).

[19] Liu, H., Xu, M., Cai, C., Chen, J., Gu, Y. & Xia, Y. Cobalt-catalyzed Z to E isomerization of alkenes: an approach to (E)-β-substituted styrenes. *Org. Lett.* **22**, 1193–1198 (2020).

[20] He, Y., Liu, C., Yu, L. & Zhu, S. Enantio- and regioselective NiH-catalyzed reductive hydroarylation of vinylarenes with aryl iodides. *Angew. Chem. Int. Ed.* **59**, 21530–21534 (2020).

[21] Pospíšil, J. & Markó, I. E. Efficient and stereoselective synthesis of allylic ethers and alcohols, *Org. Lett.* **8**, 5983–5986 (2006).

[22] Zhang, C., Liu, P.-X., Huang, L.-Y., Wei, S.-P., Wang, L., Yang, S.-Y., Yu, X.-Q., Pu, L. & Wang, Q. Engineering P450 peroxygenase to catalyze highly enantioselective epoxidation of cis-β-methylstyrenes. *Chem. Eur. J.* **22**, 10969–10975 (2016).

[23] Xu, C., Shen, X. & Hoveyda, A. H. In situ methylene capping: a general strategy for efficient stereoretentive catalytic olefin metathesis. the concept, methodological implications, and applications to synthesis of biologically active compounds. *J. Am. Chem. Soc.* **139**, 10919–10928 (2017).

[24] Dydio, P., Detz, R. J., de Bruin, B. & Reek, J. N. Beyond classical reactivity patterns: hydroformylation of vinyl and allyl arenes to valuable β- and γ-aldehyde intermediates using supramolecular catalysis. *J. Am. Chem. Soc.* **136**, 8418−8429 (2014).

[25] Niharika, P. & Satyanarayana, G. [Pd]-catalyzed intermolecular coupling and acid mediated intramolecular cyclodehydration: one-pot synthesis of indenes. *Eur. J. Org. Chem.* **2018**, 971–979 (2018).

[26] Kim, H. & Lee, C. A mild and efficient method for the stereoselective formation of C−O bonds: palladium-catalyzed allylic etherification using zinc(II) alkoxides. *Org. Lett.* **4**, 4369–4371 (2002).

[27] Mikhaylov, A. A., Dilman, A. D., Novikov, R. A., Khoroshutina, Y. A.,

Struchkova, M. I., Arkhipov, D. E., Nelyubina, Y. V., Tabolin, A. A. & Ioffe, S. L. Tandem Pd-catalyzed C–C coupling/recyclization of 2-(2-bromoaryl)cyclopropane-1,1-dicarboxylates with primary nitro alkanes. *Tetrahedron Lett.* **57**, 11–14 (**2016**).

[28] Bejot, R., Anjaiah, S., Falck, J. R. & Mioskowski, C. α-Haloenol acetates: versatile reactants for oxetan-2-one, azetidin-2-one and isoxazolidin-5-one synthesis. *Eur. J. Org. Chem.* **2007**, 101–107 (2007).

[29] Ruan, J., Li, X., Saidi, O. & Xiao, J. Oxygen and base-free oxidative heck reactions of arylboronic acids with olefins. *J. Am. Chem. Soc.* **130**, 2424–2425 (2008).

[30] Lundgren, R. J. & Thomas, B. N. Chemo- and regioselective reductive transposition of allylic alcohol derivatives via iridium or rhodium catalysis. *Chem. Commun.* **52**, 958–961 (2016).
